# Identification and functional characterization of the German cockroach, *Blattella germanica*, short interspersed nuclear elements

**DOI:** 10.1371/journal.pone.0266699

**Published:** 2022-06-13

**Authors:** Sergei Yu. Firsov, Karina A. Kosherova, Dmitry V. Mukha

**Affiliations:** Vavilov Institute of General Genetics Russian Academy of Sciences, Moscow, Russia; University of Bari: Universita degli Studi di Bari Aldo Moro, ITALY

## Abstract

In recent decades, experimental data has accumulated indicating that short interspersed nuclear elements (SINEs) can play a significant functional role in the regulation of gene expression in the host genome. In addition, molecular markers based on SINE insertion polymorphisms have been developed and are widely used for genetic differentiation of populations of eukaryotic organisms. Using routine bioinformatics analysis and publicly available genomic DNA and small RNA-seq data, we first described nine SINEs in the genome of the German cockroach, *Blattella germanica*. All described SINEs have tRNA promoters, and the start of their transcription begins 11 bp upstream of an “A” box of these promoters. The number of copies of the described SINEs in the *B*. *germanica* genome ranges from several copies to more than a thousand copies in a SINE-specific manner. Some of the described SINEs and their degenerate copies can be localized both in the introns of genes and loci known as piRNA clusters. piRNAs originating from piRNA clusters are shown to be mapped to seven of the nine types of SINEs described, including copies of SINEs localized in gene introns. We speculate that SINEs, localized in the introns of certain genes, may regulate the level of expression of these genes by a PIWI-related molecular mechanism.

## Introduction

Short interspersed nuclear elements (SINEs) are nonautonomous retrotransposons transcribed by RNA polymerase III. The conversion of SINE RNA into DNA and the subsequent process of integration into different locations of the genome are controlled by the molecular machinery of autonomous retrotransposons [1–4]. Similar to other transposable elements (TEs), SINEs are ancient components of the genome [5], although young and highly active families of these TEs have also been described [6]. Interestingly, SINEs may account for a significant portion of the genome in some eukaryotic species but are absent in others [1, 4, 7].

Typical SINEs are 150–600 bp in length with a composite structure consisting of three parts: "head", "body" and "tail", sequentially localized starting from the 5’- end of the TE. “The head” is a fragment of one of the types of RNAs transcribed by RNA polymerase III: tRNA, 5S, or 7SL. Usually, SINEs contain tRNA fragments [8, 9]. Some SINEs may contain fragments of several RNAs of one type [10, 11] and combinations of different RNAs, for example, tRNA + 7SL [12, 13] or tRNA + 5S [14, 15]. A fundamental aspect of the functional activity of a SINE is the presence of a promoter in this part of TE, which is recognized by RNA polymerase III, which, in turn, initiates the start of transcription upstream from the promoter location. To date, the features of the structural and functional organization of the promoters of the tRNA, 5S, and 7SL RNA genes have been described in detail [16, 17]. In particular, the transcription of eukaryotic tRNA genes has been shown to use a promoter system comprised of an “A” box and a “B” box (length of 11 bp each), variably spaced from one another by ~30–60 bp in a gene-specific manner [18–21]. The initiation of transcription typically begins ~7–20 bp upstream of the “A” box promoter element [16, 18, 20].

The main structural and functional component of "the body" of SINEs is the relatively extended region responsible for the binding of the protein product of the "partner" autonomous retrotransposons with the SINE RNA and subsequent reverse transcription of this RNA and SINE integration into the genome. Note that it is far from always possible to trace the similarity of the nucleotide sequence between SINE and its autonomous "partner" [2]. Moreover, relatively recently, so-called CORE sequences, which are evolutionarily conserved nucleotide sequences with a high degree of similarity between SINEs described in the genomes of organisms that are evolutionarily distant from each other, have been identified in the “body” of some SINEs. The evolutionary value of these conserved regions is still debated [22–27].

"The tail" of typical SINEs represents repetitive microsatellite motifs or poly (A) sequences. The molecular mechanism of the formation of these sequences remains largely unclear to date.

During the integration of retrotransposons, sequential cutting occurs first at the bottom and then at the upper strand of the target site. Depending on the particular retrotransposon, the second strand break can occur downstream, upstream, or in line with the bottom strand nick, resulting in target site duplications (TSDs), target site deletions or blunt insertions, respectively [28]. Since the integration of SINEs is conditioned by the peculiarities of the partner autonomous retrotransposon, during the integration of nonautonomous retrotransposons, any of the changes described above in the target sites are theoretically possible; however, to the best of our knowledge, only the formation of TSDs has been described thus far.

Whereas transposable elements (including SINEs) have been considered selfish or junk DNA [29–31], recent findings in genomic and epigenomic studies suggest that some of their copies have functional roles in gene regulation and/or chromatin organization. SINEs are assumed to insert in genes, providing new splicing sites resulting in the generation of a SINE-containing isoform; transcription factor binding sites in the SINE sequence may affect neighboring genes; SINEs can regulate gene expression at a distance as a tissue-specific enhancer; SINEs can stabilize nucleosome positioning in neighboring regions; and SINEs can mediate the methylation of the surrounding DNA and mediate histone modifications in the region of the integration site [for review, see 32, 33]. Thus, transposable elements play an important regulatory role in ensuring the functional activity of the genome; in addition, it has been shown that the structural and functional elements that determine the activity of transposable elements can be used to create vector constructs for biotechnological purposes [for review, see 34].

Since the target-site specificity of the integration of SINEs is dictated by the molecular machine of their autonomous partners, it is logical to assume that the distribution of autonomous and nonautonomous retrotransposons in the genome will be similar. However, the retrotransposons in the genome have been shown to occupy distinct parts of the genome, and their regional densities are negatively correlated with each other [32]. SINEs are clustered in gene-rich regions, while their autonomous partners are concentrated in gene-poor regions and are depleted from promoters. It was suggested that positive selection has been operating on SINEs inserted in or close to genes during evolution [35, 36]. In this regard, interesting recent studies show that SINEs have undergone strong natural selection, causing genomic heteroplasmy and driving ecological diversity. Possible evolutionary mechanisms underlying ecological diversity at the interface between SINE mobilization and organism defense have been revealed [37, 38].

Of particular interest, from our point of view, are studies of the role of a recently discovered class of small RNAs in the regulation of the activity of TEs and the epigenetic modification of the integration sites of TEs mediated by these small RNAs. piRNAs (PIWI-interacting RNAs) are the largest class of small noncoding RNAs of 26 to 31 nucleotides in length expressed in germinal and somatic cells; they are found in complexes with proteins of the PIWI family, for which they were named [39]. piRNAs are involved in the control of TEs as part of an evolutionarily conserved mechanism [40–42]. Most of the piRNAs originate from loci known as piRNA clusters. These loci are enriched with inactive transposon sequences and are required to prevent the spread of active TEs throughout the genome. piRNAs from piRNA clusters direct PIWI proteins to cleave TE transcripts and induce their processing into new piRNAs. These new piRNAs themselves can act as PIWI guides to cleave complementary transcripts, inducing the production of piRNAs identical to initiator piRNAs derived from piRNA clusters, leading to the process of amplification of piRNA, which is called the ping-pong cycle [41, 43], reviewed by Ozata et al. [44].

piRNAs have been shown to regulate transpositional activity through RNA decay (posttranscriptional level) and/or through DNA methylation and histone modification (transcriptional level). DNA methylation and histone modification are known to be able to lead to repressive heterochromatin formation and change the dynamics of expression of a gene localized in this region [45–49]. It was shown that DNA methylation could be detected in all insect orders examined except Diptera (flies) [50]. We speculate that SINEs, localized in the introns of certain genes responsible for the performance of specific functions, by PIWI-related molecular mechanisms may change the local conformation of chromatin, which in turn will lead to a change (adjusting?) of the level of expression of these genes.

In recent decades, the German cockroach, *Blattella germanica*, has been increasingly considered a model object for studying the molecular genetic organization of eukaryotes and may serve as a suitable reference model for studying the molecular biology of insects [51–56].

In this study, we first described the structural and functional organization of SINEs of *B*. *germanica*. We examined a representative sample of approximately a thousand genes previously annotated in the genome of the German cockroach and identified the genes containing SINE copies in the introns. Degenerate copies of seven of the nine described SINEs were shown to be localized in piRNA clusters, and the corresponding piRNAs are mapped to copies of SINEs localized in gene introns. We consider the results obtained as the first step toward studying the possible regulatory function of SINEs of *B*. *germanica*.

Finally, since the number of SINEs in the genome, as a rule, is large enough and their integration occurs into random sites of the genome, the pattern of integrated copies of these TEs can be considered a polymorphic molecular genetic marker that allows solving the problems of population genetics, in particular, determining the genetic distances between populations of living organisms.

## Materials and methods

### SINEs identification

Recently, the 2-Gb genome of *B*. *germanica* was reported [54], which gives rise to the possibility of studying the features of the structural organization of SINEs of this insect. We used the sequence data presented in a public database (https://www.ncbi.nlm.nih.gov/Traces/wgs/PYGN01).

To identify SINEs in the *B*. *germanica* genome, we used two methodological approaches: the first approach was based on an automatic search using the previously published program SINE_Scan 1.1.1 [57]; the second approach was based on the development of our own search algorithm.

The local SINE_Scan 1.1.1 package for Linux (https://github.com/maohlzj/SINE_Scan) was used with the default parameters (S1 File).

The essence of our new algorithm is as follows. In the first step, the prediction of tRNA sequences in the *B*. *germanica* genome was performed using the previously published program tRNAscan 2.0.3 [58]. The local tRNAscan 2.0.3 package for 64-bit Linux (http://lowelab.ucsc.edu/tRNAscan-SE/) was used with the default parameters (S1 File).

As a result, the positions of the first nucleotides for each predicted tRNA sequence were determined. The regular Python script (S1 Script), with the possibility of saving fragments with a length of 1000 nucleotides, was used for creation of a database containing the sequences corresponding to determined tRNAs at the 5’-end and an extended region corresponding to the sequence adjacent to tRNA in genomic DNA at the 3’ end. The resulting pool of sequences was analyzed using CodonCode Aligner 8.0.2 software (https://www.codoncode.com/aligner). The "Assemble" command of this software was run with the following parameters of "Assembly" settings tab: Algorithm—Local alignments, Min. percent identity = 90, Min. overlap length = 150, with the following visual analysis of the sequences in "Contigs" folder. The candidates for SINE should have no less than three aligned sequences; the beginning of tRNA should have a shift no more than five nucleotides in length; overlapping sequences should have a length no less than 150 nucleotides (for an example see S1 Fig).

For each of the sequences considered a candidate for SINE, sequences were found in the genome of *B*. *germanica* with the maximum similarity to the analyzed sequence. The local tools from the BLAST 2.9.0+ package for 64-bit Linux (ftp://ftp.ncbi.nlm.nih.gov/blast/executables/blast+/) [59] were used to perform searches with the default parameters, followed by the analysis and filtering of the output table. Only hits with E-values < 1x10^-25^ were used for the following analysis.

At the final stage, based on the comparison of similar nucleotide sequences corresponding to each of the SINE types, consensus sequences were predicted using the Jalview V.2 program [60].

To identify evolutionarily conserved sequences within the SINEs we described, we used two approaches: 1) the CENSOR program [61, 62], available at https://www.girinst.org/censor/index.php, and 2) regular online Blastn search, which allowed to compare the sequences we analyzed with all the sequences presented in the GenBank database (https://blast.ncbi.nlm.nih.gov/Blast.cgi?PROGRAM=blastn&PAGE_TYPE=BlastSearch&LINK_LOC=blasthome)

The duplications of the integration sites were predicted online by TSD (target site duplication) Search program (https://sines.eimb.ru).

The number of copies of each of the SINE types and the coordinates of each specific copy in the genome of *B*. *germanica* were determined using the DotPlot ability in the UGENE 34.0 software package [63]. The following settings were used: identity = 90% or 80% and min. length = from the first to the last nucleotide.

### SINE piRNA analysis

A publicly available small RNA-seq database (https://www.ncbi.nlm.nih.gov/gds/?term=GEO%20GSE87031) was used for analysis. To detect the piRNAs localized in piRNA clusters, we used previously described methodological approaches [52, 53, 55] with minor changes (S2 File).

Low-quality reads were filtered out, and adapters were removed from the 22 small RNA-Seq libraries of *B*. *germanica* using Trimmomatic 0.39 software [64]. Read pairs were then merged using the Pear 0.9.11 tool [65]. Clusters of piRNAs were identified using proTRAC 2.4.3 [66] with the default parameters (S1 File).

As a result, the sequences of piRNA clusters and unique piRNA sequences with a length of 26–31 bases located in piRNA clusters were obtained.

To create a database containing all piRNA repeating reads corresponding to the sequence of unique piRNA reads, the unique piRNA sequences were compared with the pool of reads presented in the original database, from which a new database was obtained containing all piRNA repeating sequences by definition presented in piRNA clusters.

Since the copies of each type of SINE localized in the genome may differ from each other and the corresponding consensus sequence due to the accumulation of random mutations, we created a database containing all copies of Sbg1-Sbg9 localized in the genome, and the degree of similarity between the sequences of each SINE type accounted for 80% or more. Moreover, tRNA sequences, which are part of both SINEs and TE-independent tRNA clusters, are known to be able to be a source of multiple short RNAs, which are products of the PIWI-independent pathway [67, 68]. For this reason, the sequences of SINEs included in the created database did not contain sequences complementary to tRNA. piRNAs localized in piRNA clusters were mapped to the described SINE sequences using Bowtie2 software [69], forcing zero mismatches on the read length; then, this piRNA fraction was retrieved, and the accumulated pool of piRNAs (9261 reads) was used for subsequent analysis (S2 Fig).

The mapping of the described pool of SINE-related piRNAs (9261 reads) to SINEs consensus sequences and genes of *B*. *germanica* was performed by Bowtie2 software, and the result was visualized by the UGENE 34.0 program [63].

## Results and discussion

### Structural and functional organization of SINEs of *Blattella germanica*

In our study, sequences referred to SINE had to meet the following criteria: 1) had a length of at least 150 bp; 2) contained a sequence corresponding to the RNA polymerase III promoter; 3) were represented in the genome by at least three copies; and 4) were flanked by target site duplications.

We identified nine SINEs of *B*. *germanica* (Sbg1 –Sbg9). The lengths of the identified SINEs were Sbg1–306 bp, Sbg2–313 bp, Sbg3–613 bp, Sbg4–456 bp, Sbg5–582 bp, Sbg6–359 bp, Sbg7–564 bp, Sbg8–355 bp, and Sbg9–658 bp.

To identify SINEs, we used two methodological approaches (see “Materials and Methods”). We first used the automatic search of SINEs by SINE_Scan 1.1.1 program which is considered to be fast and robust, and found six SINEs. Next, to double-check the results, we used our own search algorithm and found the same six and three additional SINEs, Sbg4, Sbg5 and Sbg9 that went unrecognized by the initial SINE_Scan 1.1.1 program.

**Fig 1:**
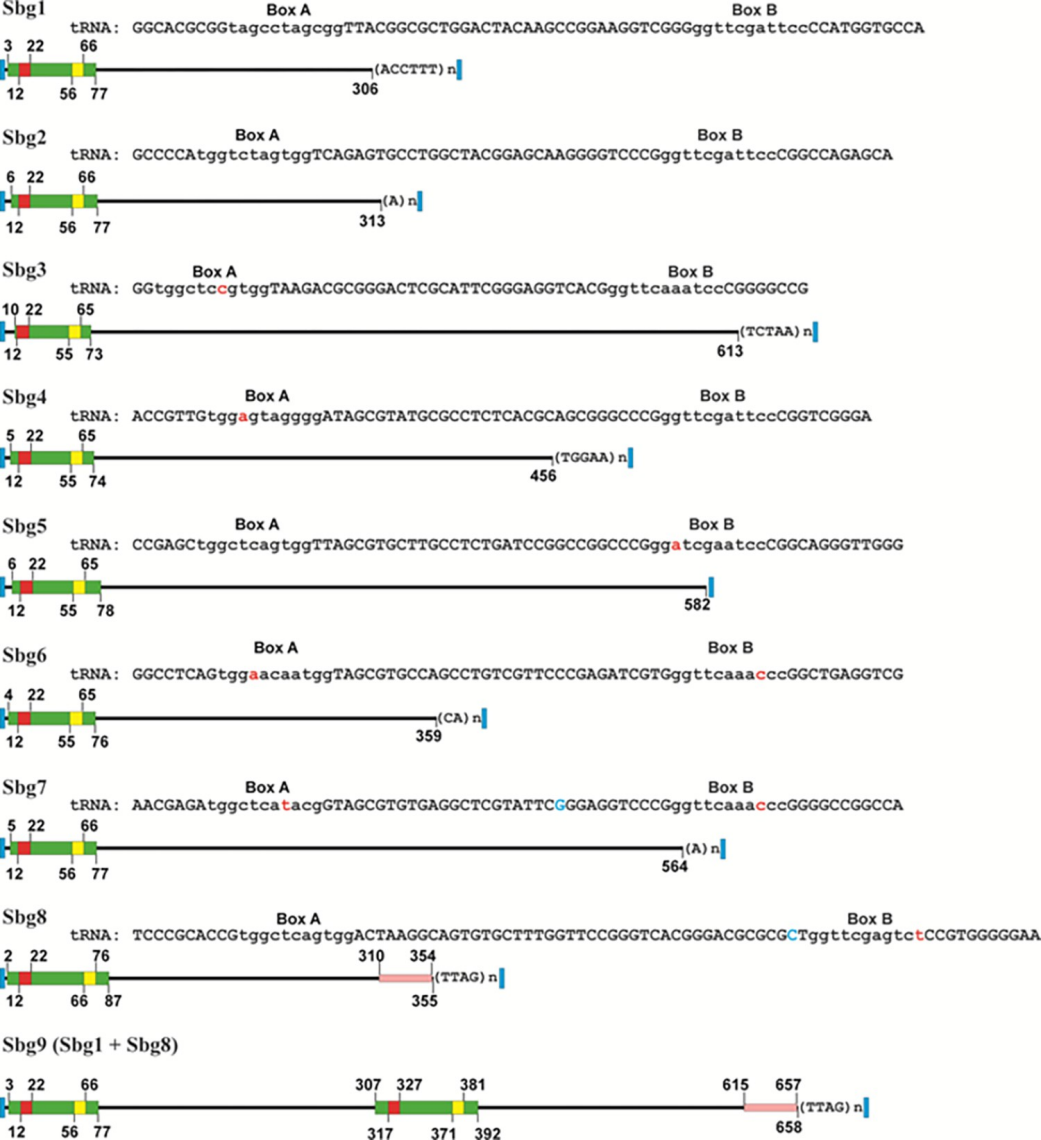
Schemes of the structural organization of the consensus sequences of SINEs of *Blattella germanica* (Sbg1-Sbg9). Areas corresponding to the following nucleotide sequences are highlighted with a colored background: tRNA-green; “A” box- red; “B” box- yellow; the region similar to the nonLTR retrotransposon-pink; target site duplication- blue. Repetitive microsatellite motifs are indicated in parentheses. “A” and “B” boxes are shown in lower case within the tRNA nucleotide sequences. Nucleotides other than canonical nucleotides are highlighted in red and blue (explanation in the text). The numbers indicate the positions of the nucleotides.

In Fig 1, the schemes of the structural organization of the consensus sequences of the SINEs identified by us are shown; in S3 Fig—both the consensus sequences and the sequences of twelve similar to the consensus sequence SINE copies, presented in the genome, along with the sequences of the nearest environment are shown. Note that Sbg9 is a dimeric SINE composed of the Sbg1 and Sbg8 sequences (see S3 Fig).

The “heads” of all identified SINEs are represented as tRNA fragments. For six SINEs (Sbg1 –Sbg6), the tRNAscan 2.0.3 program [58] predicted tRNA sequences in the consensus sequences of SINEs; however, in the consensus sequences of Sbg7 and Sbg8, the tRNA sequences were not predicted. To determine the nature of Sbg7 and Sbg8 “heads”, we analyzed by the tRNAscan 2.0.3 program all copies of SINEs identified in the genome of *B*. *germanica* that have at least 90% similarity both with each other and with the consensus sequences corresponding to Sbg7 or Sbg8. The consensus sequences of Sbg7 and Sbg8 were shown to have one nucleotide substitution each (in Figs 1 and S3 highlighted in blue and red fonts, respectively), distinguishing them from the canonical tRNA structures identified by the tRNAscan 2.0.3 program (S3 Fig). Note that some copies of both Sbg7 and Sbg8 contained tRNA sequences that were detectable by the tRNAscan 2.0.3 program.

The transcriptional mechanism of eukaryotic tRNA genes is known to use a promoter system comprised of an “A” box and a “B” box, which have the following canonical nucleotide compositions: TRGYNNARNNG and RGTTCRANTCC, respectively [70]. An “A” box and a “B” box identified in the consensus sequences of Sbg1 and Sbg2 have canonical structures (in Figs 1 and S3 “A” and “B” boxes highlighted in lowercase letters). All other SINEs identified in the genome of *B*. *germanica* have one nucleotide substitution within the “A” or/and “B” boxes (in Figs 1 and S3, the substitutions highlighted in red fonts and yellow background, respectively).

The SINEs we have described have a different number of nucleotides from their 5’-end to the beginning of the tRNA sequence detected by the tRNAscan 2.0.3 program; at the same time, the distance from the beginning of SINEs to the start of the corresponding “A” box was always 11 bp (Figs 1 and S3). We call it the "eleven nucleotide rule", indicating that the start of transcription of all we have described SINEs begins 11 bp upstream of an “A” box of RNA polymerase III promoter.

As mentioned above, the initiation of transcription of eukaryotic tRNA genes typically begins ~7 to 20 bp upstream of the “A” box promoter element in a species-specific manner. Whether the "eleven nucleotide rule" applies to all tRNA genes of *B*. *germanica*, or is characteristic only for SINEs of this insect species remains unclear and will be the subject of a separate study.

For SINEs of other species presented in the RepBase (https://www.girinst.org) [8] and SINE BASE (https://sines.eimb.ru) [9] databases, the distances from the start of transcription sites to the corresponding “A” boxes range widely (data not shown). In addition, it was shown that in *Reticulitermes lucifugus*, a closely related species of *B*. *germanica*, these distances are different for SINEs Talua, Talub, Taluc and Talud (11 bp, 10 bp, 11 bp and 5 bp, respectively; S4 Fig).

The structure of “the body” of *B*. *germanica* SINEs was investigated by comparing their nucleotide sequences with previously described TEs of various species presented in the following databases: RepBase [8], SINE BASE [9], and NCBI (https://www.ncbi.nlm.nih.gov) using the program CENSOR [61, 62] and regular online Blastn search.

We were unable to identify any new or previously described CORE sequences. For most of the SINEs of *B*. *germanica*, sequences similar to the sequences of autonomous retrotransposons, potential partners of the studied SINEs, were also not identified. The exceptions are Sbg8 and Sbg9, which contain the same sequences as the sequences of the *Locusta migratoria* RTE retrotransposon (Figs 1 and S5). Note that Sbg9 is a dimeric SINE containing the Sbg8 nucleotide sequence.

"The tails" of Sbg2 and Sbg7 represent poly(A) sequences, whereas all other *B*. *germanica* SINEs contained short tandem repeats (STRs) in this structural part of retrotransposons. Interestingly, each of the Sbg1, Sbg4, Sbg6, Sbg8, and Sbg9 copies identified in the *B*. *germanica* genome contains perfect microsatellite motifs: “accttt”, “tggaa”, “ca”, “ttag”, and “ttag”, respectively. However, only a few copies of Sbg5 contained the motif “tcaga”. Sbg3 copies contain different repeat variants, apparently representing different variants of duplications of short nucleotide sequences of the parent SINE copy (Figs 1 and S3).

The molecular mechanisms responsible for changes in the lengths of STRs have been the subject of study over the past several decades since the discovery of these structural formations in eukaryotic genomes. The main mechanism leading to an increase/decrease in the number of repeats in the STR locus is now generally accepted to be slipped strand mispairing that occurs during genomic DNA replication [71]. The number of STRs in SINE copies integrated into the genome can change according to this mechanism. At the same time, in the process of transpositions of retrotransposons, in particular SINEs, there is an additional stage at which duplications can occur, namely, the synthesis of a cDNA copy of TE, due to the activity of the reverse transcriptase enzyme. At this stage, erroneous reinitiation of the start of cDNA synthesis may occur. The presence of 3’-end repeats of different lengths and nucleotide compositions in different Sbg3 copies (S3 Fig) might be an indication that secondary initiation of synthesis of cDNA of a TE copy may be the primary cause of STR formation in SINEs.

All copies of Sbg1-Sbg9 retrotransposons identified in the genome of *B*. *germanica* had duplications of the integration site, represented by direct repeats 10–15 bp long, flanking the retrotransposons (Figs 1 and S3). Comparison of the nucleotide sequences of direct repeats flanking various copies of each of the described types of SINEs integrated into the genome did not reveal any peculiarities of their nucleotide composition, which indicates the sequence-independent (random) type of integration of these TEs.

It is known that in the process of transposition of TEs in the genome of eukaryotic organisms, the number of their copies increases; however, due to the accumulation of random mutations, a part of the TE copies integrated into the genome turns into degenerate copies that have lost the ability to transposition. Obviously, the greater the percentage of degenerate copies relative to the total number of copies of a given TE type, the greater the evolutionary age of a particular TE.

**Fig 2:**
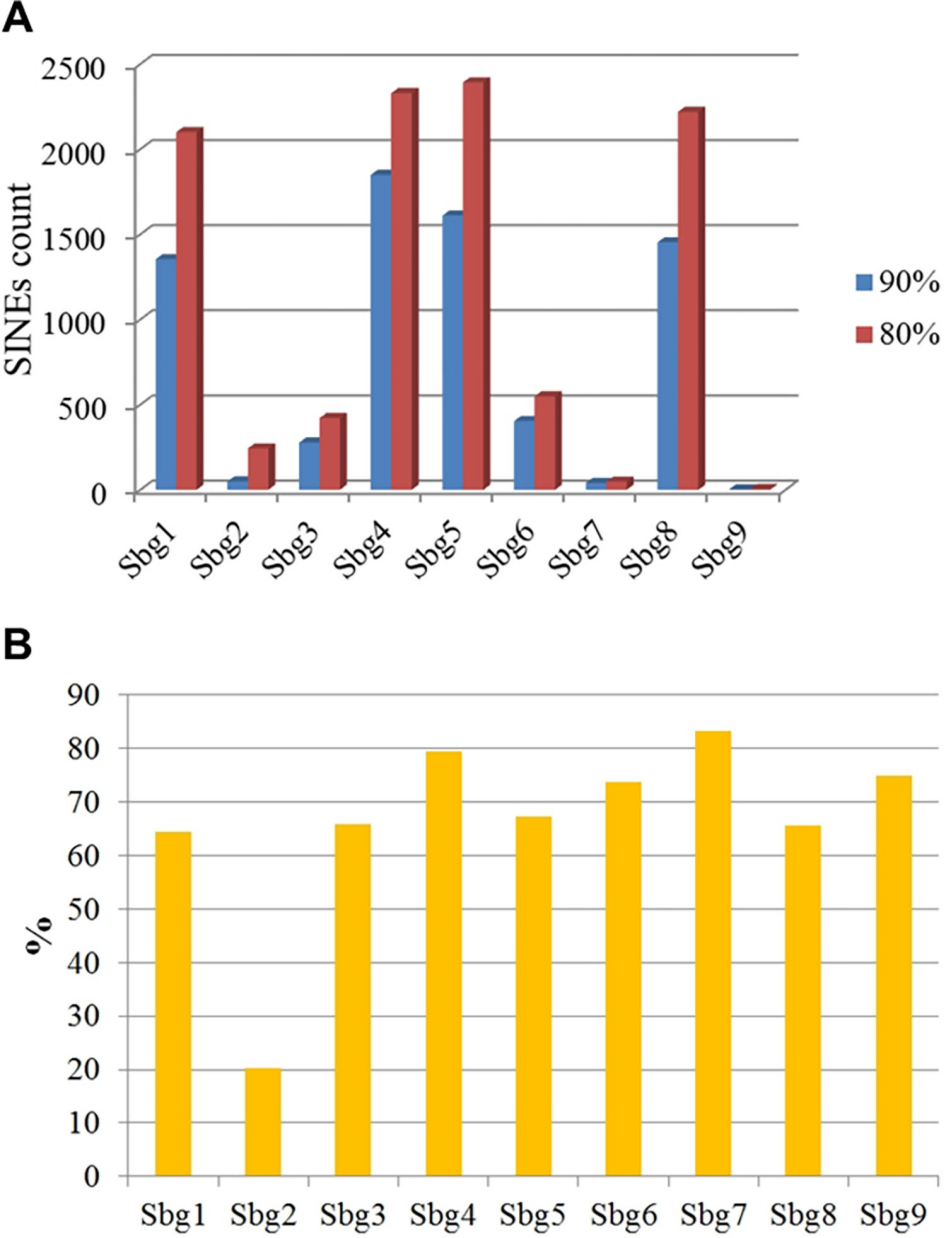
Analysis of the copy number of the described SINEs. (**A)** The ratio of the copy numbers of SINEs of each type (Sbg1-Sbg9) having different degrees of similarity in relation to their consensus sequences: blue and red bars-90% or more and 80% or more similarity, respectively. The abscissa is the SINE type; the ordinate is the number of SINE copies; (**B)** For each type of SINE, proportion of copies with the highest (90% or more) similarity to consensus sequences among copies with 80% or more similarity. The abscissa is the SINE type; the ordinate shows the percentage.

To determine the number of copies of described SINEs, the consensus sequences of each of the SINEs were compared with the nucleotide sequence of the *B*. *germanica* genome at the given parameters—90% and 80% similarity over the entire length of the SINE consensus sequence. This approach allows the determination of both the number of copies that are most similar to the consensus sequence and the percentage of relatively degenerate copies. The absolute values of the number of copies of the studied SINEs and graphical representations of their distribution in the genome are presented in S3 Fig; Fig 2A is a graph showing the copy number variability of each type of SINE depending on the degree of similarity with consensus sequences.

In general, the number of copies varies considerably depending on the SINE type: from 3 (Sbg9) to 1849 (Sbg4) copies with 90% or more similarity to consensus sequences. The number of "degenerate" copies, reflecting the evolutionary age of a particular SINE type, also varies widely depending on the SINE type (Fig 2). Fig 2B presents a diagram showing the proportion of copies with the highest (90% or more) similarity to consensus sequences among copies with 80% or more similarity. The copies of Sbg2 were shown to have the highest level of DNA sequence variability, demonstrating that they are among the oldest SINEs present in the *B*. *germanica* genome. Based on the described criterion for assessing the evolutionary age of SINEs, we should conclude that the most evolutionarily young variants of SINEs represented in the *B*. *germanica* genome are Sbg4, Sbg7, and Sbg9 (Fig 2B).

SINEs can be subdivided into distinct subfamilies by specific diagnostic nucleotide changes. Older subfamilies are generally very abundant, while younger subfamilies have fewer copies [1, 4]. Note that we were unable to identify any subfamilies within the SINEs described by us.

Sbg9 deserves special consideration. This TE is a dimeric SINE formed by the combination of two SINEs: Sbg1 and Sbg8. Apparently, among the SINEs described by us, Sbg9 is the most evolutionarily young, formed relatively recently, and, probably, for this reason it is represented in the genome by such a small number of copies. Note that dimeric SINEs are quite widespread in the described genomes of living organisms [for review, see 1, 4].

From our point of view, the SINEs described in the genome of the German cockroach, *B*. *germanica*, may have not only general scientific value but also applied value. *B*. *germanica* is a synanthropic species of organisms that can live both in human residential premises and in public institutions and on livestock farms. The spectrum of the negative influence of these insects on human life is unusually wide. A particular danger is the ability of these insects to be carriers of pathogenic microorganisms and cause severe allergic diseases in humans [72–75]. In this regard, understanding the structure of *B*. *germanica* populations and the possibility of determining the migration flows of this insect species are of particular importance. Currently, several types of molecular genetic markers are used to solve the problems of population genetics of *B*. *germanica*: polymorphism of the ribosomal RNA gene cluster [76]; polymorphism of the length of microsatellite loci [77–79]; pattern of 5’-truncated copies of R2 retrotransposon [80]. Despite the rather high resolution of the methods used, the development of new molecular genetic markers remains relevant.

SINEs are represented in the genomes by many copies, and the transposition of young copies occurs constantly at a certain level. Based on these properties, we can assume that SINEs can be considered unique informative molecular genetic markers that make it possible to differentiate populations of eukaryotic organisms. Indeed, at present, this type of marker is actively used for these purposes [for example, see 81–87]. In this study, we first described the structure of SINEs of the German cockroach, *B*. *germanica*. Over a thousand copies of some types of SINEs were shown to be present in the genome of *B*. *germanica*. We can assume that, as has been shown for other species of organisms studied in this regard, the SINEs described by us can be used to analyze the polymorphism of the integration sites of these TEs and, as a consequence, to develop a new type of molecular genetic marker that allows differentiation of populations of *B*. *germanica*. However, it is obvious that the resolution of the proposed markers must be verified experimentally.

### piRNA and SINEs of *B*. *germanica*

piRNAs play an important role in the control of the transpositional activity of mobile elements [39–49]. Do piRNAs take part in the regulation of the transposition activity of the SINEs of *B*. *germanica*?

**Fig 3:**
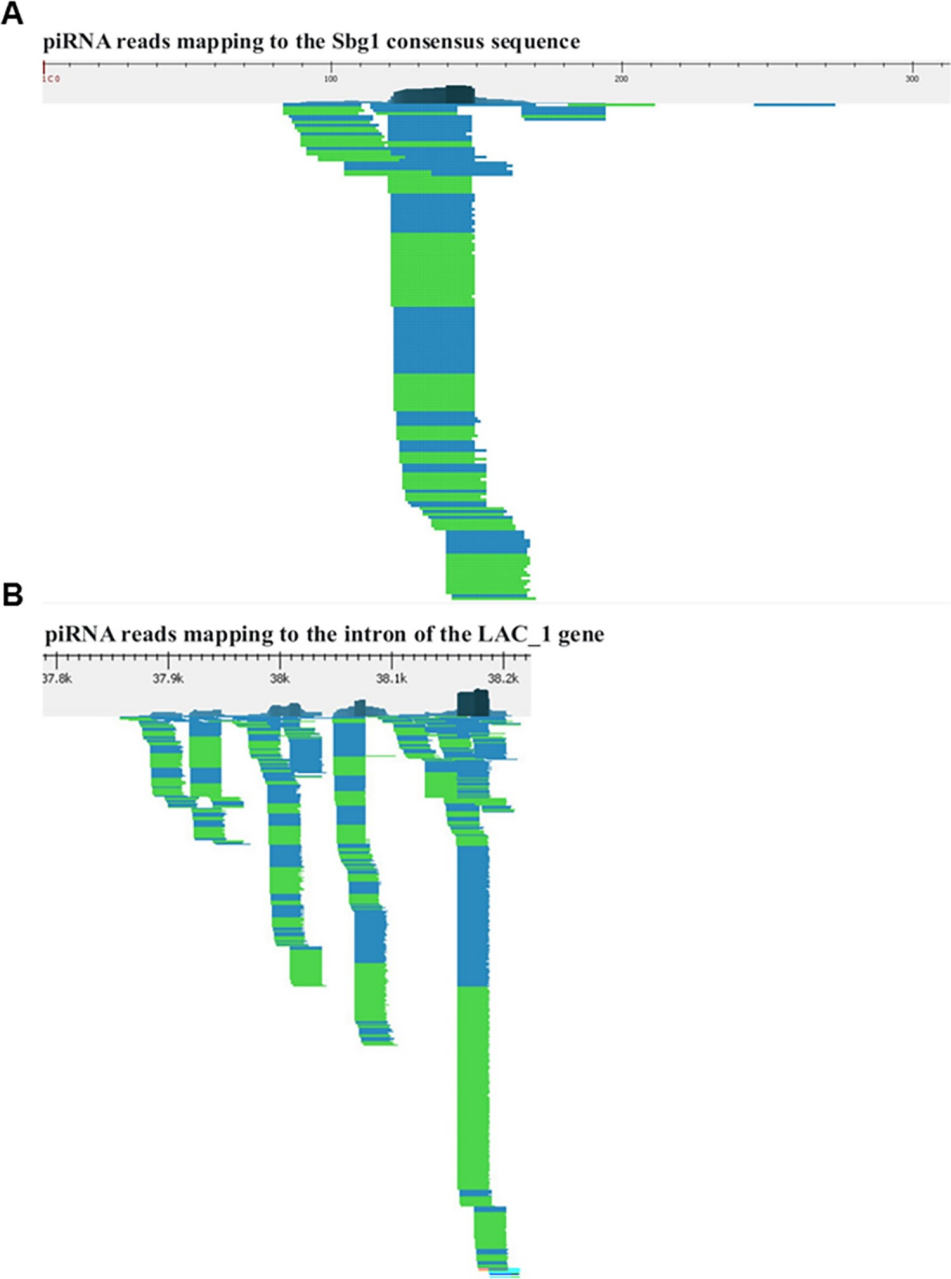
The result of piRNA reads mapping to the (A) Sbg1 consensus sequence; (B) intron of the LAC_1 gene. Reads mapped in direct orientation are highlighted in blue, and reads mapped in reverse complement orientation are green.

To address this question, we checked if piRNAs sequences would map to the SINEs of *B*. *germanica*. Fig 3A shows the result of piRNAs mapping to the Sbg1 consensus sequence (205 reads); S6 Fig shows the result of the piRNA reads mapping to the Sbg1, Sbg3-Sbg6, and Sbg8 consensus sequences. In addition, S6 Fig shows the exact number of piRNA reads mapped and the relative value of the number of reads, reflecting the number of reads per 100 bases of SINE sequence per one thousand mapped reads. Overall, mapping of piRNA reads to the consensus sequences of SINEs of *B*. *germanica* showed that two of the nine SINEs described by us (Sbg3 and Sbg7) do not contain nucleotide sequences that are complementary to piRNAs selected in the above-described way; at the same time, from 46 (Sbg2) to 2290 (Sbg4) piRNA reads were mapped to the consensus sequences of the remaining SINEs. The results obtained indicate that the transposition activity of at least some of the SINEs described by us could be regulated by piRNAs.

As noted above, piRNAs can regulate the transpositional activity of TEs not only through RNA decay but also through DNA methylation and histone modification of TE sites. DNA methylation and histone modification lead to a change in chromatin conformation and cause a change in the dynamics of transcription of a specific TE [44–49]. Obviously, in the case of localization of TEs in gene introns, a change in the chromatin conformation in a given region of the genome can lead to a change in the level of transcription of the gene in the introns of which the TE is located.

Are SINEs (or their degenerate copies) localized in the introns of *B*. *germanica* genes, and, if so, are piRNAs, potentially capable of causing local changes in chromatin conformation, mapped to these SINE copies?

To search for answers to these questions, we identified all genes localized in the first hundred contigs of the *B*. *germanica* genome, for which the potential functional activity has been determined and for which the exon-intron structure has been described. A total of 801 genes were identified. Mapping of piRNAs (included in the above-described pool of 9261 piRNA reads–S2 Fig) to these genes showed that 100 or more piRNA reads were localized within each of 241 genes, and more than 1000 piRNA reads were localized within each of 100 genes. Note that all piRNAs were mapped only within the introns of the genes described. We did not find any piRNA cluster sequences within the introns of the genes described, only fragments of degenerate copies of SINEs were found in the introns described. Table 1 shows the results of the analysis of 20 genes to which the largest number of piRNA reads was mapped; S1 Table shows the result of the analysis of 100 genes, for each of which 1000 or more piRNA reads were mapped. The names of the genes, their coordinates in the genomic DNA sequence, the number of mapped piRNA reads are presented in Table 1 and S1 Table. For example, 2978 piRNA reads were mapped to the introns of the LAC_1 gene; Fig 3B shows the fragment of the distribution of piRNA reads within this gene.

**Table 1:** The result of the analysis of twenty genes to which the largest number of SINE-related piRNA reads was mapped.

Contig ID	Strand	Start	End	Gene ID	Gene full name	Reads count
PYGN01000005	R	832095	893324	C1TC	C-1-tetrahydrofolate synthase	3557 (F—1798; R—1759)
PYGN01000073	F	440373	516715	LAC_1	Lachesin	2978 (F—1450; R—1528)
PYGN01000033	F	355625	439785	Tdc-1_0	Tyrosine decarboxylase	2889 (F—1547; R—1342)
PYGN01000033	F	656364	694669	K11H3-3	Putative tricarboxylate transport protein	2717 (F—1343; R—1374)
PYGN01000022	F	2406772	2456620	lhx3	LIM/homeobox protein Lhx3	2716 (F—1396; R—1320)
PYGN01000072	R	549086	581815	Diap1	Death-associated inhibitor of apoptosis 1	2655 (F—1375; R—1280)
PYGN01000005	R	2480629	2498464	Or88	Odorant receptor 88	2488 (F—1127; R—1361)
PYGN01000049	R	1701575	1765331	Cher_2	Filamin-A	2478 (F—1267; R—1211)
PYGN01000083	R	266347	296944	Pex1	Peroxisome biogenesis factor 1	2469 (F—1167; R—1302)
PYGN01000050	R	461156	514648	Ir76b	Ionotropic receptor 76b	2433 (F—1150; R—1283)
PYGN01000086	R	1254016	1267262	SCD	Acyl-CoA desaturase	2416 (F—1247; R—1169)
PYGN01000005	F	3153139	3200419	IFT140	Intraflagellar transport protein 140	2414 (F—1183; R—1231)
PYGN01000060	F	519801	563025	APN1_0	Aminopeptidase N	2400 (F—1127; R—1273)
PYGN01000016	F	1455716	1479561	Ir41a12	Ionotropic receptor 41a12	2290 (F—1109; R—1181)
PYGN01000056	F	343658	370413	Rcbtb1	RCC1 domain-containing protein 1	2290 (F—1181; R—1109)
PYGN01000029	R	1139008	1166057	March6	E3 ubiquitin-protein ligase MARCH6	2104 (F—1032; R—1072)
PYGN01000024	R	2118061	2140175	CCND2	G1/S-specific cyclin-D2	2081 (F—1089; R—992)
PYGN01000005	F	1745668	1812688	Tle4_1	Transducin-like enhancer protein 4	2072 (F—1020; R—1052)
PYGN01000010	F	1604309	1616070	Smg9	Protein SMG9	2055 (F—999; R—1056)
PYGN01000038	F	2126408	2158714	HIRA	Protein HIRA	2047 (F—1051; R—996)

**F** and **R** denote forward and reverse complement orientations, respectively.

Thus, the results obtained show that SINEs of varying degrees of degeneracy relative to their consensus sequences are localized within the introns of some of the genes of *B*. *germanica*. In addition, a significant number of SINE-related piRNAs derived from piRNA clusters are complementary to the preRNA sequences of certain genes and, therefore, are potentially capable of regulating the transcriptional activity of these genes.

In the course of further studies using RNA interference methods, we assume that we will block the synthesis of proteins that make up the PIWI complex and determine the level of the expression of targeted genes in the introns of which the SINEs are located. Note that a similar approach was recently implemented when the role of siRNA in the development of *B*. *germanica* was studied [56].

## Conclusions

In this study, we first described the structural and functional organization of nine SINE types in the German cockroach, *B*. *germanica*, genome. For each type of SINE, the number of copies of varying degrees of degeneracy in the genome of this insect species was determined.

It was shown that the transpositional activity of at least some of the SINEs we have described potentially can be regulated by piRNA. We suggest that the regulation of the transcriptional activity of genes with introns containing SINEs of varying degrees of degeneracy can be determined by DNA methylation and/or histone modifications in certain regions of these introns, followed by the formation of repressive heterochromatin using PIWI-related molecular mechanisms. A simple experimental model was proposed to test this hypothesis.

## Supporting information

S1 File The description of command lines used for bioinformatics analysis. (PDF)

S2 File Detection scheme for SINE-related piRNAs. (PDF)

S1 Script The regular Python script, with the possibility of saving fragments with a length of 1000 nucleotides, used for creation of a database containing the sequences corresponding to determined tRNAs at the 5’-end and an extended region corresponding to the sequence adjacent to tRNA in genomic DNA at the 3’ end. (PY)

S1 Fig The result of alignment of the extended sequences containing one of the variants of the described SINEs. Areas of DNA sequences corresponding to SINEs are highlighted in blue. Gray background–DNA sequences corresponding to the SINE environment and having a low level of similarity. Vertical lines in red, green, black, and bright blue indicate single nucleotide substitutions.
(PDF)

S2 Fig The reads of piRNA from the created database. piRNAs localized in piRNA clusters were mapped to the described SINE sequences; then, this piRNA fraction was retrieved, and the accumulated pool of piRNAs (9261 reads) was used for subsequent analysis.
(PDF)

S3 Fig Nucleotide sequences and structural features of SINEs of *Blattella germanica* (Sbg1-Sbg9). **(A)**–The consensus nucleotide sequence of the corresponding SINE with the designation of the tRNA structure. The tRNA nucleotide sequences are highlighted in gray; “A” and “B” boxes are highlighted in blue; nucleotides other than canonical nucleotides are highlighted in yellow background and red font (explanation in the text). (**B)**–Distribution of the corresponding SINE copies in the *B*. *germanica* genome. Blue vertical lines indicate the positions of SINE localized in direct orientation, red vertical lines—in inverted orientation. (**C)**–The result of the alignment of the consensus sequences and the sequences of twelve similar to the consensus sequence SINE copies presented in the genome, along with the nearest environment. Direct repeats flanking the retrotransposon are highlighted with a green background. Poly(A) sequences and short microsatellite repeats are highlighted in yellow, blue and pink backgrounds. Variable nucleotides are highlighted in red fonts. (**D)**–Alignment of Sbg1, Sbg8 and Sbg9, demonstrating that Sbg9 was formed by combining Sbg1 and Sbg8.
(PDF)

S4 Fig Nucleotide sequences and structural features of four SINEs (Talua. Talub, Taluc and Talud) of *Reticulitermes lucifugus*. The consensus nucleotide sequences of the corresponding SINEs with the designation of the tRNA structure are shown. The tRNA nucleotide sequences are highlighted in gray; “A” and “B” boxes are highlighted in yellow; nucleotides other than canonical are highlighted in red font. The distances from the start of transcription of SINEs to the corresponding “A” boxes are highlighted in pink.
(PDF)

S5 Fig The result of the comparison of the consensus Sbg8 nucleotide sequence with the sequences presented in the RepBase database (https://www.girinst.org). **(A)**–Masked Sbg8 sequence and the local sequence alignment of Sbg8 and RTE retrotransposons of *Locusta migratoria*. **(B)**–Sequence of RTE retrotransposon of *L*. *migratoria*. The sequence fragment similar to the 3’-end of Sbg8 is highlighted in blue.
(PDF)

S6 Fig The result of piRNA reads mapping to the Sbg1, Sbg3 –Sbg6, and Sbg8 consensus sequences. Reads mapped in direct orientation are highlighted in blue, and reads mapped in reverse complement orientation are green.
(PDF)

S1 Table The analysis of 100 genes, for each of which 1000 or more piRNA reads were mapped. **F** and **R** represent forward and reverse complement orientations, respectively.
(PDF)
