# Supplementary material for: Identification and functional characterization of the German cockroach, Blattella germanica, short interspersed nuclear elements
Source: PLoS One. 2022 Jun 13;17(6):e0266699. doi: 10.1371/journal.pone.0266699 (PMC9191728; doi:10.1371/journal.pone.0266699)
Supplement: S1 File — (PDF) [file pone.0266699.s001.pdf]

## The description of command lines used for bioinformatics analysis.

1)

The local SINE_Scan 1.1.1 package for Linux (https://github.com/maohlzj/SINE_Scan) was used with the default parameters. The command line used was as follows: "perl SINE_Scan_folder/SINE_Scan_process.pl -s 123 -o PYGN01 -g Blattella_germanica_genome_folder/PYGN01.fa -d Blattella_germanica_genome_folder/SINE_Scan_temp -z Blattella_germanica_genome_folder/SINE_Scan_final", where "s" is mode of running SINE_Scan, "o" is genome identifier used as prefix of final output files, "g" is the file of genomic sequences, "d" is a working directory in which intermediate files are stored, and "z" is the output folder.

2)

The local tRNAscan 2.0.3 package for 64-bit Linux (http://lowelab.ucsc.edu/tRNAscan-SE/) was used with default parameters. The command line used was as follows: "tRNAscan_folder/tRNAscan-SE Blattella_germanica_genome_folder/PYGN01_all.fasta -o Blattella_germanica_genome_folder/tRNAscan_PYGN01_all_1.txt -f Blattella_germanica_genome_folder/tRNAscan_PYGN01_all_2.txt -a Blattella_germanica_genome_folder/tRNAscan_PYGN01_all_6.txt", where "o", "f" and "a" - are different representations of output data (see program help for details).

3)

Clusters of piRNAs were identified using proTRAC 2.4.3. The command line script set was as follows:

"perl TBr2_length-filter.pl -i All_stages_paired.fa -o All_stages_paired_length-filtered.fa -min 26 -max 31

perl TBr2_collapse.pl -i All_stages_paired_length-filtered.fa

perl TBr2_duster.pl -i All_stages_paired_length-filtered.collapsed

perl sRNAmapper.pl -input All_stages_paired_collapsed_no-dust.fa -genome PYGN01.fa

perl proTRAC.pl -map All_stages_paired_collapsed_no-dust.fa.map -genome PYGN01.fa -nomotif", where "min" is the minimum sequence length (nt), "max" is the maximum sequence length (nt), "genome" is *B. germanica* genome and "nomotif" is the key to off transcription factor binding sites searching.
