# Supplementary figures and images for: Identification and functional characterization of the German cockroach, Blattella germanica, short interspersed nuclear elements
Source: PLoS One. 2022 Jun 13;17(6):e0266699. doi: 10.1371/journal.pone.0266699 (PMC9191728; doi:10.1371/journal.pone.0266699)

## Detection scheme for SINE-related piRNAs

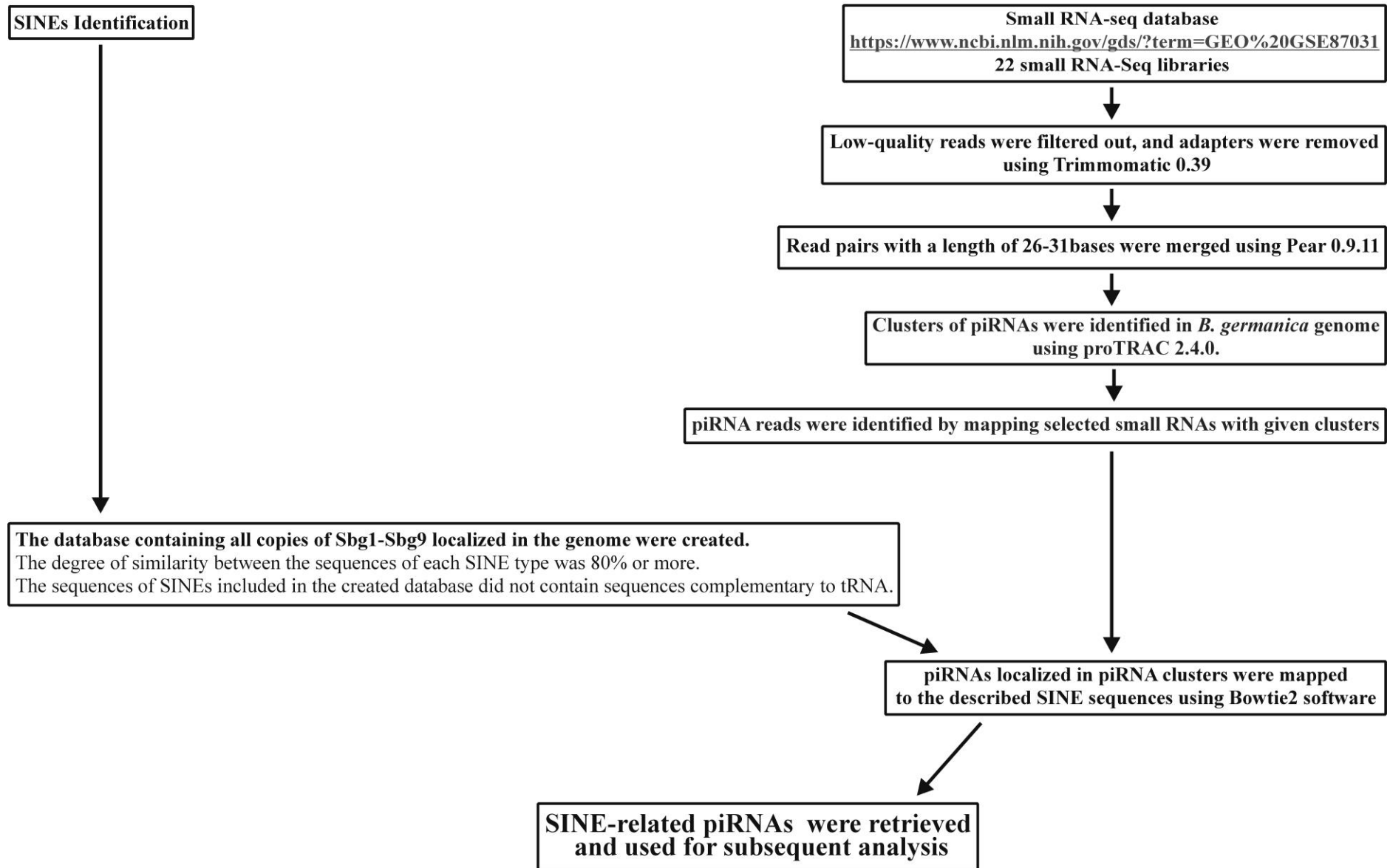

Supplement: S2 File — (PDF) [file pone.0266699.s002.pdf]
