# Supplementary material for: Identification and functional characterization of the German cockroach, Blattella germanica, short interspersed nuclear elements
Source: PLoS One. 2022 Jun 13;17(6):e0266699. doi: 10.1371/journal.pone.0266699 (PMC9191728; doi:10.1371/journal.pone.0266699)
Supplement: S1 Fig — Areas of DNA sequences corresponding to SINEs are highlighted in blue. Gray background–DNA sequences corresponding to the SINE environment and having a low level of similarity. Vertical lines in red, green, black, and bright blue indicate single nucleotide substitutions. (PDF) [file pone.0266699.s004.pdf]

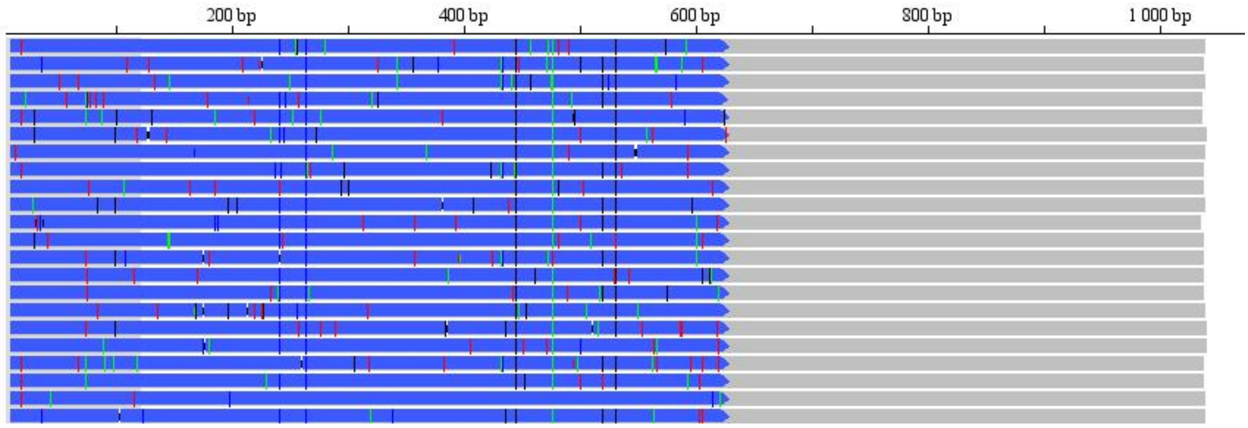

Figure S1. The result of alignment of the extended sequences containing one of the variants of the described SINEs. Areas of DNA sequences corresponding to SINEs are highlighted in blue. Gray background – DNA sequences corresponding to the SINE environment and having a low level of similarity. Vertical lines in red, green, black, and bright blue indicate single nucleotide substitutions.
