# Supplementary material for: Identification and functional characterization of the German cockroach, Blattella germanica, short interspersed nuclear elements
Source: PLoS One. 2022 Jun 13;17(6):e0266699. doi: 10.1371/journal.pone.0266699 (PMC9191728; doi:10.1371/journal.pone.0266699)
Supplement: S2 Fig — piRNAs localized in piRNA clusters were mapped to the described SINE sequences; then, this piRNA fraction was retrieved, and the accumulated pool of piRNAs (9261 reads) was used for subsequent analysis. (PDF) [file pone.0266699.s005.pdf]

Figure S2

The reads of piRNA from the created database.

piRNAs localized in piRNA clusters were mapped to the described SINE sequences; then, this piRNA fraction was retrieved, and the accumulated pool of piRNAs (9261 reads) was used for subsequent analysis.

```
>SRR4252608_11388271___Sbg4
AGGCCCAAACGGTCCCCAGACATCATC
>SRR4252613_6299809___Sbg4
GGTGTCTTTGTCTATATGTTCGTGTTGTC
>SRR4252613_8619832___Sbg4
TTGTCTATATGTTCGTGTTGTCTCGGA
>SRR4252610_12036102___Sbg4
ATCCAGGGAGGCCCTCCATGTCCTTGTGTA
>SRR4252610_10854401___Sbg4
AGGGAGGCCCTCCATGTCCTTGTGTA
>SRR4252615_12639977___Sbg4
ATGTATATGTGATCCATAGTCTATCCTC
>SRR4252610_1143394___Sbg4
TTTGTCTATATGTTCGTGTTGTCTCGGAGGT
>SRR4252615_1783692___Sbg4
TCCAGGGAGGCCCTCCATGTCCTTGTGTA
>SRR4252611_4216134___Sbg4
TTTGTCTATATGTTCGTGTTGTCTCGG
>SRR4252621_11389725___Sbg4
AATGTCAGGAAACTTTAGGCCCAAACGGT
>SRR4252611_6511575___Sbg4
TGTCAGGAAACTTTAGGCCCAAACGGT
>SRR4252607_903620___Sbg4
ACATCATCTGGCCATCATGATCATCTTCA
>SRR4252609_352339___Sbg4
TTGTCTCGGAGGTGGCCCTGGCATTGAGC
>SRR4252610_149461___Sbg4
AGTCATGTAAATAGTACCTACTAGTGGGG
>SRR4252611_9740939___Sbg4
TCCAGGGAGGCCCTCCATGTCCTTGTGTA
>SRR4252611_5694935___Sbg4
TCCTTGTGTAGTCAAAAAAGTATGAATGT
>SRR4252613_7325234___Sbg4
GTCTTTGTCTATATGTTCGTGTTGTC
>SRR4252606_8236053___Sbg4
TTGTCTCGGAGGTGGCCCTGGCATTGAGC
>SRR4252623_14498541___Sbg4
CATCATCTGGCCATCATGATCATCTTCA
>SRR4252607_10924831___Sbg4
CTTCATCATATTCGCCTTGTTGGTATGAGT
>SRR4252608_3443399___Sbg4
TGTCTATATGTTCGTGTTGTCTCGGAGGT
>SRR4252611_2781083___Sbg4
AGTCATGTAAATAGTACCTACTAGTGGGG
>SRR4252610_7984762___Sbg4
TGTGGGTGTGAATGGTGTCTTTGTCTATA
>SRR4252621_7536079___Sbg4
TGTCTTTGTCTATATGTTCGTGTTGTCTCGG
>SRR4252610_8507676___Sbg4
AGTCATGTAAATAGTACCTACTAGTGGG
>SRR4252610_5903789___Sbg4
CATCATCTGGCCATCATGATCATCTTCA
>SRR4252609_6373660___Sbg4
TTTGTCTATATGTTCGTGTTGTCTCGGAG
>SRR4252625_3464833___Sbg4
CTCCATGTCCTTGTGTAGTCAAAAAAGT
>SRR4252611_15538840___Sbg4
CTTTGTCTATATGTTCGTGTTGTCTCGGA
```

>SRR4252606_8969087___Sbg4
TCCATGTCCTTGTGTAGTCAAAAAAGTA
>SRR4252605_7971909___Sbg4
AGTGGGGGAGGTAAAACTTGGAAAATG
>SRR4252610_12065397___Sbg4
GTTGGTATGAGTGTGGGTGTGAATGGTG
>SRR4252618_10233876___Sbg4
TGTCTATATGTTCGTGTTGTCTCGGAGG
>SRR4252609_11807558___Sbg4
CCTTGTTGGTATGAGTGTGGGTGTGAAT
>SRR4252610_4257359___Sbg4
TCTTTGTCTATATGTTCGTGTTGTCTCGGA
>SRR4252611_6282076___Sbg4
AGTCATGTAAATAGTACCTACTAGTGGG
>SRR4252613_2295630___Sbg4
AGGGAGGCCCTCCATGTCCTTGTGTA
>SRR4252606_5381771___Sbg4
AGTCATGTAAATAGTACCTACTAGTGGG
>SRR4252612_5725886___Sbg4
TCTTTGTCTATATGTTCGTGTTGTCTCG
>SRR4252607_2384176___Sbg4
CATCATCTGGCCATCATGATCATCTTCA
>SRR4252608_3803291___Sbg4
TCCTTGTGTAGTCAAAAAAGTATGAATGT
>SRR4252610_9992613___Sbg4
AGTCATGTAAATAGTACCTACTAGTGGGG
>SRR4252611_4445135___Sbg4
AGTCATGTAAATAGTACCTACTAGTGGGG
>SRR4252610_6796849___Sbg4
TCATGTAAATAGTACCTACTAGTGGGGGA
>SRR4252610_6144283___Sbg4
AGTCATGTAAATAGTACCTACTAGTGGGG
>SRR4252606_4442957___Sbg4
AGGTCTTGGCTCTGTAAGGCCCGGGTGG
>SRR4252605_6665201___Sbg4
CCAGGGAGGCCCTCCATGTCCTTGTGTA
>SRR4252623_2013___Sbg4
TGAATGGTGTCTTTGTCTATATGTTCGTGT
>SRR4252611_2586476___Sbg4
TCCAGGGAGGCCCTCCATGTCCTTGTGTA
>SRR4252608_9199599___Sbg4
AGTCATGTAAATAGTACCTACTAGTGGG
>SRR4252615_10268346___Sbg4
TCTTGGCTCTGTAAGGCCCGGGTGGCGTG
>SRR4252610_11459861___Sbg4
TCTTTGTCTATATGTTCGTGTTGTCTCGGA
>SRR4252608_10921550___Sbg4
TGAATGGTGTCTTTGTCTATATGTTCGTG
>SRR4252607_13765813___Sbg4
TCTTTGTCTATATGTTCGTGTTGTCTCGGA
>SRR4252612_4331728___Sbg4
TCTTTGTCTATATGTTCGTGTTGTCTCGG
>SRR4252606_2397998___Sbg4
AGTCATGTAAATAGTACCTACTAGTGGGGGA
>SRR4252605_6975052___Sbg4
TGTGGGTGTGAATGGTGTCTTTGTCTA
>SRR4252608_8290722___Sbg4
AGTCATGTAAATAGTACCTACTAGTGGG
>SRR4252619_7680236___Sbg4
CTTTGTCTATATGTTCGTGTTGTCTCGGA
>SRR4252610_3384252___Sbg4
GTGTGGGTGTGAATGGTGTCTTTGTCTATA
>SRR4252624_10090393___Sbg4
TTTGTCTATATGTTCGTGTTGTCTCGGA
>SRR4252624_7461594___Sbg4
TCCTTGTGTAGTCAAAAAAGTATGAATGT
>SRR4252611_6076070___Sbg4
TCCAGGGAGGCCCTCCATGTCCTTGTGTA
>SRR4252624_3158595___Sbg4
TCCAGGGAGGCCCTCCATGTCCTTGTGTA

>SRR4252611_3326756___Sbg4
CATCATCTGGCCATCATGATCATCTTCA
>SRR4252621_8487580___Sbg4
ACAAATGTCAGGAAACTTTAGGCCCA
>SRR4252609_8102054___Sbg4
ACATCATCTGGCCATCATGATCATCTTCA
>SRR4252613_5004956___Sbg4
TCTTTGTCTATATGTTCGTGTTGTCTCGGA
>SRR4252609_7504203___Sbg4
TTGTCTCGGAGGTGGCCCTGGCATTGAGC
>SRR4252612_3302542___Sbg4
GCCCTCCATGTCCTTGTGTAGTCAAAAAA
>SRR4252607_10631175___Sbg4
TCCTTGTGTAGTCAAAAAAGTATGAAT
>SRR4252615_5162664___Sbg4
TCTTGGCTCTGTAAGGCCCGGGTGGCGTG
>SRR4252610_7588855___Sbg4
TCCTTGTGTAGTCAAAAAAGTATGAATGT
>SRR4252610_940713___Sbg4
ACCTACTAGTGGGGGAGGTAAAACTTGGA
>SRR4252612_3724662___Sbg4
CATCATCTGGCCATCATGATCATCTTCA
>SRR4252610_5575154___Sbg4
AGTCATGTAAATAGTACCTACTAGTGGGG
>SRR4252626_2985623___Sbg4
TTGTCTCGGAGGTGGCCCTGGCATTGAGC
>SRR4252605_6010710___Sbg4
TTTGTCTATATGTTCGTGTTGTCTCGG
>SRR4252608_6668581___Sbg4
AGTCATGTAAATAGTACCTACTAGTGGGG
>SRR4252618_5570372___Sbg4
GGGCCCAAACGGTCCCCAGACATCAT
>SRR4252608_6913390___Sbg4
CTTTGTCTATATGTTCGTGTTGTCTCGGA
>SRR4252611_12173460___Sbg4
TGGCTCTGTAAGGCCCGGGTGGCGTG
>SRR4252608_3179073___Sbg4
TTTGTCTATATGTTCGTGTTGTCTCGGAGGT
>SRR4252610_1162949___Sbg4
ATCCAGGGAGGCCCTCCATGTCCTTGTGTA
>SRR4252610_9392798___Sbg4
TCTTTGTCTATATGTTCGTGTTGTCTCGGA
>SRR4252612_7862289___Sbg4
TCTTTGTCTATATGTTCGTGTTGTCTCGGA
>SRR4252612_2424990___Sbg4
TCCATGTCCTTGTGTAGTCAAAAAAGTA
>SRR4252611_6488402___Sbg4
AAGACAAATGTCAGGAAACTTTAGGCCCA
>SRR4252609_13934578___Sbg4
CTTCATCATATTCGCCTTGTTGGTATGAGT
>SRR4252611_7786578___Sbg4
CCCGGGTGGCGTGAGTCATGTAAATAGTA
>SRR4252609_8055905___Sbg4
AGTCATGTAAATAGTACCTACTAGTGGG
>SRR4252610_5067571___Sbg4
AGTCATGTAAATAGTACCTACTAGTGG
>SRR4252611_15452996___Sbg4
TCTTTGTCTATATGTTCGTGTTGTCTCG
>SRR4252623_14679939___Sbg4
TCCTTGTGTAGTCAAAAAAGTATGAATG
>SRR4252611_9010107___Sbg4
ACATCATCTGGCCATCATGATCATCTTCA
>SRR4252610_4569694___Sbg4
CTTTGTCTATATGTTCGTGTTGTCTCGGA
>SRR4252611_11966472___Sbg4
CCATGTCCTTGTGTAGTCAAAAAAGTATG
>SRR4252609_6015920___Sbg4
TCCTTGTGTAGTCAAAAAAGTATGAATGTAT
>SRR4252619_1563680___Sbg4
AGTCATGTAAATAGTACCTACTAGTGGGG

>SRR4252606_1676994___Sbg4
TTTGTCTATATGTTCGTGTTGTCTCGG
>SRR4252611_173692___Sbg4
TCCTTGTGTAGTCAAAAAAGTATGTATGT
>SRR4252625_4699453___Sbg4
CCATAGTCTATCCTCTCCCGACAGGTC
>SRR4252612_6501553___Sbg4
GAGTCATGTAAATAGTACCTACTAGTGGG
>SRR4252607_10193407___Sbg4
TGTGTAGTCAAAAAAGTATGAATGTAAAT
>SRR4252610_5478769___Sbg4
AAGACAAATGTCAGGAAACTTTAGGCCCA
>SRR4252610_11361250___Sbg4
TTGTCTATATGTTCGTGTTGTCTCGGA
>SRR4252608_11819213___Sbg4
TGTCTATATGTTCGTGTTGTCTCGGAGGT
>SRR4252620_5241498___Sbg4
TGTCAGGAAATTTTAGGCCCAAACGGTCCCC
>SRR4252622_10680858___Sbg4
TGTCAGGAAATTTTAGGCCCAAACGGTCC
>SRR4252624_5005157___Sbg4
CATCATCTGGCCATCATGATCATCTTCA
>SRR4252609_15991538___Sbg4
CCATGTCCTTGTGTAGTCAAAAAAGTA
>SRR4252613_2019583___Sbg4
CCAGGGAGGCCCTCCATGTCCTTGTGTA
>SRR4252609_5550014___Sbg4
TGTCTATATGTTCGTGTTGTCTCGGAGG
>SRR4252614_3023489___Sbg4
TCCAGGGAGGCCCTCCATGTCCTTGTGTA
>SRR4252612_585574___Sbg4
CCCTCCATGTCCTTGTGTAGTCAAAAAAG
>SRR4252610_2513698___Sbg4
TGTCTATATGTTCGTGTTGTCTCGGA
>SRR4252616_3513936___Sbg4
AGGCCCGGGTGGCGTGAGTCATGTAAATA
>SRR4252623_3500551___Sbg4
TCTTTGTCTATATGTTCGTGTTGTCTCGGA
>SRR4252608_6308067___Sbg4
TTGTCTCGGAGGTGGCCCTGGCATTGAGC
>SRR4252608_8307507___Sbg4
AGTCATGTAAATAGTACCTACTAGTGGGG
>SRR4252610_8892400___Sbg4
AGTCATGTAAATAGTACCTACTAGTGGG
>SRR4252610_6688816___Sbg4
GGTGTCTTTGTCTATATGTTCGTGTTGTC
>SRR4252605_1471791___Sbg4
TCCTTGTGTAGTCAAAAAAGTATGAATG
>SRR4252609_7925681___Sbg4
TCCTTGTGTAGTCAAAAAAGTATGAATGT
>SRR4252605_13801545___Sbg4
TTGTCTCGGAGGTGGCCCTGGCATTGAGC
>SRR4252609_12030088___Sbg4
TGTCTCGGAGGTGGCCCTGGCATTGAGC
>SRR4252615_8252860___Sbg4
TCTATATGTTCGTGTTGTCTCGGAGGTG
>SRR4252622_2875943___Sbg4
CGGGTGGCGTGAGTCATGTAAATAGTA
>SRR4252621_12240901___Sbg4
TCTTTGTCTATATGTTCGTGTTGTCTCGGA
>SRR4252610_9475372___Sbg4
ACCTACTAGTGGGGGAGGTAAAACTTGGA
>SRR4252612_7831781___Sbg4
AGTCATGTAAATAGTACCTACTAGTGGG
>SRR4252609_12444555___Sbg4
ATCATGATCATCTTCATCATATTCGCC
>SRR4252608_7631251___Sbg4
TCCTTGTGTAGTCAAAAAAGTATGAAT
>SRR4252611_2066527___Sbg4
TCTATATGTTCGTGTTGTCTCGGAGGTG

>SRR4252605_10463697___Sbg4
ATGGTGTCTTTGTCTATATGTTCGTGTTG
>SRR4252611_7503186___Sbg4
TCCTTGTGTAGTCAAAAAAGTATGAATA
>SRR4252611_12451441___Sbg4
AGGCCCAAACGGTCCCCAGACATCAT
>SRR4252610_12966086___Sbg4
AGTCATGTAAATAGTACCTACTAGTGGGG
>SRR4252609_14421651___Sbg4
CCATGTCCTTGTGTAGTCAAAAAAGTATG
>SRR4252619_5290773___Sbg4
TCCTTGTGTAGTCAAAAAAGTATGAATG
>SRR4252611_12898033___Sbg4
TCCAGGGAGGCCCTCCATGTCCTTGTGTA
>SRR4252622_3233100___Sbg4
CCTTGTGTAGTCAAAAAAGTATGAATGTA
>SRR4252605_134981___Sbg4
CAGGTCTTGGCTCTGTAAGTCCCGGGT
>SRR4252610_5771369___Sbg4
CCAGGGAGGCCCTCCATGTCCTTGTGTA
>SRR4252610_586099___Sbg4
GAATGGTGTCTTTGTCTATATGTTCGTG
>SRR4252608_3843215___Sbg4
AGTCATGTAAATAGTACCTACTAGTGGGG
>SRR4252609_10128293___Sbg4
CCGGGTGGCGTGAGTCATGTAAATAGTA
>SRR4252612_2171168___Sbg4
CATCTGGCCATCATGATCATCTTCATCA
>SRR4252605_2078383___Sbg4
CCGGGTGGCGTGAGTCATGTAAATAGTA
>SRR4252610_948515___Sbg4
GATCATGTAAATAGTACCTACTAGTGGG
>SRR4252607_4264525___Sbg4
TTGTCTCGGAGGTGGCCCTGGCATTGAGC
>SRR4252614_6562675___Sbg4
AGGGAGGCCCTCCATGTCCTTGTGTA
>SRR4252611_3712096___Sbg4
TTTGTCTATATGTTCGTGTTGTCTCGGAG
>SRR4252609_13810536___Sbg4
TTGTCTATATGTTCGTGTTGTCTCGGA
>SRR4252608_9044514___Sbg4
CATCTTCATCATATTCGCCTTGTTGGTA
>SRR4252610_7172319___Sbg4
ACATCATCTGGCCATCATGATCATCTTCA
>SRR4252609_209177___Sbg4
ACATCATCTGGCCATCATGATCATCT
>SRR4252611_9387926___Sbg4
TAAGACAAATGTCAGGAAACTTTAGGCCCA
>SRR4252615_5665397___Sbg4
TCTTGGCTCTGTAAGGCCCGGGTGGCGTG
>SRR4252610_6894104___Sbg4
AGTCATGTAAATAGTACCTACTAGTGGGG
>SRR4252606_2644552___Sbg4
ATGGTGTCTTTGTCTATATGTTCGTGTTG
>SRR4252626_7594444___Sbg4
AGTCTATCCTCTCCCGACAGGTCTTGGCTC
>SRR4252606_7236377___Sbg4
CGTGTTGTCTCGGAGGTGGCCCTGGCATTGA
>SRR4252624_9013278___Sbg4
TTTAGGCCCAAACGGTCCCCAGACATCATCT
>SRR4252622_10187042___Sbg4
ACATCATCTGGCCATCATGATCATCTTCA
>SRR4252612_6287338___Sbg4
GGAGGCCCTCCATGTCCTTGTGTAGTCA
>SRR4252605_5146165___Sbg4
TTGTCTCGGAGGTGGCCCTGGCATTGAGC
>SRR4252611_13291331___Sbg4
CCAGGGAGGCCCTCCATGTCCTTGTGTA
>SRR4252605_4395519___Sbg4
TAAGACAAATGTCAGGAAACTTTAGGCCCA

>SRR4252612_5772095___Sbg4
AGTCATGTAAATAGTACCTACTAGTGGG
>SRR4252611_11517443___Sbg4
CCATGTCCTTGTGTAGTCAAAAAAGTA
>SRR4252611_6730321___Sbg4
TCCAGGGAGGCCCTCCATGTCCTTGTGTA
>SRR4252606_5796157___Sbg4
ATTGTCTATATGTTCGTGTTGTCTCGGA
>SRR4252608_10604552___Sbg4
ATTGTCTATATGTTCGTGTTGTCTCGGA
>SRR4252623_9757263___Sbg4
TCCAGGGAGGCCCTCCATGTCCTTGTGTA
>SRR4252611_595229___Sbg4
TGTGAGTCATGTAAATAGTACCTACTAGT
>SRR4252606_9810991___Sbg4
AGGGAGGCCCTCCATGTCCTTGTGTA
>SRR4252612_6622155___Sbg4
AGTCATGTAAATAGTACCTACTAGTGGGG
>SRR4252610_146793___Sbg4
TTAGGCCCAAACGGTCCCCAGACATCA
>SRR4252605_13335726___Sbg4
CTTTGTCTATATGTTCGTGTTGTCTCGGA
>SRR4252619_866170___Sbg4
TCCTTGTGTAGTCAAAAAAGTATGAATGT
>SRR4252605_7752273___Sbg4
ACATCATCTGGCCATCATGATCATCTTCA
>SRR4252611_11995396___Sbg4
TTGTCTCGGAGGTGGCCCTGGCATTGAGC
>SRR4252605_9782436___Sbg4
TCCAGGGAGGCCCTCCATGTCCTTGTGTA
>SRR4252610_2041852___Sbg4
TCCAGGGAGGCCCTCCATGTCCTTGTGTA
>SRR4252605_5535966___Sbg4
TTGTCTCGGAGGTGGCCCTGGCATTGAGC
>SRR4252608_12308890___Sbg4
TCCTTGTGTAGTCAAAAAAGTATGAATGT
>SRR4252608_5838948___Sbg4
AGTCATGTAAATAGTACCTACTAGTGGGG
>SRR4252611_6302211___Sbg4
TTGTCTATATGTTCGTGTTGTCTCGGAG
>SRR4252612_6172305___Sbg4
TCTGGCCATCATGATCATCTTCATCA
>SRR4252607_13424226___Sbg4
TTGTCTCGGAGGTGGCCCTGGCATTGAGC
>SRR4252608_11198170___Sbg4
TCCTTGTGTAGTCAAAAAAGTATGAATG
>SRR4252619_1395321___Sbg4
TCCTTGTGTAGTCAAAAAAGTATGAAT
>SRR4252607_4720948___Sbg4
AGTCATGTAAATAGTACCTACTAGTGGGG
>SRR4252611_14410953___Sbg4
GGAGGTAAAACTTGGAAAATGGAATGGAA
>SRR4252611_6226259___Sbg4
ACAAATGTCAGGAAACTTTAGGCCCA
>SRR4252610_5478165___Sbg4
ATCTTCATCATATTCGCCTTGTTGGT
>SRR4252610_2889009___Sbg4
AGTCATGTAAATAGTACCTACTAGTGGGG
>SRR4252610_1751131___Sbg4
TCTTTGTCTATATGTTCGTGTTGTCTCGG
>SRR4252612_2474813___Sbg4
CCTACTAGTGGGGGAGGTAAAACTTGGA
>SRR4252605_2378036___Sbg4
CCTTGTCTATATGTTCGTGTTGTCTCGGA
>SRR4252612_9137279___Sbg4
TCCAGGGAGGCCCTCCATGTCCTTGTGTA
>SRR4252622_5568336___Sbg4
TAAGACAAATGTCAGGAAACTTTAGGCCCA
>SRR4252610_4219455___Sbg4
TCTATATGTTCGTGTTGTCTCGGAGGT

>SRR4252610_10897835___Sbg4
AGTCATGTAAATAGTACCTACTAGTGGGG
>SRR4252611_8401144___Sbg4
AGTCATGTAAATAGTACCTACTAGTGGG
>SRR4252611_7719698___Sbg4
GGGAGGCCCTCCATGTCCTTGTGTAGTCA
>SRR4252611_2329295___Sbg4
TCCTTGTGTAGTCAAAAAAGTATGAATGT
>SRR4252612_2903646___Sbg4
CCAGGGAGGCCCTCCATGTCCTTGTGTA
>SRR4252605_2902610___Sbg4
TCCTTGTGTAGTCAAAAAAGTATGAATG
>SRR4252610_2610669___Sbg4
AGTCATGTAAATAGTACCTACTAGTGGG
>SRR4252616_4810967___Sbg4
AAGACAAATGTCAGGAAACTTTAGGCCAA
>SRR4252607_11426070___Sbg4
TGTTGTCTCGGAGGTGGCCCTGGCATTAAGC
>SRR4252614_5854979___Sbg4
TCCTTGTGTAGTCAAAAAAGTATGAAT
>SRR4252608_9887082___Sbg4
ATGTCAGGAAACTTTAGGCCCAAACGG
>SRR4252611_14482378___Sbg4
ATCATCTTCATCATATTCGCCTTGTTGG
>SRR4252610_6163570___Sbg4
CCTTGTCTAGTCAAAAAAGTATGAATGTA
>SRR4252607_4043713___Sbg4
TTGTTGGTATGAGTGTGGGTGTGAATGGTG
>SRR4252607_5288081___Sbg4
TTGTCTCGGAGGTGGCCCTGGCATTGAGC
>SRR4252607_11539752___Sbg4
ACTAGTGGGGGAGGTAAAACTTGGAAA
>SRR4252616_15004911___Sbg4
AATCATCTTCATCATATTCGCCTTGT
>SRR4252608_135920___Sbg4
GTTGGTATGAGTGTGGGTGTGAATGGTGTC
>SRR4252611_9440005___Sbg4
CCAGGGAGGCCCTCCATGTCCTTGTGTA
>SRR4252605_12482987___Sbg4
GGCCCGGGTGGCGTGAGTCATGTAAATAGTA
>SRR4252605_5586590___Sbg4
TCGTGTTGTCTCGGAGGTGGCCCTGGCA
>SRR4252622_4016199___Sbg4
GCATCATCTGGCCATCATGATCATCTTCA
>SRR4252608_8619202___Sbg4
TCCTTGTGTAGTCAAAAAAGTATGAATGT
>SRR4252619_5410808___Sbg4
GGCATTGAGCTGATCACTTATCCAGGGA
>SRR4252621_4799180___Sbg4
TATGACAAATGTCAGGAAACTTTAGGCCCA
>SRR4252610_13116925___Sbg4
TCCTTGTGTAGTCAAAAAAGTATGAATG
>SRR4252612_1086046___Sbg4
TTGTCTGTATGTTCGTGTTGTCTCGGAG
>SRR4252611_7858374___Sbg4
ATCATCTTCATCATATTCGCCTTGTTGG
>SRR4252624_728680___Sbg4
CAGGGAGGCCCTCCATGTCCTTGTGTAGT
>SRR4252613_4342073___Sbg4
TTGTCTATATGTTCGTGTTGTCTCGGAG
>SRR4252612_8444147___Sbg4
TGTGATCCATAGTCTATCCTCTCCCG
>SRR4252612_2257730___Sbg4
TTTGTCTATATGTTCGTGTTGTCTCGGA
>SRR4252610_837093___Sbg4
AGGGAGGCCCTCCATGTCCTTGTGTA
>SRR4252616_3407077___Sbg4
TGTCTATATGTTCGTGTTGTCTCGGAGGT
>SRR4252614_9388796___Sbg4
TGTTGTCTCGGAGGTGGCCCTGGCATT

>SRR4252611_2573412___Sbg4
TCCTTGTGTAGTCAAAAAAGTATGAAG
>SRR4252616_2797817___Sbg4
TGTCTCGGAGGTGGCCCTGGCATTGA
>SRR4252612_5164618___Sbg4
CATAGTCTATCCTCTCCCGACAGGTCTT
>SRR4252611_9446898___Sbg4
TCCTTGTGTAGTCAAAAAAGTATGAATGT
>SRR4252611_8971654___Sbg4
CCCAGACATCATCTGGCCATCATGATCA
>SRR4252609_7832089___Sbg4
CATCTGGCCATCATGATCATCTTCATCA
>SRR4252623_13245074___Sbg4
TTGTCTCGGAGGTGGCCCTGGCATTGAGC
>SRR4252610_9082267___Sbg4
AGTCATGTAAATAGTACCTACTAGTGGGG
>SRR4252610_3140481___Sbg4
AGTCATGTAAATAGTACCTACTAGTGGGG
>SRR4252611_12133252___Sbg4
TAAGACAAATGTCAGGAAACTTTAGGCCCA
>SRR4252611_9566873___Sbg4
TCTTTGTCTATATGTTCGTGTTGTCTCGG
>SRR4252609_10138399___Sbg4
AAACTTTAGGCCCAAACGGTCCCCAGACA
>SRR4252625_3418275___Sbg4
CTCCATGTCCTTGTGTAGTCAAAAAAGT
>SRR4252605_7598917___Sbg4
ACATCATCTGGCCATCATGATCATCTTCA
>SRR4252610_1744823___Sbg4
TCTCCCGACAGGTCTTGGCTCTGTAAG
>SRR4252610_9846929___Sbg4
TTTGTCTATATGTTCGTGTTGTCTCGGA
>SRR4252611_1225830___Sbg4
AGTCATGTAAATAGTACCTACTAGTGGG
>SRR4252612_6409455___Sbg4
CATAAGACAAATGTCAGGAAACTTTAGGC
>SRR4252605_12206512___Sbg4
CATCATCTGGCCATCATGATCATCTTCA
>SRR4252611_774799___Sbg4
AGGGAGCCCCTCCATGTCCTTGTGTAGTCA
>SRR4252624_8737694___Sbg4
AGTGGGGGAGGTAAAACTTGGAAAAT
>SRR4252609_16566638___Sbg4
TCCTTGTGTAGTCAAAAAAGTATGAATG
>SRR4252611_4764603___Sbg4
ATGTCAGGAAACTTTAGGCCCAAACGGTC
>SRR4252607_4440047___Sbg4
TCCTTGTGTAGTCAAAAAAGTATGAATGA
>SRR4252611_13046512___Sbg4
TGTGTAGTCAAAAAAGTATGAATGAATATG
>SRR4252612_1560650___Sbg4
ATGTCAGGAAACTTTAGGCCCAAACGGT
>SRR4252605_14976695___Sbg4
TTGTCTCGGAGGTGGCCCTGGCATTGAGC
>SRR4252611_9512226___Sbg4
TCCTTGTGTAGTCAAAAAAGTATGAATGT
>SRR4252605_8212774___Sbg4
AGTCATGTAAATAGTACCTACTAGTGGG
>SRR4252611_14206844___Sbg4
TTTGTCTATATGTTCGTGTTGTCTCGGAG
>SRR4252614_9387649___Sbg4
ATCATGATCATCTTCATCATATTCGCCT
>SRR4252607_5213519___Sbg4
TACCTACTAGTGGGGGAGGTAAAACGTG
>SRR4252605_12272383___Sbg4
TTGTCTCGGAGGTGGCCCTGGCATTGAGC
>SRR4252606_8874651___Sbg4
TTTGTCTATATGTTCGTGATGTCTCGG
>SRR4252609_3577126___Sbg4
TGTCAGGAAACTTTAGGCCCAAACGGTCCC

>SRR4252613_901431___Sbg4
TCCAGGGAGGCCCTCCATGTCCTTGTGTA
>SRR4252616_13840513___Sbg4
GATCCATAGTCTATCCTCTCCCGACAGG
>SRR4252606_10289237___Sbg4
TTTGTCTATATGTTCGTGTTGTCTCGGA
>SRR4252609_1893587___Sbg4
TCCAGGGAGGCCCTCCATGTCCTTGTGTA
>SRR4252612_9516___Sbg4
TGTGTAGTCAAAAAAGTATGAATGTATATG
>SRR4252605_14289084___Sbg4
TCTTTGTCTATATGTTCGTGTTGTCTCGGA
>SRR4252607_9823380___Sbg4
TGTCTCGGAGGTGGCCCTGGCATTGAGC
>SRR4252619_7305854___Sbg4
CTTTAGGCCCAAACGGTCCCCAGACATCA
>SRR4252620_9806436___Sbg4
AATAGTACCTACTAGTGGGGGAGGTAAA
>SRR4252607_1744098___Sbg4
TGTCTCGGAGGTGGCCCTGGCATTGAGC
>SRR4252611_12953118___Sbg4
TCCAGGGAGGCCCTCCATGTCCTTGTGTA
>SRR4252623_5892623___Sbg4
CCAGGGAGGCCCTCCATGTCCTTGTGTA
>SRR4252605_390627___Sbg4
CATAAGACAAATGTCAGGAAACTTTAGGCC
>SRR4252609_4286883___Sbg4
GTTGTCTCGGAGGTGGCCCTGGCATTGAGC
>SRR4252605_9577523___Sbg4
CCATAAGACAAATGTCAGGAAACTTTAGG
>SRR4252606_9201115___Sbg4
TTGTCTATATGTTCGTGTTGTCTCGGAA
>SRR4252610_5317498___Sbg4
AGTCATGTAAATAGTACCTACTAGTGGG
>SRR4252605_11803690___Sbg4
TTGTCTCGGAGGTGGCCCTGGCATTGAGC
>SRR4252612_7678101___Sbg4
GGGTGGCGTGAGTCATGTAAATAGTACCTA
>SRR4252621_11309174___Sbg4
AATGTCAGGAAACTTTAGGCCCAAAC
>SRR4252610_12575979___Sbg4
CTTTGTCTATATGTTCGTGTTGTCTCGGA
>SRR4252605_3406932___Sbg4
AGTCATGTAAATAGTACCTACTAGTGGG
>SRR4252605_6512306___Sbg4
GTCATGTAAATAGTACCTACTAGTGGGG
>SRR4252607_10445329___Sbg4
TGTCAGGAAACTTTAGGCCCAAACGGT
>SRR4252606_10122608___Sbg4
CCGTGTGGCGTGAGTCATGTAAATAGTA
>SRR4252609_4412196___Sbg4
ACATCATCTGGCCATCATGATCATCT
>SRR4252607_4317082___Sbg4
AGTCATGTAAATAGTACCTACTAGTGGG
>SRR4252621_4077961___Sbg4
CCCTCCATGTCCTTGTGTAGTCAAAAA
>SRR4252611_6400250___Sbg4
CCATGTCCTTGTGTAGTCAAAAAAGTA
>SRR4252623_4807765___Sbg4
AGAGTCATGTAAATAGTACCTACTAGTGG
>SRR4252613_1938173___Sbg4
CTTGGCTCTGTAAGGCCCGGGTGGCGT
>SRR4252611_7164664___Sbg4
CTGGGGTTTTTCCTCACCCATAAGACA
>SRR4252611_4656357___Sbg4
AGTCATGTAAATAGTACCTACTAGTGGGG
>SRR4252606_3049480___Sbg4
TGTGGGTGTGAATGGTGTCTTTGTCTATA
>SRR4252609_10161037___Sbg4
GTGGGTGTGAATGGTGTCTTTGTCTATA

>SRR4252614_3318320___Sbg4
GCCCTCCATGTCCTTGTGTAGTCAAAAAA
>SRR4252611_8153689___Sbg4
TCCTTGTGTAGTCAAAAAAGTATGAATGT
>SRR4252608_7654813___Sbg4
AGTCATGTAAATAGTACCTACTAGTGGGG
>SRR4252610_7079285___Sbg4
CTACTAGTGGGGGAGGTAAAACTTGGAAA
>SRR4252606_5932618___Sbg4
TTGTTGGTATGAGTGTGGGAGTGAATGGTG
>SRR4252606_2455438___Sbg4
TGTTGTCTCGGAGGTGGCCCTGGCATTGAGC
>SRR4252611_8286219___Sbg4
CCAGGGAGGCCCTCCATGTCCTTGTGTA
>SRR4252610_5676200___Sbg4
CCAGGGAGGCCCTCCATGTCCTTGTGTA
>SRR4252615_12376013___Sbg4
TACTAGTGGGGGAGGTAAAACTTGGA
>SRR4252612_5285260___Sbg4
CTTTGTCTATATGTTCGTGTTGTCTCGGA
>SRR4252605_4033687___Sbg4
GTTGTCTCGGAGGTGGCCCTGGCATTGAGC
>SRR4252605_1034871___Sbg4
TCCTTGTGTAGTCAAAAAAGTATGAATG
>SRR4252610_5595000___Sbg4
TCCAGGGAGGCCCTCCATGTCCTTGTGTA
>SRR4252608_4178405___Sbg4
AGTCATGTAAATAGTACCTACTAGTGGGG
>SRR4252609_9555390___Sbg4
CCATGTCCTTGTGTAGTCAAAAAAGTA
>SRR4252626_5311927___Sbg4
TTTTCCTCACCCATAAGACAAATGTCA
>SRR4252611_15524800___Sbg4
TCCTTGTGTAGTCAAAAAAGTATGAATGTAT
>SRR4252606_2601574___Sbg4
TCTTTGTCTATATGTTCGTGTTGTCT
>SRR4252625_2494808___Sbg4
TTGTCTCGGAGGTGGCCCTGGCATTGAGC
>SRR4252611_7411098___Sbg4
TCTTGGCTCTGTAAGGCCCGGGTGGCGTGA
>SRR4252612_5435677___Sbg4
GTGTGGGTGTGAATGGTGTCTTTGTCTATA
>SRR4252605_8410928___Sbg4
GGTGGCCCTGGCATTGAGCTGATCAC
>SRR4252608_13047108___Sbg4
CATCTGGCCATCATGATCATCTTCATCA
>SRR4252612_2651341___Sbg4
AGTCATGTAAATAGTACCTACTAGTGGGG
>SRR4252606_6781782___Sbg4
CATCTTCATCATATTCGCCTTGTTGGTA
>SRR4252605_4051097___Sbg4
TCTGGGGTTTTTCCTCACCCATAAGACA
>SRR4252610_3564128___Sbg4
TTTGTCTATATGTTCGTGTTGTCTCGGA
>SRR4252614_9267416___Sbg4
CTTTGTCTATATGTTCGTGTTGTCTCGGA
>SRR4252609_6340429___Sbg4
TTGTCTCGGAGGTGGCCCTGGCATTGAGC
>SRR4252610_12947265___Sbg4
TCCAGGGAGGCCCTCCATGTCCTTGTGTA
>SRR4252607_365285___Sbg4
ACATCATCTGGCCATCATGATCATCTTCA
>SRR4252609_10733497___Sbg4
GTCATGTAAATAGTACCTACTAGTGGGGGA
>SRR4252605_9884379___Sbg4
CCTTGTCTATATGTTCGTGTTGTCTCGGA
>SRR4252614_9308931___Sbg4
CCTTGTCTATATGTTCGTGTTGTCTC
>SRR4252621_2473350___Sbg4
CCAGGGAGGCCCTCCATGTCCTTGTGTA

>SRR4252611_7284072___Sbg4
TCTTGGCTCTGTAAGGCCCGGGTGGCGTG
>SRR4252614_1261050___Sbg4
TGTCTATATGTTCGTGTTGTCTCGGAGGC
>SRR4252611_4470135___Sbg4
TCTTGGCTCTGTAAGGCCCGGGTGGCGTG
>SRR4252610_7172347___Sbg4
TCCTTGTGTAGTCAAAAAAGTATGAATGT
>SRR4252610_8786646___Sbg4
TCCAGGGAGGCCCTCCATGTCCTTGTGTA
>SRR4252611_8804654___Sbg4
AGGGAGGCCCTCCATGTCCTTGTGTA
>SRR4252620_9523844___Sbg4
CCATAGTATATCCTCTCCCGACAGGTC
>SRR4252607_2804625___Sbg4
TGTCTCGGAGGTGGCCCTGGCATTGAGC
>SRR4252605_14900595___Sbg4
AGTCATGTAAATAGTACCTACTAGTGGGG
>SRR4252611_9886949___Sbg4
GTGTGGGTGTGAATGGTGTCTTTGTCTATA
>SRR4252606_7827886___Sbg4
TTGTCTCGGAGGTGGCCCTGGCATTGAGC
>SRR4252611_5045920___Sbg4
CGAGTGGCGTGAGTCATGTAAATAGTA
>SRR4252624_10593601___Sbg4
GTTTTCCTCACCCATAAGACAAATGTCA
>SRR4252611_2807772___Sbg4
TGGGTGTGAATGGTGTCTTTGTCTATAT
>SRR4252610_9200055___Sbg4
ATCCAGGGAGGCCCTCCATGTCCTTGTGTA
>SRR4252610_839895___Sbg4
AGGGAGGCCCTCCATGTCCTTGTGTA
>SRR4252605_1422466___Sbg4
ACCTACTAGTGGGGGAGGTAAAACTTGGA
>SRR4252610_5243094___Sbg4
CCAGGGAGGCCCTCCATGTCCTTGTGTA
>SRR4252610_11848181___Sbg4
TCGGGTGGCGTGAGTCATGTAAATAGTA
>SRR4252619_12040236___Sbg4
TGGGTGTGAATGGTGTCTTTGTCTATA
>SRR4252610_9541754___Sbg4
TGTCTTTGTCTATATGTTCGTGTTGTCTC
>SRR4252605_5783628___Sbg4
TTGTCTCGGAGGTGGCCCTGGCATTGAGC
>SRR4252619_4242233___Sbg4
TCGGAGGTGGCCCTGGCATTGAGCTGAT
>SRR4252610_6177491___Sbg4
AATGAGTCATGTAAATAGTACCTACTAG
>SRR4252606_4822176___Sbg4
TGTCTATATGTTCGTGTTGTCTCGGAGGT
>SRR4252606_3451369___Sbg4
TATCCTCTCCCGACAGGTCTTGGCTCTGTAA
>SRR4252606_4110673___Sbg4
TCCTTGTGTAGTCAAAAAAGTATGAAT
>SRR4252608_5363863___Sbg4
TGATCACTTATCCAGGGAGGCCCTCTA
>SRR4252610_7006267___Sbg4
AGTCATGTAAATAGTACCTACTAGTGGGG
>SRR4252611_1212485___Sbg4
AATGTCAGGAAACTTTAGGCCCAAACGGT
>SRR4252611_13258579___Sbg4
CCATGTCCTTGTGTAGTCAAAAAAGTA
>SRR4252610_2083089___Sbg4
TCTATCCTCTCCCGACAGGTCTTGGCTCT
>SRR4252610_12711897___Sbg4
AGTCATGTAAATAGTACCTACTAGTGGG
>SRR4252609_14547872___Sbg4
TCCTTGTGTAGTCAAAAAAGTATGAATGT
>SRR4252608_10570218___Sbg4
TCCTTGTGTAGTCAAAAAAGTATGAATGT

>SRR4252612_2992885___Sbg4
CCAGGGAGGCCCTCCATGTCCTTGTGTA
>SRR4252613_3802950___Sbg4
AGGGAGGCCCTCCATGTCCTTGTGTA
>SRR4252613_7930713___Sbg4
TCTTGGCTCTGTAAGGCCCGGGTGGCGTG
>SRR4252626_5530345___Sbg4
ATGTCTATATGTTCGTGTTGTCTCGGA
>SRR4252608_11296364___Sbg4
GTTAGGCCCAAACGGTCCCCAGACATCA
>SRR4252611_6734433___Sbg4
TTTGTCTATATGTTCGTGTTGTCTCGGAG
>SRR4252609_16401677___Sbg4
TTGTCTATATGTTCGTGTTGTCTCGGA
>SRR4252624_2853094___Sbg4
AGGAGGCCCTCCATGTCCTTGTGTAGTCA
>SRR4252610_8865017___Sbg4
ATCATCTTCATCATATTCGCCTTGTTGT
>SRR4252610_3152936___Sbg4
GGTGTCTTTGTCTATATGTTCGTGTTGT
>SRR4252608_7388241___Sbg4
TGATGTAAATAGTACCTACTAGTGGG
>SRR4252612_2029393___Sbg4
TTCGTGTTGTCTCGGAGGTGGCCCTGG
>SRR4252607_14928233___Sbg4
TGAGTCATGTAAATAGTACCTACTAGT
>SRR4252611_15382336___Sbg4
ATGTCAGGAAACTTTAGGCCCAAACGGT
>SRR4252611_11824081___Sbg4
TTTGTCTATATGTTCGTGTTGTCTCGGA
>SRR4252607_4691786___Sbg4
TCCTGGGTGAGGTTTTTCTGGGGTTTTTCC
>SRR4252607_619423___Sbg4
CTTTGTCTATATGTTCGTGTTGTCTCGG
>SRR4252605_315941___Sbg4
TATGTGATCCATAGTCTATCCTCTCCCG
>SRR4252611_86582___Sbg4
AGTCATGTAAATAGTACCTACTAGTGGGG
>SRR4252608_7068579___Sbg4
CTGGGGTTTTTCCTCACCCATAAGACA
>SRR4252610_4001806___Sbg4
ACATCATCTGGCCATCATGATCATCTTCA
>SRR4252607_10629012___Sbg4
CTTGTTGGTATGAGTGTGGGTGTAAATGGTG
>SRR4252610_5628901___Sbg4
AGGGAGGCCCTCCATGTCCTTGTGTA
>SRR4252612_8695758___Sbg4
TCCAGGGAGGCCCTCCATGTCCTTGTGTA
>SRR4252610_2055204___Sbg4
TGTCTTTGTCTATATGTTCGTGTTGTC
>SRR4252611_247988___Sbg4
TCTATATGTTCGTGTTGTCTCGGAGGTG
>SRR4252607_9119627___Sbg4
TTTGTCTATATGTTCGTGTTGTCTCGGAG
>SRR4252622_3839710___Sbg4
TGTGTAGTCAAAAAAGTATGAATGTATAC
>SRR4252610_12623577___Sbg4
CTTTGTCTATATGTTCGTGTTGTCTCGGA
>SRR4252610_6307012___Sbg4
AGTCATGTAAATAGTACCTACTAGTGGG
>SRR4252605_3762047___Sbg4
CCATGTCCTTGTGTAGTCAAAAAAGTA
>SRR4252608_9812213___Sbg4
ACATCATCTGGCCATCATGATCATCTTCA
>SRR4252609_16342558___Sbg4
TTGTCTATATGTTCGTGTTGTCTCGGA
>SRR4252611_14311065___Sbg4
CCAGGGAGGCCCTCCATGTCCTTGTGTA
>SRR4252608_5017669___Sbg4
GGTCATGTAAATAGTACCTACTAGTGGGG

>SRR4252608_5601860___Sbg4
GGTCATGTAAATAGTACCTACTAGTGGG
>SRR4252610_10014899___Sbg4
GGTCATGTAAATAGTACCTACTAGTGGG
>SRR4252610_10983732___Sbg4
GGTCATGTAAATAGTACCTACTAGTGGGG
>SRR4252610_2562536___Sbg4
GGTCATGTAAATAGTACCTACTAGTGGG
>SRR4252610_973609___Sbg4
GGTCATGTAAATAGTACCTACTAGTGGG
>SRR4252609_5322599___Sbg4
GTCATGTAAATAGTACCTACTAGTGGG
>SRR4252609_5156063___Sbg4
TCTGGCCATCATAATCATCTTCATCA
>SRR4252615_14637059___Sbg4
CCCGACAGGTCTTGGCTCTGTAAGGC
>SRR4252624_1308343___Sbg4
AAATGTCAGGAAACTTTAGGCCCAAACGG
>SRR4252619_772604___Sbg4
TCCTTGTGTAGTCAAAAAAGTATGAATGT
>SRR4252610_5047228___Sbg4
CATCATCTGGCCATCATGATCATCTTCA
>SRR4252624_5623907___Sbg4
ATCTGGCCATCATGATCATCTTCATCATA
>SRR4252606_9681031___Sbg4
TTGTCTCGGAGGTGGCCCTGGCATTGAGT
>SRR4252607_8741098___Sbg4
TTGTCTCGGAGGTGGCCCTGGCATTGAGT
>SRR4252610_11834071___Sbg4
TTGTCTCGGAGGTGGCCCTGGCATTGAGT
>SRR4252618_10414631___Sbg4
TTGTCTCGGAGGTGGCCCTGGCATTGAGT
>SRR4252610_7121248___Sbg4
TCATGTAAATAGTACCTACTAGTGGGGGA
>SRR4252606_3003077___Sbg4
CCTTGTCTATATGTTCGTGTTGTCTCGGA
>SRR4252615_4458511___Sbg4
AGTCTATCCTCTCCCGACAGGTCTTGG
>SRR4252611_4769095___Sbg4
TCTATATGTTCGTGTTGTCTCGGGGGT
>SRR4252607_10078513___Sbg4
ATGAATGTATATGTGATCCATAGTCTAT
>SRR4252605_5101456___Sbg4
AGTCATGTAAATAGTACCTACTAGTGGGG
>SRR4252607_5744082___Sbg4
TGTCAGGAAACTTTAGGCCCAAACGG
>SRR4252607_11504079___Sbg4
GTCTATATGTTCGTGTTGTCTCGGAGGT
>SRR4252610_6764968___Sbg4
CATCATCTGGCCATCATGATCATCTTCA
>SRR4252612_6208243___Sbg4
AATGTCAGGAAACTTTAGGCCCAAACGGT
>SRR4252623_6817565___Sbg4
AGCCCTCCATGTCCTTGTGTAGTCAAAAA
>SRR4252616_7311600___Sbg4
CTACTAGTGGGGGAGGTAAAACTTGGAA
>SRR4252609_4947082___Sbg4
TGTCAGGAAACTTTAGGCCCAAACGGT
>SRR4252610_3403769___Sbg4
CCCCAGACATCATCTGGCCATCATGATCA
>SRR4252611_15174150___Sbg4
ACCAGGGAGGCCCTCCATGTCCTTGTGTA
>SRR4252614_5987350___Sbg4
ACCAGGGAGGCCCTCCATGTCCTTGTGTA
>SRR4252625_676056___Sbg4
ACCAGGGAGGCCCTCCATGTCCTTGTGTA
>SRR4252609_15983531___Sbg4
TCCTTGTGTAGTCAAAAAAGTATGAATG
>SRR4252619_8785600___Sbg4
CATAGTCTATCCTCTCCCGACAGGTCTTGG

>SRR4252611_14594118___Sbg4
CATCTGGCCATCATGATCATCTTCATCA
>SRR4252605_14839287___Sbg4
TCATCTGGCCATCATGATCATCTTCATC
>SRR4252606_766472___Sbg4
GTTGGTATGAGTGTGGGTGTGAATGGTGTC
>SRR4252605_10456239___Sbg4
ATAGTCTATCCTCTCCCGACAGGTCTTGG
>SRR4252605_6413353___Sbg4
TTGTCTCGGAGGTGGCCCTGGCATTGAGC
>SRR4252609_9178707___Sbg4
TTGTCTCGGAGGTGGCCCTGGCATTGAGC
>SRR4252606_10370827___Sbg4
AGTCATGTAAATAGTACCTACTAGTGGGG
>SRR4252610_4274838___Sbg4
AGTCATGTAAATAGTACCTACTAGTGGG
>SRR4252621_13587696___Sbg4
AGTCATGTAAATAGTACCTACTAGTGGG
>SRR4252609_14940550___Sbg4
CATCATCTGGCCATCATGATCATCTTCA
>SRR4252607_5929___Sbg4
ACATCATCTGGCCATCATGATCATCTTCA
>SRR4252610_5603272___Sbg4
ACATCATCTGGCCATCATGATCATCTTCA
>SRR4252611_4341291___Sbg4
TCAGGGAGGCCCTCCATGTCCTTGTGTA
>SRR4252605_13938338___Sbg4
GTACCTACTAGTGGGGGAGGTAAAACTTGG
>SRR4252623_12265949___Sbg4
CATCATCTGGCCATCATGATCATCTTCA
>SRR4252608_5158337___Sbg4
TTGTCTATATGTTCGTGTTGTCTCGGA
>SRR4252610_8527355___Sbg4
TCCAGGGAGGCCCTCCATGTCCTTGTGTA
>SRR4252611_7026473___Sbg4
TCCAGGGAGGCCCTCCATGTCCTTGTGTA
>SRR4252607_8594105___Sbg4
TCCTTGTGTAGTCAAAAAAGTTTGAATGT
>SRR4252609_8420099___Sbg4
TCATCTGGCCATCATGATCATCTTCATCA
>SRR4252606_9006059___Sbg4
TATGAGTGTGGGTGTGAATGGTGTCTT
>SRR4252605_14163110___Sbg4
CTTTGTCTATATGTTCGTGTTGTCTCGGA
>SRR4252615_441405___Sbg4
TAAGACAAATGTCAGGAAACTTTAGGCCC
>SRR4252605_3336061___Sbg4
CATCTGGCCATCATAATCATCTTCATCA
>SRR4252607_1520456___Sbg4
TCCTTGTGTAGTCAAAAAAGTATGAATGT
>SRR4252610_3213330___Sbg4
AGTCATGTAAATAGTACCTACTAGTGGG
>SRR4252610_3682323___Sbg4
CATCTGGCCATCATAATCATCTTCATCA
>SRR4252609_42454___Sbg4
TCTGGGGTTTTTCCTCACCCATAAGACA
>SRR4252613_6308151___Sbg4
GAGGCTCTCCATGTCCTTGTGTAGTCAAA
>SRR4252611_1477654___Sbg4
GAAGGCCCTCCATGTCCTTGTGTAGTCAAA
>SRR4252606_3560920___Sbg4
AGTCATGTAAATAGTACCTACTAGTGGG
>SRR4252613_8499915___Sbg4
TCCAGGGAGGCCCTCCATGTCCTTGTGTA
>SRR4252612_941639___Sbg4
TCTTGGCTCTGTAAGGCCCGGGTGGCGTG
>SRR4252606_707366___Sbg4
TCGGGTGGCGTGAGTCATGTAAATAGTA
>SRR4252610_12979599___Sbg4
AGTCATGTAAATAGTACCTACTAGTGGGG

```
>SRR4252610_5431374___Sbg4
AGTCATGTAAATAGTACCTACTAGTGGG
>SRR4252605_8845187___Sbg4
AGTCATGTAAATAGTACCTACTAGTGGG
>SRR4252610_9949236___Sbg4
TAGTCTATCCTCTCCCGACAGGTCTTGG
>SRR4252610_2878296___Sbg4
GAGTCATGTAAATAGTACCTACTAGTGGG
>SRR4252610_6543927___Sbg4
TGTAGTCAAAAAAGTATGAATGTATATGT
>SRR4252615_2622094___Sbg4
TCTTGGCTCTGTAAGGCCCGGGTGGCGTG
>SRR4252608_12594136___Sbg4
ATGGTGTCTTTGTCTATATGTTCGTG
>SRR4252609_3037845___Sbg4
ACAAACGTCAGGAAACTTTAGGCCCA
>SRR4252607_9624536___Sbg4
TTGTCTATATGTTCGTGTTGTCTCGGA
>SRR4252610_7223372___Sbg4
TCATCTTCATCATATTCGCCTTGTTGGTA
>SRR4252610_7820292___Sbg4
GGGTGGCGTGAGTCATGTAAATAGTACCTA
>SRR4252605_9424109___Sbg4
TCCAGGGAGGCCCTCCATGTCCTTGTGTA
>SRR4252605_14039027___Sbg4
TCCTTGTGTAGTCAAAAAAGTATGAATGT
>SRR4252616_9117964___Sbg4
GTTGTCTCGGAGGTGGCCCTGGCATTGAG
>SRR4252607_6345357___Sbg4
CCTTGTCTAGTCAAAAAAGTATGAATGTA
>SRR4252608_925846___Sbg4
TCGTGTTGTCTCGGAGGTGGCCCTGGCA
>SRR4252615_12463745___Sbg4
TCCATGTCCTTGTGTAGTCAAAAAAG
>SRR4252613_8955655___Sbg4
TCTCCCGACAGGTCTTGGCTCTGTAAGGC
>SRR4252619_12693617___Sbg4
CATCTTCATCATATTCGCCTTGTTGGTA
>SRR4252611_7033479___Sbg4
AGTCCTTGTGTAGTCAAAAAAGTATGA
>SRR4252610_7387269___Sbg4
TCCTTGTGTAGTCAAAAAAGTATGAAT
>SRR4252605_7915931___Sbg4
AGTCATGTAAATAGTACCTACTAGTGGGG
>SRR4252611_7406427___Sbg4
ACATCATCTGGCCATCATGATCATCTTCA
>SRR4252611_12337233___Sbg4
TTTGTCTATATGTTCGTGTTGTCTCGGAG
>SRR4252610_11112256___Sbg4
GGGGAGGCCCTCCATGTCCTTGTGTA
>SRR4252610_1792583___Sbg4
GGGGAGGCCCTCCATGTCCTTGTGTA
>SRR4252605_11002933___Sbg4
AGGGAGGCCCTCCATGTCCTTGTGTA
>SRR4252610_11401370___Sbg4
ATGTCAGGAAACTTTAGGCCCAAACGGT
>SRR4252616_467462___Sbg4
TCCTTGTGTAGTCAAAAAAGTATGAAT
>SRR4252608_146624___Sbg4
CATCATCTGGCCATCATGATCATCTTCA
>SRR4252613_10254351___Sbg4
GGCCATCATAATCATCTTCATCATATTC
>SRR4252607_4669409___Sbg4
AGTCATGTAAATAGTACCTACTAGTGGGG
>SRR4252612_9195870___Sbg4
AATGTCAGGAAACTTTAGGCCCAAACGT
>SRR4252611_8704283___Sbg4
TCCAGGGAGGCCCTCCATGTCCTTGTGTA
>SRR4252609_10825996___Sbg4
CCATGTCCTTGTGTAGTCAAAAAAGTA
```

>SRR4252611_2685497___Sbg4
TCTTTGTCTATATGTTCGTGTTGTCTCG
>SRR4252611_14925759___Sbg4
CATCTGGCCATCATGATCATCTTCAT
>SRR4252607_6196694___Sbg4
ATGAATGTATATGTGATCCATAGTCTA
>SRR4252612_7344050___Sbg4
AGTCATGTAAATAGTACCTACTAGTGG
>SRR4252606_561370___Sbg4
CCAGGGAGGCCCTCCATGTCCTTGTGTA
>SRR4252610_12331608___Sbg4
TGGGTGTGAATGGTGTCTTTGTCTATA
>SRR4252606_1386325___Sbg4
TGTAGTCAAAAAAGTATGAATGTATA
>SRR4252610_4019681___Sbg4
TCCAGGGAGGCCCTCCATGTCCTTGTGTA
>SRR4252610_9827622___Sbg4
TGTCTATATGTTCGTGTTGTCTCGGA
>SRR4252625_2673078___Sbg4
GGTGGCCCTGGCATTGAGCTGATCACTTA
>SRR4252605_9855879___Sbg4
CTTTGTCTATGTGTTCGTGTTGTCTCGGA
>SRR4252608_8706353___Sbg4
CTTTGTCTATGTGTTCGTGTTGTCTCGGA
>SRR4252610_89799___Sbg4
TGTCTATATGTTCGTGTTGTCTCGGAGGT
>SRR4252605_10205318___Sbg4
GGTCATGTAAATAGTACCTACTAGTGGG
>SRR4252610_11257586___Sbg4
GGTCATGTAAATAGTACCTACTAGTGGG
>SRR4252610_6127608___Sbg4
GGTCATGTAAATAGTACCTACTAGTGGG
>SRR4252607_2560485___Sbg4
ATGTCAGGAAACTTTAGGCCCAAACGGT
>SRR4252605_11728203___Sbg4
TGTGTAGTCAAAAAAGTATGAATGTATA
>SRR4252610_2629391___Sbg4
GGGTGGCGGGAGTCATGTAAATAGTACCTA
>SRR4252605_14036386___Sbg4
AGTCATGTAAATAGTACCTACTAGTGGGG
>SRR4252608_890362___Sbg4
AGTCATGTAAATAGTACCTACTAGTGGGG
>SRR4252611_14717124___Sbg4
AAGACAAATGTCAGGAAACTTTAGGCCCA
>SRR4252606_9380484___Sbg4
TTCGCCTTGTTGGTATGAGTGTGGGTGTA
>SRR4252609_4755240___Sbg4
CTGGGGTTTTTCCTCACCCATAAGACA
>SRR4252608_3972275___Sbg4
AGTCATGTAAATAGTACCTACTAGTGGGG
>SRR4252606_10106910___Sbg4
CAGGCCCAAACGGTCCCCAGACATCA
>SRR4252611_10419074___Sbg4
ACCTACCAGTGGGGGAGGTAAAACTTGGA
>SRR4252609_7523689___Sbg4
CCTACCAGTGGGGGAGGTAAAACTTGGA
>SRR4252606_1730973___Sbg4
ACCTACTAGTGGGGGAGGTAAAACTTGGA
>SRR4252607_14027164___Sbg4
ACATCATCTGGCCATCATGATCATCTTCA
>SRR4252616_282234___Sbg4
GTCTCGGAGGTGGCCCTGGCATTGAGCTG
>SRR4252606_4047760___Sbg4
TCGTCTATATGTTCGTGTTGTCTCGGA
>SRR4252613_9375872___Sbg4
TCGTCTATATGTTCGTGTTGTCTCGGA
>SRR4252609_11066375___Sbg4
TGTCTATATGTTCGTGTTGTCTCGGA
>SRR4252605_11285052___Sbg4
TGTGTAGTCAAAAAAGTATGAATGTATA

>SRR4252606_8561632___Sbg4
GATCCATAGTCTATCCTCTCCCGACAGGT
>SRR4252611_13965939___Sbg4
TCTTGGCTCTGTAAGGCCCGGGTGGCGTG
>SRR4252609_5587339___Sbg4
CATCTGGCCATCATGATCATCTTCAT
>SRR4252605_13924718___Sbg4
TCTTGGCTCTGTAAGGCCCGGGTGGCGTG
>SRR4252615_4562677___Sbg4
TCTTGGCTCTGTAAGGCCCGGGTGGCGTG
>SRR4252610_6528743___Sbg4
ATGGTGTCTTTGTCTATATGTTCGTGTT
>SRR4252625_3407526___Sbg4
TGTCTTTGTCTATATGTTCGTGTTGTCT
>SRR4252609_14589498___Sbg4
CCTTGTGTAGTCAAAAAAGTATGAATGTA
>SRR4252608_10861511___Sbg4
CATCTGGCCATCATGATCATCTTCATCA
>SRR4252609_5496316___Sbg4
TGTCTATATGTTCGTGTTGTCTCGGA
>SRR4252623_7048120___Sbg4
AGTCATGTAAATAGTACCTACTAGTGGGG
>SRR4252607_11838991___Sbg4
TTTAGGCCCAAACGGTCCCCAGACATCATCT
>SRR4252608_6290089___Sbg4
TGTCTATATGTTCGTGTTGTCTCGGAGGT
>SRR4252605_5157711___Sbg4
TCCTTGTGTAGTCAAAAAAGTATGAATGTGT
>SRR4252606_8437588___Sbg4
GATCCATAGTCTATCCTCTCCCGACAGGT
>SRR4252605_12200613___Sbg4
ATCATCTTCATCATATTCGCCTTGTA
>SRR4252611_96432___Sbg4
ATCATCTTCATCATATTCGCCTTGTAGG
>SRR4252610_1464384___Sbg4
CCAGAGAGGCCCTCCATGTCCTTGTGTA
>SRR4252619_4432040___Sbg4
AGAGGCCCTCCATGTCCTTGTGTAGTCAAA
>SRR4252605_13933021___Sbg4
CCGGGTGGCGTGAGTCATGTAAATAGTA
>SRR4252625_3578812___Sbg4
TCCAGGGAGGCCCTCCATGTCCTTGTGTA
>SRR4252610_4831023___Sbg4
AGTCATGTAAATAGTACCTACTAGTGGG
>SRR4252608_2790095___Sbg4
AGTCATGTAAATAGTACCTACTAGTGGGG
>SRR4252605_10330735___Sbg4
TCCAGGGAGGCCCTCCATGTCCTTGTGTA
>SRR4252608_13204227___Sbg4
AGTCATGTAAATAGTACCTACTAGTGGGG
>SRR4252606_1751416___Sbg4
TGGTATGAGTGTGGGTGTGAATGGTGTCTT
>SRR4252619_5109363___Sbg4
CCTACTAGTGGGGGAGGTAAAACTTGGA
>SRR4252606_780721___Sbg4
TGTGGGTGTGAATGGTGTCTTTGTCT
>SRR4252623_10118910___Sbg4
CTCTCTCGACAGGTCTTGGCTCTGTAAG
>SRR4252610_6915690___Sbg4
AGTCATGTAAATAGTACCTACTAGTGGGG
>SRR4252626_5999036___Sbg4
GGAGACCCTCCATGTCCTTGTGTAGTCAA
>SRR4252605_2896040___Sbg4
AGTCATGTAAATAGTACCTACTAGTG
>SRR4252623_915879___Sbg4
GGAAACTTTAGGCCCAAACGGTCCCCAGAC
>SRR4252609_14029780___Sbg4
CTTCATCATATTCGCCTTGTTGGTATGA
>SRR4252610_9246425___Sbg4
GTCTATATGTTCGTGTTGTCTCGGAG

>SRR4252611_8279578___Sbg4
AGGGAGGCCCTCCATGTCCTTGTGTA
>SRR4252611_4833814___Sbg4
TAAGACAAATGTCAGGAAACTTTAGGCCCAA
>SRR4252606_448174___Sbg4
TCCAGGGAGGCCCTCCATGTCCTTGTGT
>SRR4252610_13220531___Sbg4
GGTCATGTAAATAGTACCTACTAGTGGGG
>SRR4252610_4319322___Sbg4
GGTCATGTAAATAGTACCTACTAGTGGGG
>SRR4252610_8206109___Sbg4
GGTCATGTAAATAGTACCTACTAGTGGG
>SRR4252611_13842890___Sbg4
GGTCATGTAAATAGTACCTACTAGTGGGG
>SRR4252613_7725176___Sbg4
TCTATATGTTCGTGTTGTCTCGGAGGTG
>SRR4252611_5599460___Sbg4
TCATGTAAATAGTACCTACTAGTGGGGGA
>SRR4252605_1775480___Sbg4
GCCAGGGAGGCCCTCCATGTCCTTGTGTA
>SRR4252611_821335___Sbg4
GCCAGGGAGGCCCTCCATGTCCTTGTGTA
>SRR4252611_1531047___Sbg4
TTTGTCTATATGTTCGTGTTGTCTCGGA
>SRR4252605_12701491___Sbg4
TCCTTGTGTAGTCAAAAAAGTATGTATGT
>SRR4252607_11108136___Sbg4
TCCTTGTGTAGTCAAAAAAGTATGTATG
>SRR4252612_9182866___Sbg4
TCTGGCCATCATAATCATCTTCATCA
>SRR4252607_7815137___Sbg4
TCCTTGTGTAGTCAAAAAAGTATGAATGT
>SRR4252619_6859977___Sbg4
GGTGTCTTTGTCTATATGTTCGTGTTGTC
>SRR4252610_6957480___Sbg4
GGTCATGTAAATAGTACCTACTAGTGGGG
>SRR4252610_9537133___Sbg4
GGTCATGTAAATAGTACCTACTAGTGGG
>SRR4252610_9908934___Sbg4
GGTCATGTAAATAGTACCTACTAGTGGGG
>SRR4252609_16135349___Sbg4
ACATCATCTGGCCATCATGATCATCTTCA
>SRR4252609_2232837___Sbg4
CCTTGTCTATATGTTCGTGTTGTCTCGGA
>SRR4252611_13803661___Sbg4
ATGTATATGTGATCCATAGTCTATCCT
>SRR4252612_6589577___Sbg4
CATCTGGCCATCATGATCATCTTCATCA
>SRR4252611_14757925___Sbg4
ATCCAGGGAGGCCCTCCATGTCCTTGTGTA
>SRR4252608_5068278___Sbg4
TTCTGGTGTTTTTCCTCACCCATAAGACA
>SRR4252606_9806465___Sbg4
CCATGTCCTTGTGTAGTCAAAAAAGTATG
>SRR4252619_13963434___Sbg4
TGCAGGGAGGCCCTCCATGTCCTTGTGTA
>SRR4252610_7132004___Sbg4
TGTGTAGTCAAAAAAGTATGAATGTATATG
>SRR4252606_4475115___Sbg4
CCGGGTGGCGTGAGTCATGTAAATAGTA
>SRR4252610_6133617___Sbg4
AGTCATGTAAATAGTACCTACTAGTGGG
>SRR4252624_3997705___Sbg4
AGGGAGGCCCTCCATGTCCTTGTGTA
>SRR4252625_981630___Sbg4
GGGTGGCTTGAGTCATGTAAATAGTACCTA
>SRR4252610_2505401___Sbg4
ACCTACTAGTGGGGGAGGTAAAACTTGGA
>SRR4252612_7705368___Sbg4
AATGTCAGGAAACTTTAGGCCCAAACGGT

```
>SRR4252608_4304784___Sbg4
GTCTATATGTTCGTGTTGTCTCGGAGGT
>SRR4252606_165092___Sbg4
TTGTCTCGGAGGTGGCCCTGGCATTGAGC
>SRR4252610_6315267___Sbg4
AGTCATGTAAATAGTACCTACTAGTGGG
>SRR4252618_1364093___Sbg4
GTGTGGGTGTGAATGGTGTCTTTGTCTATA
>SRR4252609_16553547___Sbg4
TCCTTGTGTAGTCAAAAAAGTATGAATG
>SRR4252605_11881690___Sbg4
TCATCTGGCCATCATAATCATCTTCA
>SRR4252608_6339992___Sbg4
TGTCTTTGTCTATATGTTCGTGTTGTCTC
>SRR4252605_11946043___Sbg4
TCCTTGTGTAGTCAAAAAAGTATGAATG
>SRR4252606_4214448___Sbg4
CTTTGTCTTTATGTTCGTGTTGTCTCGGA
>SRR4252610_4544128___Sbg4
AGTCATGTAAATAGTACCTACTAGTGGGG
>SRR4252609_11335880___Sbg4
TCATCTGGCCATCATGATCATCTTCATCATA
>SRR4252610_8146533___Sbg4
AATGTCAGGAAACTTTAGGCCCAAACGG
>SRR4252619_5816428___Sbg4
TTTGTCTATATGTTCGTGTTGTCTCGGA
>SRR4252611_7216869___Sbg4
TCCTTGTGTAGTCAAAAAAGTATGAATGT
>SRR4252608_7558654___Sbg4
TTCATGTCCTTGTGTAGTCAAAAAAGTA
>SRR4252610_10147152___Sbg4
AGTCATGTAAATAGTACCTACTAGTGGG
>SRR4252608_2902982___Sbg4
AGTCATGTAAATAGTACCTACTAGTGGGG
>SRR4252610_1241767___Sbg4
ACCTACTAGTTGGGGAGGTAAAACTTGGA
>SRR4252609_3203742___Sbg4
CATCTGGCCATCATAATCATCTTCATCA
>SRR4252625_9408092___Sbg4
TCCAGGGAGGCCCTCCATGTCCTTGTGTA
>SRR4252611_1433880___Sbg4
TCCTTGTGTAGTCAAAAAAGTATGAATGT
>SRR4252611_15510778___Sbg4
ACAAATGTCAGGAAACTTTAGGCCCAAA
>SRR4252625_8273520___Sbg4
CTTTGTCTATATGTTCGTGTTGTCTCGGA
>SRR4252605_8918634___Sbg4
CCATAAGACAAATGTCAGGAAACTTTAGG
>SRR4252610_2606011___Sbg4
GTGGCGTGAGTCATGTAAATAGTACCTA
>SRR4252611_8230705___Sbg4
AGGAGGCCCTCCATGTCCTTGTGTAGTCA
>SRR4252610_926673___Sbg4
ATTTGTCTATATGTTCGTGTTGTCTCGGA
>SRR4252619_11610713___Sbg4
CCTTGTTGGTATGAGTGTGGGTGTGA
>SRR4252605_640101___Sbg4
CCTTGTCTATATGTTCGTGTTGTCTCGGA
>SRR4252607_192192___Sbg4
AGTCATGTAAATAGTACCTACTAGTGGGG
>SRR4252610_9097273___Sbg4
AGTCATGTAAATAGTACCTACTAGTGGGG
>SRR4252611_7725176___Sbg4
TCCTTGTGTAGTCAAAAAAGTATGAATGT
>SRR4252612_5948497___Sbg4
ATGTCAGGAAACTTTAGGCCCAAACGGT
>SRR4252612_6020930___Sbg4
TTGTCTCGGAGGTGGCCCTAGCATTGAGC
>SRR4252618_9998883___Sbg4
TCTTGGCTCTGTAAGGCCCGGGTGGCGC
```

>SRR4252616_114813___Sbg4
CTTGGCTCTGTAAGGCCCGGGTGGCGC
>SRR4252605_2788840___Sbg4
TTGGCTCTGTAAGGCCCGGGTGGCGC
>SRR4252610_3668553___Sbg4
CATCATCTGGCCATCATGATCATCTTCA
>SRR4252606_8175992___Sbg4
GGGAGGCCCTCCATGTCCTTGTGTAGTCA
>SRR4252610_11563800___Sbg4
CCATGTCCTTGTGTAGTCAAAAAAGTA
>SRR4252609_3158344___Sbg4
TCCTTGTGTAGTCAAAAAAGTATGAAT
>SRR4252608_7723056___Sbg4
TCCTTGTGTAGTCAAAAAAGTATGAATGC
>SRR4252605_1318879___Sbg4
AGTCATGTAAATAGTACCTACTAGTGGGG
>SRR4252614_1587941___Sbg4
ATCTGGCCATCATGATCATCTTCATCAT
>SRR4252605_8971447___Sbg4
CCTTGTCTAGTCAAAAAAGTATGAATGTA
>SRR4252611_14824416___Sbg4
TCTTGGCTCTGTAAGGCCCGGGTGGCGTGA
>SRR4252608_6711812___Sbg4
AGTCATGTAAATAGTACCTACTAGTGGG
>SRR4252608_9644619___Sbg4
AGTCATGTAAATAGTACCTACTAGTGGGG
>SRR4252611_8472834___Sbg4
AATGTCAGGAAACTTTAGGCCCAAACGGTC
>SRR4252605_14548787___Sbg4
TTTGTCTATATGTTCGTGTTGTCTCGGA
>SRR4252606_10120344___Sbg4
AGTCATGTAAATAGTACCTACTAGTGGGG
>SRR4252606_433111___Sbg4
ACCTCCATGTCCTTGTGTAGTCAAAA
>SRR4252618_4340667___Sbg4
TCTTGGCTCTGTAAGGCCCAGGTGGCGTGA
>SRR4252608_11259685___Sbg4
AGTCATGTAAATAGTACCTACTAGTGGG
>SRR4252611_13597312___Sbg4
TCTTGGCTCTGTAAGGCCCGGGTGGCGTA
>SRR4252605_7148842___Sbg4
ATACCTACTAGTGGGGGAGGTAAAACTTGG
>SRR4252611_1794265___Sbg4
CCATAAGACAAATGTCAGGAAACTTTAG
>SRR4252611_14353321___Sbg4
CCCCAGACATCATCTGGCCATCATGATCA
>SRR4252610_3574268___Sbg4
CTTTGTCTATATGTTCGTGTTGTCTCGGA
>SRR4252611_3988788___Sbg4
TCCTTGTGTAGTCAAAAAAGTATGAATGT
>SRR4252611_13570105___Sbg4
TCTTGGCTCTGTAAGGCCCGGGTGGCGTGA
>SRR4252610_8299202___Sbg4
AGTCATGTAAATAGTACCTACTAGTGGGG
>SRR4252606_4624013___Sbg4
TTGTATATATGTTCGTGTTGTCTCGGA
>SRR4252610_7466321___Sbg4
GGGGAAGCCCTCCATGTCCTTGTGTA
>SRR4252612_3936901___Sbg4
TGAGTGTGGGTGTGAATGGTGTTTTT
>SRR4252611_6034749___Sbg4
ACATCATCTGGCCATCATGATCATCTTCA
>SRR4252614_6858212___Sbg4
ATTTGTCTATATGTTCGTGTTGTCTCGGA
>SRR4252616_13488359___Sbg4
ATGTCCTTGTGTAGTCAAAAAAGTATGA
>SRR4252610_9213080___Sbg4
CTTTGTCTATATGTTCGTGTTGTCTCGGA
>SRR4252626_9631965___Sbg4
TCTCGGAGGTGGCCCTGGCATTGAGCTG

>SRR4252611_14401708___Sbg4
AATGTCAGGAAACTTTAGGCCCAAACGGT
>SRR4252610_3800390___Sbg4
GCATCATCTGGCCATCATGATCATCTTCA
>SRR4252623_13519907___Sbg4
GCATCATCTGGCCATCATGATCATCTTCA
>SRR4252617_8820927___Sbg4
TCTTGGCTCTGTAAGGCCCGGGTGGCGTGA
>SRR4252621_5873004___Sbg4
TTTTTCCTCACCCATAAGACAAATGTCAG
>SRR4252622_7850071___Sbg4
TCCTTGTGTAGTCAAAAAAGTATGAATG
>SRR4252610_10881789___Sbg4
TCCTTGTGTAGTCAAAAAAGTATGTATG
>SRR4252624_3409132___Sbg4
TGTGGGTGTGAATGGTGTCTTTGTCTATA
>SRR4252606_2399704___Sbg4
TCTTTGTCTATATGTTCGTGTTGTCT
>SRR4252610_9474889___Sbg4
CTTTGTCTATATGTTCGTGTTGTCTCGGA
>SRR4252609_2851463___Sbg4
TTGTCTCGGAGGTGGCCCTGGCATTGAGC
>SRR4252609_569551___Sbg4
CATCATCTGGCCATCATGATCATCTTCA
>SRR4252610_12389477___Sbg4
AGTCATGTAAATAGTACCTACTAGTGGG
>SRR4252610_492440___Sbg4
AGTCATGTAAATAGTACCTACTAGTGGGG
>SRR4252610_3440042___Sbg4
TCATCTGGCCATCATAATCATCTTCATCA
>SRR4252624_4509265___Sbg4
AGGGAGGCCCTCCATGTCCTTGTGTA
>SRR4252605_5591219___Sbg4
CTTTGTCTTTATGTTCGTGTTGTCTCGGA
>SRR4252606_3944788___Sbg4
CTTTGTCTTTATGTTCGTGTTGTCTCGGA
>SRR4252609_13821596___Sbg4
CTTTGTCTTTATGTTCGTGTTGTCTCGGA
>SRR4252606_2437206___Sbg4
TGTCTTTATGTTCGTGTTGTCTCGGAG
>SRR4252610_12433093___Sbg4
CATCATCTGGCCATCATGATCATCTTCA
>SRR4252610_1647986___Sbg4
TGTCTATATGTTCGTGTTGTCTCGGA
>SRR4252605_9210171___Sbg4
AGTCATGTAAATAGTACCTACTAGTGGGG
>SRR4252614_6707989___Sbg4
ATGGTGTCTTTGTCTATATGTTCGTGT
>SRR4252611_13808100___Sbg4
TCCAGGGAGGCCCTCCATGTCCTTGTGTA
>SRR4252611_8474644___Sbg4
CCATGTCCTTGTGTAGTCAAAAAAGTA
>SRR4252611_1548068___Sbg4
TTTGTCTATATGTTCGTGTTGTCTCGGAG
>SRR4252611_9889327___Sbg4
TTTGTCTATATGTTCGTGTTGTCTCGG
>SRR4252610_8909869___Sbg4
TGTCTATATGTTCGTGTTGTCTCGGA
>SRR4252607_1616083___Sbg4
TCCTTGTGTAGTCAAAAAAGTATGAAT
>SRR4252613_4432884___Sbg4
TCTTTGTCTATATGTTCGTGTTGTCTCGGA
>SRR4252612_4606963___Sbg4
TGATCACTTATCCAGGGAGGCCCTCCATG
>SRR4252608_3373070___Sbg4
AGTCATGTAAATAGTACCTACTAGTGGGG
>SRR4252609_10828212___Sbg4
AGTCATGTAAATAGTACCTACTAGTGGG
>SRR4252612_5180715___Sbg4
ATCATCTGGCCATCATGATCATCTTCA

>SRR4252608_463409___Sbg4
TCGTGTTGTCTCGGAGGTGGCCCTGGCA
>SRR4252607_9506010___Sbg4
TATCATCTGGCCATCATGATCATCTTCA
>SRR4252621_13097164___Sbg4
TGTGGGTGTGAATGGTGTCTTTGTCTA
>SRR4252611_13513029___Sbg4
TGAGGCCCTCCATGTCCTTGTGTAGTCAAA
>SRR4252607_5638574___Sbg4
GTGTAGTCAAAAAAGTATGAATGTATATGTG
>SRR4252606_6573488___Sbg4
AGGGAAGCCCTCCATGTCCTTGTGTA
>SRR4252621_2591471___Sbg4
GGCCCGGGTGGCGTGAGTCATGTAAA
>SRR4252612_337709___Sbg4
AATGTCAGGAAACTTTAGGCCCAAACGA
>SRR4252609_10373330___Sbg4
AATGTCAGGAAACTTTAGGCCCAAACGGT
>SRR4252614_3540947___Sbg4
AATGTCAGGAAACTTTAGGCCCAAACG
>SRR4252610_11441085___Sbg4
TCTTTGTCTATATGTTCGTGTTGTCT
>SRR4252611_10983479___Sbg4
ACATCATCTGGCCATCATGATCATCTTCA
>SRR4252611_12347906___Sbg4
TCCAGGGAGGCCCTCCATGTCCTTGTGTA
>SRR4252605_1547178___Sbg4
ATGGTGTCTTTGTCTATATGTTCGTGT
>SRR4252609_4957941___Sbg4
TTCTGGAGTTTTTCCTCACCCATAAGACA
>SRR4252605_1183447___Sbg4
TCATCTGGCCATCATGATCATCTTCATCA
>SRR4252610_12121981___Sbg4
TCCTTGTGTAGTCAAAAAAGTATGAATG
>SRR4252613_6096747___Sbg4
GGAGGCCCTCCATGTCCTTGTGTAGTC
>SRR4252610_4909975___Sbg4
AGTCATGTAAATAGTACCTACTAGTGGG
>SRR4252606_5546109___Sbg4
CTTTGTCTTTATGTTCGTGTTGTCTCGGA
>SRR4252613_2133078___Sbg4
AGGGAGGCCCTCCATGTCCTTGTGTA
>SRR4252622_2832796___Sbg4
TCCTTGTGTAGTCAAAAAAGTATGAATG
>SRR4252610_3166193___Sbg4
GGTGGCGTGAGTCATGTAAATAGTACCTA
>SRR4252610_4682808___Sbg4
AGTCATGTAAATAGTACCTACTAGTGGG
>SRR4252605_7020984___Sbg4
CCATAGTCTATCCTCTCCCGACAGGTCT
>SRR4252624_7170790___Sbg4
TCCAGGGAGGCCCTCCATGTCCTTGTGTA
>SRR4252605_7557570___Sbg4
AGGGAGGCCCTCCATGTCCTTGTGTA
>SRR4252607_7749507___Sbg4
TCCTTGTGTAGTCAAAAAAGTATGAAT
>SRR4252605_12905291___Sbg4
ACATCATCTGGCCATCATGATCATCTTCA
>SRR4252608_294504___Sbg4
ATCACTTATCCAGGGAGGCCCTCCATG
>SRR4252621_7502771___Sbg4
TCCAGGGAGGCCCTCCATGTCCTTGTGTA
>SRR4252608_10539324___Sbg4
GTTGTCTCGGAGGTGGCCCTGGCATTGAGC
>SRR4252616_8272795___Sbg4
TGATCCATAGTCTATCCTCTCCCGACAG
>SRR4252612_3312278___Sbg4
ATCTCATCTGGCCATCATGATCATCTTCA
>SRR4252612_7667318___Sbg4
ATGTCAGGAAACTTTAGGCCCAAACGGT

>SRR4252610_12233198___Sbg4
GTGTGGGTGTGAATGGTGTCTTTGTCTATA
>SRR4252619_38928___Sbg4
CCATAGTCTATCCTCTCCCGACAGGTC
>SRR4252622_2069851___Sbg4
TTGTCTCGGAGGTGGCCCTGGCATTGAGC
>SRR4252609_14956825___Sbg4
TTCCAGGGAGGCCCTCCATGTCCTTGTGTA
>SRR4252611_13083287___Sbg4
TTCCAGGGAGGCCCTCCATGTCCTTGTGTA
>SRR4252624_4267044___Sbg4
CATCTGGCCATCATGATCATCTTCATCA
>SRR4252605_6056214___Sbg4
TTGTCTATATGTTCGTGTTGTCTCGGA
>SRR4252609_10872702___Sbg4
AGTCATGTAAATAGTACCTACTAGTGGG
>SRR4252611_1516474___Sbg4
TTTGTCTATATGTTCGTGTTGTCTCGGAG
>SRR4252620_5537503___Sbg4
TGTCAGGAAACTTTAGGCCCAAACGGTCC
>SRR4252611_1823964___Sbg4
ACATCATCTGGCCATCATGATCATCTTCA
>SRR4252610_6307171___Sbg4
ATCCAGGGGGGCCCTCCATGTCCTTGTGTA
>SRR4252605_1683798___Sbg4
TCCAGGGAGGCCCTCCATGTCCTTGTGTA
>SRR4252606_558824___Sbg4
AGGGAGGCCCTCCATGTCCTTGTGTA
>SRR4252619_14331639___Sbg4
AGTCATGTAAATAGTACCTACTAGTGGGG
>SRR4252608_349829___Sbg4
TCATCTGGCCATCATAATCATCTTCATCA
>SRR4252610_811141___Sbg4
TGTAGTCAAAAAAGTATGAATGTATATGTA
>SRR4252621_8214941___Sbg4
GGTGTCTTTGTCTATATGTTCGTGTTGTCTC
>SRR4252611_1939959___Sbg4
AATGTCAGGAAACTTTAGGCCCAAACGGT
>SRR4252612_7270826___Sbg4
AATGTCAGGAAACTTTAGGCCCAAACGGT
>SRR4252605_14589380___Sbg4
GTTGGTATGAGTGTGGGTGTGAATGGTGTC
>SRR4252609_693020___Sbg4
AGTCATATAAATAGTACCTACTAGTGGG
>SRR4252608_2353927___Sbg4
AGTCATGTAAATAGTACCTACTAGTGGG
>SRR4252612_5880412___Sbg4
TGTCTATATGTTCGTGTTGTCTCGGAGGT
>SRR4252610_6855550___Sbg4
TCCAGGGAGGCCCTCCATGTCCTTGTGTA
>SRR4252610_1708378___Sbg4
TCATGTAAACAGTACCTACTAGTGGGGGA
>SRR4252610_6043754___Sbg4
CTACTAGTGGGGGAGGTAAAACTTGGA
>SRR4252620_10555265___Sbg4
TCAGGAAACTTTAGGCCCAAACGGTCCCCA
>SRR4252619_11060067___Sbg4
CTTCATCATATTCGCCTTGTTGGTATGAGTG
>SRR4252610_11663671___Sbg4
TTTGTCTATATGTTCGTGTTGTCTCGG
>SRR4252619_5947100___Sbg4
AGCTCTGTAAGGCCCGGGTGGCGTGAGTCA
>SRR4252608_752647___Sbg4
CCTTGTCTATATGTTCGTGTTGTCTCGGA
>SRR4252610_6841113___Sbg4
TCCTTGTGTAGTCAAAAAAGTATGAATGT
>SRR4252612_4260268___Sbg4
ATCCTCTCCCGACAGGTCTTGGCTCTGT
>SRR4252611_11506980___Sbg4
GTGTGGGTGTGAATGGTGTCTTTGTCTATA

>SRR4252610_11690085___Sbg4
AGTCATGTAAATAGTACCTACTAGTGGGG
>SRR4252611_4932259___Sbg4
TTCTTCATATTCGCCTTGTTGGTATG
>SRR4252606_2399890___Sbg4
TTTGTCTATATGTTCGTGTTGTCTCGGAGGT
>SRR4252610_11060579___Sbg4
ACACCATCTGGCCATCATGATCATCTTCA
>SRR4252609_8044518___Sbg4
CACCATCTGGCCATCATGATCATCTTCA
>SRR4252607_12286544___Sbg4
ACATCATCCGGCCATCATGATCATCTTCA
>SRR4252607_6924481___Sbg4
ACATCATCCGGCCATCATGATCATCTTCA
>SRR4252610_1002590___Sbg4
ACATCATCCGGCCATCATGATCATCTTCA
>SRR4252610_4257780___Sbg4
ACATCATCCGGCCATCATGATCATCTTCA
>SRR4252610_6292553___Sbg4
ACATCATCCGGCCATCATGATCATCTTCA
>SRR4252621_5481322___Sbg4
ACATCATCCGGCCATCATGATCATCTTCA
>SRR4252610_12694893___Sbg4
CATCATCCGGCCATCATGATCATCTTCA
>SRR4252622_9025169___Sbg4
CATCATCCGGCCATCATGATCATCTTCA
>SRR4252606_3615392___Sbg4
AGTCATGTAAATAGTACCTACTAGTGGG
>SRR4252608_8705031___Sbg4
TCAGGGAGGCCCTCCATGTCCTTGTGTA
>SRR4252607_5718594___Sbg4
TCTTTGTTTATATGTTCGTGTTGTCTCGGA
>SRR4252610_5880729___Sbg4
AGTCATGTAAATAGTACCTACTAGTGGG
>SRR4252625_10055138___Sbg4
TAAGACAAATGTCAGGAAACTTTAGGCCC
>SRR4252610_3940268___Sbg4
GGTGTCTTTGTCTATATGTTCGTGTT
>SRR4252606_6721414___Sbg4
TCCATGTCCTTGTGTAGTCAAAAAAGTA
>SRR4252611_82817___Sbg4
AAGGCCCGGGTGGCGTGAGTCATGTAAAT
>SRR4252607_3079778___Sbg4
TTGTCTCGGAGGTGGCCCTGGCATTGAGC
>SRR4252605_14519429___Sbg4
AGTCATGTAAATAGTACCTACTAGTGGG
>SRR4252605_8174117___Sbg4
TCATGTAAATAGTACCTACTAGTGGGGGA
>SRR4252622_11196405___Sbg4
TCTTTGTCTATATGTTCGTGTTGTCTCGGA
>SRR4252623_14890096___Sbg4
TCCTTGTGTAGTCAAAAAAGTATGAATGT
>SRR4252605_8386429___Sbg4
AGTACCTACTAGTGGGGGAGGTAAAACTTGG
>SRR4252609_4502196___Sbg4
AGGGAGGCCCTCCATGTCCTTGTGTA
>SRR4252605_13229133___Sbg4
AGTCATGTAAATAGTACCTACTAGTGGGG
>SRR4252611_10451849___Sbg4
AGTCATGTAAATAGTACCTACTAGTGGGG
>SRR4252605_5444087___Sbg4
TGTCATGTAAATAGTACCTACTAGTGGG
>SRR4252610_10324099___Sbg4
TGTCATGTAAATAGTACCTACTAGTGGG
>SRR4252610_2469386___Sbg4
TGTCATGTAAATAGTACCTACTAGTGGG
>SRR4252610_4365919___Sbg4
TGTCATGTAAATAGTACCTACTAGTGGG
>SRR4252612_6528012___Sbg4
GGGGCCCTCCATGTCCTTGTGTAGTCAAAA

>SRR4252606_1626596___Sbg4
GGGCCCTCCATGTCCTTGTGTAGTCAA
>SRR4252614_4659734___Sbg4
TTGTCTATATGTTCGTGTTGTCTCGGAG
>SRR4252611_11981922___Sbg4
CCTACTAGTGGGGGAGGTAAAACTTGGA
>SRR4252612_7944424___Sbg4
TCTTGGCTCTGTAAGGCCCGGGTGGCGTG
>SRR4252611_2815861___Sbg4
ACATCATCTGGCCATCATGATCATCTTCA
>SRR4252610_10045692___Sbg4
TCCTTGTGTAGTCAAAAAAGTATGAATG
>SRR4252620_172927___Sbg4
TGTCAGGAAACTTTAGGCCCAAACGG
>SRR4252610_5037962___Sbg4
AGTCATGTAAATAGTACCTACTAGTGGGG
>SRR4252607_8751612___Sbg4
ACATCATCTGGCCATCATGATCATCTTCA
>SRR4252610_6676563___Sbg4
ACATCATCTGGCCATCATGATCATCTTCA
>SRR4252611_15130156___Sbg4
CCACAGTCTATCCTCTCCCGACAGGTC
>SRR4252611_2506626___Sbg4
CTGGGGTTTTTCCTCACCCATAAGACA
>SRR4252611_11460761___Sbg4
CCATGTCCTTGTGTAGTCAAAAAAGTA
>SRR4252609_16977596___Sbg4
GACAAACGTCAGGAAACTTTAGGCCCAAA
>SRR4252624_5940928___Sbg4
TCTGGCCATCATAATCATCTTCATCATA
>SRR4252611_2240878___Sbg4
TCCTTGTGTAGTCAAAAAAGTATGAATGT
>SRR4252610_1651474___Sbg4
TTGGCTCTGTAAGGCCCGGGTGGCGA
>SRR4252605_14628015___Sbg4
CGTCATGTAAATAGTACCTACTAGTGGG
>SRR4252605_9227258___Sbg4
CGTCATGTAAATAGTACCTACTAGTGGGG
>SRR4252610_705077___Sbg4
CGTCATGTAAATAGTACCTACTAGTGGG
>SRR4252623_2235911___Sbg4
TTTGTCTATATGTTCGTGTTGTCTCGGAG
>SRR4252606_3595092___Sbg4
AGTCATGTAAATAGTACCTACTAGTGGG
>SRR4252610_1339611___Sbg4
AGGGAGGCCCTCCATGTCCTTGTGTA
>SRR4252605_4060167___Sbg4
TCCTTGTGTAGTCAAAAAAGTATGTATGT
>SRR4252611_7569605___Sbg4
TCCTTGTGTAGTCAAAAAAGTATGTATG
>SRR4252613_7690731___Sbg4
GTCTATATGTTCGTGTTGTCTCGGAG
>SRR4252611_8945542___Sbg4
AGTCATGTAAATAGTACCTACTAGTGGG
>SRR4252612_451094___Sbg4
TCTTTGTCCATATGTTCGTGTTGTCTCGGA
>SRR4252611_979508___Sbg4
TTGTCCATATGTTCGTGTTGTCTCGGA
>SRR4252611_12401101___Sbg4
TTTGTCTATATGTTCGTGTTGTCTCGGAG
>SRR4252623_1260797___Sbg4
AGGCCCTCCATGTCCTTGTGTAGTCAAA
>SRR4252608_1468119___Sbg4
TCCTTGTGTAGTCAAAAAAGTATGAATGT
>SRR4252616_7860387___Sbg4
TCTCCCGACAGGTCTTGGCTCTGTAAGGC
>SRR4252605_4504438___Sbg4
CAGGTCTTGGCTCTGTAAGGCCCGGG
>SRR4252610_1340075___Sbg4
GGCGTGAGTCATGTAAATAGTACCTACTAG

>SRR4252614_8570138___Sbg4
ACTAGTGGGGGAGGTAAAACTTGGAGAAT
>SRR4252610_10405128___Sbg4
GTGGGTGTGAATGGTGTCTTTGTCTT
>SRR4252605_10883720___Sbg4
CTGTCTATATGTTCGTGTTGTCTCGGAG
>SRR4252622_5766772___Sbg4
CTTCATCATATTCGCCTTGTTGGTAC
>SRR4252611_3484083___Sbg4
CCAGGGAGACCCTCCATGTCCTTGTGTA
>SRR4252605_10440514___Sbg4
TGTCTATATGTTCGTGTTGTCTCGGAGG
>SRR4252612_2532208___Sbg4
AGGGAGGCCCTCCATGTCCTTGTGTA
>SRR4252606_3166007___Sbg4
GGGAGGCCCTACATGTCCTTGTGTAGTCA
>SRR4252605_14280525___Sbg4
ACATGTCCTTGTGTAGTCAAAAAAGTATGA
>SRR4252610_6072225___Sbg4
CTTTGTCTATATGTTCGTGTTGTCTCGGA
>SRR4252611_1768700___Sbg4
GGTGGCATGAGTCATGTAAATAGTACCTA
>SRR4252608_10364537___Sbg4
ATCATCTGGCCATCATAATCATCTTCA
>SRR4252611_14360888___Sbg4
CCAGGGAGGCCCTCCATGTCCTTGTGTA
>SRR4252610_10910428___Sbg4
TGTCATGTAAATAGTACCTACTAGTGGGG
>SRR4252610_3638930___Sbg4
TGTCATGTAAATAGTACCTACTAGTGGG
>SRR4252610_8169848___Sbg4
TGTCATGTAAATAGTACCTACTAGTGGG
>SRR4252605_9850624___Sbg4
CTTCGTCTATATGTTCGTGTTGTCTCGGA
>SRR4252605_13619748___Sbg4
TCCTTGTGTAGTCAAAAAAGAATGAAT
>SRR4252610_3371633___Sbg4
CATCTTCATCATATTCGCCTTGTTGGTA
>SRR4252622_7186133___Sbg4
TCCTTGTGTAGTCAAAAATGTATGAATG
>SRR4252610_3004400___Sbg4
ACCTACTAGTGGGGGAGGTAAAACTTGGA
>SRR4252605_5519738___Sbg4
TCATCATCTGGCCATCATGATCATCTTCA
>SRR4252611_574733___Sbg4
TCATCATCTGGCCATCATGATCATCTTCA
>SRR4252610_12772502___Sbg4
TAGTCTATATGTTCGTGTTGTCTCGGA
>SRR4252605_5604740___Sbg4
AGTCATGTAAATAGTACCTACTAGTGGGG
>SRR4252616_6642655___Sbg4
GGAGGCTCTCCATGTCCTTGTGTAGTCA
>SRR4252611_7111010___Sbg4
CCTTGTGTAGTCAAAAAAGTATGAATGTA
>SRR4252619_7240977___Sbg4
TATGTTCGTGTTGTCTCGGAGGTGGC
>SRR4252612_4771272___Sbg4
TAGGCCCAAACGGTCCCCAGACATCA
>SRR4252610_13132317___Sbg4
TGTCATGTAAATAGTACCTACTAGTGGGG
>SRR4252610_3657307___Sbg4
TGTCATGTAAATAGTACCTACTAGTGGG
>SRR4252610_9301192___Sbg4
TGTCATGTAAATAGTACCTACTAGTGGG
>SRR4252612_2754623___Sbg4
TGTCATGTAAATAGTACCTACTAGTGGGG
>SRR4252612_2737859___Sbg4
AATGTCAGGAAACTTTAGGCCCAAACGGT
>SRR4252610_7552517___Sbg4
TTGTCTCGGAGGTGGCCCTGGCATTGAGC

```
>SRR4252623_13462887___Sbg4
GTGTGGGTGTGAATGGTGTCTTTGTCTATA
>SRR4252610_12715324___Sbg4
ACATCATCTGGCCATCATGATCATCTTCA
>SRR4252610_3422962___Sbg4
AGTCATGTAAATAGTACCTACTAGTGGG
>SRR4252606_346516___Sbg4
GAGACAAATGTCAGGAAACTTTAGGCCCA
>SRR4252610_1017935___Sbg4
TCTTTGTCTATATGTTCGTGTTGTCTCGGA
>SRR4252610_11133777___Sbg4
AATGTCAGGAAACTTTAGGCCCAAAC
>SRR4252610_11364477___Sbg4
TGTCAGGAAACTTTAGGCCCAAACGGT
>SRR4252605_10531153___Sbg4
TTTGTCTATATGTTCGTGTTGTCTCGGAGA
>SRR4252611_11724949___Sbg4
CCATGTCCTTGTGTAGTCAAAAAAGTA
>SRR4252610_3883904___Sbg4
TCCTTGTGTAGTCAAAAAAGTATGAATGC
>SRR4252614_6937662___Sbg4
TCCTTGTGTAGTCAAAAAAGTATGAATGC
>SRR4252605_7049896___Sbg4
TTGTCTCGGAGGTGGCCCTGGCATTGAGC
>SRR4252617_14818559___Sbg4
ATGAGTGTGGGTGTGAATGGTGTCTTTGTCT
>SRR4252609_16385681___Sbg4
ACATCATCTGGCCATCATGATCATCTTC
>SRR4252619_6223986___Sbg4
TCTCCCGACAGGTCTTGGCTCTGTAAGGCT
>SRR4252606_6912180___Sbg4
AGTCATGTAAATAGTACCTACTAGTGGG
>SRR4252612_477731___Sbg4
CGGGTGGCGTGAGTCATGTAAATAGTA
>SRR4252610_7144354___Sbg4
AGTCATGTAAATAGTACCTACTAGTGGGG
>SRR4252608_8413312___Sbg4
TGTGGGTGTGAATGGTGTCTTTGTCTAT
>SRR4252622_8195856___Sbg4
TGTGTAGTCAAAAAAGTATGAATGTATAT
>SRR4252606_391285___Sbg4
TTTGGCTCTGTAAGGCCCGGGTGGCGTGA
>SRR4252608_4188241___Sbg4
TCGTCTATATGTTCGTGTTGTCTCGGA
>SRR4252609_14038727___Sbg4
TCCAGGGAGGCCCTCCATGTCCTTGTGTA
>SRR4252607_984249___Sbg4
TCCTTGTGTAGTCAAAAAAGTATGAAT
>SRR4252606_5591482___Sbg4
TTCGTCTATATGTTCGTGTTGTCTCGGA
>SRR4252611_10154771___Sbg4
TCCTTGTGTAGTCAAAAAAGTATGAA
>SRR4252610_7655998___Sbg4
AGTCATGTAAATAGTACCTACTAGTGGGG
>SRR4252610_219238___Sbg4
ATCATGTAAATAGTACCTACTAGTGG
>SRR4252607_14870027___Sbg4
TCTTTGTCTATATGTTCGTGTTGTCTC
>SRR4252610_9775553___Sbg4
TCCTTGTGTAGTCAAAAAAGTATGAATG
>SRR4252621_2972071___Sbg4
CTCTCCCGACAGGTCTTGGCTCTGTAAG
>SRR4252611_8708452___Sbg4
GCCTACTAGTGGGGGAGGTAAAACTTGGA
>SRR4252605_14105349___Sbg4
TTAAGGGAGGCCCTCCATGTCCTTGTGTA
>SRR4252608_12375685___Sbg4
TAAGGGAGGCCCTCCATGTCCTTGTGTA
>SRR4252622_10578478___Sbg4
TAAGGGAGGCCCTCCATGTCCTTGTGTA
```

>SRR4252610_1169735___Sbg4
AAGGGAGGCCCTCCATGTCCTTGTGTA
>SRR4252610_7611709___Sbg4
AAGGGAGGCCCTCCATGTCCTTGTGTA
>SRR4252608_3735225___Sbg4
TTGTCTCGGAGGTGGCCCTGGCATTGAGC
>SRR4252625_4242730___Sbg4
CCCGGGTGGCGTGAGTCATGTAAATAGTA
>SRR4252606_10196242___Sbg4
ATAGTCTATCCTCTCCCGACAGGTCTTG
>SRR4252611_1686610___Sbg4
CCAGGGAGGCCCTCCATGTCCTTGTGTA
>SRR4252605_15111203___Sbg4
AAAGACAAATGTCAGGAAACTTTAGGCCCA
>SRR4252606_2746774___Sbg4
AAAGACAAATGTCAGGAAACTTTAGGCCCA
>SRR4252611_1076224___Sbg4
AAAGACAAATGTCAGGAAACTTTAGGCCCA
>SRR4252612_7455833___Sbg4
GACAAATGTCAGGAAACTTTAGGCCCAA
>SRR4252611_7015744___Sbg4
ACATCATCTGGCCATCATGATCATCTTCA
>SRR4252609_9532296___Sbg4
CTTGTCTATATGTTCGTGTTGTCTCGGAG
>SRR4252611_7267871___Sbg4
TCTTGGCTCTGTAAGGCCCGGGTGGCGTGA
>SRR4252607_14854062___Sbg4
AGTCATATAAATAGTACCTACTAGTGGGG
>SRR4252608_6232557___Sbg4
GTGGGTGTGAATGGTGTCTTTGTCTATA
>SRR4252606_1495517___Sbg4
AGTCATGTAAATAGTACCTACTAGTGGGG
>SRR4252616_9423330___Sbg4
GCTTATCCAGGGAGGCCCTCCATGTCCTTG
>SRR4252609_3060584___Sbg4
AATGACAAATGTCAGGAAACTTTAGGCCCA
>SRR4252611_10030616___Sbg4
TCCAGGGAGGTCCTCCATGTCCTTGTGTA
>SRR4252605_13933802___Sbg4
GGTCATGTAAATAGTACCTACTAGTGGGG
>SRR4252605_1478044___Sbg4
GGTCATGTAAATAGTACCTACTAGTGGGG
>SRR4252605_1899809___Sbg4
GGTCATGTAAATAGTACCTACTAGTGGGG
>SRR4252611_6621313___Sbg4
GGTCATGTAAATAGTACCTACTAGTGGG
>SRR4252606_7081336___Sbg4
TACCTACTAGTGGGGGAGGTAAAACTTGGA
>SRR4252610_4632456___Sbg4
TGTCTTTGTCTATATGTTCGTGTTGTCT
>SRR4252623_10179182___Sbg4
TCCTTGTGTAGTCAAAAAAGTATGAATG
>SRR4252620_10547443___Sbg4
TTTGTCTATATGTTCGTGTTGTCTCGGAGT
>SRR4252612_7979695___Sbg4
AGGGAGGCCCTCCATGTCCTTGTGTA
>SRR4252606_6569181___Sbg4
TTGTCTATATGTTCGTGTTGTCTCGGAG
>SRR4252625_12862344___Sbg4
TCCAGGGAGGCCCTCCATGTCCTTGTGTA
>SRR4252610_9330875___Sbg4
AATGTCAGGAAACTTTAGGCCCAAACGGC
>SRR4252606_8555521___Sbg4
TGTCAGGAAACTTTAGGCCCAAACGGC
>SRR4252610_11048209___Sbg4
CCTACTAGTGGGGGAGGTAAAACTTGGA
>SRR4252605_13612791___Sbg4
TCATCATCATCATATTCGCCTTGTTGGTA
>SRR4252606_8006198___Sbg4
AGTCATGTAAATAGTACCTACTAGTGGG

>SRR4252605_2830510___Sbg4
AGGGAGGCCCTCCATGTCCTTGTGTA
>SRR4252610_13148144___Sbg4
TTGTCTATATGTTCGTGTTGTCTCGGA
>SRR4252605_12703049___Sbg4
ACTAGTGGGGGAGGTAAAACTTGGAA
>SRR4252605_9885882___Sbg4
TTGTCTCGGAGGTGGCCCTGGCATTG
>SRR4252621_9639642___Sbg4
ATGTGATCCATAGTCTATCCTCTCCCGA
>SRR4252605_2556672___Sbg4
TACCTACTAGTGGGGGAGGTAAAACTTGG
>SRR4252607_13238197___Sbg4
TCTCGGAGGTGGCCCTGGCATTGAGC
>SRR4252623_11991651___Sbg4
TCCTTGTGTAGTCAAAAAGTATGAATG
>SRR4252605_7982246___Sbg4
CAAACGGTCCCCAGACATCATCTGGC
>SRR4252616_7810487___Sbg4
TCCTTGTGTAGTCAAAAAGTATGAATGT
>SRR4252610_11009451___Sbg4
GGGTGGCGTGAGTCATGTAAATAGTACCTA
>SRR4252607_4530991___Sbg4
AGTCATGTAAATAGTACCTACTAGTGGG
>SRR4252612_5450476___Sbg4
ACCCTCCATGTCCTTGTGTAGTCAAA
>SRR4252605_3472679___Sbg4
AGTCATGTAAATAGTACCTACTAGTGGG
>SRR4252610_5982199___Sbg4
AGTCATGTAAATAGTACCTACTAGTGGG
>SRR4252611_8510485___Sbg4
CATCTGGCCATCATGATCATCTTCATCA
>SRR4252610_6794262___Sbg4
TCCCGACAGGTCTTGGCTCTGTAAGC
>SRR4252624_8257452___Sbg4
TTCGTGTTGTCTCGGAGGTGGCCCTGGC
>SRR4252610_4704408___Sbg4
GAGTCATGTAAATAGTACCTACTAGTGGG
>SRR4252611_10111619___Sbg4
AGTCATGTAAATAGTACCTACTAGTGGG
>SRR4252612_6538451___Sbg4
AGTCATGTAAATAGTACCTACTAGTGGGG
>SRR4252605_5982308___Sbg4
CATCTGGCCATCATAATCATCTTCATCA
>SRR4252613_2930377___Sbg4
TCCAGGGAGGCCCTCCATGTCCTTGTGTA
>SRR4252612_2787448___Sbg4
TCTATATGTTCGTGTTGTCTCGGAGGTG
>SRR4252612_7932308___Sbg4
ATGTCAGGAAACTTTAGGCCCAAACGGT
>SRR4252605_12936840___Sbg4
TGTCTATATGTTCGTGTTGTCTCGGAGGT
>SRR4252611_8860357___Sbg4
GGGCGGCGTGAGTCATGTAAATAGTACCTA
>SRR4252608_5114164___Sbg4
TTTGTCTATATGTTCGTGTTGTCTCGGA
>SRR4252611_8619986___Sbg4
TCATGTAAATAGTACCTACTAGTGGG
>SRR4252617_11954707___Sbg4
CCATAGTCTATCCTCTCCCGACAGGTCTT
>SRR4252605_1540326___Sbg4
TGATCACTTATCCAGGGAGACCCTCCATA
>SRR4252622_6654498___Sbg4
TCTGTAAGGCCCGGGTGGCGTGAGTCAT
>SRR4252623_13817629___Sbg4
ACATCATCTGGCCATCATGATCATCTTCA
>SRR4252614_7419019___Sbg4
CTACTAGTGGGGGAGGTAAAACTTGGAA
>SRR4252610_11193602___Sbg4
AATCATCTGGCCATCATGATCATCTTCA

>SRR4252611_5859892___Sbg4
TCCAGGGAGGCCCTCCATGTCCTTGTGTA
>SRR4252618_3902809___Sbg4
TCGGGACAAGTTTCCTGGGTGAGGTTTTT
>SRR4252616_5411026___Sbg4
TTGTTGGTATGAGTGTGGGTGTGAAC
>SRR4252611_12382150___Sbg4
ACCTACTAGTGGGGGAGGTAAAACTTGGA
>SRR4252612_3199684___Sbg4
ACCTACTTGTGGGGGAGGTAAAACTTGGA
>SRR4252610_1075908___Sbg4
TCCTTGTGTAGTCAAAAAAGTCTGAATGT
>SRR4252626_6607861___Sbg4
AATGTCAGGAAACTTTAGGCCCAAACGGTC
>SRR4252611_3719924___Sbg4
ACAGGGAGGCCCTCCATGTCCTTGTGTA
>SRR4252624_6662702___Sbg4
GGTGTCTTTGTCTATATGTTCGTGTT
>SRR4252619_10409691___Sbg4
CATCTGGCCATCATAATCATCTTCATCA
>SRR4252620_2114884___Sbg4
TCCTTGTGTAGTCAAAAAAGTATGAATG
>SRR4252610_7628368___Sbg4
TGTCTATATGTTCGTGTTGTCTCGGAGGT
>SRR4252623_14759647___Sbg4
ATGTCAGGAAACTTTAGGCCCAAACGGTCCC
>SRR4252610_1325507___Sbg4
TGTGGGTGTGAATGGTGTCTTTGTCTATA
>SRR4252610_5125476___Sbg4
AGTCATGTAAATAGTACCTACTAGTGGG
>SRR4252607_14826196___Sbg4
CCTACTAGTGGGGGAGGTAAAACTTGGA
>SRR4252606_9175722___Sbg4
CCAGGGATGCCCTCCATGTCCTTGTGTA
>SRR4252616_180513___Sbg4
AGTCTATCCTCTCCCGACAGGTCTTGG
>SRR4252610_11493913___Sbg4
AGGGAGGCCCTCCATGTCCTTGTGTA
>SRR4252623_396835___Sbg4
TCTTTGTCTATATGTTCGTGTTGTCTCGGA
>SRR4252613_139791___Sbg4
CTACTAGTGGGGGAGGTAAAACTTGGAAA
>SRR4252609_9307152___Sbg4
CATCATCTGGCCATCATGATCATCTTCA
>SRR4252621_10956432___Sbg4
AGGCCCAAACGGTCCCCAGACATCAT
>SRR4252608_6367800___Sbg4
TTTGTCTATATGTTCGTGTCGTCTCGGAG
>SRR4252609_11839543___Sbg4
TTGTCTATATGTTCGTGTCGTCTCGGAGGTG
>SRR4252605_13201597___Sbg4
AGTCATGTAAATAGTACCTACTAGTGGGG
>SRR4252612_8841125___Sbg4
CATCTGGCCATCATAATCATCTTCATCA
>SRR4252613_4890337___Sbg4
CGGAGGTGGCCCTGGCATTGAGCTGATCA
>SRR4252611_6514147___Sbg4
AGTCATGTAAATAGTACCTACTAGTGGG
>SRR4252610_5525017___Sbg4
TATCTTCATCATATTCGCCTTGTTGGTA
>SRR4252619_896243___Sbg4
ATGTATATGTGATCCATAGTCTATCCTC
>SRR4252611_6329041___Sbg4
CCGGGTGGCGTGAGTCATGTAAATAGTA
>SRR4252622_2948123___Sbg4
CATCATCTGGCCATCATGATCATCTTCA
>SRR4252610_662899___Sbg4
CCTTGTCTATATGTTCGTGTTGTCTCGGA
>SRR4252610_8505187___Sbg4
CCTTGTCTATATGTTCGTGTTGTCTCGGA

>SRR4252606_3356501___Sbg4
AGTCATGTAAATAGTACCTACTAGTGGGG
>SRR4252611_10413620___Sbg4
AATGTCAGGAAACTTTAGGCCCAAACGGT
>SRR4252611_10935724___Sbg4
TCTTTGTCTATATGTTCGTGTTGTCTCGGA
>SRR4252625_11636750___Sbg4
TCAGACAAATGTCAGGAAACTTTAGGCCCA
>SRR4252610_497093___Sbg4
TCCATGTCCTTGTGTAGTCAAAAAAGTA
>SRR4252610_1244697___Sbg4
AGTCATGTAAATAGTACCTACTAGTGG
>SRR4252611_10593060___Sbg4
TCAGGGAGGCCCTCCATGTCCTTGTGTA
>SRR4252611_6137463___Sbg4
TCAGGGAGGCCCTCCATGTCCTTGTGTA
>SRR4252610_414717___Sbg4
TCTTGGCTCTGTAAGGCCCGGGTGGCGTT
>SRR4252612_3141566___Sbg4
TCCTTGTGTAGTCAAAAAAGTTTGAA
>SRR4252611_6506149___Sbg4
TAAGGCCCGGGTGGCGTGAGTCATGTAAA
>SRR4252608_1614285___Sbg4
AGGGAGGCCCTCCATGTCCTTGTGTA
>SRR4252610_2953084___Sbg4
AGGGAGGCCCTCCATGTCCTTGTGTA
>SRR4252607_4110117___Sbg4
TCCTTGTGTAGTCAAAAAAGTATGAATG
>SRR4252605_12954927___Sbg4
TGTGTAGTCAAAAAAGTATGAATGTATGT
>SRR4252606_9623836___Sbg4
CCCGACAGGTCTTGGCTCTGAAAGGC
>SRR4252614_3443615___Sbg4
TTTGTCTATATGTTCGTGTTGTCTCGGAC
>SRR4252610_10354066___Sbg4
AGTCATGTAAATAGTACCTACTAGTGGGG
>SRR4252610_3795434___Sbg4
AAGACAAATGCCAGGAAACTTTAGGCCCAA
>SRR4252606_8951411___Sbg4
ATATGTTCGTGTTGTCTCGGAGGTGG
>SRR4252613_5911016___Sbg4
GGAGGCCCTCCATGTCCTTGTGTAGTCA
>SRR4252615_8846231___Sbg4
TATCCTCTCCCGACAGGTCTTGGCTCA
>SRR4252621_8663495___Sbg4
TCATCTGGCCATCATAATCATCTTCATCA
>SRR4252611_9737101___Sbg4
GAGGCCCCCCATGTCCTTGTGTAGTCA
>SRR4252609_4445066___Sbg4
TTGTCTCGGAGGTGGCCCTGGTATTGAGC
>SRR4252606_9823926___Sbg4
TTGTCTCGGAGGTGGCCCTGGCATTGAGC
>SRR4252616_2366891___Sbg4
GGAGGCCCTCCATGTCCTTGTGTAGTCA
>SRR4252614_8932869___Sbg4
GTCTGGCCATCATAATCATCTTCATCATA
>SRR4252610_11945059___Sbg4
CCGGGAGGCGTGAGTCATGTAAATAGTA
>SRR4252610_3550181___Sbg4
AGTCATGTAAATAGTACCTACTAGTGGGG
>SRR4252610_7631450___Sbg4
TTTCTATATGTTCGTGTTGTCTCGGAG
>SRR4252608_5318979___Sbg4
TCTTTGTCTATATGTTCGTGTTGTCTCGG
>SRR4252611_522874___Sbg4
ACTACTAGTGGGGGAGGTAAAACTTGGAA
>SRR4252610_2634901___Sbg4
CTTTGTCTATATGTTCGTGTTGTCTCGGA
>SRR4252605_14624122___Sbg4
TTGTCTCGGAGGTGGCCCTGGCATTGAGT

>SRR4252606_961197___Sbg4
ATCATAATCATCTTCATCATATTCGCCT
>SRR4252608_10669594___Sbg4
TGTCTATATGTTCGTGTTGTCTCGGAG
>SRR4252611_1309230___Sbg4
GGTGGCGTGAGTCATGTAAATAGTACCTA
>SRR4252608_3913248___Sbg4
AATCTGGCCATCATAATCATCTTCATCA
>SRR4252605_13898548___Sbg4
TCCGTGTCCTTGTGTAGTCAAAAAAGTATGA
>SRR4252605_11321674___Sbg4
TCCTTGTGTAGTCAAAAAAGTATGAATGT
>SRR4252612_4584828___Sbg4
TCCAGGGAGACCCTCCATGTCCTTGTGTA
>SRR4252609_4603023___Sbg4
TCCTTGTGTAGTCAAAAAAGTATGAATG
>SRR4252606_4668659___Sbg4
TTTAGGCCCAAACGGTCCCCAGACATCA
>SRR4252606_1368705___Sbg4
CCTACTAGTGGGGGAGGTAAAACTTGGAA
>SRR4252624_1094992___Sbg4
GTGTGGGTGTGAATGGTGTCTTTGTCTATA
>SRR4252609_15478195___Sbg4
TTTGTCTATATGTTCGTGTTGTCTCGGAG
>SRR4252605_7076285___Sbg4
AGTCATGTAAATAGTACCTACTAGTGGGG
>SRR4252622_11220108___Sbg4
ACATCATCTGGCCATCATGATCATCTTCA
>SRR4252608_6504197___Sbg4
TTTGTCTATATGTTCGTGTTGTCTCGGAGGT
>SRR4252607_4916213___Sbg4
TCCTTGTGTAGTCAAAAAGGTATGAATGT
>SRR4252610_3591149___Sbg4
CTTTGTCTACATGTTCGTGTTGTCTCGGA
>SRR4252610_11878771___Sbg4
ATAGTCTATCCTCTCCCGACAGGTCTTG
>SRR4252605_12120553___Sbg4
GGGGAGGCCCTCCATGTCCTTGTGTA
>SRR4252614_5798063___Sbg4
GGGGAGGCCCTCCATGTCCTTGTGTA
>SRR4252609_10854509___Sbg4
ATGTGATCCATGGTCTATCCTCTCCCGACA
>SRR4252616_2190743___Sbg4
GGTCTATCCTCTCCCGACAGGTCTTGGC
>SRR4252611_5594366___Sbg4
TGTTGGTATGAGTGTGGGTGTTAATGGTGTC
>SRR4252611_7985830___Sbg4
TTGTCTATGTGTTCGTGTTGTCTCGGA
>SRR4252610_7932552___Sbg4
TGTGGGTGTGAATGGTGTCTTTGTCTATA
>SRR4252606_8584113___Sbg4
CATCCATGTCCTTGTGTAGTCAAAAAAG
>SRR4252605_3587349___Sbg4
GTGTCTTTGTCTATATGTTCGTGTTGTC
>SRR4252612_3442246___Sbg4
AGGCTCTCCATGTCCTTGTGTAGTCAAA
>SRR4252612_5169647___Sbg4
AATCTGGCCATCATGATCATCTTCATCA
>SRR4252617_16245903___Sbg4
TGTCTATATGTTCGTGTTGTCTCGGAGGT
>SRR4252610_1749528___Sbg4
ACAAATGTCAGGAAACTTTAGGCCCAAAC
>SRR4252620_5745152___Sbg4
CCAGGGAGGCCCTCCATGTCCTTGTGTA
>SRR4252607_7221049___Sbg4
TGTAGTCAAAAAAGTATGAATGTATATGTG
>SRR4252612_3951420___Sbg4
AGTCATGTAAATAGTACCTACTAGTGGG
>SRR4252607_2416127___Sbg4
TTGTCTCGGAGGTGGCCCTGGCATTGAGA

>SRR4252607_10718844___Sbg4
TTTGTCTATATGTTCGTGTTGTCTCGG
>SRR4252626_6464597___Sbg4
TGTTCGTGTTGTCTCGGAGGTGGCCCTGGC
>SRR4252625_14033908___Sbg4
CATCTGGCCGTCATGATCATCTTCATCA
>SRR4252608_939270___Sbg4
GGAGGTGGCCCTGGCATTGAGCTGAT
>SRR4252611_14486281___Sbg4
AATGTCAGGAAACTTTAGGCCCAAAC
>SRR4252611_11878170___Sbg4
TCATCTGGCCATCATAATCATCTTCAT
>SRR4252607_4357213___Sbg4
ATCTGGCCATCATAATCATCTTCATCATA
>SRR4252608_11868148___Sbg4
GCCCGGGTGGCGTGAGTCATGTAAATAGT
>SRR4252612_6404191___Sbg4
TGTCCTTGTGTAGTCAAAAAAGTATGA
>SRR4252609_3992175___Sbg4
ATTTGTCTATATGTTCGTGTTGTCTCGGA
>SRR4252615_4094221___Sbg4
ATATGTGATCCATAGTCTATCCTCTC
>SRR4252625_2526604___Sbg4
TGTGTAGTCAAAAAAGTATGAATGTATATGT
>SRR4252610_2502516___Sbg4
TCCTTGTGTAGTCAAAAAAGTATGAATGT
>SRR4252612_8976067___Sbg4
TCATCTGGCCATCATGATCATCTTCATCA
>SRR4252616_54110___Sbg4
GGGAGGCCCTCCATGTCCTTGTGTAGTCAAA
>SRR4252607_9397808___Sbg4
GAGACAAATGTCAGGAAACTTTAGGCCCAA
>SRR4252611_9108915___Sbg4
TTGTCTCGGAGGTGGCCCTGGCATTGAGC
>SRR4252607_4566803___Sbg4
TCCTTGTGTAGTCAAAAAAGTATGAATG
>SRR4252612_7655742___Sbg4
TTGTCTATATGTTCGTGTTGTCTCGGA
>SRR4252621_7426149___Sbg4
GCCCTCCATGTCCTTGTGTAGTCAAAAAA
>SRR4252610_4292968___Sbg4
CCTACTAGTGGGGGAGGTAAAACTTG
>SRR4252611_13638440___Sbg4
AATGTCAGGAAACTTTAGGCCCAAACGGT
>SRR4252605_13355461___Sbg4
TGTCATGTAAATAGTACCTACTAGTGGGG
>SRR4252605_13728489___Sbg4
TGTCATGTAAATAGTACCTACTAGTGGGGGA
>SRR4252608_1363814___Sbg4
TGTCATGTAAATAGTACCTACTAGTGGGG
>SRR4252612_2622698___Sbg4
TGTCATGTAAATAGTACCTACTAGTGGGG
>SRR4252612_8993033___Sbg4
TGTCATGTAAATAGTACCTACTAGTGGG
>SRR4252606_9781962___Sbg4
GGAGGCCCTCCATGTCCTTGTGTAGTCA
>SRR4252605_4183379___Sbg4
TCCTTGTGTAGTCAAAAAAGTATGAATGT
>SRR4252613_10700954___Sbg4
TCCTTGTGTAGTCAAAAAAGTATGAATG
>SRR4252606_4800267___Sbg4
AAGACAAATGTCAGGAAACTTTAGGCCCAA
>SRR4252605_12930585___Sbg4
CCAGGGAGGCCCTCCATGTCCTTGTGTA
>SRR4252605_6180040___Sbg4
CATAGTCTATCCTCTCCCGACAGGTCTTGGC
>SRR4252608_3818434___Sbg4
TTATGTGATCCATAGTCTATCCTCTCCCG
>SRR4252612_2494256___Sbg4
TCATCTGGCCATCATGATCATCTTCATC

>SRR4252607_1483647___Sbg4
AGTCATGTAAATAGTACCTACTAGTGGGG
>SRR4252612_4511701___Sbg4
GTGTCCTTGTGTAGTCAAAAAAGTATGAA
>SRR4252606_3660578___Sbg4
GGTGGCGTGAGTCATGTAAATAGTACCTA
>SRR4252611_14523288___Sbg4
ATCATCCGGCCATCATAATCATCTTCA
>SRR4252610_7104690___Sbg4
TGTCTATATGTTCGTGTTGTCTCGGAGGT
>SRR4252606_4728302___Sbg4
TTGTCTCGGAGGTGGCCCTGGCATTGAGT
>SRR4252621_416174___Sbg4
TCCAGGGATGCCCTCCATGTCCTTGTGTA
>SRR4252621_13959862___Sbg4
GCCCTCCATGTCCTTGTGTAGTCAAAAAA
>SRR4252605_4960641___Sbg4
TGTGTAGTCAAAAAAGTATGAATGTATATG
>SRR4252605_2737317___Sbg4
AAAGACAAATGTCAGGAAACTTTAGGCCCA
>SRR4252610_4829582___Sbg4
AAAGACAAATGTCAGGAAACTTTAGGCCCA
>SRR4252605_13686375___Sbg4
TTGTCTCGGAGGTGGCCCTGGCATTGAGC
>SRR4252612_6091443___Sbg4
TCTTTGTCTATATGTTCGTGTTGTCTCGGA
>SRR4252605_6402993___Sbg4
TCGAGGGAGGCCCTCCATGTCCTTGTGTA
>SRR4252605_7541126___Sbg4
TCGAGGGAGGCCCTCCATGTCCTTGTGTA
>SRR4252610_12735718___Sbg4
TCGAGGGAGGCCCTCCATGTCCTTGTGTA
>SRR4252610_7040847___Sbg4
GAGGGAGGCCCTCCATGTCCTTGTGTA
>SRR4252610_12479869___Sbg4
CCAGGGAGGCCCTCCATGTCCTTGTGTA
>SRR4252614_6994204___Sbg4
CCTACTAGTGGGGGAGGTAAAACTTGGA
>SRR4252607_11115033___Sbg4
TTGTCTCGGAGGTGGCCCTGGCATTGAGC
>SRR4252611_2044168___Sbg4
TTGTCTCGGAGGTGGCCCTGGCATTGAGC
>SRR4252625_2210349___Sbg4
ACCTACTAGTGGGGGAGGTAAAACTTGGA
>SRR4252610_12247906___Sbg4
TTGGCTCTGTAAGGCCCGGGTGGCGT
>SRR4252605_14947246___Sbg4
TCTATCCTCTCCCGACAGGTCTTGGCTCT
>SRR4252610_871760___Sbg4
TGTGGGTGTGAATGGTGTCTTTGTCTA
>SRR4252608_2055268___Sbg4
CATCTTCATCATATTCGCCTTGTTTA
>SRR4252606_1838559___Sbg4
TGTCTTTGTCTATATGTTCGTGTTGTC
>SRR4252609_4656712___Sbg4
CCTTGTGTAGCCAAAAAAGTATGAATGTA
>SRR4252612_5065908___Sbg4
AGCCAGGGAGGCCCTCCATGTCCTTGTGTA
>SRR4252605_9326630___Sbg4
GCCAGGGAGGCCCTCCATGTCCTTGTGTA
>SRR4252610_935846___Sbg4
GCCAGGGAGGCCCTCCATGTCCTTGTGTA
>SRR4252610_1158564___Sbg4
AGTCATGTAAATAGTACCTACTAGTGGGG
>SRR4252611_13189235___Sbg4
TCCAGGGAGGCCCTCCATGTCCTTGTGTA
>SRR4252609_13897112___Sbg4
TCTTTGTCTATATGTTCGTGTTGTCTCGG
>SRR4252608_33499___Sbg4
ATCATCTTCATCATATTCGCCTTGTTT

>SRR4252616_7755975___Sbg4
TGTAGTCAAAAAAGTATGAATGTACA
>SRR4252607_5937596___Sbg4
TTGTCTCGGAGGTGGCCCTGGCATTGAGC
>SRR4252606_2975405___Sbg4
TAGACATCATCTGGCCATCATGATCA
>SRR4252611_1454440___Sbg4
TCCTTGTGTAGTCAAAAAAGTATGAAC
>SRR4252614_2546808___Sbg4
TCCTTGTGTAGTCAAAAAAGTATGAAC
>SRR4252607_7557633___Sbg4
TCCTTGTGTAGTCAAAAAAGTATGAATGT
>SRR4252607_9860195___Sbg4
TCCTTGTGTAGTCAAAAAAGTATGAAT
>SRR4252610_3010559___Sbg4
ATCATCTTCATCATATTCGCCTTGTTGA
>SRR4252611_1622226___Sbg4
GAAGGCCCTCCATGTCCTTGTGTAGTCA
>SRR4252613_2555848___Sbg4
TGTCAGGAAACTTTAGGCCCGAACGGTCCC
>SRR4252606_3056212___Sbg4
TGGGTGTGAATGGTGTCTTTGTCTATATGG
>SRR4252608_1605344___Sbg4
CCTCCATGTCCTTGTGTAGTCAAAAA
>SRR4252616_10507791___Sbg4
ACCCTCCATGTCCTTGTGTAGTCAAAAAAG
>SRR4252611_2985264___Sbg4
AATGGTGTCTTTGTCTATATGTTCGTG
>SRR4252605_14508660___Sbg4
TTGTCTCGGAGGTGGCCCTGGCATTGAGC
>SRR4252607_7942686___Sbg4
ACATCATCTGGCCATCATGATCATCTTCA
>SRR4252621_4628362___Sbg4
ACATCATCTGGCCATCATGATCATCTTCA
>SRR4252608_3069371___Sbg4
CATCTGGCCATCATGATCATCTTCATCA
>SRR4252609_2658956___Sbg4
TCCAGGGAGGCCCTCCATGTCCTTGTGTA
>SRR4252609_5235369___Sbg4
TCCAGGGAGGCCCTCCATGTCCTTGTGTA
>SRR4252608_1208018___Sbg4
AGTCATGCAAATAGTACCTACTAGTGGGG
>SRR4252605_11206028___Sbg4
CTTGTTGGTATGAGTGTGGGTGTGAATGGTG
>SRR4252610_4963921___Sbg4
TGTGGGTGTGAATGGTGTCTTTGTCTATA
>SRR4252610_8656359___Sbg4
AGTCATGTAAATAGTACCTACTAGTGGG
>SRR4252609_2381668___Sbg4
ATCATCTTCATCATATTCGCCTTGTT
>SRR4252611_672088___Sbg4
TCCATGTCCTTGTGTAGTCAAAAAAGTA
>SRR4252612_7947823___Sbg4
GATCTGGCCATCATAATCATCTTCATCATA
>SRR4252610_11590430___Sbg4
AGTCATGTAAATAGTACCTACTAGTGGGG
>SRR4252616_9198972___Sbg4
AGCTCTGTAAGGCCCGGGTGGCGTGAGT
>SRR4252619_11387718___Sbg4
AATCATCTTCATCATATTCGCCTTGTT
>SRR4252612_5706228___Sbg4
ATCATCTTCATCATATTCGCCTTGTT
>SRR4252607_3197495___Sbg4
TTGTCTCGGAGGTGGCCCTGGCATTGAGC
>SRR4252609_281323___Sbg4
TTGTCTCGGAGGTGGCCCTGGCATTGAGC
>SRR4252607_1887881___Sbg4
TAAGACAAATGTCAGGAAACTTTAGGT
>SRR4252607_3673745___Sbg4
CATCTGGCCATCATAATCATCTTCATCA

```
>SRR4252621_12916573___Sbg4
AAGAGGCCCTCCATGTCCTTGTGTAGTCA
>SRR4252614_6658868___Sbg4
AGGGAGGCCCTCCATGTCCTTGTGTA
>SRR4252624_9697625___Sbg4
GCCTACTAGTGGGGGAGGTAAAACTTGGA
>SRR4252609_2729074___Sbg4
TAATTGTGTAGTCAAAAAAGTATGAATGTA
>SRR4252615_4024362___Sbg4
AATGTCAGGAAACTTTAGGCCCAAACGGTCT
>SRR4252610_10851194___Sbg4
ACCTGGCCATCATAATCATCTTCATCATA
>SRR4252607_4024854___Sbg4
GTTGTCTCGGAGGTGGCCCTGGCATTGAGT
>SRR4252609_16026347___Sbg4
GGATGGCGTGAGTCATGTAAATAGTACCTA
>SRR4252610_5083113___Sbg4
TTTGTCTATTTGTTCGTGTTGTCTCGGA
>SRR4252611_8307797___Sbg4
TCCTTGTGTAGTCAAAAAAGTATGAATG
>SRR4252610_268195___Sbg4
GAGTCATTTAAATAGTACCTACTAGTGGGG
>SRR4252608_9311959___Sbg4
GGGCCCAAACGGTCCCCAGACATCATC
>SRR4252615_12505416___Sbg4
GGGCCCAAACGGTCCCCAGACATCAT
>SRR4252616_13536210___Sbg4
TAAGACAAATGTCAGGAAACTTTAGGCCT
>SRR4252620_9750010___Sbg4
GTCCAGGGAGGCCCTCCATGTCCTTGTGTA
>SRR4252610_12389016___Sbg4
GCATCATCTGGCCATCATGATCATCTTCA
>SRR4252611_5533388___Sbg4
GTGTAGTCAAAAAAGTATGAATGTATATG
>SRR4252608_5409834___Sbg4
GTCTTTGTCTATATGTTCGTGTTGTCTCG
>SRR4252613_10814922___Sbg4
CTCGGAGGTGGCCCTGGCATTGAGTT
>SRR4252605_8963971___Sbg4
GTGTAGTCAAAAAAGTATGAATGTATA
>SRR4252606_1561398___Sbg4
CATCATCTGGCCATCATGATCATCTTCA
>SRR4252605_14834698___Sbg4
CATCTGGCCATCATGATCATCTTCATCA
>SRR4252610_10443440___Sbg4
TACTAGTGGGGGAGGTAAAACTTGGAA
>SRR4252605_6826528___Sbg4
CCTTGTCTATATGTTCGTGTTGTCTCGGA
>SRR4252610_6948584___Sbg4
CATCTGGCCATCATAATCATCTTCATCA
>SRR4252621_5487153___Sbg4
CTATATGTTCGTGTTGTCTCGGAGGTG
>SRR4252611_15426109___Sbg4
GGGCCCGGGTGGCGTGAGTCATGTAAATA
>SRR4252608_2820993___Sbg4
AGTCATGTAAATAGTACCTACTAGTGGG
>SRR4252606_9397236___Sbg4
CTATATGTTCGTGTTGTCTCGGAGGTGGC
>SRR4252606_7945992___Sbg4
TGGGTGTGAATGGTGTCTTTGTCTATATG
>SRR4252609_2655922___Sbg4
ACATCATCTGGCCATCATGATCATCTTCA
>SRR4252610_1286459___Sbg4
TGGAGGCTCTCCATGTCCTTGTGTAGTCA
>SRR4252624_9162158___Sbg4
TCCTTGTGTAGTCAAAAAAGTATGAATGT
>SRR4252606_2874577___Sbg4
CTTGTTGGTATGAGTGTGGGTGTGAATGGTT
>SRR4252611_10891319___Sbg4
AGGGAGGCCCTCCATGTCCTTGTGTA
```

```
>SRR4252607_14316851___Sbg4
CTTTGTCTATATGTTCGTGTTGTCTCGGA
>SRR4252608_842167___Sbg4
CATCTGGCCATCATAATCATCTTCATCA
>SRR4252622_2080038___Sbg4
TCGAGGGAGGCCCTCCATGTCCTTGTGTA
>SRR4252612_4952966___Sbg4
TGTCAGGAAACTTTAGGCCCAAACGGTCCC
>SRR4252615_7447234___Sbg4
TCTATATGTTCGTGTTGTCTCGGAGGTG
>SRR4252605_1121159___Sbg4
ATCTGGCCATCATAATCATCTTCATCA
>SRR4252611_8431596___Sbg4
CTTTGTCTATATGTTCGTGTTGTCTCGGA
>SRR4252612_382415___Sbg4
CTTTGTCTATATGTTCGTGTTGTCTCGGA
>SRR4252613_9438900___Sbg4
TCCTTGTGTAGTCAAAAAAGTATGAAT
>SRR4252624_4282048___Sbg4
ATTCTCTCCCGACAGGTCTTGGCTCTGTAA
>SRR4252611_8382477___Sbg4
AGTCATGTAGATAGTACCTACTAGTGGG
>SRR4252612_5061574___Sbg4
TCCTTGTGTAGTCAAAAAAGTATGAAT
>SRR4252606_8264379___Sbg4
TGTAGTCAAAAAAGTATGAATGTATGTGC
>SRR4252621_5046775___Sbg4
TATCCTCTCCCGACAGGTCTTGGCTCTGTA
>SRR4252612_1294487___Sbg4
CCATAAGACAAATGTCAGGAAACTTTAGG
>SRR4252615_3438632___Sbg4
TTTGTCTATATGTTCGTGTTGTCTTGGA
>SRR4252608_8267792___Sbg4
TCCAGGGAAGCCCTCCATGTCCTTGTGTA
>SRR4252610_10827152___Sbg4
GTGTGGGTGTGAATGGTGTCTTTGTCTATA
>SRR4252610_2255452___Sbg4
GCATCTGGCCATCATAATCATCTTCAT
>SRR4252605_6329659___Sbg4
CCATAGTCTATCCTCTCCCGACAGGTCT
>SRR4252614_8622223___Sbg4
TGTAAGGCCGGGGTGGCGTGAGTCATGTA
>SRR4252616_7136102___Sbg4
AAGGCCGGGGTGGCGTGAGTCATGTAAATA
>SRR4252606_8325402___Sbg4
TTCGTCTATATGTTCGTGTTGTCTCGGA
>SRR4252607_14227343___Sbg4
AGTGGCGTGAGTCATGTAAATAGTACCTA
>SRR4252612_6346900___Sbg4
AGTCATGTAAATAGTACCTACTAGTGGG
>SRR4252612_2129372___Sbg4
TCCTTGTGTAGTCAAAAAAGTATGAATGA
>SRR4252612_2944822___Sbg4
TCCTTGTGTAGTCAAAAAAGTATGAATGA
>SRR4252623_8449112___Sbg4
TCCTTGTGTAGTCAAAAAAGTATGAATGA
>SRR4252610_9443819___Sbg4
CCATGTCCTTGTGTAGTCAAAAAAGTA
>SRR4252605_4133507___Sbg4
ATCTAGGGAGGCCCTCCATGTCCTTGTGTA
>SRR4252608_7919665___Sbg4
CTTCGTCTATATGTTCGTGTTGTCTCGGA
>SRR4252611_6129880___Sbg4
TGTGTAGTCAAAAAAGTATGAATGTATA
>SRR4252614_3350394___Sbg4
GATCCATAGTCTATCCTCTCCCGACAGG
>SRR4252605_3554012___Sbg4
AGCATGAGTCATGTAAATAGTACCTACTA
>SRR4252611_11508405___Sbg4
GCATCATCTGGCCATCATGATCATCTTCA
```

>SRR4252610_7317463___Sbg4
AGTCATGTAAATAGTACCTACTAGTGGG
>SRR4252622_715623___Sbg4
TGTCTATATGTTCGTGTTGACTCGGAGG
>SRR4252606_677446___Sbg4
TTTTTCCACACCCATAAGACAAATGTCA
>SRR4252620_8333291___Sbg4
TACTTATCCAGGGAGGCCCTCCATGTCCT
>SRR4252605_5481061___Sbg4
CCATGTCCTTGTGTAGTCAAAAAAGTA
>SRR4252611_9681949___Sbg4
GGGTGGCGTGAGTCATGTAAATAGTACCTA
>SRR4252605_14093417___Sbg4
AGTCATGTAAATAGTACCTACTAGTGGG
>SRR4252608_9233216___Sbg4
AGTCATGTAAATAGTACCTACTAGTGGG
>SRR4252605_13412045___Sbg4
TGTCATGTAAATAGTACCTACTAGTGGGG
>SRR4252606_7393148___Sbg4
TGTCATGTAAATAGTACCTACTAGTGGGG
>SRR4252607_11563462___Sbg4
TGTCATGTAAATAGTACCTACTAGTGGGG
>SRR4252608_1565950___Sbg4
TGTCATGTAAATAGTACCTACTAGTGGGG
>SRR4252610_12468594___Sbg4
TGTCATGTAAATAGTACCTACTAGTGGG
>SRR4252610_2362401___Sbg4
TGTCATGTAAATAGTACCTACTAGTGGG
>SRR4252610_6486120___Sbg4
TGTCATGTAAATAGTACCTACTAGTGGGG
>SRR4252605_12673358___Sbg4
GTGTGGGTGTGAATGGTGTCTTTGTCT
>SRR4252605_12323492___Sbg4
CCAGGGAGGCCCTCCATGTCCTTGTGT
>SRR4252614_721250___Sbg4
AGGGAGGCCCTCCATGTCCTTGTGTA
>SRR4252610_11856800___Sbg4
CCTACTAGTGGGGGAGATAAAACTTGGA
>SRR4252616_3008054___Sbg4
ACCAGGGAGGTCCTCCATGTCCTTGTGTA
>SRR4252612_631302___Sbg4
AGGGAGGTCCTCCATGTCCTTGTGTA
>SRR4252619_6016024___Sbg4
AGGTCCTCCATGTCCTTGTGTAGTCAAAA
>SRR4252610_2535101___Sbg4
ATCATCCGGCCATCATAATCATCTTCA
>SRR4252610_9984581___Sbg4
TCATCCGGCCATCATAATCATCTTCA
>SRR4252611_6865689___Sbg4
CCAGGGAGTCCCTCCATGTCCTTGTGTA
>SRR4252626_75324___Sbg4
TCCCTCCATGTCCTTGTGTAGTCAAA
>SRR4252622_11035398___Sbg4
TCTACTAGTGGGGGAGGTAAAACTTGGA
>SRR4252614_5380746___Sbg4
TTTGTCTATATGTTCGTGTTGTCTCGGAG
>SRR4252608_7469035___Sbg4
TGTCTATATGTTCGTGTTGTCTCGGAG
>SRR4252611_530375___Sbg4
TATGTTCGTGTTGTCTCGGAGGTGGCCCTTT
>SRR4252614_5504542___Sbg4
TCCAGGGAGGCCCTCCATGTCCTTGTGTA
>SRR4252612_7603423___Sbg4
GTTGTCTCGGAGGTGGCCCTGGCATTGAGC
>SRR4252607_7756238___Sbg4
TTGTCTCGGAGGTGGCCCTGGCATTGAGC
>SRR4252612_8830651___Sbg4
TCAGGGAGGCCCTCCATGTCCTTGTGTA
>SRR4252605_14223303___Sbg4
AGGCCCTCCATGTCCTTGTGTAGTCAAAAA

```
>SRR4252610_6368926___Sbg4
CATGTCCTTGTGTAGTCAAAAAAGTATGAAT
>SRR4252610_2363665___Sbg4
AGTCATGTCAATAGTACCTACTAGTGGGG
>SRR4252605_9922768___Sbg4
TCATCTGGCCATCATAATCATCTTCA
>SRR4252608_3868466___Sbg4
TCATCTGGCCATCATAATCATCTTCATCA
>SRR4252609_2385872___Sbg4
GTCTCGGAGGTGGCCCTGGCATTGAGC
>SRR4252621_11722034___Sbg4
GGTCCTCCATGTCCTTGTGTAGTCAAAAA
>SRR4252611_12589092___Sbg4
TCCAGGGAGGCCCTCCATGTCCTTGTGTA
>SRR4252619_14548740___Sbg4
CCAGGGAGGCCCTCCATGTCCTTGTGTA
>SRR4252610_2766333___Sbg4
TAGGGAGGCCCTCCATGTCCTTGTGTA
>SRR4252610_9068399___Sbg4
TAGGGAGGCCCTCCATGTCCTTGTGTA
>SRR4252605_2451783___Sbg4
AATGTCAGGAAACTTTAGGCCCAAACGGTCA
>SRR4252612_7907055___Sbg4
TCTCTCCCGACAGGTCTTGGCTCTGTA
>SRR4252622_9739392___Sbg4
TAAGGCCCGGGTGGCGTGAGACATGTA
>SRR4252610_5514844___Sbg4
AGACATGTAAATAGTACCTACTAGTGGG
>SRR4252607_5499276___Sbg4
TCCTTGTGTAGTCAAAAATGTATGAATG
>SRR4252610_1970837___Sbg4
TTTGTCTATGTGTTCGTGTTGTCTCGGA
>SRR4252605_3289376___Sbg4
CCGGGTAGCGTGAGTCATGTAAATAGTA
>SRR4252605_5449886___Sbg4
ACATCATCTGGCCATCATGATCATCTTCA
>SRR4252606_6187525___Sbg4
GTGTGGGTGTGAATGGTGTCTTTGTCTATA
>SRR4252612_5062452___Sbg4
GGGGAGGCCCTCCATGTCCTTGTGTA
>SRR4252610_9717946___Sbg4
TTGTCTCGGAGGTGGCCCTGGCATTGAGT
>SRR4252610_10465295___Sbg4
TGTCTCGGAGGTGGCCCTGGCATTGAGT
>SRR4252609_4296489___Sbg4
ACATGTCCTTGTGTAGTCAAAAAAGTA
>SRR4252607_12748632___Sbg4
TGTTTCCTCACCCATAAGACAAATGTCA
>SRR4252612_8569758___Sbg4
AGGGAGGCCCTCCATGTCCTTGTGTAGTCA
>SRR4252605_7288017___Sbg4
AGTCATGTAAATAGTACCTACTAGTGGGG
>SRR4252622_1683544___Sbg4
TGTAGTCAAAAAAGTATGAATGTATATGT
>SRR4252610_13216988___Sbg4
AAGTCATGTAAATAGTACCTACTAGTGGG
>SRR4252611_2862862___Sbg4
TATCTGGCCATCATGATCATCTTCATCA
>SRR4252616_11266083___Sbg4
TATCTGGCCATCATGATCATCTTCATCA
>SRR4252613_11506384___Sbg4
TCTACTAGTGGGGGAGGTAAAACTTGGA
>SRR4252616_12594424___Sbg4
TTGTCTCGGAGGTGGCCCTGGCATTGAGC
>SRR4252610_8533213___Sbg4
AGTCATGTAAATAGTACCTACTAGTGGG
>SRR4252611_4270790___Sbg4
TTTGTCTATATGTTCGTGTTGTCTCGG
>SRR4252624_4967182___Sbg4
GTTGTCTCGGAGGTGGCCCTGGCATTGAGT
```

>SRR4252611_10515614___Sbg4
TTGTCTCGGAGGTGGCCCTGGCATTGAGT
>SRR4252612_2440984___Sbg4
CATCATCTGGCCATCATGATCATCTTCA
>SRR4252610_2499350___Sbg4
ACATCATCTAGCCATCATGATCATCTTCA
>SRR4252610_9329698___Sbg4
ACATCATCTAGCCATCATGATCATCTTCA
>SRR4252608_12325273___Sbg4
CATCATCTAGCCATCATGATCATCTTCA
>SRR4252611_9387929___Sbg4
CATCATCTAGCCATCATGATCATCTTCA
>SRR4252623_2244030___Sbg4
TCCAGGGAGGCCCTCCATGTCCTTGTGTA
>SRR4252613_10916960___Sbg4
CATCTGGCCATCATAATCATCTTCATCA
>SRR4252605_14984469___Sbg4
AGTCATGTAAATAGTACCTACTAGTG
>SRR4252608_9859536___Sbg4
CATCTGGCCATCATAATCATCTTCATCA
>SRR4252608_6277263___Sbg4
CTTTGTCTATATGTTCGTGTTGTCTCGGA
>SRR4252606_9656981___Sbg4
CATCTTCTGGCCATCATGATCATCTTCA
>SRR4252610_137287___Sbg4
CATCTTCTGGCCATCATGATCATCTTCA
>SRR4252610_9786917___Sbg4
GGTGTCTTTGTCTATATGTTCGTGTTGTC
>SRR4252610_8070949___Sbg4
AAAAGACAAATGTCAGGAAACTTTAGGCCCA
>SRR4252610_12836658___Sbg4
TCTTGTCTATATGTTCGTGTTGTCTCGGA
>SRR4252610_3011727___Sbg4
TCTTGTCTATATGTTCGTGTTGTCTCGGA
>SRR4252611_7711659___Sbg4
TCTTGTCTATATGTTCGTGTTGTCTCGGA
>SRR4252611_2355796___Sbg4
TCTACTAGTGGGGGAGGTAAAACTTGG
>SRR4252611_8487719___Sbg4
TTTGTCTATATGTTCGTGTTGTCTCA
>SRR4252607_5881669___Sbg4
TCCTTGTGTAGTCAAAAAAGTATGAATG
>SRR4252610_2346853___Sbg4
TCAGGGAGGCCCTCCATGTCCTTGTGTA
>SRR4252612_6727507___Sbg4
TTTGTCTATATGTTCGTGTTGTCTCGGAGGT
>SRR4252610_11087642___Sbg4
AGTCATGTAAATAGTACCTACTAGTGGGG
>SRR4252609_11861902___Sbg4
AGTGGCGTGAGTCATGTAAATAGTACCTA
>SRR4252611_7050___Sbg4
GGAAACTTTAGTCCCAAACGGTCCCCAGACA
>SRR4252614_7462496___Sbg4
TCCTTGTGTAGTCAAAAAAGTATGAATG
>SRR4252610_12622538___Sbg4
ACCTACTAGTAGGGGAGGTAAAACTTGGA
>SRR4252606_9085027___Sbg4
CTTCGTCTATATGTTCGTGTTGTCTCGGA
>SRR4252610_12769637___Sbg4
CTTCGTCTATATGTTCGTGTTGTCTCGGA
>SRR4252620_3201567___Sbg4
TCCTTGTGTAGTCAAAAAAGTATGAATG
>SRR4252623_10080396___Sbg4
TTTCTCTATATGTTCGTGTTGTCTCGGA
>SRR4252610_3351797___Sbg4
ACATCATCTAGCCATCATGATCATCTTCA
>SRR4252611_11363810___Sbg4
CATCATCTAGCCATCATGATCATCTTCA
>SRR4252606_90566___Sbg4
ACCTACTAGTGGGGGAGGTAAAACTTGGA

>SRR4252618_7231114___Sbg4
ACTTTAGACCCAAACGGTCCCCAGACATCA
>SRR4252619_14582248___Sbg4
CCCGGGTGGCGTGAGTCATGTAAATAG
>SRR4252606_8517815___Sbg4
GGGTGGCGTGAGTCATGTAAATAGTACCTA
>SRR4252610_8277997___Sbg4
AAGGCCCGGGTGGCGTGAGTCATGTAAAT
>SRR4252610_4003862___Sbg4
AGTCATGTAAATAGTACCTACTAGTGGG
>SRR4252611_3013719___Sbg4
AATGTCAGGAAACTTTAGGCCCAAACGGTCA
>SRR4252607_14580309___Sbg4
TATCTGGCCATCATAATCATCTTCATCATA
>SRR4252612_5428096___Sbg4
TATCTGGCCATCATAATCATCTTCATCA
>SRR4252610_9750189___Sbg4
CTTAGTCTATATGTTCGTGTTGTCTCGGA
>SRR4252610_5219573___Sbg4
TTAGTCTATATGTTCGTGTTGTCTCGGA
>SRR4252607_13255428___Sbg4
TTGTCTCGGAGGTGGCCCTGGCATTGAGC
>SRR4252609_15430509___Sbg4
CATCATCTGGCCATCATGATCATCTTCA
>SRR4252605_6824934___Sbg4
GTGAATGGTGTCTTTGTCTATATGTTCGTGT
>SRR4252610_4520858___Sbg4
TTTGTCTATATGTTCGTGTTGTCTCGA
>SRR4252605_1357540___Sbg4
ATGTCAGGAAACTTTAGGCCCAAACGGTCCC
>SRR4252607_410811___Sbg4
ACCTACTAGTTGGGGAGGTAAAACTTGGA
>SRR4252609_7537462___Sbg4
GTCATGTAAATAGTACCTACTAGTGGGGA
>SRR4252607_13764832___Sbg4
TCCAGGGAGGCCCTCCATGTCCTTGTGTA
>SRR4252624_4957468___Sbg4
TGAAACTTTAGGCCCAAACGGTCCCCAGA
>SRR4252612_8768712___Sbg4
GTTGTCTCGGAGGTGGCCCTGGCATTGAGC
>SRR4252606_5997466___Sbg4
AGGGAGGCCCTCCATGTCCTTGTGTA
>SRR4252620_1627596___Sbg4
TCTACTAGTGGGGGAGGTAAAACTTGGA
>SRR4252610_6187999___Sbg4
TCATCTGGCCATCATAATCATCTTCATCA
>SRR4252622_4968728___Sbg4
ACTTTAGGCCCAAACGGTCCCCGGACA
>SRR4252611_4761887___Sbg4
TTGTCTCGGAGGTGGCCCTGGCATTGAGT
>SRR4252609_1507753___Sbg4
CCATGTCCTTGTGTAGTCAAAAAAGTA
>SRR4252610_3264479___Sbg4
CTTTGTCTATGTGTTCGTGTTGTCTCGGA
>SRR4252611_1614114___Sbg4
TCCCGACAGGTCTTGGCTCTGTAAGAC
>SRR4252611_11571827___Sbg4
TCTTTGTGTAGTCAAAAAAGTATGAATGTA
>SRR4252624_2050607___Sbg4
CCTATTAGTGGGGGAGGTAAAACTTGGA
>SRR4252611_10683222___Sbg4
TCATCTGGCCATCATGATCATCTTCATCA
>SRR4252610_7791599___Sbg4
ATATGTTCGTGTTGTCTCGGAGGTGGCCCTG
>SRR4252605_8788866___Sbg4
ATCTGGCCATCATAATCATCTTCATCA
>SRR4252610_9037628___Sbg4
CCAGGGAAGCCCTCCATGTCCTTGTGTA
>SRR4252624_3640475___Sbg4
TCCTTGTGTAGTCAAAAAAGTATGAATG

>SRR4252616_6262144___Sbg4
TACATAGTCTATCCTCTCCCGACAGGT
>SRR4252605_2478766___Sbg4
TCTTGGCTCTGTAAGGCCTGGGTGGCGTG
>SRR4252610_12833444___Sbg4
ATGTCAGGAAACTTTAGGCACAAACGGTC
>SRR4252611_710805___Sbg4
TTCGTGTTGTCTCGGAGGTGGCCCTGGA
>SRR4252612_2094408___Sbg4
TCCAGGGAGGTCCTCCATGTCCTTGTGTA
>SRR4252607_1527028___Sbg4
AGGGAGGTCCTCCATGTCCTTGTGTA
>SRR4252610_1450072___Sbg4
ATCATCTTCATCATATTCGCCTTGTTTA
>SRR4252608_6282496___Sbg4
ATGGTGTCTTTGTCTATATGTTCGTG
>SRR4252605_7551827___Sbg4
GTCTTTGTCTATATGTTCGTGTTGTCTCGGA
>SRR4252611_1448773___Sbg4
TCTTTGTCTATATGTTCGTGTTGTCTCGG
>SRR4252606_5457132___Sbg4
ACATGTCCTTGTGTAGTCAAAAAAGTA
>SRR4252606_6036511___Sbg4
ACATGTCCTTGTGTAGTCAAAAAAGTA
>SRR4252619_7939508___Sbg4
TTGTGTAGTCAAAAAAGTATGAATGTATA
>SRR4252615_1230380___Sbg4
TCCCGACAGGTCTTGGCTCTGTAAGG
>SRR4252608_570909___Sbg4
CATCTGGCCATCATGATCATCTTCATCA
>SRR4252606_4498950___Sbg4
AGGGAGGCCCTCCATGTCCTTGTGTA
>SRR4252610_1160499___Sbg4
ATCCGGGTGGCGTGAGTCATGTAAATAGT
>SRR4252612_6127301___Sbg4
TGAGTCATGTAAATAGTACCTACTAGTGG
>SRR4252609_6264249___Sbg4
TAAAGGGAGGCCCTCCATGTCCTTGTGTA
>SRR4252606_4568665___Sbg4
AAAGGGAGGCCCTCCATGTCCTTGTGTA
>SRR4252606_7640324___Sbg4
AAAGGGAGGCCCTCCATGTCCTTGTGTA
>SRR4252608_12512773___Sbg4
AAAGGGAGGCCCTCCATGTCCTTGTGTA
>SRR4252610_11180487___Sbg4
AAAGGGAGGCCCTCCATGTCCTTGTGTA
>SRR4252610_2903591___Sbg4
AAAGGGAGGCCCTCCATGTCCTTGTGTA
>SRR4252611_15112166___Sbg4
AAAGGGAGGCCCTCCATGTCCTTGTGTA
>SRR4252611_6726693___Sbg4
AAAGGGAGGCCCTCCATGTCCTTGTGTA
>SRR4252613_10239186___Sbg4
AAAGGGAGGCCCTCCATGTCCTTGTG
>SRR4252613_10979190___Sbg4
AAAGGGAGGCCCTCCATGTCCTTGTGTA
>SRR4252613_9614514___Sbg4
AAAGGGAGGCCCTCCATGTCCTTGTGTA
>SRR4252614_896800___Sbg4
AAAGGGAGGCCCTCCATGTCCTTGTGTA
>SRR4252608_7549890___Sbg4
AAGGGAGGCCCTCCATGTCCTTGTGTA
>SRR4252610_12763715___Sbg4
AAGGGAGGCCCTCCATGTCCTTGTGTA
>SRR4252611_9635643___Sbg4
TTCTTCATATTCGCCTTGTTGGTATG
>SRR4252607_6848168___Sbg4
TTTTGTCTATATGTTCGTGTTGTCTCGGA
>SRR4252611_12952299___Sbg4
CCTACTAGTGGGGGAGGTAAAACTTGGA

>SRR4252611_4001113___Sbg4
CTGGCCATCATAATCATCTTCATCATAT
>SRR4252611_10307711___Sbg4
CCAGGGAGGTCCTCCATGTCCTTGTGTA
>SRR4252607_3603930___Sbg4
TTGTCTCGGAGGTGGCCCTGGCATTGAGC
>SRR4252610_2493657___Sbg4
AGTCATGTAAATAGTACCTACTAGTGGGG
>SRR4252610_9828052___Sbg4
ATTTGTCTATATGTTCGTGTTGTCTCGGA
>SRR4252613_898815___Sbg4
TCTTTGTCTATATGTTCGTGTTGTCTCGGA
>SRR4252612_6414257___Sbg4
TCTATCCTCTCCCGACAGGTCTTGGCTCT
>SRR4252608_3318011___Sbg4
TTTGTCTACATGTTCGTGTTGTCTCGGA
>SRR4252610_7741790___Sbg4
TTGTCTACATGTTCGTGTTGTCTCGGA
>SRR4252610_13400517___Sbg4
CTCCAGGGAGGCCCTCCATGTCCTTGTGTA
>SRR4252609_3927697___Sbg4
TTGTCTCGGAGGTGGCCCTGGCATTGAGC
>SRR4252612_4449448___Sbg4
TCGGAGGTGGCCCTGGCATTGAGCTGATT
>SRR4252608_2978134___Sbg4
CTTTGTCTACATGTTCGTGTTGTCTCGGA
>SRR4252608_4285751___Sbg4
CTTTGTCTACATGTTCGTGTTGTCTCGGA
>SRR4252610_3248280___Sbg4
CTTTGTCTACATGTTCGTGTTGTCTCGGA
>SRR4252610_6029649___Sbg4
TTTGTCTACATGTTCGTGTTGTCTCGGA
>SRR4252605_2219283___Sbg4
ATAAGACAAATGTCAGGAAACTTTAGGCCT
>SRR4252618_2596383___Sbg4
CATGTCCTTGTGTAGTCAAAAAAGTAT
>SRR4252606_5436975___Sbg4
CTGGCCATCATAATCATCTTCATCATAT
>SRR4252610_11455122___Sbg4
CTTCGTCTATATGTTCGTGTTGTCTCGGA
>SRR4252613_8224194___Sbg4
TTCGTCTATATGTTCGTGTTGTCTCGGA
>SRR4252610_12486808___Sbg4
CATCTGGCCATCATAATCATCTTCAT
>SRR4252611_8231248___Sbg4
TCCTTGTGTAGTCAAAAAAGTATTAATGT
>SRR4252605_12067902___Sbg4
TCTGGAGTTTTTCCTCACCCATAAGACA
>SRR4252605_3131517___Sbg4
TCTGGAGTTTTTCCTCACCCATAAGACA
>SRR4252607_9141223___Sbg4
ATGTCAGGAAACTTTAGGCCCAAACGGTCAT
>SRR4252625_6171838___Sbg4
TCCAGGGAGACCCTCCATGTCCTTGTGTA
>SRR4252610_11732571___Sbg4
TTGGCCATCATAATCATCTTCATCATA
>SRR4252605_6735880___Sbg4
CTTGTTGGTATGAGTGTGAGTGTGAATGGTG
>SRR4252606_2830892___Sbg4
TATATGTTCGTGTTGTCTCGGAGGTGGCC
>SRR4252611_6787155___Sbg4
AGTCATGTAAATAGTACCTACTAGTGGG
>SRR4252622_410676___Sbg4
GGGAGGCCCTCCATGTCCTTGTGTAGTCA
>SRR4252607_2961741___Sbg4
TCCTTGTGTAGTCAAAAAAGTATGGATGT
>SRR4252611_11455895___Sbg4
TCTTCATCATATTCGCCTTGTTGGTATGA
>SRR4252609_13541325___Sbg4
ATCATCTGGCCATCATAATCATCTTCA

>SRR4252606_10347048___Sbg4
TCATCAGGCCATCATAATCATCTTCATCA
>SRR4252610_9327320___Sbg4
ACCAGGGAGGCCCTCCATGTCCTTGTGTA
>SRR4252611_213485___Sbg4
ACCAGGGAGGCCCTCCATGTCCTTGTGTAG
>SRR4252612_1687529___Sbg4
ACCAGGGAGGCCCTCCATGTCCTTGTGTA
>SRR4252606_8652223___Sbg4
TCCTCTCCCGACAGGTCTTGGCTCTGTAA
>SRR4252608_647542___Sbg4
TGTCTATATGTTCGTGTTGTCTCGGAGGT
>SRR4252605_13724500___Sbg4
ATCTTTGTCTATATGTTCGTGTTGTCTCGGA
>SRR4252610_6405325___Sbg4
TTGTCTCGGAGGTGGCCCTGGCATTGAGC
>SRR4252609_4721470___Sbg4
TCCTTGTGTAGTCAAAAAAGTATGAATG
>SRR4252607_6563417___Sbg4
TTTGTCTATATGTTCGTGTTGTCTCGGAG
>SRR4252609_551984___Sbg4
AGTTTCCTGGGTGAGGTTTTTCTGGGC
>SRR4252611_6295421___Sbg4
GCGAGGCCCTCCATGTCCTTGTGTAGTCA
>SRR4252610_241692___Sbg4
CGAGGCCCTCCATGTCCTTGTGTAGTCA
>SRR4252610_5454121___Sbg4
ATGTGATCCATAGTCTATCCTCTCCCGAC
>SRR4252615_4262567___Sbg4
TCTGGCCATCATAATCATCTTCATCATA
>SRR4252607_9250902___Sbg4
TTGTCTCGGAGGTGGCCCTGGCATTGAGC
>SRR4252605_14277439___Sbg4
GGGGAGGTAAAACTTGGAAAATGGAATG
>SRR4252605_2635454___Sbg4
CAAGTTTCCTGGGTGAGGTTTTTCTGGG
>SRR4252605_10093184___Sbg4
TCCTTGTGTAGTCAAAAAAGTATGAATT
>SRR4252611_1839695___Sbg4
TTTGTCTATATGTTCGTGTTGTCTCGGA
>SRR4252605_7137085___Sbg4
TAAGACAAATGTCAGGAAACTTTAGGA
>SRR4252612_5744539___Sbg4
TCTCGGAGGTGGCCCTGGCATTGAGCTGC
>SRR4252607_726252___Sbg4
TTGTCTCGGAGGTGGCCCTGGCATTGAGT
>SRR4252612_6882955___Sbg4
ATGTCAGGAAACTTTAGGCCCAAACGGT
>SRR4252608_18463___Sbg4
TTTGTCTATATGTTCGTGTTGTCTCGGAGG
>SRR4252610_11360172___Sbg4
TGTCTATATGTTCGTGTTGTCTCGGAGGT
>SRR4252605_8019553___Sbg4
AGTCATGTAAATAGTACCTACTAGTG
>SRR4252609_10310867___Sbg4
CCTTGTCTAGTCAAAAAAGTATGAATGTA
>SRR4252614_8185190___Sbg4
TCTTTGTCTATATGTTCGTGTTGTCTCG
>SRR4252605_12826099___Sbg4
TGATCACTTATCCAGGGAGGCCCTCTATG
>SRR4252610_10835468___Sbg4
TCCTTGTGTAGTCAAAAAAGTATGGATGT
>SRR4252610_8276490___Sbg4
TCTTGTCTATATGTTCGTGTTGTCTCGGA
>SRR4252611_5259150___Sbg4
GACATTGAGCTGATCACTTATCCAGGGA
>SRR4252606_3129592___Sbg4
TACTAGTGGGGGAGGTAAAACTTGGAA
>SRR4252609_8520828___Sbg4
TTGTCTCGGAGGTGGCCCTGGCATTGAGCA

>SRR4252610_8430297___Sbg4
TAGGCCCAAACGGTCCCCAGACATCA
>SRR4252610_11215231___Sbg4
TTCGTGTTGTCTCGGAGGTGGCTCTG
>SRR4252610_10109971___Sbg4
ATTTGTCTATATGTTCGTGTTGTCTCGGA
>SRR4252611_3202477___Sbg4
ATGTCAGGAAACTTTAGGCCCAAACGGC
>SRR4252608_3089121___Sbg4
AGCATTGAGCTGATCACTTATCCAGGGA
>SRR4252605_14342533___Sbg4
TCCTTGTGTAGTCAAAAAAGTATGAATG
>SRR4252611_2234873___Sbg4
AGTCATGTAAATAGTACCTACTAGTGGGG
>SRR4252612_4174540___Sbg4
ACCTGGCCATCATAATCATCTTCATCATA
>SRR4252611_14279912___Sbg4
AGGGAGGACCTCCATGTCCTTGTGTAGTCA
>SRR4252609_8900859___Sbg4
TCCTTGTGTAGTCAAAAAAGTATGTATG
>SRR4252612_7252291___Sbg4
TCCTTGTGTAGTCAAAAAAGTATGTATG
>SRR4252621_8424911___Sbg4
TCCTTGTGTAGTCAAAAAAGTATGTATG
>SRR4252605_13516311___Sbg4
ATTTGTCTATATGTTCGTGTTGTCTCGGA
>SRR4252611_13939417___Sbg4
ACCTTCTAGTGGGGGAGGTAAAACTTGG
>SRR4252609_10602838___Sbg4
CCATAGTCTATCCTCTCCCGACAGGTCT
>SRR4252609_10949175___Sbg4
TACCTACTAGTGGGGGAGGTAAAACTTGG
>SRR4252616_2800527___Sbg4
TCATAATCATCTTCATCATACTCGCCTT
>SRR4252621_6755094___Sbg4
TCTTGTGTAGTCAAAAAAGTATGAATGTA
>SRR4252610_12046032___Sbg4
TTTGTCTATATGTTCGTGTTGTCTCGGAG
>SRR4252621_11098058___Sbg4
ACCTACTAGTGGGGGAGGTAAAACTTGGA
>SRR4252610_7414484___Sbg4
AGTCATGTAAATAGTACCTACTAGTGGG
>SRR4252610_4388277___Sbg4
TCCAGGGAGTCCCTCCATGTCCTTGTGTA
>SRR4252610_8066166___Sbg4
AGTCATGTAAATAGTACCTACTAGTGGGG
>SRR4252610_7212749___Sbg4
TCCAGGGAGGCCCTCCATGTCCTTGTGT
>SRR4252611_14663441___Sbg4
ATGTCAGGAAACTTTAGGCCCAAACGGTC
>SRR4252613_7171106___Sbg4
CTGGCCATCATAATCATCTTCATCATAT
>SRR4252608_183438___Sbg4
GTTGGTATGAGTGTGGGTGTGAATGGTGTC
>SRR4252609_7814464___Sbg4
TTTGTCTATATGTTCGTGTTGTCTCGGAG
>SRR4252609_6143744___Sbg4
GGTGGCGTGAGTCATGTAAATAGTACCTA
>SRR4252611_2308682___Sbg4
AGTCATGTAAATAGTACCTACTAGTGGGG
>SRR4252610_6120223___Sbg4
ATCATCAGGCCATCATAATCATCTTCA
>SRR4252605_7475584___Sbg4
AGCATTGAGCTGATCACTTATCCAGGGA
>SRR4252610_7373861___Sbg4
TAGTGGGGGAGGTAAAACTTGGAAAATG
>SRR4252607_10585793___Sbg4
TCCTTGTGTAGTCAAAAAAGTATGAATGT
>SRR4252611_13335598___Sbg4
TCCTTGTGTAGTCAAAAAAGTATGAATGT

>SRR4252618_2176386___Sbg4
AGTGGGGGAGGTAAAACTTGGAAAAT
>SRR4252607_13997450___Sbg4
ACAAATGTCAGGAAACTTTAGGCCCAAACGG
>SRR4252605_1328735___Sbg4
TTTGTCTATATGTTCGTGTTGTCTCGGAG
>SRR4252611_8312573___Sbg4
CCAGGGAGGCCCTCCATGTCCTTGTGTA
>SRR4252623_7624580___Sbg4
TTCTCCTCACCCATAAGACAAATGTCA
>SRR4252611_1723549___Sbg4
CCGAGTGGCGTGAGTCATGTAAATAGTA
>SRR4252611_12996627___Sbg4
AGTGGCGTGAGTCATGTAAATAGTACCTA
>SRR4252607_3213083___Sbg4
TCCTTGTGTAGTCAAAAAAGTATGAATGT
>SRR4252611_14523430___Sbg4
TGTGTAGTCAAAAAAGTATGAATGTTTAT
>SRR4252612_2186300___Sbg4
CTCGCCCATAAGACAAATGTCAGGAAA
>SRR4252607_5745777___Sbg4
TCTTTGTCTATATGTTCGTGTTGTCTCGG
>SRR4252622_9614776___Sbg4
TCCAGGGAGGCCCTCCATGTCCTTGTGTA
>SRR4252605_7708475___Sbg4
GTGAATGTATATGTGATCCATAGTCTA
>SRR4252622_8212886___Sbg4
GGGTGGCGTGAGTCATGTAAATAGTACCTA
>SRR4252615_7908162___Sbg4
CATGTCCTTGTGTAGTCAAAAAAGTATGA
>SRR4252622_8211018___Sbg4
TCCTTGTGTAGTCAAAAAAGTATGAATG
>SRR4252610_12154785___Sbg4
ACCCTCCATGTCCTTGTGTAGTCAAA
>SRR4252608_7215485___Sbg4
AATCATCTGGCCATCATGATCATCTTCA
>SRR4252610_557130___Sbg4
AGTCATGTAAATAGTACCTACTAGTGGGG
>SRR4252609_3042921___Sbg4
ACATCATCTGGCCATCATGATCATCTTCA
>SRR4252623_12792605___Sbg4
AGGTGGCCCTGGCATTGAGCTGATCACTTA
>SRR4252624_6390093___Sbg4
TCATGTAAATAGTACCTACTAGTGGGGGA
>SRR4252612_5090508___Sbg4
AGTCATTTAAATAGTACCTACTAGTGG
>SRR4252606_677012___Sbg4
GAAGACAAATGTCAGGAAACTTTAGGCCCA
>SRR4252612_7846049___Sbg4
TGTCAGGAAACTTTAGGCCCAAACGGTCCT
>SRR4252610_4203482___Sbg4
TCCAGGGAGGTCCTCCATGTCCTTGTGTA
>SRR4252615_14675602___Sbg4
TATGTTCGTGTTGTCTCGGAGGTGGCT
>SRR4252605_10002688___Sbg4
CATCATCTGGCCATCATGATCATCTTCA
>SRR4252612_3280878___Sbg4
TCCTTGTGTAGTCAAAAAAGTGTGAAT
>SRR4252611_14629686___Sbg4
AGGCCCACACGGTCCCCAGACATCAT
>SRR4252606_1656747___Sbg4
TCCAGGGAGGCCCTCCATGTCCTTGTGTA
>SRR4252607_13066512___Sbg4
TGTCTATATGTTCGTGTTGTCTCGGAGGC
>SRR4252621_106797___Sbg4
ATCCATGTCCTTGTGTAGTCAAAAAAGTATG
>SRR4252609_4842244___Sbg4
CCATGTCCTTGTGTAGTCAAAAAAGTA
>SRR4252605_3515533___Sbg4
GGGAGGCTCTCCATGTCCTTGTGTAGTCA

>SRR4252616_4075069___Sbg4
GGGAGGCCTTCCATGTCCTTGTGTAGTCA
>SRR4252612_1015383___Sbg4
TATGTCTTTGTCTATATGTTCGTGTT
>SRR4252626_9435464___Sbg4
ATGTCTTTGTCTATATGTTCGTGTTGTCT
>SRR4252610_6942063___Sbg4
CTTTGTCTATATGTTCGTGTTGTCTCGGA
>SRR4252605_13490665___Sbg4
TCCTTGTGTAGTCAAAAAAGTATGAATG
>SRR4252609_11797513___Sbg4
AATGTCAGGAAACTTTAGACCCAAACGGT
>SRR4252610_4585895___Sbg4
CTTTGTCTATATGTTCGTGTTGTCTCGGA
>SRR4252611_5553305___Sbg4
TTTGTCTATATGTTCGTGTTGTCTCGGAG
>SRR4252610_7504887___Sbg4
GACTTTGTCTATATGTTCGTGTTGTCTCGGA
>SRR4252605_7255371___Sbg4
TCCTTGTGTAGTCAAAAAAGTATGAATGA
>SRR4252612_6110152___Sbg4
AGTCATGTAAATAGTACCTACTAGTGGG
>SRR4252623_4088329___Sbg4
AGGCCCAAACGGTCCCCAGACATCAT
>SRR4252606_4371592___Sbg4
CTTGTTGGTATGAGTGTGGGTGTGAATGGT
>SRR4252606_7859448___Sbg4
TGTGGGTGTGAATGGTGTTTTTGTCTATA
>SRR4252611_12091___Sbg4
AGTCATGTAAATAGTACCTACTAGTGGG
>SRR4252611_9318278___Sbg4
GTTGTCTCGGAGGTGGCCCTGGCATTGAGC
>SRR4252611_13833885___Sbg4
TTTGTCTATATGTTCGTGTTGTCTCGGAG
>SRR4252616_12919074___Sbg4
ACCCGGGTGGCGTGAGTCATGTAAATAGT
>SRR4252612_3706900___Sbg4
TCCTTGTGTAGTCAAAAAAGTATGAATGT
>SRR4252609_6640628___Sbg4
TTTGTCTATATGTTCGTGTTGTCTCGGC
>SRR4252609_1256057___Sbg4
ATGTCCATGTGTAGTCAAAAAAGTATGA
>SRR4252619_7704561___Sbg4
TGTGTAGTCAAAAAAGTATGAATGTA
>SRR4252611_7470849___Sbg4
AGAGGTGGCCCTGGCATTGAGCTGATC
>SRR4252611_2525237___Sbg4
AGTCATGTAAATAGTACCTACTAGTGGG
>SRR4252608_4546455___Sbg4
ACTTTGTCTATATGTTCGTGTTGTCTCGGA
>SRR4252610_5959725___Sbg4
TCCTTGTGTAGTCAAAAAAGTATGAATG
>SRR4252622_877025___Sbg4
TTCAGGGAGGCCCTCCATGTCCTTGTGTA
>SRR4252623_13125173___Sbg4
TCAGGGAGGCCCTCCATGTCCTTGTGTA
>SRR4252605_419846___Sbg4
TCCTTGTGTAGTCAAAAAAGAATGAATG
>SRR4252623_3401873___Sbg4
TCTTGGCTCTGTAAGACCCGGGTGGCGT
>SRR4252610_893257___Sbg4
ACCTACTAGTGGGGGAGGTAAAACTTGGA
>SRR4252610_11930907___Sbg4
AGTCATGTAAATAGTACCTACTAGTGGGG
>SRR4252611_8299714___Sbg4
GTCCTTGTGTAGTCAAAAAAGTATGAATGTA
>SRR4252610_8464419___Sbg4
TGTAGTCAAAAAAGTATGAATGTATATGC
>SRR4252608_10656202___Sbg4
ACATCATCTGGCCATCATGATCATCTTC

>SRR4252611_2555442___Sbg4
ATCATCTTCATCATATTCGCCTTGTTGA
>SRR4252610_6354817___Sbg4
AGGGAGGCCCTCCATGTCCTTGTGTA
>SRR4252606_4108734___Sbg4
TTGTCTCGGAGGTGGCCCTGGCATTGAGT
>SRR4252605_6978409___Sbg4
GTCTCGGAGGTGGCCCTGGCATTGAGT
>SRR4252611_2176854___Sbg4
TCCTCCTTGTCCTTGTGTAGTCAAAAA
>SRR4252605_6495969___Sbg4
TTGTCTCGGAGGTGGCCCTGGCATTGAGT
>SRR4252607_10500991___Sbg4
TTGTCTCGGAGGTGGCCCTGGCATTGAGT
>SRR4252607_11940839___Sbg4
TTGTCTCGGAGGTGGCCCTGGCATTGAGT
>SRR4252607_2911892___Sbg4
TTGTCTCGGAGGTGGCCCTGGCATTGAGT
>SRR4252607_9446210___Sbg4
ATTTGTCTATATGTTCGTGTTGTCTCGGA
>SRR4252622_9414586___Sbg4
ACCGGGTGGCGTGAGTCATGTAAATAGTA
>SRR4252610_4101454___Sbg4
GAGTCATGTAAATAGTACCTACTAGTGGG
>SRR4252610_64748___Sbg4
GTCATGTAAATAGTACCTACTAGTGGGG
>SRR4252622_6883744___Sbg4
GCCTACTAGTGGGGGAGGTAAAACTTGGA
>SRR4252607_12214509___Sbg4
GTGGGGGAGGTAAAACTTGGAAAATGGA
>SRR4252614_3936903___Sbg4
TCTATATGTTCGTGTTGTCTCGGAGGT
>SRR4252607_4305583___Sbg4
TTGTCTCGGAGGTGGCCCTGGCCTTGAGC
>SRR4252611_2306907___Sbg4
AGTCATGTAAATAGTACCTACTAGTGGGG
>SRR4252605_5336762___Sbg4
TCTTGTGTAGTCAAAAAAGTATGAATGTA
>SRR4252611_4761944___Sbg4
ATGTCAGGAAACTTTAGGCCCAAACGGTCCC
>SRR4252609_3535304___Sbg4
TTGTCTCGGAGGTGGCCCTGGCATTGAGC
>SRR4252605_14504278___Sbg4
CATCTGGCCATCATAATCATCTTCATCA
>SRR4252611_5069713___Sbg4
TCCAGGGAGGCCCTCCATGTCCTTGTGTA
>SRR4252623_13118794___Sbg4
CCTACTACTGGGGGAGGTAAAACTTGGA
>SRR4252610_1877795___Sbg4
TGTCTATATGTTCGTGTTGTCTCGGA
>SRR4252621_10991663___Sbg4
ATGTCCTTGTGTAGTCAAAAAAGTATGAATG
>SRR4252608_2496003___Sbg4
AGTCATGTAAATAGTACCTACTAGTGGG
>SRR4252610_7758356___Sbg4
AGTCATGTAAATAGTACCTACTAGTGGGG
>SRR4252626_9048164___Sbg4
AGTCATGTAAATAGTACCTACTAGTGGG
>SRR4252611_10361112___Sbg4
CCATGTCCTTGTGTAGTCAAAAAAGTA
>SRR4252605_14093887___Sbg4
GGGAGGCCCTCCATGTCCTTGTGTAGTCA
>SRR4252611_10594213___Sbg4
AGGTCTTGGCTCTGTAAGACCCGGGTGGCGT
>SRR4252608_4268287___Sbg4
ATGAATGTATATGTGATCCATAGTCTA
>SRR4252608_7545920___Sbg4
TCCTTGTGTAGTCAAAAAAGTATGAATG
>SRR4252611_15413005___Sbg4
TGTGTAGTCAAAAAAGTATGAATGTAT

>SRR4252605_10333312___Sbg4
AGGTGGCGTGAGTCATGTAAATAGTACCTA
>SRR4252607_14026504___Sbg4
AGGTGGCGTGAGTCATGTAAATAGTACCTA
>SRR4252611_5028582___Sbg4
AGGTGGCGTGAGTCATGTAAATAGTACCTA
>SRR4252624_742684___Sbg4
TTTGTCTATATGTTCGTGTTATCTCGGAG
>SRR4252620_10407293___Sbg4
TGTCTATATGTTCGTGTTATCTCGGAG
>SRR4252606_5695388___Sbg4
AATGTCCTTGTGTAGTCAAAAAAGTATGA
>SRR4252614_3250269___Sbg4
TCCTTGTGTAGTCAAAAAAGTATGAATGTT
>SRR4252616_11487646___Sbg4
CATCTTCATCATATTCGCCTTGTTGGTA
>SRR4252611_12613935___Sbg4
AGTCATGTAAATAGTACCTACTAGTGGG
>SRR4252624_10302853___Sbg4
TCCTTGTGTAGTCAAAAAAGTATGAATA
>SRR4252606_8318146___Sbg4
GAGTCATGTAAATAGTACCTACTAGTGGG
>SRR4252622_1903224___Sbg4
CCTTGTGTAGTCAAAAAAGTATGAATGTA
>SRR4252605_10814129___Sbg4
AAGACAAATGTCAGGAAACTTTAGGCCT
>SRR4252610_4529388___Sbg4
TGTCTATATGTTCGTGTTGTCTCGGA
>SRR4252611_3862287___Sbg4
ATGTCCTTGTGTAGTCAAAAAAGTATGA
>SRR4252611_2085744___Sbg4
TAAGACAAATGTCAGGAAACTTTAGGCCCA
>SRR4252610_518016___Sbg4
AAGACAAATGCCAGGAAACTTTAGGCCCA
>SRR4252605_158954___Sbg4
CCGGGTAGCGTGAGTCATGTAAATAGTA
>SRR4252610_592828___Sbg4
AAGTCATGTAAATAGTACCTACTAGTGGG
>SRR4252616_10578947___Sbg4
GTCCCCAGACATCATCTGGCCATCAT
>SRR4252614_7280293___Sbg4
TATGTTCGTGTTGTCTCGGAGGTGGT
>SRR4252609_10150404___Sbg4
TGTCAGGAAACTTTAGGCCCAAACGGTCCT
>SRR4252610_11054069___Sbg4
TATCTGGCCATCATAATCATCTTCATCA
>SRR4252606_8956755___Sbg4
TCCAGGGAAGCCCTCCATGTCCTTGTGTA
>SRR4252607_12813646___Sbg4
AGTCATGTAAATAGTACCTACTAGTGGGG
>SRR4252610_2925808___Sbg4
AATGGTGTCTTTGTCTATATGTTCGTGTT
>SRR4252610_3834191___Sbg4
CTTTGTCTATATGTTCGTGTTGTCTCGGA
>SRR4252610_433052___Sbg4
TTTGTCTATATGTTCGTGTTGTCTCGGA
>SRR4252608_10922766___Sbg4
AGTCATGTAAATAGTACCTACTAGTGG
>SRR4252609_201222___Sbg4
GTCATGTAAATAGTACCTACTAGTGGGG
>SRR4252613_8379007___Sbg4
TGTCTATATGTTCGTGTTGTCTCGGAGGT
>SRR4252624_10403671___Sbg4
GGGTGGTGTGAGTCATGTAAATAGTACCTA
>SRR4252606_5510950___Sbg4
AGGGAGGCCCTCCATGTCCTTGTGTA
>SRR4252608_10889832___Sbg4
TCCTTGTGTAGTCAAAAATGTATGAATGT
>SRR4252608_6843998___Sbg4
TCCTTGTGTAGCCAAAAAAGTATGAATGTA

>SRR4252612_5892838___Sbg4
TCTACTAGTGGGGGAGGTAAAACTTGGA
>SRR4252609_7022207___Sbg4
CAACATCTGGCCATCATAATCATCTTCA
>SRR4252608_1368664___Sbg4
ACATCTGGCCATCATAATCATCTTCATCA
>SRR4252605_398627___Sbg4
CTTTGTCTTTATGTTCGTGTTGTCTCGGA
>SRR4252622_2114322___Sbg4
TTGTCTTTATGTTCGTGTTGTCTCGGA
>SRR4252606_9759334___Sbg4
CCCAAACGGTCCCCAGACATCATCTGGC
>SRR4252609_15913073___Sbg4
ATCATAATCATCTTCATCATATTCGCC
>SRR4252619_12871022___Sbg4
CTGGGGAGGCCCTCCATGTCCTTGTGTA
>SRR4252610_12906638___Sbg4
TGGGGAGGCCCTCCATGTCCTTGTGTA
>SRR4252610_1997963___Sbg4
TGGGGAGGCCCTCCATGTCCTTGTGTA
>SRR4252616_12868702___Sbg4
GGGGAGGCCCTCCATGTCCTTGTGTA
>SRR4252610_809121___Sbg4
AGTCATGTAAATAGTACCTACTAGTGGGG
>SRR4252623_5309777___Sbg4
ATTAGGCCCAAACGGTCCCCAGACATCA
>SRR4252612_286486___Sbg4
ATGTCAGGAAACTTTAGGCCCAAACGGT
>SRR4252610_856886___Sbg4
ATAGTCTATCCTCTCCCGACAGGTCTTG
>SRR4252608_1028257___Sbg4
ACAACATCTGGCCATCATAATCATCTTCA
>SRR4252609_2712329___Sbg4
ACAACATCTGGCCATCATAATCATCTTCA
>SRR4252610_1419872___Sbg4
ACAACATCTGGCCATCATAATCATCTTCA
>SRR4252612_1584597___Sbg4
ACAACATCTGGCCATCATAATCATCTTCA
>SRR4252607_8971741___Sbg4
TTTGTCTATATGTTCGTGTTGTCTCGGAGGT
>SRR4252610_12996913___Sbg4
TTCGTGTTGTCTCGGAGGTGGCCCTAGC
>SRR4252606_6769489___Sbg4
AATAGTCTATCCTCTCCCGACAGGTCTTG
>SRR4252615_15645366___Sbg4
AGGTCTTGGCTCTGTAAGTCCCGGGTGGC
>SRR4252624_1347314___Sbg4
ATCTGGCCATCATAATCATCTTCATCATA
>SRR4252610_6735999___Sbg4
TGGGAGGCCCTCCATGTCCTTGTGTA
>SRR4252607_4413478___Sbg4
ATTTGTCTACATGTTCGTGTTGTCTCGGA
>SRR4252606_2223316___Sbg4
TTGTCTACATGTTCGTGTTGTCTCGGA
>SRR4252625_3500174___Sbg4
TTGTCTATATGTTCGTGTTGTCTCGGAGC
>SRR4252608_546863___Sbg4
TCCTTGTGTAGTCAAAAAAGTATGAGT
>SRR4252611_2136048___Sbg4
AATCCTCTCCCGACAGGTCTTGGCTCTGTA
>SRR4252609_12690026___Sbg4
TTGTCTCGGAGGTGGCCCTGGCATTGAGT
>SRR4252613_398980___Sbg4
TCCAGGGTGGCCCTCCATGTCCTTGTGTA
>SRR4252609_15752859___Sbg4
ATCATCTTCATCATATTCGCCTTGTTTA
>SRR4252613_9914260___Sbg4
ACCTACTAGTGGGGGAGGTAAAACTTGGA
>SRR4252605_6306651___Sbg4
CAGGTGGCGTGAGTCATGTAAATAGTA

>SRR4252613_1609532___Sbg4
TTGTCTATATGTTCGTGTTGTCTTGGA
>SRR4252612_5066711___Sbg4
CCAGGGAGGCCCTCCATGTCCTTGTGTA
>SRR4252606_3199108___Sbg4
CTTCGTCTATATGTTCGTGTTGTCTCGGA
>SRR4252607_7667554___Sbg4
CTTCGTCTATATGTTCGTGTTGTCTCGGA
>SRR4252622_3968807___Sbg4
TGTAGTCAAAAAAGTATGAATGTATA
>SRR4252605_10676983___Sbg4
GTAGTCTATCCTCTCCCGACAGGTCTTGG
>SRR4252624_6553428___Sbg4
GTAGTCTATCCTCTCCCGACAGGTCTTGGC
>SRR4252610_10864755___Sbg4
AGACATGTAAATAGTACCTACTAGTGGGG
>SRR4252609_1256398___Sbg4
TCATCAGGCCATCATAATCATCTTCA
>SRR4252608_11356466___Sbg4
GGTCATGTAAATAGTACCTACTAGTGGGG
>SRR4252610_11136192___Sbg4
GGTCATGTAAATAGTACCTACTAGTGGG
>SRR4252611_6175515___Sbg4
GGTCATGTAAATAGTACCTACTAGTGGGG
>SRR4252624_70751___Sbg4
CATCATCTGGCCATCATGATCATCTTCA
>SRR4252613_2581318___Sbg4
TCTTGGCTCTGTAAGACCCGGGTGGCGT
>SRR4252611_1282468___Sbg4
TGTCTCGGAGGTGGCCCTGGCATTGAGCT
>SRR4252606_2357900___Sbg4
GAGGCCCTCCATGTCCTTGTGTAGTCAAA
>SRR4252610_13310715___Sbg4
GTCTATATGTTCGTGTTGTCTCGGAGGT
>SRR4252614_2504513___Sbg4
TGTAGTCAAAAAAGTATGAATGTATATGT
>SRR4252611_11404247___Sbg4
TCTCCCGACAGGTCTTGGCTCTGTAAGGCCT
>SRR4252608_9804003___Sbg4
GTGGGTGTGAATGGTGTCTTTGTCTATA
>SRR4252610_813458___Sbg4
CCATGTTCTTGTGTAGTCAAAAAAGTA
>SRR4252614_3544161___Sbg4
GTAGGCCCAAACGGTCCCCAGACATCAT
>SRR4252606_2547330___Sbg4
CATCTGGCCATCATAATCATCTTCATCA
>SRR4252605_10670697___Sbg4
GGCGTGAGTCATGTAAATAGTACCTACTA
>SRR4252606_1748986___Sbg4
TGTAGTCAAAAAAGTATGAATGTATA
>SRR4252607_5281503___Sbg4
TCCTTGTGTAGTCAAAAAAGTATGAATA
>SRR4252607_1291601___Sbg4
TCCTTGTGTAGTCAAAAAAGTATGAAT
>SRR4252609_15704417___Sbg4
GTCTCGGAGGTGGCCCTGGCATTGAGC
>SRR4252609_7088406___Sbg4
TTGTCTCGGAGGTGGCCCTGACATTGAGC
>SRR4252610_2473716___Sbg4
CCTACTAGTGGGGGAGGTAAAACTTGGA
>SRR4252611_4780517___Sbg4
GAGACAACACGAACATATAGACAAAGA
>SRR4252612_4771735___Sbg4
TACTTTTTTGACTACACAAGGACATGG
>SRR4252619_9479743___Sbg4
TCCGAGACAACACGAACATATAGACA
>SRR4252611_1120682___Sbg4
TGGGCCTAAAGTTTCCTGACATTTGTCTT
>SRR4252610_6078441___Sbg4
CCCACTAGTAGGTACTATTTACATGACT

>SRR4252605_5669139___Sbg4
CCACTAGTAGGTACTATTTACATGACT
>SRR4252624_1803749___Sbg4
TCCGAGACAACACGAACATATAGACAAAG
>SRR4252605_13555644___Sbg4
TACTTTTTTGACTACACAAGGACATGG
>SRR4252609_2468418___Sbg4
TATAGACAAAGACACCATTCACACCCACAC
>SRR4252611_14387339___Sbg4
CCCACTAGTAGGTACTATTTACATGACT
>SRR4252612_8329015___Sbg4
CCCCACTAGTAGGTACTATTTACATGACT
>SRR4252610_2776567___Sbg4
TACTATTTACATGACTCACGCCACCCGG
>SRR4252608_2511787___Sbg4
ACATTCATACTTTTTTGACTACACAAGGA
>SRR4252610_12054___Sbg4
CCCCACTAGTAGGTACTATTTACATGACT
>SRR4252612_424284___Sbg4
CAAGGACATGGAGGGCCTCCCTGGAT
>SRR4252607_1361937___Sbg4
GAAGATGATCATGATGGCCAGATGATGT
>SRR4252610_7211167___Sbg4
CCCCACTAGTAGGTACTATTTACATGACT
>SRR4252608_1739468___Sbg4
TCCGAGACAACACGAACATATAGACAAAG
>SRR4252607_9919211___Sbg4
ACATTCATACTTTTTTGACTACACAAGGA
>SRR4252612_2729867___Sbg4
ACAACACGAACATATAGACAAAGACACCA
>SRR4252605_12917763___Sbg4
TACTTTTTTGACTACACAAGGACATGGA
>SRR4252624_1289456___Sbg4
CCCCAGAAAAACCTCACCCAGGAAACTTG
>SRR4252610_7883422___Sbg4
AGACAACACGAACATATAGACAAAGA
>SRR4252607_6226502___Sbg4
ACATTCATACTTTTTTGACTACACAAGGA
>SRR4252611_14237499___Sbg4
CCGTTTGGGCCTAAAGTTTCCTGACAT
>SRR4252616_14149996___Sbg4
GGCCTAAAGTTTCCTGACATTTGTCTTA
>SRR4252610_664057___Sbg4
GCTCAATGCCAGGGCCACCTCCGAGACAA
>SRR4252606_3057659___Sbg4
TACTATTTACATGACTCACGCCACCCGG
>SRR4252612_7402498___Sbg4
CAGCTCAATGCCAGGGCCACCTCCGAGACA
>SRR4252605_7432449___Sbg4
CCCCACTAGTAGGTACTATTTACATGACT
>SRR4252610_10500829___Sbg4
CCCACTAGTAGGTACTATTTACATGACT
>SRR4252616_4469253___Sbg4
CATTCATACTTTTTTGACTACACAAGGA
>SRR4252607_10181056___Sbg4
CCCCACTAGTAGGTACTATTTACATGACT
>SRR4252617_9096450___Sbg4
AGGACATGGAGGGCCTCCCTGGATAAGT
>SRR4252614_7077767___Sbg4
AGACAACACGAACATATAGACAAAGACA
>SRR4252608_4631742___Sbg4
CATTCATACTTTTTTGACTACACAAGGA
>SRR4252610_8378910___Sbg4
TACACAAGGACATGGAGGGCCTCCCT
>SRR4252610_13186739___Sbg4
CCCACTAGTAGGTACTATTTACATGACT
>SRR4252611_4219797___Sbg4
CATGGAGGGCCTCCCTGGATAAGTGATCA
>SRR4252607_8569731___Sbg4
ACCTCCGAGACAACACGAACATATAGACAAA

>SRR4252612_2985782___Sbg4
CAACAAGGCGAATATGATGAAGATGATCA
>SRR4252610_1647053___Sbg4
CCCCACTAGTAGGTACTATTTACATGACT
>SRR4252609_5884243___Sbg4
CCTCCGAGACAACACGAACATATAGACA
>SRR4252610_13351793___Sbg4
CCCCACTAGTAGGTACTATTTACATGACT
>SRR4252608_3663136___Sbg4
CCCACTAGTAGGTACTATTTACATGACT
>SRR4252621_60142___Sbg4
GCTCAATGCCAGGGCCACCTCCGAGACAA
>SRR4252611_3670574___Sbg4
TTTGGGCCTAAAGTTTCCTGACATTTGT
>SRR4252610_5911481___Sbg4
CCCCACTAGTAGGTACTATTTACATGACT
>SRR4252609_15417077___Sbg4
GCTCAATGCCAGGGCCACCTCCGAGACAACA
>SRR4252616_11503742___Sbg4
AAGGCGAATATGATGAAGATGATCATGAT
>SRR4252608_10783546___Sbg4
CCCACTAGTAGGTACTATTTACATGACT
>SRR4252609_8336477___Sbg4
TGTCTTATGGGTGAGGAAAAACCCCAG
>SRR4252605_666854___Sbg4
TAGGTACTATTTACATGACTCACGCCAC
>SRR4252605_13083576___Sbg4
ACATTCATACTTTTTTGACTACACAAGGA
>SRR4252610_10173801___Sbg4
CCCACTAGTAGGTACTATTTACATGACT
>SRR4252615_11398878___Sbg4
ATTCATACTTTTTTGACTACACAAGGA
>SRR4252610_1762078___Sbg4
TGGGCCTAAAGTTTCCTGACATTTGTCTTA
>SRR4252611_6902627___Sbg4
TATTTACATGACTCACGCCACCCGGGCCT
>SRR4252611_5082597___Sbg4
CACGCCACCCGGGCCTTACAGAGCCAAGA
>SRR4252609_10558170___Sbg4
TACTTTTTTGACTACACAAGGACATGG
>SRR4252611_7286543___Sbg4
CCCCACTAGTAGGTACTATTTACATGAC
>SRR4252613_10360700___Sbg4
ACCTGTCGGGAGAGGATAGACTATGGA
>SRR4252605_3595120___Sbg4
ACATTCATACTTTTTTGACTACACAAGGA
>SRR4252608_3744912___Sbg4
CCCCACTAGTAGGTACTATTTACATGACT
>SRR4252606_10414336___Sbg4
CCAAGACCTGTCGGGAGAGGATAGACTAT
>SRR4252618_2967472___Sbg4
CACCTCCGAGACAACACGAACATATAGA
>SRR4252611_13694905___Sbg4
TCCGAGACAACACGAACATATAGACA
>SRR4252606_8416544___Sbg4
CCCCACTAGTAGGTACTATTTACATGAC
>SRR4252620_8304675___Sbg4
ACACGAACATATAGACAAAGACACCATTCAC
>SRR4252615_14595580___Sbg4
CACGCCACCCGGGCCTTACAGAGCCAAGA
>SRR4252609_2572797___Sbg4
TACATTCATACTTTTTTGACTACACAAGGA
>SRR4252612_3853050___Sbg4
TACTTTTTTGACTACACAAGGACATGGA
>SRR4252615_14210648___Sbg4
TACTTTTTTGACTACACAAGGACATGG
>SRR4252606_7113384___Sbg4
TACACAAGGACATGGAGGGCTTCCCTG
>SRR4252609_3071609___Sbg4
TGAAGATGATCATGATGGCCAGATGATGT

>SRR4252611_7466198___Sbg4
TTTGGGCCTAAAGTTTCCTGACATTTGTCTT
>SRR4252606_9099736___Sbg4
TGATGAAGATGATCATGATGGCCAGA
>SRR4252613_10168272___Sbg4
TACACAAGGACATGGAGGGCCTCCCTGGA
>SRR4252625_2501817___Sbg4
ATGAAGATGATCATGATGGCCAGATGAT
>SRR4252605_12782274___Sbg4
CATACTTTTTTGACTACACAAGGACATGG
>SRR4252609_14147213___Sbg4
TACACAAGGACATGGAGGGCCTCCCTGG
>SRR4252622_6797903___Sbg4
TCCAAGTTTTACCTCCCCCACTAGTAGGT
>SRR4252621_3670524___Sbg4
TACACAAGGACATGGAGGGCCTCCCTGGAT
>SRR4252610_12600435___Sbg4
CTCCGAGACAACACGAACATATAGAC
>SRR4252612_7162746___Sbg4
TGATGTCTGGGGACCGTTTGGGCCTAAAG
>SRR4252623_4772622___Sbg4
TCCAAGTTTTACCTCCCCCACTAGTAGGT
>SRR4252621_7603125___Sbg4
TACATTCATACTTTTTTGACTACACAAGGA
>SRR4252610_4408533___Sbg4
CCCACTAGTAGGTACTATTTACATGACT
>SRR4252608_10661457___Sbg4
GATGAAGATGATCATGATGGCCAGATG
>SRR4252609_1318524___Sbg4
GCTCAATGTCAGGGCCACCTCCGAGACAA
>SRR4252615_5398684___Sbg4
CACGCCACCCGGGCCTTACAGAGCCAAGA
>SRR4252610_5307704___Sbg4
GCTCAATGCCAGGGCCACCTCCGAGACA
>SRR4252610_168696___Sbg4
CCAAGTTTTACCTCCCCCACTAGTAGGT
>SRR4252607_52412___Sbg4
TCCCCCACTAGTAGGTACTATTTACATGA
>SRR4252605_8316092___Sbg4
AGACCTGTCGGGAGAGGATAGACTATGGA
>SRR4252616_5024353___Sbg4
CTTTTTTGACTACACAAGGACATGGA
>SRR4252611_8635412___Sbg4
TGACTACACAAGGACATGGAGGGCCTCCCT
>SRR4252605_7527817___Sbg4
CCGTTTGGGCCTAAAGTTTCCTGACATT
>SRR4252608_459584___Sbg4
CCCACTAGTAGGTACTATTTACATGACT
>SRR4252611_3712653___Sbg4
TACAGAGCCAAGACCTGTCGGGAGAGGATA
>SRR4252607_6129173___Sbg4
TGAAGATGATCATGATGGCCAGATGATGT
>SRR4252615_7455556___Sbg4
TCACGCCACCCGGGCCTTACAGAGCCAAGA
>SRR4252608_1456350___Sbg4
TCCGAGACAACACGAACATATAGACAAAG
>SRR4252622_2996857___Sbg4
TATAGACAAAGACACCATTCACACCCACAC
>SRR4252610_13403613___Sbg4
TCCGAGACAACACGAACATATAGACAAAGA
>SRR4252606_8108619___Sbg4
GCCTCCGAGACAACACGAACATATAGACAAA
>SRR4252622_5281171___Sbg4
GCCTCCGAGACAACACGAACATATAGACA
>SRR4252610_6016959___Sbg4
CCCCACTAGTAGGTACTATTTACATGACT
>SRR4252610_3881115___Sbg4
TACACAAGGACATGGAGGGCCTCACTGGA
>SRR4252606_5160958___Sbg4
CCCCACTAGTAGGTACTATTTACATGACT

>SRR4252608_12954900___Sbg4
TACACAAGGACATGGAGGGCCTCCCTG
>SRR4252621_12962796___Sbg4
TCCGAGACAACACGAACATATAGACAAAG
>SRR4252613_5540307___Sbg4
GCCAAGACCTGTCGGGAGAGGATAGACTAT
>SRR4252611_3924102___Sbg4
TACACAAGGACATGGAGGGCCTCCCTGGA
>SRR4252605_5896709___Sbg4
CCCCACTAGTAGGTACTATTTACATGACT
>SRR4252608_9586571___Sbg4
CCCCACTAGTAGGTACTATTTACATGACT
>SRR4252611_14749437___Sbg4
CATTCATACTTTTTTGACTACACAAGGA
>SRR4252611_1816858___Sbg4
CTCCGAGACAACACGAACATATAGACAAA
>SRR4252610_9714046___Sbg4
CCCACTAGTAGGTACTATTTACATGACT
>SRR4252606_2983294___Sbg4
CACCATTCACACCCACACTCATACCAACAAG
>SRR4252612_6093971___Sbg4
TACACAAGGACATGGAGGGCCTCCCTGGAT
>SRR4252612_9036773___Sbg4
CCTGTCGGGAGAGGATAGACTATGGATC
>SRR4252610_5477652___Sbg4
CGAGACAACACGAACATATAGACAAAGAC
>SRR4252610_3901174___Sbg4
TACACAAGGACATGGAGGGCCTCCCTGGA
>SRR4252610_11289139___Sbg4
TCCAAGTTTTACCTCCCCCACTAGTAGG
>SRR4252609_2691446___Sbg4
GCTCAATGCCAGGGCCACCTCCGAGACAA
>SRR4252613_4100262___Sbg4
CCTGTCGGGAGAGGATAGACTATGGATC
>SRR4252605_14519943___Sbg4
TACCAACAAGGCGAATATGATGAAGATG
>SRR4252618_2957281___Sbg4
TCCAAGTTTTACCTCCCCCACTAGTAG
>SRR4252607_3454210___Sbg4
ACTCATACCAACAAGGCGAATATGATGAAG
>SRR4252620_7549698___Sbg4
TGAAGATGATCATGATGGCCAGATGATGT
>SRR4252612_8268664___Sbg4
CATTCATACTTTTTTGACTACACAAGGA
>SRR4252612_2146442___Sbg4
TACACAAGGACATGGAGGGCCTCCCTGG
>SRR4252605_10513289___Sbg4
TGAAGATGATCATGATGGCCAGATGATGT
>SRR4252607_8839425___Sbg4
TACCGTTTGGGCCTAAAGTTTCCTGACATT
>SRR4252608_6141406___Sbg4
TACCGTTTGGGCCTAAAGTTTCCTGACAT
>SRR4252609_16401836___Sbg4
TGGGCCTAAAGTTTCCTGACATTTGTCTTAT
>SRR4252623_9527068___Sbg4
ACCTCCGAGACAACACGAACATATAGACA
>SRR4252611_13629105___Sbg4
TGAAGATGATCATGATGGCCAGATGATGT
>SRR4252605_4137940___Sbg4
TTTGACTACACAAGGACATGGAGGGCC
>SRR4252605_355581___Sbg4
GCTCAATGCCAGGGCCACCTCCGAGACA
>SRR4252611_1983848___Sbg4
TAGGTACTATTTACATGACTCACGCCACCC
>SRR4252609_8530238___Sbg4
CTAGTAGGTACTATTTACATGACTCACGC
>SRR4252619_14459361___Sbg4
CCAAGTTTTACCTCCCCCACTAGTAGGTACT
>SRR4252611_12934401___Sbg4
CCCACTAGTAGGTACTATTTACATGAT

>SRR4252606_4846379___Sbg4
GCTCAATGCCAGGGCCACCTCCGAGACAA
>SRR4252607_10668284___Sbg4
GCTCAATGCCAGGGCCACCTCCGAGACA
>SRR4252606_126724___Sbg4
ACCTCCGAGACAACACGAACATATAGACA
>SRR4252610_7641932___Sbg4
CAAGACCTGTCGGGAGAGGATAGACTAT
>SRR4252611_6672093___Sbg4
TACACAAGGACATGGAGGGCCTCCCTGGATA
>SRR4252626_850176___Sbg4
TTTTGACTACACAAGGACATGGAGGGCCTC
>SRR4252613_4869693___Sbg4
TACACAAGGACATGGAGGGCCTCCCTGGA
>SRR4252617_16085263___Sbg4
AGCCAAGACCTGTCGGGAGAGGATAG
>SRR4252611_1153211___Sbg4
TCCGAGACAACACGAACATATAGACAAAGA
>SRR4252610_3649309___Sbg4
CGAGACAACACGAACATATAGACAAAG
>SRR4252621_9355513___Sbg4
TCCGAGACAACACGAACATATAGACAAAG
>SRR4252607_10149193___Sbg4
GCTCAATGCCAGGGCCACCTCCGAGACAA
>SRR4252609_916873___Sbg4
TGAAGATGATCATGATGGCCAGATGATGT
>SRR4252607_1643817___Sbg4
TCCGAGACAACACGAACATATAGACAA
>SRR4252610_7414772___Sbg4
TGAAGATGATCATGATGGCCAGATGATGT
>SRR4252610_10995770___Sbg4
TCCCCCACTAGTAGGTACTATTTACATGA
>SRR4252605_15077863___Sbg4
TATTTACATGACTCACGCCACCCGGGCCT
>SRR4252605_24546___Sbg4
CCAAGACCTGTCGGGAGAGGATAGACTATGG
>SRR4252612_3847752___Sbg4
GCTCAATGCCAGGGCCACCTCCGAGACAA
>SRR4252613_9519235___Sbg4
CCGTTTGGGCCTAAAGTTTCCTGACAT
>SRR4252624_5390399___Sbg4
TACACAAGGACATGGAGGGCCTCCCTGGA
>SRR4252612_7992923___Sbg4
CCGAGACAACACGAACATATAGACAAAGA
>SRR4252611_12943350___Sbg4
TGGGCCTAAAGTTTCCTGACATTTGTCTT
>SRR4252605_3239109___Sbg4
CCGTTTGGGCCTAAAGTTTCCTGACATTT
>SRR4252608_8428213___Sbg4
TACACAAGGACATGGAGGGCCTCCCTGGAA
>SRR4252608_13207541___Sbg4
CCCACTAGTAGGTACTATTTACATGACT
>SRR4252611_11797988___Sbg4
CATGGAGGGCCTCCCTGGATAAGTGATCA
>SRR4252611_3627604___Sbg4
TGATGAAGATGATCATGATGGCCAGATG
>SRR4252610_13625200___Sbg4
TACACAAGGACATGGAGGGCCTCCCT
>SRR4252610_5253446___Sbg4
CATTCATACTTTTTTGACTACACAAGGA
>SRR4252605_14786234___Sbg4
GATGAAGATGATCATGATGGCCAGATGA
>SRR4252605_7353208___Sbg4
CCCCACTAGTAGGTACTATTTACATGACT
>SRR4252608_5566162___Sbg4
TACACAAGGACATGGAGGGCTTCCCTG
>SRR4252605_6842102___Sbg4
CCCACACTCATACCAACAAGGCGAAT
>SRR4252610_12614732___Sbg4
CATTCATACTTTTTTGACTACACAAGGA

>SRR4252626_3188750___Sbg4
TGAAGATGATCATGATGGCCAGATGATG
>SRR4252605_4367142___Sbg4
CCCCACTAGTAGGTACTATTTACATGACT
>SRR4252605_10564447___Sbg4
TACACAAGGACATGGAGGGCCTCCCTGGAT
>SRR4252610_4458582___Sbg4
TCCAAGTTTTACCTCCCCCACTAGTAGG
>SRR4252610_5233720___Sbg4
TTCCAAGTTTTACCTCCCCCACTAGTAG
>SRR4252612_5692188___Sbg4
TCCGAGACAACACGAACATATAGACAAAG
>SRR4252622_7880658___Sbg4
TGAAGATGATCATGATGGCCAGATGATGT
>SRR4252605_9779444___Sbg4
CCCCACTAGTAGGTACTATTTACATGACT
>SRR4252609_6440551___Sbg4
TCCGAGACAACACGAACATATAGACAA
>SRR4252611_8660017___Sbg4
ACCGTTTGGGCCTAAAGTTTCCTGACATT
>SRR4252619_2732629___Sbg4
CATTCATACTTTTTTGACTACACAAGGA
>SRR4252611_8030493___Sbg4
TGAAGATGATTATGATGGCCAGATGAT
>SRR4252611_2468243___Sbg4
GAGGATAGACTATGGATCACATATACAT
>SRR4252608_6478118___Sbg4
CTCCGAGACAACACGAACATATAGACAAA
>SRR4252610_5202070___Sbg4
TAGGTACTATTTACATGACTCACGCCACCT
>SRR4252623_12558601___Sbg4
AGACCTGTCGGGAGAGGATAGACTATGGA
>SRR4252607_11470958___Sbg4
ATTCATACTTTTTTGACTACACAAGGA
>SRR4252605_13426398___Sbg4
TCCGAGACAACACGAACATATAGACAAAT
>SRR4252625_3239636___Sbg4
CACATTCATACTTTTTTGACTACACAAGG
>SRR4252610_4381473___Sbg4
CCCCACTAGTAGGTACTATTTACATGACT
>SRR4252610_1610840___Sbg4
GCATTCATACTTTTTTGACTACACAAGGA
>SRR4252611_5387205___Sbg4
GCATTCATACTTTTTTGACTACACAAGGA
>SRR4252611_3862003___Sbg4
ATTCATACTTTTTTGACAACACAAGGA
>SRR4252610_5707004___Sbg4
CCCACTAGTAGGTACTATTTACATGACT
>SRR4252626_3670184___Sbg4
ACCGTTTGGGCCTAAAGTTTCCTGACATT
>SRR4252606_10115762___Sbg4
TGCCAGGGCCACCTCCGAGACAACACGA
>SRR4252611_6272079___Sbg4
ACCGTTTGGGCCTAAAGTTTCCTGACATT
>SRR4252608_7697341___Sbg4
CCTCCGAGACAACACGAACATATAGACA
>SRR4252610_5531652___Sbg4
TCCGAGACAACACGAACATATAGACAAA
>SRR4252607_6978540___Sbg4
TGATCATGATGGCCAGATGATGTCTGGGG
>SRR4252611_1839635___Sbg4
CCCACTAGTAGGTACTATTTACATGACT
>SRR4252609_2589277___Sbg4
TACTTTTTTGACTACACAAGGACATGG
>SRR4252610_9468136___Sbg4
GCTCAATGCCAGGGCCACCTCCGAGACAA
>SRR4252620_6079297___Sbg4
AAGTTTTACCTCCCCCACTAGTAGGTACT
>SRR4252620_9359976___Sbg4
TATTTACATGACTCACGCCACCCGGGCCA

>SRR4252609_3636316___Sbg4
TACCAACAAGGCGAATATGATGAAGATG
>SRR4252607_4801138___Sbg4
GGGACCGTTTGGGCCTAAAGTTTCCTGACA
>SRR4252609_4326453___Sbg4
CGTTTGGGCCTAAAGTTTCCTGACATTTG
>SRR4252622_7198927___Sbg4
CATTCATACTTTTTTGACTACACAAGGA
>SRR4252605_7501614___Sbg4
TGAAGATGATCATGATGGCCAGATGATGT
>SRR4252607_8344240___Sbg4
CATTCATACTTTTTTGACTACACAAGGA
>SRR4252618_4279995___Sbg4
TTTTTGACTACACAAGGACATGGAGGGC
>SRR4252610_2586235___Sbg4
TACACAAGGACATGGAGGGCTTCCCTGGA
>SRR4252610_891196___Sbg4
TTGGGCCTAAAGTTTCCTGACATTTGT
>SRR4252609_10588560___Sbg4
CATTCATACTTTTTTGACTACACAAGGA
>SRR4252621_9555637___Sbg4
TGGGCCTAAAGTTTCCTGACATTTGTCTTA
>SRR4252612_4762462___Sbg4
AGACCTGTCGGGAGAGGATAGACTATGA
>SRR4252605_14436205___Sbg4
CCCCACTAGTAGGTACTATTTACATGACT
>SRR4252610_8686445___Sbg4
CCCCACTAGTAGGTACTATTTACATGACT
>SRR4252612_233501___Sbg4
TACCAACAAGGCGAATATGATGAAGATG
>SRR4252624_10565023___Sbg4
GCTCAATGCCAGGGCCACCTCCGAGACAA
>SRR4252605_11815594___Sbg4
ACCGTTTGGGCCTAAAGTTTCCTGACA
>SRR4252612_1968370___Sbg4
TACACAAGGACATGGAGGGCCTCCCTGG
>SRR4252617_7114745___Sbg4
CTCCGAGACAACACGAACATATAGACA
>SRR4252607_8358646___Sbg4
TAGGTACTATTTACATGACTCACGCCATCT
>SRR4252619_1035834___Sbg4
GCCACCTCCGAGACAACACGAACATA
>SRR4252610_7048019___Sbg4
CCCACTAGTAGGTACTATTTACATGACT
>SRR4252608_9246050___Sbg4
TACACAAGGACATGGAGGGCCTCCCTGGA
>SRR4252608_11094932___Sbg4
CCCCACTAGTAGGTACTATTTACATGACT
>SRR4252608_519647___Sbg4
CCCCACTAGTAGGTACTATTTACATGACT
>SRR4252607_10111393___Sbg4
ACATTCATACCTTTTTGACTACACAAGGA
>SRR4252610_7433535___Sbg4
CCCCACTAGTAGGTACTATTTACATGACT
>SRR4252614_1197227___Sbg4
ATTCATACTTTTTTGACTACACAAGGA
>SRR4252609_5173226___Sbg4
CGAGACAACACGAACATATAGACAAAGA
>SRR4252609_9550643___Sbg4
GCTCAATGCCAGGGCCACCTCCGAGACAA
>SRR4252610_7265404___Sbg4
GCTCAATGCCAGGGCCACCTCCGAGACAA
>SRR4252610_11080185___Sbg4
CCAACAAGGCGAATATGATGAAGATGAT
>SRR4252612_83801___Sbg4
ACCGTTTGGGCCTAAAGTTTCCTGACATT
>SRR4252613_9203248___Sbg4
TTTCCAAGTTTTACCTCCCCCACTAGTAG
>SRR4252621_5193750___Sbg4
TACACAAGGACATGGAGGGCCTCCCTGGA

>SRR4252612_913120___Sbg4
GAGACAACACGAACATATAGACAAAGA
>SRR4252605_13023074___Sbg4
TCCGAGACAACACGAACATATAGACAAAG
>SRR4252609_3257837___Sbg4
TACACAAGGACATGGAGGGCCTCCCTGG
>SRR4252619_8705900___Sbg4
TATGATGAAGATGATCATGATGGCCAGAT
>SRR4252610_3685286___Sbg4
TCCGAGACAACACGAACATATAGACAAAG
>SRR4252610_2160573___Sbg4
TCCAAGTTTTACCTCCCCCACTAGTAGGT
>SRR4252610_8818760___Sbg4
CCCCACTAGTAGGTACTATTTACATGACT
>SRR4252608_4021429___Sbg4
CGGGCTTTACAGAGCCAAGACCTGTCGGG
>SRR4252606_1961718___Sbg4
CCTGTCGGGAGAGGATAGACTATGGATC
>SRR4252610_8399256___Sbg4
TCCGAGACAACACGAACATGTAGACAA
>SRR4252614_5511052___Sbg4
TCCGAGACAACACGAACATGTAGACAAAG
>SRR4252615_2268168___Sbg4
GGCCTAAAGTTTCCTGACATTTGTCTTAT
>SRR4252621_11018178___Sbg4
ATGAAGATGATCATGATGGCCAGATGA
>SRR4252610_8867259___Sbg4
CCCCACTAGTAGGTACTAATTACATGACT
>SRR4252605_6304767___Sbg4
CATTCATACTTTTTTGACTACACAAGGA
>SRR4252606_9862655___Sbg4
TCCGAGACAACACGAACATATAGACAAAG
>SRR4252607_3479056___Sbg4
TCCGAGACAACACGAACATATAGACAAAG
>SRR4252626_6638492___Sbg4
GCTCAATGCCAGGGCCACCTCCGAGACAA
>SRR4252605_12418579___Sbg4
ACCGTTTGGGCCTAAAGTTTCCTGACAT
>SRR4252616_6789957___Sbg4
TATAGACAAAGACACCATTCACACCCACA
>SRR4252610_9200529___Sbg4
TGGGCCTAAAGTTTCCTGACATTTGTCTT
>SRR4252607_10711807___Sbg4
GCTCAATGCCAGGGCCACCTCCGAGACAA
>SRR4252610_2722056___Sbg4
TACACAAGGACATGGAGGGCCTCCCTGGA
>SRR4252607_6827158___Sbg4
GCATTCATACTTTTTTGACTACACAAGGA
>SRR4252608_12094687___Sbg4
GCATTCATACTTTTTTGACTACACAAGGA
>SRR4252612_6028021___Sbg4
GCATTCATACTTTTTTGACTACACAAGGA
>SRR4252606_6432695___Sbg4
TGACTACACAAGGACATGTAGGGCCTCCC
>SRR4252605_2106733___Sbg4
GCCGGGCCTTACAGAGCCAAGACCTGTC
>SRR4252624_6649060___Sbg4
TATAGACAAAGACACCATTCACACCCACA
>SRR4252612_8030893___Sbg4
TACACAAGGACATGGAGGGCCTCCCTGGA
>SRR4252625_14600137___Sbg4
GACACCATTCACACCCACACTCATAGC
>SRR4252610_2918597___Sbg4
GCAACAAGGCGAATATGATGAAGATGAT
>SRR4252610_5969085___Sbg4
CCCCACTAGTAGGTACTATTTACATGACT
>SRR4252606_1171083___Sbg4
ACCTCCGAGACAACACGAACATATAGACAAA
>SRR4252616_14693410___Sbg4
CACCTCCGAGACAACACGAACATATAGA

```
>SRR4252605_5000147___Sbg4
CCCCACTAGTAGGTACTATTTACATGACT
>SRR4252626_6767682___Sbg4
TACACAAGGACATGGAGGGCCTCCCA
>SRR4252610_9249307___Sbg4
CCCACTAGTAGGTACTATTTACATGACTC
>SRR4252612_2789191___Sbg4
TACACAAGGACATGGAGGGCCTCCCT
>SRR4252610_9566989___Sbg4
CCCACTAGTAGGTACTATTTACATGACT
>SRR4252611_7176319___Sbg4
TATTTACATGACTCACGCCACCCGGGCCT
>SRR4252611_5480973___Sbg4
CACACATACATTCATACTTTTTTGACTACAC
>SRR4252620_3649905___Sbg4
TCCGAGACAACACGAACATATAGACAAA
>SRR4252608_12305013___Sbg4
TCCGAGACAACACGAACATATAGACAAAG
>SRR4252607_1293170___Sbg4
CCCACTAGTAGGTACTATTTACATGACT
>SRR4252608_398203___Sbg4
GCATTCATACTTTTTTGACTACACAAGGA
>SRR4252606_6409112___Sbg4
TACACAAGGACATGGAGGGCCTCCCTG
>SRR4252605_9380340___Sbg4
CCCCACTAGTAGGTACTATTTACATGACT
>SRR4252610_2196071___Sbg4
CCAAGACCTGTCGGGAGAGGATAGACTAT
>SRR4252624_2282889___Sbg4
TACACAAGGACATGGAGGGCCTCCCTGGA
>SRR4252610_8373161___Sbg4
ACGCCACCCGGGCCTTACAGAGCCAAGA
>SRR4252609_16779934___Sbg4
TACTTTTTTGACTACACAAGGACATGG
>SRR4252612_8696632___Sbg4
ATGATGGCCAGATGATGTCTGGGGAC
>SRR4252624_3466883___Sbg4
TGATCATGATGGCCAGATGATGTCTGGGG
>SRR4252611_15138449___Sbg4
TCGAGACAACACGAACATATAGACAAA
>SRR4252610_2567258___Sbg4
ACACATTCATACTTTTTTGACTACACAAGGA
>SRR4252620_7969827___Sbg4
AGGGCCACCTCCGAGACAACACGAACATA
>SRR4252615_6178536___Sbg4
GCTCAATGCCAGGGCCACCTCCGAGACAA
>SRR4252610_2543815___Sbg4
ACCGTTTGGGCCTAAAGTTTCCTGACATT
>SRR4252611_11203749___Sbg4
TACATTCATACTTTTTTGACTACACAAGGA
>SRR4252607_7437274___Sbg4
TATACATTCATACTTTTTTGACTACACAAGG
>SRR4252611_4087544___Sbg4
TACACAAGGACATGGAGGGCCTCCCT
>SRR4252609_1036224___Sbg4
CAATGCCAGGGCCACCTCCGAGACAACA
>SRR4252625_7562164___Sbg4
GGCCACCTCCGAGACAACACGAACATATA
>SRR4252610_11198554___Sbg4
TCCGAGACAACACGAACATATAGACAAAG
>SRR4252605_4209232___Sbg4
TACACAAGGACATGGAGGGCCTCCCTGG
>SRR4252606_2977284___Sbg4
ACCTCCGAGACAACACGAACATATAGACA
>SRR4252605_2639168___Sbg4
GCTCAATGCCAGGGCCACCTCCGAGACAAC
>SRR4252610_8171740___Sbg4
TTTGACTACACAAGGACATGGAGGGCCTC
>SRR4252610_2072930___Sbg4
CGCCACCCGGGCCTTACAGAGCCAAGACC
```

>SRR4252611_3859164___Sbg4
TACACAAGGACATGGAGGGCCTCCCTGGA
>SRR4252612_4908619___Sbg4
TAGACAAAGACACCATTCACACCCACA
>SRR4252610_12877452___Sbg4
CCCACTAGTAGGTACTATTTACATGACTC
>SRR4252611_2051716___Sbg4
TATACATTCATACTTTTTTGACTACACA
>SRR4252610_9297069___Sbg4
TCCAAGTTTTACCTCCCCCACTAGTAGGT
>SRR4252610_11041351___Sbg4
CCCACTAGTAGGTACTATTTACATGACT
>SRR4252605_3528851___Sbg4
CCCCACTAGTAGGTACTATTTACATGACT
>SRR4252608_6556689___Sbg4
CCCCACTAGTAGGTACTATTTACATGACT
>SRR4252622_3798100___Sbg4
ACCGTTTGGGCCTAAAGTTTCCTGACA
>SRR4252608_8696759___Sbg4
GCTCAATGCCAGGGCCACCTCCGAGACAA
>SRR4252607_9500799___Sbg4
GCTCAATGCCAGGGCCACCTCCGAGACAA
>SRR4252605_11429690___Sbg4
TGAAGATGATCATGATGGCCAGATGATGT
>SRR4252610_8252260___Sbg4
CCGAGACAACACGAACATATAGACAAAGA
>SRR4252610_4905212___Sbg4
CACCATTCACACCCACACTCATACCAACAAG
>SRR4252605_13803682___Sbg4
GCTCAATGCCAGGGCCACCTCCGAGACAA
>SRR4252608_8643018___Sbg4
TGATGAAGATGATTATGATGGCCAGATG
>SRR4252614_928405___Sbg4
AAGACCTGTCGGGAGAGGATAGACTATGGA
>SRR4252616_6136135___Sbg4
CGAGACAACACGAACATATAGACAAAGACA
>SRR4252612_7646119___Sbg4
TAGGTACTATTTACATGACTCACGCCACCC
>SRR4252609_9170715___Sbg4
TACACAAGGACATGGAGGGCCTCCCTGGA
>SRR4252612_7544421___Sbg4
CAAGACCTGTCGGGAGAGGATAGACTAT
>SRR4252605_10710095___Sbg4
GACACCATTCACACCCACACTCATACCAACA
>SRR4252612_813877___Sbg4
TCACGCCACCCGGGCCTTACAGAGCCAAGA
>SRR4252605_13745341___Sbg4
GCTCAATGCCAGGGCCACCTCCGAGACAA
>SRR4252611_5129457___Sbg4
CACATATACATTCATACTTTTTTGACTA
>SRR4252610_10190404___Sbg4
TTTTTGACTACACAAGGACATGGAGGGC
>SRR4252611_7840820___Sbg4
TACACAAGGACATGGAGGGCCTCCCT
>SRR4252613_9849784___Sbg4
TACACAAGGACATGGAGGGCCTCCCTGGATT
>SRR4252611_10159468___Sbg4
TACCAACAAGGCGAATATGATGAAGATG
>SRR4252611_3064793___Sbg4
TACATTCATACTTTTTTGACTAGACAAGG
>SRR4252611_2602524___Sbg4
TGAAGATGATCATGATGGCCAGATGATG
>SRR4252623_10288799___Sbg4
ACATTCATACTTTTTTGACTACACAAGGA
>SRR4252611_5880885___Sbg4
TACTTTTTTGACTACACAAGGACATGGA
>SRR4252612_3325284___Sbg4
GCTCAATGCCAGGGCCACCTCCGAGAC
>SRR4252606_218279___Sbg4
CCCACTAGTAGGTACTATTTACATGACTC

>SRR4252611_13034474___Sbg4
GATCACATATACATTCATACTTTTTTGACTA
>SRR4252605_5493787___Sbg4
TACTATTTACATGACTCACGCCACCAGG
>SRR4252609_8199726___Sbg4
TGAAGATGATCATGATGGCCAGATGATGT
>SRR4252611_1110670___Sbg4
CCAACAAGGCGAATATGATGAAGATGAT
>SRR4252610_7343388___Sbg4
TACTTTTTTGACTACACAAGGACATAG
>SRR4252606_5142882___Sbg4
TCTCAATGCCAGGGCCACCTCCGAGACAACA
>SRR4252607_7259873___Sbg4
TCTCAATGCCAGGGCCACCTCCGAGACAA
>SRR4252605_7338365___Sbg4
TCCAAGTTTTACCTCCCCCACTAGTAGGT
>SRR4252625_8276049___Sbg4
TTTTGACTACACAAGGACATGGAGGGCCT
>SRR4252625_4787229___Sbg4
TGGGCCTAAAGTTTCCTGACATTTGTCTTG
>SRR4252610_9260207___Sbg4
TACACAAGGACATGGAGGGCCTCCCT
>SRR4252607_10012348___Sbg4
TCCGAGACAACACGAACATATAGACAAGG
>SRR4252624_928986___Sbg4
TCCAAGTTTTACCTCCCCCACTAGTAGGT
>SRR4252613_5867073___Sbg4
ACGCCACCCGGGCCTTACAGAGCCAAGA
>SRR4252610_11526288___Sbg4
CCCACTAGTAGGTACTATTTACATGACT
>SRR4252609_11384196___Sbg4
TGCCAGGGCCACCTCCGAGACAACACGA
>SRR4252615_15076733___Sbg4
TTTGACTACACAAGGACATGGAGGGT
>SRR4252623_13180443___Sbg4
TACACAAGGACATGGAGGGCCTCCCTG
>SRR4252610_12447994___Sbg4
ACGAACATATAGACAAAGACACCATT
>SRR4252612_526183___Sbg4
CCCACTAGTAGGTACTATTTACATGACT
>SRR4252613_6110695___Sbg4
TACTATTTACATGACTCACGCCACCCGG
>SRR4252610_2668757___Sbg4
TACTTTTTTGACTACACAAGGACATGG
>SRR4252611_9443868___Sbg4
TAGTAGGTACTATTTACATGACTCACACC
>SRR4252608_5130410___Sbg4
GCTCAATGCCAGGGCCACCTCCGAGACAA
>SRR4252607_11435723___Sbg4
TGCCAGGGCCACCTCCGAGACAACACGA
>SRR4252607_3339648___Sbg4
TCCGAGACAACACGAACATATAGACAAGG
>SRR4252606_4244912___Sbg4
TGAAGATGATCATGATGGCCAGATGATGT
>SRR4252610_9574808___Sbg4
TACAGAGCCAAGACCTGTCGGGAGAGG
>SRR4252607_4247259___Sbg4
CCCCACTAGTAGGTACTATTTACATGACT
>SRR4252610_2414303___Sbg4
CCCACTAGTAGGTACTATTTACATGACT
>SRR4252609_5768060___Sbg4
GCTCAATGCCAGGGCCACCTCCGAGACAA
>SRR4252610_10248802___Sbg4
TGAAGATGATCATGATGGCCAGATGATG
>SRR4252621_9082976___Sbg4
TCCAAGTTTTACCTCCCCCACTAGTAGG
>SRR4252606_6710818___Sbg4
CAAGACCTGTCGGGAGAGGATAGACTAT
>SRR4252613_6478924___Sbg4
TCCGAGACAACACGAACATATTGACAAAG

>SRR4252610_12080543___Sbg4
TGGCGAATATGATGAAGATGATCATGATGG
>SRR4252611_12822827___Sbg4
TTCCAAGTTTTACCTCCCCCACTAGTAGG
>SRR4252611_6503586___Sbg4
CACGCCACCCGGGCCTTACAGAGCCAAGA
>SRR4252613_829287___Sbg4
TTTGACTACACAAGGACATGGAGGGCCTCC
>SRR4252610_13261576___Sbg4
CCCCACTAGTAGGTACTATTTACATGACT
>SRR4252624_1501417___Sbg4
CTTACAGAGCCAAGACCTGTCGGGAGAGGA
>SRR4252622_1547393___Sbg4
AGCCAAGACCTGTCGGGAGAGGATAGACT
>SRR4252605_9460057___Sbg4
GTCGGGAGAGGATAGACTATGGATCACA
>SRR4252605_12518615___Sbg4
TATTCATACTTTTTTGACTACACAAGGA
>SRR4252611_4607055___Sbg4
ACGCCACCCGGGCCTTACAGAGCCAAGA
>SRR4252607_3060386___Sbg4
TACACAAGGACATGGAGGGCCTCCCTGGA
>SRR4252610_8906206___Sbg4
TGAAGATGATCATGATGGCCAGATGATGC
>SRR4252606_8452958___Sbg4
CGAGACAACACGAACATATAGACAAA
>SRR4252611_3451875___Sbg4
CATTCATACTTTTTTGACTACACAAGGA
>SRR4252607_6122358___Sbg4
TACACAAGGACATGGAGGGCCTCCCT
>SRR4252613_8953812___Sbg4
TCCGAGACAACACGAACATATAGACAAAG
>SRR4252605_4284020___Sbg4
TGTCTTATGGGTGAGGAAAAACCCCAGA
>SRR4252611_7456015___Sbg4
TCCATTCCATTTTCCAAGTTTTACCTC
>SRR4252624_675978___Sbg4
TCACATATACATTCATACTTTTTTGACTGC
>SRR4252625_10424684___Sbg4
CCCACTAGTAGGTACTATTTACATGACTC
>SRR4252607_4500052___Sbg4
TGAAGATGATCATGATGGCCAGATGATGC
>SRR4252610_12338209___Sbg4
TGAAGATGATCATGATGGCCAGATGATGC
>SRR4252611_15354069___Sbg4
TGAAGATGATCATGATGGCCAGATGATGC
>SRR4252610_13433064___Sbg4
TACACAAGGACATGGAGGGCCTCCCTGG
>SRR4252607_10131110___Sbg4
TGAAGATGATCATGATGGCCAGATGATGC
>SRR4252608_2787285___Sbg4
CCCACTAGTAGGTACTATTTACATGACT
>SRR4252609_15359821___Sbg4
GCTCAATGCCAGGGCCACCTCCGAGACAA
>SRR4252610_9796427___Sbg4
TATAGACAAAGACACCATTCACACCCA
>SRR4252610_13101561___Sbg4
TCCGAGACAACACGAACATATAGACGAAG
>SRR4252605_12001602___Sbg4
TACTTTTTTGACTACACAAGGACATGGA
>SRR4252613_2757650___Sbg4
TACACAAGGACATGGAGGGCCTCCCTGGA
>SRR4252614_199600___Sbg4
CCCACTAGTAGGTACTATTTACATGAC
>SRR4252606_4654679___Sbg4
GCTCAATGCCAGGGCCACCTCCGAGAC
>SRR4252606_5918576___Sbg4
CACGAACATATAGACAAAGACACCATTC
>SRR4252621_13791870___Sbg4
TCCGAGACAACACGAACATATAGACAAAG

>SRR4252611_10988768___Sbg4
TGATGAAGATGATCATGATGGCCAGATG
>SRR4252610_4489494___Sbg4
GCTCAATGCCAGGGCCACCTCCGAGACA
>SRR4252618_8154444___Sbg4
TATTTACATGACTCACGCCACCCGGGCCTC
>SRR4252607_5560870___Sbg4
ACACTCATACTTTTTTGACTACACAAGGA
>SRR4252616_13231187___Sbg4
CCTCCGAGACAACACGAACATATAGACA
>SRR4252606_164641___Sbg4
TCCGAGACAACACGAACATATAGACAAGG
>SRR4252605_11981397___Sbg4
TACACAAGGACATGGAGGGCCTCCCTGGA
>SRR4252610_7987354___Sbg4
ACGAACATATAGACAAAGACACCATT
>SRR4252606_8887729___Sbg4
CTGTCGGGAGAGGATAGACTATGGAT
>SRR4252609_16101020___Sbg4
CATAGAGGGCCTCCCTGGATAAGTGATCA
>SRR4252611_13473937___Sbg4
TACATTCATACTTTTTTGACTACTCAAGG
>SRR4252607_6004136___Sbg4
TCCGAGACAACACGAACATATAGACAAAGA
>SRR4252621_11054822___Sbg4
CACCATTCACACCCACACTCATACCAACAAG
>SRR4252611_1056815___Sbg4
ATTTACATGACTCACGCCACCCGGGCCTT
>SRR4252605_8831815___Sbg4
CATATACATGCATACTTTTTTGACTACACA
>SRR4252605_13100586___Sbg4
GCTCAATGACAGGGCCACCTCCGAGACAA
>SRR4252621_5110346___Sbg4
CCCCACTAGTAGGTACTATTTACATGACT
>SRR4252610_299807___Sbg4
CCCACTAGTAGGTACTATTTACATGACT
>SRR4252605_2520567___Sbg4
TAGGTACTATTTACATGACTCACGCCACCC
>SRR4252607_14005269___Sbg4
TCCGAGACAACACGAACATATAGACAAAG
>SRR4252611_2642909___Sbg4
CACGCCACCCGGGCCTTACAGAGCCAAGA
>SRR4252610_703945___Sbg4
CCCCACTAGTAGGTACTATTTACATGACT
>SRR4252609_12737517___Sbg4
GCTCAATGCCAGGGCCACCTCCGAGACAA
>SRR4252610_3276973___Sbg4
CCCACTAGTAGGTACTATTTACATGACT
>SRR4252607_7603927___Sbg4
ACATTCATACTTTTTTGACTACACAAGGA
>SRR4252610_2250698___Sbg4
ATTCATACTTTTTTGACTACACAAGGA
>SRR4252612_4964817___Sbg4
TCCGAGACAACACGAACATATAGACAAA
>SRR4252609_12745800___Sbg4
CCCACTAGTAGGTACTATTTACATGACT
>SRR4252614_2345109___Sbg4
AGACATCATTCACACCCACACTCATACCA
>SRR4252605_13041141___Sbg4
CCCACTAGTAGGTACTATTTACATGACT
>SRR4252605_8530805___Sbg4
TGAAGATGATCATGATGGCCAGATGATG
>SRR4252611_6269798___Sbg4
TGATCATGATGGCCAGATGATATCTGGG
>SRR4252605_8042964___Sbg4
TCCGAGACAACACGAACATATAGACAAGG
>SRR4252612_3653076___Sbg4
CTCCGAGACAACACGAACATATAGACAAA
>SRR4252611_8655900___Sbg4
TGAAGATGATCATGATGGCCAGATGATGC

>SRR4252610_10827815___Sbg4
ACGCCACCCGGGCCTTACAGAGCCAA
>SRR4252612_2834389___Sbg4
AACCGTTTGGGCCTAAAGTTTCCTGACAT
>SRR4252611_10854658___Sbg4
TCACATATACATTCATACTTTTTTGACT
>SRR4252612_6034659___Sbg4
ACCTCCGAGACAACACGAACATATAGACA
>SRR4252609_9097966___Sbg4
ATGAAGATGATCATGATGGCCAGATG
>SRR4252607_14848878___Sbg4
TGAAGATGATCATGATGGCCAGATGATGT
>SRR4252610_4694929___Sbg4
TACTTTTTTGACTACACAAGGACATGA
>SRR4252605_13218674___Sbg4
TCCGAGACAACACGAACATATAGACAAAG
>SRR4252606_2953589___Sbg4
ACCTGTCGGGAGAGGATAGACTATGGATC
>SRR4252608_6165476___Sbg4
TATAGACAAAGACACCATTCACACCCACA
>SRR4252605_7624783___Sbg4
TGAAGATGATCATGATGGCCAGATGATGT
>SRR4252612_6568985___Sbg4
TCCGAGACAACACGAACATATAGACAAAG
>SRR4252610_2441345___Sbg4
CCCACTAGTAGGTACTATTTACATGACT
>SRR4252605_5064916___Sbg4
TGATCAGCTCAATGCCAGGGCCACCTCCGA
>SRR4252605_10402251___Sbg4
TCCGAGACAACACGAACATATAGACAAAG
>SRR4252612_3223472___Sbg4
CTCCGAGACAACACGAACATATAGACAAA
>SRR4252606_595467___Sbg4
CCCCACTAGTAGGTACTATTTACATGACC
>SRR4252610_6039278___Sbg4
CCACTAGTAGGTACTATTTACATGACC
>SRR4252623_3458820___Sbg4
TACACAAGGACATGGAGGGCCTCCCT
>SRR4252614_2444250___Sbg4
TTTTTGACTACACAAGGACATGGAGGGCT
>SRR4252607_9365332___Sbg4
ATACATTCATACTTTTTTGACTACACAAGGA
>SRR4252605_2093212___Sbg4
ACTCAATGCCAGGGCCACCTCCGAGACAA
>SRR4252606_2397112___Sbg4
ACTCAATGCCAGGGCCACCTCCGAGACAA
>SRR4252606_3495981___Sbg4
ACTCAATGCCAGGGCCACCTCCGAGACAA
>SRR4252606_879989___Sbg4
ACTCAATGCCAGGGCCACCTCCGAGACAA
>SRR4252611_7022339___Sbg4
TGATGAAGATGATCATGATGGCCAGATG
>SRR4252612_3706965___Sbg4
ACCGTTTGGGCCTAAAGTTTCCTGACATT
>SRR4252608_9364644___Sbg4
ACCTCCGAGACAACACGAACATATAGACA
>SRR4252610_1189787___Sbg4
ACCAACAAGGCGAATATGATGAAGATGA
>SRR4252612_4386001___Sbg4
TACATTCATACTTTTTTGACTAGACAAG
>SRR4252611_12070024___Sbg4
TGAAGATGATCATGATGGCCAGATGATG
>SRR4252609_344032___Sbg4
CCATTTTCCAAGTTTTACCTCCCCCACTA
>SRR4252607_5010182___Sbg4
CACATATACATTCATACTTTTTTGACTA
>SRR4252607_11807105___Sbg4
TGAAGATGATCATGATGGCCAGATGATGT
>SRR4252606_2987027___Sbg4
CCTGTCGGGAGAGGATAGACTATGGATC

>SRR4252619_14680898___Sbg4
ATTCACACTCACACTCATACCAACAAGGCGA
>SRR4252609_6106686___Sbg4
CCCACTAGTAGGTACTATTTACATGACT
>SRR4252610_12486321___Sbg4
TCCGAGACAACACGAACATATAGACAAAG
>SRR4252623_1312424___Sbg4
TGATGAAGATGATCATGATGGCCAGATG
>SRR4252623_6394976___Sbg4
TACTTTTTTGACTACACAAGGACATGG
>SRR4252610_4465412___Sbg4
TACACAAGGACATGGAGGGCCTCCCT
>SRR4252606_6919983___Sbg4
TACTTTTTTGACTACACAAGGACATGGA
>SRR4252611_1287668___Sbg4
TACACAAGGACATGGAGGGCCTCCCTGGA
>SRR4252605_1152705___Sbg4
GACACCATTCACACCCACACTCATACCAAC
>SRR4252605_5274360___Sbg4
CACCATTCACACCCACACTCATACCAACAAG
>SRR4252612_3051402___Sbg4
CCCCACTAGTAGGTACTATTTACATGACT
>SRR4252612_6614418___Sbg4
TACATTCATACTTTTTTGACTAGACA
>SRR4252612_7287319___Sbg4
ACCGTTTGGGCCTAAAGTTTCCTGACATT
>SRR4252619_1260054___Sbg4
TAGACTATGGATCACATATACATTCAT
>SRR4252607_14916119___Sbg4
CCGAGACAACACGAACATATAGACAAAGAC
>SRR4252606_6383604___Sbg4
ACATTCATACTTTTTTGACTACACAAGGA
>SRR4252607_14011183___Sbg4
CACCATTTACACCCACACTCATACCAACAAG
>SRR4252605_13355837___Sbg4
CCAAGTTTTACCTCCCCCACTAGTTGGTA
>SRR4252605_12730954___Sbg4
TACACAAGGACATGGAGGGCCTCCCT
>SRR4252614_7783441___Sbg4
TACACAAGGACATGGAGGGCCTCCCTGGT
>SRR4252624_4425411___Sbg4
GCTCAATGCCAGGGCCACCTCCGAGACAA
>SRR4252617_4032594___Sbg4
TCCGAGACAACACGAACATATAGACAAAGA
>SRR4252611_13540512___Sbg4
CCGTTTGGGCCTAAAGTTTCCTGACATT
>SRR4252606_1245254___Sbg4
CCTGTCGGGAGAGGATAGACTATGGATC
>SRR4252611_13502767___Sbg4
TGACTACACAAGGACATGGAGGGCCTCCCT
>SRR4252616_3927045___Sbg4
AGGACATGGAGGGCCTCCCTGGATAAGTG
>SRR4252621_1077822___Sbg4
TCCGAGACAACACGAACATATAGACAAAG
>SRR4252610_9498298___Sbg4
AACAAGGCGAATATGATGAAGATGAT
>SRR4252611_12122788___Sbg4
TGAAGATGATCATGATGGCCAGATGATGT
>SRR4252606_8375921___Sbg4
TGAAGATGATCATGATGGCCAGATGATG
>SRR4252614_6037230___Sbg4
TCGTTTGGGCCTAAAGTTTCCTGACAT
>SRR4252614_559288___Sbg4
ACCTCCGAGACAACACGAACATATAG
>SRR4252610_13436299___Sbg4
TACACAAGGACATGGAGGGCCTCCCTGGA
>SRR4252610_12944926___Sbg4
CCCCACTAGTAGGTACTATTTACATGACT
>SRR4252605_3286765___Sbg4
GCTCAATGCCAGGGCCACCTCCGAGACAA

>SRR4252610_10341526___Sbg4
CACCATTCACACCCACACTCATACCAACAAG
>SRR4252611_7215518___Sbg4
TGAAGATGATCATGATGGCCAGATGATGT
>SRR4252608_9774196___Sbg4
CCCACTAGTAGGTACTATTTACATGACT
>SRR4252611_6313676___Sbg4
ACTCCGAGACAACACGAACATATAGA
>SRR4252610_11143175___Sbg4
CCCCACTAGTAGGTACTATTTACATGACT
>SRR4252621_958809___Sbg4
TCCGAGACAACACGAACATATAGACAAAG
>SRR4252617_399150___Sbg4
TCCAAGTTTTACCTCCCCCACTAGTAGG
>SRR4252611_11254024___Sbg4
TACTTTTTTGACTACACAAGGACATGG
>SRR4252606_5020592___Sbg4
GCTCAATGCCAGGGCCACCTCCGAGACAACA
>SRR4252624_7004739___Sbg4
TCCGAGACAACACGAACATATAGACAAAGA
>SRR4252610_6280511___Sbg4
CCCACTAGTAGGTACTATTTACATGACT
>SRR4252606_3165808___Sbg4
TTCCAAGTTTTACCTCCCCCACTAGTA
>SRR4252605_7472757___Sbg4
TCCGAGACAACACGAACATATAGACAAAG
>SRR4252610_3230737___Sbg4
ACCGTTTGGGCCTAAAGTTTCCTGACATT
>SRR4252605_12747656___Sbg4
TCCAAGTTTTACCTCCCCCACTAGTAGGT
>SRR4252611_12683354___Sbg4
TATACATTCATACTTTTTTGACTACACA
>SRR4252614_9637579___Sbg4
CGAACATATAGACAAAGACACCATTC
>SRR4252625_13534296___Sbg4
ACCGTTTGGGCCTAAAGTTTCCTGACATT
>SRR4252613_503721___Sbg4
TCAATGCCAGGGCCACCTCCGAGACAAC
>SRR4252623_14625422___Sbg4
TCCAAGTTTTACCTCCCCCACTAGTAGGT
>SRR4252609_12368426___Sbg4
CTCCGAGACAACACGAACATATAGACAAA
>SRR4252613_8381363___Sbg4
CCGTTTGGGCCTAAAGTTTCCTGACATTT
>SRR4252605_13053159___Sbg4
CCCCACTAGTAGGTACTATTTACATGACT
>SRR4252605_11262051___Sbg4
CCCCACTAGTAGGTACTATTTACATGACT
>SRR4252623_608472___Sbg4
CCCCACTAGTAGGTACTATTTACATGACT
>SRR4252608_1771981___Sbg4
CCCACTAGTAGGTACTATTTACATGACT
>SRR4252624_9194947___Sbg4
GCTCAATGCCAGGGCCACCTCCGAGACAA
>SRR4252610_9357673___Sbg4
TCAGCTCAATGCCAGGGCCACCTCCGAGA
>SRR4252608_10925693___Sbg4
CTCCGAGACAACACGAACATATAGACAAA
>SRR4252608_11003470___Sbg4
CCCACTAGTAGGTACTATTTACATGACT
>SRR4252607_41408___Sbg4
AAGACCTGTCGGGAGAGGATAGACTATG
>SRR4252611_11831194___Sbg4
CCCACTAGTAGGTACTAATTACATGACT
>SRR4252610_2114248___Sbg4
CCCACTAGTAGGTACTATTTACATGACT
>SRR4252621_13484618___Sbg4
TCCGAGACAACACGAACATATAGACAAAG
>SRR4252621_11255358___Sbg4
TCCGAGACAACACGAACATATAGACAA

```
>SRR4252610_2559642___Sbg4
TCCGAGACAACACGAACATATAGACAAGG
>SRR4252609_4386854___Sbg4
CGTTTGGGCCTAAAGTTTCCTGACATTTG
>SRR4252612_1816715___Sbg4
TATTTACATGACTCACGCCACCCGGGCCT
>SRR4252610_13078843___Sbg4
TGATGAAGATGATCATGATGGCCAGATG
>SRR4252607_8037922___Sbg4
GCTCAATGCCAGGGCCACCTCCGAGACAA
>SRR4252607_6766238___Sbg4
CACCATTCACACCCACACTCATACCAAC
>SRR4252605_7197207___Sbg4
GCTCAATGCCAGGGCCACCTCCGAGACA
>SRR4252611_14620500___Sbg4
TTTGTCTTATGGGTGAGGAAAAACCCCAG
>SRR4252612_3938963___Sbg4
TACAGAGCCAAGACCTGTCGGGAGAGGAT
>SRR4252615_7089270___Sbg4
CATTCATACTTTTTTGACTACACAAGGACAT
>SRR4252608_11076212___Sbg4
TGAAGATGATCATGATGGCCAGATGATG
>SRR4252611_1125209___Sbg4
TGGGCCTAAAGTTTCCTGACATTTGTCTTA
>SRR4252624_3110318___Sbg4
TCATACCAACAAGGCGAATATGATGAAG
>SRR4252610_11349545___Sbg4
CCCCACTAGTAGGTACTATTTACATGACT
>SRR4252608_1346104___Sbg4
CCCCACTAGTAGGTACTATTTACATGACC
>SRR4252610_10458824___Sbg4
CCCCACTAGTAGGTACTATTTACATGACC
>SRR4252610_2580286___Sbg4
CCCCACTAGTAGGTACTATTTACATGACC
>SRR4252612_708761___Sbg4
CCCCACTAGTAGGTACTATTTACATGACC
>SRR4252614_1515074___Sbg4
TCCGAGACAACACGAACATATAGACAAAGA
>SRR4252612_2453877___Sbg4
TCCAAGTTTTACCTCCCCCACTAGTAGG
>SRR4252609_12953112___Sbg4
ATAGACAAAGACACCATTCACACCCACAC
>SRR4252608_13088905___Sbg4
TACATTCATACTTTTTTGACTACACAAGGA
>SRR4252608_12736146___Sbg4
CCCCACTAGTAGGTACTATTTACATGACT
>SRR4252607_8811925___Sbg4
TCACATATACATTCATACTTTTTTGACA
>SRR4252614_397246___Sbg4
TCCGAGACAACACGAACATATAGACA
>SRR4252607_14616020___Sbg4
TGAAGATGATCATGATGGCCAGATGATGT
>SRR4252610_16556___Sbg4
TCCTGACATTTGTCTTATGGGAGAGG
>SRR4252608_4624755___Sbg4
TCCGAGACAACACGAACATATAGACGAAG
>SRR4252612_6333271___Sbg4
GAGGATAGACTATGGATCACATATACAT
>SRR4252616_8422945___Sbg4
TCCAAGTTTTACCTCCCCCACTAGTAGG
>SRR4252617_2795305___Sbg4
ACCTCCGAGACAACACGAACATATAGACA
>SRR4252609_11561013___Sbg4
GAGACAACACGAACATATAGACAAAGA
>SRR4252621_7678382___Sbg4
GACCTGTCGGGAGAGGATAGACTATGGA
>SRR4252610_7132967___Sbg4
TGAAGATGATCATGATGGCCAGATGATGT
>SRR4252606_7799922___Sbg4
ACCGTTTGGGCCTAAAGTTTCCTGACA
```

>SRR4252611_10353287___Sbg4
TGAAGATGATCATGATGGCCAGATGATG
>SRR4252610_1986317___Sbg4
CCCCACTAGTAGGTACTATTTACATGACT
>SRR4252605_14737346___Sbg4
TAGGTACTATTTACATGACTCACGCCGCCC
>SRR4252610_926758___Sbg4
ACATTCATACTTTTTTGACTACACAAGGA
>SRR4252610_6330409___Sbg4
TGAAGATGATCATGATGGCCAGATGATGA
>SRR4252611_13229287___Sbg4
TACTTTTTTGACTACACAAGGACATGG
>SRR4252607_2371118___Sbg4
ACATATACATTCATACTTTTTTGACTACA
>SRR4252606_6866132___Sbg4
TCCGAGACAACACGAACATATAGACAAAG
>SRR4252610_9479825___Sbg4
CCGAGACAACACGAACATATAGACAAAG
>SRR4252605_6222320___Sbg4
CTGTCGGGAGAGGATAGACTATGGATCACAT
>SRR4252609_13603837___Sbg4
GCTCAATGCCAGGGCCACCTCCGAGACAA
>SRR4252619_7007193___Sbg4
CACGCCACCCGGGCCTTACAGAGCCAAGA
>SRR4252617_15704692___Sbg4
AACACGAACATATAGACAAAGACACCATT
>SRR4252607_6064744___Sbg4
ACTCATACCAACAAGGCGAATATGATGAAG
>SRR4252609_4781806___Sbg4
CATTCATACTTTTTTGACTACACAAGGA
>SRR4252610_1319940___Sbg4
CCCCACTAGTAGGTACTATTTACATGACC
>SRR4252610_4898170___Sbg4
CCCCACTAGTAGGTACTATTTACATGACC
>SRR4252608_1574251___Sbg4
CCCACTAGTAGGTACTATTTACATGACC
>SRR4252610_2277295___Sbg4
CCCACTAGTAGGTACTATTTACATGACC
>SRR4252612_3853870___Sbg4
CCCACTAGTAGGTACTATTTACATGACC
>SRR4252606_4523395___Sbg4
TGACTACACAAGGACATGGAGGGCCTCCC
>SRR4252610_9153524___Sbg4
CCCACTAGTAGGTACTATTTACATGACT
>SRR4252621_8356266___Sbg4
CCCACTAGTAGGTACTATATACATGACT
>SRR4252605_9812181___Sbg4
ACATTCAAACTTTTTTGACTACACAAGGA
>SRR4252608_11788418___Sbg4
GCATATAGACAAAGACACCATTCACACCCA
>SRR4252605_3853874___Sbg4
ACCAGGGCCACCTCCGAGACAACACGAACA
>SRR4252605_197041___Sbg4
ATGATGAAGATGATCATGATGGCCAGATGAT
>SRR4252609_2084277___Sbg4
CATTCATACTTTTTTGACTACACAAGGA
>SRR4252610_2299956___Sbg4
TACACAAGGACATGGAGGGCCTCTCT
>SRR4252611_11856146___Sbg4
ACAAGGACATGGAGGGCCTCTCTGGATA
>SRR4252612_2147005___Sbg4
TGAAGATGATCATGATGGCCAGATGATGT
>SRR4252605_1620500___Sbg4
ATTCATACTTTTTTGACTACACAAGGA
>SRR4252607_9291700___Sbg4
CATTCAAACTTTTTTGACTACACAAGGA
>SRR4252612_5906151___Sbg4
TGATGAAGATGATCATGATGGCTAGATG
>SRR4252609_8231873___Sbg4
TGAAGATGATCATGATGGCTAGATGATGT

>SRR4252610_10783018___Sbg4
TGAAGATGATCATGATGGCTAGATGATGT
>SRR4252611_14610983___Sbg4
TGAAGATGATCATGATGGCTAGATGATGT
>SRR4252612_3953129___Sbg4
TGAAGATGATCATGATGGCTAGATGATG
>SRR4252612_4807179___Sbg4
TGAAGATGATCATGATGGCTAGATGATGT
>SRR4252624_6142798___Sbg4
TGAAGATGATCATGATGGCTAGATGATGT
>SRR4252606_6472876___Sbg4
ACATTCATACTTTTTTGACTACACAAGGA
>SRR4252610_12689501___Sbg4
TGAAGATGATCATGATGGCCAGATGATGT
>SRR4252610_936459___Sbg4
CCCACTAGTAGGTACTATTTACATGACT
>SRR4252611_7449206___Sbg4
CACGCCACCCGGGCCTTACAGAGCCA
>SRR4252610_9209930___Sbg4
CCCACTAGTAGGTACTATTTACATGACT
>SRR4252611_14939735___Sbg4
TGAAGATGATCATGATGGCCAGATGATGT
>SRR4252617_9544059___Sbg4
TGGGCCTAAAGTTTCCTGACATTTGTCTTAT
>SRR4252610_2906955___Sbg4
CACGCCACCCGGGCCTTACAGAGCCAAGA
>SRR4252611_2543613___Sbg4
ACGCCACCCGGGCCTTACAGAGCCAAGA
>SRR4252611_15078511___Sbg4
GGGACCGTTTGGGCCTAAAGTTTCCTGACAT
>SRR4252614_234517___Sbg4
CCCACTAGTAGGTACTATTTACATGACT
>SRR4252611_13301627___Sbg4
GGATCACATATACATTCATACTTTTTTGAC
>SRR4252610_426868___Sbg4
TACACAAGGACATGGAGGGCCTCCCT
>SRR4252610_3501563___Sbg4
CCCCACTAGTAGGTACTATTTACATGACT
>SRR4252624_9289010___Sbg4
TGAAGATGATCATGATGGCCAGATGATGT
>SRR4252610_7774225___Sbg4
TATAGACAAAGACACCATTCACACCCACAC
>SRR4252612_1129343___Sbg4
ACCTGTCGGGAGAGGATAGACTATGGATC
>SRR4252617_10753585___Sbg4
CCTCCGAGACAACACGAACATATAGACA
>SRR4252605_11525828___Sbg4
TCCGAGACAACACGAACATATAGACA
>SRR4252613_3260746___Sbg4
CGAGACAACACGAACATATAGACAAAGA
>SRR4252624_5216618___Sbg4
TATGATGAAGATGATTATGATGGCCAGAT
>SRR4252606_10298638___Sbg4
CCCACTAGTAGGTACTATTTACATGACT
>SRR4252610_2101043___Sbg4
CCCCACTAGTAGGTACTATTTACATGACT
>SRR4252609_62716___Sbg4
CCCACTAGTAGGTACTATTTACATGACT
>SRR4252610_1199249___Sbg4
TGCCAGGGCCACCTCCGAGACAACACGAA
>SRR4252610_11367673___Sbg4
TCCGAGACAACACGAACATATAGACCAA
>SRR4252611_1874372___Sbg4
TCCGAGACAACACGAACATATAGACGAAG
>SRR4252614_2579969___Sbg4
TCCAAGACAACACGAACATATAGACAAA
>SRR4252605_14388300___Sbg4
GGGACCGTTTGGGCCTAAAGTTTCCTGACAT
>SRR4252606_2181072___Sbg4
CCCCACTAGTAGGTACTATTTACATGACT

>SRR4252610_7786025___Sbg4
TACACAAGGACATGGAGGGCCTCCCTG
>SRR4252614_5221848___Sbg4
ACAAAGACACCATTCACACCCACACTCA
>SRR4252605_6781096___Sbg4
TGATGAAGATGATCATGATGGCCATATGA
>SRR4252611_13232184___Sbg4
TGAAGATGATCATGATGGCCATATGATG
>SRR4252605_6248061___Sbg4
TCCCCCACTAGTAGGTACTATTTACATGAT
>SRR4252616_12215496___Sbg4
ACCTCCGAGACAACACGAACATATAGA
>SRR4252625_10024494___Sbg4
GATCAATGCCAGGGCCACCTCCGAGACAA
>SRR4252610_2330509___Sbg4
CCCACTAGTAGGTACTATTTACATGACT
>SRR4252611_8023696___Sbg4
GCATTCATACTTTTTTGACTACACAAGGA
>SRR4252611_2591181___Sbg4
TTTTTGACTACACAAGGACATGGAGGG
>SRR4252618_3758___Sbg4
ACCTCCGAGACAACACGAACATATAGA
>SRR4252610_8000172___Sbg4
TGACTACACAAGGACATGGAGGGCCTCT
>SRR4252616_2577875___Sbg4
ACTCAATGCCAGGGCCACCTCCGAGA
>SRR4252605_12553403___Sbg4
CCCCACTAGTAGGTACTATTTACATGACT
>SRR4252612_4547005___Sbg4
TACTATTTACATGACTCACGCCACTCGG
>SRR4252610_11734564___Sbg4
TACACAAGGACATGGAGGGCCTCCCTGG
>SRR4252605_5725004___Sbg4
TGAAGATGATCATGATGGCCAGATGATGT
>SRR4252611_5743142___Sbg4
ACTAGTAGGTACTATTTACATGACTCAC
>SRR4252607_8346059___Sbg4
CCGTTTGGGCCTAAAGTTTCCTGACATT
>SRR4252607_774487___Sbg4
CCCCACTAGTAGGTACTATTTACATGACTT
>SRR4252611_12441089___Sbg4
TGAAGATGATCATGATGGCCAGATGATGT
>SRR4252611_8878981___Sbg4
TGAAGATGATCATGATGGCCAGATGATGT
>SRR4252610_342166___Sbg4
TGAAGATGATCATGATGGCCAGATGATGT
>SRR4252611_14317842___Sbg4
GGGACCGTTTGGGCCTAAAGTTTCCTGACA
>SRR4252606_7157219___Sbg4
TGATGAAGATGATCATGATGGCCAGATG
>SRR4252611_12171948___Sbg4
ACCGTTTGGGCCTAAAGTTTCCTGACATT
>SRR4252613_4380031___Sbg4
TAGCCAAGACCTGTCGGGAGAGGATA
>SRR4252605_12596877___Sbg4
CCCACTAGTAGGTACTATTTACATGACTC
>SRR4252609_13167719___Sbg4
CATACTTTTTTGACTACACAAGGACA
>SRR4252605_8186571___Sbg4
TGAAGATGATCATGATGGCCAGATGATGT
>SRR4252610_53246___Sbg4
TGAAGATGATCATGATGGCCAGATGATGT
>SRR4252611_6807954___Sbg4
TCCGAGACAACACGAACATATAGACAAAG
>SRR4252605_14190378___Sbg4
CACCATTCACACCCACACTCATACCAACAAG
>SRR4252607_5027082___Sbg4
TGAAGATGATCATGATGGCCAGATGATGT
>SRR4252620_8563033___Sbg4
TTCCAAGTTTTACCTCCCCCACTAGTAG

>SRR4252611_9729983___Sbg4
TGAAGATGATCATGATGGCCAGATGATG
>SRR4252610_3193296___Sbg4
TGACTACACAAGGACATGGAGGGCCTCCC
>SRR4252612_2263455___Sbg4
TGAAGATGATCATGATGGCCAGATGATGT
>SRR4252612_7319281___Sbg4
ACCGTTTGGGCCTAAAGTTTCCTGACATT
>SRR4252610_13344351___Sbg4
CCCCACTAGTAGGTACTATTTACATGACT
>SRR4252610_6113043___Sbg4
TACCAACAAGGCGAATATGATGAAGATG
>SRR4252610_11845816___Sbg4
CCCCACTAGTAGGTACTATTTACATGACT
>SRR4252610_9184489___Sbg4
TATAGACAAAGACACCATTCACACCCACA
>SRR4252612_571506___Sbg4
TAAACAAGGCGAATATGATGAAGATGAT
>SRR4252623_6252943___Sbg4
TCACGCCACCCGGGCCTTACAGAGCCAAGA
>SRR4252609_10410413___Sbg4
GCTCAATGCCAGGGCCACCTCCGAGACAA
>SRR4252610_12679433___Sbg4
TCCAAGTTTTACCTCCCCCACTAGTAGC
>SRR4252610_12028558___Sbg4
TATACATTCATACTTTTTTGACTACAC
>SRR4252610_1028300___Sbg4
CCCCACTAGTAGGTACTATTTACATGACT
>SRR4252610_13384658___Sbg4
CGAGACAACACGAACATATAGACAAA
>SRR4252609_1287624___Sbg4
TATAGACAAAGACACCATTCACACCCAC
>SRR4252625_11934050___Sbg4
CCCACTAGTAGGTACTATTTACATGACT
>SRR4252611_2563999___Sbg4
TTTACATGACTCACGCCACCCGGGCCTT
>SRR4252621_8312423___Sbg4
CCCACTAGTAGGTACTATATACATGACT
>SRR4252612_1626885___Sbg4
TACACAAGGACATGGAGGGCCTCCCTGGA
>SRR4252610_11624841___Sbg4
CCCACTAGTAGGTACTATTTACATGACT
>SRR4252625_3877637___Sbg4
TGATGAAGATGATCATGATGGCCAGATGA
>SRR4252610_8780647___Sbg4
CCCCACTAGTAGGTACTATTTACATGACT
>SRR4252608_918920___Sbg4
TTTGACTACACAAGGACATGGAGGGACTC
>SRR4252605_2030601___Sbg4
ACCTCAGAGACAACACGAACATATAGACAAA
>SRR4252612_6643399___Sbg4
CCTACAAGGCGAATATGATGAAGATGAT
>SRR4252626_694270___Sbg4
TGAAGATGATCATGATGGCCAGATGATGT
>SRR4252607_12207407___Sbg4
GCTCAATGCCAGGGCCACCTCCGAGACAA
>SRR4252612_6168915___Sbg4
TCCGAGACAACACGAACATATAGACAAAG
>SRR4252607_8119802___Sbg4
CCCACTAGTAGGTACTATTTACATGACT
>SRR4252610_9746437___Sbg4
TCCAAGTTTTACCTCCCCCACTAGTAGGT
>SRR4252610_6801692___Sbg4
GCTCAATGCCAGGGCCACCTCCGAGACAA
>SRR4252610_6127579___Sbg4
CCGAGACAACACGAACATATAGACAAAGA
>SRR4252606_9302605___Sbg4
TACCAACAAGGCGAATATGATGAAGATG
>SRR4252610_8597462___Sbg4
CATATACATTCATACTTTTTTGACTACACA

```
>SRR4252611_14595987___Sbg4
ACATTCATACTTTTTTGACTACACAAGGA
>SRR4252611_9085224___Sbg4
TAGGTACTATTTACATGACTCACGCCACCT
>SRR4252624_6425427___Sbg4
GAGGATAGACTATGGATCACATATACAT
>SRR4252624_5814838___Sbg4
TACACAAGGACATGGAGGGCCTCCCTGG
>SRR4252605_14593137___Sbg4
TCCGAGACAACACGAACATATAGACAAA
>SRR4252605_6298013___Sbg4
GGCCTAAAGTTTCCTGACATTTGTCTTATG
>SRR4252608_3788082___Sbg4
CCGTTTGGGCCTAAAGTTTCCTGACA
>SRR4252605_172697___Sbg4
TTTTGACTACACAAGGACATGAAGGG
>SRR4252611_4037860___Sbg4
CATTCATACTTTTTTGACTACACAAGGA
>SRR4252611_3584842___Sbg4
TGAAGATGATCATGATGGCCAGATGATGC
>SRR4252607_11917566___Sbg4
TTCCGAGACAACACGAACATATAGACAAA
>SRR4252612_8156950___Sbg4
TGAAGATGATCATGATGGCCAGATGATGT
>SRR4252605_2639271___Sbg4
TGAAGATGATCATGATGGCCAGATGATGT
>SRR4252610_10976422___Sbg4
TACACAAGGACATGGAGGGCCTCCCT
>SRR4252608_6285660___Sbg4
ACCTCCGAGACAACACGAACATATAGAC
>SRR4252610_1249644___Sbg4
TGAAGATGATCATGATGGCCAGATGTTGT
>SRR4252605_746233___Sbg4
TTTTTGACTACACAAGGACATGGAGGGC
>SRR4252611_6854695___Sbg4
TCCAAGTTTTACCTCCCCCACTAGTAGGT
>SRR4252620_9342886___Sbg4
TACCAACAAGGCGAATATGATGAAGATG
>SRR4252610_10330618___Sbg4
CATTCATACTTTTTTGACTACACAAGGA
>SRR4252610_13596076___Sbg4
CCACTAGTAGGTACTATTTACATGACTC
>SRR4252610_11221469___Sbg4
CCCCACTAGTAGGTACTATTTACATGACT
>SRR4252610_1391209___Sbg4
TCTCCGAGACAACACGAACATATAGACA
>SRR4252613_2716304___Sbg4
TCTCCGAGACAACACGAACATATAGACA
>SRR4252605_7580551___Sbg4
ACCGTTTCGGCCTAAAGTTTCCTGACAT
>SRR4252608_738849___Sbg4
CCCCACTAGTAGGTACTATTTACATGACT
>SRR4252610_4835476___Sbg4
TACTATTTACATGACTCACGCCACCCGG
>SRR4252626_5081180___Sbg4
CATTCATACATTTTTGACTACACAAGGA
>SRR4252609_15743126___Sbg4
GATGAAGATGATCATGATGGCCAGATGA
>SRR4252623_379094___Sbg4
TCCAAGTTTTACCTCCCCCACTAGTAGG
>SRR4252607_2841185___Sbg4
TCCGAGACAACACGAACATATGGACAA
>SRR4252607_2665700___Sbg4
CATTCATACCTTTTTGACTACACAAGGA
>SRR4252611_13500298___Sbg4
CTCCGAGACAACACGAACATATAGACAAA
>SRR4252608_9230264___Sbg4
TGAAGATGATCATGATGGCCAGATGATGT
>SRR4252605_10037803___Sbg4
TACACAAGGACATGGAGGGCCTCCCTGGT
```

```
>SRR4252609_6861820___Sbg4
TACACAAGGACATGGAGGGCCTCCCT
>SRR4252612_6019653___Sbg4
TACACAAGGACATGGAGGGCCTCCCTGGT
>SRR4252606_4082207___Sbg4
GGGGACCGTTTGGGCCTAAAGTTTCCTGACA
>SRR4252605_13583834___Sbg4
ACATTCATACGTTTTTGACTACACAAGGA
>SRR4252619_7354596___Sbg4
GGGCCTTACAGAGCCAAGACCTGTCGGGA
>SRR4252607_2610765___Sbg4
TGAAGATGATCATGATGGCCAGATGATGC
>SRR4252608_9706694___Sbg4
CCCCACTAGTAGGTACTATTTACATGACT
>SRR4252610_7121855___Sbg4
CCCCACTAGTAGGTACTATTTACATGACT
>SRR4252611_1580814___Sbg4
ATGAAGATGATCATGATGGCCAGATGATG
>SRR4252623_1481864___Sbg4
CCCACTAGTAGGTACTATTTACATGACT
>SRR4252606_8223158___Sbg4
CCACTAGTAGGTACTATTTACATGACT
>SRR4252612_1069606___Sbg4
TCCGAGACAACACGAACATATAGACCAA
>SRR4252605_4278990___Sbg4
TCCAAGTTTTACCTCCCCCACTAGTAGG
>SRR4252614_745134___Sbg4
TCCAAGTTTTACCTCCCCCACTAGTAGG
>SRR4252614_6736461___Sbg4
GGACCGTTTGGGCCTAAAGTTTCCTGAC
>SRR4252612_4876868___Sbg4
TGACATTTGTCTTATGGGTGAGGAAA
>SRR4252610_3638993___Sbg4
TCCCCCACTAGTAGGTACTATTTACATG
>SRR4252608_7631874___Sbg4
TACACAAGGACATGGAGGGCCTCCCTGGA
>SRR4252610_2977067___Sbg4
CCCACTAGTAGGTACTATTTACATGACT
>SRR4252621_8563496___Sbg4
GGACCGTTTGAGCCTAAAGTTTCCTGACA
>SRR4252606_10156626___Sbg4
CCCCACTAGTAGGTACTATTTACATGACT
>SRR4252610_13438933___Sbg4
CCCCACTAGTAGGTACTATTTACATGACT
>SRR4252605_6771221___Sbg4
ATACATTCATACTTTTTTGACTACACAAGGA
>SRR4252609_6198366___Sbg4
ATTCATACTTTTTTGACTACACAAGGA
>SRR4252605_10185641___Sbg4
TTTGGGCCTAAAGTTTCCTGACATTTGTCTC
>SRR4252621_8812095___Sbg4
TGATGAAGATGATTATGATGGCCAGATGA
>SRR4252605_12058165___Sbg4
AATTTTCCAAGTTTTACCTCCCCCACTA
>SRR4252606_2937312___Sbg4
TCCGAGACAACACGAACATGTAGACAA
>SRR4252609_10817835___Sbg4
TCCGAGACAACACGAACATGTAGACAAAG
>SRR4252610_10778566___Sbg4
TCCGAGACAACACGAACATGTAGACAAAG
>SRR4252612_8494653___Sbg4
TCCGAGACAACACGAACATGTAGACAAA
>SRR4252608_4774740___Sbg4
TAAACAAGGCGAATATGATGAAGATGAT
>SRR4252611_15025284___Sbg4
TCCGAGACAACACGAACATATAGACAAGG
>SRR4252613_10757862___Sbg4
TCCGAGACAACACGAACATATAGACAA
>SRR4252608_9276620___Sbg4
CCCCACTAGTAGGTACTATTTACATGACT
```

>SRR4252605_168165___Sbg4
ACCGTTTGGGCCTAAAGTTTCCTGACATT
>SRR4252611_11630061___Sbg4
TCCAAGTTTTACCTCCCCCACTAGTAGGT
>SRR4252615_4317510___Sbg4
TATGATGAAGATGATTATGATGGCCAGA
>SRR4252610_9597822___Sbg4
TCCAAGTTTTACCTCCCCCACTAGTAGGT
>SRR4252610_12236353___Sbg4
ATCGGGAGAGGATAGACTATGGATCACA
>SRR4252610_4992034___Sbg4
CCCCACTAGTAGGTACTATTTACATGACT
>SRR4252610_2511306___Sbg4
TACTATTTACATGACTCACGCCACCCGG
>SRR4252626_2903951___Sbg4
TACACAAGGACATGGAGGACCTCCCTGG
>SRR4252605_12768711___Sbg4
ACCTCCGAGACAACACGAACATATAGACAAA
>SRR4252611_66678___Sbg4
AGGCCACCTCCGAGACAACACGAACATA
>SRR4252611_4967838___Sbg4
ACATTCATACTTTTTTGACTACACAAGGA
>SRR4252607_6983584___Sbg4
TGAAGATGATCATGATGGCCAGATGATGT
>SRR4252615_1277363___Sbg4
CACGCCACCCGGGCCTTACAGAGCCAAGA
>SRR4252606_9890450___Sbg4
ACTAGTAGGTACTATTTACATGACTCACGA
>SRR4252609_5425964___Sbg4
CTAGTAGGTACTATTTACATGACTCACGA
>SRR4252606_250547___Sbg4
ACCTCCGAGACAACACGAACATATAGACAAA
>SRR4252623_14665078___Sbg4
TATAGACAAAGACACCATTCACACCCAC
>SRR4252610_4283679___Sbg4
TGAAGATGATCATGATGGCCAGATGATG
>SRR4252606_6019740___Sbg4
TACACAAGGACATGGAGGGCCGCCCTGGA
>SRR4252606_7681378___Sbg4
TACACAAGGACATGGAGGGCCGCCCTG
>SRR4252610_7241034___Sbg4
TACACAAGGACATGGAGGGCCGCCCTGG
>SRR4252612_6758383___Sbg4
TACTTTTTTGACTACACAAGGACATGGA
>SRR4252605_3575892___Sbg4
ACTCAATGCCAGGGCCACCTCCGAGACAA
>SRR4252606_8495885___Sbg4
ACTCAATGCCAGGGCCACCTCCGAGACAA
>SRR4252607_1391695___Sbg4
ACTCAATGCCAGGGCCACCTCCGAGACAA
>SRR4252607_4260552___Sbg4
ACTCAATGCCAGGGCCACCTCCGAGACAAC
>SRR4252618_8084741___Sbg4
TCCGAGACAACACGAACATATAGACAAAG
>SRR4252610_9611557___Sbg4
CCCCACTAGTAGGTACTATTTACATGACT
>SRR4252606_1465466___Sbg4
TAGGTACTATTTACATGACTCACGCCACCT
>SRR4252610_1914611___Sbg4
TACCAACAAGGCGAATATGATGAAGATG
>SRR4252610_9020292___Sbg4
TACACAAGGACATGGAGGGCCTCCCTA
>SRR4252606_3465101___Sbg4
ACACAAGGACATGGAGGGCCTCCCTA
>SRR4252608_9153556___Sbg4
ACCTCCGAGACAACACGAACATATAGACA
>SRR4252609_4604230___Sbg4
TCCGAGACAACACGAACATATAGACAA
>SRR4252610_2745910___Sbg4
TACCAACAAGGCGAATATGATGAGGATGA

>SRR4252605_6623393___Sbg4
GCATTCATACTTTTTTGACTACACAAGGA
>SRR4252609_9148143___Sbg4
TGAAGATGATCATGATGGCCAGATGATGT
>SRR4252626_8532543___Sbg4
TACTATTTACATGACTCACGCAACCCGG
>SRR4252610_9210713___Sbg4
TACACAAGGACATGGAGGGACTCCCTGGA
>SRR4252614_9413278___Sbg4
ACCGTTTGGGCCTAAAGTTTCCTGACA
>SRR4252610_5033938___Sbg4
TACACAAGGACATGGAGGGCCTCCCT
>SRR4252610_1315728___Sbg4
CACTAATACCAACAAGGCGAATATGATGAAG
>SRR4252610_9482547___Sbg4
CCGTTTGGGCCTAAAGTTTCCTGACATTTGT
>SRR4252609_8529074___Sbg4
GTTTGGGCCTAAAGTTTCCTGACATT
>SRR4252622_9161346___Sbg4
TCCGAGACAACACGAACACATAGACA
>SRR4252610_6977334___Sbg4
TGTCTTATGGGTGAGGAAAAACTCCAGA
>SRR4252611_2405652___Sbg4
TGTCTTATGGGTGAGGAAAAACTCCAGA
>SRR4252605_8223709___Sbg4
AGAGCCAAGACCTGTCGGGAGAGGATAGACC
>SRR4252605_9236351___Sbg4
AGAGCCAAGACCTGTCGGGAGAGGATAGACC
>SRR4252611_2904091___Sbg4
CCACTAGTAGGTACTATTTACATGACTC
>SRR4252605_9633767___Sbg4
TGAAGATGATCATGATGGCCAGATGATGG
>SRR4252612_662448___Sbg4
TGACTACACAAGGACATGGAGGGTCTCCC
>SRR4252615_192113___Sbg4
TCCAAGTTTTACCTCCCCCACTAGTGG
>SRR4252607_14632005___Sbg4
TCCGAGACATCACGAACATATAGACAA
>SRR4252606_3720982___Sbg4
TCCGAGACAACACGAACATATAGACAGAGA
>SRR4252607_7307955___Sbg4
ACCAACAAGGCGAATATGATGAAGATGAT
>SRR4252610_515386___Sbg4
TAGGTACTATTTACATGACTCACGCCACCC
>SRR4252606_2593078___Sbg4
GCTCAATGCCAGGGCCACCTCCGAGACAA
>SRR4252618_5776344___Sbg4
GCTCAATGCCAGGGCCACCTCCGAGACAA
>SRR4252619_4330842___Sbg4
TACATGACTCACGCCACCCGGGCCTTACA
>SRR4252606_5887803___Sbg4
TAGGTACTATTTACATGACTCACGCC
>SRR4252608_6980528___Sbg4
CGAGACAACACGAACATATAGACAAAGAT
>SRR4252607_13822836___Sbg4
TACCATTCACACCCACACTCATACCAACAAG
>SRR4252610_10745344___Sbg4
TATGATGAAGATGATTATGATGGCCAGA
>SRR4252605_11668400___Sbg4
CCCCACTAGTAGGTACTATTTACATGACT
>SRR4252605_8633172___Sbg4
TATAGACAAAGACACCATTCACACCCAC
>SRR4252613_2670173___Sbg4
CATTCATACTTTTTTGACTACACAAGGA
>SRR4252611_6120020___Sbg4
CCCCACTAGTAGGTACTATTTACATGACT
>SRR4252610_8168024___Sbg4
CCCCACTAGTAGGTACTATTTACATGACT
>SRR4252605_8912824___Sbg4
ACATTCATACTTTTTTGACTACACAAGGA

>SRR4252623_9806847___Sbg4
TTTTTGACTACACAAGGACATGGAGGGT
>SRR4252610_12131357___Sbg4
GACCTGTCGGGAGAGGATGGACTATG
>SRR4252613_9835313___Sbg4
GACCTGTCGGGAGAGGATGGACTATGGA
>SRR4252622_2872703___Sbg4
TCCGAGACAACACGAACACATAGACAAAG
>SRR4252606_2775517___Sbg4
TGAAGATGATCATGATGGCCAGATGATG
>SRR4252612_8690637___Sbg4
GGGACCGTTTGGGCCTAAAGTTTCCTGACAT
>SRR4252623_10598042___Sbg4
TCCAAGTTTTACCTCCCCCACTAGTAGG
>SRR4252607_13895725___Sbg4
GCTCGATGCCAGGGCCACCTCCGAGACAA
>SRR4252608_3719973___Sbg4
TGAAGATGATTATGATGGCCAGATGA
>SRR4252608_356197___Sbg4
TCCGAGACAACACGAACATATAGACAAAG
>SRR4252617_8009304___Sbg4
ACGAACATATAGACAAAGACACCATT
>SRR4252610_9519784___Sbg4
CATTCATACTTTTTTGACTACACAAGGA
>SRR4252612_4937009___Sbg4
TCATACTTTTTTGACTACACAAGGACATT
>SRR4252605_13680952___Sbg4
TGGGCCTAAAGTTTCCTGACATTTGTCTTA
>SRR4252607_10503018___Sbg4
GCATTCATACTTTTTTGACTACACAAGGA
>SRR4252608_1848867___Sbg4
GCATTCATACTTTTTTGACTACACAAGGA
>SRR4252610_12403533___Sbg4
GCATTCATACTTTTTTGACTACACAAGGA
>SRR4252608_9537379___Sbg4
CCCCACTAGTAGGTACTATTTACATGAT
>SRR4252611_10552186___Sbg4
TACACAAGGACATGGAGGGCCTCCCT
>SRR4252609_4861688___Sbg4
TAGACAAAGACACCATTCACACCCAC
>SRR4252608_11338350___Sbg4
ATGATGAAGATGATTATGATGGCCAGAT
>SRR4252607_1528055___Sbg4
TTTGTCTTATGGGTGAGGAAAAACCCCAG
>SRR4252621_13787691___Sbg4
TATAGACAAAGACACCATTCACACCCACA
>SRR4252609_4142099___Sbg4
TCCAAGTTTTACCTCCCCCACTAGTAGGT
>SRR4252611_7025551___Sbg4
ATTCATACTTTTTTGACTACACAAGGA
>SRR4252611_9536779___Sbg4
ATACATTCATACTTTTTTGACTACACAAGGA
>SRR4252605_14085775___Sbg4
TACACAAGGACATGGAGGGCCTCCCTAGA
>SRR4252614_5770380___Sbg4
TATAGACAAAGACACCATTCACACCCACAC
>SRR4252610_9868608___Sbg4
TCCAAGTTTTACCTCCCCCACTAGTAGGT
>SRR4252606_5252296___Sbg4
ACTTTTTTGACTACACAAGGACATGG
>SRR4252611_6600651___Sbg4
CCGTTTGGGCCTAAAGTTTCCTGACATT
>SRR4252610_13137987___Sbg4
TCCGAGACAACACGAACACATAGACAAAG
>SRR4252613_5960154___Sbg4
ATGAAGATGATCATGATGGCCAGATG
>SRR4252610_65929___Sbg4
TGATGAAGATGATCATGATGGCCAGATG
>SRR4252619_1750931___Sbg4
AAGGACATGGAGGGCCTCCCTGGATA

>SRR4252611_10452898___Sbg4
AGACCGTTTGGGCCTAAAGTTTCCTGACATT
>SRR4252611_1734472___Sbg4
TGGGCCTAAAGTTTCCTGACATTTGTCTT
>SRR4252624_10365088___Sbg4
TAGGTACTATTTACATGACTCACGCCACT
>SRR4252608_8535477___Sbg4
ACATTCATACTTTTTTGACTACACAAGG
>SRR4252616_11576497___Sbg4
GCCTCCGAGACAACACGAACATATAGA
>SRR4252606_4770609___Sbg4
AGAGACAACACGAACATATAGACAAA
>SRR4252610_6886336___Sbg4
AGACACCATTCACACCCACACTCATACCA
>SRR4252610_10193609___Sbg4
CCCCACTAGTAGGTACTATTTACATGACT
>SRR4252623_10118772___Sbg4
TGATGAAGATGATCATGATGGCCAGATG
>SRR4252612_7073450___Sbg4
TGAAGATGATCATGATGGCCAGATGATG
>SRR4252610_9026068___Sbg4
ATTCATACTTTTTTGACTACACAAGGACAT
>SRR4252610_17756___Sbg4
CCCCACTAGTAGGTACTATTTACATGACT
>SRR4252608_8467947___Sbg4
CCCCACTAGTAGGTACTATTTACATGACT
>SRR4252610_8254939___Sbg4
TAGGTACTATTTACATGACTCACGCCA
>SRR4252619_14204554___Sbg4
ACATTCATACTTTTTTGACTACACAAGGA
>SRR4252611_131726___Sbg4
ATCACGCCACCCGGGCCTTACAGAGCCAAGA
>SRR4252620_6887362___Sbg4
TTTGACTACACAAGGACATGGAGGGCCTC
>SRR4252611_12054483___Sbg4
AGACCTGTCGGGAGAGGATAGACTATG
>SRR4252608_10356029___Sbg4
TCCCCCACTAGTAGGTACTATTTATATGA
>SRR4252610_4441029___Sbg4
CCCCACTAGTAGGTACTATTTACATGACT
>SRR4252610_3255999___Sbg4
TTGACTACACAAGGACATGGAGGGCCTCCC
>SRR4252612_3244690___Sbg4
TACTATTTACATGACTCACGCCACCCGG
>SRR4252611_14633554___Sbg4
ACATTCATACTTTTTTGACTACACAAGGA
>SRR4252606_7790506___Sbg4
GCTCAATGCCAGGGCCACCTCCGAGACAA
>SRR4252605_11072837___Sbg4
GAGGATAGACTATGGATCACATATACAT
>SRR4252605_7474121___Sbg4
ATGAAGATGATTATGATGGCCAGATGA
>SRR4252605_4059597___Sbg4
CCCCACTAGTAGGTACTATTTACATGACT
>SRR4252608_5924356___Sbg4
CCCACTAGTAGGTACTATTTACATGACTC
>SRR4252624_4389658___Sbg4
TACACAAGGACATGGAGGGCCTCCCTGGA
>SRR4252613_11155105___Sbg4
CATATACATTCATACTTTTTTGACTACA
>SRR4252606_5731687___Sbg4
TATAGACAAAGACACCATTCACACCCAC
>SRR4252610_12013720___Sbg4
TACTATTTACATGACTCACGCCACCCGGGC
>SRR4252605_9505149___Sbg4
CACGCCACCCGGGCCTTACAGAGCCAA
>SRR4252605_14345988___Sbg4
CCCCACTAGTAGGTACTATTTACATGACT
>SRR4252624_5130646___Sbg4
TCCCTGGATAAGTGATCAGCTCAATGC

>SRR4252612_6572075___Sbg4
CCCCACTAGTAGGTACTATTTACATGACT
>SRR4252608_6925533___Sbg4
TCCGAGACAACACGAACATATAGACAAGG
>SRR4252610_9236967___Sbg4
TCCGAGACAACACGAACATATAGACAAGG
>SRR4252611_773622___Sbg4
TCCGAGACAACACGAACATATAGACAAGG
>SRR4252623_838984___Sbg4
ACATTCATACTTTTTTGACTACACAAGGA
>SRR4252607_7629052___Sbg4
CATTCATACTTTTTTGACTACACAAGGA
>SRR4252612_1330507___Sbg4
TCACACCCACACTCATACCAACAAGGCA
>SRR4252612_7467056___Sbg4
GATCAGCTCAATGCCAGGGCCACCTCCGA
>SRR4252612_3507215___Sbg4
ACATTCATAATTTTTTGACTACACAAGGA
>SRR4252609_14512257___Sbg4
TACACAAGGACATGGAGGGCCTCCCTGGA
>SRR4252606_3708570___Sbg4
ACCTCCGAGACAACACGAACATATAGACAAA
>SRR4252609_6986982___Sbg4
GCTCAATGCCAGGGCCACCTCCGAGACAA
>SRR4252610_11403756___Sbg4
TACACAAGGACATGGAGGGCCTCCCC
>SRR4252612_1885647___Sbg4
TACACAAGGACATGGAGGGCCTCCCC
>SRR4252605_3838542___Sbg4
TACCAACAAGGCGAATATGATGAAGAC
>SRR4252607_366165___Sbg4
AGATGATGTCTGGGGACCGTTTGGGCCTAAA
>SRR4252606_3571075___Sbg4
CTGTCGGGAGAGGATAGACTATGGATCAT
>SRR4252609_4369218___Sbg4
ACATTCATACTTTTTTGACTACACAAGGA
>SRR4252608_9100049___Sbg4
TACACAAGGACATGGAGGGCCTCCCA
>SRR4252614_1054253___Sbg4
TACACAAGGACATGGAGGGCCTCCCA
>SRR4252608_4481683___Sbg4
CAGCTCAATGCCAGGGCCACCTCCGAGACAA
>SRR4252619_10107294___Sbg4
GCCTTACAGAGTCAAGACCTGTCGGGAGAG
>SRR4252614_4604370___Sbg4
GATCACATATACATTCATACTTTTTTGA
>SRR4252610_13034393___Sbg4
CCCACTAGTAGGTACTATTTACATGACT
>SRR4252610_744213___Sbg4
GCCGTTTGGGCCTAAAGTTTCCTGACATT
>SRR4252624_1824762___Sbg4
CTCCGAGACAACACGAACATATAGACAAA
>SRR4252616_13021596___Sbg4
ACACAAGGACATGGAGGGCCTCCCTGG
>SRR4252607_9705269___Sbg4
CCTGTCGGGAGAGGATAGACTATGGATC
>SRR4252605_6542338___Sbg4
CCCCACTAGTAGGTACTATTTACATGAC
>SRR4252611_3674836___Sbg4
ACATTCATACTTTTTTGACTACACAAGGA
>SRR4252606_4164532___Sbg4
CCCCACTAGTAGGTACTATTTACATGACC
>SRR4252610_11729346___Sbg4
CCCACTAGTAGGTACTATTTACATGACC
>SRR4252610_2706337___Sbg4
CCCCACTAGTAGGTACTATTTACATGACT
>SRR4252608_7857605___Sbg4
TGTCTTATGGGTGAGGAAAAACTCCAGA
>SRR4252618_1923233___Sbg4
TATTTACATGACTCACGCCACCCCGGCCT

```
>SRR4252612_6101632___Sbg4
TACATGACTCACGCCACCCCGGCCTTACAG
>SRR4252624_3080853___Sbg4
ACATTCATACTTTTTTGACTACACAAGGA
>SRR4252607_9761544___Sbg4
TCCATTCCATTTTCCAAGTTTTACCTCCT
>SRR4252606_8293957___Sbg4
TGGGCCTAAAGTTTCCTGACATTTGTCTT
>SRR4252605_11136775___Sbg4
ACTAGTAGGTACTATTTACATGACTCACGT
>SRR4252612_8049297___Sbg4
TGATGAAGATGATTATGATGGCCAGATG
>SRR4252623_10775905___Sbg4
CATTCATACTTTTTTGACTACACAAGGA
>SRR4252625_2438806___Sbg4
TGACTACACAAGGACATGGAGGGCCTCCC
>SRR4252621_9478191___Sbg4
TGATGAAGATGATCATGATGGCCAGATG
>SRR4252624_6868462___Sbg4
AGACCTGTCGGGAGAGGATAGACTATG
>SRR4252610_4481137___Sbg4
TCGGGAGAGGATAGACTATGGATCAT
>SRR4252609_4197317___Sbg4
TACTTTTTTGACTACACAAGGACATGG
>SRR4252613_11278346___Sbg4
ACCGTTTGGGCCTAAAGTTTCCTGACATT
>SRR4252610_6628303___Sbg4
CTAGTAGGTACTATTTACATGACTCACGC
>SRR4252625_5482264___Sbg4
CGTTTGGGCCTAAAGTTTCCTGACATT
>SRR4252619_14705353___Sbg4
TTTCCAAGTTTTACCTCCCCCACTAGTA
>SRR4252610_13182329___Sbg4
ACACATTCATACTTTTTTGACTACACA
>SRR4252625_6562660___Sbg4
TGAAGATGATCATGATGGCCAGATGATG
>SRR4252610_8549861___Sbg4
TATAGACAAAGACACCATTCACACCCACAC
>SRR4252616_7639635___Sbg4
GATGAAGATGATTATGATGGCCAGATGA
>SRR4252609_1541182___Sbg4
GCTCAATGCCAGGGCCACCTCCGAGACAA
>SRR4252609_3180155___Sbg4
TGAAGATGATCATGATGGCCAGATGATG
>SRR4252606_10154358___Sbg4
GCCGTTTGGGCCTAAAGTTTCCTGACAT
>SRR4252607_8244229___Sbg4
GCCGTTTGGGCCTAAAGTTTCCTGACATT
>SRR4252623_5231458___Sbg4
CTCCGAGACAACACGAACATATAGACAAA
>SRR4252610_939451___Sbg4
CCAACAAGGCGAATATGATGAAGATGAT
>SRR4252610_10492345___Sbg4
TTCCAAGTTTTACCTCCCCCACTAGTA
>SRR4252611_12263921___Sbg4
TCACGCCACCCGGGCCTTACAGAGCCAAGA
>SRR4252612_558569___Sbg4
GCCAGGGCCACCTCCGAGACAACACGAACA
>SRR4252609_16973070___Sbg4
TATAGACAAAGACACCATTCACACCCACAC
>SRR4252618_3285177___Sbg4
ACCGTTTGGGCCTAAAGTTTCCTGACA
>SRR4252605_13573708___Sbg4
CAATGCTAGGGCCACCTCCGAGACAA
>SRR4252624_1196220___Sbg4
CATTCATACTTTTTTGACTACACAAGGA
>SRR4252606_3493191___Sbg4
AGCTCAATGCCAGGGCCACCTCCGAGA
>SRR4252619_11751597___Sbg4
TGATGAAGATGATTATGATGGCCAGATG
```

>SRR4252611_710992___Sbg4
ATTCATACTTTTTTGACTACACAAGGA
>SRR4252623_13970324___Sbg4
TACACAAGGACATGGAGGGCCTCCCTGG
>SRR4252610_704432___Sbg4
ACCGTTTGAGCCTAAAGTTTCCTGACATT
>SRR4252625_6673408___Sbg4
CCCCACTAGTAGGTACTATTTACATGACT
>SRR4252616_1478258___Sbg4
TATAGACAAAGACACCATTCACACCCA
>SRR4252624_8050981___Sbg4
TGATGAAGATGATTATGATGGCCAGA
>SRR4252611_5179813___Sbg4
CCCCACTAGTAGGTACTATTTACATGACC
>SRR4252612_4206731___Sbg4
CCCACTAGTAGGTACTATTTACATGACC
>SRR4252612_4325529___Sbg4
CCCACTAGTAGGTACTATTTACATGACC
>SRR4252616_3622027___Sbg4
GGACCGTTTGGGCCTAAAGTTTCCTGACA
>SRR4252608_366157___Sbg4
ACCGTTTGGGCCTAAAGTTTCCTGACAT
>SRR4252611_12636898___Sbg4
CTCCGAGACAACACGAACATATAGACAAA
>SRR4252607_13647673___Sbg4
ATTCATACTTTTTTGACTACACAAGGA
>SRR4252622_8666146___Sbg4
ATACACATTCATACTTTTTTGACTACACA
>SRR4252612_5647415___Sbg4
TACACAAGGACATGGAGGGCCTCCCTGA
>SRR4252606_10339050___Sbg4
TGGGCCTAAAGTTTCCTGACATTTGTCTTG
>SRR4252605_12863407___Sbg4
GCTCAATGCCAGGGCCACCTCCGAGACAACA
>SRR4252612_4437975___Sbg4
TTAACAAGGCGAATATGATGAAGATGAT
>SRR4252610_5817390___Sbg4
CACGAACATATAGACAAAGACACCATTC
>SRR4252609_7284087___Sbg4
TGAAGATGATCATGATGGCCAGATGATGT
>SRR4252609_8609489___Sbg4
TGAAGATGATCATGATGGCCAGATGATGT
>SRR4252613_262949___Sbg4
TCCAAGTTTTACCTCCCCCACTAGTAGG
>SRR4252610_2799221___Sbg4
CCCCACTAGTAGGTACTATTTACATGACT
>SRR4252626_6533550___Sbg4
TGACTACACAAGGACATGGAGGGCCT
>SRR4252611_1082904___Sbg4
TACACAAGGACATGGAGGGCCTCCCTGGA
>SRR4252611_9421960___Sbg4
TACACAAGGACATGGAGGGCCTCCCT
>SRR4252610_11109193___Sbg4
CCCACTAGTAGGTACTATTTACATGACT
>SRR4252607_8055551___Sbg4
CATTCATACTTTTTTGACTACACAAGGA
>SRR4252612_6443076___Sbg4
TACACAAGGACATGGAGGGCCTCCCT
>SRR4252605_12788432___Sbg4
GAGGATAGACTATGGATCACATATACAC
>SRR4252608_6061559___Sbg4
CCCCACTAGTAGGTACTATTTACATGACT
>SRR4252611_8512528___Sbg4
TCCCCCACTAGTAGGTACTATTTACATGA
>SRR4252611_11566637___Sbg4
ACCTCCGAGACAACACGAACATATAGACAAA
>SRR4252616_1199966___Sbg4
TTCCATTCCATTTTCCAAGTTTTACC
>SRR4252607_11480145___Sbg4
CCCCACTAGTAGGTACTATTTACATGACTT

>SRR4252610_7439489___Sbg4
CCCCACTAGTAGGTACTATTTACATGACT
>SRR4252612_5894066___Sbg4
TGCCAGAGCCACCTCCGAGACAACACGA
>SRR4252610_1131095___Sbg4
TCCGAGACAACACGAACATATAGACCAA
>SRR4252610_347115___Sbg4
TCCGAGACAACACGAACATATAGACCAA
>SRR4252605_10998599___Sbg4
CCGAGACAACACGAACATATAGACCAA
>SRR4252622_744571___Sbg4
CCAAGTTTTACCTCCCCCACTAGTAGGT
>SRR4252613_7396951___Sbg4
ATGATGTCTGGGGACCGTTTGGGCCT
>SRR4252610_2678499___Sbg4
CATTCATACTTTTTTGACTACACAAGGA
>SRR4252610_7859532___Sbg4
CCCCACTAGTAGGTACTATTTACATGACT
>SRR4252619_15151215___Sbg4
TGACTACACAAGGACATGGAGGGCCGCCC
>SRR4252610_12978591___Sbg4
TACACAAGGACATGGAGGGCCGCCCTGG
>SRR4252611_2689902___Sbg4
TACACAAGGACATGGAGGGCCGCCCTGGA
>SRR4252607_7484705___Sbg4
TGATGAAGATGATTATGATGGCCAGATGT
>SRR4252606_7391914___Sbg4
TGAAGATGATTATGATGGCCAGATGTCT
>SRR4252608_7265368___Sbg4
TGATGAAGATGATCATGATGGCCGGATGA
>SRR4252610_8032990___Sbg4
TGATGAAGATGATCATGATGGCCGGATGA
>SRR4252609_662619___Sbg4
TGAAGATGATCATGATGGCCGGATGATGT
>SRR4252605_13212956___Sbg4
GAAGATGATCATGATGGCCGGATGATGT
>SRR4252608_3407271___Sbg4
CCCCACTAGTAGGTACTATTTACATGACT
>SRR4252613_440676___Sbg4
AGCCACCTCCGAGACAACACGAACATATA
>SRR4252608_6779058___Sbg4
TACACAAGGACATGGAGGGCCTCCCT
>SRR4252611_9657319___Sbg4
TGATGAAGATGATCATGATGGCCAGATG
>SRR4252625_10988623___Sbg4
TGGGCCTAAAGTTTCCTGACATTTGTCTTA
>SRR4252608_10685617___Sbg4
TGGGCCTAAAGTTTCCTGACATTTGTCTTT
>SRR4252611_4293438___Sbg4
TGGGCCTAAAGTTTCCTGACATTTGTCTTT
>SRR4252623_10330154___Sbg4
TCCGAGACAACACGAACACATAGACAAA
>SRR4252609_9423046___Sbg4
TCCGAGACAACACGAACATATAGACA
>SRR4252609_3482265___Sbg4
CCCCACTAGTAGGTACTATTTACATGACT
>SRR4252607_3346576___Sbg4
TATGATGAAGATGATTATGATGGCCAGATG
>SRR4252610_4289710___Sbg4
CCCACTAGTAGGTACTATTTACATGACT
>SRR4252611_11385007___Sbg4
CTTTTTTACTACACAAGGACATGGAG
>SRR4252605_11506622___Sbg4
TGGGCCTAAAGTTTCCTGACATTTGTCTTT
>SRR4252608_10731561___Sbg4
TGGGCCTAAAGTTTCCTGACATTTGTCTTT
>SRR4252608_3043051___Sbg4
TATGATGAAGATGATCATGATGGACAGATG
>SRR4252610_6155940___Sbg4
CCCCACTAGTAGGTACTATTTACATGACT

>SRR4252607_10158560___Sbg4
ACCGTTTGGGCCTAAAGTTTCCTGACAT
>SRR4252607_8533196___Sbg4
TCCGAGACAACACGAACATATAGACAAGG
>SRR4252611_15004829___Sbg4
AGACCGTTTGGGCCTAAAGTTTCCTGACATT
>SRR4252612_1894822___Sbg4
TCCCTGGATAAGTGATCATCTCAATGCC
>SRR4252611_9043014___Sbg4
TCCCCCACTAGTAGGTACTATTTACATGA
>SRR4252606_9244692___Sbg4
CCCCACTAGTAGGTACTATTTACATGACT
>SRR4252605_7127322___Sbg4
TCCGAGACAACACGAACATGTAGACAA
>SRR4252612_2065610___Sbg4
TCCGAGACAACACGAACATGTAGACAAA
>SRR4252612_5621220___Sbg4
TCCGAGACAACACGAACATGTAGACAAAG
>SRR4252610_3056554___Sbg4
TGACTACACAAGGACATGGAGGGCCTCCCA
>SRR4252606_9165258___Sbg4
TACACAAGGACATGGAGGGCCTCCCAGGAT
>SRR4252625_4122570___Sbg4
TACACAAGGACATGGAGGGCCTCCCAGGA
>SRR4252610_11445116___Sbg4
GCCAGGGCCACCTCCGAGACAACACGA
>SRR4252607_3464079___Sbg4
TACATTCATACTTTTTTGACTACACAGG
>SRR4252612_6115618___Sbg4
CTCCGAGACAACACGAACATATAGACAAA
>SRR4252626_1614593___Sbg4
TGAAGATGATCATGATGGCCAGATGATGT
>SRR4252609_2529125___Sbg4
GCTCAATGCCAGGGCCACCTCCGAGAC
>SRR4252610_8734286___Sbg4
CTCCGAGACAACACGAACATATAGAGAA
>SRR4252608_12509234___Sbg4
TCGTTTGGGCCTAAAGTTTCCTGACAT
>SRR4252614_6002446___Sbg4
TCGTTTGGGCCTAAAGTTTCCTGACAT
>SRR4252612_1621154___Sbg4
CTTTTTTGACTACACAAGGACATGGAG
>SRR4252607_6663721___Sbg4
GCTCAATGCCAGGGCCACCTCCGAGACAA
>SRR4252610_12941966___Sbg4
ATTCATACTTTTTTGACTACACAAGGA
>SRR4252606_7228280___Sbg4
CCCACTAGTAGGTACTATTTACATGACT
>SRR4252613_7885240___Sbg4
TACACAAGGACATGGAGGGCCTCCCTG
>SRR4252611_3456883___Sbg4
TAAGTGATCAGCTCAATGCCAGGGTT
>SRR4252605_11146519___Sbg4
TCAATGCCAGGGCCACCTCCGAGACAACACG
>SRR4252609_5652759___Sbg4
TGATGAAGATGATTATGATGGCCAGATG
>SRR4252608_4164653___Sbg4
AGAGCCAAGACCTGTCGGGAGAGGATAGA
>SRR4252609_7555930___Sbg4
CCCCACTAGTAGGTACTATTTACATGACT
>SRR4252612_7615147___Sbg4
ATTTACATGACTCACGCCACCCGGGT
>SRR4252610_10878149___Sbg4
CCCACTAGTAGGTACTATTTACATGACTC
>SRR4252614_1671048___Sbg4
TTTGACTACACAAGGACATGGAGAGCCTC
>SRR4252608_6089219___Sbg4
TAAACAAGGCGAATATGATGAAGATGAT
>SRR4252607_142549___Sbg4
TATGATGAAGATGATTATGATGGCCAGAT

>SRR4252625_13602531___Sbg4
ACACGAACATATAGACAAAGACACCAT
>SRR4252622_8859254___Sbg4
GCCAAGACCTGTCGGGAGAGGATAGGCTAT
>SRR4252609_12401434___Sbg4
TAGGTACTATTTACATGACTCACGCCACTG
>SRR4252609_6957845___Sbg4
TAGGTACTATTTACATGACTCACGCCACT
>SRR4252610_8271846___Sbg4
TAGGTACTATTTACATGACTCACGCCACTG
>SRR4252606_8400907___Sbg4
ACCTCCGAGACAACACGAACATATAGACA
>SRR4252610_11123703___Sbg4
GATGAAGATGATCATGATGGCCGGATG
>SRR4252609_14591831___Sbg4
TGAAGATGATCATGATGGCCGGATGATGT
>SRR4252609_7335486___Sbg4
TGAAGATGATCATGATGGCCGGATGATGT
>SRR4252610_1517404___Sbg4
TGAAGATGATCATGATGGCCGGATGATGT
>SRR4252611_5539320___Sbg4
TGAAGATGATCATGATGGCCGGATGATGT
>SRR4252620_4824131___Sbg4
TATAGACAAAGACACCATTCACACCCA
>SRR4252610_4939040___Sbg4
TGATGAAGATGATTATGATGGCCAGATG
>SRR4252610_4604593___Sbg4
CCCACTAGTAGGTACTATTTACATGACT
>SRR4252613_9764133___Sbg4
TTTTTTGACTACACAAGGACATGGAGAGC
>SRR4252612_399317___Sbg4
TTTGACTACACAAGGACATGGAGAGCCTCC
>SRR4252619_14217282___Sbg4
TCCGAGACAACACGAACATATAGACAAAG
>SRR4252613_9488880___Sbg4
CGAGACAACACGAACATATAGACAAA
>SRR4252606_3518919___Sbg4
ATGAAGATGATTATGATGGCCTGATGA
>SRR4252625_9216779___Sbg4
CCCCACTAGTAGGTACTATTTACATGGC
>SRR4252606_6648739___Sbg4
TGAAGATGATCATGATGGCCAGATGATA
>SRR4252610_1467712___Sbg4
TGAAGATGATCATGATGGCCAGATGATA
>SRR4252611_6173484___Sbg4
ATTCATTCATACTTTTTTGACTACACA
>SRR4252609_3869726___Sbg4
ACATTCATACTTTTTTGACTACACAAGGA
>SRR4252611_3489936___Sbg4
TACACAAGGACATGGAGGGCCTCCCTGGA
>SRR4252611_15226582___Sbg4
CATTCATACTTTTTTGACTACACAAGGA
>SRR4252606_9348438___Sbg4
CCCACTAGTAGGTACTATTTACATGACT
>SRR4252625_3548641___Sbg4
TCCAAGTTTTACCTCCCCCACTAGTAGGTAC
>SRR4252611_13372738___Sbg4
CCACTAGTAGGTACTATTTACATGACTC
>SRR4252611_9036399___Sbg4
CTCCGAGACAACACGAACATATAGACAA
>SRR4252624_8794023___Sbg4
TTTGGGCCTAAAGTTTCCTGACATTTGT
>SRR4252609_14349472___Sbg4
TGTCTTATGGGTGAGGAAAAACCCCAA
>SRR4252610_11743075___Sbg4
GCCTCCGAGACAACACGAACATATAGACA
>SRR4252612_3514447___Sbg4
TATTCATACTTTTTTGACTACACAAGGA
>SRR4252611_12828473___Sbg4
GGGCCTAAAGTTTCCTGACATTTGTCTTA

>SRR4252609_11478826___Sbg4
TACACAAGGACATGGAGGGTCTCCCTGG
>SRR4252611_9268558___Sbg4
CCGAGACAACACGAACATATAGACAAA
>SRR4252622_3960220___Sbg4
TGAAGATGATCATGATGGCCAGATGATGC
>SRR4252610_8904413___Sbg4
TACACAAGGACATGGAGGGCCTCCCT
>SRR4252613_1825654___Sbg4
GCTCAATGCCAGGGCCACCTCCGAGACA
>SRR4252611_11617126___Sbg4
GCTCAATGTCAGGGCCACCTCCGAGACA
>SRR4252608_2717235___Sbg4
ACCTCCGAGACAACACGAACATATAGACA
>SRR4252609_3778615___Sbg4
TCCGAGACAACACGAACATATAGACAA
>SRR4252622_4765808___Sbg4
ATTCATACTTTTTTGACTACACAAGGA
>SRR4252623_12519629___Sbg4
CCGTTTGGACCTAAAGTTTCCTGACAT
>SRR4252605_12059429___Sbg4
GCTCAATGCCAGGGCCACCTCCGAGACAA
>SRR4252611_3738746___Sbg4
CCTACAAGGCGAATATGATGAAGATGAT
>SRR4252610_12592081___Sbg4
TCCAAGTTTTACCTCCCCCACTAGTAGGTA
>SRR4252610_1160826___Sbg4
TCCGAGACAACACGAACATATAGACAGA
>SRR4252610_8986485___Sbg4
TCCGAGACAACACGAACATATAGACAGAC
>SRR4252609_7935537___Sbg4
CCCCACTAGTAGGTACTATTTACATGACT
>SRR4252611_11583558___Sbg4
TATGATGAAGATGATTATGATGGCCAGAT
>SRR4252623_2098454___Sbg4
TTTCCTGACATTTGTCTTATGGGCGAG
>SRR4252607_7521173___Sbg4
TTTCCAAGTTTTACCTCCCCCACTAGT
>SRR4252615_12171504___Sbg4
CACGCCACCCGGGCCTTACAGAGCCAAGA
>SRR4252606_7625849___Sbg4
GCTCAATGCCAGGGCCACCTCCGAGACAA
>SRR4252605_4682519___Sbg4
CTCAATTCCAGGGCCACCTCCGAGACAA
>SRR4252613_11238967___Sbg4
ACCGTTTGGGCCTAAAGTTTCCTGACATT
>SRR4252611_9852216___Sbg4
CACGCCACCCGGGCCTTACAGAGCCAAGA
>SRR4252610_7273949___Sbg4
TACTTTTTTGACTACACAAGGACATGGA
>SRR4252612_4142346___Sbg4
CCAAGTTTTACCTCCCCCACTAGTAGGT
>SRR4252608_745220___Sbg4
CCCCACTAGTAGGTACTATTTACATGACT
>SRR4252611_6489267___Sbg4
TACACAAGGACATGGAGGGCATCCCTGA
>SRR4252607_3370886___Sbg4
CCCCACTAGTAGGTACTAATTACATGACT
>SRR4252610_11193846___Sbg4
CCCCACTAGTAGGTACTAATTACATGACT
>SRR4252611_645562___Sbg4
CCCCACTAGTAGGTACTAATTACATGACT
>SRR4252619_11784765___Sbg4
ACACATTCATACTTTTTTGACTACACA
>SRR4252616_9714445___Sbg4
CATTCATACTTTTTTGACTACACAAGGA
>SRR4252605_15125123___Sbg4
TACACAAGGACATGGAGGGCCTCCCTGT
>SRR4252610_5268591___Sbg4
CCCACTAGTAGGTACTATTTACATGACT

>SRR4252610_4154247___Sbg4
TCCAAGTTTTACCTCCCCCACTAGTAGGT
>SRR4252605_3986324___Sbg4
GCTCAATGCCAGGGCCACCTCCGAGACAA
>SRR4252618_10240719___Sbg4
TGGGCCTAAAGTTTCCTGACATTTGTCTTA
>SRR4252610_1073792___Sbg4
CCCCACTAGTAGGTACTATTTACATGACT
>SRR4252611_7830540___Sbg4
TGAAGATGATCATGATGGCCAGATGATGT
>SRR4252609_6336745___Sbg4
CCCACTAGTAGGTACTATTTACATGACT
>SRR4252621_7268502___Sbg4
TACACAAGGACATGGAGGGCCTCCCTG
>SRR4252611_1906323___Sbg4
GCCGTTTGGGCCTAAAGTTTCCTGACATT
>SRR4252608_2696060___Sbg4
CCCCACTAGTAGGTACTATTTACATAACT
>SRR4252610_3016003___Sbg4
GCTCAATGCCAGGGCCACCTCCGAGACA
>SRR4252612_4909491___Sbg4
TCCGAGACAACACGAACATGTAGACAAAGA
>SRR4252607_14708091___Sbg4
CTCCAAGACAACACGAACATATAGACAAA
>SRR4252614_4195491___Sbg4
TCCAAGACAACACGAACATATAGACAAA
>SRR4252612_257606___Sbg4
ACGCCACCAGGGCCTTACAGAGCCAAGA
>SRR4252609_15555128___Sbg4
AGGGCCTTACAGAGCCAAGACCTGTCGGGAG
>SRR4252611_2257806___Sbg4
AAACAAGGCGAATATGATGAAGATGAT
>SRR4252606_5768759___Sbg4
TCCGAGACAACACGAACATGTAGACAA
>SRR4252610_5281456___Sbg4
TCCGAGACAACACGAACATGTAGACAAAG
>SRR4252607_7495412___Sbg4
TACACAAGGACATGGAGGGCCTCCCT
>SRR4252623_15028460___Sbg4
TGATGAAGATGATTATGATGGCCAGATG
>SRR4252607_13232781___Sbg4
ATACATTCATACTTTTTTGACTACACAAGGA
>SRR4252609_3502683___Sbg4
ACATTCATACTTTTTTGACTACACAAGGA
>SRR4252611_11597078___Sbg4
AGGACCGTTTGGGCCTAAAGTTTCCTGACAT
>SRR4252615_2649431___Sbg4
AGGACCGTTTGGGCCTAAAGTTTCCTGACA
>SRR4252610_648247___Sbg4
TCCGAGACAACACGAACATATAGACTAA
>SRR4252612_8811755___Sbg4
TCCGAGACAACACGAACATATAGACTAA
>SRR4252605_14657997___Sbg4
ATTCATACTTTTTTGACTACACAAGG
>SRR4252613_8112003___Sbg4
TACTTTTTTGACTACACAAGGACATGG
>SRR4252606_3413651___Sbg4
CCACCTCCGAGACAACACGAACATAT
>SRR4252605_5493103___Sbg4
TTTTGACTACACAAGGACATGGAGGGCATC
>SRR4252610_8437038___Sbg4
TACACAAGGACATGGAGGGCATCCCTGGA
>SRR4252612_3418002___Sbg4
TACACAAGGACATGGAGGGCATCCCTGG
>SRR4252622_1994852___Sbg4
CCTCCGAGACGACACGAACATATAGACA
>SRR4252608_9860288___Sbg4
ACACGAACATATAGACAAAGACACCAT
>SRR4252609_232398___Sbg4
GCTCAATGCCAGGGCCACCTCCGAGACA

>SRR4252617_5582455___Sbg4
CCTCCGAGACAACACGAACATATAGACA
>SRR4252605_852322___Sbg4
TCCGAGACAACACGAACATATAGACAGA
>SRR4252606_3243220___Sbg4
TCCGAGACAACACGAACATATAGACAGAG
>SRR4252618_1263325___Sbg4
TATTTACATGACTCACGCCACCCGGGCCT
>SRR4252611_9465606___Sbg4
ACATATACATTCATACTTTTTGACTACA
>SRR4252624_3372126___Sbg4
TATAGACAAAGACACCATTCACACCCACA
>SRR4252606_4914833___Sbg4
CGGTTTGGGCCTAAAGTTTCCTGACA
>SRR4252611_2196557___Sbg4
ACATTCATACTTTTTTGACTACACAAGGA
>SRR4252607_8188881___Sbg4
TTTGTCTTATGGGTGAGGAAAAACTCCAGA
>SRR4252609_13793395___Sbg4
TGTCTTATGGGTGAGGAAAAACTCCAGA
>SRR4252610_12544741___Sbg4
TACATTCATACTTTTTTGACAACACAAGGA
>SRR4252610_8908032___Sbg4
CCGTTTGGGCCTAAAGTTTCCTGACATT
>SRR4252613_4249356___Sbg4
ACACATATACATTCATACTTTTTTGACTACA
>SRR4252610_4835369___Sbg4
TGGGCCTAAAGTTTCCTGACATTTGTCTT
>SRR4252621_2262724___Sbg4
GCTCAATGCCAGGGCCACCTCCGAGACAA
>SRR4252610_8975751___Sbg4
CCCCACTAGTAGGTACTATTTACATGACC
>SRR4252608_343755___Sbg4
CCCACTAGTAGGTACTATTTACATGACC
>SRR4252610_11446882___Sbg4
CCCACTAGTAGGTACTATTTACATGACC
>SRR4252610_12750927___Sbg4
CCCACTAGTAGGTACTATTTACATGACC
>SRR4252613_7454705___Sbg4
TCAGCTCAATGCCAGGGCCACCTCTGAG
>SRR4252619_12052749___Sbg4
CATTCATACTTTTTTGACTACACAAGGA
>SRR4252610_8365223___Sbg4
ATTCATACTTTTTTGACTACACAAGGA
>SRR4252611_7330640___Sbg4
CCCACTAGTAGGTACTATTTACATGACT
>SRR4252616_2370893___Sbg4
TCTTACAGAGCCAAGACCTGTCGGGAGA
>SRR4252615_8903916___Sbg4
ACATTTATACTTTTTTGACTACACAAGGA
>SRR4252610_5720432___Sbg4
TATAGACAAAGACACCATTCACACCCACAC
>SRR4252615_3914433___Sbg4
TAGGTACTATTTACATGACTCATGCT
>SRR4252612_5035395___Sbg4
TGATGTCTGGGGACCGTTTGGGCCTA
>SRR4252606_6281270___Sbg4
TCCGAGACAACACGAACATTTAGACAAA
>SRR4252624_6506771___Sbg4
CCCCACTAGTAGGTACTATTTACATGAT
>SRR4252611_1048271___Sbg4
TACACAAGGACATGGAGGACCTCCCT
>SRR4252613_152579___Sbg4
TGCCAGGGCCACCTCCGAGACAACATG
>SRR4252619_5802765___Sbg4
TACACAAGGACATGGAGGGCCTCCCTGG
>SRR4252621_10920584___Sbg4
TCCTGACATTTGTCTTATGGGTGAGGAA
>SRR4252613_11510092___Sbg4
CATTCATACTTTTTTGACTACACAAGGA

>SRR4252611_13263722___Sbg4
TACACAAGGACATGGAGGGCCTCCCTGGA
>SRR4252611_4870051___Sbg4
TACACAAGGACATGGAGGGCCTCCCTGGA
>SRR4252605_7283011___Sbg4
TATACATTCATACTTTTTTGACTACACA
>SRR4252610_1981846___Sbg4
TCCGAGACAACACGAACATATAGACAAAGA
>SRR4252607_12198433___Sbg4
TATTTACATGACTCACGCCACTCGGG
>SRR4252609_15088169___Sbg4
CATTCATACTTTTTTGACTACACAAGGA
>SRR4252605_1529423___Sbg4
GCTCAATGCCAGGGCCACCTCCGAGACAACA
>SRR4252607_1505783___Sbg4
CCGAGACAACACGAACATATAGACAAAGA
>SRR4252620_7910663___Sbg4
GCTCAATGCCAGGGCCACCTCCGAGACAA
>SRR4252618_10030000___Sbg4
TCCGAGACAACACGAACATATAGACAAAG
>SRR4252605_5410340___Sbg4
TTTTTTGACTACACAAGGACATGGAGGGA
>SRR4252622_6956979___Sbg4
TGAAGATGATCATGATGGCCAGATGATGT
>SRR4252609_14876426___Sbg4
ATATGATGAAGATGATCATGATGGCC
>SRR4252610_12932014___Sbg4
CCCCACTAGTAGGTACTATTTACATGACT
>SRR4252608_10157535___Sbg4
ACCTCCGAGACAACACGAACATATAGACAAA
>SRR4252610_9746839___Sbg4
TATAGACAAAGACACCATTCACACCCAC
>SRR4252605_8935847___Sbg4
TTCCAAGTTTTACCTCCCCCACTAGTAGG
>SRR4252611_12325723___Sbg4
TGATGAAGATGATCATGATGGCCAGATG
>SRR4252623_7924005___Sbg4
TACTTTTTTGACTACACAAGGACATGG
>SRR4252619_1514102___Sbg4
AAGGCGAATATGATGAAGATGATTATGAT
>SRR4252610_4447453___Sbg4
TACACAAGGACATGGAGGGCCTCCCGGGA
>SRR4252606_740256___Sbg4
TGATGAAGATGATTATGATGGCCAGAT
>SRR4252616_2734367___Sbg4
ATCACATATACATTCATACTTTTTTGACTAC
>SRR4252610_12936593___Sbg4
ACCTCCGAGACAACACGAACATATAGACA
>SRR4252616_12426029___Sbg4
AAACAAGGCGAATATGATGAAGATGATTAT
>SRR4252605_12154264___Sbg4
CCCCACTAGTAGGTACTATTTACATGACC
>SRR4252610_2954185___Sbg4
CCCCACTAGTAGGTACTATTTACATGACC
>SRR4252611_7746504___Sbg4
CCCCACTAGTAGGTACTATTTACATGACC
>SRR4252605_7973076___Sbg4
CCCCACTAGTAGGTACTATTTACATGACT
>SRR4252617_3906309___Sbg4
CACGCCACCCGGGCCTTACAGAGCCAAGA
>SRR4252623_5771298___Sbg4
TGATGAAGATGATTATGATGGCCAGATGA
>SRR4252609_14604195___Sbg4
TACATTCATACTTTTTTGACTACACAAGT
>SRR4252610_1960556___Sbg4
TCCGAGACAACACGAACATATAGACAAA
>SRR4252608_4502117___Sbg4
CCCCACTAGTAGGTACTATTTACATGACT
>SRR4252610_2656687___Sbg4
ACTTTTTTGACTACACAAGGACATGGA

>SRR4252608_3198094___Sbg4
TACACAAGGACATGGAGGGCCTCCCC
>SRR4252611_5913853___Sbg4
TACACAAGGACATGGAGGGCCTCCCC
>SRR4252610_11014900___Sbg4
GGGCCTAAAGTTTCCTGACATTTGTCTTA
>SRR4252611_6427863___Sbg4
CCCCACTAGTAGGTACTATTTACATGACT
>SRR4252610_6754129___Sbg4
CCCACTAGTAGGTACTATTTACATGACT
>SRR4252610_2811844___Sbg4
CCCCACTAGTAGGTACTATTTACATGACT
>SRR4252610_4407279___Sbg4
GCCACCTCCGAGACAACACGAACATATAG
>SRR4252610_13168218___Sbg4
CCCCACTAGTAGGTACTATTTACATGACC
>SRR4252605_1470243___Sbg4
CCCACTAGTAGGTACTATTTACATGACC
>SRR4252608_8377748___Sbg4
CCCACTAGTAGGTACTATTTACATGACC
>SRR4252612_584715___Sbg4
CCCACTAGTAGGTACTATTTACATGACC
>SRR4252607_10472344___Sbg4
CATATACATTCATACTTTTTTGACTACACA
>SRR4252608_2242562___Sbg4
TACTTTTTTGACTACACAAGGACATGG
>SRR4252610_692011___Sbg4
TGACTACACAAGGACATGGAGGGCCTGC
>SRR4252612_8643729___Sbg4
TACACAAGGACATGGAGGGCCTGCCTGG
>SRR4252606_807758___Sbg4
TCCGAGACAACACGAACATATAGACAAAGA
>SRR4252621_13413151___Sbg4
CCGAGACAACACGAACATATAGACAAA
>SRR4252606_7911477___Sbg4
TCCGAGACAACACGAACATATAGACAAA
>SRR4252608_5002106___Sbg4
ACCGTTTGGGCCTAAAGTTTCCTGACATT
>SRR4252625_417610___Sbg4
CCGTTTGGGCCTAAAGTTTCCTGACATT
>SRR4252626_6747402___Sbg4
CAGGGCCACCTCCGAGACAACACGAAA
>SRR4252607_3965291___Sbg4
ACCTCCCCCACTAGTAGGTACTATTTACATG
>SRR4252612_1514902___Sbg4
GCCTCCGAGACAACACGAACATATAGAC
>SRR4252612_7145631___Sbg4
CATTCATACTTTTTTGACTACACAAGGA
>SRR4252608_7063197___Sbg4
TACACAAGGACATGGAGGGCATCCCTGG
>SRR4252610_13072355___Sbg4
TACACAAGGACATGGAGGGCATCCCTGGA
>SRR4252620_3507269___Sbg4
AAAACCACAGAAAAACCTCACCCAGGAAA
>SRR4252607_9705628___Sbg4
ATTCATACTTTTTTGACTACACAAGGA
>SRR4252611_14518605___Sbg4
TAAACAAGGCGAATATGATGAAGATGAT
>SRR4252611_11679121___Sbg4
TACTTTTTTGACTACACACGGACATGG
>SRR4252606_1875952___Sbg4
TCCGAGACAACACGAACATGTAGACAAAG
>SRR4252624_2670980___Sbg4
TACACAAGGACATGGAGGGCCTCCCTGGA
>SRR4252611_2026133___Sbg4
CCGTTTGGGCCTAAAGTTTCCTGACATT
>SRR4252610_9707070___Sbg4
CCCCACTAGTAGGTACTATTTACATGACT
>SRR4252607_1543009___Sbg4
AACCGAGACAACACGAACATATAGACAAA

>SRR4252607_7329393___Sbg4
AACCGAGACAACACGAACATATAGACAA
>SRR4252612_3870209___Sbg4
AACCGAGACAACACGAACATATAGACAAA
>SRR4252605_10318762___Sbg4
TAAAGTTTCCTGACATTTGTCTTATGG
>SRR4252618_1279344___Sbg4
ACATTCATACTTTTTTGACTACACAAGGA
>SRR4252610_9113958___Sbg4
CCCACTAGTAGGTACTATTTACATGACT
>SRR4252623_5779970___Sbg4
TACACAAGGACATGGAGGGCCTCCCTGG
>SRR4252614_507503___Sbg4
CACCTCCGAGACAACACGAACATATAGA
>SRR4252618_5765109___Sbg4
TCCAAGTTTTACCTCCCCCACTAGTAGG
>SRR4252611_8732566___Sbg4
CCCCACTAGTAGGTACTATTTACATGACT
>SRR4252610_9278281___Sbg4
CCCACTAGTAGGTACTATTTACATGACT
>SRR4252609_9566172___Sbg4
CCGAGACAACACGAACATATAGACAAAGA
>SRR4252612_1280149___Sbg4
CCCCACTAGTAGGTACTATTTACATGAAT
>SRR4252610_158449___Sbg4
CCCACTAGTAGGTACTATTTACATGAAT
>SRR4252609_6874813___Sbg4
TACACAAGGACATGGAGGGCCTCCCTGGAA
>SRR4252610_3883357___Sbg4
TACACAAGGACATGGAGGGCCTCCCTGGAA
>SRR4252614_2237503___Sbg4
ACCTCCGAGACAACACGAACATATAG
>SRR4252611_12103691___Sbg4
AGACCTGTCGGGAGAGGATAGACTATG
>SRR4252605_11150276___Sbg4
AGCCAGATGATGTCTGGGGACCGTTTGG
>SRR4252610_9107020___Sbg4
CCCCACTAGTAGGTACTATTTACATGACT
>SRR4252609_3971178___Sbg4
CGGGAGAGGATAGACTATGGATCACA
>SRR4252611_10738038___Sbg4
TCCGAGACAACACGAACATATAGACAAAG
>SRR4252611_10739190___Sbg4
TATGATGAAGATGATTATGATGGCCAGAT
>SRR4252622_5385185___Sbg4
ATTTACATGACTCACGCCACCCGGCA
>SRR4252611_9666214___Sbg4
TACACAAGGACATGGAGGGCCTCCCTGGA
>SRR4252605_8131314___Sbg4
GATGAAGATGATCATGATGGCCAGAT
>SRR4252605_13706556___Sbg4
CCCCACTAGTAGGTACTATTTACATGACT
>SRR4252610_8237546___Sbg4
CCCCACTAGTAGGTACTATTTACATGACA
>SRR4252611_3053923___Sbg4
CCCACTAGTAGGTACTATTTACATGACA
>SRR4252605_13949042___Sbg4
TACACAAGGACATGGAGGGCCTCCTT
>SRR4252605_11546278___Sbg4
ACCTCCGAGACAACACGAACATATAGACAAA
>SRR4252608_8903357___Sbg4
TCCGAGACAACACGAACATATAGACAAAT
>SRR4252607_4944579___Sbg4
ATTCATTCATACTTTTTTGACTACAC
>SRR4252605_7151285___Sbg4
TCATTCATACTTTTTTGACTACACAAGGA
>SRR4252605_10390825___Sbg4
TACACAAGGACATGGAGGGCCTCCCTGG
>SRR4252610_9562562___Sbg4
GAAGATGATCATGATGGCCAGATGATGT

>SRR4252611_5636774___Sbg4
TCCAAGTTTTACCTCCCCCACTAGTAGG
>SRR4252610_2154160___Sbg4
TCCAAGTTTTACCTCCCCCACTAGTAGGT
>SRR4252612_4066397___Sbg4
TGAAGATGATTATGATGGCCAGATGA
>SRR4252610_9321331___Sbg4
GAACAAGGCGAATATGATGAAGATGAT
>SRR4252612_9176546___Sbg4
GGCGAGTATGATGAAGATGATTATGATGGC
>SRR4252607_10184133___Sbg4
TATGATGAAGATGATTATGATGGCCAGATG
>SRR4252623_6654572___Sbg4
TGATGAAGATGATTATGATGGCCAGATGA
>SRR4252608_1128895___Sbg4
TACACAAGGACATGGAGGGCCTCCCA
>SRR4252611_10485312___Sbg4
ACCGTTTGGGCCTAAAGTTTCCTGACATT
>SRR4252626_6938426___Sbg4
ACATTCATACTTTTTTGACTACACAAGGA
>SRR4252606_5823239___Sbg4
TCCGAGACAACACGAACATGTAGACAAAG
>SRR4252609_386654___Sbg4
TTTTTGACTACACAAGGACAAGGAGG
>SRR4252607_14438480___Sbg4
AGACCGTTTGGGCCTAAAGTTTCCTGACATT
>SRR4252619_3604407___Sbg4
AACGCCACCCGGGCCTTACAGAGCCAAGA
>SRR4252613_41312___Sbg4
AACTCAATGCCAGGGCCACCTCCGAG
>SRR4252607_1300086___Sbg4
CCGAGACAACACGAACATATAGACAAA
>SRR4252611_6233479___Sbg4
ATTCATACTTTTTTGACTACACAAGGA
>SRR4252605_2847775___Sbg4
TCCGAGACAACACGAACATGTAGACAA
>SRR4252606_10248555___Sbg4
TCCGAGACAACACGAACATGTAGACAAAG
>SRR4252610_3291814___Sbg4
TCCGAGACAACACGAACATGTAGACAAAG
>SRR4252610_844452___Sbg4
TCCGAGACAACACGAACATGTAGACAAAG
>SRR4252608_2364668___Sbg4
TCCATTCCATTTTCCAAGTTTTACCTCCT
>SRR4252616_475955___Sbg4
TCATACTTTTTTGACTACACAAGGACATG
>SRR4252605_15055756___Sbg4
GCTCAATGCCAGGGCCACCTCCGAGACAA
>SRR4252612_4824656___Sbg4
AGGGCCACCTCCGAGACAACACGAACATA
>SRR4252611_12560822___Sbg4
CCCACTAGTAGGTACTATTTACATGACT
>SRR4252611_539919___Sbg4
TACACAAGGACATGGAGGACCTCCCT
>SRR4252612_6000566___Sbg4
TACACAAGGACATGGAGGACCTCCCTGGA
>SRR4252610_11384450___Sbg4
ACCGTTTGGGCCTAAAGTTTCCTGACATT
>SRR4252611_8301649___Sbg4
TATGATGAAGATGATTATGATGGCCAGAT
>SRR4252609_917988___Sbg4
TACACAAGGACATGGAGGGCCTCCCTGGAT
>SRR4252625_6922085___Sbg4
TGAAGATGATCATGATGGCCAGATGATGT
>SRR4252605_7934894___Sbg4
TACACAAGGACATGGAGGGCCTCCCTCGAT
>SRR4252609_3207964___Sbg4
TACACAAGGACATGGAGGGCCTCCCTCGA
>SRR4252610_2895085___Sbg4
CTCCGAGACAACACGAACATATAGACAAA

>SRR4252610_9704081___Sbg4
CCCACTAGTAGGTACTATTTACATGACTA
>SRR4252612_6556834___Sbg4
GAGCCAAGACCTGTCGGGAGAGGATA
>SRR4252619_10340560___Sbg4
ATCAGCTCAATGCCAGGGCCACCTCCGAGA
>SRR4252610_7630301___Sbg4
CCCCACTAGTAGGTACTATTTACATGACT
>SRR4252622_1043489___Sbg4
TCCGAGACAACACGAACACATAGACAAAA
>SRR4252610_2473753___Sbg4
TAGGTACTATTTACATGACTCACGCCACCT
>SRR4252611_1542940___Sbg4
TTCCATTCCATTTTCCAAGTTTTACCT
>SRR4252609_11266758___Sbg4
CATATAGACAAAGACACCATTCACACCC
>SRR4252605_3069212___Sbg4
CCCCACTAGTAGGTACTATTTACATGACT
>SRR4252610_11542135___Sbg4
CCCCACTAGTAGGTACTATTTACATGACT
>SRR4252615_6809667___Sbg4
TACACAAGGACATGGAGGGCTTCCCTG
>SRR4252611_1284192___Sbg4
GACAACACGAACATATAGACAAAGACACC
>SRR4252612_2696448___Sbg4
ACTAGTAGGTACTATTTACATGACTCACGCT
>SRR4252610_10737581___Sbg4
CCCCACTAGTAGGTACTATTTACATGACT
>SRR4252610_12620757___Sbg4
CCCACTAGTAGGTACTATTTACATGACT
>SRR4252605_3099512___Sbg4
GACAACACGAACATATAGACAAAGACACT
>SRR4252620_7642446___Sbg4
CGAATATGATGAAGATGATTATGATGGCC
>SRR4252611_3117365___Sbg4
TATAGACAAAGACACCATTCACACCCAC
>SRR4252612_4435397___Sbg4
TATGATGAAGATGATTATGATGGCCAGAT
>SRR4252623_7424425___Sbg4
TTCCATTTTCCAAGTTTTACCTCCCCCACT
>SRR4252609_1741928___Sbg4
TACTATTTACATGACTCACGCCACTCGG
>SRR4252610_13543331___Sbg4
TATACATTCACACTTTTTTGACTACACAA
>SRR4252608_1845083___Sbg4
ACATTCACACTTTTTTGACTACACAAGGA
>SRR4252609_4969811___Sbg4
CATTCACACTTTTTTGACTACACAAGGA
>SRR4252610_2074607___Sbg4
TCCGAGACAACACGAACATATAGACAAAG
>SRR4252610_9322567___Sbg4
TCCGAGACAACACGAACATATAGACAAAGT
>SRR4252606_4075849___Sbg4
CTAGTAGGTACTATTTACATGACTCACGT
>SRR4252606_5068867___Sbg4
TGAAGATGATCATGATGGCTAGATGATG
>SRR4252607_7095501___Sbg4
TGAAGATGATCATGATGGCTAGATGATG
>SRR4252610_2011521___Sbg4
TGAAGATGATCATGATGGCTAGATGATGT
>SRR4252610_7412850___Sbg4
TGAAGATGATCATGATGGCTAGATGATG
>SRR4252611_11241091___Sbg4
TGAAGATGATCATGATGGCTAGATGATGT
>SRR4252612_275338___Sbg4
TGAAGATGATCATGATGGCTAGATGATGT
>SRR4252607_5522080___Sbg4
GCTCAATGCCAGGGCCACCTCCGAGACAA
>SRR4252608_2951225___Sbg4
CCCCACTAGTAGGTACTATTTACTTGACT

>SRR4252605_4797506___Sbg4
ACTCAATGCCAGGGCCACCTCCGAGACAA
>SRR4252608_9025345___Sbg4
ACTCAATGCCAGGGCCACCTCCGAGACAA
>SRR4252612_8002972___Sbg4
ACTCAATGCCAGGGCCACCTCCGAGACAA
>SRR4252616_4625692___Sbg4
ATTTACATGACTCACGCCACCCGGGAC
>SRR4252605_5629411___Sbg4
ACCTCCGAGACAACACGAACATATAGACAAA
>SRR4252610_1729118___Sbg4
TGACTACACAAGGACATGGAGGGCCTCCA
>SRR4252610_2314427___Sbg4
TACACAAGGACATGGAGGGCCTCCCT
>SRR4252617_10327177___Sbg4
ACCTGTCGGGAGAGGATAGACTATGGATC
>SRR4252605_10405453___Sbg4
CCAGGGCCACCTCCGAGACAACACGAAAAA
>SRR4252608_9733822___Sbg4
TACTTTTTTGACTACACATGGACATGG
>SRR4252605_14124240___Sbg4
ACCTCCGAGACAACACGAACATATAGACAAA
>SRR4252619_1500184___Sbg4
ACATTCATACTTTTTTGACTACACAAGGA
>SRR4252605_1320371___Sbg4
TACTTTTTTGACTACACAAGGACATGG
>SRR4252607_13692341___Sbg4
CCCCACTAGTAGGTACTATTTACATGACT
>SRR4252605_8377698___Sbg4
ATATGATGAAGATGATTATGATGGCCAGA
>SRR4252612_6764233___Sbg4
ACACATTCATACTTTTTTGACTACAC
>SRR4252610_6065773___Sbg4
AGGCCTAAAGTTTCCTGACATTTGTCTTA
>SRR4252605_13646181___Sbg4
GCTCAATGCCAGGGCCACCTCCGAGACAA
>SRR4252607_7843295___Sbg4
ACATTCATACTTTTTTGACTACACAAGGA
>SRR4252610_10399024___Sbg4
GCCTCCGAGACAACACGAACATATAGAC
>SRR4252610_9110760___Sbg4
GCCTCCGAGACAACACGAACATATAGACAAA
>SRR4252611_3090340___Sbg4
TACACAAGGACATGGAGGGACTCCCTGGAT
>SRR4252623_1132325___Sbg4
TACACAAGGACATGGAGGGACTCCCTGGA
>SRR4252616_2503392___Sbg4
CAGGGCCACCTCCGAGACAACACGAACATA
>SRR4252612_4225578___Sbg4
TACACAAGGACATGGAGGGCCTCCCTGGAT
>SRR4252610_9742619___Sbg4
CCCACTAGTAGGTACTATTTACATGACG
>SRR4252608_6793455___Sbg4
TCCGAGACAACACGAACATATAGACG
>SRR4252609_12831555___Sbg4
TACATTCATACTTTTTTGACTACACAAGG
>SRR4252612_5589011___Sbg4
TCATGATGGCCAGATGATGTCTGGGGACC
>SRR4252611_6153648___Sbg4
CTAGTAGGTACTATTTACATGACTCACGC
>SRR4252605_12129733___Sbg4
TATAGACAAAGACACCATTCACACCCACA
>SRR4252621_2178746___Sbg4
CGAGACAACACGAACATACAGACAAAGA
>SRR4252609_8382850___Sbg4
GCTCAATGCCAGGGCCACCTCCGAGACAA
>SRR4252608_6703526___Sbg4
TCCCCCACTAGTAGGTACTATTTACATGA
>SRR4252607_4490467___Sbg4
ACATTCATACTTTTTTGACTACACAAGGA

>SRR4252623_5890954___Sbg4
TGACTACACAAGGACATGGAGGGCCTCCCT
>SRR4252611_15002016___Sbg4
CACGGAGGGCCTCCCTGGATAAGTGATCA
>SRR4252610_8348696___Sbg4
CTCCGAGACAACACGAACATATAGACAA
>SRR4252609_14992268___Sbg4
TACTTTTTTGACTACACAAGGACATGG
>SRR4252607_6488243___Sbg4
TAGGGCCACCTCCGAGACAACACGAA
>SRR4252608_12941435___Sbg4
CCCACTAGTAGGTACTATTTACATGACT
>SRR4252618_2084313___Sbg4
GGTCGGGAGAGGATAGACTATGGATCACA
>SRR4252616_15838813___Sbg4
GGTCACCTCCGAGACAACACGAACATA
>SRR4252610_10788930___Sbg4
ACGTTCATACTTTTTTGACTACACAAGGA
>SRR4252610_8408488___Sbg4
ACGTTCATACTTTTTTGACTACACAAGGA
>SRR4252610_1497432___Sbg4
CGTTCATACTTTTTTGACTACACAAGGA
>SRR4252606_392403___Sbg4
GTTCATACTTTTTTGACTACACAAGGA
>SRR4252607_6895277___Sbg4
GTTCATACTTTTTTGACTACACAAGGA
>SRR4252610_1477878___Sbg4
GTTCATACTTTTTTGACTACACAAGGACAT
>SRR4252606_9661163___Sbg4
CCCCACTAGTAGGTACTATTTACATGACT
>SRR4252624_5949238___Sbg4
CCCCACTAGTAGGTACTATTTACATGACT
>SRR4252606_1641102___Sbg4
TCCGAGACAACACGAACATATAGACAAAGC
>SRR4252616_15765103___Sbg4
CCGAGACAACACGAACATATAGACAAA
>SRR4252618_9631516___Sbg4
TGATGTCTGGGGACCGTTTGGGCCTAAA
>SRR4252618_9775413___Sbg4
GGGTCTTACAGAGCCAAGACCTGTCGGGA
>SRR4252619_3603381___Sbg4
ATATACATTCATACTTTTTTGACTACACA
>SRR4252605_4387428___Sbg4
TACACAAGGACATGGAGGGCCTCCCTGGA
>SRR4252611_3400946___Sbg4
TATGATGAAGATGATCATGATGGCCAGATG
>SRR4252611_9840797___Sbg4
ACCTGTCGGGAGAGGATAGACTATGGATCA
>SRR4252611_3384938___Sbg4
CTCCGAGACAACACGAACATATAGACAAA
>SRR4252611_10256128___Sbg4
GCTCAATGCCAGGGCCACCTCCGAGACAA
>SRR4252605_4097079___Sbg4
CCCCACTAGTAGGTACTATTTACATGACG
>SRR4252608_12500620___Sbg4
CCCCACTAGTAGGTACTATTTACATGACG
>SRR4252605_7062842___Sbg4
ACCGTTTGGGCCTAAAGTTTCCTGACAT
>SRR4252610_11242607___Sbg4
TCCAAGTTTTACCTCCCCCACTAGTAGG
>SRR4252612_2909872___Sbg4
TACACAAGGACATGGAGGGCCTCCCTGGA
>SRR4252610_2841531___Sbg4
TACACAAGGACATGGAGGGCCTCCCT
>SRR4252611_10780293___Sbg4
TGAAGATGATCATGATGGCCAGATGATGT
>SRR4252606_4575628___Sbg4
ACCGTTTGGGCCTAAAGTTTCCTGACA
>SRR4252611_4926268___Sbg4
GAGACAACACGAACATACAGACAAAGA

>SRR4252610_11768734___Sbg4
TGAAGATGATCATGATGGCCAGATGGTGT
>SRR4252623_5045653___Sbg4
TGAAGATGATCATGATGGCCAGATGGTGT
>SRR4252605_6773698___Sbg4
TCCGAGACAACACGAACATAAAGACAAAG
>SRR4252609_5244035___Sbg4
TCCGAGACAACACGAACATAAAGACAAA
>SRR4252609_9125117___Sbg4
TCCGAGACAACACGAACATAAAGACAAAG
>SRR4252612_368588___Sbg4
TCCGAGACAACACGAACATAAAGACAAAG
>SRR4252610_10626788___Sbg4
TTTGACTACACAAGGACATGGAGGGCA
>SRR4252606_5043450___Sbg4
TGATGAAGATGATTATGATGGCCAGATGA
>SRR4252613_478921___Sbg4
ACCGCTTGGGCCTAAAGTTTCCTGACATT
>SRR4252609_9133742___Sbg4
CCGAGACAACACGAACATATAGACAAAG
>SRR4252625_14224271___Sbg4
TGCCAGGGCCACCTCCGAGACAACACGA
>SRR4252608_7272634___Sbg4
ACCGTTTGGGCCTAAAGTTTCCTGACA
>SRR4252624_2393634___Sbg4
ATGATGTCTGGGGACCGTTTGGGCCC
>SRR4252624_2907604___Sbg4
TCCGAGACAACACGAACATATAGACATAG
>SRR4252608_3974862___Sbg4
ACATTCATACTTTTTTGACTACACAAGGA
>SRR4252623_5051572___Sbg4
TACACAAGGACATGGAGGGCCTCCCTG
>SRR4252612_7901964___Sbg4
TACACAAGGACATGGAGGGCCTCCCTGG
>SRR4252624_755821___Sbg4
GTTCAATGCCAGGGCCACCTCCGAGACAA
>SRR4252605_9316429___Sbg4
TCCCCCACTAGTAGGTACTATTTACATGA
>SRR4252626_8594298___Sbg4
TCCCCCACTAGTAGGTACTATTTACATGAC
>SRR4252610_5293620___Sbg4
CCCCACTAGTAGGTACTATTTACATGACT
>SRR4252606_7397237___Sbg4
GCTCAATGCCAGGGCCACCTCCGAGACAA
>SRR4252612_7914063___Sbg4
TCCGAGACAACACGAACATGTAGACAAA
>SRR4252610_5305422___Sbg4
TAGACAAAGACACCATTCACACCCACA
>SRR4252607_10269314___Sbg4
ACATACATACTTTTTTGACTACACAAGGA
>SRR4252606_9295083___Sbg4
TAGACAAAGACACCATTCACACCCACA
>SRR4252619_9021773___Sbg4
TATTTACATGACTCACGCCACCCGGGCCT
>SRR4252612_5346249___Sbg4
TATACATTCATACTTTTTTGACTACACAA
>SRR4252609_15755640___Sbg4
ACATTCATACTTTTTTGACTACACAAGGA
>SRR4252607_10784059___Sbg4
TACCAACAAGGCGAATATGATGAAGATG
>SRR4252612_8898670___Sbg4
TATGATGAAGATGATTATGATGGCCAGGT
>SRR4252607_11777304___Sbg4
TTCATACTTTTTTGACTATACAAGGACA
>SRR4252610_2976796___Sbg4
TTCCAAGTTTTACCTCCCCCACTAGTAG
>SRR4252610_11301312___Sbg4
TCCAAGTTTTACCTCCCCCACTAGTAGT
>SRR4252619_12441051___Sbg4
CATATACATTCATACTTTTTTGACTACA

>SRR4252606_5851877___Sbg4
TACACAAGGACATGGAGGGTCTCCCTGGT
>SRR4252608_10605019___Sbg4
TACACAAGGACATGGAGGGTCTCCCTGGT
>SRR4252608_12884961___Sbg4
TACACAAGGACATGGAGGGTCTCCCTGGT
>SRR4252610_6609703___Sbg4
TAGGTACTATTTACATGACTCACGCCACCC
>SRR4252611_5488282___Sbg4
TAGGTACTATTTACATGACTCACGCCAT
>SRR4252608_5126064___Sbg4
TACACAAGGACATGGAGGGCCTCCCTGG
>SRR4252619_14879964___Sbg4
TAGACTATGGATCACATATACATTCAT
>SRR4252610_6170930___Sbg4
CTCCGAGACAACACGAACATATAGACAA
>SRR4252612_5489808___Sbg4
TAGGTACTATTTACATGACTCATGCCAT
>SRR4252610_11973431___Sbg4
GCTCAATGCCAGGGCCACCTCCGAGACA
>SRR4252611_9670635___Sbg4
CATTCATACTTTTTTGACTACACAAGGA
>SRR4252606_2256066___Sbg4
TACACAAGGACATGGAGGGCCTCCCC
>SRR4252623_11582496___Sbg4
AGGCCTTACAGAGCCAAGACCTGTCGGGA
>SRR4252612_4172729___Sbg4
TGATCATGATGGCCAGATGATGTCTGGG
>SRR4252606_7873087___Sbg4
ACGTTTGGGCCTAAAGTTTCCTGACATTT
>SRR4252611_8043489___Sbg4
ACGTTTGGGCCTAAAGTTTCCTGACATTTGT
>SRR4252609_1540251___Sbg4
TACACAAGGACATGGAGGGCATCCCTGGA
>SRR4252610_9202626___Sbg4
TACACAAGGACATGGAGGGCATCCCTGGA
>SRR4252619_4383019___Sbg4
ACCGTTTTGGCCTAAAGTTTCCTGACAT
>SRR4252606_8535883___Sbg4
TACACAAGGACATGGAGGGCCTCCCT
>SRR4252606_377483___Sbg4
CCCCACTAGTAGGTACTATTTACATGACT
>SRR4252618_1109954___Sbg4
TCACGCCACCCGGGCCTTACAGAGCCAAGA
>SRR4252623_14637198___Sbg4
TCCAAGTTTTACCTCCCCCATTAGTAGGT
>SRR4252610_1881320___Sbg4
ACATTCATACTTTTTTGACTACACAAGGA
>SRR4252610_10116769___Sbg4
TGAAGATGATCATGATGGCCAGATGATGT
>SRR4252605_11892238___Sbg4
TGAAGATGATTATGATGGCCAGATGTTGT
>SRR4252610_1897015___Sbg4
TGAAGATGATTATGATGGCCAGATGTTG
>SRR4252611_14759296___Sbg4
TGAAGATGATTATGATGGCCAGATGT
>SRR4252613_2955379___Sbg4
CACGCCACCCGGGCCTTACAGAGCCAAGA
>SRR4252607_9348270___Sbg4
TACTTTTTTGACTACACAAGGACATGG
>SRR4252615_161331___Sbg4
CTGTCTGGAGAGGATAGACTATGGAT
>SRR4252611_4535345___Sbg4
TACACAAGGACATGGAGGACCTCCCT
>SRR4252609_8632194___Sbg4
CATACTTTTTTGACTACACAAGGACATGG
>SRR4252605_8397652___Sbg4
TCCGAGACAACACGAACATGTAGACAAAG
>SRR4252610_9484423___Sbg4
TCCGAGACAACACGAACATGTAGACA

```
>SRR4252605_15143792___Sbg4
TGAAGATGATCATGATGGCCAGATGACGT
>SRR4252610_11114552___Sbg4
TGACTACACAAGGACATGTAGGGCCTCC
>SRR4252610_12952049___Sbg4
CCCACTAGTAGGTACTATTTACATGACTC
>SRR4252611_13276373___Sbg4
TGAAGATGATCATGATGGCCAGATGATG
>SRR4252623_7198251___Sbg4
TGATGAAGATGATCATGATGGCCAGATG
>SRR4252605_288539___Sbg4
TGAAGATGATCATGATGGCCAGATGAT
>SRR4252608_12107764___Sbg4
CCCCACTAGTAGGTACTATTTACATGACT
>SRR4252606_354946___Sbg4
TCCAAGTTTTACCTCCCCCACTAGTT
>SRR4252623_14555678___Sbg4
TCTCAATGCCAGGGCCACCTCCGAGACAA
>SRR4252609_12758210___Sbg4
GCTCAATGCCAGGGCCACCTCCGAGACAA
>SRR4252621_5453544___Sbg4
ACGCCACCCGGGCCTTACAGAGACAAGA
>SRR4252616_6021449___Sbg4
TCATACTTTTTTGACTACACAAGGACAT
>SRR4252613_9009217___Sbg4
TTTTTGACTACACAAGGACATGGAGGGC
>SRR4252611_10909587___Sbg4
CCCCACTAGTAGGTACTATTTACATGACT
>SRR4252609_2238416___Sbg4
TATACATTCATACTTTTTTGACTACACA
>SRR4252621_2554509___Sbg4
ACACGAACATATAGACAAAGACACCATT
>SRR4252610_6321317___Sbg4
TGAAGATGATCATGATGGCCAGATGATGT
>SRR4252607_7993635___Sbg4
CCCACTAGTAGGTACTATTTACATGAATC
>SRR4252607_11121383___Sbg4
ACCTCCGAGACAACACGAACATATAGACA
>SRR4252612_3903210___Sbg4
CTCCGAGACAACACGAACATATAGACA
>SRR4252621_12570569___Sbg4
TCCGAGACAACACGAACATATAGACAAAG
>SRR4252622_197187___Sbg4
TATTCATACTTTTTTGACTACACAAGGA
>SRR4252605_7970198___Sbg4
GCTCAATGCCAGGGCCACCTCCGAGACAA
>SRR4252607_4501895___Sbg4
GCTCAATGCCAGGGCCACCTCCGAGACAA
>SRR4252609_9445690___Sbg4
TATGATGAAGATGATTATGATGGCCAG
>SRR4252617_10553912___Sbg4
GGTTCACATATACATTCATACTTTTTT
>SRR4252610_7595100___Sbg4
ATGAAGATGATTATGATGGCCAGATGA
>SRR4252606_5617145___Sbg4
CATATACATTCATACTTTTTTGACTACACA
>SRR4252605_1550088___Sbg4
CATTCATTCTTTTTTGACTACACAAGGA
>SRR4252610_8361040___Sbg4
TACACAAGGACATGGAGGGTCTCCCTGG
>SRR4252610_720331___Sbg4
TATGATGAAGATGATTATGATGGCCAGAT
>SRR4252620_1454069___Sbg4
ATACATTCATACTTTTTTGACTACACAA
>SRR4252605_4110766___Sbg4
TCAGCTCAATGCCAGGGCCACCTCCGAT
>SRR4252622_8742288___Sbg4
TTACAGAGACAAGACCTGTCGGGAGAGGATA
>SRR4252610_4704213___Sbg4
TATAGACAAAGACACCATTCACACCCACAC
```

>SRR4252612_2433111___Sbg4
ACCGTTTGGGCCTAAAGTTCCTGACA
>SRR4252610_4552046___Sbg4
TCCAAGTTTTACCTCCCCCACTAGTAGGT
>SRR4252607_2086647___Sbg4
TGAAGATGATCATGATGGCCAGATGATGC
>SRR4252609_10034214___Sbg4
TGAAGATGATCATGATGGCCAGATGATGC
>SRR4252610_1437145___Sbg4
TGAAGATGATCATGATGGCCAGATGATGC
>SRR4252613_9255850___Sbg4
TGAAGATGATCATGATGGCCAGATGATGC
>SRR4252624_1332885___Sbg4
CCCCACTAGTAGGTACTATTTACATGACT
>SRR4252625_3000090___Sbg4
TAGGTACTATTTACATGACTCACGTCACCC
>SRR4252608_3593872___Sbg4
TGCCAGGGCCACCTCCGAGACAACACGA
>SRR4252624_10788308___Sbg4
AACAAGGCGAATATGATGAAGATGATCA
>SRR4252607_14876929___Sbg4
TGAAGATGATCATGATGGCCAGATGATGT
>SRR4252610_10752957___Sbg4
TCCAAGTTTTACCTCCCCCACTAGTAGGT
>SRR4252622_10639898___Sbg4
TGAAGATGATCATGATGGCCAGATGATGT
>SRR4252614_2538298___Sbg4
ACATATACATTCATACTTTTTTGACTACA
>SRR4252610_630948___Sbg4
TAAGTGATCAACTCAATGCCAGGGCCACC
>SRR4252612_7039001___Sbg4
ACTCAATGCCAGGGCCACCTCCGAGACAA
>SRR4252605_6382428___Sbg4
AGACAACACGAACATATAGACAAAGACA
>SRR4252610_13090345___Sbg4
CCCCACTAGTAGGTACTATTTACATGACT
>SRR4252606_6247142___Sbg4
ACATTCATACTTTTTTGACTACACAAGGA
>SRR4252611_5145368___Sbg4
CGGGAGAGGATAGACTATGGATCACATA
>SRR4252611_8566204___Sbg4
ACATTCATACTTTTTTGACTACACAAGGA
>SRR4252610_10296527___Sbg4
TGATGAAGATGATTATGATGGCCGGATG
>SRR4252605_9681834___Sbg4
TGAAGATGATTATGATGGCCGGATGAT
>SRR4252606_4812437___Sbg4
TGAAGATGATTATGATGGCCGGATGAT
>SRR4252610_10614135___Sbg4
TGAAGATGATTATGATGGCCGGATGAT
>SRR4252608_10683326___Sbg4
TCCCCCACTAGTAGGTACTATTTACATGA
>SRR4252605_14618979___Sbg4
GGAGAGGATAGACTATGGATCACATAC
>SRR4252613_7492818___Sbg4
TGACTACACAAGGACATGGAGGGCCTCCCT
>SRR4252606_6617649___Sbg4
TACACAAGGACATGGAGGGCCTCCCTT
>SRR4252612_2698018___Sbg4
TACACAAGGACATGGAGGGCCTCCCTTC
>SRR4252607_7026894___Sbg4
TGACTACACAAGGACATGGAGAGCCTCC
>SRR4252606_1885246___Sbg4
TGATGTCTGGGGACCGTTTGGGCCTAAAG
>SRR4252612_8304315___Sbg4
CCCCACTAGTAGGTACTATTTACATGACT
>SRR4252616_14174174___Sbg4
TCACGCCACCCGGGCCTTACAGAGCCAAGA
>SRR4252620_7263578___Sbg4
TACAGAGCCAAGACCTGTCGGGAGAGGAT

```
>SRR4252623_14321172___Sbg4
TTTCGAAGTTTTACCTCCCCCACTAGTA
>SRR4252610_10219662___Sbg4
TAGGTACTATTTACATGACTCACGCCACC
>SRR4252610_8655906___Sbg4
CCCACTAGTAGGTACTATTTACACGACT
>SRR4252611_4640805___Sbg4
TACACAAGGACATGGAGGGCCTCCCT
>SRR4252610_1653205___Sbg4
TGAAGATGATCATGATGGCCAGATGATGC
>SRR4252611_8161752___Sbg4
ATATGATGAAGATGATTATGATGACCAG
>SRR4252610_8328167___Sbg4
TTTTGACTACACAAGGACATGGAGGGC
>SRR4252607_9970707___Sbg4
ACCGTTTGGGCCTAAAGTTTCCTGACATT
>SRR4252605_375619___Sbg4
TCCATTTTCCAAGTTTTACCTCCCCCACT
>SRR4252610_8415211___Sbg4
TCCGAGACAACACGAACATATAGACAAA
>SRR4252605_7785286___Sbg4
ATGGAGAGCCTCCCTGGATAAGTGATCA
>SRR4252609_2714831___Sbg4
GCTCAATGCCAGGGCCACCTCCGAGACAA
>SRR4252605_13220042___Sbg4
CTGTCGGGAGAGGATAGACTATGGATT
>SRR4252606_5320082___Sbg4
TTTTTGACTACACAAGGACATGGAGG
>SRR4252611_8214065___Sbg4
TACTTTTTTGACTACACAAGGACATGA
>SRR4252612_6882711___Sbg4
TGAAGATGATTATGATGGCCAGATGAT
>SRR4252605_2249902___Sbg4
CCCACTAGTAGGTACTATTTACATGACT
>SRR4252612_6046164___Sbg4
TGACTACACAAGGACATGGAGGACCT
>SRR4252611_14398089___Sbg4
TACACAAGGACATGGAGGGCCTCCCTGG
>SRR4252609_16998503___Sbg4
TACTTTTTTGACTACACAAGGACATGG
>SRR4252613_656508___Sbg4
TGACTACACAAGGACATGGAGGGCCTG
>SRR4252612_4534310___Sbg4
ACCGTTTGGGCCTAAAGTTTCCTGACATT
>SRR4252607_8241136___Sbg4
AAATTTTTTGACTACACAAGGACATGGA
>SRR4252608_6065029___Sbg4
TCCAAGTTTTACCTCCCCCACTAGTAGGT
>SRR4252607_11863801___Sbg4
GCTCAATGCCAGGGCCACCTCCGAGACAA
>SRR4252613_4469804___Sbg4
TCCAAGACAACACGAACATATAGACAAA
>SRR4252611_12833186___Sbg4
GATGATGTCTGGGGACCGTTTGGGCCTAAA
>SRR4252614_9233414___Sbg4
TCCGAGACAACACGAACATATAGACAAAG
>SRR4252610_4729423___Sbg4
CAAGTTATACCTCCCCCACTAGTAGGTA
>SRR4252608_3627410___Sbg4
CCCACTAGTAGGTACTATTTATATGACT
>SRR4252616_8195030___Sbg4
CAAGACCTGTCGGGAGAGGATAGACTATGGA
>SRR4252623_10370055___Sbg4
TTCCAAGTTTTACCTCCCCCACTAGTAG
>SRR4252608_10588696___Sbg4
CCCACTAGTAGGTACTATTTACATGACT
>SRR4252614_8244030___Sbg4
TGACTACACAAGGACATGGAGAGCCTCCC
>SRR4252610_12359962___Sbg4
CATTCATACTTTTTTGACTACACAAGGA
```

>SRR4252623_6211921___Sbg4
CTTTTTTGACTACACAAGGACATGGAG
>SRR4252610_2473462___Sbg4
CCTCCCTGGATAAGTGATCTGCTCAATGC
>SRR4252611_7711464___Sbg4
AAGGCGAATATGATGAAGATGATTAT
>SRR4252610_2620489___Sbg4
TAGGTACTATTTACATGACTCACGCCACC
>SRR4252606_7946700___Sbg4
TACACAAGGACATGGAGGGCCTCCCTGGT
>SRR4252610_4154221___Sbg4
TACACAAGGACATGGAGGGCCTCCCTGGT
>SRR4252610_7784031___Sbg4
TACACAAGGACATGGAGGGCCTCCCTGGT
>SRR4252612_3082238___Sbg4
CTAGTAGGTACTATTTACATGACTCACGCT
>SRR4252619_2113289___Sbg4
TACCCGGGCCTTACAGAGCCAAGACCTGTC
>SRR4252612_6314829___Sbg4
CCCACTAGTAGGTACTATTTACATGACT
>SRR4252613_7591200___Sbg4
TCCGAGACAACACGAACATATAGACGAA
>SRR4252609_3655563___Sbg4
TACATTCATACTTTTTTGACTACACAAGC
>SRR4252610_13420793___Sbg4
CCCCACTAGTAGGTACTATTTACATAACT
>SRR4252610_7936154___Sbg4
CCCACTAGTAGGTACTATTTACATAACT
>SRR4252608_10709109___Sbg4
TGGGCCTAAAGTTTCCTGACATTTGTCTTTT
>SRR4252621_13871703___Sbg4
CCCACTAGTAGGTACTATTTACATGACT
>SRR4252607_7739707___Sbg4
CATTCATACTTTTTTGACTACACAAGGA
>SRR4252622_9971884___Sbg4
TACACAAGGACATGGAGGGCCTCCCTG
>SRR4252615_3394347___Sbg4
TACACAAGGACATGGAGGGCCTCCCT
>SRR4252610_3232767___Sbg4
CCTCAATGCCAGGGCCACCTCCGAGACAA
>SRR4252610_11152775___Sbg4
CCCCACTAGTAGGTACTATTTACATGACT
>SRR4252624_8813776___Sbg4
TTGACTACACAAGGACATGGAGGGCCTCCC
>SRR4252613_3065093___Sbg4
TACACAAGGACATGGAGGGCCTCCCT
>SRR4252616_14132577___Sbg4
AGGACCACCTCCGAGACAACACGAACA
>SRR4252606_1488146___Sbg4
TACACAAGGACATGGAGGGCCTCCCT
>SRR4252605_8685357___Sbg4
TATGATGAAGATGATTATGATGGCCAGAT
>SRR4252610_941663___Sbg4
CCCCACTAGTAGGTACTATTTACATGACT
>SRR4252611_1538564___Sbg4
TGACTACACAAGGACATGGAGGGCCTCT
>SRR4252623_8548995___Sbg4
TACACAAGGACATGGAGGGCTTCCCTGGA
>SRR4252609_1782570___Sbg4
GCTCAATGCCAGGGCCACCTCCGAGACAA
>SRR4252610_3697498___Sbg4
CCCACTAGTAGGTACTATTTTCATGACT
>SRR4252610_9934622___Sbg4
CCCACTAGTAGGTACTATTTTCATGACT
>SRR4252610_6834741___Sbg4
TACATTCATACTTTTTTGACTAGACAAGGA
>SRR4252605_8679036___Sbg4
TACACAAGGACATGGAGGGCCTCCCT
>SRR4252608_8781240___Sbg4
GACACCATTCACACCCACACTCATACCAAC

>SRR4252606_6371954___Sbg4
GCTCAATGTCAGGGCCACCTCCGAGACAA
>SRR4252606_1787629___Sbg4
TACACAAGGACATGGAGGGCCTCCTT
>SRR4252611_1646878___Sbg4
TACACAAGGACATGGAGGGCCTCCTT
>SRR4252605_2625664___Sbg4
GCTCAATGCCAGGGCCACCTCCGAGACAA
>SRR4252605_71248___Sbg4
TTTGGGCCTAAAGTTTCCTGACATTT
>SRR4252609_7372877___Sbg4
TATAGACAAAGACACCATTCACACCCACA
>SRR4252610_9065269___Sbg4
CCCACTAGTAGGTACTATTTACATGACTT
>SRR4252615_11420645___Sbg4
GACCTGTCGGGAGAGGATAGACTATGG
>SRR4252605_1959049___Sbg4
ACATTCTTACTTTTTTGACTACACAAGGA
>SRR4252609_13262832___Sbg4
CATTCTTACTTTTTTGACTACACAAGGA
>SRR4252609_2323914___Sbg4
TACATTCATACTTTTTTGACTACACAAGG
>SRR4252625_12517954___Sbg4
CATTCATACTTTTTTGACTACACAAGGA
>SRR4252609_9314141___Sbg4
CATACTTTTTTGACTACACAAGGACATGG
>SRR4252611_7483386___Sbg4
TCCGAGACAACACGAACATATAGACAAAGA
>SRR4252610_2291989___Sbg4
TGAAGATGATTATGATGGCCAGATGAC
>SRR4252612_8661861___Sbg4
TGGGCCTAAAGTTTCCTGACATTTGTCTTT
>SRR4252610_8669198___Sbg4
CCGATACAACACGAACATATAGACAAA
>SRR4252617_12857198___Sbg4
CAACACGAACATATAGACAAAGACACC
>SRR4252619_8589012___Sbg4
CCCCCACTAGTAGGTACTATTTACATGACT
>SRR4252622_8907903___Sbg4
CATACTTTTTTGACTACACAAGGACAT
>SRR4252605_5685539___Sbg4
TCGGGAGAGGATAGACTATGGATCACATAT
>SRR4252622_4267891___Sbg4
TACACAAGGACATGGAGGGTCTCCCTGA
>SRR4252608_7615803___Sbg4
TGATGAAGATGATCATGACGGCCAGATG
>SRR4252610_13126460___Sbg4
TTCCAAGTTTTACCTCCCCCACTAGTAGA
>SRR4252605_9764402___Sbg4
CCCCACTAGTAGGTACTATTTACATGACC
>SRR4252610_69585___Sbg4
CCCCACTAGTAGGTACTATTTACATGACC
>SRR4252608_1088680___Sbg4
CCCACTAGTAGGTACTATTTACATGACC
>SRR4252610_1187367___Sbg4
CCCACTAGTAGGTACTATTTACATGACC
>SRR4252610_3114045___Sbg4
CCCACTAGTAGGTACTATTTACATGACC
>SRR4252623_3050932___Sbg4
CCCACTAGTAGGTACTATTTACATGACC
>SRR4252619_2662294___Sbg4
TGCCAGGGCCACCTCCGAGACAACACGAA
>SRR4252606_1035098___Sbg4
AAGGCGAATATGATGAAGATGATTATGAC
>SRR4252608_7810695___Sbg4
TGTCTTATGGGTGAGGAAAAACTCCAGA
>SRR4252612_8970274___Sbg4
TAGGTACTATTTACATGACTCACGCCATT
>SRR4252610_11844695___Sbg4
CCCCACTAGTAGGTACTATTTAGATGACT

>SRR4252610_10815072___Sbg4
CCCACTAGTAGGTACTATTTACATGACT
>SRR4252607_4771422___Sbg4
TCCGAGACAACACGAACATATAGACA
>SRR4252610_4612874___Sbg4
TGAAGATGATTATGATGGCCAGATGAT
>SRR4252625_5037820___Sbg4
TGGACCATTTGGGCCTAAAGTTTCCTGACA
>SRR4252610_4169737___Sbg4
TACACAAGGACATGGAGGGCCTCCCAGG
>SRR4252611_2740744___Sbg4
TACACAAGGACATGGAGGGCCTCCCA
>SRR4252621_14007907___Sbg4
TACACAAGGACATGGAGGGCCTCCCAGG
>SRR4252619_15065844___Sbg4
CCCCACTAGTAGGTACTATTTACATGACT
>SRR4252613_7070789___Sbg4
GTTTGGGCCTAAAGTTTCCTGACATT
>SRR4252606_6699592___Sbg4
TACACAAGGACATGGAGGGCATCCCTG
>SRR4252623_5978476___Sbg4
TACACAAGGACATGGAGGGCATCCCTGG
>SRR4252605_5427410___Sbg4
CCCCACTAGTAGGTACTATTTACATGACC
>SRR4252610_6550332___Sbg4
CCCCACTAGTAGGTACTATTTACATGACC
>SRR4252610_6683658___Sbg4
CCCCACTAGTAGGTACTATTTACATGACC
>SRR4252610_9768545___Sbg4
CCCCACTAGTAGGTACTATTTACATGACC
>SRR4252610_6545455___Sbg4
CCCACTAGTAGGTACTATTTACATGACC
>SRR4252610_6867222___Sbg4
CCCACTAGTAGGTACTATTTACATGACC
>SRR4252611_2457129___Sbg4
GACCTGTCGGGAGAGGATAGACTGTGG
>SRR4252612_4567206___Sbg4
TGAGACAACACGAACATATAGACAAA
>SRR4252612_6408421___Sbg4
GACAACACGAACATATAGACAAAGACACA
>SRR4252605_5305492___Sbg4
ATGATGTCTGGGGACCGTTTGGGACT
>SRR4252623_7486176___Sbg4
GTAGGTACTATTTACATGACTCACGCCACC
>SRR4252609_4654589___Sbg4
ACTCAATGCCAGGGCCACCTCCGAGACAA
>SRR4252623_9679165___Sbg4
ACTCAATGCCAGGGCCACCTCCGAGACAA
>SRR4252611_1550486___Sbg4
CCGTTTGGGCCTAAAGTTTCCTGACATT
>SRR4252607_5366115___Sbg4
GCTCAATGCCAGGGCCACCTCCGAGACAA
>SRR4252611_1922682___Sbg4
AGACCGTTTGGGCCTAAAGTTTCCTGACA
>SRR4252612_8977706___Sbg4
TTTTCCAAGTTTTACCTCCCCCACTGGT
>SRR4252609_15844516___Sbg4
GCCGGGGCCACCTCCGAGACAACACGAACA
>SRR4252608_12740181___Sbg4
CCCCACTAGTAGGTACTATTTACATGACT
>SRR4252625_8965986___Sbg4
CCCCACTAGTAGGTACTATTTACATGACT
>SRR4252606_5993675___Sbg4
AATACATTCATACTTTTTTGACTACAC
>SRR4252612_1563780___Sbg4
CCGAGACAACACGAACATATAAACAAAGA
>SRR4252625_8562885___Sbg4
ACTGTACATTCATACTTTTTTGACTACA
>SRR4252612_3825447___Sbg4
CCCCACTAGTAGGTACTATTTACATGACT

>SRR4252609_15192759___Sbg4
CTAGTAGGTACTATTTACATGACTCACG
>SRR4252608_3840214___Sbg4
TGCCAGGGCCACCTCCGAGACAACACGA
>SRR4252611_1844013___Sbg4
TGATGTCTGGGGACCGTTTGGGCCTAAA
>SRR4252610_1022702___Sbg4
CTCCGAGACAACACGAACATATAGACA
>SRR4252609_1760382___Sbg4
GTCTGGGGACAGTTTGGGCCTAAAGTTTCCT
>SRR4252610_1272891___Sbg4
CCCACTAGTAGGTACTATTTACATGACT
>SRR4252618_8693926___Sbg4
AGCCTTACAGAGCCAAGACCTGTCGGGAGA
>SRR4252619_12997055___Sbg4
ACATTCATACTTTTTTGACTACACAAGGA
>SRR4252610_3638363___Sbg4
ATACTTTTTTGACTACACAAGGACATGG
>SRR4252610_6785897___Sbg4
ACATTCATACTTTTTTGACTACACAAGGA
>SRR4252609_179142___Sbg4
TACTTTTTTGACTACACAAGGACATGG
>SRR4252610_5007462___Sbg4
TACACAAGGACATGGAGGGCGTCCCTG
>SRR4252610_1100046___Sbg4
CCCCACTAGTAGGTACTATTTACATGACT
>SRR4252610_7042098___Sbg4
TGATGAAGATGATTATGATGGCCAGATGA
>SRR4252610_12814675___Sbg4
TCCGAGACAACACGAACATATAGACA
>SRR4252610_8627107___Sbg4
CCCCACTAGTAGGTACTATTTACCTGACC
>SRR4252611_4876930___Sbg4
TCTGGGGACCGTTTGGGCCTAAAGTGTCT
>SRR4252608_12716883___Sbg4
TATGATGAAGATGATTATGATGGCCAGATG
>SRR4252605_3389685___Sbg4
CCGAGACAACACGAACATATAGACAAA
>SRR4252617_667190___Sbg4
AGAGCCAAGACCTGTCGGGAGAGGATAGAC
>SRR4252608_9922941___Sbg4
AACCTCCGAGACAACACGAACATATAGA
>SRR4252622_7577148___Sbg4
ACAACACGAACATATAGACAAAGACACA
>SRR4252607_7673742___Sbg4
TTTCCTGACATTTGTCTTATGGGCGAGG
>SRR4252605_2798213___Sbg4
ATAGACTATGGATCACATATACATTCAT
>SRR4252607_2434788___Sbg4
CCGAGACAACACGAACATATAGACAAA
>SRR4252610_11642190___Sbg4
TAGGTACTATTTACATGACTCACGCCACCT
>SRR4252608_790740___Sbg4
TACACAAGGACATGGAGGGCCTCCCTCG
>SRR4252623_9502705___Sbg4
TACACAAGGACATGGAGGGCCTCCCTCGA
>SRR4252612_3716119___Sbg4
CAAGGACATGGAGGGCCTCCCTCGATA
>SRR4252616_12680354___Sbg4
TCCGAGACAACACGAACATATAGACAAA
>SRR4252611_3769724___Sbg4
TTTCCTGACATTTGTCTTATGGGCGA
>SRR4252611_6990820___Sbg4
TACGCCACCCGGGCCTTACAGAGCCA
>SRR4252623_993487___Sbg4
TACGCCACCCGGGCCTTACAGAGCCAAGA
>SRR4252623_1412512___Sbg4
CATTCATTCTTTTTTGACTACACAAGGA
>SRR4252610_8293811___Sbg4
TCCAAGTTTTACCTCCCCCACTAGTAGGT

>SRR4252612_4251957___Sbg4
GCCTCCGAGACAACACGAACATATAGAC
>SRR4252606_10117443___Sbg4
TGGGCCTAAAGTTTCCTGACATTTGTCTTT
>SRR4252610_106461___Sbg4
TGGGCCTAAAGTTTCCTGACATTTGTCTTT
>SRR4252623_7495393___Sbg4
CCCCACTAGTAGGTACTATTTACATGACT
>SRR4252612_3404981___Sbg4
TCAATGCCAGGGCCACCTCCGAGACAACC
>SRR4252607_12295484___Sbg4
TCTCAATGCCAGGGCCACCTCCGAGACAA
>SRR4252611_5837012___Sbg4
TACACAAGGACATGGAGGGCCTCCCTA
>SRR4252622_3552658___Sbg4
TACACAAGGACATGGAGGGCCTCCCTAGAT
>SRR4252605_9556842___Sbg4
CCCACTAGTAGGTACTATTTACATGACT
>SRR4252605_4626961___Sbg4
TCCGAGACAACACGAACATATAAACAA
>SRR4252610_12897657___Sbg4
CATTTTCCAGGTTTTACCTCCCCCACTA
>SRR4252624_3224561___Sbg4
TGACTACACAAGGACATGGAGGGCCGCCC
>SRR4252610_9122485___Sbg4
TACACAAGGACATGGAGGGCCGCCCTGGA
>SRR4252611_336717___Sbg4
TCCGAGACAACACGAACATATAGACAAAG
>SRR4252610_618089___Sbg4
TGTCGGGAGAGGATAGACTATGGATCACT
>SRR4252621_11123091___Sbg4
ACATTCATACTTTTTTGACTACACAAGGA
>SRR4252605_5185286___Sbg4
GTTTTCCAAGTTTTACCTCCCCCACTAGT
>SRR4252611_5917329___Sbg4
TTTGACTACACAAGGACATGGAGGGCCTCC
>SRR4252624_5133250___Sbg4
TATGATGAAGATGATTATGATGGCCAGAT
>SRR4252610_7593811___Sbg4
CATTCATACGTTTTTGACTACACAAGGA
>SRR4252612_5091140___Sbg4
GTCCGAGACAACACGAACATATAGACAAA
>SRR4252605_7096146___Sbg4
TGACTACACAAGGACATGTAGGGCCTCCC
>SRR4252617_7493372___Sbg4
ATCAGCTCAATGCCAGGGCCACCTCCG
>SRR4252606_8001627___Sbg4
CACATTCATACTTTTTTGACTACACAAGGA
>SRR4252612_2473089___Sbg4
TGGGCCTAAAGTTTCCTGACATTTGTCTT
>SRR4252608_7543504___Sbg4
GCCGTTTGGGCCTAAAGTTTCCTGACATT
>SRR4252622_3914374___Sbg4
GCCGTTTGGGCCTAAAGTTTCCTGACAT
>SRR4252609_8856258___Sbg4
CATTCAAACTTTTTTGACTACACAAGGA
>SRR4252609_13796058___Sbg4
CACATTCATACTTTTTTGACTACACAAGG
>SRR4252605_4486838___Sbg4
AGGGCCACCTCCGAGACAACATGAACATAT
>SRR4252620_4337530___Sbg4
AGACCGTTTGGGCCTAAAGTTTCCTGACA
>SRR4252608_12448454___Sbg4
CATTCATACTTTTTTGACTACACAAGGA
>SRR4252610_4029932___Sbg4
TCCAAGTTTTACCTCCCCCACTAGTAGGT
>SRR4252608_3412321___Sbg4
ACCTCCGAGACAACACGAACATATAGACA
>SRR4252623_6014820___Sbg4
GAGCCAAGACCTGTCGGGAGAGGATT

>SRR4252621_3903070___Sbg4
CCTCCGAGAAAACACGAACATATAGACAAA
>SRR4252605_7704770___Sbg4
CCAGGGCCACCTCCGAGACAACACGAACA
>SRR4252611_1186648___Sbg4
TTTGACTACACAAGGACATGGAGAGCCTC
>SRR4252615_13812964___Sbg4
ACATTTATACTTTTTTGACTACACAAGGA
>SRR4252610_3125773___Sbg4
TGACTACACAAGGACATGGAGGGCCTCCCC
>SRR4252611_2831723___Sbg4
TACACAAGGACATGGAGGGCCTCCCTGGA
>SRR4252606_9115196___Sbg4
TACACAAGGACATGGAGGGCCTCTCT
>SRR4252618_7893302___Sbg4
CACGCCACCCGGGCCTTACAGAGCCAAGA
>SRR4252621_13887648___Sbg4
GAATATGATGAAGATGATTAAGATGGCC
>SRR4252607_2193453___Sbg4
TACACAAGGACATGGAGGGTCTCCCTGG
>SRR4252620_11390508___Sbg4
TACTATTTACATGACTCACGCCACCAGG
>SRR4252608_12838916___Sbg4
TACACAAGGACATGGAGGGCCTCCCC
>SRR4252608_8505824___Sbg4
TACACAAGGACATGGAGGGCCTCCCC
>SRR4252608_1312790___Sbg4
TGTCTTATGGGTGAGGAAAAACTCCAGA
>SRR4252610_2054043___Sbg4
TACACAAGGACATGGAGGGCCTCCCA
>SRR4252605_9764222___Sbg4
TACACAAGGACATGGAGGGCCTCCCG
>SRR4252610_5220191___Sbg4
TACACAAGGACATGGAGGGCCTCCCGGAT
>SRR4252611_2794904___Sbg4
TACACAAGGACATGGAGGGCCTCCCGGA
>SRR4252606_8530204___Sbg4
TGAAGATGATTATGATGGCCTGATGA
>SRR4252623_174208___Sbg4
TGAAGATGATTATGATGGCCTGATGAT
>SRR4252623_6333162___Sbg4
TGAAGATGATTATGATGGCCTGATGAT
>SRR4252606_6879126___Sbg2
TAATATAAGAATCTTTCCTGTGCTCGTCT
>SRR4252620_3741075___Sbg2
TGTGCTCGCCCATGGTCTGGTATTTAGGT
>SRR4252608_4623005___Sbg2
TAATATAAGAATCTTTCCTGTGCTCGCCA
>SRR4252612_5756118___Sbg2
TAATATAAGAATCTTTCCTGTGCTCGCCA
>SRR4252612_5880384___Sbg2
TAATATAAGAATCTTTCCTGTGCTCGCCA
>SRR4252613_2506167___Sbg2
TAATATAAGAATCTTTCCTGTGCTCGCCA
>SRR4252611_5730192___Sbg2
TAAGAATCTTTCCTGTGCTCGCCCATTTT
>SRR4252610_280263___Sbg2
TAAGAATCTTTCCTGTGCTCGCCCATGGTC
>SRR4252613_4546015___Sbg2
AAGGACCCTCCTGGCTCTCCATATCCA
>SRR4252611_323605___Sbg2
TCCCAACCTCAGAAATGGGTTACAATACA
>SRR4252609_13431491___Sbg2
GGAGGGAATACCAGTATGTCGACAAGACA
>SRR4252611_9688612___Sbg2
GGACCCTCCTGGCACTCCATATCCATTATCA
>SRR4252610_8630618___Sbg2
CCACGCCAGGAGGGAATACCAGTATG
>SRR4252607_11820576___Sbg2
CCGCCTCCCAGGTGTCCCAACCTCAGAA

>SRR4252610_2060743___Sbg2
TACAGCCACGCCAGGAGGGAATACCAGTA
>SRR4252610_2125153___Sbg2
ACAGCCACGCCAGGAGGGAATACCAGTA
>SRR4252612_4390410___Sbg2
CCGCCTCCCAGGTGTCCCAACCTCAGAA
>SRR4252608_12455435___Sbg2
AATGGGTTACAATACAGCCACGCCAGGA
>SRR4252610_11514715___Sbg2
ACAGCCACGCCAGGAGGGAATACCAGTA
>SRR4252623_12425263___Sbg2
TAATATAAGAATCTTTCCTGTGCTCGCCC
>SRR4252609_6821791___Sbg2
CCCGCCTCCCAGGTGTCCCAACCTCAGAA
>SRR4252611_13817538___Sbg2
GGAGGGAATACCAGTATGTCGACAAGAC
>SRR4252607_11594905___Sbg2
ACCCTCCTGGCACTCCATATCCATTATCA
>SRR4252611_7841987___Sbg2
ATACAGCCACGCCAGGAGGGAATACCAGTA
>SRR4252610_10789904___Sbg2
ACAGCCACGCCAGGAGGGAATACCAGTA
>SRR4252618_284772___Sbg2
AAGTTAGGTTTAAGGACCCTCCTGGCACT
>SRR4252607_13741035___Sbg2
GGTTACAATACAGCCACGCCAGGAGGGAA
>SRR4252609_6385385___Sbg2
ACAGCCACGCCAGGAGGGAATACCAGTA
>SRR4252611_11806433___Sbg2
ATGTCGACAAGACAACATGGTGGCATTGAAT
>SRR4252606_7820112___Sbg2
ACCCGCCTCCCAGGTGTCCCAACCTCAGA
>SRR4252619_851735___Sbg2
AGCCACGCCAGGAGGGAATACCAGTA
>SRR4252606_6955797___Sbg2
TGGTTACAATACAGCCACGCCAGGAGGGA
>SRR4252610_5426693___Sbg2
TGGTTACAATACAGCCACGCCAGGAGGGA
>SRR4252610_9793283___Sbg2
TGGTTACAATACAGCCACGCCAGGAGGGA
>SRR4252609_5327331___Sbg2
ACAGCCACGCCAGGAGGGAATACCAGTA
>SRR4252621_9873812___Sbg2
ACAGCCACGCCAGGAGGGAATACCAGTA
>SRR4252607_3045894___Sbg2
GGTTACAATACAGCCACGCCAGGAGGGAA
>SRR4252608_12221199___Sbg2
ACCCTCCTGGCACTCCATATCCATTATCA
>SRR4252626_3840009___Sbg2
TCCCAACCTCAGAAATGGGTTACAATACA
>SRR4252610_8296215___Sbg2
ACAGCCACGCCAGGAGGGAATACCAGTA
>SRR4252610_2518881___Sbg2
GGTTACAATACAGCCACGCCAGGAGGGAA
>SRR4252623_14048088___Sbg2
TAAGTTAGGTTTAAGGACCCTCCTGGCAC
>SRR4252608_934518___Sbg2
ACCCTCCTGGCACTCCATATCCATTA
>SRR4252607_1683782___Sbg2
CCACGCCAGGAGGGAATACCAGTATGTCGA
>SRR4252625_10617219___Sbg2
TACAGCCACGCCAGGAGGGAATACCAGTA
>SRR4252605_7263532___Sbg2
ACCCGCCTCCCAGGTGTCCCAACCTCAGA
>SRR4252624_8069671___Sbg2
TAATATAAGAATCTTTCCTGTGCTCGCC
>SRR4252621_973686___Sbg2
GGTTACAATACAGCCACGCCAGGAGGGAA
>SRR4252611_2888195___Sbg2
ACAGCCACGCCAGGAGGGAATACCAGTA

>SRR4252612_6176444___Sbg2
ACAGCCACGCCAGGAGGGAATACCAGTA
>SRR4252612_4579270___Sbg2
TTAAGTTAGGTTTAAGGACCCTCCTGGCACA
>SRR4252606_10038344___Sbg2
CCAGGAGGGAATACCAGTATGTCGACA
>SRR4252616_12194952___Sbg2
TTAGGTTTAAGGACCCTCCTGGCACT
>SRR4252608_6011829___Sbg2
CCGCCTCCCAGGTGTCCCAACCTCAGAA
>SRR4252610_12837251___Sbg2
ACAGCCACGCCAGGAGGGAATACCAGTA
>SRR4252607_7800202___Sbg2
TACAGCCACGCCAGGAGGGAATACCAGTA
>SRR4252612_3493845___Sbg2
ACAGCCACGCCAGGAGGGAATACCAGTA
>SRR4252605_13888458___Sbg2
ACAGCCACGCCAGGAGGGAATACCAGTA
>SRR4252614_3100016___Sbg2
AAGTTAGGTTTAAGGACCCTCCTGGCAC
>SRR4252608_7374120___Sbg2
CCCGCCTCCCAGGTGTCCCAACCTCAGAA
>SRR4252606_6602819___Sbg2
CCGCCTCCCAGGTGTCCCAACCTCAGAA
>SRR4252605_572317___Sbg2
ACAGCCACGCCAGGAGGGAATACCAGTA
>SRR4252606_8254974___Sbg2
ACCCGCCTCCCAGGTGTCCCAACCTCAGA
>SRR4252624_7459962___Sbg2
TCCTGGCACTCCATATCCATTATCACCTTA
>SRR4252611_1635840___Sbg2
TTTCCCATCGGGACAATGTAACCCGCCTCCC
>SRR4252606_1073881___Sbg2
TTACAATACAGCCACGCCAGGAGGGAA
>SRR4252609_10332619___Sbg2
ACAGCCACGCCAGGAGGGAATACCAGTA
>SRR4252609_5466188___Sbg2
TAGGTTTAAGGACCCTCCTGGCACTC
>SRR4252608_10438949___Sbg2
GGTTACAATACAGCCACGCCAGGAGGGAA
>SRR4252611_2579800___Sbg2
GGAGGGAATACCAGTATGTCGACAAGACA
>SRR4252617_11872619___Sbg2
GACAAGACAACATGGTGGCATTGAAT
>SRR4252624_2408850___Sbg2
ACCCTCCTGGCACTCCATATCCATTATCA
>SRR4252608_10235730___Sbg2
CCCGCCTCCCAGGTGTCCCAACCTCAGA
>SRR4252606_8402114___Sbg2
GGGTTACAATACAGCCACGCCAGGAGGGA
>SRR4252621_2438370___Sbg2
AGTTAGGTTTAAGGACCCTCCTGGCAC
>SRR4252611_4496053___Sbg2
TAGGTTTAAGGACCCTCCTGGCACTCCAT
>SRR4252605_6291899___Sbg2
ACCCTCCTGGCACTCCATATCCATTATCA
>SRR4252612_2488659___Sbg2
ACAGCCACGCCAGGAGGGAATACCAGTA
>SRR4252617_3590404___Sbg2
TCTTTAATATAAGAATCTTTCCTGTGCTCGC
>SRR4252610_12348289___Sbg2
ACCCGCCTCCCAGGTGTCCCAACCTCAGA
>SRR4252626_6021919___Sbg2
ACAGCCACGCCAGGAGGGAATACCAGTA
>SRR4252623_8136365___Sbg2
TTAATATAAGAATCTTTCCTGTGCTCG
>SRR4252605_12834187___Sbg2
ACCCGCCTCCCAGGTGTCCCAACCTCAGA
>SRR4252623_5117374___Sbg2
CTTTAATATAAGAATCTTTCCTGTGCTC

>SRR4252606_2727120___Sbg2
ACCCTCCTGGCACTCCATATCCATTATCA
>SRR4252608_11068238___Sbg2
TAAGTTAGGTTTAAGGACCCTCCTGGC
>SRR4252608_10098491___Sbg2
CCGCCTCCCAGGTGTCCCAACCTCAGA
>SRR4252610_7084702___Sbg2
ACAGCCACGCCAGGAGGGAATACCAGTATG
>SRR4252610_5831115___Sbg2
CCCGCCTCCCAGGTGTCCCAACCTCAGAA
>SRR4252606_1519002___Sbg2
GCCTCCCAGGTGTCCCAACCTCAGAA
>SRR4252607_2510053___Sbg2
TACAGCCACGCCAGGAGGGAATACCAGTA
>SRR4252610_2046057___Sbg2
CCGCCTCCCAGGTGTCCCAACCTCAGAA
>SRR4252611_10697758___Sbg2
ACAGCCACGCCAGGAGGGAATACCAGTA
>SRR4252611_12051063___Sbg2
ACAGCCACGCCAGGAGGGAATACCAGTA
>SRR4252608_2333263___Sbg2
GGAGGGAATACCAGTATGTCGACAAGACA
>SRR4252606_6859094___Sbg2
TAAGAATCTTTCCTGTGCTCGCCCATGGTC
>SRR4252612_3555347___Sbg2
TCGCCTCCCAGGTGTCCCAACCTCAGAA
>SRR4252611_11622227___Sbg2
GGTTACAATACAGCCACGCCAGGAGGGAA
>SRR4252605_13629590___Sbg2
CAGCCACGCCAGGAGGGAATACCAGTA
>SRR4252623_6869699___Sbg2
TTTCCTGTGCTCGCCCATGGTCTGGC
>SRR4252622_6319710___Sbg2
TCCGCCTCCCAGGTGTCCCAACCTCAGAA
>SRR4252609_336183___Sbg2
TCCCAACCTCAGAAATGGGTTACAATACA
>SRR4252611_13476518___Sbg2
TACAGCCACGCCAGGAGGGAATACCAGTA
>SRR4252610_2615366___Sbg2
GGTTACAATACAGCCACGCCAGGAGGGAA
>SRR4252607_11864556___Sbg2
CCACGCCAGGAGGGAATACCAGTATGTCGA
>SRR4252610_249846___Sbg2
GGTTACAATACAGCCACGCCAGGAGGGA
>SRR4252608_6865907___Sbg2
TTCCTGTGCTCGCCCATGGTCTGGTATT
>SRR4252609_15508837___Sbg2
AATTTTCTTTAATATAAGAATCTTTCCTG
>SRR4252611_12687632___Sbg2
TAAGAATCTTTCCTGTGCTCGCCCATGGT
>SRR4252619_15302581___Sbg2
ACAGCCACGCCAGGAGGGAATACCAGTA
>SRR4252615_6569977___Sbg2
CCAACCTCAGAAATGGGTTACAATACA
>SRR4252610_11647460___Sbg2
GGAGGGAATACCAGTATGTCGACAAGACA
>SRR4252605_14508129___Sbg2
TTAATATAAGAATCTTTCCTGTGCTCGC
>SRR4252608_2209403___Sbg2
ACAGCCACGCCAGGAGGGAATACCAGTA
>SRR4252610_668268___Sbg2
AGCCACGCCAGGAGGGAATACCAGTA
>SRR4252623_5736300___Sbg2
CTCCTGGCACTCCATATCCATTATCA
>SRR4252610_3802401___Sbg2
ACAGCCACGCCAGGAGGGAATACCAGTATG
>SRR4252612_1696577___Sbg2
ACAGCCACGCCAGGAGGGAATACCAGTA
>SRR4252612_7536896___Sbg2
AACCTCAGAAATGGGTTACAATACAGCCA

>SRR4252623_14029812___Sbg2
TTAAGTTAGGTTTAAGGACCCTCCTGGCACT
>SRR4252618_5000657___Sbg2
TATAAGAATCTTTCCTGTGCTCGCCCAT
>SRR4252610_5936424___Sbg2
ACAGCCACGCCAGGAGGGAATACCAGTA
>SRR4252610_9559544___Sbg2
ACAGCCACGCCAGGAGGGAATACCAGTA
>SRR4252605_6448585___Sbg2
ACAGCCACGCCAGGAGGGAATACCAGTA
>SRR4252608_11280385___Sbg2
AGCCACGCCAGGAGGGAATACCAGTA
>SRR4252610_7344146___Sbg2
CCGCCTCCCAGGTGTCCCAACCTCAGAA
>SRR4252611_13824538___Sbg2
ACCCTCCTGGCACTCCATATCCATTATCA
>SRR4252620_6041007___Sbg2
GGTTACAATACAGCCACGCCAGGAGGGAA
>SRR4252606_4046059___Sbg2
GGTTACAATACAGCCACGCCAGGAGGGAA
>SRR4252607_1387954___Sbg2
ACAGCCACGCCAGGAGGGAATACCAGTA
>SRR4252610_9768273___Sbg2
AGTTAGGTTTAAGTTAGGTTTAAGGA
>SRR4252611_10887241___Sbg2
GCATTTAGGTTAAGTTAGGTTTAAGTTA
>SRR4252607_8320555___Sbg2
ACAGCCACGCCAGGAGGGAATACCAGTA
>SRR4252616_6716803___Sbg2
GGAGGGAATACCAGTATGTCGACAAGACA
>SRR4252609_16664724___Sbg2
ACCCTCCTGGCACTCCATATCCATTATCA
>SRR4252621_5487345___Sbg2
GGTTACAATACAGCCACGCCAGGAGGGAA
>SRR4252606_2760029___Sbg2
TAATATAAGAATCTTTCCTGTGCTCGCCCA
>SRR4252612_7862769___Sbg2
ACAGCCACGCCAGGAGGGAATACCAGT
>SRR4252607_3711442___Sbg2
CCAGGAGGGAATACCAGTATGTCGACA
>SRR4252608_1856035___Sbg2
CCGCCTCCCAGGTGTCCCAACCTCAGAA
>SRR4252610_10134251___Sbg2
CCGCCTCCCAGGTGTCCCAACCTCAGAA
>SRR4252606_3921209___Sbg2
ACAGCCACGCCAGGAGGGAATACCAGTA
>SRR4252621_6429529___Sbg2
ACAGCCACGCCAGGAGGGAATACCAGTA
>SRR4252619_11005596___Sbg2
TAATATAAGAATCTTTCCTGTGCTCGCCC
>SRR4252616_6237854___Sbg2
TAAGAATCTTTCCTGTGCTCGCCCAT
>SRR4252622_763752___Sbg2
TCCCAACCTCAGAAATGGGTTACAATACA
>SRR4252607_2520193___Sbg2
CCGCCTCCCAGGTGTCCCAACCTCAGAA
>SRR4252606_2986812___Sbg2
ACAGCCACGCCAGGAGGGAATACCAGTA
>SRR4252608_11046330___Sbg2
GGTTACAATACAGCCACGCCAGGAGGGAA
>SRR4252611_9090383___Sbg2
TGTCCCAACCTCAGAAATGGGTTACAATA
>SRR4252607_9036843___Sbg2
TAAGAATCTTTCCTGTGCTCGCCCATG
>SRR4252611_11361676___Sbg2
GGTTACAATACAGCCACGCCAGGAGGGAA
>SRR4252608_4835878___Sbg2
GTTACAATACAGCCACGCCAGGAGGGAA
>SRR4252609_402240___Sbg2
ACAGCCACGCCAGGAGGGAATACCAGTA

```
>SRR4252610_12159421___Sbg2
CCCGCCTCCCAGGTGTCCCAACCTCAGAA
>SRR4252610_9373937___Sbg2
GCCTCCCAGGTGTCCCAACCTCAGAA
>SRR4252612_5700969___Sbg2
ATACAGCCACGCCAGGAGGGAATACCAGTA
>SRR4252610_11827208___Sbg2
ACAGCCACGCCAGGAGGGAATACCAGTA
>SRR4252608_2808503___Sbg2
ACAGCCACGCCAGGAGGGAATACCAGTA
>SRR4252605_15149780___Sbg2
AGCCACGCCAGGAGGGAATACCAGTA
>SRR4252609_9477915___Sbg2
TGCCTCCCAGGTGTCCCAACCTCAGAA
>SRR4252608_2131119___Sbg2
AGCCACGCCAGGAGGGAATACCAGTA
>SRR4252611_11875004___Sbg2
TAAGAATCTTTCCTGTGCTCGCCCATGG
>SRR4252624_6812931___Sbg2
TACAGCCACGCCAGGAGGGAATACCAGTA
>SRR4252608_4366523___Sbg2
TAATATAAGAATCTTTCCTGTGCTCGCCC
>SRR4252605_14642198___Sbg2
ACCCTCCTGGCACTCCATATCCATTATCA
>SRR4252609_16458987___Sbg2
ACAGCCACGCCAGGAGGGAATACCAGTA
>SRR4252605_13241471___Sbg2
AGAGAGCCAGGAGGGTCCTTAAACCTA
>SRR4252610_9150724___Sbg2
ATGGACGAGCACAGGAAAGATTCTTA
>SRR4252614_3596612___Sbg2
AAATACCAGACCATGGGCGAGCACAGGAAA
>SRR4252613_9981253___Sbg2
ATGGATATGGAGAGCCAGGAGGGTCCTT
>SRR4252621_1373693___Sbg2
GGAGAGCCAGGAGGGTCCTTAAACCTAAC
>SRR4252611_69223___Sbg2
CCAGGAGGGTCCTTAAACCTAACTTA
>SRR4252615_2533668___Sbg2
TAAATACCAGACCATGGGCGAGCACAG
>SRR4252610_1075171___Sbg2
TACTGGTATTCCCTCCTGGCGTGGCTGTA
>SRR4252611_2532788___Sbg2
TACTGGTATTCCCTCCTGGCGTGGCTGTA
>SRR4252623_13231398___Sbg2
TACTGGTATTCCCTCCTGGCGTGGCTGTA
>SRR4252607_9107078___Sbg2
TAACCCATTTCTGAGGTTGGGACACCTGG
>SRR4252610_11549540___Sbg2
TACTGGTATTCCCTCCTGGCGTGGCTGT
>SRR4252620_1133157___Sbg2
GCCAGACCATGGGCGAGCACAGGAAA
>SRR4252610_9189495___Sbg2
GGTGATAATGGATATGGAGTGCCAGGAGG
>SRR4252611_14805354___Sbg2
TGTCTTGTCGACATACTGGTATTCCCTCC
>SRR4252613_10480329___Sbg2
TACTGGTATTCCCTCCTGGCGTGGCTGTA
>SRR4252610_483797___Sbg2
TTCTGAGGTTGGGACACCTGGGAGGC
>SRR4252613_1334761___Sbg2
GCGAGCACAGGAAAGATTCTTATATT
>SRR4252605_7174226___Sbg2
CGAGCACAGGAAAGATTCTTATATTAAAG
>SRR4252605_2168373___Sbg2
ATGGATATGGAGAGCCAGGAGGGTCCTT
>SRR4252612_7283834___Sbg2
TACTGGTATTCCCTCCTGGCGTGGCTGT
>SRR4252621_8100361___Sbg2
TCCTGGCGTGGCTGTATTGTAACCCA
```

>SRR4252607_2401691___Sbg2
TTCTGAGGTTGGGACACCTGGGAGGCGG
>SRR4252608_3251351___Sbg2
GACCATGGGCGAGCACAGGAAAGATTCTTA
>SRR4252621_7318778___Sbg2
AGTGCCAGGAGGGTCCTTAAACCTAACTTAA
>SRR4252606_231656___Sbg2
TGTCTTGTCGACATACTGGTATTCCCTCC
>SRR4252608_2851720___Sbg2
TACTGGTATTCCCTCCTGGCGTGGCTGT
>SRR4252611_13297103___Sbg2
TACTGGTATTCCCTCCTGGCGTGGCTGT
>SRR4252612_4394761___Sbg2
TACTGGTATTCCCTCCTGGCGTGGCTGT
>SRR4252611_2358843___Sbg2
TGTATTGTAACCCATTTCTGAGGTTGGGAC
>SRR4252606_1200539___Sbg2
TCTGAGGTTGGGACACCTGGGAGGCGGGT
>SRR4252618_4895157___Sbg2
GGCGAGCACAGGAAAGATTCTTATATTAAA
>SRR4252620_8388736___Sbg2
ACCAGGAGGGTCCTTAAACCTAACTTAA
>SRR4252611_127805___Sbg2
CCAGGAGGGTCCTTAAACCTAACTTA
>SRR4252609_6948018___Sbg2
TCTGAGGTTGGGACACCTGGGAGGCGG
>SRR4252608_8048479___Sbg2
TGATAATGGATATGGAGTGCCAGGAGG
>SRR4252608_2185707___Sbg2
TTCTGAGGTTGGGACACCTGGGAGGCGGG
>SRR4252609_16726019___Sbg2
TCTGAGGTTGGGACACCTGGGAGGCGGGT
>SRR4252611_14552471___Sbg2
TACTGGTATTCCCTCCTGGCGTGGCTGT
>SRR4252608_849906___Sbg2
TTCCCTCCTGGCGTGGCTGTATTGTAAC
>SRR4252619_13240868___Sbg2
CTGGCGTGGCTGTATTGTAACCCATTTC
>SRR4252610_13597127___Sbg2
TTCTGAGGTTGGGACACCTGGGAGGCGG
>SRR4252608_7592684___Sbg2
TACTGGTATTCCCTCCTGGCGTGGCTGT
>SRR4252610_5423798___Sbg2
TTCCCTCCTGGCGTGGCTGTATTGTAAC
>SRR4252605_10973166___Sbg2
TGTCTTGTCGACATACTGGTATTCCCTCA
>SRR4252611_8229513___Sbg2
TCTGAGGTTGGGACACCTGGGAGGCGGG
>SRR4252612_5445276___Sbg2
TACTGGTATTCCCTCCTGGCGTGGCTGT
>SRR4252606_10380772___Sbg2
TCTGAGGTTGGGACACCTGGGAGGCGGGT
>SRR4252611_5166324___Sbg2
TACTGGTATTCCCTCCTGGCGTGGCTGT
>SRR4252608_4925403___Sbg2
TCTGAGGTTGGGACACCTGGGAGGCGGGT
>SRR4252608_3131493___Sbg2
TACTGGTATTCCCTCCTGGCGTGGCTGT
>SRR4252610_5885740___Sbg2
GACCATGGGCGAGCACAGGAAAGATTCTTA
>SRR4252624_706564___Sbg2
GTGCCAGGAGGGTCCTTAAACCTAACTTA
>SRR4252610_5486659___Sbg2
AAATACCAGACCATGGGCGAGCACAGGAA
>SRR4252612_4866684___Sbg2
TGTCTTGTCGACATACTGGTATTCCCTCC
>SRR4252607_3827074___Sbg2
TACTGGTATTCCCTCCTGGCGTGGCTGTA
>SRR4252606_4020631___Sbg2
GGGCGAGCACAGGAAAGATTCTTATATTA

>SRR4252608_5788620___Sbg2
TTCTGAGGTTGGGACACCTGGGAGGCGG
>SRR4252613_1688933___Sbg2
TACTGGTATTCCCTCCTGGCGTGGCTGT
>SRR4252606_8281693___Sbg2
TGATAATGGATATGGAGTGCCAGGAG
>SRR4252608_7254289___Sbg2
GGCGTGGCTGTATTGTAACCCATTTC
>SRR4252611_12353462___Sbg2
TACTGGTATTCCCTCCTGGCGTGGCTGT
>SRR4252608_8152742___Sbg2
TCGACATACTGGTATTCCCTCCTGGCGTGA
>SRR4252611_2094247___Sbg2
GGCGAGCACAGGAAAGATTCTTATATTAA
>SRR4252612_3140195___Sbg2
TACTGGTATTCCCTCCTGGCGTGGCTGT
>SRR4252606_8900661___Sbg2
TCGACATACTGGTATTCCCTCCTGGCGTGG
>SRR4252626_7702218___Sbg2
TTCCCTCCTGGCGTGGCTGTATTGTAACC
>SRR4252610_3352107___Sbg2
CGTGGCTGTATTGTAACCCATTTCTGAGGTT
>SRR4252609_11846775___Sbg2
TGTATTGTAACCCATTTCTGAGGTTGGGA
>SRR4252623_7405430___Sbg2
TGATAATGGATATGGAGTGCCAGGAGGGT
>SRR4252605_3179250___Sbg2
GACCATGGGCGAGCACAGGAAAGATTCTTA
>SRR4252605_358541___Sbg2
TACTGGTATTCCCTCCTGGCGTGGCTGT
>SRR4252610_6522497___Sbg2
TACTGGTATTCCCTCCTGGCGTGGCTGT
>SRR4252611_7882922___Sbg2
TCTGAGGTTGGGACACCTGGGAGGCGGGT
>SRR4252605_4702402___Sbg2
TGATAATGGATATGGAGTGCCAGGAGGG
>SRR4252623_520016___Sbg2
CCATGGGCGAGCACAGGAAAGATTCTTA
>SRR4252611_12034984___Sbg2
TTGTCTTGTCGACATACTGGTATTCCCTCT
>SRR4252611_3087860___Sbg2
TGTCTTGTCGACATACTGGTATTCCCTCT
>SRR4252623_8981556___Sbg2
TGTCTTGTCGACATACTGGTATTCCCTCT
>SRR4252619_1580422___Sbg2
TTCTGAGGTTGGGACACCTGGGAGGCGG
>SRR4252612_7672180___Sbg2
TACTGGTATTCCCTCCTGGCGTGGCTGT
>SRR4252611_1749059___Sbg2
ATGGATATGGAGAGCCAGGAGGGTCCTTAAA
>SRR4252612_3482965___Sbg2
ATACCAGACCATGGGCGAGCACAGGAAAGA
>SRR4252608_6284077___Sbg2
TTCTGAGGTTGGGACACCTGGGAGGCGG
>SRR4252611_3303850___Sbg2
TGGAGTGCCAGGAGGGTCCTTAAACCT
>SRR4252609_10460325___Sbg2
TACTGGTATTCCCTCCTGGCGTGGCTGT
>SRR4252610_13455167___Sbg2
TGTCGACATACTGGTATTCCCTCCTGG
>SRR4252612_3841809___Sbg2
TCGACATACTGGTATTCCCTCCTGGCGTGG
>SRR4252610_2676078___Sbg2
TTCCCTCCTGGCGTGGCTGTATCGTAACC
>SRR4252619_2112142___Sbg2
TGATAATGGATATGGAGTGCCAGGAGGGT
>SRR4252609_6651305___Sbg2
CCATGGGCGAGCACAGGAAAGATTCTTA
>SRR4252611_11342716___Sbg2
CCATGGGCGAGCACAGGAAAGATTCTTA

>SRR4252611_7996381___Sbg2
TACTGGTATTCCCTCCTGGCGTGGCTGA
>SRR4252622_9049626___Sbg2
TACTGGTATTCCCTCCTGGCGTGGCTGA
>SRR4252611_15523508___Sbg2
TAAGGTGATAATGGATATGGAGTGCCAG
>SRR4252612_3270714___Sbg2
ATAATGGATATGGAGTGCCAGGAGGG
>SRR4252612_6888407___Sbg2
TGTCTTGTCGACATACTGGTATTCCCTCC
>SRR4252608_6041720___Sbg2
TGATAATGGATATGGAGTGCCAGGAGGGT
>SRR4252606_3718877___Sbg2
TGATAATGGATATGGAGTGCCAGGAGGGT
>SRR4252611_14738930___Sbg2
TACTGGTATTCCCTCCTGGCGTGGCTGT
>SRR4252619_12162530___Sbg2
TATTCCCTCCTGGCGTGGCTGTATTGTA
>SRR4252608_7913773___Sbg2
TCTGAGGTTGGGACACCTGGAAGGCG
>SRR4252608_7744868___Sbg2
TACTGGTATTCCCTCCTGGCGTGGCTGT
>SRR4252610_6996449___Sbg2
TCCCTCCTGGCGTGGCTGTATTGTAACCC
>SRR4252605_3263504___Sbg2
TGATAATGGATATGGAGTGCCAGGAGGTT
>SRR4252606_5502823___Sbg2
TGCGAGCACAGGAAAGATTCTTATATTA
>SRR4252610_5253316___Sbg2
TTCCCTCCTGGCGTGGCTGTATTGTAAC
>SRR4252611_11989276___Sbg2
TGTCTTGTCGACATACTGGTATTCCCTCC
>SRR4252611_13774440___Sbg2
TACTGGTATTCCCTCCTGGCGTGGCTGTA
>SRR4252606_2678707___Sbg2
TACTGGTATTCCCTCCTGGCGTGGCTGT
>SRR4252622_2544616___Sbg2
TACTGGTATTCCCTCCTGGCGTGGCTGT
>SRR4252608_7306490___Sbg2
TGATAATGGATATGGAGTGCCAGGAGGGT
>SRR4252625_11388279___Sbg2
TGATAATGGATATGGAGTGCCAGGAGGGT
>SRR4252609_11010657___Sbg2
TACTGGTATTCCCTCCTGGCGTGGCTGT
>SRR4252608_102901___Sbg2
TACTGGTATTCCCTCCTGGCGTGGCTGT
>SRR4252605_8420609___Sbg2
TACTGGTATTCCCTCCTGGCGTGGCT
>SRR4252610_1066548___Sbg2
TTCCCTCCTGGCGTGGCTGTATTGTAAC
>SRR4252607_9820323___Sbg2
TTCTGAGGTTGGGACACCTGGGAGGCGG
>SRR4252619_6507851___Sbg2
TTCTGAGGTTGGGACACCTGGGAGGCGG
>SRR4252608_10967691___Sbg2
TGATAATGGATATGGAGTGCCAGGAGGG
>SRR4252621_13487896___Sbg2
TGCCAGGAGGGTCCTTAAACCTAACTTAA
>SRR4252608_3144923___Sbg2
TACTGGTATTCCCTCCTGGCGTGGCTGT
>SRR4252605_11071886___Sbg2
TACTGGTATTCCCTCCTGGCGTGGCTGT
>SRR4252613_5604676___Sbg2
TACTGGTATTCCCTCCTGGCGTGGCTGTA
>SRR4252606_1912931___Sbg2
TACTGGTATTCCCTCCTGGCGTGGCTGTA
>SRR4252606_4266230___Sbg2
TACTGGTATTCCCTCCTGGCGTGGCTGT
>SRR4252613_7308997___Sbg2
TACTGGTATTCCCTCCTGGCGTGGCTGT

>SRR4252605_5329488___Sbg2
TTCTGAGGTTGGGACACCTGGGAGGCGGG
>SRR4252625_14099911___Sbg2
TACTGGTATTCCCTCCTGGCGTGGCTGT
>SRR4252606_4086766___Sbg2
TTCCCTCCTGGCGTGGCTGTATTGTAACC
>SRR4252610_6171038___Sbg2
TACTGGTATTCCCTCCTGGCGTGGCTGT
>SRR4252605_9683984___Sbg2
TGATAATGGATATGGAGTGCCAGGAGGGT
>SRR4252610_2916746___Sbg2
TTCTGAGGTTGGGACACCTGGGAGGCGG
>SRR4252625_11707296___Sbg2
GCCAGGAGGGTCCTTAAACCTAACTT
>SRR4252611_12961984___Sbg2
TACTGGTATTCCCTCCTGGCGTGGCTGTA
>SRR4252616_14274614___Sbg2
TCCCTCCTGGCGTGGCTGTATTGTAACCC
>SRR4252624_5694568___Sbg2
TCCCTCCTGGCGTGGCTGTATTGTAACCC
>SRR4252606_1852105___Sbg2
TTCTGAGGTTGGGACACCTGGGAGGCGG
>SRR4252608_12080684___Sbg2
TTCTGAGGTTGGGACACCTGGGAGGCGG
>SRR4252612_7196043___Sbg2
TCTGAGGTTGGGACACCTGGGAGGCGGGT
>SRR4252609_5956002___Sbg2
TTCTGAGGTTGGGACACCTGGGAGGCGG
>SRR4252610_6680382___Sbg2
TGTCGACATACTGGTATTCCCTCCTGGC
>SRR4252611_2987721___Sbg2
TAAATGCCAGACCATGGGCGAGCACAGGA
>SRR4252606_3379567___Sbg2
TACTGGTATTCCCTCCTGGCGTGGCTGT
>SRR4252607_8221062___Sbg2
TACTGGTATTCCCTCCTGGCGTGGCTGT
>SRR4252610_10306135___Sbg2
TACTGGTATTCCCTCCTGGCGTGGCTGT
>SRR4252619_8128444___Sbg2
TACTGGTATTCCCTCCTGGCGTGGCTGT
>SRR4252610_3722835___Sbg2
TAACCCATTTCTGAGGTTGGGACACCTG
>SRR4252611_7205051___Sbg2
TACTGGTATTCCCTCCTGGCGTGGCTGTA
>SRR4252607_3760972___Sbg2
TTGTAACCCATTTCTGAGGTTGGGACACA
>SRR4252620_10646390___Sbg2
GCCAGACCATGGGCGAGCACAGGAAA
>SRR4252609_15248047___Sbg2
TACTGGTATTCCCTCCTGGCGTGGCTG
>SRR4252610_6853622___Sbg2
CGTGGCTGTATTGTAACCCATTTCTGAGGTT
>SRR4252610_11466757___Sbg2
GCGAGCACAGGAAAGATTCTTATATTAAA
>SRR4252611_6577845___Sbg2
TACTGGTATTCCCTCCTGGCGTGGCTGT
>SRR4252621_11443185___Sbg2
TACTGGTATTCCCTCCTGGCGTGGCTGT
>SRR4252608_4389381___Sbg2
TTCTGAGGTTGGGACACCTGGGAGGCGA
>SRR4252611_6251119___Sbg2
TTCTGAGGTTGGGACACCTGGGAGGCGA
>SRR4252610_7743955___Sbg2
TGATAATGGATATGGAGTGCCAGGAGGGT
>SRR4252606_1556573___Sbg2
TTCTGAGGTTGGGACACCTGGGAGGC
>SRR4252611_8242341___Sbg2
TAAACCTAACTTAAACCTAACTTAACCTA
>SRR4252606_841444___Sbg2
TCCCTCCTGGCGTGGCTGTATTGTAACCT

>SRR4252610_10921662___Sbg2
TCCCTCCTGGCGTGGCTGTATTGTAACCT
>SRR4252610_6785402___Sbg2
TCCCTCCTGGCGTGGCTGTATTGTAACCT
>SRR4252614_1744050___Sbg2
TCCCTCCTGGCGTGGCTGTATTGTAACCT
>SRR4252616_15765879___Sbg2
TCCCTCCTGGCGTGGCTGTATTGTAACCT
>SRR4252624_1313101___Sbg2
TCCCTCCTGGCGTGGCTGTATTGTAACCT
>SRR4252610_3573297___Sbg2
CCCTCCTGGCGTGGCTGTATTGTAACCT
>SRR4252621_10842280___Sbg2
TACTGGTATTCCCTCCTGGCGTGGCTGT
>SRR4252611_883738___Sbg2
CCTTAAACCTAACTTAAACCTAACTTAAC
>SRR4252612_6333013___Sbg2
TACTGGTATTCCCTCCTGGCGTGGCTGT
>SRR4252607_3269528___Sbg2
CTCCTGGCGTGGCTGTATTGTAACCCAT
>SRR4252608_833710___Sbg2
TGATAATGGATATGGAGTGCCAGGAGGGT
>SRR4252616_4397874___Sbg2
AGGTCCTTAAACCTAACTTAAACCTAACTT
>SRR4252614_7973455___Sbg2
TCTTGTCGACATACTGGTATTCCCTCC
>SRR4252605_2279336___Sbg2
ATGCCAGGAGGGTCCTTAAACCTAACTTA
>SRR4252607_1336847___Sbg2
TACTGGTATTCCCTCCTGGCGTGGCT
>SRR4252611_5855432___Sbg2
TGTCTTGTCGACATACTGGTATTCCCTCC
>SRR4252622_8438623___Sbg2
TCTGAGGTTGGGACACCTGGGAGGCGG
>SRR4252608_7300877___Sbg2
GGCGTGGCTGTATTGTAACCCATTTC
>SRR4252612_8856068___Sbg2
TCTGAGGTTGGGACACCTGGGAGGCGG
>SRR4252625_2401520___Sbg2
TGTCGACATACTGGTATTCCCTCCTGG
>SRR4252611_8049033___Sbg2
TACTGGTATTCCCTCCTGGCGTGGCTGT
>SRR4252612_7819428___Sbg2
TACTGGTATTCCCTCCTGGCGTGGCTGT
>SRR4252606_5469701___Sbg2
TTCCCTCCTGGCGTGGCTGTATTGTAACC
>SRR4252610_3891395___Sbg2
TTCCCTCCTGGCGTGGCTGTATTGTAACC
>SRR4252610_3549924___Sbg2
TCCTGGCGTGGCTGTATTGTAACCCATT
>SRR4252620_5208845___Sbg2
TGAGGTTGGGACACCTGGGAGGCGGGTT
>SRR4252617_13436343___Sbg2
GGGCGAGCACAGGAAAGATTCTTATA
>SRR4252605_13491587___Sbg2
CGAGCACAGGAAAGATTCTTATATTAAAG
>SRR4252605_2226118___Sbg2
TGTCTTGTCGACATACTGGTATTCCCTCT
>SRR4252610_10039207___Sbg2
TGTCTTGTCGACATACTGGTATTCCCTCT
>SRR4252625_1016386___Sbg2
TAACCCATTTCTGAGGTTGGGACACCTGG
>SRR4252611_8150650___Sbg2
TACTGGTATTCCCTCCTGGCGTGGCTGT
>SRR4252605_13934606___Sbg2
TCCCTCCTGGCGTGGCTGTATTGTAACC
>SRR4252617_8309941___Sbg2
AGTGCCAGGAGGGTCCTTAAACCTAA
>SRR4252609_2686688___Sbg8
AACCACACGTCACCCCTTCACTGGTTGGA

>SRR4252611_10889066___Sbg8
ACATGTGTGACCCTGAGGCCAGCAGCCGGC
>SRR4252621_7444294___Sbg8
GTTGGATGACGGTTCACCTCCATGTCTTA
>SRR4252610_1077794___Sbg8
ACCACACGTCACCCCTTCACTGGTTGGA
>SRR4252610_9356214___Sbg8
TAGGACATGTGTGACCCTGAGGCCAGCAG
>SRR4252606_6947272___Sbg8
TAGGACATGTGTGACCCTGAGGCCAGCAG
>SRR4252605_14931820___Sbg8
ACCACACGTCACCCCTTCACTGGTTGGA
>SRR4252610_2234935___Sbg8
ACCACACGTCACCCCTTCACTGGTTGGA
>SRR4252606_8328702___Sbg8
TAGGACATGTGTGACCCTGAGGCCAGCAGC
>SRR4252607_4040950___Sbg8
ACCACACGTCACCCCTTCACTGGTTGGA
>SRR4252618_6586806___Sbg8
TAGGACATGTGTGACCCTGAGGCCAGCAG
>SRR4252610_1707841___Sbg8
TAGGACATGTGTGACCCTGAGGCCAGCA
>SRR4252607_5429915___Sbg8
TAGGACATGTGTGACCCTGAGGCCAGCAG
>SRR4252625_7148285___Sbg8
TAGGACATGTGTGACCCTGAGGCCAGCAGC
>SRR4252612_1103143___Sbg8
GTTCACCTCCATGTCTTAGGACATGT
>SRR4252608_6952807___Sbg8
TAGGACATGTGTGACCCTGAGGCCAGCAG
>SRR4252607_13755130___Sbg8
GTTGGATGACGGTTCACCTCCATGTCTTA
>SRR4252605_2295318___Sbg8
TAGGACATGTGTGACCCTGAGGCCAGCAG
>SRR4252605_7004409___Sbg8
TAGGACATGTGTGACCCTGAGGCCAGCAG
>SRR4252610_6559427___Sbg8
TAGGACATGTGTGACCCTGAGGCCAGCAG
>SRR4252617_8957767___Sbg8
GACATGTGTGACCCTGAGGCCAGCAGCC
>SRR4252608_10728344___Sbg8
ACCACACGTCACCCCTTCACTGGTTGGA
>SRR4252610_7800003___Sbg8
TAGGACATGTGTGACCCTGAGGCCAGCAG
>SRR4252610_5540701___Sbg8
ATGGATGACGGTTCACCTCCATGTCTTA
>SRR4252610_3038958___Sbg8
CCATGTCTTAGGACATGTGTGACCCTG
>SRR4252610_4611299___Sbg8
TAGGACATGTGTGACCCTGAGGCCAGCA
>SRR4252619_9573183___Sbg8
TAGGACATGTGTGACCCTGAGGCCAGCAG
>SRR4252606_3830835___Sbg8
ACCACACGTCACCCCTTCACTGGTTGGA
>SRR4252605_8037483___Sbg8
GGTTGGATGACGGTTCACCTCCATGTCTTA
>SRR4252609_10955703___Sbg8
AACCACACGTCACCCCTTCACTGGTTGGA
>SRR4252605_11484624___Sbg8
CCACACGTCACCCCTTCACTGGTTGGATG
>SRR4252610_1153181___Sbg8
AACCACACGTCACCCCTTCACTGGTTGGA
>SRR4252610_4884284___Sbg8
TTGGATGACGGTTCACCTCCATGTCTTA
>SRR4252612_8285206___Sbg8
TGGATGACGGTTCACCTCCATGTCTTA
>SRR4252606_10153560___Sbg8
TAGGACATGTGTGACCCTGAGGACAGCAG
>SRR4252610_3179048___Sbg8
TAGGACATGTGTGACCCTGAGGACAGCAG

>SRR4252610_67940___Sbg8
TAGGACATGTGTGACCCTGAGGACAGCAG
>SRR4252608_8724144___Sbg8
ACATGTGTGACCCTGAGGCCAGCAGCCGG
>SRR4252611_5650183___Sbg8
ACCACACGTCACCCCTTCACTGGTTGG
>SRR4252607_13807241___Sbg8
GTTGGATGACGGTTCACCTCCATGTCTTA
>SRR4252610_11838792___Sbg8
GTTGGATGACGGTTCACCTCCATGTCTTA
>SRR4252610_10069446___Sbg8
TAGGACATGTGTGACCCTGAGGCCAGCAG
>SRR4252610_6147386___Sbg8
AACCACACGTCACCCCTTCACTGGTTGGA
>SRR4252607_9634326___Sbg8
ACCACACGTCACCCCTTCACTGGTTGGA
>SRR4252610_10306643___Sbg8
AACCACACGTCACCCCTTCACTGGTTGGA
>SRR4252610_6740169___Sbg8
TAGGACATGTGTGACCCTGAGGCCAGCAG
>SRR4252611_10474751___Sbg8
ACCACACGTCACCCCTTCACTGGTTGGA
>SRR4252605_345631___Sbg8
CCACACGTCACCCCTTCACTGGTTGGA
>SRR4252608_5588489___Sbg8
TAGGACATGTGTGACCCTGAGGCCAGCAG
>SRR4252613_10647321___Sbg8
TAGGACATGTGTGACCCTGAGGCCAGCA
>SRR4252605_2839792___Sbg8
ACATGTGTGACCCTGAGGCCAGCAGCCGGG
>SRR4252606_8116205___Sbg8
AACCACACGTCACCCCTTCACTGGTTGGA
>SRR4252610_6861881___Sbg8
AACCACACGTCACCCCTTCACTGGTTGGA
>SRR4252612_461890___Sbg8
CTACACGTCACCCCTTCACTGGTTGGA
>SRR4252610_4569595___Sbg8
TACACGTCACCCCTTCACTGGTTGGA
>SRR4252610_9962525___Sbg8
TAGGACATGTGTGACCCTGAGGCCAGC
>SRR4252607_14926953___Sbg8
AACCACACGTCACCCCTTCACTGGTTGGA
>SRR4252608_9194580___Sbg8
TAGGACATGTGTGACCCTGAGGCCAGCAG
>SRR4252606_5117462___Sbg8
ATCACACGTCACCCCTTCACTGGTTGGA
>SRR4252608_382417___Sbg8
TCACACGTCACCCCTTCACTGGTTGGA
>SRR4252608_10701874___Sbg8
GACCACACGTCACCCCTTCACTGGTTGGA
>SRR4252607_13063778___Sbg8
ACCACACGTCACCCCTTCACTGGTTGGA
>SRR4252612_6200031___Sbg8
AACCACACGTCACCCCTTCACTGGTTGGA
>SRR4252605_14184749___Sbg8
AACCACACGTCACCCCTTCACTGGTTGGA
>SRR4252607_2923228___Sbg8
CCACACGTCACCCCTTCACTGGTTGGA
>SRR4252623_15064698___Sbg8
CCACACGTCACCCCTTCACTGGTTGGAT
>SRR4252605_6564239___Sbg8
ACCACACGTCACCCCTTCACTGGTTGGA
>SRR4252605_3589281___Sbg8
ACATGTGTGACCCTGAGGGCAGCAGCCGG
>SRR4252607_4666979___Sbg8
TAGGACATGTGTGACCCTGAGGCCAGCAG
>SRR4252605_14720129___Sbg8
CCACACGTCACCCCTTCACTGGTTGGATG
>SRR4252610_2116144___Sbg8
AACCACACGTCACCCCTTCACTGGTTGGA

```
>SRR4252605_7239328___Sbg8
TAGGACATGTGTGACCCTGAGGCCAGCAG
>SRR4252607_1884298___Sbg8
AACCACACGTCACCCCTTCACTGGTTGGA
>SRR4252608_3964409___Sbg8
GGTTGGATGACGGTTCACCTCCATGTCTT
>SRR4252610_13517573___Sbg8
ACCACACGTCACCCCTTCACTGGTTGGA
>SRR4252606_4451411___Sbg8
GTTGGATGACGGTTCACCTCCATGTCTTA
>SRR4252609_1732524___Sbg8
GGATGACGGTTCACCTCCATGTCTTA
>SRR4252620_8732360___Sbg8
TAGGACATGTGTGACCCTGAGGCCAGCAG
>SRR4252610_1853921___Sbg8
AACCACACGTCACCCCTTCACTGGTTGGA
>SRR4252605_1227577___Sbg8
TAGGACATGTGTGACCCTGAGGCCAGC
>SRR4252608_4521184___Sbg8
TAGGACATGTGTGACCCTGAGGCCAGCAA
>SRR4252607_6326763___Sbg8
AACCACACGTCACCCCTTCACTGGTTGGA
>SRR4252608_4772822___Sbg8
TAACCACACGTCACCCCTTCACTGGTT
>SRR4252608_8690780___Sbg8
TAGGACATGTGTGACCCTGAGGCCAGC
>SRR4252610_3795131___Sbg8
TAGGACATGTGTGACCCTGAGGCCAGCAC
>SRR4252612_3058591___Sbg8
TAGGACATGTGTGACCCTGAGGCCAGCAC
>SRR4252607_872087___Sbg8
ACCACACGTCACCCCTTCACTGGTTGGAT
>SRR4252612_6238519___Sbg8
GGTTGGATGACGGTTCACTTCCATGTCTTA
>SRR4252609_2376812___Sbg8
GTTGGATGACGGTTCACTTCCATGTCTTA
>SRR4252610_8447365___Sbg8
AACCACACGTCACCCCTTCACTGGTTGGA
>SRR4252608_7616038___Sbg8
ACCACACGTCACCCCTTCACTGGTTGGA
>SRR4252621_2363294___Sbg8
GTTGGATGACGGTTCACCTCCATGTCTTA
>SRR4252606_611127___Sbg8
TAGGACATGTGTGACCCTGAGGCCAGCAG
>SRR4252621_10724547___Sbg8
TAGGATGACGGTTCACCTCCATGTCTTA
>SRR4252605_1416052___Sbg8
TAGGACATGTGTGACCCTGAGGCCAGCAG
>SRR4252610_10317811___Sbg8
TAGGACATGTGTGACCCTGAGGCCAGCAG
>SRR4252609_10313890___Sbg8
TGGATGACGGTTCACCTCCATGTCTTA
>SRR4252615_4175981___Sbg8
TAGGACATGTGTGAGCCTGAGGCCAG
>SRR4252605_10268847___Sbg8
ACCACACGTCACCCCTTCACTGGTTGGA
>SRR4252616_4987065___Sbg8
CTAACCACACGTCACCCCTTCACTGGT
>SRR4252612_7127581___Sbg8
ACCACACGTCACCCCTTCACTGGTTGGA
>SRR4252611_11230200___Sbg8
ACATGTGTGACCCTGAGGCCAGCAGCCGGT
>SRR4252607_14059965___Sbg8
CCACACGTCACCCCTTCACTGGTTGGA
>SRR4252621_2554226___Sbg8
TAGGACATGTGTGACCCTGAGGCCAGC
>SRR4252607_5102121___Sbg8
CTAACCACACGTCACCCCTTCACTGGTTG
>SRR4252608_7836999___Sbg8
ACCACACGTCACCCCTTCACTGGTTGGA
```

```
>SRR4252609_7337181___Sbg8
ACCACACGTCACCCCTTCACTGGTTGGA
>SRR4252610_100286___Sbg8
TTGGATGACGGTTCACCTCCATGTCTTA
>SRR4252607_1515___Sbg8
TAGGACATGTGTGACCCTGAGGCCAGCAG
>SRR4252607_1741243___Sbg8
TGGATGACGGTTCACCTCCATGTCTTA
>SRR4252607_1479578___Sbg8
ATTGGATGACGGTTCACCTCCATGTCTTA
>SRR4252606_3472211___Sbg8
GACCACACGTCACCCCTTCACTGGTTGGA
>SRR4252608_632205___Sbg8
GACCACACGTCACCCCTTCACTGGTTGGA
>SRR4252610_2110521___Sbg8
GACCACACGTCACCCCTTCACTGGTTGGA
>SRR4252605_7684144___Sbg8
TAACCACACGTCACCCCTTCACTGGTT
>SRR4252608_7374236___Sbg8
CCACACGTCACCCCTTCACTGGTTGGA
>SRR4252607_11111443___Sbg8
ACCACACGTCACCCCTTCACTGGTTGGA
>SRR4252605_9544078___Sbg8
GACCACACGTCACCCCTTCACTGGTTGGA
>SRR4252609_8928515___Sbg8
ACATGTGTGACCCTGAGGCCAGCAGCCGG
>SRR4252607_3869137___Sbg8
ATGGATGACGGTTCACCTCCATGTCTTA
>SRR4252624_3337345___Sbg8
CCACACGTCACCCCTTCACTGGTTGGA
>SRR4252611_13921743___Sbg8
TAACCACACGTCACCCCTTCACTGGT
>SRR4252605_11043908___Sbg8
TAGGACATGTGTGACCCTGAGGCCAGCAA
>SRR4252608_4956621___Sbg8
TAGGACATGTGTGACCCTGAGGCCAGCAA
>SRR4252624_4101774___Sbg8
TAGGACATGTGTGACCCTGAGGCCAGC
>SRR4252608_388239___Sbg8
TAACCACACGTCACCCCTTCACTGGTTGGA
>SRR4252610_12474224___Sbg8
CCGGCTAGTAGGCCTAGGTCCTTCATG
>SRR4252610_1105458___Sbg8
TAGGACATGTGTGACCCTGAGGCCAGCAG
>SRR4252610_5160591___Sbg8
TAGGACATGTGTGACCCTGAGGCCAGCAG
>SRR4252608_9962046___Sbg8
CCATGTCTTAGGACATGTGTGACCCTG
>SRR4252612_6047507___Sbg8
TAGGACATGTGTGACCCTGAGGTCAG
>SRR4252612_8115513___Sbg8
CTAACCACACGTCACCCCTTCACTGGTTGG
>SRR4252605_11073770___Sbg8
TAGGACATGTGTGACCCTGAGGTCAGC
>SRR4252605_13067489___Sbg8
TAGGACATGTGTGACCCTGAGGTCAGC
>SRR4252611_11033515___Sbg8
TAGGACATGTGTGACCCTGAGGTCAGC
>SRR4252611_6935461___Sbg8
TAGGACATGTGTGACCCTGAGGTCAGCAG
>SRR4252612_6189175___Sbg8
TAGGACATGTGTGACCCTGAGGTCAGCAG
>SRR4252612_7374023___Sbg8
TAGGACATGTGTGACCCTGAGGTCAGC
>SRR4252612_763072___Sbg8
TAGGACATGTGTGACCCTGAGGTCAGCA
>SRR4252613_9308499___Sbg8
TAGGACATGTGTGACCCTGAGGTCAGCAG
>SRR4252608_11221675___Sbg8
TAGGACATGTGTGACCCTGAGGCCAGCAG
```

>SRR4252623_13431525___Sbg8
ACATGTGTGACCCTGAGGCCAGCAGCCGGC
>SRR4252624_8047217___Sbg8
GACCACACGTCACCCCTTCACTGGTTGGA
>SRR4252611_13916647___Sbg8
TGTGACCCTGAGGTCAGCAGCCGGCTAG
>SRR4252610_1055525___Sbg8
AACCACACGTCACCCCTTCACTGGTTGGA
>SRR4252607_13860838___Sbg8
TAGGACATGTGTGACCCTGAGGCCAGC
>SRR4252611_11524826___Sbg8
TAGGACATGTGTGACCCTGAGGCCAGC
>SRR4252606_441117___Sbg8
TAGGACATGTGTGACCCTGAGGCCAG
>SRR4252605_9103323___Sbg8
AACCACACGTCACCCCTTCACTGGTTGGA
>SRR4252610_13134289___Sbg8
GTGACCCTGAGGCCAGCAGCCGGCTTGTA
>SRR4252608_7034353___Sbg8
TAGGACATGTGTGACCCTGAGGCCAGA
>SRR4252612_6124528___Sbg8
TAGGACATGTGTGACCCTGAGGCCAGA
>SRR4252611_13648668___Sbg8
CCACACGTCACCCCTTCACTGGTTGGATG
>SRR4252624_1886210___Sbg8
TAGGACATGTGTGACCCTGAGGCCAGCAGT
>SRR4252623_11674388___Sbg8
TCACACGTCACCCCTTCACTGGTTGGA
>SRR4252608_2438053___Sbg8
CCATGTCTTAGGACATGTGTGACCCTG
>SRR4252611_1061811___Sbg8
ACATGTGTGACCCTGAGGCCAGCAGCC
>SRR4252612_8448034___Sbg8
TAGGACATGTGTGACCCTGAGGCCAGT
>SRR4252605_6059225___Sbg8
ACCACACGTCACCCCTTCACTGGTTGGA
>SRR4252606_2267536___Sbg8
ACATGTGTGACCCTGAGGCCAGCAGCA
>SRR4252607_4891544___Sbg8
TAGGACATGTGTGACCCTGAGGCCAGCAG
>SRR4252606_3439129___Sbg8
GTTGGATGACGGTTCACCTCCATGTCTTA
>SRR4252610_13473867___Sbg8
AACCACACGTCACCCCTTCACTGGTTGGA
>SRR4252607_7669779___Sbg8
CCACACGTCACCCCTTCACTGGTTGGA
>SRR4252608_5920703___Sbg8
TAGGACATGTGTGACCCTGAGGCCAGCAG
>SRR4252610_7974854___Sbg8
GTTGGATGACGGTTCACCTCCATGTCTTA
>SRR4252622_9436068___Sbg8
TAGGACATGTGTGACCCTGAGGCCAGCAG
>SRR4252621_9013133___Sbg8
TAGGACATGTGTGACCCTGAGGCCAGC
>SRR4252608_6204743___Sbg8
AGTTGGATGACGGTTCACCTCCATGTCTTA
>SRR4252621_167562___Sbg8
AGTTGGATGACGGTTCACCTCCATGTCTTA
>SRR4252622_530911___Sbg8
TGTTGGATGACGGTTCACCTCCATGTCTTA
>SRR4252622_5469625___Sbg8
GACCGGTACCCACCCAGCATCGTGAGGAA
>SRR4252606_8257582___Sbg8
CCATGTCTTAGGACATGTGTGACGCTG
>SRR4252608_6523653___Sbg8
ATGTCTTAGGACATGTGTGACGCTGAGG
>SRR4252611_13184444___Sbg8
TAGGACATGTGTGACCCTGAGGCCAGCAG
>SRR4252606_2203625___Sbg8
TCACACGTCACCCCTTCACTGGTTGGAT

>SRR4252612_6617583___Sbg8
TGGATGACGGTTCACCTCCATGTCTTA
>SRR4252610_10991032___Sbg8
ACCACACGTCACCCCTTCACTGGTTGGA
>SRR4252610_7340845___Sbg8
GACCGGTGCTCACCCAGCATCGTGAGGAA
>SRR4252611_12294897___Sbg8
AACCGGTGTCCACCCAGCATCGTGAGGAAA
>SRR4252606_109028___Sbg8
TAGGACATGTGTGACCCTGAGGCCATC
>SRR4252606_8093555___Sbg8
TAGGACATGTGTGACCCTGAGGCCAT
>SRR4252620_10070534___Sbg8
TAGGACATGTGTGACCCTGAGGCCATC
>SRR4252620_10112552___Sbg8
TAGGACATGTGTGACCCTGAGGCCATC
>SRR4252608_11688453___Sbg8
GACACGTCACCCCTTCACTGGTTGGA
>SRR4252608_17929___Sbg8
CTTCACTGGTTGGATGATGGTTCACCTCT
>SRR4252612_6058816___Sbg8
TGACGGTTCACTTCCATGTCTTAGGACAT
>SRR4252608_4797876___Sbg8
GGTTGGATGACAGTTCACCTCCATGTCTT
>SRR4252608_3834076___Sbg8
GTTGGATGACAGTTCACCTCCATGTCTTA
>SRR4252606_8510556___Sbg8
CTGCTGGCCTCAGGGTCACACATGTCCTA
>SRR4252612_5699823___Sbg8
CTGCTGGCCTCAGGGTCACACATGTCCTA
>SRR4252624_7150997___Sbg8
TCCAACCAGTGAAGGGGTGACGTGTGGTT
>SRR4252606_7638190___Sbg8
CTGCTGGCCTCAGGGTCACACATGTCCTA
>SRR4252605_11050367___Sbg8
CTGCTGGCCTCAGGGTCACACATGTCCTA
>SRR4252623_14394199___Sbg8
ATCCAACCAGTGAAGGGGTGACGTGTGG
>SRR4252607_11677014___Sbg8
CTGCTGGCCTCAGGGTCACACATGTCCTA
>SRR4252612_6947921___Sbg8
TAAGACATGGAGGTGAACCGTCATCCAAC
>SRR4252607_10867593___Sbg8
TAAGACATGGAGGTGAACCGTCATCCAAC
>SRR4252610_10740932___Sbg8
GCTGGCCTCAGGGTCACACATGTCCTA
>SRR4252608_1908644___Sbg8
TCCAACCAGTGAAGGGGTGACGTGTGGT
>SRR4252608_9535752___Sbg8
TTGCTGGCCTCAGGGTCACACATGTCCTA
>SRR4252610_8130737___Sbg8
GCTGGCCTCAGGGTCACACATGTCCTA
>SRR4252608_3741386___Sbg8
CTGGCCTCAGGGTCACACATGTCCTA
>SRR4252610_11259954___Sbg8
TCCAACCAGTGAAGGGGTGACGTGTGGTT
>SRR4252624_1595116___Sbg8
CTGCTGGCCTCAGGGTCACACATGTCCTA
>SRR4252610_13182359___Sbg8
TACAAGCCGGCTGCTGGCCTCAGGGTCAC
>SRR4252611_13632646___Sbg8
TCCAACCAGTGAAGGGGTGACGTGTGGT
>SRR4252605_6490409___Sbg8
TAAGACATGGAGGTGAACCGTCATCCAACC
>SRR4252610_10311448___Sbg8
TAAGACATGGAGGTGAACCGTCATCCAACC
>SRR4252623_233748___Sbg8
TCCAACCAGTGAAGGGGTGACGTGTG
>SRR4252623_6973789___Sbg8
TCCAACCAGTGAAGGGGTGACGTGTGGT

>SRR4252606_7930633___Sbg8
TAAGACATGGAGGTGAACCGTCATTC
>SRR4252608_5424853___Sbg8
TCCAACCAGTGAAGGGGTGACGTGTGGT
>SRR4252621_7120881___Sbg8
TCCAACCAGTGAAGGGGTGACGTGTGGTT
>SRR4252608_2110104___Sbg8
TCCAACCAGTGAAGGGGTGACGTGTGGTT
>SRR4252608_11130297___Sbg8
TCCAACCAGTGAAGGGGTGACGTGTGGT
>SRR4252610_9175192___Sbg8
TAAGACATGGAGGTGAACCGTCATCCA
>SRR4252605_11751239___Sbg8
TCCAACCAGTGAAGGGGTGACGTGTGGT
>SRR4252608_10545952___Sbg8
TCCAACCAGTGAAGGGGTGACGTGTGGT
>SRR4252608_8706363___Sbg8
TCCAACCAGTGAAGGGGTGACGTGTGGTT
>SRR4252608_12292111___Sbg8
TCCAACCAGTGAAGGGGTGACGTGTGG
>SRR4252606_1148877___Sbg8
TCCAACCAGTGAAGGGGTGACGTGTGGT
>SRR4252611_10982392___Sbg8
CCGGCTGCTGGCCTCAGGGTCACACATGT
>SRR4252608_965174___Sbg8
TCCAACCAGTGAAGGGGTGACGTGTGG
>SRR4252608_999684___Sbg8
TCCAACCAGTGAAGGGGTGACGTGTGGTT
>SRR4252605_3679913___Sbg8
TTCCTCACGATGCTGGGTGGGCACCGGTC
>SRR4252608_361666___Sbg8
TCCAACCAGTGAAGGGGTGACGTGTGGTT
>SRR4252606_770735___Sbg8
CTATCATAGCTTCCCACTTTCCTCACGA
>SRR4252610_1636804___Sbg8
TCCAACCAGTGAAGGGGTGACGTGTGGT
>SRR4252619_11444630___Sbg8
TCCAACCAGTGAAGGGGTGACGTGTGGTT
>SRR4252606_6659830___Sbg8
TCCAACCAGTGAAGGGGTGACGTGTGGTT
>SRR4252608_10964183___Sbg8
TCCAACCAGTGAAGGGGTGACGTGTGGT
>SRR4252624_6108771___Sbg8
TCCAACCAGTGAAGGGGTGACGTGTGG
>SRR4252609_11728816___Sbg8
TCCAACCAGTGAAGGGGTGACGTGTGG
>SRR4252610_2346153___Sbg8
TCCAACCAGTGAAGGGGTGACGTGTGGT
>SRR4252606_7682934___Sbg8
TCCAACCAGTGAAGGGGTGACGTGTGGT
>SRR4252610_3298304___Sbg8
TCCAACCAGTGAAGGGGTGACGTGTGGTT
>SRR4252608_4710325___Sbg8
TCCAACCAGTGAAGGGGTGACGTGTGGTT
>SRR4252614_4200825___Sbg8
CAGGGTCACACATGTCCTAAGACATGGA
>SRR4252620_10641515___Sbg8
AGCCGGCTGCTGGCCTCAGGGTCACACA
>SRR4252609_5382552___Sbg8
TCCAACCAGTGAAGGGGTGACGTGTGGTT
>SRR4252606_2176881___Sbg8
CTGCTGGCCTCAGGGTCACACATGTCCTA
>SRR4252606_4247563___Sbg8
CTGCTGGCCTCAGGGTCACACATGTCCTA
>SRR4252610_1851369___Sbg8
TGCTGGCCTCAGGGTCACACATGTCCTA
>SRR4252625_11686530___Sbg8
AGCCGGCTGCTGGCCTCAGGGTCACACATGT
>SRR4252610_11171088___Sbg8
TCCAACCAGTGAAGGGGTGACGTGTGGTT

>SRR4252619_4614007___Sbg8
TCCAACCAGTGAAGGGGTGACGTGTGGT
>SRR4252610_10713937___Sbg8
TCCGGCTGCTGGCCTCAGGGTCACACATGT
>SRR4252607_10317431___Sbg8
TCCAACCAGTGAAGGGGTGACGTGTGGA
>SRR4252610_10040149___Sbg8
TCCAACCAGTGAAGGGGTGACGTGTGGA
>SRR4252610_7365180___Sbg8
TCCAACCAGTGAAGGGGTGACGTGTGGA
>SRR4252611_12162549___Sbg8
TCCAACCAGTGAAGGGGTGACGTGTGGAT
>SRR4252614_6980283___Sbg8
TCCAACCAGTGAAGGGGTGACGTGTGGAT
>SRR4252623_10697274___Sbg8
TCCAACCAGTGAAGGGGTGACGTGTGGAT
>SRR4252607_10803495___Sbg8
CAACCAGTGAAGGGGTGACGTGTGGAT
>SRR4252606_24940___Sbg8
CTGCTGGCCTCAGGGTCACACATGTCCTA
>SRR4252611_14266027___Sbg8
AGGTGAACCATCATCCAACCAGTGAAG
>SRR4252610_10140254___Sbg8
TCATCCAACCAGTGAAGGGGTGACGTGTGG
>SRR4252608_851756___Sbg8
CTGCTGGCCTCAGGGTCACACATGTCCTA
>SRR4252607_1334903___Sbg8
TCCAACCAGTGAAGGGGTGACGTGTGGTC
>SRR4252612_3750654___Sbg8
TCCAACCAGTGAAGGGGTGACGTGTGGT
>SRR4252605_1483849___Sbg8
TCCAACCAGTGAAGGGGTGACGTGTGGT
>SRR4252608_3287973___Sbg8
CTGCTGGCCTCAGGGTCACACATGTCCTA
>SRR4252608_7617515___Sbg8
TCCAACCAGTGAAGGGGTGACGTGTGG
>SRR4252623_8767268___Sbg8
TCCAACCAGTGAAGGGGTGACGTGTG
>SRR4252611_13138326___Sbg8
GCCGGCTGCTGGCCTCAGGGTCACACATGT
>SRR4252610_2451798___Sbg8
TAAGACATGGAGGTGAACCGTCATCCAAC
>SRR4252621_3996553___Sbg8
TCCAACCAGTGAAGGGGTGACGTGTGGTT
>SRR4252619_3973861___Sbg8
TCCAACCAGTGAAGGGGTGACGTGTGGTT
>SRR4252611_4938156___Sbg8
CCGGCTGCTGGCCTCAGGGTCACACATGT
>SRR4252618_2259477___Sbg8
CCGGCTGCTGGCCTCAGGGTCACACATGT
>SRR4252608_9849797___Sbg8
TGCTGGCCTCAGGGTCACACATGTCCTA
>SRR4252605_12015703___Sbg8
TAAGACATGGAAGTGAACCGTCATCCAAC
>SRR4252610_2378206___Sbg8
TAAGACATGGAAGTGAACCGTCATCCAACC
>SRR4252610_742579___Sbg8
TAAGACATGGAAGTGAACCGTCATCCA
>SRR4252611_14740086___Sbg8
TAAGACATGGAAGTGAACCGTCATCCAACC
>SRR4252605_1043061___Sbg8
TCCAACCAGTGAAGGGGTGACGTGTGGT
>SRR4252624_6124805___Sbg8
TCCAACCAGTGAAGGGGTGACGTGTGGT
>SRR4252610_10684258___Sbg8
TCCAACCAGTGAAGGGGTGACGTGTGGT
>SRR4252610_7346961___Sbg8
TCCAACCAGTGAAGGGGTGACGTGTGGT
>SRR4252611_4637913___Sbg8
AGCCGGCTGCTGGCCTCAGGGTCACACATGT

>SRR4252607_606214___Sbg8
TCCAACCAGTGAAGGGGTGACGTGTGGT
>SRR4252606_4288458___Sbg8
CTGCTGGCCTCAGGGTCACACATGTCCTA
>SRR4252606_10021891___Sbg8
GCTGGCCTCAGGGTCACACATGTCCTA
>SRR4252605_13608090___Sbg8
AAGACATGGAGGTGAACCGTCATCCAACT
>SRR4252623_1386550___Sbg8
AAGACATGGAGGTGAACCGTCATCCAACT
>SRR4252611_27158___Sbg8
GCTGACCTCAGGGTCACACATGTCCTA
>SRR4252612_5273968___Sbg8
GCTGACCTCAGGGTCACACATGTCCTA
>SRR4252606_946061___Sbg8
TCCAACCAGTGAAGGGGTGACGTGTGTT
>SRR4252607_3070508___Sbg8
TATCCAACCAGTGAAGGGGTGACGTGTGG
>SRR4252608_1046083___Sbg8
TCCAACCAGTGAAGGGGTGACGTGTGGTT
>SRR4252610_11001394___Sbg8
GCTGGCCTCAGGGTCACACATGTCCTA
>SRR4252608_6201716___Sbg8
TCCAACCAGTGAAGGGGTGACGTGTGGTA
>SRR4252608_8050757___Sbg8
TCCAACCAGTGAAGGGGTGACGTGTGGTA
>SRR4252605_12530427___Sbg8
ATCCAACCAGTGAAGGGGTGACGTGTGG
>SRR4252612_1558021___Sbg8
TCCAACCAGTGAAGGGGTGACGTGTGGT
>SRR4252621_306896___Sbg8
TAAGACATGGAGGTGAACTGTCATCCAAC
>SRR4252608_1167834___Sbg8
TCCAACCAGTGAAGGGGTGACGTGTT
>SRR4252610_3471973___Sbg8
TCCAACCAGTGAAGGGGTGACGTGTGGTT
>SRR4252609_13549147___Sbg8
CCGGCTGCTGGCCTCAGGGTCACACATGT
>SRR4252612_8599699___Sbg8
AACCAGTGAAGGGGTGACGTGTGGTTA
>SRR4252607_6716648___Sbg8
ACCAGTGAAGGGGTGATGTGTGGTTAGC
>SRR4252608_9103437___Sbg8
TCCAACCAGTGAAGGGGTGACGTGTGGTT
>SRR4252622_182863___Sbg8
TCCAACCAGTGAAGGGGTGACGTGTGGTTT
>SRR4252607_10059301___Sbg8
TAAGACATGGAGGTGAACCGTCATCCAAC
>SRR4252610_690952___Sbg8
TCCAACCAGTGAAGGGGTGACGTGTGGTT
>SRR4252605_7327612___Sbg8
CCGGCTGCTGGCCTCAGGGTCACACATGT
>SRR4252608_6448128___Sbg8
CTGCTGGCCTCAGGGTCACACATGTCCTA
>SRR4252605_3331046___Sbg8
GCTGGCCTCAGGGTCACACATGTCCTA
>SRR4252610_2993839___Sbg8
TACCAGCCGGCTACTGGCCTCAGGGTCACA
>SRR4252608_2210281___Sbg8
TAAGACATGGAGGTGAACCGTCATCCAACA
>SRR4252612_6196352___Sbg8
TAAGACATGGAGGTGAACCGTCATCC
>SRR4252610_9162994___Sbg8
ATGAAGGGCCTAGGCCTACTAGCCGG
>SRR4252607_7504739___Sbg8
GCTGGCCTCAGGGTCACACATGTCCTA
>SRR4252609_2279162___Sbg8
GCTGGCCTCAGGGTCACACATGTCCTA
>SRR4252606_3216784___Sbg8
GCGGCTGCTGGCCTCAGGGTCACACATGT

>SRR4252612_5632272___Sbg8
CTGCTGGCCTCAGGGTCACACATGTCCTA
>SRR4252608_13061101___Sbg8
GCTGGCCTCAGGGTCACACATGTCCTA
>SRR4252619_10566020___Sbg8
GCTGGCCTCAGGGTCACACATGTCCTAA
>SRR4252605_14043939___Sbg8
TCCAACCAGTGAAGGGGTGACGTGTGGTT
>SRR4252611_10791665___Sbg8
TCCAACCAGTGAAGGGGTGACGTGTGGTTT
>SRR4252612_8730457___Sbg8
TAAGACATGGAGGTGAACTGTCATCCAAC
>SRR4252625_2508189___Sbg8
TAAGACATGGAGGTGAACTGTCATCCAA
>SRR4252612_5241549___Sbg8
ACCAGTGAAGGGGTGACGTGTGGTTT
>SRR4252611_6173669___Sbg8
AGCCGGCTGCTGGCCTCAGGGTCACAC
>SRR4252610_8802765___Sbg8
TCCAACCAGTGAAGGGGTGACGTGTGAT
>SRR4252606_5983825___Sbg8
TAAGACATGGAGGTGAACTGTCATCCAAC
>SRR4252606_9723675___Sbg8
TCCAACCAGTGAAGGGGTGACGTGTGGT
>SRR4252606_9828465___Sbg8
AGGTGAACTGTCATCCAACCAGTGAAG
>SRR4252611_5793861___Sbg8
AGGTGAACTGTCATCCAACCAGTGAAGG
>SRR4252605_13885879___Sbg8
ACTGGCCTCAGGGTCACACATGTCCTA
>SRR4252610_1166174___Sbg8
TACAAGCCGGCTGCTGGCCTCAGGGTCACAC
>SRR4252606_6325693___Sbg8
CTGCTGGCCTCAGGGTCACACATGTCCTA
>SRR4252611_5319649___Sbg8
AAGACATGGAGGTGAACCGTCATCCAAC
>SRR4252621_12668893___Sbg8
TAAGACATGGAGGTGAACCGTCATCCTA
>SRR4252609_10889225___Sbg8
TAAGACATGGAGGTGAACCGTCATCC
>SRR4252609_5426512___Sbg8
TCCAACCAGTGAAGGGGTGACGTGTAG
>SRR4252608_6492075___Sbg8
TCCAACCAGTGAAGGGGTGACGTGTGGT
>SRR4252605_11368529___Sbg8
ACTGGCCTCAGGGTCACACATGTCCTA
>SRR4252610_4427314___Sbg8
TAAGACATGGAAGTGAACCGTCATCCAAC
>SRR4252610_6497604___Sbg8
ACTGGCCTCAGGGTCACACATGTCCTA
>SRR4252606_6655951___Sbg8
CTGCTGGCCTCAGGGTCACACATGTCCTA
>SRR4252613_7468350___Sbg8
TAAGACATGGAGGTGAACTGTCATCCAAC
>SRR4252608_320816___Sbg8
TCCAACCAGTGAAGGGGTGACGTGTGTT
>SRR4252610_2872663___Sbg8
TCCAACCAGTGAAGGGGTGACGTGTGT
>SRR4252610_2956788___Sbg8
TCCAACCAGTGAAGGGGTGACGTGTGT
>SRR4252608_6136903___Sbg8
CTGCTGGCCTCAGGGTCACACATGTCCTA
>SRR4252623_5596280___Sbg8
TCCAACCAGTGAAGGGGTGACGTGTGG
>SRR4252608_10369400___Sbg8
TTGCTGGCCTCAGGGTCACACATGTCCTA
>SRR4252608_2881008___Sbg8
TTGCTGGCCTCAGGGTCACACATGTCCTA
>SRR4252610_927364___Sbg8
TTGCTGGCCTCAGGGTCACACATGTCCTA

>SRR4252605_14682250___Sbg8
CATCCAACCAGTGAAGGGGTGACGTGTGT
>SRR4252610_1077989___Sbg8
TCCAACCAGTGAAGGGGTGACGTGTGT
>SRR4252605_13866875___Sbg8
GCTGACCTCAGGGTCACACATGTCCTA
>SRR4252621_10836758___Sbg8
GCTGACCTCAGGGTCACACATGTCCTAAGA
>SRR4252611_13573069___Sbg8
CTGACCTCAGGGTCACACATGTCCTA
>SRR4252610_526648___Sbg8
TCCAACCAGTGAAGGGGTGACGTGTGGT
>SRR4252621_12616304___Sbg1
CCCTGGGGTGCACTCAGCCTCTAACAGAA
>SRR4252606_9674856___Sbg1
GGGGTGCACTCAGCCTCTAACAGAAATGA
>SRR4252611_12837069___Sbg1
GGTGCACTCAGCCTCTAACAGAAATGA
>SRR4252605_9449017___Sbg1
GGGTGCACTCAGCCTCTAACAGAAATGA
>SRR4252619_14331104___Sbg1
CCGTAACCTCCCGTCACCCTAAGGGCCT
>SRR4252611_11868597___Sbg1
CCTGGGGTGCACTCAGCCTCTAACAGAA
>SRR4252609_2315107___Sbg1
CCTGGGGTGCACTCAGCCTCTAACAGAA
>SRR4252605_8601637___Sbg1
TGGGGTGCACTCAGCCTCTAACAGAA
>SRR4252611_10164102___Sbg1
CCTGGGGTGCACTCAGCCTCTAACAGAA
>SRR4252611_2692473___Sbg1
TCTAACAGAAATGAGTACCAGGAGCTT
>SRR4252606_7618421___Sbg1
GCCCTGGGGTGCACTCAGCCTCTAACAGA
>SRR4252606_1393822___Sbg1
TTCCTGGGGGTGAAGGCGGCTGCCTCGTG
>SRR4252605_1738318___Sbg1
TCTAACAGAAATGAGTACCAGGAGCTTTT
>SRR4252622_168950___Sbg1
GAATTTTTCAATTGGTATAATCCTTCTGGT
>SRR4252612_1339366___Sbg1
TAACAGAAATGAGTACCAGGAGCTTT
>SRR4252623_7977998___Sbg1
CTGGGGTGCACTCAGCCTCTAACAGAA
>SRR4252606_7289487___Sbg1
GCCCTGGGGTGCACTCAGCCTCTAACAGA
>SRR4252608_5411269___Sbg1
TGGGGTGCACTCAGCCTCTAACAGAA
>SRR4252611_455523___Sbg1
TCAATTGGTATAATCCTTCTGGCCGCACT
>SRR4252622_2394313___Sbg1
CCCTGGGGTGCACTCAGCCTCTAACAGAA
>SRR4252605_12403889___Sbg1
CACAGGCCTTGTATGCCGATTGTCTGCAA
>SRR4252605_11594530___Sbg1
CCTGGGGTGCACTCAGCCTCTAACAGAA
>SRR4252619_4593298___Sbg1
CACAGGCCCTGTATGCCGATTGTCTGCAAA
>SRR4252622_7421378___Sbg1
CCTGGGGTGCACTCAGCCTCTAACAGAA
>SRR4252611_9419668___Sbg1
CCCTGGGGTGCACTCAGCCTCTAACAGAA
>SRR4252612_613253___Sbg1
CCCTGGGGTGCACTCAGCCTCTAACAGAA
>SRR4252624_4742476___Sbg1
CCCTGGGGTGCACTCAGCCTCTAACAGAA
>SRR4252609_7138222___Sbg1
GGGTGCACTCAGCCTCTAACAGAAATGA
>SRR4252611_4024382___Sbg1
TCTGGCCGCACTATGGCCCTGGGGTGCACT

>SRR4252610_10971___Sbg1
TGGGGTGCACTCAGCCTCTAACAGAA
>SRR4252625_7522342___Sbg1
GCCCTGGGGTGCACTCAGCCTCTAACAGA
>SRR4252619_14654722___Sbg1
TTCAATTGGTATAATCCTTCTGGCCGCA
>SRR4252609_11284743___Sbg1
GGGTGAACTCAGCCTCTAACAGAAATGA
>SRR4252621_8654040___Sbg1
TCAGCCTCTAACAGAAATGAGTACCAGGA
>SRR4252607_5124173___Sbg1
TCTAACAGAAATGAGTACCAGGAGCTT
>SRR4252612_581818___Sbg1
CCCTGGGGTGCACTCAGCCTCTAACAGAA
>SRR4252608_110466___Sbg1
CACAGGCCTTGTATGCCGATTGTCTGCAAA
>SRR4252613_9461329___Sbg1
CCTGGGGTGCACTCAGCCTCTAACAGAA
>SRR4252605_9075273___Sbg1
TGGGGTGCACTCAGCCTCTAACAGAA
>SRR4252607_14750992___Sbg1
ATTTTTCAATTGGTATAATGCTTCTG
>SRR4252611_12594981___Sbg1
TGACAACCTCACAGGCCTTGTATGCCGATT
>SRR4252605_12552211___Sbg1
CCCTGGGGTGCACTCAGCCTCTAACAGA
>SRR4252619_3468664___Sbg1
CAGGCCTTGTATGCCGATTGTCTGCAAA
>SRR4252611_55598___Sbg1
TCTAACAGAAATGAGTACCAGGAGCTT
>SRR4252623_2912105___Sbg1
CCTGGGGTGCACTCAGCCTCTAACAGAA
>SRR4252610_13154880___Sbg1
TCTAACAGAAATGAGTACCAGGAGCTTTT
>SRR4252620_3820404___Sbg1
TGGGTGCACTCAGCCTCTAACAGAAATGA
>SRR4252619_11922805___Sbg1
CAGGCCCTGTATGCCGATTGTCTGCAAA
>SRR4252608_9301195___Sbg1
GCCCTGGGGTGCACTCAGCCTCTAACAGA
>SRR4252611_6425655___Sbg1
TTTTCAATTGGTATAATCCTTCTGGCCGT
>SRR4252611_7897229___Sbg1
TCAATTGGTATAATCCTTCTGGCCGT
>SRR4252620_5645540___Sbg1
TGCCGTAACCTCCCGTCACCCTAAGGTC
>SRR4252610_290787___Sbg1
CCTGGAGTGCACTCAGCCTCTAACAGAAA
>SRR4252606_535578___Sbg1
TGGCCCTGGAGTGCACTCAGCCTCTAACA
>SRR4252611_13200026___Sbg1
TACAGGCCCTGTATGCCGATTGTCTGCAA
>SRR4252612_3522462___Sbg1
ACTCAGCCTCTAACAGAAATGAGTACCAG
>SRR4252611_1625919___Sbg1
CCTGGGGTGCACTCAGCCTCTAACAGAA
>SRR4252605_2198446___Sbg1
CCTGGGGTGCACTCAGCCTCTAACAGAA
>SRR4252605_13024752___Sbg1
AACCTCACAGGCCTTGTATGCCGATTG
>SRR4252606_633970___Sbg1
GGGGTGCACTCAGCCTCTAACAGAAATGA
>SRR4252610_10432919___Sbg1
CCGATTGTCTGCAAAGATGGGTGCCGTA
>SRR4252606_7121265___Sbg1
ACAGGCCCTGTATGCCGATTGTCTGCAA
>SRR4252620_5085995___Sbg1
TTTTTCAATTGGTATAATCCTTCTGGCCA
>SRR4252610_11157112___Sbg1
CCATGGGGTGCACTCAGCCTCTAACAGAA

```
>SRR4252605_12643653___Sbg1
ATGGGGTGCACTCAGCCTCTAACAGAA
>SRR4252619_11985075___Sbg1
AGGGTGCACTCAGCCTCTAACAGAAATGA
>SRR4252621_7745414___Sbg1
ACTCAGCCTCTAACAGAAATGAGTACCAG
>SRR4252619_11007539___Sbg1
CCCTGGGGTGCACTCAGCCTCTAACAGA
>SRR4252623_6399993___Sbg1
GGCCTTGTATGCCGATTGTCTACAAA
>SRR4252618_1973943___Sbg1
TCCTGGGGGTGAAGGCGGCTGCCTCGTG
>SRR4252606_490925___Sbg1
ACCCTGGGGTGCACTCAGCCTCTAACAGA
>SRR4252612_8022202___Sbg1
CCCTGGGGTGCACTCAGCCTCTAACAGAA
>SRR4252610_7918073___Sbg1
GCCCTGGGGTGCACTCAGCCTCTAACAGA
>SRR4252611_12253421___Sbg1
CAGCCTCTAACAGAAATGAGTACCAG
>SRR4252612_2052941___Sbg1
TCTAACAGAAATGAGTACCAGGAGCTTTC
>SRR4252605_320809___Sbg1
TCTGGCCGCACTATGGCCCTGGGGTGCACT
>SRR4252609_1458401___Sbg1
TCTAACAGAAATGAGTACCAGGAACTTTC
>SRR4252626_1875980___Sbg1
ATGCCGATTGTCTGCAAAGATGGGTGCCG
>SRR4252611_6713504___Sbg1
TCTAACAGAAATGAGTACCAGGAACTTT
>SRR4252609_11861428___Sbg1
CCTGGGGTGCACTCAGCCTCTAACAGAA
>SRR4252607_7487909___Sbg1
CCCTGGGGTGCACTCAGCCTCTAACAGA
>SRR4252623_9817841___Sbg1
CCCTGGGGTGCACTCAGCCTCTAACAGAA
>SRR4252626_5912403___Sbg1
TACAGGCCTTGTATGCCGATTGTCTGCAAA
>SRR4252621_13192473___Sbg1
TTTCAATTGGTATAATCCTTCTGGCCGCA
>SRR4252610_7313136___Sbg1
CACAGGCCTTGTATGCCGATTGTCTGCAAA
>SRR4252605_10358931___Sbg1
TATGCCGATTGTCTGCAAAGCTGGGTGCCG
>SRR4252611_12047329___Sbg1
ACCGATTGTCTGCAAAGATGGGTGCCGTA
>SRR4252614_8136088___Sbg1
CTATGGCCCTGGGGTGCACTCAGCCTCTA
>SRR4252610_5663365___Sbg1
CCCTGGGGTGTACTCAGCCTCTAACAGA
>SRR4252605_9953516___Sbg1
CCTGGGGTGTACTCAGCCTCTAACAGAA
>SRR4252611_4671221___Sbg1
TTTTTCAATTGGTATAATCCTTCTGGCCGA
>SRR4252621_9073451___Sbg1
TCTAACAGAAATGAGTACCAGAAGCTTT
>SRR4252611_2216059___Sbg1
ATAGGCCCTGTATGCCGATTGTCTGCAAA
>SRR4252608_12147290___Sbg1
ACAGGCCTTGTATGCCGATTGTCTGCAAA
>SRR4252611_11468545___Sbg1
CCCTGGGGTGCACTCAGCCTCTAACAGA
>SRR4252608_5004297___Sbg1
CCTAGGGTGCACTCAGCCTCTAACAGAA
>SRR4252612_7642434___Sbg1
CAGGCCTTGTATGCCGATTGTCTGCA
>SRR4252611_5661223___Sbg1
ACAGAAATGAGTACCAGGAGCTTTCCT
>SRR4252611_5389261___Sbg1
TCTAACAGAAATGAGTACCAGGAGCTTTC
```

>SRR4252612_167064___Sbg1
TCAATTGGTATAATCCTTCTGGCTGCACT
>SRR4252611_9943100___Sbg1
TTTTTCAATTGGTATAATCCTTCTGGCT
>SRR4252606_9659286___Sbg1
CCCGGGGTGCACTCAGCCTCTAACAGAA
>SRR4252609_15145497___Sbg1
CGGGGTGCACTCAGCCTCTAACAGAA
>SRR4252605_1873075___Sbg1
CCCTGGGGTTCACTCAGCCTCTAACAGA
>SRR4252605_10968167___Sbg1
CCGATTGTCTGCAAAGATGGGTGCCGTA
>SRR4252606_7805325___Sbg1
CCCAGGGGTGCACTCAGCCTCTAACAGAA
>SRR4252606_407429___Sbg1
AGGGGTGCACTCAGCCTCTAACAGAAAT
>SRR4252624_5434595___Sbg1
GCCCTGGGGTGCACTCAGCCTCTAACAGA
>SRR4252607_4210662___Sbg1
ACAGGCCTTGTATGCCGATTGTCTGAA
>SRR4252612_6390490___Sbg1
CAGGCCTTGTATGCCGATTGTCTGCAA
>SRR4252605_13976392___Sbg1
CCTGGGGTGCACTCAGCCTCTAACAGAA
>SRR4252614_274081___Sbg1
TCCTGGGGGTAAAGGCGGCTGCCTCGTGT
>SRR4252622_2370785___Sbg1
AGGCCTTGTATGCCGATTGTCTGCAAAGA
>SRR4252609_10802889___Sbg1
TGGGGCTGACAACCTCACAGGCCTTGTA
>SRR4252612_564897___Sbg1
CCAGGGGTGCACTCAGCCTCTAACAGAA
>SRR4252606_9870166___Sbg1
AGGGGTGCACTCAGCCTCTAACAGAAATGA
>SRR4252610_2568032___Sbg1
AGGGGTGCACTCAGCCTCTAACAGAAAT
>SRR4252608_11507702___Sbg1
CAGGCCTTGTATGCCGATTGTCTGCA
>SRR4252619_7226330___Sbg1
TCTAACAGAAATGAGTACCAGGAGCTTTC
>SRR4252614_3373067___Sbg1
TCTAACAGAAATGAGTACCAGGAGCTTT
>SRR4252610_7219785___Sbg1
TTTTTCAATTGGTATAATCCTTCTGGC
>SRR4252613_1098321___Sbg1
TAAAGGCGGCTGCCTCGTGGGGCTGA
>SRR4252621_12323421___Sbg1
CTGGGGTGCACTCAGCCTCTAACAGAA
>SRR4252610_13016276___Sbg1
CCCTGGGGTGCACTCAGCCTCTAACAGAA
>SRR4252624_10459711___Sbg1
CCCTGGGGTGCACTCAGCCTCTAACAGAA
>SRR4252605_10742461___Sbg1
TAATCCTTCTGGCCGCACTATGGCCCTGA
>SRR4252606_9623587___Sbg1
CCCGGGGTGCACTCAGCCTCTAACAGAA
>SRR4252614_3098635___Sbg1
ACTATGGCCCTGGGGTGCACTCAGCCTCTA
>SRR4252607_12014593___Sbg1
TTGTATGCCGATTGTCTGCAAAGATGTGT
>SRR4252605_10840298___Sbg1
CCTAGGGTGCACTCAGCCTCTAACAGAA
>SRR4252621_10885409___Sbg1
CCTAGGGTGCACTCAGCCTCTAACAGAA
>SRR4252624_3347592___Sbg1
CCTAGGGTGCACTCAGCCTCTAACAGAA
>SRR4252615_11591720___Sbg1
TCTAACAGAAATGAGTACCAGGAGCTTTT
>SRR4252612_1432710___Sbg1
CCTGGGGTGCACTCAGCCTCTAACAGAA

>SRR4252619_8921460___Sbg1
ACTATGGCCCTGGGGTGCACTCAGCCTCTA
>SRR4252611_10799385___Sbg1
CCCTGGGGTGCACTCAGCCTCTAACAGA
>SRR4252606_727854___Sbg1
TTCCTGGGGGTGAAGGCGGCTGCCTCGTG
>SRR4252612_8827241___Sbg1
GCCGATTGTCTGCAAAGATGGGTGCCGTA
>SRR4252619_10349290___Sbg1
CTGGGGTGCACTCAGCCTCTAACAGAAA
>SRR4252605_335065___Sbg1
CCCTGGGCTGCACTCAGCCTCTAACAGAA
>SRR4252621_10535984___Sbg1
CTGGGGTGCACTCAGCCTCTAACAGAAAT
>SRR4252605_12828057___Sbg1
TGACAACCTCACAGGCCTTGTATGCCGAT
>SRR4252611_4349316___Sbg1
CCTGGGGTGCACTCAGCCTCTAACAGAA
>SRR4252620_9554911___Sbg1
TTGGTATAATCCTTCTGGCCGCACTATGG
>SRR4252611_9630306___Sbg1
CACAGGCCTTGTATGCCGATTGTCTGCAA
>SRR4252625_12362134___Sbg1
CACAGGCCTTGTATGCCGATTGTCTGCAA
>SRR4252610_3562642___Sbg1
TCTGGCCGCACTATGGCCCTGGGGTGC
>SRR4252609_8109620___Sbg1
TCTAACAGAAATGAGTACCAGGAGCTATC
>SRR4252611_14499397___Sbg1
CACAGGCCTTGTATGCCGATTGTCTGCAAA
>SRR4252611_6244360___Sbg1
AGAAATGAGTACCAGGAGCTTTCCCG
>SRR4252620_8368280___Sbg1
ACCTCCCGTCACCCTAAGGGCCTTCCTATT
>SRR4252611_8650066___Sbg1
CAGCCTCTAACAGAAATGAGTACCAGGA
>SRR4252616_4310227___Sbg1
TGCCGATTGTCTGCAAAGATGGGTGCCGTA
>SRR4252606_2973542___Sbg1
CCCAGGGGTGCACTCAGCCTCTAACAGAA
>SRR4252607_4399042___Sbg1
CCCAGGGGTGCACTCAGCCTCTAACAGAA
>SRR4252611_11356907___Sbg1
CCAGGGGTGCACTCAGCCTCTAACAGAA
>SRR4252612_4418544___Sbg1
TATAATCCTTCTGGCCGCACTATGACCC
>SRR4252606_3361926___Sbg1
CACAGGCCCTGTATGCCGATTGTCTGCAA
>SRR4252614_2710640___Sbg1
ACAGGCCCTGTATGCCGATTGTCTGCAA
>SRR4252619_259069___Sbg1
GGGGTGCACTCAGCCTCTAACAGAAATGA
>SRR4252612_3692583___Sbg1
TTCCTGGGGGTGAAGGCGGCTGCCTCGTG
>SRR4252607_8603270___Sbg1
AGGCCTTGTATGCCGATTGTCTGCAAAGA
>SRR4252609_10066042___Sbg1
CCTGGGGTGCACTCAGCCTCTAACAGAA
>SRR4252609_11005889___Sbg1
CCTGGGGTGCACTCAGCCTCTAACAGAA
>SRR4252611_13746412___Sbg1
TATAATCCTTCTGGCCGCAGTATGGCCCTG
>SRR4252605_13957659___Sbg1
CCTGGGGTGCACTCAGCCTCTAACAGAA
>SRR4252610_3351856___Sbg1
TTGGGGTGCACTCAGCCTCTAACAGAAAT
>SRR4252606_596661___Sbg1
AATTGGTATAATCCTTCTGGCCGCACTA
>SRR4252621_10969748___Sbg1
GTCCTGGGGTGCACTCAGCCTCTAACAGA

>SRR4252605_255839___Sbg1
GCGGCTGCCTCGTGGGGCTGACAACCTCATA
>SRR4252606_7509551___Sbg1
CACAGGCCCTGTATGCCGATTGTCTGCAAA
>SRR4252621_5240319___Sbg1
CCCTGGGGTGCACTCAGCCTCTAACAGAA
>SRR4252606_3998292___Sbg1
CCTGGGGTGCACTCAGCCTCTAACAGAA
>SRR4252610_7688626___Sbg1
TCCTGGGGTGCACTCAGCCTCTAACAGAA
>SRR4252608_9290949___Sbg1
TCAGCTTCTAACAGAAATGAGTACCAGGA
>SRR4252610_4192946___Sbg1
AGGGTGCACTCAGCCTCTAACAGAAAT
>SRR4252609_7615028___Sbg1
CCGTAACCTCCCGTCACCCTAAGGGCTT
>SRR4252611_11976259___Sbg1
TCTAACAGAAATGAGTACAAGGAGCTTTC
>SRR4252611_11619812___Sbg1
CCTGGGGTGCACTCAGCCTCTAACAGAA
>SRR4252610_5065167___Sbg1
TATAATCCTTCTGGCCGCACTATGACCC
>SRR4252621_11511450___Sbg1
TAATCCTTCTGGCCGCACTATGACCCTG
>SRR4252607_2302830___Sbg1
TTCAATTGGTATAATCCTTCTGGCCGCG
>SRR4252612_4185937___Sbg1
TGAGGTGCACTCAGCCTCTAACAGAAATGA
>SRR4252606_5439727___Sbg1
CCCTGGGGTGCACTCAGCCTCTAACAGAA
>SRR4252625_13276168___Sbg1
GATTGTCTGCAAAGATGGGTGCCGTAA
>SRR4252609_1941024___Sbg1
GGGGTGCGCTCAGCCTCTAACAGAAATGA
>SRR4252619_2583387___Sbg1
TCTAACAGAAATGAGTACCAGGAGCTTT
>SRR4252612_768320___Sbg1
GCCCTGGGGTGCACTCAGCCTCTAACA
>SRR4252610_1832624___Sbg1
CACAGGCCTTGTATGCCGATTGTCTGCAAA
>SRR4252605_7796886___Sbg1
TGCCGATTGTCTGCAAAGATGGGTGCCGTA
>SRR4252610_3423675___Sbg1
TTTTTCAATTGGTATAATCTTTCTGGCC
>SRR4252611_30204___Sbg1
TGACAACCTCACAGGCCTTGAATGCCGATT
>SRR4252611_5578768___Sbg1
TGACAACCTCACAGGCCTTGAATGCCGATT
>SRR4252612_3186760___Sbg1
TGACAACCTCACAGGCCTTGAATGCCGATT
>SRR4252608_11366712___Sbg1
TACAGGCCTTGTATGCCGATTGTCTGCAA
>SRR4252612_2803339___Sbg1
TCTAACAGAAATGAGTACCAGGAGCTTTT
>SRR4252610_13003825___Sbg1
CACAGGCCTTGTATGCCGATTGTCTGCAA
>SRR4252609_15893364___Sbg1
GTGCACTCAGCCTCTAACAGAAATGA
>SRR4252611_12335068___Sbg1
CACAGGCCTTGTATGCCGATTGTCTGCAA
>SRR4252625_7466767___Sbg1
ACAGGCCTTGTATGCCGATTGTCTGA
>SRR4252605_2613870___Sbg1
CACAGGCCTTGTATGCCGATTGTCTGCAAA
>SRR4252611_8091200___Sbg1
CCTGGGATGCACTCAGCCTCTAACAGAA
>SRR4252610_12705822___Sbg1
GTGGAGCTGACAACCTCACAGGCCTTGTA
>SRR4252607_12760451___Sbg1
CCTGGGGTGCACTCAGCCTCTAACAGAA

>SRR4252610_1232794___Sbg1
CACAGGCCTTGTATGCCGATTGTCTGCAAA
>SRR4252609_2641174___Sbg1
CCTGGGGTGTACTCAGCCTCTAACAGAA
>SRR4252625_2183085___Sbg1
CTGGGGTGTACTCAGCCTCTAACAGA
>SRR4252621_9294848___Sbg1
CAGCCTCTAACAGAAATGAGTACCAGGA
>SRR4252606_10084109___Sbg1
ACCCTGGGGTGCACTCAGCCTCTAACAGA
>SRR4252606_8995522___Sbg1
ACCCTGGGGTGCACTCAGCCTCTAACAGA
>SRR4252606_7135232___Sbg1
CCTGGGGTGCACTCAGCCTCTAACAGAA
>SRR4252618_2732186___Sbg1
TTCCTGGGGGTAAAGGCGGCTGCCTCGTG
>SRR4252612_7022221___Sbg1
TAAAGGCGGCTGCCTCGTGGGGCTGACAAT
>SRR4252612_868838___Sbg1
TATGCCGATTGTCTGCAAATATGGGTGCC
>SRR4252610_9418741___Sbg1
CCTGGAGTGCACTCAGCCTCTAACAGAAA
>SRR4252612_1405878___Sbg1
CCTGGGTGCACTCAGCCTCTAACAGAA
>SRR4252619_5472577___Sbg1
CACAGGCCTTGTATGCCGATTGTCTGCAA
>SRR4252619_3605117___Sbg1
CTGGGGTGCACTCAGCCTCTAACAGAA
>SRR4252620_887306___Sbg1
TTCCTGGGGGTGAAGGCGTCTGCCTCGTG
>SRR4252611_24402___Sbg1
CCTGGGGTGCACTCAGCCTCTAACAGAA
>SRR4252613_1987794___Sbg1
CAGCTTCTAACAGAAATGAGTACCAG
>SRR4252609_2814107___Sbg1
TGGGGTGCACTCAGCCTCTAACAGAAATGA
>SRR4252611_7693020___Sbg1
CAGCCTCTAACAGAAATGAGTACCAGGA
>SRR4252619_3165368___Sbg1
TATAATCCTTCTGGCCGCACTATGACCC
>SRR4252612_9137792___Sbg1
TTCCTGGGGGTAAAGGCGGCTGCCTCGTG
>SRR4252612_3622896___Sbg1
TGCCGATTGTCTGCAAAGATGGGTGCCGTA
>SRR4252610_7890366___Sbg1
TCTAACAGAAATTAGTACCAGGAGCTTT
>SRR4252605_907618___Sbg1
GCCCTGGGGTGCACTCAGCCTCTAACAGA
>SRR4252606_7326208___Sbg1
GCCCTGGGGTGCACTCAGCCTCTAACAGA
>SRR4252610_11240155___Sbg1
CACAGGCCTTGTATGCCGATTGTCTGCAA
>SRR4252605_8688465___Sbg1
ATAGCCCTGGGGTGCACTCAGCCTCTAACAG
>SRR4252605_12491881___Sbg1
CCTGGGGTGCACTCAGCCTCTAACAGAA
>SRR4252605_2188887___Sbg1
TTGGCCCTGGGGTGCACTCAGCCTCTAACA
>SRR4252620_4614342___Sbg1
TAGCCTCTAACAGAAATGAGTACCAGGA
>SRR4252610_11703575___Sbg1
CCCCGGGGTGCACTCAGCCTCTAACAGAA
>SRR4252612_1346509___Sbg1
ACAGGCCTTGTATGCCGATTGTCTGCAAA
>SRR4252610_11306763___Sbg1
TAACAGAAATGAGTACCAAGAGCTTTCCT
>SRR4252609_13303208___Sbg1
CCCTGGGGTTCACTCAGCCTCTAACAGA
>SRR4252611_9287253___Sbg1
GGGGTTCACTCAGCCTCTAACAGAAA

```
>SRR4252610_8568504___Sbg1
CCTGGGGTGCACTCAGCCTCTAACAGAA
>SRR4252608_775464___Sbg1
GATTCACTCAGCCTCTAACAGAAATTAG
>SRR4252606_1134915___Sbg1
TCCTGGGGTTCACTCAGCCTCTAACAGA
>SRR4252608_12500585___Sbg1
TCCTGGGGTTCACTCAGCCTCTAACAGAA
>SRR4252612_1464046___Sbg1
TCTGTTAGAGGCTGAGTGCACCCCAGGG
>SRR4252607_12097042___Sbg1
TCTGTTAGAGGCTGAGTGCACCCCAGGGC
>SRR4252626_436565___Sbg1
AAAGCTCCTGGTACTCATTTCTGTTAGA
>SRR4252610_13263331___Sbg1
TTCTGTTAGAGGCTGAGTGCACCCCAGGG
>SRR4252606_7140174___Sbg1
TTCTGTTAGAGGCTGAGTGCACCCCAGG
>SRR4252610_9156423___Sbg1
TCATTTCTGTTAGAGGCTGAGTGCACCCC
>SRR4252611_890281___Sbg1
TTCTGTTAGAGGCTGAGTGCACCCCAGG
>SRR4252608_12604268___Sbg1
TCTGTTAGAGGCTGAGTGCACCCCAGGGC
>SRR4252614_5952640___Sbg1
CCCAGGAAAGCTCCTGGTACTCATTTCTGC
>SRR4252607_10862437___Sbg1
TTCTGTTAGAGGCTGAGTGCACCCCAGGG
>SRR4252611_4208636___Sbg1
TTCTGTTAGAGGCTGAGTGCACCCCAGG
>SRR4252612_3724657___Sbg1
TCATTTCTGTTAGAGGCTGAGTGCACT
>SRR4252608_6553401___Sbg1
CCATAGTGCGGCCAGAAGGATTATACCAA
>SRR4252611_11977242___Sbg1
TCATTTCTGTTAGAGGCTCAGTGCACC
>SRR4252609_4214778___Sbg1
TTCTGTTAGAGGCTGAGTGCACCCCAGGG
>SRR4252611_799872___Sbg1
TCCTGGTACTCATTTCTGTTAGAGGCTG
>SRR4252611_4935329___Sbg1
GTGCGGCCAGAAGGATTATACCAATTGA
>SRR4252612_3240186___Sbg1
GCCAGAAGGATTATACCAATTGAAAAA
>SRR4252605_7532328___Sbg1
ACACGAGGCAGCCGCCTTCACCCCCAGGAA
>SRR4252605_11658298___Sbg1
GAAAGCTCCTGGTACTCATTTCTGTTAGA
>SRR4252605_8983184___Sbg1
TTCTGTTAGAGGCTGAGTGCACCCCAGG
>SRR4252610_6331724___Sbg1
TTCTGTTAGAGGCTGAGTGCACCCCAGGG
>SRR4252610_12978685___Sbg1
TTCTGTTAGAGGCTGAGTGCACCCCAGA
>SRR4252611_10448649___Sbg1
CAGGGCCATAGTGCGGCCAGAAGGATTATA
>SRR4252610_841930___Sbg1
TTTGCAGACAATCGGCATACAAGGCCTGTG
>SRR4252625_3947725___Sbg1
TTCTGTTAGAGGCTGAGTGCACCCCAGGG
>SRR4252605_6746402___Sbg1
GAATGCTCCTGGTACTCATTTCTGTTAGA
>SRR4252608_5760231___Sbg1
TCATTTCTGTTAGAGGCTGAGTGCACCCC
>SRR4252619_10480187___Sbg1
TTCTGTTAGAGGCTGAGTGCACCCCAG
>SRR4252608_3846395___Sbg1
TCTGTTAGAGGCTGAGTGCACCCCAGGGC
>SRR4252612_8728197___Sbg1
TTCTGTTAGAGGCTGAGTGCACCCCAAGG
```

>SRR4252619_1709271___Sbg1
TACAAGGCCTGTGAGGTTGTCAGCCCCA
>SRR4252609_15313484___Sbg1
GAAAGCTCTTGGTACTCATTTCTGTTAGA
>SRR4252608_8339350___Sbg1
TCTGTTAGAGGCTGAGTGCACCCCAGGG
>SRR4252610_3581570___Sbg1
TCTGTTAGAGGCTGAGTGCACCCCAGGGC
>SRR4252621_13889626___Sbg1
ATTTCTGTTAGAGGCTGAGTACACCCCAGG
>SRR4252612_1205649___Sbg1
ATTTCTGTTAGAGGCTGAGTGCACCCC
>SRR4252605_6320752___Sbg1
AGGCCATAGTGCGGCCAGAAGGATTATA
>SRR4252610_9996413___Sbg1
TTCTGTTAGAGGCTGAGTGAACCCCAG
>SRR4252611_3108096___Sbg1
TTGCAGACAATCGGCATACAAGGCCTGTG
>SRR4252605_11773635___Sbg1
ATTTCTGTTAGAGGCTGAGTGCATCCC
>SRR4252612_1417902___Sbg1
TACGGCACCCATCTTTGCAGACAATCGGC
>SRR4252621_8158081___Sbg1
TCTGTTAGAGGCTGAGTGCACCCCAGGG
>SRR4252608_8370125___Sbg1
GCGGCCAGAAGGATTATACCAATTGAA
>SRR4252623_1761408___Sbg1
TCTGTTAGAGGCTGAGTGTACCCCAGGG
>SRR4252608_7869280___Sbg1
TTTGCAGACAATCGGCATACAGGGCCTGT
>SRR4252610_8237007___Sbg1
TTCTGTTAGAGGCTGAGTGCACCCCA
>SRR4252605_14328479___Sbg1
GGGCCATAGTGCGGCCAGAAGGATTATA
>SRR4252609_10777736___Sbg1
TCCTGGTACTCATTTCTGTTAGAAGCTG
>SRR4252607_14474293___Sbg1
TTTGCAGACAATCGGCATACAAGGCCTGT
>SRR4252609_11475654___Sbg1
TTCTGTTAGAGGCTGAGTGCACCCCAGG
>SRR4252605_2338569___Sbg1
TCAGGGCCATAGTGCGGCCAGAAGGATTA
>SRR4252619_5007340___Sbg1
TGTGAGGTTGTCAGCCCCACGAGGCAGCCG
>SRR4252607_9156812___Sbg1
AGGCCATAGTGCGGCCAGAAGGATTATA
>SRR4252612_8462492___Sbg1
TTCTGTTAGAGGCTGAGTGCACCCCAGGG
>SRR4252611_146529___Sbg1
CAATCGGCATACAAGGCCTGTGAGGTTG
>SRR4252625_7884394___Sbg1
TCTGTTAGAGGCTGAGTGCACCCCAGGGC
>SRR4252615_11460078___Sbg1
AGTGCACCCCAGGGCCATAGTGCGGCCAGA
>SRR4252621_10471995___Sbg1
GGGTCATAGTGCGGCCAGAAGGATTATA
>SRR4252621_7029086___Sbg1
TTTGCAGACAATCGGCATACAAGGCCTGTG
>SRR4252609_14147849___Sbg1
TTCTGTTAGAGGCTGAGTGCACCCCAGG
>SRR4252606_7134755___Sbg1
TTCTGTTAGAGGCTGAGTGCACCCCAGGG
>SRR4252606_2199137___Sbg1
TTTGCAGACAATCGGCATACAGGGCCTGTG
>SRR4252614_4228371___Sbg1
GAAAGCTCCTGGTACTCATTTCTGTTAGA
>SRR4252609_12047621___Sbg1
TTCTGTTAGAGGCTGAGTGCACCCCAGG
>SRR4252609_7757933___Sbg1
TTCTGTTAGAGGCTGAGTGCACCCCG

>SRR4252610_11344125___Sbg1
GAAAGCTCCTGGTACTCATTTCTGTTAGA
>SRR4252609_9154331___Sbg1
TTCTGTTAGAGGCTGAGTGCACCCCAGG
>SRR4252611_9783765___Sbg1
TTCTGTTAGAGGCTGAGTGCAACCCAGGG
>SRR4252624_8271722___Sbg1
TTCTGTTAGAGGCTGAGTGCACCCCAGG
>SRR4252612_1895211___Sbg1
TTCTGTTAGAGGCTGAGTGCACCCCAGGG
>SRR4252611_11397752___Sbg1
ATAGTGCGGCCAGAAGGATTATACCAATT
>SRR4252605_9442319___Sbg1
TACGGCACCCATCTTTGCAGACAATCGG
>SRR4252605_11643468___Sbg1
AGGTAAAGCTATCCCTATACAGGCCTA
>SRR4252611_6979826___Sbg1
CAGACAATCGGCATACAAGGCCTGTGAGG
>SRR4252605_7837085___Sbg1
TTTGCAGACAATCGGCATACAAGGCCTGTG
>SRR4252605_7452504___Sbg1
ACAGGGCCATAGTGCGGCCAGAAGGATTATA
>SRR4252605_9997204___Sbg1
TCTGTTAGAGGCTGAGTGCACCCCAGGGC
>SRR4252606_7905119___Sbg1
TAGTGCGGCCAGAAGGATTATACCAATT
>SRR4252608_828731___Sbg1
TACGGCACCCATCTTTGCAGACAATCGGCA
>SRR4252605_7834079___Sbg1
GAAGCTCCTGGTACTCATTTCTGTTAGA
>SRR4252613_5759538___Sbg1
GGCCAGAAGGATTATACCAATTGAAAAA
>SRR4252605_8867646___Sbg1
CTTTGCAGACAATCGGCATACAAGGCCTG
>SRR4252612_7598448___Sbg1
TTCTGTTAGAGGCTGAGTGCACCCCAT
>SRR4252612_6622706___Sbg1
AAAGCTCCTGGTACTCATTTCTGTTAGA
>SRR4252619_10799384___Sbg1
TCATTTCTGTTAGAGGCTGAGTGCACCC
>SRR4252605_5581161___Sbg1
TTCTGTTAGAGGCTGAGTGCACCCCAGGG
>SRR4252610_6505410___Sbg1
TTCTGTTAGAGGCTGAGTGCACCCCAG
>SRR4252611_14457693___Sbg1
TCCTGGTACTCATTTCTGTTAGAGGATG
>SRR4252614_6677793___Sbg1
CTTTGCAGACAATCGGCATACAAGGCCT
>SRR4252612_6499092___Sbg1
ATCGGCATACAAGGCCTGTGAGGTTGTCA
>SRR4252615_7784197___Sbg1
ATCGGCATACAAGGCCTGTGAGGTTGTCA
>SRR4252607_5180076___Sbg1
TTTGCAGACAATCGGCATACAAGGCCTGT
>SRR4252611_14314884___Sbg1
TTGCAGACAATCGGCATATAAGGCCTGT
>SRR4252612_125911___Sbg1
TTCTGTTAGAGGCTGAGTGCACCCCAGGG
>SRR4252606_9307821___Sbg1
TTCTGTTAGAGGCTGAGTGCACCCCAAGG
>SRR4252607_2339343___Sbg1
AAGCTCCTGGTACTCATTTCTGTTAGA
>SRR4252610_10690980___Sbg1
TCCTGGTACTCATTTCTGTTAGAGGTTG
>SRR4252622_4715764___Sbg1
TTGCAGACAATCGGCATACAAGGCCTGTG
>SRR4252605_8656046___Sbg1
TTCTGTTAGAGGCTGAGTGCACCCCA
>SRR4252621_2496438___Sbg1
TTCTGTTAGAGGCTGAGTGCACCCCAGGG

>SRR4252606_4889258___Sbg1
AGGGTCATAGTGCGGCCAGAAGGATTATA
>SRR4252623_7003723___Sbg1
TTGCAGACAATCGGCATACAAGGCCTG
>SRR4252611_3361408___Sbg1
TACAAGGCCTGTGAGGTTGTCAGCCCCAC
>SRR4252606_3575028___Sbg1
TACGGCACCCATCTTTGCAGACAATCGGC
>SRR4252605_5997141___Sbg1
TTCTGTTAGAGGCTGAGTGCACCCCAGGG
>SRR4252611_11511955___Sbg1
CCATAGTGCGGCCAGAAGGATTATACCAA
>SRR4252624_6480674___Sbg1
GGAGAGCTCCTGGTACTCATTTCTGTTAGA
>SRR4252624_2980029___Sbg1
TCATTTCTGTTAGAGGCTGAGTGCACCCC
>SRR4252612_8187113___Sbg1
TCCTGGTACTAATTTCTGTTAGAGGCTGA
>SRR4252606_273494___Sbg1
CACGAGGCAGCCGCCTTCACCCCCAGGAA
>SRR4252606_8427236___Sbg1
AAAGCTCCTGGTACTCATTTCTGTTAGA
>SRR4252609_16849424___Sbg1
TTCTGTTAGAGGCTGAGTGCACCCCAGG
>SRR4252611_126677___Sbg1
TTCTGTTAGAGGCTGAGTGCACCCCAGTG
>SRR4252606_5496875___Sbg1
TTGCAGACAATCGGCATACAAGGCCTGT
>SRR4252610_12133887___Sbg1
TTCTGTTAGAGGCTGAGTGCACCCCAGGG
>SRR4252609_15392729___Sbg1
TTCTGTTAGAGGCTGAGTGCACCCCAGGG
>SRR4252611_14020402___Sbg1
TTCTGTTAGAGGCTGAGTGCACCCCAGGG
>SRR4252615_910725___Sbg1
AGTGCACCCCAGGGCCATAGTGCGGCCAGA
>SRR4252619_2458359___Sbg1
TCATTTCTGTTAGAGGCTGAGTGCACCCT
>SRR4252610_8013788___Sbg1
TTTCTGTTAGAGGCTGAGTGCACCCT
>SRR4252610_8902740___Sbg1
TTCTGTTAGAGGCTGAGTGCTCCCCAGG
>SRR4252611_512096___Sbg1
TTCTGTTAGAGGCTGAGTGCTCCCCAGGG
>SRR4252609_12814906___Sbg1
TCTGTTAGAGGCTGAGTGCTCCCCAGGG
>SRR4252605_6165779___Sbg1
TTCTGTTAGAGGCTGAGTGCACCCCAGG
>SRR4252617_12129871___Sbg1
CATAGTGCGGCCAGAAGGATTATACCAATT
>SRR4252607_7215441___Sbg1
TTCTGTTAGAGGCTGAGTGCACCCCAGGT
>SRR4252610_7132500___Sbg1
TTCTGTTAGAGGCTGAGTGCACCCCAGGT
>SRR4252610_3835098___Sbg1
CGCCCAGAAGGATTATACCAATTGAAAAA
>SRR4252607_8798117___Sbg1
GGAGGTTACAGCACCCATCTTTGCAGACAAT
>SRR4252610_132837___Sbg1
TTCTGTTAGAGGCTGAGTGCACCCCAGGG
>SRR4252611_14489421___Sbg1
AATCGGCATACAAGGCCTGTGAGGTTGTCA
>SRR4252620_9339785___Sbg1
TCTGTTAGAGGCTGAGTGCACCCCAGGGT
>SRR4252606_9630245___Sbg1
GGGTCATAGTGCGGCCAGAAGGATTATA
>SRR4252614_8530076___Sbg1
ATAGTGCGGCCAGAAGGATTATACCAATT
>SRR4252606_4723831___Sbg1
TAGAGGCTGAGTGCACCCCAGGGCCATGG

>SRR4252611_2739231___Sbg1
GAAAGCTCCTGGTACTCATTTCTGTTAGA
>SRR4252610_7289704___Sbg1
TTCTGTTAGAGGCTGAGTGCACCCCAGGG
>SRR4252610_2628084___Sbg1
TTCTGTTAGAGGCTGAGTGCACCCCC
>SRR4252626_9568308___Sbg1
TTCTGTTAGAGGCTGAGTGCACCCCC
>SRR4252620_9141367___Sbg1
AATCGGCATACAAGGCCTGTGAGGTTGA
>SRR4252612_6911855___Sbg1
TCTGTTAGAGGCTGAGTGCACCCCAGGGT
>SRR4252610_12870442___Sbg1
TACAAGGCCTGTGAGGTTGTCTGCCCCAC
>SRR4252610_8024507___Sbg1
TTTGCAGACAATCGGCATACAAGGCT
>SRR4252606_917742___Sbg1
TCTGTTAGAGGCTGAGTGCACCCCAGGGC
>SRR4252611_8451594___Sbg1
TTGCAGACAATCGGCATACAAGGCCTGTG
>SRR4252608_6749456___Sbg1
TTGCAGACAATCGGCATACAAGGCCTG
>SRR4252606_5803392___Sbg1
TCATTTCTGTTAGAGGCTGAGTGCACCCCA
>SRR4252609_1791117___Sbg1
TTCTGTTAGAGGCTGAGTGCACCCCAG
>SRR4252605_697685___Sbg1
TTTGCAGACAATCGGCATACAAGGCCTGTG
>SRR4252620_10300694___Sbg1
TTTGCAGACAATCGGCATACAAGGCCTGTG
>SRR4252607_12473715___Sbg1
ACACGAGGCAGCCGCCTTTACCCCCAGGAA
>SRR4252616_7616984___Sbg1
TCAGAAGGATTATACCAATTGAAAAA
>SRR4252611_13961366___Sbg1
AGCTCCTGGTACTCATTTCTGTTAGA
>SRR4252605_8942285___Sbg1
ATTTCTGTTAGAGGCTGAATGCACCCC
>SRR4252625_12164732___Sbg1
TTCTGTTAGAGGCTGAGTGCACCCCAGGG
>SRR4252620_3627515___Sbg1
ACCAGAAGGATTATACCAATTGAAAAA
>SRR4252619_10290689___Sbg1
TTTCTGTTAGAGGCTGAGTGCACCCCAG
>SRR4252610_6771687___Sbg1
TTCTGTTAGAGGCTGAGTGCACCCCAGGG
>SRR4252611_4949952___Sbg1
ATTTCTGTTAGAGGCTGAGTGCACCC
>SRR4252619_3005560___Sbg1
TTCTGTTAGAGGCTGAGTGCACCCTAG
>SRR4252621_9355890___Sbg1
TTTGCAGACAATCGGCATACAAAGCCTGTA
>SRR4252605_561369___Sbg1
CCAGAAGGATTATACCAATTGAAAAA
>SRR4252609_4401109___Sbg1
TCTGTTAGAGGCTGAGTGCACCCCAGGGC
>SRR4252610_7830361___Sbg1
TCTGTTAGAGGCTGAGTGCACCCCAGGGT
>SRR4252611_7335795___Sbg1
TTGCAGACAATCGGCATACAGGGCCTGT
>SRR4252607_12657441___Sbg1
TTCTGTTAGAGGCTGAGTGCACCCCAGG
>SRR4252612_2295028___Sbg1
GAAAGCTCCTGGTACTCATTTCTGTTAGA
>SRR4252616_7467745___Sbg1
AAAGCTCCTGGTACTCATTTCTGTTAGA
>SRR4252610_4167963___Sbg1
AGAAGGCCCTTAGGGTGACGGGAGGTTA
>SRR4252605_10176110___Sbg1
TTGCAGACAATCGGCATACAAGGCCTGTG

>SRR4252608_7759625___Sbg1
TTCTGTTAGAGGCTGAGTACACCCCAG
>SRR4252622_9826262___Sbg1
TTCTGTTAGAGGCTGAGTGCACCCCA
>SRR4252611_14817627___Sbg1
TCTGTTAGAGGCTGAGTGCACCCCAGGG
>SRR4252607_13719745___Sbg1
TTCTGTTAGAGGCTGAGTGCACCCCAGG
>SRR4252607_9717420___Sbg1
TTCTGTTAGAGGCTGAGTGCACCCCAGGG
>SRR4252609_6943773___Sbg1
TCTGTTAGAGGCTGAGTGTACCCCAGGG
>SRR4252611_10405720___Sbg1
AAGTGCGGCCAGAAGGATTATACCAAT
>SRR4252626_6774919___Sbg1
TCATTTCTGTTAGAGGCTGAGTGCACCCC
>SRR4252606_6323197___Sbg1
TAGAGGCTGAGTGCACCCCAGGGCCATA
>SRR4252611_3400730___Sbg1
TGCCAGAAGGATTATACCAATTGAAAAA
>SRR4252619_13170909___Sbg1
ACGGCCAGAAGGATTATACCAATTGAAA
>SRR4252619_15530364___Sbg1
ACGGCCAGAAGGATTATACCAATTGAAAA
>SRR4252619_13623188___Sbg1
AGGAAAGCTCCTGGTACTCATTTCTGTTA
>SRR4252611_8896655___Sbg1
TACAAGGCCTGTGAGGTTGTCAACCCCA
>SRR4252612_108182___Sbg1
CTCCTGGTACTCATTTCTGTTAGAGGCT
>SRR4252607_11175759___Sbg1
TTCTGTTAGAGGCTGAGTGCACCCCAGGG
>SRR4252610_1672194___Sbg1
TTTGCAGACAATCGGCATACAAGGCCTA
>SRR4252618_9527551___Sbg1
TACGGCACCCATCTTTGCAGACAATCGGCA
>SRR4252622_6662007___Sbg1
TCATTTCTGTTAGAGGCTGAGTGCACCC
>SRR4252610_460977___Sbg1
TGGTACTCATTTCTGTTAGAGGCTGAGTG
>SRR4252611_2176368___Sbg1
TGGTACTCATTTCTGTTAGAGGCTGAGT
>SRR4252611_8166910___Sbg1
AAGCTCCTGGTACTCATTTCTGTTAGA
>SRR4252624_10523599___Sbg1
TTCTGTTAGAGGCTGAGTGCACCCCAGGG
>SRR4252611_10096925___Sbg1
GAAAGCTCCTGGTACTCATTTCTGTTAGA
>SRR4252608_968344___Sbg1
TCATTTCTGTTAGAGGCTGAGTGCACCCC
>SRR4252605_2535410___Sbg1
GTCGGCATACAAGGCCTGTGAGGTTGTCA
>SRR4252607_7265005___Sbg1
TTCTGTTAGAGGCTGAGTGCACCCCAGGT
>SRR4252612_5273936___Sbg1
AAAGCTCCTGGTACTCATTTCTGTTAGA
>SRR4252612_230150___Sbg1
TTCTGTTAGAGGCTGAGTGCACCCCAAG
>SRR4252611_9147673___Sbg1
TTCTGTTAGAGGCTGAGTGCACCCCAAGG
>SRR4252609_6288126___Sbg1
TTTGCAGACAATCGGCATACAAGGCCTGTG
>SRR4252606_9837347___Sbg1
TTCTGTTAGAGGCTGAGTGCACCCCAGA
>SRR4252626_2625211___Sbg1
TTCTGTTAGAGGCTGAGTGCACCCCAGA
>SRR4252607_13060249___Sbg1
TTCTGTTAGAGGCTGAGTGCACTCCAT
>SRR4252605_13933137___Sbg1
ATCGGCATACAAGGCCTGTGAGGTTGTCA

>SRR4252605_10163957___Sbg1
TCTTTGCAGACAATCGGCATACGAGGCCT
>SRR4252611_9770555___Sbg1
TCATTTCTGTTAGAGGCTGAGTGCACT
>SRR4252611_12158651___Sbg1
AGTGCGGCCAGAAGGATTATACCAATTG
>SRR4252611_4240005___Sbg1
ATTCGGCATACAAGGCCTGTGAGGTTGTCA
>SRR4252619_2907923___Sbg1
TCCTGGTACTCATTTCTGTTAGAGGCTG
>SRR4252611_13084577___Sbg1
TTCTGTTAGAGGCTGAGTGCACACCAGGG
>SRR4252624_5329599___Sbg1
TTCTGTTAGAGGCTGAGTGCACACCAGG
>SRR4252609_10778951___Sbg1
TTCTGTTAGAGGCTGAGTGTACCCCAGG
>SRR4252607_6864638___Sbg1
TTTGCAGACAATCGGCATACAAGGCCTGTG
>SRR4252606_2477159___Sbg1
TCTGTTAGAGGCTGAGTGCACCCCAG
>SRR4252609_15727468___Sbg1
TTCTGTTAGAGGCTGAGTGCACCCCAGGC
>SRR4252611_12720845___Sbg1
TTGCAGACAATCGGCATAAAAGGCCTGTG
>SRR4252611_1296238___Sbg1
AGAGGCCATAGTGCGGCCAGAAGGATTA
>SRR4252611_5991923___Sbg1
AGAGGCCATAGTGCGGCCAGAAGGATTA
>SRR4252610_1749857___Sbg1
TTCTGTTAGAGGCTGAGTGCACCCCAGT
>SRR4252607_9941946___Sbg1
TTCTGTTAGAGGCTGAGTGAACCCCAGGG
>SRR4252610_4576960___Sbg1
CTCCTGGTACTCATTTCTGTTAGAGGCTG
>SRR4252618_4941952___Sbg1
TTACGGCACCCATCTTTGCAGACAATC
>SRR4252612_941146___Sbg1
GCTGCCAGAAGGATTATACCAATTGAAA
>SRR4252611_9321766___Sbg1
TACAAGGCCTGTGAGGTTGTCAGCCCCAT
>SRR4252609_1133214___Sbg1
TTCTGTTAGAGGCTGAGTGAACCCCAG
>SRR4252611_14562703___Sbg1
TTCTGTTAGAGGCTGAGTGAACCCCAGG
>SRR4252610_4257744___Sbg1
TTTGCAGACAATCGGCATACAAGGCCTGT
>SRR4252609_15417228___Sbg1
GCACCCATCTTTTCAGATATTCGGCATACA
>SRR4252605_12906126___Sbg1
ACCCATCTTTTCAGATATTCGGCATACA
>SRR4252607_10438693___Sbg1
ACCCATCTTTTCAGATATTCGGCATACA
>SRR4252611_10891063___Sbg1
ACCCATCTTTTCAGATATTCGGCATACA
>SRR4252612_4840208___Sbg1
ACCCATCTTTTCAGATATTCGGCATACA
>SRR4252606_8409109___Sbg1
TACAAGGCCTGTGAGGTTGTCAGCCCCAT
>SRR4252614_9704697___Sbg1
TCATTTCTGTTAGAGGCTGTGTGCACCCC
>SRR4252610_3679080___Sbg6
GCGCTCCTAGGAGCAATAGTGTGAACGTCCA
>SRR4252623_12759405___Sbg6
GCGCTCCTAGGAGCAATAGTGTGAACGTCCA
>SRR4252610_459167___Sbg6
GCGCTCCTAGGAGCAATAGTGTGAACGTCCA
>SRR4252621_11142724___Sbg6
GCGCTCCTAGGAGCAATAGTGTGAACGTCCA
>SRR4252610_5194088___Sbg6
CCTAGGAGCAATAGTGTGAACGTCCAA

>SRR4252617_12434310___Sbg6
CCAAATCCGGCGCGAGAGTTCGCCACT
>SRR4252611_2058968___Sbg6
GCGCTCCTAGGAGCAATAGTGTGAACGTCCA
>SRR4252608_4335493___Sbg6
GCCAAGTTTCGTACCCAAATAGAGTCCA
>SRR4252609_1712922___Sbg6
TCCTAGGAGCAATAGTGTGAACGTCCAA
>SRR4252617_12634952___Sbg6
AGATGGCATGTAAAAGAACCCTTGGGT
>SRR4252606_2537926___Sbg6
GGAGTAATAGTGTGAACGTCCAAATCCGG
>SRR4252610_13524763___Sbg6
GCGCTCCTAGGAGCAATAGTGTGAACGTCCA
>SRR4252617_9555700___Sbg6
CCTAGGAGCAATAGTGTGAACGTCCAAA
>SRR4252608_1945266___Sbg6
TCCTAGGAGCAATAGTGTGAACGTCCAA
>SRR4252608_2982736___Sbg6
GCGCTCCTAGGAGCAATAGTGTGAACGTCCA
>SRR4252623_3454855___Sbg6
GCGCTCCTAGGAGCAATAGTGTGAACGTCCA
>SRR4252607_8405354___Sbg6
CCTAGGAGCAATAGTGTGAACGTCCA
>SRR4252612_5930423___Sbg6
GGAGCAATAGTGTGAACGTCCAAATCCG
>SRR4252606_6226127___Sbg6
TCGGTTAGATGGCATGTAAAAGAACTCT
>SRR4252606_1606396___Sbg6
TTCGTACCCTCGGCAAAATTAAATCCCA
>SRR4252621_13017421___Sbg6
CTCCTAGGAGCAATAGTGTGAACGTCCAA
>SRR4252610_2279752___Sbg6
CCTAGGAGCAATAGTGTGAACGTCCAA
>SRR4252619_175596___Sbg6
TTAGATGGCATGTAAAAGAACCCTTGGGT
>SRR4252608_426944___Sbg6
GGAGCAATAGTGTGAACGTCCAAATCCG
>SRR4252605_913160___Sbg6
GGAGCAATAGTGTGAACGTCCAAATCCG
>SRR4252607_8136040___Sbg6
TGGCATGTAAAAGAACCCTTGGGTACA
>SRR4252610_9023856___Sbg6
TTAGGGTACAAAAAATCCATGGCACGCA
>SRR4252611_2942961___Sbg6
CCTAGGAGCAATAGTGTGAACGTCCAA
>SRR4252610_4790810___Sbg6
TCCTAGGAGCAATAGTGTGAACGTCCAA
>SRR4252623_7164796___Sbg6
GCCAAGTTTCGTACCCAAATAGAGTC
>SRR4252624_6321377___Sbg6
GCGCTCCTAGGAGCAATAGTGTGAACGTCCA
>SRR4252605_9658291___Sbg6
AGGAGCAATAGTGTGAACGTCCAAAT
>SRR4252612_6059371___Sbg6
CCTAGGAGCAATAGTGTGAACGTCCA
>SRR4252606_299282___Sbg6
GCTCCTAGGAGCAATAGTGTGAACGTCCA
>SRR4252610_2372995___Sbg6
CCGGGTCTGACAACGACGTAGTAAGTCGAGA
>SRR4252608_12842929___Sbg6
GGAGCAATAGTGTGAACGTCCAAATCCG
>SRR4252607_7493259___Sbg6
TCCTAGGAGCAATAGTGTGAACGTCCAA
>SRR4252606_7967337___Sbg6
GTACACTTCGTACCCTCGGCAAAATTA
>SRR4252610_12381062___Sbg6
TCGATTAGATGGCATGTAAAAGAACCCTT
>SRR4252623_1697782___Sbg6
GCGCTCCTAGGAGCAATAGTGTGAACGTCCA

>SRR4252621_11582595___Sbg6
GGAGCAATAGTGTGAACGTCCAAATC
>SRR4252613_7489240___Sbg6
TCCAAGTTTCGTACCCAAATAGAGTCCACA
>SRR4252611_7722483___Sbg6
AGTTTCGTACCCAAATAGAGTCCACACTA
>SRR4252610_11962614___Sbg6
GCGCTCCTAGGAGCAATAGTGTGAACGTCCA
>SRR4252611_3124760___Sbg6
CGGCCGGGTCTGACAACGACGTAGTAAGTC
>SRR4252605_243422___Sbg6
AAGTCGAGATTCAGCCCATTAGATACA
>SRR4252607_4225513___Sbg6
TCGGCCGGGTCTGACAACGACGTAGTAAGT
>SRR4252610_12199850___Sbg6
GCGCTCCTAGGAGCAATAGTGTGAACGTCCA
>SRR4252607_14133208___Sbg6
TTAGATGGCATGTAAAAGAACACTTGG
>SRR4252610_9606836___Sbg6
TAGATGGCATGTAAAAGAACACTTGGGT
>SRR4252620_10315285___Sbg6
TAGATGGCATGTAAAAGAACACTTGGGTA
>SRR4252620_6276975___Sbg6
TAGATGGCATGTAAAAGAACACTTGGG
>SRR4252620_752497___Sbg6
TAGATGGCATGTAAAAGAACACTTGGGTA
>SRR4252608_13224604___Sbg6
GCGCTCCTAGGAGCAATAGTGTGAACGTCCA
>SRR4252609_6527735___Sbg6
TCTAGGAGCAATAGTGTGAACGTCCAA
>SRR4252610_5420846___Sbg6
AAGTCGAGATTCAGCCCATTAGATACA
>SRR4252612_5804378___Sbg6
TAGATGGCATGTAAAAGAACCCTTGGGTAC
>SRR4252619_5558386___Sbg6
GCGCTCCTAGGAGCAATAGTGTGAACGTCCA
>SRR4252621_2342851___Sbg6
GCGCTCCTAGGAGCAATAGTGTGAACGTCCA
>SRR4252612_8858372___Sbg6
TCCTAGGAGCAATAGTGTGAACGTCCA
>SRR4252608_12135334___Sbg6
CTCCTAGGAGCAATAGTGTGAACGTCCAA
>SRR4252626_361135___Sbg6
TCCTAGGAGCAATAGTGTGAACGTCCAA
>SRR4252605_3869441___Sbg6
AAGTCGAGATTCAGCCCATTAGATACA
>SRR4252611_4003784___Sbg6
AATTAAATCCCGGCCAAGTTTCGTACCCA
>SRR4252605_6184829___Sbg6
TACACTTCGTACCCTCGGCAAAATTA
>SRR4252612_561095___Sbg6
TGGCATGTAAAAGAACCCTTGGGTACAC
>SRR4252606_2972109___Sbg6
GCGCTCCTAGGAGCAATAGTGTGAACGTCCA
>SRR4252610_714327___Sbg6
GCGCTCCTAGGAGCAATAGTGTGAACGTCCA
>SRR4252611_2885173___Sbg6
TTCGTACCCAAATAGAGTTCACACTAGC
>SRR4252605_5708912___Sbg6
TGGCATGTAAAAGAACCCTTGGGTATACTT
>SRR4252606_7003632___Sbg6
GCGCTCCTAGGAGCAATAGTGTGAACGTCCA
>SRR4252612_7233048___Sbg6
CCTAGGAGCAATAGTGTGAACGTCCAA
>SRR4252610_4288386___Sbg6
TCCTAGAAGCAATAGTGTGAACGTCCAA
>SRR4252606_1306794___Sbg6
CCTAGAAGCAATAGTGTGAACGTCCAA
>SRR4252610_10196553___Sbg6
GCGCTCCTAGGAGCAATAGTGTGAACGTCCA

>SRR4252605_28125___Sbg6
GCGCTCCTAGGAGCAATAGTGTGAACGTCCA
>SRR4252605_11406736___Sbg6
AAGTCGAGATTCAGCCCATTAGATACA
>SRR4252611_7692208___Sbg6
TCCTAGGAGCAATAGTGTGAACGTCCAA
>SRR4252610_10710624___Sbg6
GCAAAATTAAATCCTGGCCAAGTTTCGT
>SRR4252606_6223366___Sbg6
GGTCGAGATTCAGCCCATTAGATACA
>SRR4252610_7720630___Sbg6
GCGCTCCTAGGAGCAATAGTGTGAACGTCCA
>SRR4252609_4749226___Sbg6
CCTAGGAGCAATAGTGTGAACGTCCAA
>SRR4252608_12837493___Sbg6
TGTAAAAGAACCCTTGGGTACACTTCGTA
>SRR4252610_4991544___Sbg6
CCTAGGAGCAATAGTGTGAACGTCCAA
>SRR4252608_4058225___Sbg6
GTTTCGTACACAAATAGAGTCCACACTA
>SRR4252610_4532685___Sbg6
GTTTCGTACACAAATAGAGTCCACACTA
>SRR4252619_3777388___Sbg6
GTTTCGTACACAAATAGAGTCCACACTA
>SRR4252610_4120531___Sbg6
GCGCTCCTAGGAGCAATAGTGTGAACGTCCA
>SRR4252611_13017336___Sbg6
CACTTCATACCCTCGGCAAAATTAAA
>SRR4252610_9407677___Sbg6
AAGTCGAGATTCAGCCCATTAGATACA
>SRR4252605_2346912___Sbg6
ACGCTCCTAGGAGCAATAGTGTGAACGTCCA
>SRR4252607_8154992___Sbg6
ACGCTCCTAGGAGCAATAGTGTGAACGTCCA
>SRR4252608_9267099___Sbg6
ACGCTCCTAGGAGCAATAGTGTGAACGTCCA
>SRR4252609_15705849___Sbg6
ACGCTCCTAGGAGCAATAGTGTGAACGTCCA
>SRR4252610_11338917___Sbg6
ACGCTCCTAGGAGCAATAGTGTGAACGTCCA
>SRR4252610_1844343___Sbg6
ACGCTCCTAGGAGCAATAGTGTGAACGTCCA
>SRR4252610_1985723___Sbg6
ACGCTCCTAGGAGCAATAGTGTGAACGTCCA
>SRR4252610_4973818___Sbg6
ACGCTCCTAGGAGCAATAGTGTGAACGTCCA
>SRR4252610_6472641___Sbg6
ACGCTCCTAGGAGCAATAGTGTGAACGTCCA
>SRR4252611_5695183___Sbg6
ACGCTCCTAGGAGCAATAGTGTGAACGTCCA
>SRR4252612_2292611___Sbg6
ACGCTCCTAGGAGCAATAGTGTGAACGTCCA
>SRR4252612_3348573___Sbg6
ACGCTCCTAGGAGCAATAGTGTGAACGTCCA
>SRR4252624_6172583___Sbg6
ACGCTCCTAGGAGCAATAGTGTGAACGTCCA
>SRR4252615_214317___Sbg6
AAATAGAGTCCACACTAGCGCTCCTAG
>SRR4252606_6007892___Sbg6
GCGCTCCTAGGAGCAATAGTGTGAACGTCCA
>SRR4252619_9987061___Sbg6
GCGCTCCTAGGAGCAATAGTGTGAACGTCCA
>SRR4252625_9820966___Sbg6
CATGGCACGCATGTCATCGGTTAGAT
>SRR4252610_6690273___Sbg6
TCCTAGGAGCAATAGTGTGAACGTCCAA
>SRR4252611_12801995___Sbg6
ACTCGGAGAAACTCTCATCCCTGGGGTCG
>SRR4252623_7707058___Sbg6
GCGCTCCTAGGAGCAATAGTGTGAACGTCCA

>SRR4252623_8723213___Sbg6
CTCCTAGGAGCAATAGTGTGAACGTCCAA
>SRR4252623_3303266___Sbg6
CCTAGGAGCAATAGTGTGAACGTCCAA
>SRR4252610_4669009___Sbg6
TCCTAGGAGCAATAGTGTGAACGTCCA
>SRR4252607_5372536___Sbg6
CCTAGGAGCAATAGTGTGAACGTCCAA
>SRR4252621_6204151___Sbg6
GCCAAGTTTCGTACCCAAATAGAGTCCA
>SRR4252606_5272258___Sbg6
TGCCAACTCGGAGAAACTCTCATCCT
>SRR4252619_7425793___Sbg6
AGTTTCGTACCCAAATAGAGTCCACACTA
>SRR4252609_6228505___Sbg6
CTGGGGTCGGCCGGGTCTGACAACGA
>SRR4252607_3968387___Sbg6
TTAGATGGCATGTAAAAGAACCCTTGG
>SRR4252605_11648477___Sbg6
TCCTAGGAGCAATAGTGTGAACGTCCA
>SRR4252610_5015828___Sbg6
GCGCTCCTAGGAGCAATAGTGTGAACGTCCA
>SRR4252610_310886___Sbg6
TCCTAGGAGCAATAGTGTGAACGTCCAA
>SRR4252606_9975716___Sbg6
TCGATTAGATGGCATGTAAAAGAACCCTT
>SRR4252621_13290270___Sbg6
CGGTTAGATGGCATGTAAAAGAACCCT
>SRR4252605_4118842___Sbg6
TGGCATGTAAAAGAACCCTTGGGTACACTT
>SRR4252611_5590177___Sbg6
CCTAGGAGCAATAGTGTGAACGTCCAA
>SRR4252610_180824___Sbg6
GCGCTCCTAGGAGCAATAGTGTGAACGTCCA
>SRR4252612_5269681___Sbg6
TCGGCCGGGTCTGACAACGACGTAGTA
>SRR4252610_9921724___Sbg6
CCTAGGAGCAATAGTGTGAACGTCCA
>SRR4252612_3047796___Sbg6
CAAGTTTCGTACCCAAATAGAGTCCA
>SRR4252623_2142448___Sbg6
GCGCTCCTAGGAGCAATAGTGTGAACGTCCA
>SRR4252610_8427202___Sbg6
AAGTCGAGATTCAGCCCATTAGATACA
>SRR4252605_3796974___Sbg6
ACGCTCCTAGGAGCAATAGTGTGAACGTCCA
>SRR4252605_5752774___Sbg6
ACGCTCCTAGGAGCAATAGTGTGAACGTCCA
>SRR4252606_5853031___Sbg6
ACGCTCCTAGGAGCAATAGTGTGAACGTCCA
>SRR4252606_6110367___Sbg6
ACGCTCCTAGGAGCAATAGTGTGAACGTCCA
>SRR4252606_7885469___Sbg6
ACGCTCCTAGGAGCAATAGTGTGAACGTCCA
>SRR4252607_4662426___Sbg6
ACGCTCCTAGGAGCAATAGTGTGAACGTCCA
>SRR4252608_2933932___Sbg6
ACGCTCCTAGGAGCAATAGTGTGAACGTCCA
>SRR4252608_3158773___Sbg6
ACGCTCCTAGGAGCAATAGTGTGAACGTCCA
>SRR4252608_579574___Sbg6
ACGCTCCTAGGAGCAATAGTGTGAACGTCCA
>SRR4252610_1756785___Sbg6
ACGCTCCTAGGAGCAATAGTGTGAACGTCCA
>SRR4252610_378132___Sbg6
ACGCTCCTAGGAGCAATAGTGTGAACGTCCA
>SRR4252610_5139479___Sbg6
ACGCTCCTAGGAGCAATAGTGTGAACGTCCA
>SRR4252610_5454626___Sbg6
ACGCTCCTAGGAGCAATAGTGTGAACGTCCA

```
>SRR4252610_5686918___Sbg6
ACGCTCCTAGGAGCAATAGTGTGAACGTCCA
>SRR4252610_6030052___Sbg6
ACGCTCCTAGGAGCAATAGTGTGAACGTCCA
>SRR4252610_7805211___Sbg6
ACGCTCCTAGGAGCAATAGTGTGAACGTCCA
>SRR4252610_9643374___Sbg6
ACGCTCCTAGGAGCAATAGTGTGAACGTCCA
>SRR4252622_6972554___Sbg6
ACGCTCCTAGGAGCAATAGTGTGAACGTCCA
>SRR4252623_9746960___Sbg6
ACGCTCCTAGGAGCAATAGTGTGAACGTCCA
>SRR4252606_5579613___Sbg6
GCGCTCCTAGGAGCAATAGTGTGAACGTCCA
>SRR4252606_4255186___Sbg6
ATCCCTGGGGTCGGCCGGGTCTGACAACG
>SRR4252610_12727874___Sbg6
TGGCATGTAAAAGAACCCTTGGGTATACT
>SRR4252610_11974408___Sbg6
CTCCTAGGAGCAATAGTGTGAACGTCCA
>SRR4252606_7290432___Sbg6
AATCGAGATTCAGCCCATTAGATACA
>SRR4252610_218131___Sbg6
TAGATGGCATGTAAAAGAACCCTTGGGT
>SRR4252608_9278211___Sbg6
GCGCTCCTAGGAGCAATAGTGTGAACGTCCA
>SRR4252624_9338764___Sbg6
AAATTAAATCCCGGCCAAGTTTCGTACCCA
>SRR4252606_6282060___Sbg6
TCGGTTAGATGGCATGTAAAAGAACCCT
>SRR4252620_6773826___Sbg6
GCGCTCCTAGGAGCAATAGTGTGAACGTCCA
>SRR4252621_13998639___Sbg6
GCGCTCCTAGGAGCAATAGTGTGAACGTCCA
>SRR4252624_10141497___Sbg6
GCGCTCCTAGGAGCAATAGTGTGAACGTCCA
>SRR4252610_211387___Sbg6
CCGCTCCTAGGAGCAATAGTGTGAACGTCCA
>SRR4252610_6280947___Sbg6
GCGCTCCTAGGAGCAATAGTGTGAACGTCCA
>SRR4252621_1518932___Sbg6
GCGCTCCTAGGAGCAATAGTGTGAACGTCCA
>SRR4252615_10810265___Sbg6
ACCAAGTTTCGTACCCAAATAGAGTCCA
>SRR4252605_5115360___Sbg6
ACGCTCCTAGGAGCAATAGTGTGAACGTCCA
>SRR4252606_6633431___Sbg6
ACGCTCCTAGGAGCAATAGTGTGAACGTCCA
>SRR4252607_11397117___Sbg6
ACGCTCCTAGGAGCAATAGTGTGAACGTCCA
>SRR4252608_11825880___Sbg6
ACGCTCCTAGGAGCAATAGTGTGAACGTCCA
>SRR4252608_12879180___Sbg6
ACGCTCCTAGGAGCAATAGTGTGAACGTCCA
>SRR4252610_10216609___Sbg6
ACGCTCCTAGGAGCAATAGTGTGAACGTCCA
>SRR4252610_1112741___Sbg6
ACGCTCCTAGGAGCAATAGTGTGAACGTCCA
>SRR4252610_11256798___Sbg6
ACGCTCCTAGGAGCAATAGTGTGAACGTCCA
>SRR4252610_13431712___Sbg6
ACGCTCCTAGGAGCAATAGTGTGAACGTCCA
>SRR4252610_4919235___Sbg6
ACGCTCCTAGGAGCAATAGTGTGAACGTCCA
>SRR4252610_620316___Sbg6
ACGCTCCTAGGAGCAATAGTGTGAACGTCCA
>SRR4252610_9477435___Sbg6
ACGCTCCTAGGAGCAATAGTGTGAACGTCCA
>SRR4252610_9595671___Sbg6
ACGCTCCTAGGAGCAATAGTGTGAACGTCCA
```

>SRR4252611_10474050___Sbg6
ACGCTCCTAGGAGCAATAGTGTGAACGTCCA
>SRR4252607_11825231___Sbg6
GCGCTCCTAGGAGCAATAGTGTGAACGTCCA
>SRR4252609_14527186___Sbg6
CCTAGGAGCAATAGTGTGAACGTCCAA
>SRR4252624_2352769___Sbg6
GATGGCATGTAAAAGAACCCTTGGGTACACT
>SRR4252609_10648295___Sbg6
TCCTATGAGCAATAGTGTGAACGTCCAA
>SRR4252610_9180990___Sbg6
TCCTATGAGCAATAGTGTGAACGTCCAA
>SRR4252613_5695068___Sbg6
CCTATGAGCAATAGTGTGAACGTCCAA
>SRR4252620_5057395___Sbg6
GCGCTCCTAGGAGCAATAGTGTGAACGTCCA
>SRR4252608_1732377___Sbg6
CTCCTAGGAGCAATAGTGTGAACGTCCAA
>SRR4252605_2266095___Sbg6
GCGCTCCTAGGAGCAATAGTGTGAACGTCCA
>SRR4252611_11601464___Sbg6
TTCGTACCCAAATAGAGTCCAAACTAGC
>SRR4252611_11657443___Sbg6
TTCGTACCCAAATAGAGTCCAAACTAGC
>SRR4252612_9080487___Sbg6
GCGCTCCTAGGAGCAATAGTGTGAACGTCCA
>SRR4252608_1153984___Sbg6
CTCCTAGGAGCAATAGTGTGAACGTCCAA
>SRR4252610_9632819___Sbg6
GGTCAAGTTTCGTACCCAAATAGAGTCCA
>SRR4252610_8567169___Sbg6
GCGCTCCTAGGAGCAATAGTGTGAACGTCCA
>SRR4252612_4047529___Sbg6
TTTTAGGGTACAAAAAATCCATGGCA
>SRR4252608_8260660___Sbg6
TGCCAACTCGGAGAAACTCTCATCCCTG
>SRR4252612_8989265___Sbg6
GCGCTCCTAGGAGCAATAGTGTGAACGTCCA
>SRR4252611_1262131___Sbg6
AAGTTTCGTACCCAAATAGAGTCCACACCT
>SRR4252605_442784___Sbg6
TCCTAGGAGCAATAGTGTGAACGTCCAAA
>SRR4252606_9738107___Sbg6
GCGCTCCTAGGAGCAATAGTGTGAACGTCCA
>SRR4252610_1059993___Sbg6
GCGCTCCTAGGAGCAATAGTGTGAACGTCCA
>SRR4252610_11793056___Sbg6
ATCCTAGGAGCAATAGTGTGAACGTCCA
>SRR4252606_8818933___Sbg6
TCCTAGGAGCAATAGTGTGAACGTCCA
>SRR4252605_2259077___Sbg6
GCGCTCCTAGGAGCAATAGTGTGAACGTCCA
>SRR4252611_11050832___Sbg6
CCTAGGAGCAATAGTGTGAACGTCCAA
>SRR4252611_14820375___Sbg6
GCTCCTATGAGCAATAGTGTGAACGTCCA
>SRR4252605_1310552___Sbg6
CCTAGGAACAATAGTGTGAACGTCCAA
>SRR4252612_1538281___Sbg6
CCTAGGAGCAATAGTGTGAACGTCCAA
>SRR4252610_13026458___Sbg6
GCGCTCCTAGGAGCAATAGTGTGAACGTCCA
>SRR4252606_7806173___Sbg6
CGCTCCTAGGAGCAATAGTGTGAACGTCCA
>SRR4252608_5448734___Sbg6
TGAGCAATAGTGTGAACGTCCAAATCCG
>SRR4252622_3279741___Sbg6
TAGATGGCATGTAAAAGAACCCTTGGGT
>SRR4252605_9032652___Sbg6
TTAGATGGCATGTAAAAGAACCCTTGGG

>SRR4252611_2257981___Sbg6
TGGCATGTAAAAGAACCCTTGGGTACACTTT
>SRR4252605_10373210___Sbg6
CCTAGGAGCAATAGTGTGAACGTCCAA
>SRR4252608_11074829___Sbg6
CCTAGGAGCAATAGTGTGAACGTCCAA
>SRR4252610_10562319___Sbg6
GCGCTCCTAGGAGCAATAGTGTGAACGTCCA
>SRR4252610_1521356___Sbg6
AAGTGTGAACGTCCAAATCCGGCGCGAGA
>SRR4252608_4798109___Sbg6
CTCCTAGGAGCAATAGTGTGAACGTCCAA
>SRR4252613_1106146___Sbg6
TTAGATGGCATGTAAAAGAACCCTTGGT
>SRR4252610_1232160___Sbg6
GCGCTCCTAGGAGCAATAGTGTGAACGTCCA
>SRR4252606_2817974___Sbg6
GCGCTCCTAGGAGCAATAGTGTGAACGTCCA
>SRR4252610_7108538___Sbg6
TCGTCGGATTTTTAGGGTACAAAAAATCC
>SRR4252624_1431010___Sbg6
CCAAGTTTCGTACCCAAATAGAGTCCACA
>SRR4252607_9095870___Sbg6
TCCTAAGAGCAATAGTGTGAACGTCCAA
>SRR4252605_3534521___Sbg6
AATTAAATCCCGGCCAAGTTTCGTACCCA
>SRR4252613_8588017___Sbg6
TTAGATGGCATGTAAAAGAACCCTTGGG
>SRR4252610_3031169___Sbg6
GAGCTCCTAGGAGCAATAGTGTGAACGTCCA
>SRR4252619_4652988___Sbg6
GAGCTCCTAGGAGCAATAGTGTGAACGTCCA
>SRR4252610_5147515___Sbg6
AGCTCCTAGGAGCAATAGTGTGAACGTCCA
>SRR4252605_8843998___Sbg6
AAAGAACCCTTGGGTACACTTCGTACCCT
>SRR4252610_2762775___Sbg6
CCTAGGAGCAATAGTGTGAACGTCCAA
>SRR4252612_8330920___Sbg6
TTCGTACCCTCGGCAAAATTAAATCCTG
>SRR4252621_12490621___Sbg6
TCGATTAGATGGCATGTAAAAGAACCCTT
>SRR4252605_14169423___Sbg6
CCTAGGAGCAATAGTGTGAACGTCCAA
>SRR4252610_4229993___Sbg6
GCGCTCCTAGGAGCAATAGTGTGAACGTCCA
>SRR4252609_10978046___Sbg6
GCGCTCCTAGGAGCAATAGTGTGAACGTCCA
>SRR4252610_721862___Sbg6
GAGCTCCTAGGAGCAATAGTGTGAACGTCCA
>SRR4252612_9091171___Sbg6
TAGATGGCATGTAAAAGAACCCTTGGG
>SRR4252611_6188986___Sbg6
TTCGTACCCAAATAGAGTCCACACTAACG
>SRR4252610_6553486___Sbg6
GCGCTCCTAGGAGCAATAGTGTGAACGTCCA
>SRR4252616_1715603___Sbg6
TCGGTTAGATGGCATGTAAAAGAACCCTTG
>SRR4252607_4038797___Sbg6
TCTAGGAGCAATAGTGTGAACGTCCAA
>SRR4252609_13518329___Sbg6
TCTAGGAGCAATAGTGTGAACGTCCAA
>SRR4252611_1400045___Sbg6
TCTAGGAGCAATAGTGTGAACGTCCAA
>SRR4252605_13697453___Sbg6
GCGCTCCTAGGAGCAATAGTGTGAACGTCCA
>SRR4252610_4657491___Sbg6
CCTAGGAGCAATAGTGTGAACGTCCA
>SRR4252606_867587___Sbg6
GCGCTCCTAGGAGCAATAGTGTGAACGTCCA

>SRR4252610_5673664___Sbg6
TCCTAGGAGCAATAGTGTGAACGTCCAA
>SRR4252611_7749561___Sbg6
TCCTAGGAGCAATAGTGTGAACGTCCAA
>SRR4252605_3138142___Sbg6
ATCAAAAAATCCATGGCACGCATGTCA
>SRR4252606_40447___Sbg6
CCTAGGAGCAATAGTGTGAACGTCCAA
>SRR4252606_8858609___Sbg6
CTCCTAGGAGCAATAGTGTGAACGTCCA
>SRR4252621_13279140___Sbg6
GCGCTCCTAGGAGCAATAGTGTGAACGTCCA
>SRR4252622_10148205___Sbg6
ACAAGTTTCGTACCCAAATAGAGTCCACA
>SRR4252611_12674801___Sbg6
AGGGTACAAAAAATCCATGGCACGCA
>SRR4252610_7908626___Sbg6
CGATTAGATGGCATGTAAAAGAACCCTTC
>SRR4252610_7954718___Sbg6
CGATTAGATGGCATGTAAAAGAACCCTTC
>SRR4252606_4043009___Sbg6
GCGCTCCTAGGAGCAATAGTGTGAACGTCCA
>SRR4252611_1239451___Sbg6
TCGGAGAAACTCTCATCCCTGGGGTCGGC
>SRR4252608_7015056___Sbg6
CCTAGGAGCAATAGTGTGAACGTCCAA
>SRR4252607_10435626___Sbg6
TTCGTACCCAAATAGAGTCCACACTAGC
>SRR4252610_12318748___Sbg6
GCGCTCCTAGGAGCAATAGTGTGAACGTCCA
>SRR4252610_5432434___Sbg6
ATCCTAGGAGCAATAGTGTGAACGTCCA
>SRR4252619_11926309___Sbg6
TCGTACCCAAATAGAGTCCACACTAGC
>SRR4252608_4969824___Sbg6
AGGTCGTCGGATTTTTAGGGTACAAAAA
>SRR4252611_11480821___Sbg6
AGGTCGTCGGATTTTTAGGGTACAAAAAA
>SRR4252605_4042329___Sbg6
GGTCGTCGGATTTTTAGGGTACAAAAAA
>SRR4252619_12012710___Sbg6
CCTAGGAGAAATAGTGTGAACGTCCAA
>SRR4252619_6686274___Sbg6
TCGGTTAGATGGCATGTAAAAGAACCCT
>SRR4252605_14450620___Sbg6
ACGCTCCTAGGAGCAATAGTGTGAACGTCCA
>SRR4252606_2936832___Sbg6
ACGCTCCTAGGAGCAATAGTGTGAACGTCCA
>SRR4252606_789476___Sbg6
ACGCTCCTAGGAGCAATAGTGTGAACGTCCA
>SRR4252608_12363595___Sbg6
ACGCTCCTAGGAGCAATAGTGTGAACGTCCA
>SRR4252608_6127698___Sbg6
ACGCTCCTAGGAGCAATAGTGTGAACGTCCA
>SRR4252610_10010129___Sbg6
ACGCTCCTAGGAGCAATAGTGTGAACGTCCA
>SRR4252610_11175155___Sbg6
ACGCTCCTAGGAGCAATAGTGTGAACGTCCA
>SRR4252610_2601777___Sbg6
ACGCTCCTAGGAGCAATAGTGTGAACGTCCA
>SRR4252610_2772394___Sbg6
ACGCTCCTAGGAGCAATAGTGTGAACGTCCA
>SRR4252610_5144794___Sbg6
ACGCTCCTAGGAGCAATAGTGTGAACGTCCA
>SRR4252610_7797248___Sbg6
ACGCTCCTAGGAGCAATAGTGTGAACGTCCA
>SRR4252612_7715401___Sbg6
ACGCTCCTAGGAGCAATAGTGTGAACGTCCA
>SRR4252624_4030704___Sbg6
ACGCTCCTAGGAGCAATAGTGTGAACGTCCA

>SRR4252610_10264659___Sbg6
GCGCTCCTAGGAGCAATAGTGTGAACGTCCA
>SRR4252610_1715162___Sbg6
ATTTCGTACCCAAATAGAGTCCACACTA
>SRR4252610_3784342___Sbg6
AAGTCGAGATTCAGCCCATTAGATACA
>SRR4252610_5871986___Sbg6
GATCCTAGGAGCAATAGTGTGAACGTCCA
>SRR4252606_5644101___Sbg6
ATCCTAGGAGCAATAGTGTGAACGTCCA
>SRR4252611_2166059___Sbg6
GAGCTCCTAGGAGCAATAGTGTGAACGTCCA
>SRR4252621_6793837___Sbg6
GAGCTCCTAGGAGCAATAGTGTGAACGTCCA
>SRR4252624_9751700___Sbg6
GAGCTCCTAGGAGCAATAGTGTGAACGTCCA
>SRR4252608_9170176___Sbg6
AGCTCCTAGGAGCAATAGTGTGAACGTCCA
>SRR4252611_9187228___Sbg6
AGCTCCTAGGAGCAATAGTGTGAACGTCCA
>SRR4252623_5179113___Sbg6
AGAGTTCACACTAGCACTCCTAGGAGCAA
>SRR4252610_8599730___Sbg6
ACTCCTAGGAGCAATAGTGTGAACGTCCA
>SRR4252612_4699927___Sbg6
ACTCCTAGGAGCAATAGTGTGAACGTCCA
>SRR4252619_3370033___Sbg6
ACTCCTAGGAGCAATAGTGTGAACGTCCA
>SRR4252610_894072___Sbg6
TGGCATGTAAAAGAACCCTTGGGTACACT
>SRR4252605_675262___Sbg6
GGAGCAATAGTGTGAACGTCCAAATCCG
>SRR4252608_6221109___Sbg6
GCGCTCCTAGGAGCAATAGTGTGAACGTCCA
>SRR4252610_1203840___Sbg6
AAGTCGAGATTCAGCCCATTAGATACA
>SRR4252610_5506605___Sbg6
GCGCTCCTAGGAGCAATAGTGTGAACGTCCA
>SRR4252610_13079659___Sbg6
CTCCTAGGAGCAATAGTGTGAACGTCCA
>SRR4252606_10031144___Sbg6
GCACCTAGGAGCAATAGTGTGAACGTCCA
>SRR4252606_10203325___Sbg6
GCACCTAGGAGCAATAGTGTGAACGTCCA
>SRR4252606_1785424___Sbg6
GCACCTAGGAGCAATAGTGTGAACGTCCA
>SRR4252606_4274156___Sbg6
GCACCTAGGAGCAATAGTGTGAACGTCCA
>SRR4252606_4696417___Sbg6
GCACCTAGGAGCAATAGTGTGAACGTCCA
>SRR4252606_658204___Sbg6
GCACCTAGGAGCAATAGTGTGAACGTCCA
>SRR4252607_14291700___Sbg6
GCACCTAGGAGCAATAGTGTGAACGTCCA
>SRR4252607_2694651___Sbg6
GCACCTAGGAGCAATAGTGTGAACGTCCA
>SRR4252607_353822___Sbg6
GCACCTAGGAGCAATAGTGTGAACGTCCA
>SRR4252607_7003552___Sbg6
GCACCTAGGAGCAATAGTGTGAACGTCCA
>SRR4252608_4927770___Sbg6
GCACCTAGGAGCAATAGTGTGAACGTCCA
>SRR4252608_960619___Sbg6
GCACCTAGGAGCAATAGTGTGAACGTCCA
>SRR4252610_10208227___Sbg6
GCACCTAGGAGCAATAGTGTGAACGTCCA
>SRR4252610_1194238___Sbg6
GCACCTAGGAGCAATAGTGTGAACGTCCA
>SRR4252610_12941921___Sbg6
GCACCTAGGAGCAATAGTGTGAACGTCCA

```
>SRR4252610_4426525___Sbg6
GCACCTAGGAGCAATAGTGTGAACGTCCA
>SRR4252610_4774332___Sbg6
GCACCTAGGAGCAATAGTGTGAACGTCCA
>SRR4252610_5156521___Sbg6
GCACCTAGGAGCAATAGTGTGAACGTCCA
>SRR4252610_5715374___Sbg6
GCACCTAGGAGCAATAGTGTGAACGTCCA
>SRR4252610_8125675___Sbg6
GCACCTAGGAGCAATAGTGTGAACGTCCA
>SRR4252610_8462007___Sbg6
GCACCTAGGAGCAATAGTGTGAACGTCCA
>SRR4252610_9368374___Sbg6
GCACCTAGGAGCAATAGTGTGAACGTCCA
>SRR4252610_9552228___Sbg6
GCACCTAGGAGCAATAGTGTGAACGTCCA
>SRR4252610_9795307___Sbg6
GCACCTAGGAGCAATAGTGTGAACGTCCA
>SRR4252611_13737166___Sbg6
GCACCTAGGAGCAATAGTGTGAACGTCCA
>SRR4252611_14161279___Sbg6
GCACCTAGGAGCAATAGTGTGAACGTCCA
>SRR4252611_4737001___Sbg6
GCACCTAGGAGCAATAGTGTGAACGTCCA
>SRR4252611_8330637___Sbg6
GCACCTAGGAGCAATAGTGTGAACGTCCA
>SRR4252611_9292649___Sbg6
GCACCTAGGAGCAATAGTGTGAACGTCCA
>SRR4252621_2080245___Sbg6
GCACCTAGGAGCAATAGTGTGAACGTCCA
>SRR4252621_6528304___Sbg6
GCACCTAGGAGCAATAGTGTGAACGTCCA
>SRR4252605_11980982___Sbg6
CACCTAGGAGCAATAGTGTGAACGTCCA
>SRR4252605_14663307___Sbg6
CACCTAGGAGCAATAGTGTGAACGTCCAA
>SRR4252605_3500389___Sbg6
CACCTAGGAGCAATAGTGTGAACGTCCA
>SRR4252605_4146655___Sbg6
CACCTAGGAGCAATAGTGTGAACGTCCAA
>SRR4252606_2229589___Sbg6
CACCTAGGAGCAATAGTGTGAACGTCCAA
>SRR4252606_2729223___Sbg6
CACCTAGGAGCAATAGTGTGAACGTCCAA
>SRR4252606_316484___Sbg6
CACCTAGGAGCAATAGTGTGAACGTCCAA
>SRR4252606_3632472___Sbg6
CACCTAGGAGCAATAGTGTGAACGTCCA
>SRR4252606_466135___Sbg6
CACCTAGGAGCAATAGTGTGAACGTCCAAA
>SRR4252606_4825700___Sbg6
CACCTAGGAGCAATAGTGTGAACGTCCA
>SRR4252606_4860488___Sbg6
CACCTAGGAGCAATAGTGTGAACGTCCAA
>SRR4252606_498037___Sbg6
CACCTAGGAGCAATAGTGTGAACGTCCAA
>SRR4252607_10307677___Sbg6
CACCTAGGAGCAATAGTGTGAACGTCCA
>SRR4252607_13019102___Sbg6
CACCTAGGAGCAATAGTGTGAACGTCCAA
>SRR4252607_5495443___Sbg6
CACCTAGGAGCAATAGTGTGAACGTCCAA
>SRR4252608_10065883___Sbg6
CACCTAGGAGCAATAGTGTGAACGTCCAA
>SRR4252608_10798725___Sbg6
CACCTAGGAGCAATAGTGTGAACGTCCA
>SRR4252608_11081991___Sbg6
CACCTAGGAGCAATAGTGTGAACGTCCAA
>SRR4252608_12264590___Sbg6
CACCTAGGAGCAATAGTGTGAACGTCCAA
```

>SRR4252608_1841369___Sbg6
CACCTAGGAGCAATAGTGTGAACGTCCAA
>SRR4252608_2252745___Sbg6
CACCTAGGAGCAATAGTGTGAACGTCCAA
>SRR4252608_2704167___Sbg6
CACCTAGGAGCAATAGTGTGAACGTCCAA
>SRR4252608_4089259___Sbg6
CACCTAGGAGCAATAGTGTGAACGTCCA
>SRR4252608_5122965___Sbg6
CACCTAGGAGCAATAGTGTGAACGTCCA
>SRR4252608_5266137___Sbg6
CACCTAGGAGCAATAGTGTGAACGTCCAA
>SRR4252608_5804381___Sbg6
CACCTAGGAGCAATAGTGTGAACGTCCAA
>SRR4252608_6797667___Sbg6
CACCTAGGAGCAATAGTGTGAACGTCCA
>SRR4252608_8808480___Sbg6
CACCTAGGAGCAATAGTGTGAACGTCCAA
>SRR4252608_8852069___Sbg6
CACCTAGGAGCAATAGTGTGAACGTCCAA
>SRR4252609_13431932___Sbg6
CACCTAGGAGCAATAGTGTGAACGTCCAA
>SRR4252609_7197790___Sbg6
CACCTAGGAGCAATAGTGTGAACGTCCAA
>SRR4252610_10186555___Sbg6
CACCTAGGAGCAATAGTGTGAACGTCCAA
>SRR4252610_1045213___Sbg6
CACCTAGGAGCAATAGTGTGAACGTCCA
>SRR4252610_10672318___Sbg6
CACCTAGGAGCAATAGTGTGAACGTCCAA
>SRR4252610_10765477___Sbg6
CACCTAGGAGCAATAGTGTGAACGTCCA
>SRR4252610_10804465___Sbg6
CACCTAGGAGCAATAGTGTGAACGTCCAA
>SRR4252610_11066922___Sbg6
CACCTAGGAGCAATAGTGTGAACGTCCAA
>SRR4252610_110835___Sbg6
CACCTAGGAGCAATAGTGTGAACGTCCAA
>SRR4252610_1208493___Sbg6
CACCTAGGAGCAATAGTGTGAACGTCCAA
>SRR4252610_1389525___Sbg6
CACCTAGGAGCAATAGTGTGAACGTCCAA
>SRR4252610_1550753___Sbg6
CACCTAGGAGCAATAGTGTGAACGTCCA
>SRR4252610_2528691___Sbg6
CACCTAGGAGCAATAGTGTGAACGTCCA
>SRR4252610_3096575___Sbg6
CACCTAGGAGCAATAGTGTGAACGTCCA
>SRR4252610_3866521___Sbg6
CACCTAGGAGCAATAGTGTGAACGTCCAA
>SRR4252610_425867___Sbg6
CACCTAGGAGCAATAGTGTGAACGTCCA
>SRR4252610_4503864___Sbg6
CACCTAGGAGCAATAGTGTGAACGTCCA
>SRR4252610_5254855___Sbg6
CACCTAGGAGCAATAGTGTGAACGTCCA
>SRR4252610_6108577___Sbg6
CACCTAGGAGCAATAGTGTGAACGTCCA
>SRR4252610_787723___Sbg6
CACCTAGGAGCAATAGTGTGAACGTCCAA
>SRR4252610_9019215___Sbg6
CACCTAGGAGCAATAGTGTGAACGTCCA
>SRR4252610_9449557___Sbg6
CACCTAGGAGCAATAGTGTGAACGTCCAA
>SRR4252610_9945427___Sbg6
CACCTAGGAGCAATAGTGTGAACGTCCAA
>SRR4252611_2664122___Sbg6
CACCTAGGAGCAATAGTGTGAACGTCCA
>SRR4252614_4434605___Sbg6
CACCTAGGAGCAATAGTGTGAACGTCCAA

>SRR4252621_6173798___Sbg6
CACCTAGGAGCAATAGTGTGAACGTCCAA
>SRR4252623_9602094___Sbg6
CACCTAGGAGCAATAGTGTGAACGTCCAA
>SRR4252624_2967534___Sbg6
CACCTAGGAGCAATAGTGTGAACGTCCAA
>SRR4252626_8994352___Sbg6
CACCTAGGAGCAATAGTGTGAACGTCCAA
>SRR4252605_13073646___Sbg6
ACCTAGGAGCAATAGTGTGAACGTCCAA
>SRR4252605_2056107___Sbg6
ACCTAGGAGCAATAGTGTGAACGTCCAAA
>SRR4252605_2982109___Sbg6
ACCTAGGAGCAATAGTGTGAACGTCCAA
>SRR4252605_7578010___Sbg6
ACCTAGGAGCAATAGTGTGAACGTCCAA
>SRR4252605_9014020___Sbg6
ACCTAGGAGCAATAGTGTGAACGTCCAA
>SRR4252605_9032138___Sbg6
ACCTAGGAGCAATAGTGTGAACGTCCAA
>SRR4252606_239300___Sbg6
ACCTAGGAGCAATAGTGTGAACGTCCAA
>SRR4252606_2777105___Sbg6
ACCTAGGAGCAATAGTGTGAACGTCCAA
>SRR4252606_3139857___Sbg6
ACCTAGGAGCAATAGTGTGAACGTCCAA
>SRR4252606_4917494___Sbg6
ACCTAGGAGCAATAGTGTGAACGTCCAA
>SRR4252607_13775347___Sbg6
ACCTAGGAGCAATAGTGTGAACGTCCAA
>SRR4252607_4591346___Sbg6
ACCTAGGAGCAATAGTGTGAACGTCCAAA
>SRR4252608_11571899___Sbg6
ACCTAGGAGCAATAGTGTGAACGTCCAA
>SRR4252608_1230448___Sbg6
ACCTAGGAGCAATAGTGTGAACGTCCAA
>SRR4252608_13163549___Sbg6
ACCTAGGAGCAATAGTGTGAACGTCCAA
>SRR4252608_8163202___Sbg6
ACCTAGGAGCAATAGTGTGAACGTCCAA
>SRR4252608_8473035___Sbg6
ACCTAGGAGCAATAGTGTGAACGTCCAA
>SRR4252609_1011336___Sbg6
ACCTAGGAGCAATAGTGTGAACGTCCAA
>SRR4252610_12865617___Sbg6
ACCTAGGAGCAATAGTGTGAACGTCCAA
>SRR4252610_12872132___Sbg6
ACCTAGGAGCAATAGTGTGAACGTCCAA
>SRR4252610_12913___Sbg6
ACCTAGGAGCAATAGTGTGAACGTCCAA
>SRR4252610_1468923___Sbg6
ACCTAGGAGCAATAGTGTGAACGTCCAA
>SRR4252610_1703923___Sbg6
ACCTAGGAGCAATAGTGTGAACGTCCAA
>SRR4252610_3220864___Sbg6
ACCTAGGAGCAATAGTGTGAACGTCCAA
>SRR4252610_6091443___Sbg6
ACCTAGGAGCAATAGTGTGAACGTCCAA
>SRR4252610_6607838___Sbg6
ACCTAGGAGCAATAGTGTGAACGTCCAA
>SRR4252610_8346322___Sbg6
ACCTAGGAGCAATAGTGTGAACGTCCAAA
>SRR4252610_842028___Sbg6
ACCTAGGAGCAATAGTGTGAACGTCCAA
>SRR4252611_1114852___Sbg6
ACCTAGGAGCAATAGTGTGAACGTCCAA
>SRR4252611_4297276___Sbg6
ACCTAGGAGCAATAGTGTGAACGTCCAAA
>SRR4252611_4436226___Sbg6
ACCTAGGAGCAATAGTGTGAACGTCCAA

>SRR4252612_8288900___Sbg6
ACCTAGGAGCAATAGTGTGAACGTCCAA
>SRR4252614_3966081___Sbg6
ACCTAGGAGCAATAGTGTGAACGTCCAA
>SRR4252621_8487657___Sbg6
ACCTAGGAGCAATAGTGTGAACGTCCAA
>SRR4252622_2464295___Sbg6
ACCTAGGAGCAATAGTGTGAACGTCCAA
>SRR4252623_14932092___Sbg6
ACCTAGGAGCAATAGTGTGAACGTCCAA
>SRR4252623_7173159___Sbg6
ACCTAGGAGCAATAGTGTGAACGTCCAA
>SRR4252624_3120097___Sbg6
ACCTAGGAGCAATAGTGTGAACGTCCAA
>SRR4252624_1225893___Sbg6
GCGCTCCTAGGAGCAATAGTGTGAACGTCCA
>SRR4252609_1962767___Sbg6
CCTAGGAGCAATAGTGTGAACGTCCAA
>SRR4252610_12751683___Sbg6
GCGCTCCTAGGAGCAATAGTGTGAACGTCCA
>SRR4252607_7860364___Sbg6
ATTAAATCCTGGCCAAGTTTCGTACCCA
>SRR4252614_1001387___Sbg6
CTCCTAGGAGCAATAGTGTGAACGTCCAA
>SRR4252611_181797___Sbg6
TCCTAGGAGCTATAGTGTGAACGTCCA
>SRR4252610_12624377___Sbg6
GTCATCGGTTAGATGGCATGTAAAAG
>SRR4252605_14299351___Sbg6
GCGCTCCTAGGAGCAATAGTGTGAACGTCCA
>SRR4252622_8006672___Sbg6
CGGCCGGGTCTGACAACGACGTAGTA
>SRR4252607_5077574___Sbg6
TCCTAGAAGCAATAGTGTGAACGTCCAA
>SRR4252606_7921046___Sbg6
GCCGGATTTGGACGTTCACACTATTGCTCC
>SRR4252605_4939234___Sbg6
TTGGACGTTCACACTATTGCTCCTAGG
>SRR4252605_7198124___Sbg6
TGGACGTTCACACTATTGCTCCTAGGAGCGC
>SRR4252625_6565968___Sbg6
CAGGGATGAGAGTTTCTCCGAGTTGGCA
>SRR4252606_2771994___Sbg6
TGGACGTTCACACTATTGCTCCTAGGAGCGC
>SRR4252610_9791470___Sbg6
TGGACGTTCACACTATTGCTCCTAGGAGCGC
>SRR4252611_11987854___Sbg6
GTACCCAAGGGTTCTTTTACATGCCATCTA
>SRR4252606_5371229___Sbg6
TGTATCTAATGGGCTGAATCTCGACTT
>SRR4252608_9541763___Sbg6
TGGACGTTCACACTATTGCTCCTAGGAGCGC
>SRR4252605_6962088___Sbg6
ACCCAAGTGTTCTTTTACATGCCATCTA
>SRR4252605_8856035___Sbg6
ACCCAAGTGTTCTTTTACATGCCATCTAA
>SRR4252610_11455137___Sbg6
ACCCAAGTGTTCTTTTACATGCCATCTA
>SRR4252612_2538272___Sbg6
ACCCAAGTGTTCTTTTACATGCCATCTA
>SRR4252612_518717___Sbg6
CCCAAGTGTTCTTTTACATGCCATCTAA
>SRR4252607_4099553___Sbg6
TGGGTACGAAACTTGGCCGGGATTTATT
>SRR4252609_13701687___Sbg6
TGGACGTTCACACTATTGCTCCTAGGAGCGC
>SRR4252609_8785509___Sbg6
TTGGACGTTCACACTATTGCTCCTAGG
>SRR4252610_2976877___Sbg6
GACTTACTACGTCGTTGTCAGACCCGG

>SRR4252605_902176___Sbg6
TGCGTGCCATGGATTTTTTGTACCCTAA
>SRR4252606_4767604___Sbg6
TTGGACGTTCACACTATTGCTCCTAGG
>SRR4252612_67786___Sbg6
TGGACGTTCACACTATTGCTCCTAGGAGCG
>SRR4252620_3541502___Sbg6
CGAAACTTTGCCAGGATTTAATTTTGCCGA
>SRR4252611_330707___Sbg6
TTGGACGTTCACACTATTGCTCCTAGG
>SRR4252610_4346788___Sbg6
TTGGACGTTCACACTATTGCTCCTAGGAG
>SRR4252610_4411256___Sbg6
TGGACGTTCACACTATTGCTCCTAGGAGCA
>SRR4252605_479704___Sbg6
CTCTATTTGGGTACGAAACTTGGCCAGG
>SRR4252607_10894970___Sbg6
TTGGACGTTCACACTATTGCTTCTAGG
>SRR4252619_11629303___Sbg6
TTGGACGTTCACACTATTGCTTCTAGGA
>SRR4252610_5386437___Sbg6
TGTATCTAATGGGCTGAATCTCGACTT
>SRR4252606_415944___Sbg6
TGGACGTTCACACTATTGCTCCTAGGAGT
>SRR4252623_7290042___Sbg6
GACTCTATTTGGGTACGAAACTTGGC
>SRR4252609_8114792___Sbg6
TTGGACGTTCACACTATTGCTCCTAGG
>SRR4252606_2888104___Sbg6
TGGACGTTCACACTATTGCTCCTAGGAGCGT
>SRR4252606_5286719___Sbg6
TGGACGTTCACACTATTGCTCCTAGGAGCGT
>SRR4252606_6739667___Sbg6
TGGACGTTCACACTATTGCTCCTAGGAGCGT
>SRR4252608_11490149___Sbg6
TGGACGTTCACACTATTGCTCCTAGGAGCGT
>SRR4252610_12798147___Sbg6
TGGACGTTCACACTATTGCTCCTAGGAGCGT
>SRR4252610_13245702___Sbg6
TGGACGTTCACACTATTGCTCCTAGGAGCGT
>SRR4252610_2184654___Sbg6
TGGACGTTCACACTATTGCTCCTAGGAGCGT
>SRR4252610_2213842___Sbg6
TGGACGTTCACACTATTGCTCCTAGGAGCGT
>SRR4252610_3997354___Sbg6
TGGACGTTCACACTATTGCTCCTAGGAGCGT
>SRR4252610_4110471___Sbg6
TGGACGTTCACACTATTGCTCCTAGGAGCGT
>SRR4252610_5281123___Sbg6
TGGACGTTCACACTATTGCTCCTAGGAGCGT
>SRR4252610_6004301___Sbg6
TGGACGTTCACACTATTGCTCCTAGGAGCGT
>SRR4252610_6751235___Sbg6
TGGACGTTCACACTATTGCTCCTAGGAGCGT
>SRR4252611_8383867___Sbg6
TGGACGTTCACACTATTGCTCCTAGGAGCGT
>SRR4252623_13864853___Sbg6
TGGACGTTCACACTATTGCTCCTAGGAGCGT
>SRR4252610_87322___Sbg6
TGGACGTTCACACTATTGCTCCTAGGAGCGC
>SRR4252606_9959245___Sbg6
TGGACGTTCACACTATTGCTCCTAGGAGC
>SRR4252612_1986118___Sbg6
TTGGACGTTCACACTATTGCTCCTAGGAG
>SRR4252609_11698707___Sbg6
TGGACGTTCACACTATTGCTCCTAGGAGCTC
>SRR4252614_1033735___Sbg6
TTGGACGTTCACACTATTGCTCCTAGGAG
>SRR4252605_8516293___Sbg6
TTGGGTACGAAACTTGGCCAGGATTTAATT

>SRR4252619_8967242___Sbg6
TTGGACGTTCACACTATTGCTCCTAGGAGC
>SRR4252611_15129170___Sbg6
TGGGTACGAAACTTGGCCGGGATTTAATT
>SRR4252608_2972485___Sbg6
TGTATCTAATGGGCTGAATCTCGACTT
>SRR4252608_7331778___Sbg6
TGGACGTTCACACTATTGCTCCTAGGAG
>SRR4252605_5800331___Sbg6
TGTATCTAATGGGCTGAATCTCGACTT
>SRR4252610_902365___Sbg6
TTGGACGTTCACACTATTGCTCCTAGG
>SRR4252606_2978403___Sbg6
TGGACGTTCACACTATTGCTCCTAGGAA
>SRR4252608_12146314___Sbg6
TGGACGTTCACACTATTGCTCCTAGGAGCGC
>SRR4252611_11920717___Sbg6
TTGGACGTTCACACTATTGCTCCTAGG
>SRR4252616_3916443___Sbg6
ACCCAAGTGTTCTTTTACATGCCATCTA
>SRR4252611_69703___Sbg6
TTGGACGTTCACACTATTGCTCCTAGGA
>SRR4252615_6367973___Sbg6
CGCTAGTGTGGACTCTATTTGGGTACGAA
>SRR4252607_8088505___Sbg6
AGTACCCAAGGGTTCTTTTACATGCCATCTA
>SRR4252609_12546041___Sbg6
TGGGTACGAAACTTGGCCGGGATTTAATT
>SRR4252610_10939___Sbg6
ACCCAAGGGTTCTTTTACATGCCATCTA
>SRR4252622_966865___Sbg6
TGGACGTTCACACTATTGCTCCTAGGAGCGC
>SRR4252615_6296448___Sbg6
TCAGGATTTAATTTTGCCGAGGGTACGAA
>SRR4252611_4804164___Sbg6
TTGGACGTTCACACTATTGCTCCTAGG
>SRR4252605_9787157___Sbg6
TGGACGTTCACACTATTGCTCCTAGGAGCGC
>SRR4252610_7890616___Sbg6
TTGGACGTTCACACTATTGCTCCTAGGAG
>SRR4252620_44517___Sbg6
GCGTGCCATGAATTTTTTGTACCCTAAAA
>SRR4252610_7724177___Sbg6
TGGACGTTCACACTATTGCTCCTAGGAGCGC
>SRR4252610_12801191___Sbg6
TGGACGTTCACACTATTGCTCCTAGGAGCGC
>SRR4252610_2782647___Sbg6
TGGACGTTCACACTATTGCTCCTAGGAGCGC
>SRR4252610_5860295___Sbg6
TGGACGTTCACACTATTGCTCCTAGGAGCGC
>SRR4252610_8685999___Sbg6
TGGACGTTCACACTATTGCTCCTAGGAGCGC
>SRR4252615_5020633___Sbg6
TGTGGACTCTATTTGGGTACGAAACTTGG
>SRR4252609_13895943___Sbg6
CCCAAGGGTTCTTTTACATGCCATCTAA
>SRR4252612_6908562___Sbg6
TGGACGTTCACACTATTGCTCCTAGGAGCGC
>SRR4252621_8514549___Sbg6
TGGACGTTCACACTATTGCTCCTAGGAGCGC
>SRR4252625_5828669___Sbg6
TGGACGTTCACACTATTGCTCCTAGGAGCGC
>SRR4252609_11649447___Sbg6
TGGACGTTCACACTATTGCTCCTAGGAGCGC
>SRR4252605_12338155___Sbg6
TTGGACGTTCACACTATTGCTCCTAGG
>SRR4252605_7448881___Sbg6
TGACATGCGTGCTATGGATTTTTGTACC
>SRR4252606_6609451___Sbg6
TGACATGCGTGCTATGGATTTTTGTACCC

>SRR4252608_13035850___Sbg6
TGACATGCGTGCTATGGATTTTTGTACC
>SRR4252609_2602927___Sbg6
TGACATGCGTGCTATGGATTTTTGTACCC
>SRR4252611_771965___Sbg6
TGACATGCGTGCTATGGATTTTTGTACCCT
>SRR4252612_2688175___Sbg6
TGACATGCGTGCTATGGATTTTTGTACCC
>SRR4252612_3006587___Sbg6
TGACATGCGTGCTATGGATTTTTGTACCC
>SRR4252612_4553497___Sbg6
TGACATGCGTGCTATGGATTTTTGTACC
>SRR4252613_93330___Sbg6
TGACATGCGTGCTATGGATTTTTGTACCC
>SRR4252614_1006836___Sbg6
TGACATGCGTGCTATGGATTTTTGTACC
>SRR4252615_14527103___Sbg6
TGACATGCGTGCTATGGATTTTTGTACCC
>SRR4252616_8030516___Sbg6
TGACATGCGTGCTATGGATTTTTGTACC
>SRR4252621_4349610___Sbg6
TGACATGCGTGCTATGGATTTTTGTACCC
>SRR4252618_5732820___Sbg6
GACATGCGTGCTATGGATTTTTGTACCC
>SRR4252609_15202492___Sbg6
CCGGATTTGGACGTTCACACTATTGCTC
>SRR4252606_6242265___Sbg6
ACTCTCGCACCGGATTTGGACGTTCACA
>SRR4252606_9044914___Sbg6
TGGACGTTCACACTATTGCTCCTAGGAGCGC
>SRR4252610_3629253___Sbg6
TGGACGTTCACACTATTGCTCCTAGGAGCGC
>SRR4252610_4791346___Sbg6
TGGACGTTCACACTATTGCTCCTAGG
>SRR4252608_9340811___Sbg6
TGGACGTTCACACTATTGCTCCTAGGAG
>SRR4252609_11278441___Sbg6
TGGACGTTCACACTATTGCTCCTAGGAGC
>SRR4252619_1532199___Sbg6
TGGACGTTCACACTATTGCTCCTAGGAGCGC
>SRR4252607_14549793___Sbg6
TACGAAACTTGGCCAGGATTTAATTTTGC
>SRR4252611_232054___Sbg6
TTGGACGTTCACACTATTGCTCCGAGG
>SRR4252623_1892477___Sbg6
CAGGATTTAATTTTGCCGAGGGTACGAA
>SRR4252612_8542691___Sbg6
GTACCCAAGGGTTCTTTTACATGCCA
>SRR4252619_5608278___Sbg6
TGGACGTTCACACTATTGCTCCTAGGAGCGC
>SRR4252611_13724759___Sbg6
TGCGTGCCATGGATTTTTTGTACCCTA
>SRR4252612_6291907___Sbg6
CCGACCCCAGGGATGAGAGTTTCTCCGAG
>SRR4252605_9846881___Sbg6
TGCGTGCCATGGATTTTTTGTACCCTAA
>SRR4252619_5701175___Sbg6
TGGACGTTCACACTATAGCTCCTAGGAGC
>SRR4252605_13095892___Sbg6
TTGGACGTTCACACTATTGCTCCTAGG
>SRR4252611_8393961___Sbg6
TTGGACGTTCACACTATTGCTCCTAGG
>SRR4252606_1692385___Sbg6
TTGGACGTTCACACTATTGCTCCTAGGAG
>SRR4252610_6705975___Sbg6
TGGACGTTCACACTATTGCTCCTAGGAGCGC
>SRR4252606_7167701___Sbg6
TGGACGTTCACACTATTGCTCCTAGGAGCGC
>SRR4252610_4581634___Sbg6
TGGACGTTCACACTATTGCTCCTAGGAGCGC

>SRR4252610_2118864___Sbg6
GTGGACTCTATTTGGGTACGAAACTTGGCT
>SRR4252610_5139650___Sbg6
TTGGACGTTCACACTATTGCTCCTAGG
>SRR4252619_9695841___Sbg6
TGGACGTTCACACTATTGCTCCTAGGAGCGC
>SRR4252610_6437034___Sbg6
GCGTTAGTGTGGACTCTATTTGGGTACGAA
>SRR4252610_7862246___Sbg6
TGGACGTTCACACTATTGCTCCTAGGAGCGC
>SRR4252611_7177997___Sbg6
TCTATTTGGGTACGAAACTTGACCAGGAT
>SRR4252611_6246970___Sbg6
TCTATTTGGGTACGAAACTTGACCAGGAT
>SRR4252610_3890449___Sbg6
TGGACGTTCACACTATTGCTCCTAGGAGCGC
>SRR4252624_4314240___Sbg6
TACGAAACTTGGCCAGGATTTAATTTTG
>SRR4252610_13175302___Sbg6
TGGACGTTCACACTATTGCTCCTAGGAGCGC
>SRR4252612_7442819___Sbg6
TCTCGACTTACTACGTCGTTGTCAGACCC
>SRR4252608_6651932___Sbg6
TGGACGTTCACACTATTGCTCCTAGGAGCGC
>SRR4252608_3483758___Sbg6
TGTGGACTCTATTTGGGTACGAAACTTGGC
>SRR4252606_9995522___Sbg6
TGGACGTTCACACTATTGCTCCTAGGAGC
>SRR4252610_12091988___Sbg6
TGGACGTTCACACTATTGCTCCTAGGAGCGC
>SRR4252610_50506___Sbg6
TGGGTACGAAACTTGGCCGGGATTTAATT
>SRR4252617_12905739___Sbg6
CTCGCGCCGGATTTGGACGTTCACACTAT
>SRR4252610_10030184___Sbg6
TTGGACGTTCACACTATTGCTCCTAGGAG
>SRR4252611_971758___Sbg6
CGACCCCAGGGATGAGAGTTTCTCCGAGTTG
>SRR4252625_1778876___Sbg6
TTGGACGTTCACACTATTGCTCCTAGGAG
>SRR4252619_10765657___Sbg6
GGACGTTCACACTATTGCTCCTAGGAGCGC
>SRR4252606_3622578___Sbg6
TAGTGGCGAACTCTCGCGCCGGATTTGG
>SRR4252611_11456449___Sbg6
TTGGACGTTCACACTATTGCTCCTAGG
>SRR4252607_4371751___Sbg6
AAGGGTTCTTTTACATGCCATCTAATCG
>SRR4252606_7035138___Sbg6
TCGACCCCAGGGATGAGAGTTTCTCCGA
>SRR4252607_12089266___Sbg6
ACGTGCCATGGATTTTTTGTACCCTAAAA
>SRR4252617_9615067___Sbg6
TTTGGACGTTCACACTATTGCTCCTAGG
>SRR4252609_14437920___Sbg6
TGGACGTTCACACTATTGCTCCTAGG
>SRR4252608_1398713___Sbg6
TAGTGTGGACTCTATTTGGGTACGAAA
>SRR4252619_3827895___Sbg6
TTGGACGTTCACACTATTGCTCCTAGGAGT
>SRR4252618_9823409___Sbg6
TTTGGACGTTCACACTATTGCTCCTAGG
>SRR4252623_4468277___Sbg6
TTTGGACGTTCACACTATTGCTCCTAGGA
>SRR4252608_8946181___Sbg6
TGTATCTAATGGGCTGAATCTCGACTT
>SRR4252608_5284827___Sbg6
TTGGACGTTCACACTATTGCTCCTAGG
>SRR4252608_1164442___Sbg6
TACGAAACTTGGCCAGGATTTAATTTTG

>SRR4252606_2402007___Sbg6
TGGACGTTCACACTATTGCTCCTAGGAGCGC
>SRR4252608_11469652___Sbg6
TGCGTGCCATGGATTTTTTGTACCCTAA
>SRR4252606_10418406___Sbg6
TTGGACGTTCACACTATTGCTCCTAGGAG
>SRR4252607_13572532___Sbg6
CCCAAGGGTTCTTTTACATGCCATCTA
>SRR4252624_892986___Sbg6
TTGGACGTTCACACTATTGCTTCTAGGA
>SRR4252610_7453665___Sbg6
TGGACGTTCACACTATTGCTCCTAGGAG
>SRR4252611_3576731___Sbg6
GATTTGGACGTTCACACTATTACTCCT
>SRR4252610_3779710___Sbg6
TAGTGTGGACTCTATTTGTGTACGAAAC
>SRR4252610_8961235___Sbg6
TGGACGTTCACACTATTGCTCCTAGGAGCGC
>SRR4252605_10613926___Sbg6
TTGGACGTTCACACTATTGCCCCTAGG
>SRR4252606_2040829___Sbg6
TTGGACGTTCACACTATTGCTCCTAGGA
>SRR4252608_2810253___Sbg6
TGGACGTTCACACTATTGCTCCTAGGAGCGC
>SRR4252608_4580051___Sbg6
TGGACGTTCACACTATTGCTCCTAGGA
>SRR4252617_3791445___Sbg6
TGTATCTAATGGGCTGAATCTCGACT
>SRR4252609_490858___Sbg6
TTGGACGTTCACACTATTGCTCCTAGGA
>SRR4252612_2852332___Sbg6
TTGGACGTTCACACTATTGCTCCTAGGA
>SRR4252608_256962___Sbg6
TGGACGTTCACACTATTGCTCCTAGGAGT
>SRR4252610_7620325___Sbg6
TGGACGTTCACACTATTGCTCCTAGGAG
>SRR4252605_12242971___Sbg6
GGGGTACGAAGTGTACCCAAGGGTTCTTT
>SRR4252605_8827714___Sbg6
CCAGGGATGAGAGTTTCTCCGAGTTGGCA
>SRR4252615_2505151___Sbg6
TCCTAGGAGCACTAGTGTGGACTCTA
>SRR4252611_8454516___Sbg6
TTTGGACGTTCACACTATTGCTCCTAGGA
>SRR4252610_12896034___Sbg6
TGGACGTTCACACTATTGCTCCTAGGAGCGC
>SRR4252606_9955025___Sbg6
CGAACTCTCGCGCCGGATTTGGACGTG
>SRR4252605_302484___Sbg6
TGGACTCTATTTGGGTACGGAACTTGGC
>SRR4252609_7499400___Sbg6
TTGGACGTTCACACTATTGCTGCTAGG
>SRR4252610_3825708___Sbg6
TTGGACGTTCACACTATTGCTGCTAGG
>SRR4252610_13118729___Sbg6
TGGACGTTCACACTATTGCTGCTAGGA
>SRR4252612_8132803___Sbg6
TGGACGTTCACACTATTGCTGCTAGGAGC
>SRR4252607_6979128___Sbg6
TGGACGTTCACACTATTGCTCCTAGGAGCC
>SRR4252607_8280776___Sbg6
TGGACGTTCACACTATTGCTCCTAGGAGCGC
>SRR4252605_1168514___Sbg6
TGGACGTTCACACTATTGCTCCTAGGAGCGC
>SRR4252610_2364003___Sbg6
TGGACGTTCACACTATTGCTCCTAGGAGCGC
>SRR4252609_6741715___Sbg6
AAGGGTTCTTTTACATGCCATCTAATCG
>SRR4252605_11732040___Sbg6
TTGGACGTTCACACTATTGCTCTTAGG

>SRR4252605_8409406___Sbg6
TTGGACGTTCACACTATTGCTCTTAGGA
>SRR4252610_10699687___Sbg6
TGTATCTAATGGGCTGAATCTCGACTT
>SRR4252625_2650665___Sbg6
TTGGACGTTCACACTATTGCTCCTAGGAG
>SRR4252621_8043819___Sbg6
TACCCAAGGGTTCTTTTACATGCCATCTA
>SRR4252607_10142550___Sbg6
TTGGACGTTCACACTATTGCTTCTAGGA
>SRR4252608_7099267___Sbg6
TGGACGTTCACACTATTGCTTCTAGGAGC
>SRR4252622_2359096___Sbg6
AGGGTTCTTTTACATGCCATCTAACCGA
>SRR4252606_7472597___Sbg6
TTTGGACGTTCACACTATTACTCCTAGGA
>SRR4252610_117012___Sbg6
AGTGTACCCAAGGGTTCTTTTACATGCCA
>SRR4252610_13064985___Sbg6
CCGGATTTGGACGTTCACACTATTGCTCT
>SRR4252611_5476811___Sbg6
TAAGGGTTCTTTTACATGCCATCTAA
>SRR4252610_8660476___Sbg6
CCCAAGGGTTCTTTTACATGCCATCTAA
>SRR4252606_5698305___Sbg6
TGGACGTTCACACTATTGCTCCTAGGAGCGC
>SRR4252607_6395715___Sbg6
TCAAGGGTTCTTTTACATGCCATCTAA
>SRR4252618_4451100___Sbg6
GCCCAAGGGTTCTTTTACATGCCATC
>SRR4252610_5597430___Sbg6
TGGACGTTCACACTATTGCTCCTAGGAGCGC
>SRR4252622_3300977___Sbg6
AGGTTCTTTTACATGCCATCTAACCGATGA
>SRR4252623_3467349___Sbg6
TGGACGTTCACACTATTGCTCCTAGGAGCGC
>SRR4252607_11010765___Sbg6
GTACCCAAGGGTTCTTTTACATGCCATCTA
>SRR4252622_10956362___Sbg6
ACCCAAGGGTTCTTTTACATGCCATCTA
>SRR4252619_2393081___Sbg6
TCTAATCGATGACATGCGTGCCATGGAT
>SRR4252611_7424495___Sbg6
TGGACGTTCACACTATTGCTCCTAGGAGCGC
>SRR4252609_10659184___Sbg6
GACCCCAGGGATGAGAGTTTCTCCGAGTTGG
>SRR4252612_2830048___Sbg6
CCGACCCCAGGGATGAGAGTTTCTCCGA
>SRR4252612_8715151___Sbg6
ACCGACCCCAGGGATGAGAGTTTCTCCGA
>SRR4252610_9676982___Sbg6
AATTGGACGTTCACACTATTGCTCCTA
>SRR4252606_7237606___Sbg6
TTGGACGTTCACACTATTGCTCCTAGGAG
>SRR4252609_7775103___Sbg6
TGGGTACGAAACTTGGCCGGGATTTAA
>SRR4252610_11176531___Sbg6
TGGACGTTCACACTATTGCTCCTAGGAG
>SRR4252611_5661123___Sbg6
CAGGGATGAGAGTTTCTCCGAGTTGGCA
>SRR4252610_12960543___Sbg6
TTGGACGTTCACACTATAGCTCCTAGGA
>SRR4252612_1444793___Sbg6
TTGGACGTTCACACTATAGCTCCTAGG
>SRR4252614_3160181___Sbg6
TTGGACGTTCACACTATAGCTCCTAGG
>SRR4252618_5317570___Sbg6
TTGGACGTTCACACTATAGCTCCTAGG
>SRR4252608_3508496___Sbg6
TGGACGTTCACACTATAGCTCCTAGGAGCGC

>SRR4252612_6473890___Sbg6
TGGACGTTCACACTATAGCTCCTAGGAGC
>SRR4252619_9822408___Sbg6
TGGACGTTCACACTATAGCTCCTAGGAGCGC
>SRR4252605_4280368___Sbg6
TTGGACGTTCACACTATTGCTTCTAGG
>SRR4252609_1419295___Sbg6
TTGGACGTTCACACTATTGCTTCTAGG
>SRR4252623_10987021___Sbg6
TTGGACGTTCACACTATTGCTTCTAGGA
>SRR4252610_2297497___Sbg6
TGGACGTTCACACTATTGCTCCTAGGAGCGC
>SRR4252623_3900491___Sbg6
TGGACGTTCACACTATTGCTCCTAGGAGCGC
>SRR4252605_12016716___Sbg6
TGTATCTAATGGGCTGAATCTCGACTT
>SRR4252625_11786826___Sbg6
TACCCAAGGGTTCTTTTACATGCCATCTAA
>SRR4252606_7932730___Sbg6
TTTGGACGTTCACACTATTGCTCCTAGGT
>SRR4252606_9859481___Sbg6
TTTGGACGTTCACACTATTGCTCCTAGGTG
>SRR4252610_13564297___Sbg6
TTTGGACGTTCACACTATTGCTCCTAGGT
>SRR4252621_3417751___Sbg6
TTTGGACGTTCACACTATTGCTCCTAGGT
>SRR4252621_8461617___Sbg6
TTTGGACGTTCACACTATTGCTCCTAGGT
>SRR4252605_1676527___Sbg6
TTGGACGTTCACACTATTGCTCCTAGGT
>SRR4252605_1994849___Sbg6
TTGGACGTTCACACTATTGCTCCTAGGTG
>SRR4252605_4707917___Sbg6
TTGGACGTTCACACTATTGCTCCTAGGT
>SRR4252605_5023668___Sbg6
TTGGACGTTCACACTATTGCTCCTAGGT
>SRR4252605_779362___Sbg6
TTGGACGTTCACACTATTGCTCCTAGGT
>SRR4252605_8643221___Sbg6
TTGGACGTTCACACTATTGCTCCTAGGT
>SRR4252606_10380907___Sbg6
TTGGACGTTCACACTATTGCTCCTAGGT
>SRR4252606_1846727___Sbg6
TTGGACGTTCACACTATTGCTCCTAGGT
>SRR4252606_1865325___Sbg6
TTGGACGTTCACACTATTGCTCCTAGGTG
>SRR4252606_2424824___Sbg6
TTGGACGTTCACACTATTGCTCCTAGGTG
>SRR4252606_2899655___Sbg6
TTGGACGTTCACACTATTGCTCCTAGGT
>SRR4252606_4692368___Sbg6
TTGGACGTTCACACTATTGCTCCTAGGT
>SRR4252606_4969107___Sbg6
TTGGACGTTCACACTATTGCTCCTAGGT
>SRR4252606_6088190___Sbg6
TTGGACGTTCACACTATTGCTCCTAGGTG
>SRR4252606_8263522___Sbg6
TTGGACGTTCACACTATTGCTCCTAGGT
>SRR4252606_8294882___Sbg6
TTGGACGTTCACACTATTGCTCCTAGGT
>SRR4252606_8412870___Sbg6
TTGGACGTTCACACTATTGCTCCTAGGT
>SRR4252606_8507236___Sbg6
TTGGACGTTCACACTATTGCTCCTAGGTG
>SRR4252606_8730119___Sbg6
TTGGACGTTCACACTATTGCTCCTAGGTG
>SRR4252606_9657599___Sbg6
TTGGACGTTCACACTATTGCTCCTAGGTG
>SRR4252607_14730693___Sbg6
TTGGACGTTCACACTATTGCTCCTAGGTG

>SRR4252607_3113927___Sbg6
TTGGACGTTCACACTATTGCTCCTAGGTG
>SRR4252607_5551854___Sbg6
TTGGACGTTCACACTATTGCTCCTAGGT
>SRR4252608_11058534___Sbg6
TTGGACGTTCACACTATTGCTCCTAGGTG
>SRR4252608_11122691___Sbg6
TTGGACGTTCACACTATTGCTCCTAGGTG
>SRR4252608_11976302___Sbg6
TTGGACGTTCACACTATTGCTCCTAGGTG
>SRR4252608_1593348___Sbg6
TTGGACGTTCACACTATTGCTCCTAGGT
>SRR4252608_2207896___Sbg6
TTGGACGTTCACACTATTGCTCCTAGGTG
>SRR4252608_3738797___Sbg6
TTGGACGTTCACACTATTGCTCCTAGGTG
>SRR4252608_4173984___Sbg6
TTGGACGTTCACACTATTGCTCCTAGGT
>SRR4252608_4973590___Sbg6
TTGGACGTTCACACTATTGCTCCTAGGTG
>SRR4252608_6277257___Sbg6
TTGGACGTTCACACTATTGCTCCTAGGT
>SRR4252608_835151___Sbg6
TTGGACGTTCACACTATTGCTCCTAGGT
>SRR4252608_9052666___Sbg6
TTGGACGTTCACACTATTGCTCCTAGGTG
>SRR4252608_9399369___Sbg6
TTGGACGTTCACACTATTGCTCCTAGGT
>SRR4252609_10647761___Sbg6
TTGGACGTTCACACTATTGCTCCTAGGT
>SRR4252609_15843873___Sbg6
TTGGACGTTCACACTATTGCTCCTAGGTG
>SRR4252609_1943301___Sbg6
TTGGACGTTCACACTATTGCTCCTAGGT
>SRR4252609_6117093___Sbg6
TTGGACGTTCACACTATTGCTCCTAGGTG
>SRR4252610_10670115___Sbg6
TTGGACGTTCACACTATTGCTCCTAGGTG
>SRR4252610_11071890___Sbg6
TTGGACGTTCACACTATTGCTCCTAGGT
>SRR4252610_11842263___Sbg6
TTGGACGTTCACACTATTGCTCCTAGGTG
>SRR4252610_12465098___Sbg6
TTGGACGTTCACACTATTGCTCCTAGGTG
>SRR4252610_1512964___Sbg6
TTGGACGTTCACACTATTGCTCCTAGGT
>SRR4252610_2495712___Sbg6
TTGGACGTTCACACTATTGCTCCTAGGTG
>SRR4252610_249653___Sbg6
TTGGACGTTCACACTATTGCTCCTAGGTG
>SRR4252610_2919473___Sbg6
TTGGACGTTCACACTATTGCTCCTAGGT
>SRR4252610_3148168___Sbg6
TTGGACGTTCACACTATTGCTCCTAGGT
>SRR4252610_4261077___Sbg6
TTGGACGTTCACACTATTGCTCCTAGGTG
>SRR4252610_5644615___Sbg6
TTGGACGTTCACACTATTGCTCCTAGGTG
>SRR4252610_6025985___Sbg6
TTGGACGTTCACACTATTGCTCCTAGGT
>SRR4252610_6369546___Sbg6
TTGGACGTTCACACTATTGCTCCTAGGTG
>SRR4252610_6688916___Sbg6
TTGGACGTTCACACTATTGCTCCTAGGT
>SRR4252610_6698656___Sbg6
TTGGACGTTCACACTATTGCTCCTAGGTG
>SRR4252610_8159402___Sbg6
TTGGACGTTCACACTATTGCTCCTAGGT
>SRR4252610_8941561___Sbg6
TTGGACGTTCACACTATTGCTCCTAGGT

>SRR4252610_9007338___Sbg6
TTGGACGTTCACACTATTGCTCCTAGGTG
>SRR4252611_10044309___Sbg6
TTGGACGTTCACACTATTGCTCCTAGGT
>SRR4252611_10866494___Sbg6
TTGGACGTTCACACTATTGCTCCTAGGT
>SRR4252611_1975766___Sbg6
TTGGACGTTCACACTATTGCTCCTAGGT
>SRR4252611_6096238___Sbg6
TTGGACGTTCACACTATTGCTCCTAGGT
>SRR4252611_7540179___Sbg6
TTGGACGTTCACACTATTGCTCCTAGGTG
>SRR4252611_7662129___Sbg6
TTGGACGTTCACACTATTGCTCCTAGGT
>SRR4252612_1817784___Sbg6
TTGGACGTTCACACTATTGCTCCTAGGTG
>SRR4252613_3275556___Sbg6
TTGGACGTTCACACTATTGCTCCTAGGT
>SRR4252619_11072069___Sbg6
TTGGACGTTCACACTATTGCTCCTAGGT
>SRR4252621_1740057___Sbg6
TTGGACGTTCACACTATTGCTCCTAGGTG
>SRR4252621_1868985___Sbg6
TTGGACGTTCACACTATTGCTCCTAGGT
>SRR4252621_2409266___Sbg6
TTGGACGTTCACACTATTGCTCCTAGGTG
>SRR4252621_6224226___Sbg6
TTGGACGTTCACACTATTGCTCCTAGGTG
>SRR4252623_11461005___Sbg6
TTGGACGTTCACACTATTGCTCCTAGGT
>SRR4252623_476961___Sbg6
TTGGACGTTCACACTATTGCTCCTAGGT
>SRR4252623_4819741___Sbg6
TTGGACGTTCACACTATTGCTCCTAGGTG
>SRR4252624_8151686___Sbg6
TTGGACGTTCACACTATTGCTCCTAGGT
>SRR4252605_10390075___Sbg6
TGGACGTTCACACTATTGCTCCTAGGTGC
>SRR4252605_14922291___Sbg6
TGGACGTTCACACTATTGCTCCTAGGTGC
>SRR4252605_1717111___Sbg6
TGGACGTTCACACTATTGCTCCTAGGTGC
>SRR4252605_2148954___Sbg6
TGGACGTTCACACTATTGCTCCTAGGTGC
>SRR4252605_5212659___Sbg6
TGGACGTTCACACTATTGCTCCTAGGTGC
>SRR4252605_7636189___Sbg6
TGGACGTTCACACTATTGCTCCTAGGTGC
>SRR4252606_10418676___Sbg6
TGGACGTTCACACTATTGCTCCTAGGTGC
>SRR4252606_3901230___Sbg6
TGGACGTTCACACTATTGCTCCTAGGTGC
>SRR4252606_4659908___Sbg6
TGGACGTTCACACTATTGCTCCTAGGTGC
>SRR4252606_6443610___Sbg6
TGGACGTTCACACTATTGCTCCTAGGTG
>SRR4252607_11860559___Sbg6
TGGACGTTCACACTATTGCTCCTAGGTG
>SRR4252607_452077___Sbg6
TGGACGTTCACACTATTGCTCCTAGGTGC
>SRR4252607_6068228___Sbg6
TGGACGTTCACACTATTGCTCCTAGGTGC
>SRR4252607_6956373___Sbg6
TGGACGTTCACACTATTGCTCCTAGGTGC
>SRR4252607_7490108___Sbg6
TGGACGTTCACACTATTGCTCCTAGGTGC
>SRR4252608_10766046___Sbg6
TGGACGTTCACACTATTGCTCCTAGGTGC
>SRR4252608_1786092___Sbg6
TGGACGTTCACACTATTGCTCCTAGGT

>SRR4252608_4803606___Sbg6
TGGACGTTCACACTATTGCTCCTAGGTGC
>SRR4252608_5113976___Sbg6
TGGACGTTCACACTATTGCTCCTAGGTG
>SRR4252608_7807714___Sbg6
TGGACGTTCACACTATTGCTCCTAGGTGC
>SRR4252609_15665072___Sbg6
TGGACGTTCACACTATTGCTCCTAGGTG
>SRR4252609_1935823___Sbg6
TGGACGTTCACACTATTGCTCCTAGGTG
>SRR4252610_10882125___Sbg6
TGGACGTTCACACTATTGCTCCTAGGTGC
>SRR4252610_12089802___Sbg6
TGGACGTTCACACTATTGCTCCTAGGT
>SRR4252610_1481104___Sbg6
TGGACGTTCACACTATTGCTCCTAGGTGC
>SRR4252610_3214828___Sbg6
TGGACGTTCACACTATTGCTCCTAGGTGC
>SRR4252610_4371162___Sbg6
TGGACGTTCACACTATTGCTCCTAGGTGC
>SRR4252610_4881357___Sbg6
TGGACGTTCACACTATTGCTCCTAGGTGC
>SRR4252610_5068480___Sbg6
TGGACGTTCACACTATTGCTCCTAGGTGC
>SRR4252610_5440430___Sbg6
TGGACGTTCACACTATTGCTCCTAGGTGC
>SRR4252610_5660141___Sbg6
TGGACGTTCACACTATTGCTCCTAGGTG
>SRR4252610_5710486___Sbg6
TGGACGTTCACACTATTGCTCCTAGGTGC
>SRR4252610_5834687___Sbg6
TGGACGTTCACACTATTGCTCCTAGGTGC
>SRR4252610_6976669___Sbg6
TGGACGTTCACACTATTGCTCCTAGGTGC
>SRR4252610_7005268___Sbg6
TGGACGTTCACACTATTGCTCCTAGGT
>SRR4252610_7169099___Sbg6
TGGACGTTCACACTATTGCTCCTAGGTGC
>SRR4252610_7343350___Sbg6
TGGACGTTCACACTATTGCTCCTAGGTGC
>SRR4252610_7433405___Sbg6
TGGACGTTCACACTATTGCTCCTAGGTGC
>SRR4252610_9437627___Sbg6
TGGACGTTCACACTATTGCTCCTAGGTGC
>SRR4252611_12199322___Sbg6
TGGACGTTCACACTATTGCTCCTAGGTGC
>SRR4252611_2651949___Sbg6
TGGACGTTCACACTATTGCTCCTAGGTGC
>SRR4252611_7502805___Sbg6
TGGACGTTCACACTATTGCTCCTAGGTGC
>SRR4252612_6093381___Sbg6
TGGACGTTCACACTATTGCTCCTAGGTG
>SRR4252621_1839434___Sbg6
TGGACGTTCACACTATTGCTCCTAGGTG
>SRR4252623_10731926___Sbg6
TGGACGTTCACACTATTGCTCCTAGGTGC
>SRR4252624_4305633___Sbg6
TGGACGTTCACACTATTGCTCCTAGGTGC
>SRR4252619_5665607___Sbg6
TGGACGTTCACACTATTGCTCCTAGGAGCGC
>SRR4252605_10215728___Sbg6
GTATCTAATGGGCTGAATCTCGACTT
>SRR4252605_11607862___Sbg6
CGGATTTGGACGTTCACACTATTGCTCC
>SRR4252610_3110343___Sbg6
TGGACGTTCACACTATTGCTCCTAGGAGCGC
>SRR4252608_13197950___Sbg6
TGTATCTAATGGGCTGAATCTCGACTT
>SRR4252605_14396252___Sbg6
TCGACTTACTACGTCGTTGTCAGACCCGG

```
>SRR4252605_11011268___Sbg6
TGCCGACCCCAGGGATGAGAGTTTCTCCGA
>SRR4252608_1541915___Sbg6
TGGACGTTCACACTATTGCTCCTAGGAGCGC
>SRR4252611_13866034___Sbg6
CCAAGGGTTCTTTTACATGCCATCTA
>SRR4252611_6348854___Sbg6
TTGGACGTTCACACTATTGCTCCTAGG
>SRR4252611_13403334___Sbg6
TGGGTACGAAACTTGGCCAGGATTTAATTT
>SRR4252612_9146598___Sbg6
GGTGTACCCAAGGGTTCTTTTACATGCCA
>SRR4252608_2547776___Sbg6
TGTATCTAATGGGCTGAATCTCGACTT
>SRR4252608_10249807___Sbg6
CCGACCCCAGGGATGAGAGTTTCTCCGA
>SRR4252611_12340326___Sbg6
ACCCAAGGGTTCTTTTACATGCCATCTA
>SRR4252610_113678___Sbg6
CGGATTTGGACGTTCACACTATTGCTCT
>SRR4252610_11971173___Sbg6
TGGACGTTCACACTATTGCTCCTAGGAGCGC
>SRR4252611_7564848___Sbg6
TGCGTGCCATGGATTTTTTGTACCCA
>SRR4252608_8210022___Sbg6
TGGACGTTCACACTATTGCTCCTAGGAGCGC
>SRR4252605_2648527___Sbg6
TGGACGTTCACACTATTGCTCCTAGG
>SRR4252610_10826193___Sbg6
TGGACGTTCACACTATTGCTCCTAGGA
>SRR4252610_1215468___Sbg6
TGGACGTTCACACTATTGCTCCTAGGA
>SRR4252610_3567012___Sbg6
TTGGACGTTCACACTATTGCTCCTAGG
>SRR4252619_7839327___Sbg6
CCGACCCCAGGGATGAGAGTTTCTCCGA
>SRR4252624_8328222___Sbg6
ACCCGGCCGACCCCAGGGATGAGAGTTTC
>SRR4252623_10629616___Sbg6
TTGGACGTTCACACTATTGCTCCTAGGA
>SRR4252608_12721649___Sbg6
TCTAATGGGCTGCATCTCGACTTACTACG
>SRR4252605_7087556___Sbg6
CCGACCCCAGGGATGAGAGTTTCTCCGAG
>SRR4252619_12691576___Sbg6
TACCCAAGGGTTCTTTTACATGCCATCTA
>SRR4252605_3941736___Sbg6
CCATGGGTTCTTTTACATGCCATCTA
>SRR4252610_1115472___Sbg6
TTGGACGTTCACACTATTGCTCCTAGG
>SRR4252610_4061281___Sbg6
TGGACGTTCACACTATTGCTCCTAGGAGCGC
>SRR4252610_9829402___Sbg6
TGGACGTTCACACTATTGCTCCTAGGAGCGC
>SRR4252611_2686714___Sbg6
TGGACTCTATTTGGGTACGAAACTTGGC
>SRR4252611_7778627___Sbg6
TGTATCTAATGGGCTGAATCTCGACTT
>SRR4252608_4840790___Sbg5
GAGTGGTGGTGTACAACTTCTGATCACTAA
>SRR4252607_14028218___Sbg5
GTGGTGGTGTACAACTTCTGATCACTAA
>SRR4252619_9998431___Sbg5
CCTTGGCCCACATAGGGCTGTTGAGCTAT
>SRR4252620_8851091___Sbg5
TATGTAGAGAAGGCATATGTCATTGTTG
>SRR4252608_12553398___Sbg5
TCTTTCATATCCATAATCGTCACTCACAT
>SRR4252614_9742567___Sbg5
ACATCATACCACACCACACACACAATT
```

>SRR4252611_14439547___Sbg5
GGGTTTCACCTTCTCCCTCTTTCATATCCA
>SRR4252626_2550387___Sbg5
GTCTAGAAAACCGGGGCTATCGGCTGTGGG
>SRR4252610_11459238___Sbg5
CTCTTTCATATCCATAATCGTCACTCAC
>SRR4252613_3087186___Sbg5
CATATCCATAATCGTCACTCACATCA
>SRR4252608_13104074___Sbg5
TGTACAACTTCTGATCACTAAATTGTGC
>SRR4252611_9486247___Sbg5
AGTGGTGGTGTACAACTTCTGATCACTAA
>SRR4252609_14554451___Sbg5
ACATGCCGGCACCGGGTTTCACCTTC
>SRR4252608_2089046___Sbg5
CCATACTACCCCCCGTTAATTCCGTCCTA
>SRR4252620_1494920___Sbg5
TGTACAACTTCTGATCACTAAATTGTG
>SRR4252621_2770207___Sbg5
GAGTGGTGGTGTACAACTTCTGATCACTAA
>SRR4252623_14389663___Sbg5
GGTGGTGGTGTACAACTTCTGATCACTAA
>SRR4252621_13256009___Sbg5
ACACTCATCCCCCTATCCACACACTTACA
>SRR4252605_6688349___Sbg5
TGGTGTACAACTTCTGATCACTAAATTGT
>SRR4252611_7197672___Sbg5
AGTGATCCGTCCGTCGGATGGGGACGTTA
>SRR4252611_1639720___Sbg5
TGGTGTACAACTTCTGATCACTAAATTGT
>SRR4252606_5799198___Sbg5
GCTCATCCCCCTATCCACACACTTACA
>SRR4252611_9973022___Sbg5
AGTGGTGGTGTACAACTTCTGATCACTAA
>SRR4252606_2238722___Sbg5
GAGTGGTGGTGTACAACTTCTGATCACTAA
>SRR4252610_5280370___Sbg5
GAGTGGTGGTGTACAACTTCTGATCACTAA
>SRR4252621_4105310___Sbg5
TTCATATCCATAATCGTCACTCACATCAT
>SRR4252624_7085522___Sbg5
CCATACTACCCCCCGTTAATTCCGTCCTA
>SRR4252605_1698955___Sbg5
GGAGTGGTGGTGTACAACTTCTGATCACT
>SRR4252605_7460207___Sbg5
CTTGGTGCTTTTCGAAAGGAGTAGGCT
>SRR4252605_5609660___Sbg5
GGAGTGGTGGTGTACAACTTCTGATCACTA
>SRR4252610_10935024___Sbg5
GAGTGGTGGTGTACAACTTCTGATCACTAA
>SRR4252625_8288403___Sbg5
TTGATAGTGATCCGTCCGTCGGAAGGGG
>SRR4252606_1251684___Sbg5
TATGTAGAGAAGGCATATGTCATTGTTG
>SRR4252611_2285761___Sbg5
TTGATAGTGATCCGTCCGTCGGATGGGGA
>SRR4252621_13480369___Sbg5
AGTGATCCGTCCGTCGGATGGGGACG
>SRR4252608_2546631___Sbg5
ACATACACAATACAACACTATACACACA
>SRR4252622_3157804___Sbg5
TTGTTGATAGTGATCCGTCCGTCGGATGG
>SRR4252605_6812550___Sbg5
CCATACTACCCCCCGTTAATTCCGTCCTA
>SRR4252616_10480541___Sbg5
CTTGGCCCACATAGGGCTGTTGAGCTATA
>SRR4252609_13335271___Sbg5
GTGATCCGTCCGTCGGATGGGGACGTTAAA
>SRR4252612_5945780___Sbg5
GAGTGGTGGTGTACAACTTCTGATCACTAA

>SRR4252605_8454607___Sbg5
CCAGGTTTCACCTTCTCCCTCTTTCATA
>SRR4252624_10422811___Sbg5
GAGTGGTGGTGTACAACTTCTGATCACTAA
>SRR4252611_10140779___Sbg5
ACCGGGTTTCACCTTCTCCCTCTTTCATA
>SRR4252610_12502269___Sbg5
TGGTGTACAACTTCTGATCACTAAATTGC
>SRR4252611_625286___Sbg5
ACACACTTGCACACCAATCCATTAGCT
>SRR4252621_8044345___Sbg5
TTTCATATCCATAATCGTCACTCACATCA
>SRR4252625_9862828___Sbg5
TCATATCCATAATCGTCACTCACATCATA
>SRR4252608_5668352___Sbg5
GAGTGGTGGTGTACAACTTCTGATCACTAA
>SRR4252615_10883113___Sbg5
GTGGTGTACAACTTCTGATCACTAAATTGT
>SRR4252625_7172359___Sbg5
GGCTATCGGCTGTGGGGACTAGCGTAGT
>SRR4252609_5758148___Sbg5
TGGTGGTGTACAACTTCTGATCACTAAA
>SRR4252611_4947223___Sbg5
GAGTGGTGGTGTACAACTTCTGATCACTAA
>SRR4252625_3290553___Sbg5
TCGGCTGTGGGGACCAGCGTAGTACTGACCA
>SRR4252612_6580174___Sbg5
CCGGTCCCCTTGGTGCTTTTCGAAAGGA
>SRR4252606_6445312___Sbg5
TTGATAGTGATCCGTCCGTCGGATGGGG
>SRR4252615_8528960___Sbg5
TTAATTCCGTCCTATGGCGGCAGCCGCTC
>SRR4252610_10180047___Sbg5
TGTACAACTTCTGATCACTAAATTGT
>SRR4252606_3266478___Sbg5
GAGTGGTGGTGTACAACTTCTGATCACTAA
>SRR4252611_123815___Sbg5
GCACCGGGTTTCACCTTCTCCCTCTTTCA
>SRR4252611_1126593___Sbg5
GTTTCACCTTCTCCCTCTTTCATATCCA
>SRR4252610_7184196___Sbg5
TTGATAGTGATCCGTCCGTCGGATGGGG
>SRR4252620_3537143___Sbg5
TCCATAATCGTCACTCACATCATACCACA
>SRR4252607_11535707___Sbg5
TCGGCTGTGGGGACCAGCGTAGTACTG
>SRR4252611_9382589___Sbg5
AGGAGTGGTGGTGTACAACTTCTGATCACT
>SRR4252610_10787110___Sbg5
TGCTTTTCGAAAGGAGTAGGCTACATGC
>SRR4252620_7736273___Sbg5
TATGTAGAGAAGGCATATGTCATTGTTGA
>SRR4252612_6325805___Sbg5
ATGTCATTGTTGATAGTGATCCGTCCGT
>SRR4252606_7907155___Sbg5
GAGTGGTGGTGTACAACTTCTGATCACTAA
>SRR4252611_3876641___Sbg5
AGAAGGCATACGTCATTGTTGATAGTGA
>SRR4252605_1241933___Sbg5
AAGGCATACGTCATTGTTGATAGTGA
>SRR4252609_15263707___Sbg5
GTGATCCGTCCGTCGGATGGGGACGTTA
>SRR4252611_9371101___Sbg5
CCGGGTTTCACCTTCTCCCTCTTTCATA
>SRR4252613_11409088___Sbg5
TCCATAATCGTCACTCACATCATACCACAC
>SRR4252613_11448656___Sbg5
TCCATAATCGTCACTCACATCATACCACAC
>SRR4252605_10826157___Sbg5
AATCGTCACTCACATCATACCACACCA

>SRR4252608_9038126___Sbg5
TGTACAACTTCTGATCACTAAATTGT
>SRR4252625_2159199___Sbg5
TCTTTCATATCCATAATCGTCACTCACA
>SRR4252606_1086688___Sbg5
CATGCCGGCACCGGGTTTCACCTTCTCC
>SRR4252611_6015778___Sbg5
TTCATATCCATAATCGTCACTCACATCA
>SRR4252612_7646732___Sbg5
GAGTGGTGGTGTACAACTTCTGATCACTAA
>SRR4252622_1075896___Sbg5
GAGAAGGCATATGTCATTGTTGATAGTGA
>SRR4252610_10768641___Sbg5
GAGTGGTGGTGTACAACTTCTGATCACTAA
>SRR4252617_9221420___Sbg5
TAGAAAACCGGGGCTATCGGCTGTGGGG
>SRR4252625_7905407___Sbg5
CCAGGTTTCACCTTCTCCCTCTTTCATA
>SRR4252610_992047___Sbg5
GAGTGGTGGTGTACAACTTCTGATCACTA
>SRR4252619_6414099___Sbg5
CCCCCTATCCACACACTTACACACCA
>SRR4252621_10620572___Sbg5
TTGATAGTGATCCGTCCGTCGGATGG
>SRR4252610_3554507___Sbg5
CCATACTACCCCCCGTTAATTCCGTCCTA
>SRR4252605_8565617___Sbg5
AGCCGCTCGGTAAGCCTTGGCCCACAT
>SRR4252611_1044___Sbg5
GGAGTGGTGGTGTACAACTTCTGATCACT
>SRR4252606_2227218___Sbg5
TGGTGTACAACTTCTGATCACTAAATTGTGC
>SRR4252612_8827493___Sbg5
TTGATAGTGATCCGTCCGTCGGATGGGGAC
>SRR4252611_1173192___Sbg5
TAGTGATCCGTCCGTCGGATGGGGACGT
>SRR4252619_3078251___Sbg5
GTGTACAACTTCTGATCACTAAATTGT
>SRR4252606_2053544___Sbg5
GAGTGGTGGTGTACAACTTCTGATCACTAA
>SRR4252608_11282376___Sbg5
TTCATATCCATAATCGTCACTCACATCAT
>SRR4252616_14361796___Sbg5
CATATCCATAATCGTCACTCACATCATA
>SRR4252605_9342373___Sbg5
ATACTACCCCCCGTTAATTCCGTCCTA
>SRR4252608_11274482___Sbg5
TTGATAGTGATCCGTCCGTCGGATGGGGAC
>SRR4252605_8104807___Sbg5
TGTACAACTTCTGATCACTAAATTGT
>SRR4252620_3968376___Sbg5
TCCATAATCGTCACTCACATCATACC
>SRR4252607_6819953___Sbg5
TAGAAAACCGGGGCTATCGGCTGTGGGG
>SRR4252622_169768___Sbg5
CATATCCATAATCGTCACTCACATCA
>SRR4252619_14697477___Sbg5
CATCATACCACACCACACACACAATACAA
>SRR4252622_44695___Sbg5
ACCGTGTTTCACCTTCTCCCTCTTTCATA
>SRR4252614_5850973___Sbg5
GCGTGGTGGTGTACAACTTCTGATCACTAA
>SRR4252622_6084829___Sbg5
GCGTGGTGGTGTACAACTTCTGATCACTAA
>SRR4252611_8608359___Sbg5
AGCTGGTCTAGAAAACCGGGGTTATCGGCT
>SRR4252608_3325987___Sbg5
CCGTCCGTCGGATGGGGACGTTAAGCCT
>SRR4252622_1575076___Sbg5
CGTCCGTCGGATGGGGACGTTAAGCCT

>SRR4252606_5234292___Sbg5
ACTTTTCGAAAGGAGTAGGCTACATGC
>SRR4252618_10703122___Sbg5
TATCCATAATCGTCACTCACATCATA
>SRR4252606_9920499___Sbg5
TGGTGTACAACTTCTGATCACTAAATTGT
>SRR4252623_12647920___Sbg5
CGTCCGTCGGATGGGGACGTTAAGCCG
>SRR4252621_13461124___Sbg5
TGGTGTACAACTTCTGATCACTAAATTGT
>SRR4252624_3159411___Sbg5
CCAGGTTTAACCTTCTCCCTCTTTCATA
>SRR4252609_4339304___Sbg5
TTAACCTTCTCCCTCTTTCATATCCATAA
>SRR4252621_13617413___Sbg5
ACCGTCACTCACATCATACCACACCACACA
>SRR4252605_7210579___Sbg5
TCACACACTTACACACCGATCCATTAACTG
>SRR4252616_9875981___Sbg5
CTTGGCCCACATAGGGCTGTTGAGCT
>SRR4252605_7787035___Sbg5
AGTGGTGGTGTACAACTTCTGATCACTAA
>SRR4252605_10920970___Sbg5
ATCCGTCCGTCGGATGGGGACGTTAT
>SRR4252613_7252136___Sbg5
TCACACACTTACACACCGATCCATTAACT
>SRR4252616_9691426___Sbg5
TTCACCTTCTCCCTCTTTCATATCCATA
>SRR4252605_14049927___Sbg5
GCCGGTCCCCTTGGTGCTTTTCGAAAGGA
>SRR4252608_10874559___Sbg5
CCATACTACCCCCCGTTAATTCCGTCCTA
>SRR4252624_6015605___Sbg5
CCATACTACCCCCCGTTAATTCCGTCCTA
>SRR4252612_1094664___Sbg5
GATAGTGATCCGTCCGTCGGATGGGGACGTT
>SRR4252619_11111189___Sbg5
TACAACTTCTGATCACTAAATTGTGCGC
>SRR4252611_9058466___Sbg5
ACCGGGTTTCACCTTCTCCCTCTTTCATA
>SRR4252605_14266194___Sbg5
CCTTACTACCCCCCGTTAATTCCGTCCTA
>SRR4252620_4188969___Sbg5
GAAGGCATTTGTCATTGTTGATAGTGA
>SRR4252611_12343564___Sbg5
CCGGGTTTCACCTTCTCCCTCTTTCATA
>SRR4252606_6640705___Sbg5
TATCCATAATCGTCACTCACATCATAC
>SRR4252612_150891___Sbg5
ACAATACAACACTATACACACACTCAT
>SRR4252611_5401199___Sbg5
TCGGCTGTGGGGACCAGCATAGTACTGA
>SRR4252615_3950945___Sbg5
TCGGCTGTGGGGACCAGCATAGTACTG
>SRR4252616_4258340___Sbg5
TCGGCTGTGGGGACCAGCATAGTACTG
>SRR4252625_5983602___Sbg5
TGGTGTACAACTTCTGATCACTAAATTGT
>SRR4252613_585734___Sbg5
CCATACTACCCCCCGTTAATTCCGTCCTA
>SRR4252606_1568548___Sbg5
CCACACCACACACACAATACAACACTA
>SRR4252611_12318436___Sbg5
GTGTACAACTTCTGATCACTAAATTGT
>SRR4252608_4557018___Sbg5
TGTACAACTTCTGATCACTAAATTGT
>SRR4252613_3833681___Sbg5
TACAACTTCTGATCACTAAATTGTGCAC
>SRR4252608_10654718___Sbg5
TATGTAGAGAAGGCATATGTCATTGTTGA

>SRR4252614_389119___Sbg5
ACACCTTCTCCCTCTTTCATATCCATAA
>SRR4252616_13052693___Sbg5
TCGGCTGTGGGGACCAGCGTAGTACTGA
>SRR4252607_5375652___Sbg5
CTGTGGGGACCAGCGTAGTACTGACCATACT
>SRR4252608_2627878___Sbg5
GAGTGGTGGTGTACAACTTCTGATCACTAA
>SRR4252605_4369473___Sbg5
TCGGATGGGGACGTTAAGCCGTCGGACCCC
>SRR4252608_6141438___Sbg5
TCTCCCTCTTTCATATCCATAATCGTCAC
>SRR4252612_4309964___Sbg5
GGTGGTGGTGTACAACTTCTGATCACTAA
>SRR4252606_8707342___Sbg5
CATACTACCCCCCGTTAATTCCGTCCTA
>SRR4252616_9308708___Sbg5
CATACTACCCCCCGTTAATTCCGTCCTA
>SRR4252614_5626359___Sbg5
CTTGGCCCACATAGGGCTGTTGAGCTATA
>SRR4252605_892012___Sbg5
CATATCCATAATCGTCACTCACATCAT
>SRR4252608_10386236___Sbg5
TCCATAATCGTCACTCACATCATACCACA
>SRR4252623_10257380___Sbg5
TCGGCTGTGGGGACCAGCGTAGTGCTGAC
>SRR4252610_869674___Sbg5
GAGTGGTGGTGTACAACTTCTGATCACTAA
>SRR4252612_4519546___Sbg5
GTGTACAACTTCTGATCACTAAATTTT
>SRR4252612_8255600___Sbg5
CACCGGGTTTCACCTTCTCCCTCTTTCA
>SRR4252612_7279450___Sbg5
TTGATAGTGATCCGTCCGTCGGATGGGGAC
>SRR4252612_6437051___Sbg5
CATCATACCACACCACACACACAATACA
>SRR4252618_3667873___Sbg5
GTATCCATAATCGTCACTCACATCATA
>SRR4252623_8685740___Sbg5
GTATCCATAATCGTCACTCACATCATA
>SRR4252610_334813___Sbg5
ACACACACAATACAACACTATACACAC
>SRR4252605_8144382___Sbg5
GAGTGGTGGTGTACAACTTCTGATCACTAA
>SRR4252610_4277496___Sbg5
TGTACAACTTCTGATCACTAAATTGT
>SRR4252608_1533064___Sbg5
TTGATAGTGATCCGTCCGTCGGATGGGGAC
>SRR4252608_7763418___Sbg5
TATCCACACACTTACACACCGATCCATTA
>SRR4252621_10291408___Sbg5
GAGTGGTGGTGTACAACTTCTGATCACTAA
>SRR4252608_1068995___Sbg5
GAGTGGTGGTGTACAACTTCTGATCACTAA
>SRR4252611_11871801___Sbg5
CCATACTACCCCCCGTTAATTCCGTCCTA
>SRR4252605_7492934___Sbg5
TATGTAGAGAAGGCATATGTCATTGTTG
>SRR4252605_3310734___Sbg5
CATTGTTGATAGTGATCCGTCCGTCGGTTT
>SRR4252609_15809737___Sbg5
GGAGTGGTGGTGTACAACTTCTGATCAC
>SRR4252621_4579401___Sbg5
ATCCATAATCGTCACTCACATCATACCAC
>SRR4252613_6944079___Sbg5
AATGTCATTGTTGATAGTGATCCGTCCGTC
>SRR4252616_4289420___Sbg5
AAAGGAGTAGGCTACATGCCGGCACCGGG
>SRR4252621_1573857___Sbg5
TCATATCCATAATCGTCACTCACATCA

>SRR4252621_9120695___Sbg5
CATATCCATAATCGTCACTCACATCATA
>SRR4252620_60152___Sbg5
TATCGGCTGTGGGGACCAGCGTAGTACTA
>SRR4252610_1442580___Sbg5
TATCCACACACTTACACACCGATCCATTA
>SRR4252605_3230494___Sbg5
ATTCCGTCCTATGGCGGCAGCCGCTCGGTA
>SRR4252608_8483134___Sbg5
TGTACAACTTCTGATCACTAAATTGT
>SRR4252605_4458573___Sbg5
GAGTGGTGGTGTACAACTTCTGATCACTAA
>SRR4252619_14339246___Sbg5
TGGTGTACAACTTCTGATCACTAAAT
>SRR4252618_10559318___Sbg5
GTGGGGACCAGCGTAGTACTGACCATA
>SRR4252610_6098258___Sbg5
CAATACTACCCCCCGTTAATTCCGTCCTA
>SRR4252606_4774601___Sbg5
GAGTGGTGGTGTACAACTTCTGATCACTA
>SRR4252622_5416752___Sbg5
GTCCACACACTTACACACCAATCCATTAG
>SRR4252624_8471343___Sbg5
TTATCGGCTGTGGGGACCAGCGTAGTAAT
>SRR4252612_6546494___Sbg5
CCGGTCCCCTTGGTGCTTTTCGAAAGGA
>SRR4252611_582686___Sbg5
TCACCTTCTCCCTCTTTCATATCCATAA
>SRR4252624_8119345___Sbg5
ACCGGGTTTCACCTTCTCCCTCTTTCATA
>SRR4252611_6286938___Sbg5
GAGTGGTGGTGTACAACTTCTGATCACTA
>SRR4252624_1027019___Sbg5
GAGTGGTGGTGTACAACTTCTGATCACTAA
>SRR4252606_3566574___Sbg5
TATCCACACACTTACACACCAATCCATTA
>SRR4252605_9935721___Sbg5
CCATACTACCCCCCGTTAATTCCGTCCTA
>SRR4252605_11579328___Sbg5
TTGGCCCACATAGGGCTGTTGAGCTATAGA
>SRR4252608_294940___Sbg5
TATCCACACACTTACACACCAATCCATTA
>SRR4252611_112112___Sbg5
CCATACTACCCCCCGTTAATTCCGTCCTA
>SRR4252611_1978811___Sbg5
TCGGGTTTCACCTTCTCCCTCTTTCATA
>SRR4252611_7634324___Sbg5
CACACTTACACACCAATCCATTAGCA
>SRR4252612_2667332___Sbg5
CCCTATCCACACACTTACACACCGATC
>SRR4252613_9630577___Sbg5
TCGGCTGTGGGGATCAGCGTAGTACTGAC
>SRR4252616_9044657___Sbg5
TGTACAACTTCTGATCACTAAATTGTGC
>SRR4252611_9744095___Sbg5
ACACCAATCCATTAGCTGGTCTAGAAAACCG
>SRR4252610_833964___Sbg5
GGAGTGGTGGTGTACAACTTCTGATCACT
>SRR4252607_6327696___Sbg5
TATGTAGAGAAGGCATATGTCATTGT
>SRR4252605_14761413___Sbg5
TGGTGTACAACTTCTGATCACTAAATTG
>SRR4252608_3969759___Sbg5
CACACTTACACACCGATCCATTAACT
>SRR4252605_5560780___Sbg5
GAGAGGTGGTGTACAACTTCTGATCACTAA
>SRR4252606_4593311___Sbg5
AGAGGTGGTGTACAACTTCTGATCACTAA
>SRR4252612_2637056___Sbg5
GAGTGGTGGTGTACAACTTCTGATCACTAA

>SRR4252623_1989435___Sbg5
GTTAAATCCCGAACTCCTCCGCAATG
>SRR4252610_8804388___Sbg5
TCATATCCATAATCGTCACTCACATCATA
>SRR4252612_2768694___Sbg5
TCATATCCATAATCGTCACTCACATCATA
>SRR4252605_7869540___Sbg5
ATATCCATAATCGTCACTCACATCATACC
>SRR4252611_68832___Sbg5
TTGATAGTGATCCGTCCGTCGGATGGGGAC
>SRR4252619_12336804___Sbg5
TTGATAGTGATCCGTCCGTCGGATGG
>SRR4252615_8915957___Sbg5
TTGTTGATAGTGATCCGTCCGTCGGATGG
>SRR4252608_12505718___Sbg5
CTCTTTCATATCCATAATCGTCACTCAC
>SRR4252611_2005083___Sbg5
TTGATAGTGATCCGTCCGTCGGATGGGGAC
>SRR4252618_5651921___Sbg5
TGTCATTGTTGATAGTGATCCGTCCGTC
>SRR4252625_777442___Sbg5
CGCTCGGTAAGCCTTGGCCCACATAGGACTG
>SRR4252613_6821896___Sbg5
ACTCACATCATACCACACCACACACAC
>SRR4252610_8412006___Sbg5
GCCGGTCCCCTTGGTGCTTTTCGAAAGGA
>SRR4252605_6537450___Sbg5
GAGTGGTGGTGTACAACTTCTGATCACTAA
>SRR4252605_207586___Sbg5
TGGTGTACAACTTCTGATCACTAAATTGT
>SRR4252606_3939523___Sbg5
TCGGCTGTGGGGACCAGCGTGGTACTGAC
>SRR4252610_9932397___Sbg5
GAGTGGTGGTGTACAACTTCTGATCACTAA
>SRR4252618_8062836___Sbg5
TATGTCATTGTTGATAGTGATCCGTCCGT
>SRR4252608_10658417___Sbg5
TTCATATCCATAATCGTCACTCACATCATA
>SRR4252624_980534___Sbg5
AGTGGTGGTGTACAACTTCTGATCACTAA
>SRR4252614_3299049___Sbg5
TGATAGTGATCCGTCCGTCGGATGGGGAT
>SRR4252611_2824466___Sbg5
TGATCCGTCCGTCGGATGGGGACGTTAAG
>SRR4252606_4548807___Sbg5
ACACAATACAACACTATACACACACT
>SRR4252619_14549276___Sbg5
TCTAGAAAACCGGGGCTATCGGCTGT
>SRR4252611_3156445___Sbg5
CCATACTACCCCCCGTTAATTCCGTCCTA
>SRR4252606_375834___Sbg5
GAGTGGTGGTGTACAACTTCTGATCACTAA
>SRR4252611_6134913___Sbg5
AATCCTGAGACTAGGAGGAGTGGTGGT
>SRR4252612_3491553___Sbg5
TATGTTGAGAAGGCATATGTCATTGTTG
>SRR4252610_9535468___Sbg5
CATATCCATAATCGTCACTCACATCATA
>SRR4252621_8694104___Sbg5
TCCACACACTTACACACCGATCCATTA
>SRR4252611_13547978___Sbg5
TATGTAGAGAAGGCATATGTCATTGTTG
>SRR4252606_762369___Sbg5
GAGTGGTGGTGTACAACTTCTGATCACTAA
>SRR4252610_7500536___Sbg5
GAGTGGTGGTGTACAACTTCTGATCACTA
>SRR4252615_1138327___Sbg5
TTGATAGTGATCCGTCCGTCGGATGG
>SRR4252606_4744460___Sbg5
GTGGTGGTGTACAACTTCTGATCACTA

>SRR4252609_15926380___Sbg5
TATGTAGAGAAGGCATATGTCATTGTTGA
>SRR4252606_8500089___Sbg5
TCGGTAAGCCTTGGCCCACATAGGGCTGC
>SRR4252611_11674114___Sbg5
CCATACTACACCCCGTTAATTCCGTCCTA
>SRR4252605_6268349___Sbg5
TGGTGTACAACTTCTGATCACTAAATTGT
>SRR4252619_5584297___Sbg5
GAGTGGTGGTGTACAACTTCTGATCACTA
>SRR4252610_8516007___Sbg5
ACACTCATCCCCCTATCCACACACTTACA
>SRR4252619_169818___Sbg5
TTGGCCCACATAGGGCTGTTGAGCTAT
>SRR4252611_2335688___Sbg5
GGTGGTATACAACTTCTGATCACTAA
>SRR4252613_829345___Sbg5
CTACATGCCGGCACCGGGTTTCACCT
>SRR4252613_4399727___Sbg5
TCGGCTGTGGGGATCAGCGTAGTACTG
>SRR4252623_8294908___Sbg5
TCCATAATCGTCACTCACATCATACCACA
>SRR4252605_143290___Sbg5
TGGTGTACAACTTCTGATCACTAAATTGT
>SRR4252610_5159882___Sbg5
TGTACAACTTCTGATCACTAAATTGTA
>SRR4252616_3334210___Sbg5
GGTGGTGTACAACTTCTGATCACTAAATTGC
>SRR4252605_13577638___Sbg5
TGGTGTACAACTTCTGATCACTAAATTGC
>SRR4252606_4820645___Sbg5
TGGTGTACAACTTCTGATCACTAAATTGC
>SRR4252608_12569847___Sbg5
TGGTGTACAACTTCTGATCACTAAATTGC
>SRR4252611_5633052___Sbg5
CATACTACCCCCCGTTAATTCCGTCCTA
>SRR4252612_5853627___Sbg5
TGATAGTGATCCGTCCGTCGGATGGGGAC
>SRR4252615_9917442___Sbg5
CCTTGGCCCACATAGGGCTGTTGCGCTAT
>SRR4252607_6131190___Sbg5
AGAGTGGTGGTGTACAACTTCTGATCACTA
>SRR4252611_867443___Sbg5
AGAGTGGTGGTGTACAACTTCTGATCACTAA
>SRR4252614_7434244___Sbg5
AGAGTGGTGGTGTACAACTTCTGATCACTA
>SRR4252621_580944___Sbg5
AGAGTGGTGGTGTACAACTTCTGATCACTAA
>SRR4252611_1002136___Sbg5
TGTTGATAGTGATCCGTCCGTCGGATGG
>SRR4252608_6494899___Sbg5
GAGTGGTGGTGTACAACTTCTGATCACTAA
>SRR4252611_6997631___Sbg5
CACCGGGTTTCACCTTCTCCCTCTTTCATA
>SRR4252605_12567125___Sbg5
GAGAAGGCATATGTCATTGTTGATAGTGA
>SRR4252612_5470395___Sbg5
TGATAGTGATCCGTCCGTCGGATGGGGA
>SRR4252607_11731249___Sbg5
ACACTCATCCTCCTATCCACACACTTACA
>SRR4252606_7549418___Sbg5
GAGTGGTGGTGTACAACTTCTGATCACTAA
>SRR4252612_6396049___Sbg5
GAGTGGTGGTGTACAACTTCTGATCACTA
>SRR4252612_7445664___Sbg5
GAGAAGGCATATGTCATTGTTGATAGTGA
>SRR4252610_12607416___Sbg5
GGTTTCACCTTCTCCCTCTTTCATATCCA
>SRR4252609_10839969___Sbg5
CCATACTACCCCCCGTTAATTCCGTCCTA

>SRR4252609_6392129___Sbg5
GAATGGTGGTGTACAACTTCTGATCACTAA
>SRR4252605_14123570___Sbg5
TATGTAGAGAAGGCATATGTCATTGTTG
>SRR4252625_3298218___Sbg5
CCATACTACCCCCCGTTAATTCCGTCCTA
>SRR4252613_2283245___Sbg5
TTGATAGTGATCCGTCCGTCGGATGGGGAC
>SRR4252613_1163788___Sbg5
GATCCGTCCGTCGGATGGGGACGTTAAG
>SRR4252611_4974601___Sbg5
GGTGGTGTACAACTTCTGATCACTAA
>SRR4252607_111956___Sbg5
ATGCCGGCACCGGGTTTCACCTTCTCT
>SRR4252622_1079591___Sbg5
TATGTAGAGAAGGCATATGTCATTGTTG
>SRR4252611_5737282___Sbg5
AGTGGTGGTGTACAACTTCTGATCACTAA
>SRR4252612_8492415___Sbg5
ATTCCGTCCTATGGCGGCAGCCGCTCGGTA
>SRR4252622_9332861___Sbg5
GGTTTCACATTCTCCCTCTTTCATATCCA
>SRR4252623_4058552___Sbg5
TCATATCCATAATCGTCACTCACATCATA
>SRR4252624_8230572___Sbg5
GAATGGTGGTGTACAACTTCTGATCACTAA
>SRR4252608_7818862___Sbg5
TGGTGGTGTACAACTTCTGATCACTAA
>SRR4252617_2889541___Sbg5
TCACACACTTACACACCGATCCATTA
>SRR4252610_12079465___Sbg5
CTCTTTCATATCCATAATCGTCACTCA
>SRR4252622_3664211___Sbg5
TGTTGATAGTGATCCGTCCGTCGGATGT
>SRR4252607_3712388___Sbg5
TTGATAGTGATCCGTCCGTCGGATGT
>SRR4252614_4258084___Sbg5
TTCATATCCATAATCGTCACTCACATCAT
>SRR4252606_9312449___Sbg5
GCCGGTCCCCTTGGTGCTTTTCGAAAGGA
>SRR4252624_8116096___Sbg5
TTGATAGTGATCCGTCCGTCGGATGGGGAC
>SRR4252607_10882774___Sbg5
TGGTGTACAACTTCTGATCACTAAATTGA
>SRR4252605_6373757___Sbg5
GGTGCTGGTGTACAACTTCTGATCACTAA
>SRR4252610_518920___Sbg5
TGTTGATAGTGATCCGTCCGTCGGATGG
>SRR4252605_13899293___Sbg5
ATCATATCCATAATCGTCACTCACATCATA
>SRR4252619_10910754___Sbg5
ATCATATCCATAATCGTCACTCACATCATA
>SRR4252623_2005969___Sbg5
ATCATATCCATAATCGTCACTCACATCATA
>SRR4252605_9205755___Sbg5
TGGTGTACAACTTCTGATCACTAAATTGT
>SRR4252621_8063562___Sbg5
CATATCCATAATCGTCACTCACATCATA
>SRR4252616_11693046___Sbg5
GAGTGATGGTGTACAACTTCTGATCACTAA
>SRR4252610_13137104___Sbg5
GAGTGGTGGTGTACAACTTCTGATCACTAA
>SRR4252625_12474375___Sbg5
GAGTGGTGGTGTACAACTTCTGATCACTAA
>SRR4252619_4390154___Sbg5
CACACACACAGTACAACACTATACACACA
>SRR4252605_434405___Sbg5
AAGTGGTGGTGTACAACTTCTGATCACTAA
>SRR4252610_304225___Sbg5
TCATATCCATAATCGTCACTCACATCATA

>SRR4252610_13008828___Sbg5
TGGTGGTGTACAACTTCTGATCACTAAA
>SRR4252605_4436841___Sbg5
TTGATAGTGATCCGTCCGTCGGATGG
>SRR4252616_5976232___Sbg5
ACCGGGTTTCACCTTCTCCCTCTTTCAT
>SRR4252624_3417742___Sbg5
TCGGCTGTGGGGACCAGCGTAGTACTG
>SRR4252619_2040137___Sbg5
TCATACTACCCCCCGTTAATTCCGTCCTA
>SRR4252612_3650638___Sbg5
GTAGGCTACATGCCGGCACCGGGTTT
>SRR4252605_2817191___Sbg5
GAGTGGTGGTGTACAACTTCTGATCACTAA
>SRR4252621_8448852___Sbg5
GAGTGGTGGTGTACAACTTCTGATCACTAA
>SRR4252606_9909918___Sbg5
CCATACTACCCCCCGTTAATTCCGTCCTA
>SRR4252610_5915518___Sbg5
CATACCACACCACACACACAATACAACA
>SRR4252611_7146770___Sbg5
CCATACTACCCCCCGTTAATTCCGTCCTA
>SRR4252625_10371678___Sbg5
GGTGTACAACTTCTGATCACTAAATTGTG
>SRR4252610_378294___Sbg5
GAGAAGGCATATGTCATTGTTGATAGTGA
>SRR4252621_4646610___Sbg5
GAGTGGTGGTGTACAACTTCTGATCACTAA
>SRR4252610_8406076___Sbg5
GAGTGGTGGTGTACAACTTCTGATCACTAA
>SRR4252623_980248___Sbg5
GAGTGGTGGTGTACAACTTCTGATCACTAA
>SRR4252607_7081445___Sbg5
TTCATATCCATAATCGTCACTCACATCA
>SRR4252624_9305049___Sbg5
GGTTTCACCTTCTCCCTCTTTCATATCCA
>SRR4252605_2591037___Sbg5
TTCCGTCCTATGGCGGCAGCCGCTTGG
>SRR4252621_11793508___Sbg5
TGGTGTACAACTTCTGATCACTAAATTGT
>SRR4252609_380491___Sbg5
ATATCCATAATCGTCACTCACATCATA
>SRR4252611_9625343___Sbg5
CCTATTCACACACTTACACACCGATCCA
>SRR4252610_12840244___Sbg5
GAGTGGTGGTGTACAACTTCTGATCACTAA
>SRR4252612_8870076___Sbg5
TGATAGTGATCCGTCCGTCGGATGGGGACGC
>SRR4252605_14817262___Sbg5
ATATCCATAATCGTCACTCACATCAT
>SRR4252622_4366439___Sbg5
AGTGGTGGTGTACAACTTCTGATCACTAA
>SRR4252609_15174949___Sbg5
GGGGCTATCGGCTGTGGGGACCAGCGTAG
>SRR4252614_2138446___Sbg5
TATGTAGAGAAGGCATATGTCATTGTTG
>SRR4252608_3278051___Sbg5
ACCGGATTTCACCTTCTCCCTCTTTCATA
>SRR4252609_4070903___Sbg5
TTCATATCCATAATCGTCACTCACATC
>SRR4252616_9669622___Sbg5
TCCATAATCGTCACTCACATCATACCACA
>SRR4252610_11012166___Sbg5
GAGTGGTGGTGTACAACTTCTGATCACTA
>SRR4252610_7035412___Sbg5
GAGTGGTGGTGTACAACTTCTGATCACTAA
>SRR4252605_2952155___Sbg5
TGGTGTACAACTTCTGATCACTAAATTG
>SRR4252605_7842685___Sbg5
AGTACTGACCATACTACCCCCCGTTAA

>SRR4252614_7939644___Sbg5
GTACAACTTCTGATCACTAAATTGTGCCCC
>SRR4252612_6738060___Sbg5
GAGTAATTAATTAAAATTTCGAATCCT
>SRR4252614_969097___Sbg5
CCACACCACACACACAATACAACACTAT
>SRR4252620_5454210___Sbg5
TCACATCATACCACACCACACACACAATAC
>SRR4252611_5502326___Sbg5
TCCGTCCGTCGGATGGGGACGTTAAGC
>SRR4252605_4882065___Sbg5
TATGTAGAGAAGGCATATGTCATTGTTG
>SRR4252605_7662138___Sbg5
GAGAGGTGGTGTACAACTTCTGATCACTAA
>SRR4252605_12367962___Sbg5
GAGTGGTGGTGTACAACTTCTGATCACTAA
>SRR4252621_2086171___Sbg5
ATTCCGTCCTATGGCGGCAGCCGCTCGGTA
>SRR4252605_10161744___Sbg5
TTCATATCCATAATCGTCACTCACATC
>SRR4252605_3984343___Sbg5
CCGGGTTTCACCTTCTCCCTCTTTCATA
>SRR4252608_4381900___Sbg5
ATCCACACACTTACACACCGATCCATTA
>SRR4252611_907272___Sbg5
GTGATCCGTCCGTCGGATGGGGACGTTA
>SRR4252616_3444826___Sbg5
CCTCCGCAATGACTATGTAGAGAAGGCA
>SRR4252607_1633187___Sbg5
GCCGGGTTTCACCTTCTCCCTCTTTCATA
>SRR4252624_6250967___Sbg5
TGGTGGTGTACAACTTCTGATCACTAAA
>SRR4252608_6863212___Sbg5
TGTACAACTTCTGATCACTAAATTGT
>SRR4252613_6319369___Sbg5
GCCGGTCCCCTTGGTGCTTTTCGAAAGGA
>SRR4252624_10381876___Sbg5
GAGTGGTGGTGTACAACTTCTGATCACTAA
>SRR4252610_12633544___Sbg5
TGGTGTACAACTTCTGATCACTAAATTGT
>SRR4252611_3232508___Sbg5
TACCGGGTTTCACCTTCTCCCTCTTTCATA
>SRR4252608_4220759___Sbg5
TTGATAGTGATCCGTCCGTCGGATGG
>SRR4252605_9329519___Sbg5
TACAACTTCTGATCACTAAATTGTGC
>SRR4252606_4932880___Sbg5
ATCATATCCATAATCGTCACTCACATCATA
>SRR4252623_12363279___Sbg5
ATCATATCCATAATCGTCACTCACATCATA
>SRR4252609_11484148___Sbg5
GAGTGGTGGTGTACAACTTCTGATCACTAA
>SRR4252612_5505566___Sbg5
ACACACAATACAACACTATACACACTCA
>SRR4252610_8696759___Sbg5
ATATCCATAATCGTCACTCACATCATA
>SRR4252610_8854860___Sbg5
TCCATAATCGTCACTCACATCATACCACA
>SRR4252605_1279744___Sbg5
CACTCATCTTCCTATCCACACACTTACA
>SRR4252610_6382705___Sbg5
GAGTGGTGGTGTACAACTTCTGATCACTAA
>SRR4252611_5838376___Sbg5
GTGTACAACTTCTGATCACTAAATTG
>SRR4252610_3529456___Sbg5
TCATATCCATAATCGTCACTCACATCATA
>SRR4252620_2464935___Sbg5
GAAAGGAGTAGGCTACATGCCGGGACCAGGT
>SRR4252608_10133112___Sbg5
AAGGAGTAGGCTACATGCCGGGACCAG

```
>SRR4252606_3530197___Sbg5
GTAGGCTACATGCCGGGACCAGGTTTCA
>SRR4252607_12355136___Sbg5
GTAGGCTACATGCCGGGACCAGGTTTCA
>SRR4252610_10723227___Sbg5
GTAGGCTACATGCCGGGACCAGGTTT
>SRR4252611_3710017___Sbg5
GTAGGCTACATGCCGGGACCAGGTTTCA
>SRR4252613_5597016___Sbg5
GTAGGCTACATGCCGGGACCAGGTTTCAC
>SRR4252616_9270629___Sbg5
GTAGGCTACATGCCGGGACCAGGTTTCA
>SRR4252610_6962587___Sbg5
CCAGGTTTCACCTTCTCCCTCTTTCATA
>SRR4252611_7493852___Sbg5
AGTGGTGGTGTACAACTTCTGATCACTAA
>SRR4252606_2105211___Sbg5
ATGTCATTGCTGATAGTGATCCGTCCGT
>SRR4252619_7915647___Sbg5
CTTGGCCCACATAGGGCTGTTGAGCTATA
>SRR4252612_2437784___Sbg5
GAGTGGTGGTGTACAACTTCTGATCACTAA
>SRR4252609_15858193___Sbg5
CATATCCATAATCGTCACTCACATCATA
>SRR4252612_5949244___Sbg5
TAGAAAACCGGGGCTATCGGCTGTGGGT
>SRR4252608_3474258___Sbg5
GAGTGGTGGTGTACAACTTCTGATCACTAA
>SRR4252606_1703159___Sbg5
CCATACTACCCCCCGTTAATTCCGTCCTA
>SRR4252612_59458___Sbg5
CCGGGTTTCACCTTCTCCCTCTTTCATA
>SRR4252625_3030350___Sbg5
TGATAGTGAACCGTCCGTCGGATGGGGA
>SRR4252608_7281883___Sbg5
TCCGCAATGACTATGTAGAGAAGGCAT
>SRR4252611_2741663___Sbg5
TGATCCGTCCGTCGGATGGGGACGTT
>SRR4252621_1364224___Sbg5
ATTGTTGATAGTGATCCGTCCGTCGGA
>SRR4252616_14331661___Sbg5
ATTGTTGATAGTGATCCGTCCGTCGGA
>SRR4252622_10578948___Sbg5
GAGTGGTGGTGTACAACTTCTGATCACTAA
>SRR4252610_8389049___Sbg5
GAGTGGTGGTGTACAACTTCTGATCACTAA
>SRR4252612_6369672___Sbg5
TATGTCATTGTTGATAGTGATCCGTCCGT
>SRR4252615_7339360___Sbg5
CTCTTTCATATCCATAATCGTCACTC
>SRR4252611_9067984___Sbg5
GAGTGGTGGTGTACAACTTCTGATCACTAA
>SRR4252611_1398814___Sbg5
CATACTACCCCCGTTAATTCCGTCCTA
>SRR4252615_5540875___Sbg5
TAATTCCGTCCTATGGCGGCAGCCGCTCG
>SRR4252612_8764491___Sbg5
GTGGTGGTGTACAACTTCTGATCACTAAA
>SRR4252610_2564578___Sbg5
CACTATCCCCCGTTAATTCCGTCCTA
>SRR4252621_4612715___Sbg5
CATATCCATAATCGTCACTCACATCATA
>SRR4252610_8600736___Sbg5
GAGTGGTGGTGTACAACTTCTGATCACTAA
>SRR4252605_13291694___Sbg5
GTGTACAACTTCTGATCACTAAATTGTGT
>SRR4252607_2617502___Sbg5
TGGTGTACAACTTCTGATCACTAAATTGT
>SRR4252608_9496622___Sbg5
TGTACAACTTCTGATCACTAAATTGT
```

>SRR4252624_6393655___Sbg5
GAGTGGTGGTGTACAACTTCTGATCACTAA
>SRR4252606_8379972___Sbg5
AAGTGGTGGTGTACAACTTCTGATCACTA
>SRR4252611_9384908___Sbg5
AAGTGGTGGTGTACAACTTCTGATCACT
>SRR4252622_364588___Sbg5
AAGTGGTGGTGTACAACTTCTGATCACTAA
>SRR4252606_7420200___Sbg5
TGGTGTACAACTTCTGATCACTAAATTG
>SRR4252605_3933289___Sbg5
CCATACTACCCCCCGTTAATTCCGTCCTA
>SRR4252605_8766724___Sbg5
CGTCCGTCGGATGGGGACGTTAAGCCA
>SRR4252607_10971153___Sbg5
ACACACACAGTACAACACTATACACACA
>SRR4252621_1044798___Sbg5
TATCCATAATCGTCACTCACATCATAC
>SRR4252610_2320274___Sbg5
TGGTGGTGTACAACTTCTGATCACTAA
>SRR4252607_11619647___Sbg5
AGAAGGCATATGTCATTGTTGATAGTGA
>SRR4252605_12461864___Sbg5
ATACTACCCCCCGTTAATTCCGTCCTA
>SRR4252605_8323026___Sbg5
GATCACTAAATTGTACACCAAATTCA
>SRR4252606_4719978___Sbg5
ACCTGGTTTCACCTTCTCCCTCTTTCATA
>SRR4252611_5865171___Sbg5
GAGTGGTGGTGTACAACTTCTGATCACTAA
>SRR4252624_4419145___Sbg5
ATACTACCCCCCGTTAATTCCGTCCTA
>SRR4252608_4355842___Sbg5
TGTACAACTTCTGATCACTAAATTGT
>SRR4252614_6078305___Sbg5
AAGTGGTGGTGTACAACTTCTGATCACTAA
>SRR4252621_12043373___Sbg5
TATCGGCTGTGGGGACCAGCGTAGTACTGA
>SRR4252622_11033453___Sbg5
TCGAAAGGAGTAGGCTACATGCCGGCA
>SRR4252609_14864559___Sbg5
TACTACCCCCCGTTAATTCCGTCCTA
>SRR4252610_2009536___Sbg5
AGTGGTGGTGTACAACTTCTGATCATTA
>SRR4252611_1786668___Sbg5
CATACTACCCCCCGTTAATTCCGTCCTA
>SRR4252611_6744464___Sbg5
GAGTGGTGGTGTACAACTTCTGATCACTAA
>SRR4252606_9728744___Sbg5
GAGTGGTGGTGTACAACTTCTGATCACTAA
>SRR4252605_2199915___Sbg5
CCATACTACCCCCCGTTAATTCCGTCCTA
>SRR4252619_9227867___Sbg5
GCTGTGGGGACCAGCGTAGTTCTGACCA
>SRR4252619_6632910___Sbg5
AGTGCTGGTGTACAACTTCTGATCACTAA
>SRR4252619_9846488___Sbg5
AGTGCTGGTGTACAACTTCTGATCACTAA
>SRR4252606_3037174___Sbg5
ATACTACCTCCCGTTAATTCCGTCCTA
>SRR4252606_9954580___Sbg5
AGAAGGCATACGTCATTGTTGATAGTGA
>SRR4252611_12719739___Sbg5
GCTTTTCGAAAGGAGTAGGCTACATG
>SRR4252610_11916425___Sbg5
TTGATAGTGATCCGTCCGTCGGATGG
>SRR4252606_5494973___Sbg5
GAGTGGTGGTGTACAACTTCTGATCACTAA
>SRR4252606_2373706___Sbg5
TGAGTGGTGGTGTACAACTTCTGATCACTAA

>SRR4252610_12813439___Sbg5
TGAGTGGTGGTGTACAACTTCTGATCACTA
>SRR4252606_10424597___Sbg5
GCACCGGGTTTCACCTTCTCCCTCTTTCATA
>SRR4252609_9955292___Sbg5
TGATAGTGATCCGTCCGTCGGATGGGGAA
>SRR4252612_9143940___Sbg5
TTGATAGTGATCCGTCCGTCGGATGGGGAC
>SRR4252621_1554213___Sbg5
GAGTGGTGGTGTACAACTTCTGATCACTAA
>SRR4252606_5825489___Sbg5
TCCTCCTATTCACACACTTACACACCGA
>SRR4252611_14090966___Sbg5
TCATATCCATAATCGTCACTCACATCA
>SRR4252605_6524419___Sbg5
TCATATCCATTATCGTCACTCACATCATA
>SRR4252619_6565615___Sbg5
GTGGTGTACAACTTCTGATCAGTAAATT
>SRR4252608_713044___Sbg5
TTGATAGTGATCCGTCCGTCGGATGGGGAC
>SRR4252611_9001049___Sbg5
TTGATAGTGATCCGTCCGTCGGATGA
>SRR4252624_1597525___Sbg5
GGTTTCACCTTCTCCCTCTTTCATATCCA
>SRR4252624_10598624___Sbg5
CCAGGTTTCACCTTCTCCCTCTTTCATA
>SRR4252614_8769691___Sbg5
TTGATAGTGATCCGTCCGTCGGATGGGGAC
>SRR4252619_1377271___Sbg5
CCATACTATCCCCCGTTAATTCCGTCCTA
>SRR4252606_9790598___Sbg5
GGAGTGGTGGTGTACAACTTCTGATCACTA
>SRR4252612_4470985___Sbg5
TCGGCTGTGGGGACCAGCGTAGTACTGAT
>SRR4252626_7658614___Sbg5
ACCTGGGTTAAATCCCGAACTCCTCCGCA
>SRR4252614_5459479___Sbg5
TATGTAGAGAAGGCATATGTCATTGT
>SRR4252623_1944636___Sbg5
TGATAGTGATCCGTCCGTCGGATGGGGAC
>SRR4252606_7575228___Sbg5
CCCTCTTTCATATCCATAATCGTCACTCA
>SRR4252606_10154649___Sbg5
TGGTGTACAACTTCTGATCACTAAATTGT
>SRR4252605_9935050___Sbg5
CTCTTTCATATCCATAATCGTCACTCACA
>SRR4252611_8745320___Sbg5
CCATACTACCCCCCGTTAATTCCGTCCTA
>SRR4252619_6120889___Sbg5
CCATACTACCCCCCGTTAATTCCGTCCTA
>SRR4252623_11307053___Sbg5
ATATCCATAATCGTCACTCACATCAT
>SRR4252619_397762___Sbg5
CATCCATAATCGTCACTCACATCATA
>SRR4252611_5847765___Sbg5
GGGACCAGCGTAGTACTGACCATACTA
>SRR4252610_8620397___Sbg5
TTGATAGTGATCCGTCCGTCGGATGG
>SRR4252611_9292994___Sbg5
CCGGGTTTCACCTTCTCCCTCTTTCATA
>SRR4252612_3695502___Sbg5
ACACTCATCTCCCTATTCACACACTTACA
>SRR4252613_3139922___Sbg5
CATACTACCCCCCGTTAATTCCGTCCTA
>SRR4252605_12814851___Sbg5
GAAGTGGTGGTGTACAACTTCTGATCACTA
>SRR4252608_1585878___Sbg5
AAGTGGTGGTGTACAACTTCTGATCACTAA
>SRR4252610_6487950___Sbg5
AAGTGGTGGTGTACAACTTCTGATCACTA

>SRR4252612_7443200___Sbg5
GAGTGGTGGTGTACAACTTCTGATCACTA
>SRR4252619_14473896___Sbg5
ATATCCATAATCGTCACTCACATCATA
>SRR4252611_3671720___Sbg5
AGTGGTGGTGTACAACTTCTGATCACTAA
>SRR4252606_7657341___Sbg5
TTCATATCCATAATCGTCACTCGCATCAT
>SRR4252607_13291647___Sbg5
TATGTAGAGAAGGCATATGTCATTGTT
>SRR4252606_623932___Sbg5
GAGTGGTGGTGTACAACTTCTGATCACTAA
>SRR4252609_13744100___Sbg5
GAGTGGTGGTGTACAACTTCTGATCACTAA
>SRR4252612_8776501___Sbg5
AATTCCGCCCTATGGCGGCAGCCGCTCGGTA
>SRR4252623_3836537___Sbg5
CTTCTCCCTCTTTCATATCCATAATC
>SRR4252622_4653888___Sbg5
TCGGCTGTGGGGACCAGCATAGTACTGA
>SRR4252622_8850294___Sbg5
GAGTGGTGGTGTACAACTTCTGATCACTAA
>SRR4252610_6807558___Sbg5
GAGTGGTGGTGTACAACTTCTGATCACTAA
>SRR4252616_6170781___Sbg5
TAGAAAACCGGGGTTATCGGCTGTGGGG
>SRR4252626_6159794___Sbg5
GAATGGTGGTGTACAACTTCTGATCACTAA
>SRR4252608_12777210___Sbg5
ATGGTGGTGTACAACTTCTGATCACTAAA
>SRR4252626_211945___Sbg5
GAGTGGTGGTGTACAACTTCTGATCACTAA
>SRR4252608_4223458___Sbg5
TACAACTTCTGATCACTAAATTGTGCAT
>SRR4252612_8981656___Sbg5
TACAACTTCTGATCACTAAATTGTGCAT
>SRR4252606_8819797___Sbg5
GAGTGGTGGTGTACAACTTCTGATCACTAA
>SRR4252611_6950906___Sbg5
TTGATAGTGATCCGTCCGTCGGATGGGGAC
>SRR4252611_9308335___Sbg5
TTGATAGTGATCCGTCCGTCGGATGGGG
>SRR4252611_484806___Sbg5
TCCGTCCGTCGGATGGGGACGTTATGC
>SRR4252605_14647419___Sbg5
GGAGTGGTGGTGTACAACTTCTGATCACT
>SRR4252606_466291___Sbg5
TCCGTCCGTCGGATGGGGACGTTAAGC
>SRR4252608_3608125___Sbg5
TATGTAGAGAAGGCATATGTCATTGTTGA
>SRR4252605_12558776___Sbg5
ACATACTACCCCCCGTTAATTCCGTCCTA
>SRR4252606_5842063___Sbg5
ACATACTACCCCCCGTTAATTCCGTCCTA
>SRR4252618_7574667___Sbg5
CATATCCATAATCGTCACTCACATCATAC
>SRR4252606_4350009___Sbg5
GAGTGGTGGTGTACAACTTCTGATCACTAA
>SRR4252611_8594637___Sbg5
TTGATAGTGATCCGTCCGTCGGATGGGGAC
>SRR4252622_4087991___Sbg5
AGTGGTGTACAACTTCTGATCACTAA
>SRR4252611_8204808___Sbg5
TTGGCCCACATAGGGCTGTTGAGCTATAGA
>SRR4252606_6328182___Sbg5
TATCCATAATCGTCACTCACATCATA
>SRR4252625_7183032___Sbg5
GGAGTGGTGGTGTACAACTTCTGATCACT
>SRR4252610_9516481___Sbg5
GAGTGGTGGTGTACAACTTCTGATCACTAA

>SRR4252613_9796245___Sbg5
TCATATCCATAATCGTCACTCACATCAT
>SRR4252605_3517553___Sbg5
TTCATATCCATAATCGTCACTCACATCATAC
>SRR4252606_8809795___Sbg5
GCTGTGGGACCAGCGTAGTACTGACA
>SRR4252612_5222017___Sbg5
TATGTAGAGAAGGCATATGTCATTGTTGA
>SRR4252619_14286116___Sbg5
TGATCCGTCCGTCGGATGGGACGTTA
>SRR4252606_9206099___Sbg5
CACCAAATTCACCTGGGTTAAATCCA
>SRR4252612_3291134___Sbg5
TCGGCTGTGGGGACCAGCATAGTACTG
>SRR4252611_10108949___Sbg5
CCATACTACCCCCCGTTAATTCCGTCCTA
>SRR4252611_8526784___Sbg5
CCATACTACCCCCCGTTAATTCCGTCCTA
>SRR4252605_8640111___Sbg5
GCACACACAATACAACACTATACACACA
>SRR4252606_1683212___Sbg5
GCACACACAATACAACACTATACACACA
>SRR4252620_7852274___Sbg5
GCACACACAATACAACACTATACACACA
>SRR4252608_9286347___Sbg5
GTGGTGGTGTACAACTTCTGATCACTAA
>SRR4252624_3947840___Sbg5
TTTGTTGATAGTGATCCGTCCGTCGGATG
>SRR4252619_1816439___Sbg5
ATTCATATCCATAATCGTCACTCACATCA
>SRR4252621_5730320___Sbg5
ATTCATATCCATAATCGTCACTCACATCATA
>SRR4252610_4213447___Sbg5
AAGTGGTGGTGTACAACTTCTGATCACTAA
>SRR4252611_3494158___Sbg5
GAGTGGTGGTGTACAACTTCTGATCACTAA
>SRR4252610_10558981___Sbg5
TGTACAACTTCTGATCACTAAATTGT
>SRR4252614_425873___Sbg5
GGTGTACAACTTCTGATCACTAAATTGTGCA
>SRR4252625_8140250___Sbg5
GGTAAGCCTTGGCCCACATAGGGCTGT
>SRR4252621_11006230___Sbg5
GACCATACTACCCCCCGTTAATTCCG
>SRR4252615_11573165___Sbg5
CTTGGCCCACATAGGGCTGTTGAGCTAT
>SRR4252621_9117444___Sbg5
CCATACTACCCCCCGTTAATTCCGTCCTA
>SRR4252611_7927358___Sbg5
ACGTCATTGTTGATAGTGATCCGTCCGT
>SRR4252608_11005634___Sbg5
TCCGTCCGTCGGATGGGACGTTAGG
>SRR4252605_7953421___Sbg5
GCATACTACCCCCCGTTAATTCCGTCCTA
>SRR4252611_13508639___Sbg5
TTGATAGTGATCCGTCCGTCGGATGGGG
>SRR4252606_4094336___Sbg5
GAGTGGTGGTGTACAACTTCTGATCACTAA
>SRR4252611_7134825___Sbg5
TCATATCCATAATCGTCACTCACATCATA
>SRR4252614_5703652___Sbg5
CCACACACACAATACAACACTATACAC
>SRR4252621_9279868___Sbg5
AAGCCGTCGGACCCCTTGGTGTTTTTCGA
>SRR4252612_9102712___Sbg5
GAGTAATTAATTAAAATTTCGAATCCTG
>SRR4252606_4637910___Sbg5
GAGTGGTGGTGTACAACTTCTGATCACTAA
>SRR4252611_11132012___Sbg5
CCATACTACCCCCCGTTAATTCCGTCCTA

>SRR4252606_5422675___Sbg5
ACACACACTCATCCTCCTATCCACACA
>SRR4252608_11693701___Sbg5
TATCGGCTGTGGGGACCAGCGTAGTACT
>SRR4252614_579913___Sbg5
TACAACTTCTGATCACTAAACTGTACAC
>SRR4252611_5603188___Sbg5
TTGATAGTGATCCGTCCGTCGGATGG
>SRR4252614_2783439___Sbg5
ATATCCATAATCGTCACTCACATCAC
>SRR4252618_2115532___Sbg5
GTGTACAACTTCTGATCACTAAATTG
>SRR4252610_6841479___Sbg5
TGCTTTTCGAAAGGAGTAGGCTACAT
>SRR4252610_2290420___Sbg5
TTCATATCCATAATCGTCACTCACATCAT
>SRR4252606_1747216___Sbg5
CCATACTATCCCCCGTTAATTCCGTCCTA
>SRR4252612_1495069___Sbg5
CATATTACCCCCCGTTAATTCCGTCCTA
>SRR4252606_3535228___Sbg5
GAGTGGTGGTGTACAACTTCTGATCACTAA
>SRR4252611_8589117___Sbg5
CACTCATCACCCTATCCACACACTTACA
>SRR4252608_600232___Sbg5
CCACACCACACACACAATACAACACTA
>SRR4252609_12975422___Sbg5
TTGATAGTGATCCGTCCGTCGGATGGGG
>SRR4252619_1066827___Sbg5
CCATACTACCCCCCGTTAATTCCGTCCTA
>SRR4252606_4358070___Sbg5
CTCTTTCATATCCATAATCGTCACTCACATC
>SRR4252611_8746396___Sbg5
TTGATAGTGATCCGTCCGTCGGATGGGGA
>SRR4252610_10376656___Sbg5
ACACTCATCTCCCTATTCACACACTTACA
>SRR4252605_3652439___Sbg5
TGGTGTACAACTTCTGATCACTAAATTGT
>SRR4252615_10370920___Sbg5
TATGTCATTGTTGATAGTGATCCGTCCGC
>SRR4252611_10048078___Sbg5
CCATAATCGTCACTCACATCATACCA
>SRR4252611_13079403___Sbg5
ATCCATTAGCTGGTCTAGAAAACCGGG
>SRR4252622_8281990___Sbg5
TTGATAGTGATCCGTCCGTCGGATGGGG
>SRR4252621_11509346___Sbg5
GCCTTGGCCCACATCGGGCTGTTGAGCTATA
>SRR4252621_11298032___Sbg5
GCCGGTCCCCTTGGTGCTTTTCGAAAGGA
>SRR4252619_577450___Sbg5
TCTAGAAAACCGGGGTTATCGGCTGTGG
>SRR4252611_1343995___Sbg5
TTGATAGTGATCCGTCCGTCGGATGGGG
>SRR4252606_3518542___Sbg5
GGAGTGGTGGTGTACAACTTCTGATCACT
>SRR4252606_9212257___Sbg5
TTCATATCCATAATCGTCACTCACATCAT
>SRR4252611_3291377___Sbg5
CACACTTACACACCGATCCATTAACT
>SRR4252614_4910772___Sbg5
TAGAAAACCGGGGCTATCGGCTGTGGGG
>SRR4252606_6531387___Sbg5
GAAATGGTGGTGTACAACTTCTGATCACTA
>SRR4252605_3954295___Sbg5
AAATGGTGGTGTACAACTTCTGATCACTA
>SRR4252605_7578469___Sbg5
AAATGGTGGTGTACAACTTCTGATCACTAA
>SRR4252608_5974578___Sbg5
AAATGGTGGTGTACAACTTCTGATCACTAA

>SRR4252619_12242754___Sbg5
ATGGTGGTGTACAACTTCTGATCACTAA
>SRR4252606_1348941___Sbg5
TCTCCCTCTTTCATATCCATAATCGTCA
>SRR4252610_2302930___Sbg5
CAGTGGTGGTGTACAACTTCTGATCACTAA
>SRR4252608_6784420___Sbg5
TATCCATAATCGTCACTCACATCATA
>SRR4252619_6026375___Sbg5
TGGTGGTGTACAACTTCTGATCACTAA
>SRR4252612_681021___Sbg5
TCTTTCATATCCATAATCGTCACTCACATC
>SRR4252609_2579099___Sbg5
TTGATAGTGATCCGTCCGTCGGATGGGAC
>SRR4252610_6841105___Sbg5
GGAGTGGTGGTGTACAACTTCTGATCACTAA
>SRR4252623_6309933___Sbg5
GAGTGGTGGTGTACAACTTCTGATCACTAA
>SRR4252612_1058952___Sbg5
TTGATAGTGATCCGTCCGTCGGATGG
>SRR4252606_9242892___Sbg5
ATCCGCACACATACACAATGATCAAATA
>SRR4252611_7998079___Sbg5
AATTCCGCCCTATGGCGGCAGCCGCTCGGTA
>SRR4252612_2312605___Sbg5
CATACTACCCCCCGTTAATTCCGTCCTA
>SRR4252606_8398879___Sbg5
GGGAGTGGTGGTGTACAACTTCTGATCAC
>SRR4252606_7875731___Sbg5
CACTTACACACCAATCCATTAGCTGG
>SRR4252605_11609981___Sbg5
TGGTGTACAACTTCTGATCACTAAATTGTAC
>SRR4252623_7969441___Sbg5
GAGTGGTGGTGTACAACTTCTGATCACTAA
>SRR4252620_7439___Sbg5
CCGCTCGGTAAGCCTTGGCCCACATAGGT
>SRR4252608_4037269___Sbg5
GAGTAGGCTACATGCCGGCACCGGGTTTCA
>SRR4252623_911059___Sbg5
GTGGTGGTGTACAACTTCTGATCACTAA
>SRR4252605_1458846 7___Sbg5
ACACTCATCCTCCTATTCACACACTTACA
>SRR4252610_8580077___Sbg5
GAGTGCTGGTGTACAACTTCTGATCACTAA
>SRR4252610_5420730___Sbg5
CTCTTTCATATCCATAATCGTCACTCACAT
>SRR4252613_5304976___Sbg5
TACAACTTCTGATCACTAAATTGTGCAC
>SRR4252623_10944663___Sbg5
AAGTGGTGGTGTACAACTTCTGATCACTAA
>SRR4252609_543822___Sbg5
GTGATCCGTCCGTCGGATGGGGACGTTA
>SRR4252605_762385___Sbg5
TCGGCTGTGGGGACCAGCGTAGTACTGACA
>SRR4252622_49327___Sbg5
CACCACACACACAATACAACACTATA
>SRR4252612_366727___Sbg5
ACACTCATCTCCCTATTCACACACTTACA
>SRR4252616_7932898___Sbg5
TGTCATTGTTGATAGTGATCCGTCCGTCG
>SRR4252609_13116083___Sbg5
CCATACTGCCCCCCGTTAATTCCGTCCTA
>SRR4252605_9294226___Sbg5
TTTCATATCCATAATCGTCACTCACATCATA
>SRR4252610_5030304___Sbg5
TCGGCTGTGGGGACCAGCGTAGTACTGA
>SRR4252626_9636568___Sbg5
GAGTGGTGGTGTACAACTTCTGATCACTAA
>SRR4252605_2926535___Sbg5
TACAACTTCTGATCACTAAATTGTGCA

>SRR4252611_7136829___Sbg5
CCTTGGCCCACATCGGGCTGTTGAGCTAT
>SRR4252605_9618618___Sbg5
TACAACTTCTGATCACTAAATTGTGC
>SRR4252621_11140630___Sbg5
CCGGTCCCCTTGGTGCTTTTCGAAAGGA
>SRR4252606_6696035___Sbg5
CAAAATTTCGAATCCTGAGACTAGGAGGA
>SRR4252623_12714296___Sbg5
CCGGTCCCCTTGGTGCTTTTCGAAAGGA
>SRR4252611_189461___Sbg5
CACCGGGTTTCACCTTCTCCCTCTTTCATA
>SRR4252610_1353761___Sbg5
TCCATAATCGTCACTCACATCATACCAC
>SRR4252619_5465063___Sbg5
TACAACTTCTGATCACTAAATTGTGCAC
>SRR4252614_3900954___Sbg5
TCATTGTTGATAGTGATCCGTCCGTCG
>SRR4252616_12811790___Sbg5
GAGTAGGCTACATGCCGGCACCGGGTTTCAC
>SRR4252621_9531873___Sbg5
GTTTCACCTTCTCCCTCTTTCATATCCA
>SRR4252610_902542___Sbg5
ACACTCATCCCCCTATCCACACACTTACA
>SRR4252605_8227053___Sbg5
TGGTGTACAACTTCTGATCACTAAATTGT
>SRR4252611_12818892___Sbg5
ACGGGTTTCACCTTCTCCCTCTTTCATA
>SRR4252624_2806639___Sbg5
ACGGGTTTCACCTTCTCCCTCTTTCATA
>SRR4252606_3734558___Sbg5
GAGTGGTGGTGTACAACTTCTGATCACTAA
>SRR4252612_4594761___Sbg5
GTGGTGGTGTACAACTTCTGATCACTAAA
>SRR4252606_9255825___Sbg5
GTCATATCCATAATCGTCACTCACATCATA
>SRR4252610_12547055___Sbg5
ACACTCATCCTCCTATCCGCACACATA
>SRR4252620_6384995___Sbg5
TCCTCCTATCCGCACACATACACAATGA
>SRR4252610_10089255___Sbg5
TCCTATCCGCACACATACACAATGATCA
>SRR4252612_2022991___Sbg5
CCGCACACATACACAATGATCAAATA
>SRR4252606_9533547___Sbg5
CTTGGCCCACATCGGGCTGTTGAGCT
>SRR4252611_183831___Sbg5
GTGGTGTACAACTTCTGATCACTAAATTGT
>SRR4252606_8793151___Sbg5
CCTCCTATCCGCACACATACACAATGA
>SRR4252611_5867353___Sbg5
ATCCGCACACATACACAATGATCAAATA
>SRR4252607_11292041___Sbg5
CCGCACACATACACAATGATCAAATA
>SRR4252610_10826148___Sbg5
TGGTGCTTTTCGAAAGGAGTAGGCTACA
>SRR4252605_5133836___Sbg5
AGAGTGGTGGTGTACAACTTCTGATCACTAA
>SRR4252606_361965___Sbg5
AGAGTGGTGGTGTACAACTTCTGATCACTA
>SRR4252616_7958673___Sbg5
AGAGTGGTGGTGTACAACTTCTGATCACTA
>SRR4252609_4399234___Sbg5
AGTGGTGGTGTACAACTTCTGATCACTAA
>SRR4252611_12803250___Sbg5
TCCGTCCGTCGGATGGGGACGTTAAGCCT
>SRR4252620_1290381___Sbg5
TGTTGATAGTGATCCGTCCGTCGGATGG
>SRR4252611_6883527___Sbg5
AATTCCGTCCTATGGCGGCAGCCGCTCGGTA

>SRR4252610_3817621___Sbg5
TCGAATCCTGAGACTAGGAGGAGTGGTGG
>SRR4252605_2569763___Sbg5
ACTTCTGATCACTAAATTGTACACCA
>SRR4252620_4184933___Sbg5
GTGATCCGTCCGTCGGATGGGGACGTT
>SRR4252616_15177501___Sbg5
ACTCATCCTCCTATCCGCACACATAC
>SRR4252612_265993___Sbg5
ATCCGCACACATACACAATGATCAAATA
>SRR4252606_1852371___Sbg5
GAGTGGTGGTGTACAACTTCTGATCACTAA
>SRR4252606_5929674___Sbg5
GAGTGGTGGTGTACAACTTCTGATCACTAA
>SRR4252610_13125596___Sbg5
GAGTGGTGGTGTACAACTTCTGATCACTAA
>SRR4252610_4762923___Sbg5
ACCATTCTACCCCCCGTTAATTCCGTCCTA
>SRR4252615_10038526___Sbg5
GGAGTGGTGGTGTACAACTTCTGATCACTA
>SRR4252613_9069135___Sbg5
AGTGGTGGTGTACAACTTCTGATCACTAA
>SRR4252610_3639486___Sbg5
AAGTGGTGGTGTACAACTTCTGATCACTAA
>SRR4252611_4935850___Sbg5
AAGTGGTGGTGTACAACTTCTGATCACTA
>SRR4252625_9785673___Sbg5
CACACACTTACACACCGATCCATTAACTG
>SRR4252606_3665770___Sbg5
CCTCCTATCCGCACACATACACAATGAT
>SRR4252606_2114520___Sbg5
TCCTCCTATCCGCACACATACACAATGA
>SRR4252607_9269908___Sbg5
CGGATTTCACCTTCTCCCTCTTTCATA
>SRR4252620_5042881___Sbg5
ATTTCACCTTCTCCCTCTTTCATATCCA
>SRR4252611_981293___Sbg5
GGGCTACATGCCGGCACCGGGTTTCAC
>SRR4252605_4078326___Sbg5
GCTGTGGGGACCAGCGTAGTACTGACT
>SRR4252619_937956___Sbg5
TCGGTAAGCCTTGGCCCACATAGGGCTGTTG
>SRR4252621_10345663___Sbg5
GAGTGGTGATGTACAACTTCTGATCACTAA
>SRR4252616_336873___Sbg5
TTAAATCCCGAACTCCTCCGCAATGACT
>SRR4252605_8325531___Sbg5
TTTTTCGAAAGGAGTAGGCTACATGCCGGT
>SRR4252612_7129548___Sbg5
TCGGCTGTGGGGACCAGCGTAGTACT
>SRR4252605_15176080___Sbg5
TGGTGTACAACTTCTGATCACTAAATTGT
>SRR4252610_4923167___Sbg5
GAGAAGGCATACGTCATTGTTGATAGTGA
>SRR4252612_4720929___Sbg5
CATACTACCCCCCGTTAATTCCGTCCTA
>SRR4252606_31287___Sbg5
TGGTGGTGTACAACTTCTGATCACTAA
>SRR4252608_11209309___Sbg5
TGGTGTACAACTTCTGATCACTAAATTGT
>SRR4252611_11288395___Sbg5
GGGAGTGGTGGTGTACAACTTCTGATCAC
>SRR4252608_1300674___Sbg5
CTCCCTCTTTCATATCCATAATCGTCA
>SRR4252611_9337479___Sbg5
ACAGCCGCTCGGTAAGCCTTGGCCCACA
>SRR4252616_9304693___Sbg5
TAAATCCCGAACTCCTCCGCAATGACC
>SRR4252610_9994796___Sbg5
GAGTGGTGGTGTACAACTTCTGATCACTAA

>SRR4252612_8199260___Sbg5
GAGTGGTGGTGTACAACTTCTGATCACTAA
>SRR4252610_1900779___Sbg5
CACACTCATCCTCCTATCCGCACACATA
>SRR4252605_13018544___Sbg5
TGTACAACTTCTGATCACTAAATTGTGC
>SRR4252611_15170924___Sbg5
GATCCGTCCGTCGGATGGGGACGTTA
>SRR4252610_2501162___Sbg5
TCCTCCTATCCGCACACATACACAATGA
>SRR4252626_374964___Sbg5
TCGGGTTTCACCTTCTCCCTCTTTCATA
>SRR4252615_14190242___Sbg5
CTTGGCCCACATAGGGCTGTTGAGCTAT
>SRR4252611_1032188___Sbg5
TTGATAGTGATCCGTCCGTCGGATGGGGAC
>SRR4252605_13017037___Sbg5
CCGGGGTTATCGGCTGTGGGGACCAGCGTA
>SRR4252610_11282903___Sbg5
GAGTGGTGGTGTACAACTTCTGATCACTAA
>SRR4252621_3985106___Sbg5
TCCGTCCTATGGCGGCAGCCGCTCGGTA
>SRR4252611_10713375___Sbg5
ACTCATCTCCCTATTCACACACTTACACA
>SRR4252618_7823833___Sbg5
TCGGCTGTGGGGACCAGCATAGTACTGA
>SRR4252606_8014900___Sbg5
GAGTGGTGGTGTACAACTTCTGATCACTAA
>SRR4252606_6824064___Sbg5
CCACACTACCCCCCGTTAATTCCGTCCTA
>SRR4252605_6951602___Sbg5
TGGTGGTGTACAACTTCTGATCACTAA
>SRR4252615_11377552___Sbg5
AATTCCGCCCTATGGCGGCAGCCGCTCGGTA
>SRR4252611_13824731___Sbg5
AGTGGTGGTGTACAACTTCTGATCACTAA
>SRR4252612_4086069___Sbg5
TATCGGCTGTGGGGACCAGCGTAGTACTA
>SRR4252612_2872562___Sbg5
TCATATCTATAATCGTCACTCACATCAT
>SRR4252619_7783233___Sbg5
TCATATCTATAATCGTCACTCACATCAT
>SRR4252612_7083572___Sbg5
AGTGGTGGTGTACAACTTCTGATCACTAA
>SRR4252621_10356124___Sbg5
GAGTGGTGGTGTACAACTTCTGATCACTAA
>SRR4252616_13733917___Sbg5
AGAAGGCATATGTCATTGTTGATAGTGA
>SRR4252613_4835564___Sbg5
TCCTCCTATCCGCACACATACACAATGA
>SRR4252610_1819253___Sbg5
ATCCGCACACATACACAATGATCAAATA
>SRR4252610_3749325___Sbg5
GAGTGGTGGTGTACAACTTCTGATCACTAA
>SRR4252605_13082265___Sbg5
TGGTGTACAACTTCTGATCACTAAATTGT
>SRR4252615_1633041___Sbg5
TGATCCGTCCGTCGGATGGGGACGTT
>SRR4252609_12413381___Sbg5
CATATCCATAATCGTCACTCACATCATA
>SRR4252605_8001337___Sbg5
CACTACACACACAATACAACACTATA
>SRR4252606_1994397___Sbg5
TCCTCCTATCCGCACACATACACAATGA
>SRR4252607_4306926___Sbg5
CCGCACACATACACAATGATCAAATA
>SRR4252605_10430900___Sbg5
TGCTTTTCGAAAGGAGTAGGCTACATGC
>SRR4252610_12861546___Sbg5
GAGTGGTGGTGTACAACTTCTGATCACTAA

>SRR4252610_8709820___Sbg5
GAGTGGTGGTGTACAACTTCTGATCACTAA
>SRR4252610_1371097___Sbg5
TCCTCCTATCCGCACACATACACAATGA
>SRR4252609_12156374___Sbg5
TTCATATCCATAATCGTCACTCACATCATA
>SRR4252605_13327495___Sbg5
GGAGTGGTGGTGTACAACTTCTGATCACTAA
>SRR4252610_1112956___Sbg5
GAGTGGTGGTGTACAACTTCTGATCACTAA
>SRR4252612_4479775___Sbg5
TTGATAGTGATCCGTCCGTCGGATGGGGAA
>SRR4252608_6518187___Sbg5
TAGAAAACCGGGGTTATCGGCTGTGGG
>SRR4252611_7243919___Sbg5
TGGCCCACATCGGGCTGTTGAGCTATAGA
>SRR4252611_1028119___Sbg5
TCGGCTGTGGGGACCAGCATAGTACTGA
>SRR4252607_1609962___Sbg5
CCGGTCCCCTTGGTGCTTTTCGAAAGGA
>SRR4252610_1971491___Sbg5
ATACTACCCCCCGTTAATTCCGTCCTA
>SRR4252619_6057439___Sbg5
GCAGTGGTGGTGTACAACTTCTGATCACTAA
>SRR4252610_3971118___Sbg5
TGGTGTACAACTTCTGATCACTAAATTG
>SRR4252624_2896420___Sbg5
TCGGCTGTGGGGACCAGCGTAGTACTGA
>SRR4252617_4601971___Sbg5
TCCATAATCGTCACTCACATCATACCACAC
>SRR4252610_1768548___Sbg5
TGGTCTAGAAAACCGGGGCTATCGGCTGT
>SRR4252610_9943243___Sbg5
TCGGCTGTGGGGACCAGCATAGTACTGA
>SRR4252615_10786434___Sbg5
TCGGCTGTGGGGACCAGCATAGTACTG
>SRR4252616_8983682___Sbg5
TCGGCTGTGGGGACCAGCATAGTACTG
>SRR4252613_9945692___Sbg5
GAGTGGTGGTGTACAACTTCTGATCACTAA
>SRR4252612_6052657___Sbg5
GAGTGGTGGTGTACAACTTCTGATCACTAA
>SRR4252605_5885046___Sbg5
TTGATAGTGATCCGTCCGTCGGATGGGGACT
>SRR4252605_7069901___Sbg5
ATCCGCACACATACACAATGATCAAATA
>SRR4252606_8075163___Sbg5
ATCCGCACACATACACAATGATCAAATA
>SRR4252610_5204036___Sbg5
GAGTGGTGGTGTACAACTTCTGATCACTAA
>SRR4252611_12534720___Sbg5
CCTCCTATCCGCACACATACACAATGA
>SRR4252610_9493175___Sbg5
ACAATACAACACTATACACACACTCAT
>SRR4252606_7787615___Sbg5
CCATACTACCCCCCGTTAATTCCGTCCTA
>SRR4252618_956878___Sbg5
TATCGGCTGTGGGGACCAGCGTAGTAC
>SRR4252609_7200650___Sbg5
GGTTAAATCCCGAACTCCTCCGCAATG
>SRR4252606_1070695___Sbg5
GGAGTGGTGGTGTACAACTTCTGATCACTAA
>SRR4252606_6779325___Sbg5
TCCTCCTATCCGCACACATACACAATGA
>SRR4252621_9096201___Sbg5
GAGTGGTGGTGTACAACTTCTGATCACTAA
>SRR4252609_15416699___Sbg5
GAGTGGTGGTGTACAACTTCTGATCACTAA
>SRR4252606_4641628___Sbg5
GAGTCGTGGTGTACAACTTCTGATCACTAA

```
>SRR4252619_5909134___Sbg5
GTGGTGTACAACTTCTGATCACTAAATTG
>SRR4252615_11045148___Sbg5
TAGAAAACCGGGGCTATCGGCTGTGGG
>SRR4252621_10962979___Sbg5
GAGTGGTGGTGTACAACTTCTGATCACTAA
>SRR4252607_14352681___Sbg5
TACAACTTCTGATCACTAAATTGTACACT
>SRR4252605_11239887___Sbg5
GAGTGGTGGTGTACAACTTCTGATCG
>SRR4252607_1631948___Sbg5
GTGTACAACTTCTGATCGCTAAATTG
>SRR4252613_2318461___Sbg5
TCCATAATCGTCACTCACATCATACCAC
>SRR4252620_1699269___Sbg5
TCCTCCGCAATGACTATGTAGAGAAGGCATA
>SRR4252619_12090955___Sbg5
AAGCCGTCGGACCCCTTGGTGTTTTTCGA
>SRR4252612_672865___Sbg5
AGGCATATGTCATTGTTGATAGTGATCCGT
>SRR4252610_2139704___Sbg5
TCCGCACACATACACAATGATCAAATA
>SRR4252614_2006119___Sbg5
GTGTACAACTTCTGATCACTAAATTT
>SRR4252608_11359987___Sbg5
GAGTGGTGGTGTACAACTTCTGATCACTAA
>SRR4252610_6279897___Sbg5
TAATTCCGTCCTATGGCGGCAGCCGCTCG
>SRR4252611_5006803___Sbg5
TGTACAACTTCTGATCACTAAATTGTGC
>SRR4252610_440876___Sbg5
CCTCCTATCCGCACACATACACAATGA
>SRR4252611_7094170___Sbg5
ATCCGCACACATACACAATGATCAAATA
>SRR4252614_5468443___Sbg5
GAGTGGTGGTGTACAACTTCTGATCACTAA
>SRR4252612_7551790___Sbg5
GGTGGTGTACAACTTCTGATCACTAA
>SRR4252611_7148710___Sbg5
AGTGATCCGTCCGTCGGATGGGGACGTTA
>SRR4252609_9597697___Sbg5
CTTGGCCCACATCGGGCTGTTGAGCT
>SRR4252605_9966760___Sbg5
GAGAAGGCATATGTCATTGTTGATAGTGA
>SRR4252611_10655090___Sbg5
CCTATCCGCACACATACACAATGATCAA
>SRR4252612_6460073___Sbg5
AGAAGGCATACGTCATTGTTGATAGTGA
>SRR4252611_9370326___Sbg5
GTGGTGGTGTACAACTTCTGATCACTAA
>SRR4252611_6907088___Sbg5
CCTCCTATCCGCACACATACACAATGA
>SRR4252611_3502020___Sbg5
TGGTGTACAACTTCTGATCACTAAAT
>SRR4252611_532930___Sbg5
TTCCGCCCTATGGCGGCAGCCGCTCGGTA
>SRR4252611_162471___Sbg5
CCGGGTTTCACCTTCTCCCTCTTTCATA
>SRR4252605_12336674___Sbg5
GTGTACAACTTCTGATCACTAAATTGTGC
>SRR4252611_666469___Sbg5
TAGTACTGACCATACTACCCCCTGTTA
>SRR4252610_5506078___Sbg5
ACACACACTCATCCTCCTATCCGCACA
>SRR4252613_3485927___Sbg5
TCCTCCTATCCGCACACATACACAATGA
>SRR4252620_1158132___Sbg5
TTATCGGCTGTGGGGACCAGCGTAGT
>SRR4252606_6919674___Sbg5
AGAAGGCATATGTCATTGTTGATAGTGA
```

>SRR4252622_7510437___Sbg5
TGATAGTGATCCGTCCGTCGGATGGGGACGT
>SRR4252608_8094876___Sbg5
TGGTGTACAACTTCTGATCACTAAATTGT
>SRR4252610_4319908___Sbg5
CATATCCATAATCGTCACTCACATCATA
>SRR4252612_8214166___Sbg5
ATATCCATAATCGTCACTCACATCATA
>SRR4252610_299357___Sbg5
ACACTCATCCCCCTATTCACACACTTACA
>SRR4252610_7708984___Sbg5
GAGTGGTGGTGTACAACTTCTGATCACTAA
>SRR4252610_3059447___Sbg5
GAGTGGTGGTGTACAACTTCTGATCACTAA
>SRR4252612_411583___Sbg5
CACCGGGTCTCACCTTCTCCCTCTTTCATA
>SRR4252611_12022014___Sbg5
ACCGGGTCTCACCTTCTCCCTCTTTCATA
>SRR4252612_654095___Sbg5
TTGTTGATAGTGATCCGTCCGTCGGATG
>SRR4252610_2003581___Sbg5
CATATTCATAATCGTCACTCACATCATA
>SRR4252607_11902080___Sbg5
ACACAATTACACACCAATCCATTAGCTG
>SRR4252612_1718565___Sbg5
CTAGAAAACCGGGGCTATCGGCTGTT
>SRR4252621_3988068___Sbg5
GAGGGGTGGTGTACAACTTCTGATCACTAA
>SRR4252611_13998921___Sbg5
TCCGTCCGTCGGATGGGGACGTTAAGT
>SRR4252610_12004871___Sbg5
TCCTCCTATCCGCACACATACACAATGA
>SRR4252605_13956556___Sbg5
GTGTACAACTTCTGATCACTAAATTGT
>SRR4252618_10675929___Sbg5
TGTTGATAGTGATCCGTCCGTCGGATG
>SRR4252612_7886554___Sbg5
ACTCATCCTCCTATCCGCACACATACAC
>SRR4252606_1095366___Sbg5
CACTCATCCTCCTATCCGCACACATACA
>SRR4252613_4061819___Sbg5
CCTCCTATCCGCACACATACACAATGA
>SRR4252606_1256946___Sbg5
GAGTGGTGGTGTACAACTTCTGATCACTAA
>SRR4252612_3736052___Sbg5
GAGTGGTGGTGTACAACTTCTGATCACT
>SRR4252618_8152284___Sbg5
TAAATTGTACACCAATTTCACCTGGGC
>SRR4252619_9803666___Sbg5
TCATATCCATAATCGTCACTCACATCA
>SRR4252605_5491033___Sbg5
CTATCCGCACACATACACAATGATCAAA
>SRR4252613_10542980___Sbg5
CCTCCTATCCGCACACATACACAATGA
>SRR4252606_1936287___Sbg5
AAGTGGTGGTGTACAACTTCTGATCACTAA
>SRR4252610_10481004___Sbg5
AAGTGGTGGTGTACAACTTCTGATCACTAA
>SRR4252606_2450536___Sbg5
CCTCCTATCCGCACACATACACAATGA
>SRR4252615_10724663___Sbg5
TGGTGGTGTACAACTTCTGATCACTAAATTG
>SRR4252616_663829___Sbg5
GATCAAATAGCTGGTCTAGAAAACCG
>SRR4252612_658521___Sbg5
TCCTATCCGCACACATACACAATGATCA
>SRR4252611_5637780___Sbg5
ATCCGCACACATACACAATGATCAAATA
>SRR4252617_163382___Sbg5
GTGGTGTACAACTTCTGATCACTAAAT

>SRR4252606_4312846___Sbg5
TCGGATGGGGACGTTAAGCCGTCGGACT
>SRR4252611_399900___Sbg5
CGCACACATACACAATGATCAAATAGCT
>SRR4252616_5035509___Sbg5
TAGAAAACCGGGGTTATCGGCTGTGGGG
>SRR4252611_303034___Sbg5
AGAGGTGGTGTACAACTTCTGATCACTAA
>SRR4252609_11413902___Sbg5
CTCCCTCTTTCATATCCATAATCGTCA
>SRR4252610_11829139___Sbg5
TACACACACTCATCCTCCTATCCGCACA
>SRR4252610_389267___Sbg5
ACACTCATCCTCCTATCCGCACACATA
>SRR4252606_3840003___Sbg5
GGAGTGGTGGTGTACAACTTCTGATCACTA
>SRR4252621_6211654___Sbg5
TGGTGTACAACTTCTGATCACTAAATTGTG
>SRR4252612_6143381___Sbg5
GTGATCCGTCCGTCGGATGGGGACGTTA
>SRR4252615_13740078___Sbg5
TCTAGAAAACCGGGGTTATCGGCTGTGG
>SRR4252605_11865231___Sbg5
ACACACACAATACAACACTATACACACA
>SRR4252612_8104722___Sbg5
CATCCTCCTATCCGCACACATACACAATGA
>SRR4252618_773928___Sbg5
TGGTGTACAACTTCTGATCACTAAATTGT
>SRR4252613_6174082___Sbg5
TGCTTTTCGAAAGGAGTAGGCTACATGC
>SRR4252622_8159965___Sbg5
TTATCGGCTGTGGGGATCAGCGTAGTACT
>SRR4252605_4206912___Sbg5
CTCCCTCTTTCATATCCATAATCGTC
>SRR4252611_632046___Sbg5
TTCATATCCATAATCGTCACTCACGTCATA
>SRR4252612_6710799___Sbg5
CCCACACCACACACACAATACAACACTA
>SRR4252612_6220482___Sbg5
TAGAAAACCGGGGTTATCGGCTGTGGGG
>SRR4252611_6509813___Sbg5
TTCCGTCCTATGGCGGCAGCCGCTCGGTA
>SRR4252619_3635493___Sbg5
GAGTGGTGGTGTACAACTTCTGATCACTA
>SRR4252611_2937501___Sbg5
TTCATATCCATAATCGTCACTCACATT
>SRR4252613_10050927___Sbg5
TTCATATCCATAATCGTCACTCACATT
>SRR4252611_13277009___Sbg5
TCCGTCCGTCGGATGGGGACGTTAAGCT
>SRR4252608_8164411___Sbg5
TGGTGTACAACTTCTGATCACTAAATTGC
>SRR4252608_4412068___Sbg5
TGTACAACTTCTGATCACTAAATTGC
>SRR4252610_6199149___Sbg5
GAGTGGTGGTGTACAACTTCTGATCACTAA
>SRR4252608_823784___Sbg5
TCCTCCTATCCGCACACATACACAATGA
>SRR4252610_11339323___Sbg5
TATCCGCACACATACACAATGATCAAA
>SRR4252613_10310719___Sbg5
TAGAAAACCGGGGTTATCGGCTGTGGGG
>SRR4252610_6528635___Sbg5
GAGTGGTGGTGTACAACTTCTGATCACTAA
>SRR4252610_9099465___Sbg5
TCGGCTGTGGGGACCAGCGTAGTACTG
>SRR4252621_9009407___Sbg5
CCGGGGCTATCGGCTGTGGGGACCAGCGC
>SRR4252608_3256062___Sbg5
TCCTCCTATCCGCACACATACACAATGA

>SRR4252606_4338621___Sbg5
ATCCGCACACATACACAATGATCAAATA
>SRR4252613_6253799___Sbg5
CCGCACACATACACAATGATCAAATA
>SRR4252623_12716848___Sbg5
GAGTGGTGGTGTACAACTTCTGATCACTAA
>SRR4252610_5105601___Sbg5
ATCCGCACACATACACAATGATCAAATA
>SRR4252611_5378138___Sbg5
AGTGGTGGTGTACAACTTCTGATCACTAA
>SRR4252611_4548387___Sbg5
TATGTAGAGAAGGCATATGTCATTGTT
>SRR4252618_4820760___Sbg5
CAATCCATTAACTGGTCTAGAAAACCG
>SRR4252611_3206081___Sbg5
GCCGGTCCCCTTGGTGCTTTTCGAAAGGA
>SRR4252612_1080092___Sbg5
GAGTGGTGGTGTACAACTTCTGATCACTAA
>SRR4252619_8474804___Sbg5
TGGTGGTGTACAACTTCTGATCACTAA
>SRR4252611_5244690___Sbg5
GAGTGGTGGTGTACAACTTCTGATCACTAA
>SRR4252624_7010725___Sbg5
ATCCACACACATACACAATGATCAAATA
>SRR4252605_82761___Sbg5
ACCTACTACCCCCCGTTAATTCCGTCCTA
>SRR4252609_14233373___Sbg5
AAGCCGTCGGACCCCTTGGTGTTTTTCGA
>SRR4252611_5234621___Sbg5
TTAATTCCGCCCTATGGCGGCAGCCGCTCG
>SRR4252605_10878982___Sbg5
AGAGTGGTGGTGTACAACTTCTGATCACTAA
>SRR4252611_6465458___Sbg5
AGAGTGGTGGTGTACAACTTCTGATCACTA
>SRR4252612_5225265___Sbg5
AGAGTGGTGGTGTACAACTTCTGATCACTAA
>SRR4252613_7898813___Sbg5
ACCATACTACCCCCCGTTAATTCCGTCCTA
>SRR4252607_6772633___Sbg5
TGTACAACTTCTGATCACTAAATTAAA
>SRR4252605_13735738___Sbg5
TTGATAGTGATCCGTCCGTCGGATGGGGAA
>SRR4252613_10620717___Sbg5
CAACTTCTGATCACTAAATTGTACAC
>SRR4252614_9148951___Sbg5
TCGGCTGTGGGGACCAGCATAGTACT
>SRR4252616_13201693___Sbg5
GGAGTGGTGGTGTACAACTTCTGATCACT
>SRR4252605_3184704___Sbg5
TCCGTCCGTCGGATGGGGACGTTAAG
>SRR4252605_7730036___Sbg5
AGTGGTGGTGTACAACTTCTGATCACTAA
>SRR4252610_4186107___Sbg5
ATCCCGAACTCCTCCGCAATGACTATG
>SRR4252621_11052914___Sbg5
ATCCGCACACATACACAATGATCAAATA
>SRR4252611_4003816___Sbg5
GAGTGGTGGTGTACAACTTCTGATCACTAA
>SRR4252605_8651672___Sbg5
AGTGGTGGTGTACAACTTCTGATCACTAA
>SRR4252611_1792738___Sbg5
TAATCGTCACTCACATCATACCACACCA
>SRR4252605_5817023___Sbg5
GGAAGGCATATGTCATTGTTGATAGTGA
>SRR4252625_3894458___Sbg5
TTAATTAAAATTTTGAATCCTGAGACTAG
>SRR4252623_2426297___Sbg5
GAGTAGTGGTGTACAACTTCTGATCACTAA
>SRR4252610_1249112___Sbg5
AGAGTGGTGGTGTACAACTTCTGATCACTA

>SRR4252610_12838777___Sbg5
AGAGTGGTGGTGTACAACTTCTGATCACTA
>SRR4252610_6778138___Sbg5
AGAGTGGTGGTGTACAACTTCTGATCACTA
>SRR4252612_5479844___Sbg5
AGAGTGGTGGTGTACAACTTCTGATCACTA
>SRR4252624_8878301___Sbg5
AGAGTGGTGGTGTACAACTTCTGATCACTAA
>SRR4252611_14333246___Sbg5
TCGGTAAGCCTTGGCCCACATTGGGCTG
>SRR4252606_1676899___Sbg5
CCGGTCCCCTTGGTGCTTTTCGAAAGGA
>SRR4252623_838321___Sbg5
AGTGGTGGTGTACAACTTCTGATCACTAA
>SRR4252621_4013906___Sbg5
GGAGTGGTGGTGTACAACTTCTGATCACTAA
>SRR4252615_13665304___Sbg5
GGAGTGGTGGTGTACAACTTCTGATCACTA
>SRR4252622_5570223___Sbg5
TCCCTCTTCCATATCCATAATCGTCACTC
>SRR4252612_8910229___Sbg5
CCATAATCGTCACTCACATCATACCACA
>SRR4252606_4965620___Sbg5
TCCTCCTATCCGCACACATACACAATGA
>SRR4252606_674245___Sbg5
CTATCCGCACACATACACAATGATCAAA
>SRR4252624_10597163___Sbg5
CGTCCGTCGGATGGGGACGTTAAGCT
>SRR4252608_280317___Sbg5
TCCCGAACTCCTCCGCAATGACTATGTGG
>SRR4252606_9130886___Sbg5
TATCCGCACACATACACAATGAACAAAT
>SRR4252609_9070617___Sbg5
TCCGCACACATACACAATGAACAAATAGC
>SRR4252610_4700202___Sbg5
TCCGCACACATACACAATGAACAAATAGC
>SRR4252615_10734276___Sbg5
CCATAATCGTCACTCACATCATACCA
>SRR4252610_2752049___Sbg5
GAGTGGTGGTGTACAACTTCTGATCACTAA
>SRR4252606_5869286___Sbg5
GAGTGGTGGTGTACAACTTCTGATCACTAA
>SRR4252610_11819547___Sbg5
ACCATACTACCCCCCGTTAATTCCGTCCTA
>SRR4252621_10628101___Sbg5
TTCCGTCCTATGGCGGCAGCCGCTCGGTA
>SRR4252610_109384___Sbg5
TACACACACTCATCCTCCTATCCGCACA
>SRR4252611_3088590___Sbg5
TTCCGCCCTATGGCGGCAGCCGCTCGGTA
>SRR4252608_6900341___Sbg5
CACACACTTACACACCGATCCATTAACTG
>SRR4252605_1682852___Sbg5
CCATACTACCCCCCGTTAATTCCGTCCTA
>SRR4252623_10555538___Sbg5
GTTAAATCCCGAACTCCTCCGCAATG
>SRR4252622_2279305___Sbg5
TCGGCTGTGGGGATCAGCGTAGTACTGAC
>SRR4252609_2047634___Sbg5
TAGAAAACCGGGGTTATCGGCTGTGGGG
>SRR4252611_12143893___Sbg5
GTGGTGGTGTACAACTTCTGATCACTAA
>SRR4252621_9015131___Sbg5
TCTCCCTCTTTCATATCCATAATCGT
>SRR4252611_8804400___Sbg5
TCATATCCATAATCGTCACTCACATCAT
>SRR4252610_4541444___Sbg5
TCCTCATATCCGCACACATACACAATGA
>SRR4252610_13230376___Sbg5
CTCATATCCGCACACATACACAATGA

>SRR4252611_14790608___Sbg5
TACAACTTCTGATCACTAAATTGTGCAC
>SRR4252622_571324___Sbg5
TCCGTCCGTCGGATGGGGACGTTAAGCCA
>SRR4252609_750894___Sbg5
CTTCTCCCTCTTTCATATCCATAATCGT
>SRR4252618_3631073___Sbg5
GTGGTGTACAACTTCTGATCACTGAATTG
>SRR4252612_7115478___Sbg5
GAGTGGTGGTGTACAACTTCTGATCACTAA
>SRR4252610_9711206___Sbg5
TCCTCCTATCCGCACACATACACAATGA
>SRR4252612_8466923___Sbg5
TCCTCCTATCCGCACACATACACAATGA
>SRR4252614_6058879___Sbg5
TAGAAAACCGGGGTTATCGGCTGTGGGG
>SRR4252605_9536580___Sbg5
TGGTGGTGTACAACTTCTGATCACTAA
>SRR4252612_2093256___Sbg5
ACACTCATCCTCCTATCCGCACACATACA
>SRR4252606_5053260___Sbg5
ACCACGCACACAATACAACACTATACACA
>SRR4252623_4665261___Sbg5
GTGGTGTACAACTTCTGATCACTAAAC
>SRR4252610_20098___Sbg5
ATGTCATTGTTGATAGTGATCCGTCCGTC
>SRR4252608_2389321___Sbg5
TGGTGTACAACTTCTGATCTCTAAATTG
>SRR4252612_1961315___Sbg5
CCTCTTTCATATCCATAATCGTCACTCA
>SRR4252611_1594267___Sbg5
TTCATATCCATAATCGTCACTCACGTCAT
>SRR4252615_14434711___Sbg5
TGTTGATAGTGATCCGTCCGTCGGATG
>SRR4252608_4371260___Sbg5
ATCCGCACACATACACAATGATCAAATA
>SRR4252612_1810387___Sbg5
GAGTGGTGGTGTACAACTTCTGATCACTA
>SRR4252606_4940684___Sbg5
GAGAAGGCATACGTCATTGTTGATAGTGA
>SRR4252612_6464210___Sbg5
CTCCTATCCGCACACATACACAATGATC
>SRR4252612_2204032___Sbg5
TAGAAAACCGGGGTTATCGGCTGTGGGG
>SRR4252610_12893658___Sbg5
TTGATAGTGATCCGTCCGTCGGATGGGG
>SRR4252610_9152300___Sbg5
ATATCCATAATCGTCACTCACATCATA
>SRR4252617_12631298___Sbg5
TGTACAACTTCTGATCACTAAATTGT
>SRR4252610_10093061___Sbg5
TCCATAATCGTCACTCACATCATACCACAC
>SRR4252606_8099241___Sbg5
TGTTTCATATCCATAATCGTCACTCACAT
>SRR4252606_2379055___Sbg5
TGGTGGCGTACAACTTCTGATCACTAA
>SRR4252606_4096380___Sbg5
TGGTGGCGTACAACTTCTGATCACTAA
>SRR4252611_4454913___Sbg5
TTCCGCCCTATGGCGGCAGCCGCTCGGTA
>SRR4252619_5889046___Sbg5
GGGAGTGGTGGTGTACAACTTCTGATCACTA
>SRR4252605_14122400___Sbg5
GAGTGGTGGTGTACAACTTCTGATCACTAA
>SRR4252608_10979841___Sbg5
GAGTGGTGGTGTACAACTTCTGATCACTAA
>SRR4252624_10080487___Sbg5
AGTGGTGGTGTACAACTTCTGATCACT
>SRR4252605_8183209___Sbg5
ATCCGCACACATACACAATGATCAAATA

>SRR4252611_11055618___Sbg5
ATCCGCACACATACACAATGATCAAATA
>SRR4252621_3415232___Sbg5
GAGTGGTGGTGTACAACTTCTGATCACTAA
>SRR4252610_13515962___Sbg5
GAGTGGTGGTGTACAACTTCTGATCACTA
>SRR4252625_6461907___Sbg5
TGTTGATAGTGATCCGTCCGTCGGATGGGG
>SRR4252619_14346458___Sbg5
TAGAAAACCGGGGTTATCGGCTGTGGGG
>SRR4252618_10677338___Sbg5
ATTCCGCCCTATGGCGGCAGCCGCTCGGTA
>SRR4252622_870405___Sbg5
AGTGGTGGTGTACAACTTCTGATCACTAA
>SRR4252606_9370787___Sbg5
GAGTGGTGGTGTACAACTTCTGATCACTAA
>SRR4252607_1759311___Sbg5
TATCCGCACACATACACAATGATCAAAT
>SRR4252611_3040926___Sbg5
AGTGGTGGTGTACAACTTCTGATCACTAA
>SRR4252611_11375364___Sbg5
TTGATAGTGATCCGTCCGTCGGATGGGGA
>SRR4252621_12856466___Sbg5
TCCGTCCGTCGGATGGGGACGTTAAGCTG
>SRR4252605_2802130___Sbg5
CCTCCGCAATGACTATGTAGAGAAGGCA
>SRR4252620_7726364___Sbg5
TATGTAGAGAAGGCATATGTCATTGTTGA
>SRR4252606_9746183___Sbg5
ATATCCATAATCGTCACTCACATCATA
>SRR4252606_6399691___Sbg5
ACACTCATCCTCCTATTCACACACTTACA
>SRR4252612_5716991___Sbg5
TTGATAGTGATCCGTCCGTCGGATGGGGACA
>SRR4252611_2957696___Sbg5
ATATCCATAATCGTCACTCACATCATA
>SRR4252605_7360431___Sbg5
CCTATCCGCACACATACACAATGATCA
>SRR4252607_6756701___Sbg5
ATCCGCACACATACACAATGATCAAATA
>SRR4252623_986790___Sbg5
TGGTGGTGTACAACTTCTGATCACTAAAT
>SRR4252612_5760921___Sbg5
GAGTGGTGGTGTACAACTTCTGATCACTAA
>SRR4252623_13577215___Sbg5
GAGTGGTGGTGTACAACTTCTGATCACTAA
>SRR4252611_8916469___Sbg5
TTCCGTCCTATGGCGGCAGCCGCTCGGTA
>SRR4252611_3058064___Sbg5
ATACAACACTATACACACACTCATCACCCT
>SRR4252619_9610600___Sbg5
TAGAAAACCGGGGTTATCGGCTGTGGGG
>SRR4252606_9151337___Sbg5
GAGTGGTGGTGTACAACTTCTGATCACTAA
>SRR4252624_49165___Sbg5
TCCTATCCGCACACATACACAATGATCA
>SRR4252612_1684369___Sbg5
GAGTGGTGGTGTACAACTTCTGATCACTAA
>SRR4252612_8154646___Sbg5
CATATCCATAATCGTCACTCACATCATA
>SRR4252624_7491241___Sbg5
TGTCATTGTTGATAGTGATCCGTCCGT
>SRR4252608_4996226___Sbg5
TCCTCCTATCCGCACACATACACAATGA
>SRR4252611_14323350___Sbg5
CACACAATACAACACTATACACACACTA
>SRR4252611_14377256___Sbg5
CACACAATACAACACTATACACACACTA
>SRR4252615_11057039___Sbg5
TAATTCCGTCCTATGGCGGCAACCGCTCG

```
>SRR4252623_8995310___Sbg5
CCGGGTTTCACCTTCTCCCTCTTTCATA
>SRR4252610_5770618___Sbg5
AACACACACAATACAACACTATACACACA
>SRR4252608_12445904___Sbg5
TGTACAACTTCTGATCACTAAATTGT
>SRR4252606_6499455___Sbg5
CTACATGCCGGCACCGGGTTTCACCTTCT
>SRR4252610_10813191___Sbg5
TACACACACTCATCCTCCTATCCGCACA
>SRR4252610_5449302___Sbg5
ATCCGCACACATACACAATGATCAAATA
>SRR4252612_2440620___Sbg5
TGATAGTGATCCGTCCGTCGGATGGGG
>SRR4252605_949251___Sbg5
GATCCGTCCGTCGGATGGGGACGTTA
>SRR4252610_6571258___Sbg5
CACTCATCCTCCTATCCGCACACATACA
>SRR4252613_8260132___Sbg5
TAGAAAACCGGGGTTATCAGCTGTGGGG
>SRR4252611_7516760___Sbg5
ACTACCCCCCGTTAATTCCGTCCTATGG
>SRR4252611_13984521___Sbg5
TAGTGATCCGTCCGTCGGATGGGGACGG
>SRR4252608_3298503___Sbg5
TAGAAAACCGGGGTTATCGGCTGTGTG
>SRR4252621_4404748___Sbg5
GACAGTGATCCGTCCGTCGGATGGGGAC
>SRR4252622_8028916___Sbg5
ACGGTCCCCTTGGTGCTTTTCGAAAGGA
>SRR4252621_8049735___Sbg5
TATCGGCTGTGGGGACCAGCGTAGTACTG
>SRR4252607_13954731___Sbg5
TTGATAGTGATCCGTCCGTCGGATGGGG
>SRR4252606_5621341___Sbg5
ACACCGGGTTTCACCTTCTCCCTCTTTCATA
>SRR4252608_3572216___Sbg5
GAGTGGTGGTGTACAACTTCTGATCACTAA
>SRR4252611_7550276___Sbg5
TTCATATCCATAATCGTCACTCACATCAT
>SRR4252614_5542217___Sbg5
TCGGCTGTGGGGACCAGCATAGTACTGAT
>SRR4252613_8946584___Sbg5
GTAGGCTACATGCCGGCACCGGGTTTCAC
>SRR4252606_5002439___Sbg5
GAGTGGTGGTGTACAACTTCTGATCACTAA
>SRR4252606_6191702___Sbg5
GAGTGGTGGTGTACAACTTCTGATCACTAA
>SRR4252610_7768363___Sbg5
GAGTGGTGGTGTACAACTTCTGATCACTAA
>SRR4252611_7008710___Sbg5
GATTAAATCCCGAACTCCTCCGCAATGACT
>SRR4252611_674223___Sbg5
TCATATCCATAATCGTCACTCATATCATA
>SRR4252605_14564799___Sbg5
TTGATAGTGATCCGTCCGTCGGATGGGGAC
>SRR4252606_7840978___Sbg5
TCATTGTTGATAGTGATCCGTCCGTC
>SRR4252616_3661368___Sbg5
TATCCATAATCGTCACTCACATCATA
>SRR4252607_791829___Sbg5
TCACACACTTACACACCGATCCATTAACTG
>SRR4252609_14849739___Sbg5
GAGTGGTGGTGTACAACTTCTGATCATTAA
>SRR4252623_8141874___Sbg5
GAGTGGTGGTGTACAACTTCTGATCATTAA
>SRR4252610_12868960___Sbg5
AGTGGTGGTGTACAACTTCTGATCATTAA
>SRR4252605_13996759___Sbg5
TGGTGTACAACTTCTGATCATTAAATT
```

>SRR4252616_2446687___Sbg5
TCCATAATCGTCACTCACATCATACCA
>SRR4252605_8941596___Sbg5
GATCACTAAATTGTACACCAAATTCA
>SRR4252611_11310806___Sbg5
GCCGGTCCCCTTGGTGCTTTTCGAAAGGA
>SRR4252611_6630228___Sbg5
AGGCATATGTCATTGTTGATAGTGATCCGTC
>SRR4252606_10028878___Sbg5
AACACTCATCCCCCTATCCACACACTTACA
>SRR4252612_6280043___Sbg5
ACAATACAACACTATACACACACTCA
>SRR4252611_7582873___Sbg5
ATCCGCACACATACACAATGATCAAATA
>SRR4252605_10310218___Sbg5
TCCTCCTATCCGCACACATACACAATGA
>SRR4252620_9520924___Sbg5
ATACCACACCACACACACAATACAACTC
>SRR4252605_2868461___Sbg5
TCGCACACATACACAATGATCAAATA
>SRR4252612_8994948___Sbg5
TCGCACACATACACAATGATCAAATAGCT
>SRR4252611_1370671___Sbg5
ATCCGCACACATACACAATGATCAAATA
>SRR4252608_5911808___Sbg5
ATCCGCACACATACACAATGATCAAATA
>SRR4252606_3768802___Sbg5
TGATAGTGATCCGTCCGTCGGATGGGACG
>SRR4252619_12899245___Sbg5
TATCATATCCATAATCGTCACTCACATCA
>SRR4252624_7628725___Sbg5
ATCATATCCATAATCGTCACTCACATCATA
>SRR4252608_12135768___Sbg5
TTGATAGTGATCCGTCCGTCGGATGGGA
>SRR4252612_8975044___Sbg5
AGAAGGCATATGTCATTGTTGATAGTGA
>SRR4252614_6988069___Sbg5
TGTACAACTTCTGATCACTAAATTGTGCAC
>SRR4252624_2354471___Sbg5
CCATACTACCCCCCGTTAATTCCGTCCTA
>SRR4252612_3985605___Sbg5
TCGGTCCCCTTGGTGCTTTTCGAAAGGA
>SRR4252617_6212237___Sbg5
ACCCTCCTATCCGCACACATACACAATGA
>SRR4252620_629995___Sbg5
TATCGGCTGTGGGGATCAGCGTAGTAC
>SRR4252621_8364911___Sbg5
GAGTGGTGGTGTACAACTTCTGATCACTAA
>SRR4252612_1239346___Sbg5
AGTGGTGGTGTACAACTTCTGATCACTAA
>SRR4252608_3965671___Sbg5
GAGTGGTGGTGTACAACTTCTGATCACTAA
>SRR4252610_11223718___Sbg5
CCTCCTATCCGCACACATACACAATGA
>SRR4252612_334721___Sbg5
TCCTATCCGCACACATACACAATGATC
>SRR4252609_1759646___Sbg5
ATCCGCACACATACACAATGATCAAATA
>SRR4252605_4736249___Sbg5
TCCGCACACATACACAATGATCAAATA
>SRR4252611_1359454___Sbg5
ACCGGGTTTCACCTTCTCCCTCTTTCATA
>SRR4252612_3758146___Sbg5
TCGGTAAGCCTTGGCCCACATGGGGCTGTT
>SRR4252605_903445___Sbg5
TCCTCCTATCCGCACACATACACAATGA
>SRR4252612_1112654___Sbg5
ACACTCATCCTCCTATTCACACACTTACA
>SRR4252606_6244286___Sbg5
GTTATCGGCTGTGGGGACCAGCGTAC

>SRR4252605_12761217___Sbg5
TCCTCCTATCCGCACACATACACAATGA
>SRR4252610_8958217___Sbg5
ACACACACTCATCCTCCTATCCGCACA
>SRR4252610_7569062___Sbg5
TCCTCCTATCCGCACACATACACAATGA
>SRR4252619_2676086___Sbg5
ACACCACACACACAATACAACACTATA
>SRR4252620_6187756___Sbg5
TGTTGATAGTGATCCGTCCGTCGGATGG
>SRR4252611_8540543___Sbg5
TCTAGAAAACCGGGGTTATCGGCTGTGG
>SRR4252605_14820175___Sbg5
GTGCTTTTCGAAAGGAGTAGGCTACATG
>SRR4252611_710786___Sbg5
GTAGAGAAGGCATATGTCATTGTTGATA
>SRR4252626_3796334___Sbg5
AATTCCGCCCTATGGCGGCAGCCGCTCGG
>SRR4252605_14993224___Sbg5
CCTGAGACTAGGAGGAGTGGTGGCGT
>SRR4252611_7736221___Sbg5
GGTTAAATCCCGAACTCCTCCGCAATG
>SRR4252605_10548189___Sbg5
CCGAACTCCTCCGCAATGACTATGTTGAGA
>SRR4252605_4826473___Sbg5
ATACAACACTATACACACACTCATCCT
>SRR4252611_2011144___Sbg5
CCTCCTATCCGCACACATACACAATGA
>SRR4252608_3091616___Sbg5
TAGAAAACCGGGGTTATCGGCTGTGGGG
>SRR4252619_13805284___Sbg5
GAGTGGTGGCGTACAACTTCTGATCACTAA
>SRR4252623_7470061___Sbg5
GAGTGGTGGCGTACAACTTCTGATCACTAA
>SRR4252608_11084897___Sbg5
GAGTGGTGGTGTACAACTTCTGATCACTAA
>SRR4252620_11016492___Sbg5
TGTACAACTTCTGATCACTAAATTGT
>SRR4252622_1397347___Sbg5
TTGGCCCACATAGGGCTGTTGCGCTAT
>SRR4252610_7867221___Sbg5
TGGTGTACAACTTCTGATCACTAAATTGT
>SRR4252612_420386___Sbg5
TTGATAGTGATCCGTCCGTCGGATGT
>SRR4252623_3555676___Sbg5
CACACACACAATACAACACTATACACACA
>SRR4252610_7723090___Sbg5
ATCTTTCATATCCATAACCGTCACTCACA
>SRR4252620_1884463___Sbg5
TAGAAAACCGGGGTTATCGGCTGTGGGG
>SRR4252619_4008233___Sbg5
TCGGCTGTGGGGATCAGCGTAGTACTG
>SRR4252606_7792458___Sbg5
TGGTGTACAACTTCTGATCACTAAATTGG
>SRR4252607_85988___Sbg5
TGTACAACTTCTGATCACTAAATTGG
>SRR4252610_7932688___Sbg5
TGGTGTACAACTTCTGATCACTACATTG
>SRR4252612_5539121___Sbg5
ACACACAATACAACACTATACACACTCA
>SRR4252608_6454598___Sbg5
TCCTCCTATCCGCACACATACACAATGA
>SRR4252610_12133761___Sbg5
GGTGGTGGTGTACAACTTCTGATCACTAA
>SRR4252611_10978414___Sbg5
TTGATAGTGATCCGTCCGTCGGATGGGGAC
>SRR4252610_4038265___Sbg5
TCATATCCATAATCGTCACTCACATCATA
>SRR4252612_8236829___Sbg5
ATCCGCACACATACACAATGATCAAATA

```
>SRR4252621_1224475___Sbg5
TAGAAAACCGGGGTTATCGGCTGTGGG
>SRR4252621_10516641___Sbg5
TGGTGTACAACTTCTGATCACTAAATTGT
>SRR4252623_5367516___Sbg5
GAGACTAGGAGGAGTGGTGGTGTACAACTTC
>SRR4252610_2086919___Sbg5
GTGTACAACTTCTGATCACTAAATTGTGC
>SRR4252606_2226078___Sbg5
GAGTGGTGGTGTACAACTTCTGATCACTAA
>SRR4252606_6209270___Sbg5
GAGTGGTGGTGTACAACTTCTGATCACTAA
>SRR4252617_3806136___Sbg5
TGTTGATAGTGATCCGTCCGTCGGATGG
>SRR4252609_15170367___Sbg5
CCTTGGTGTTTTTCGAAAGGAGTAGGCT
>SRR4252610_4527912___Sbg5
CAACACTATACACACACTCATCCTCCTA
>SRR4252611_12497379___Sbg5
CCTCCTATCCGCACACATACACAATGA
>SRR4252612_8127376___Sbg5
ATCCGCACACATACACAATGATCAAATA
>SRR4252606_8441537___Sbg5
TCCTCCTATCCGCACACATACACAATGA
>SRR4252611_1305428___Sbg5
TCCTCCTATCCGCACACATACACAATGA
>SRR4252610_488289___Sbg5
CCTCCTATCCGCACACATACACAATGA
>SRR4252606_6978395___Sbg5
CTCCTATCCGCACACATACACAATGATCA
>SRR4252621_6008848___Sbg5
AATGGTGGTGTACAACTTCTGATCACTAA
>SRR4252621_12585321___Sbg5
CATCCTCCTATCCGCACACATACACA
>SRR4252609_15535760___Sbg5
GAATGGTGGTGTACAACTTCTGATCACTA
>SRR4252612_8798699___Sbg5
GAATGGTGGTGTACAACTTCTGATCACTAA
>SRR4252610_787089___Sbg5
ATCCGCACAAATACACAATGATCAAATA
>SRR4252623_7800805___Sbg5
TCCGCACAAATACACAATGATCAAATA
>SRR4252606_8404880___Sbg5
CGAACTCCTCCGCAATGACTATGTAGA
>SRR4252606_3432245___Sbg5
ACCGGTCCCCTTGGTGCTTTTCGAAAGGA
>SRR4252620_6016874___Sbg5
ACCGGTCCCCTTGGTGCTTTTCGAAAGGA
>SRR4252605_8399969___Sbg5
CCAGGTTTCACCTTCTCCCTCTTTCATA
>SRR4252621_8761625___Sbg5
TATCGGCTGTGGGGACCAGCGTGGTACT
>SRR4252606_5607440___Sbg5
TCGGCTGTGGGGACCAGCGTGGTACTGAC
>SRR4252606_854176___Sbg5
TCGGCTGTGGGGACCAGCGTGGTACTGA
>SRR4252606_4139206___Sbg5
ATCCGTCCGTCGGATGGGGACGTTAA
>SRR4252606_4480303___Sbg5
ACCGGTCCCCTTGGTGCTTTTCGAAAGGA
>SRR4252608_9355932___Sbg5
ATAAGTCATTGTTGATAGTGATCCGTCC
>SRR4252610_208974___Sbg5
GAGTGGTGGTGTACAACTTCTGATCACTAA
>SRR4252610_4444375___Sbg5
TTGATAGTGATCCGTCCGTCGGATGT
>SRR4252610_8965753___Sbg5
GAGTGGTGGTGTACAACTTCTGATCACTAA
>SRR4252621_6138481___Sbg5
GCCGGTCCCCTTGGTGCTTTTCGAAAGGA
```

>SRR4252608_12401695___Sbg5
CCAGATTTCACCTTCTCCCTCTTTCATA
>SRR4252615_8370696___Sbg5
CTTGGCCCACATCGGGCTGTTGAGCTAT
>SRR4252624_8712084___Sbg5
TGTACAACTTCTGATCACTAAATTGTG
>SRR4252610_11127894___Sbg5
AAGTGGTGGTGTACAACTTCTGATCACTA
>SRR4252610_4762857___Sbg5
AAGTGGTGGTGTACAACTTCTGATCACTAA
>SRR4252611_2885442___Sbg5
AAGTGGTGGTGTACAACTTCTGATCACTAA
>SRR4252606_5754688___Sbg5
TGAGTGGTGGTGTACAACTTCTGATCACT
>SRR4252612_7306355___Sbg5
TGAGTGGTGGTGTACAACTTCTGATCACT
>SRR4252610_3946734___Sbg5
GTGGTGGTGTACAACTTCTGATCACTAA
>SRR4252607_6474214___Sbg5
GAGAAGGCATACGTCATTGTTGATAGTG
>SRR4252612_4443337___Sbg5
TCGGTCCCCTTGGTGCTTTTCGAAAGGA
>SRR4252610_13395633___Sbg5
GTGGTTGTGTACAACTTCTGATCACTAA
>SRR4252608_5162044___Sbg5
CACACACAATACAACACTATACACACA
>SRR4252613_905194___Sbg5
TCTAGAAAACCGGGGTTATCGGCTGTG
>SRR4252610_7593982___Sbg5
TCCGTCCGTCGGATGGGGACGTTAAGCT
>SRR4252609_13000098___Sbg5
TCCATAATCGTCACTCACATCATACCACACC
>SRR4252608_5680232___Sbg5
TTGATAGTGATCCGTCCGTCGGATGGGG
>SRR4252611_13813904___Sbg5
ACAACTTCTGATCACTAAATTGTACA
>SRR4252606_7267487___Sbg5
GAATGGTGGTGTACAACTTCTGATCACTA
>SRR4252612_3195638___Sbg5
ACTCATCCTCCTATCCGCACACATACA
>SRR4252621_10311136___Sbg5
ATGGTGGTGTACAACTTCTGATCACTAA
>SRR4252606_798639___Sbg5
ACACACACTCATCCTCCTATCCGCACA
>SRR4252606_1259174___Sbg5
CACACATACACAATGATCAAATAGCT
>SRR4252610_8717850___Sbg5
CTTCCATATCCATAATCGTCACTCACA
>SRR4252615_13291597___Sbg5
TTCCGCCCTATGGCGGCAGCCGCTCGGTA
>SRR4252612_4549057___Sbg5
CCACACCACACACACAATACAACACTATA
>SRR4252612_1571269___Sbg5
GCACACATACACAATGATCAAATAGCT
>SRR4252606_10000295___Sbg5
CGTCATTGTTGATAGTGATCCGTCCGTC
>SRR4252608_14664___Sbg5
TGCTTTTCGAAAGGAGTAGGCTACATGA
>SRR4252611_10862685___Sbg5
TCAAATCCATAATCGTCACTCACATCATA
>SRR4252606_6750334___Sbg5
GAGTGGTGGTGTACAACTTCTGATCACTA
>SRR4252613_4492906___Sbg5
GAGTGGCGGTGTACAACTTCTGATCACTAA
>SRR4252605_2185931___Sbg5
TCACTCACATCATACCACACCACACA
>SRR4252621_10287825___Sbg5
GGAGTGGTGGTGTACAACTTCTGATCACTAA
>SRR4252605_14725804___Sbg5
GAGTGGTGGTGTACAACTTCTGATCACTAA

```
>SRR4252606_9862807___Sbg5
GAGTGGTGGTGTACAACTTCTGATCACTAA
>SRR4252610_13108789___Sbg5
TATCCATAATCGTCACTCACATCATACCAC
>SRR4252612_5535588___Sbg5
TTGGCCCACATCGGGCTGTTGAGCTACAG
>SRR4252620_3257605___Sbg5
TTGGCCCACATCGGGCTGTTGAGCTAC
>SRR4252606_5201994___Sbg5
TGGCCCACATCGGGCTGTTGAGCTAC
>SRR4252610_10442088___Sbg5
GAGTGGTGGTGTACAACTTCTGATCACTAA
>SRR4252610_4873904___Sbg5
CCGGTCCCCTTGGTGCTTTTCGAAAGGA
>SRR4252625_12795439___Sbg5
AGTGGTGGTGTACAACTTCTGATCAC
>SRR4252610_13375812___Sbg5
TGTACAACTTCTGATCACTAAATTGT
>SRR4252612_7790014___Sbg5
TCTTTCATACCCATAATCGTCACTCACAT
>SRR4252624_10763126___Sbg5
TCTAGAAAACCGGGGTTATCGGCTGTG
>SRR4252610_12386405___Sbg5
ACACTCATCCCCCTATCCACACACTTACA
>SRR4252611_12687741___Sbg5
GAGTGGTGGTGTACAACTTCTGATCACTAA
>SRR4252611_1891994___Sbg5
TATCCGCACACATACACAATGATCAAATA
>SRR4252610_7864736___Sbg5
TGGTGTACAACTTCTGATCACTAAATTGT
>SRR4252606_10123675___Sbg5
TATCCATAATCGTCACTCACATCATA
>SRR4252606_2215807___Sbg5
GTCACTCACATCATACCACACCACACACAC
>SRR4252606_2216135___Sbg5
GGAGTGGTGGTGTACAACTTCTGATCAC
>SRR4252621_3444685___Sbg5
GGAGTGGTGGTGTACAACTTCTGATCACTAA
>SRR4252605_12302126___Sbg5
GAGTGGTGGTGTACAACTTCTGATCACTAA
>SRR4252623_9723737___Sbg5
CCGCACACATACACAATGATCAAATA
>SRR4252621_8252781___Sbg5
CCGGGTTTCACCTTCTCCCTCTTTCATA
>SRR4252608_3974259___Sbg5
AGTGGTGGTGTACAACTTCTGATCAAAT
>SRR4252611_2696049___Sbg5
GAGTGGTGGTGTACAACTTCTGATCACTAA
>SRR4252608_5567354___Sbg5
GAGTGGTGGTGTACAACTTCTGATCACTAA
>SRR4252624_6392895___Sbg5
TTGATAGTGATCCGTCCGTCGGATGGGGAC
>SRR4252605_13958198___Sbg5
TCCGTCCGTCGGATGGGGACGTTAAGT
>SRR4252612_1324280___Sbg5
TTCATATCCATAATCGTCACTCACGTC
>SRR4252614_5031564___Sbg5
GTGATCCGTCCGTCGGATGGGGACGTTA
>SRR4252625_11571192___Sbg5
TCGGCTGTGGGGACCAGCGTAATACTGAC
>SRR4252606_4349387___Sbg5
CTCCTATCCGCACACATACACAATGA
>SRR4252619_2854197___Sbg5
CTCGGTAAGCCTTGGCCCACATCGGGCTG
>SRR4252612_6626857___Sbg5
TGGCCCACATCGGGCTGTTGAGCTAT
>SRR4252621_1461709___Sbg5
TCCTCCTATCCGCACACATACACAATGA
>SRR4252607_10771619___Sbg5
TCGGCTGTGGGGACCAGCGTAGTACTGAC
```

>SRR4252605_2623283___Sbg5
ATTGTGGTGTACAACTTCTGATCACTAA
>SRR4252611_7881556___Sbg5
CCATACTACCCCCCGTTAATTCCGTCCTA
>SRR4252610_1720018___Sbg5
TAGTGGTGGTGTACAACTTCTGATCACTAA
>SRR4252612_7636493___Sbg5
TAGTGGTGGTGTACAACTTCTGATCACTA
>SRR4252624_2059212___Sbg5
TAGTGGTGGTGTACAACTTCTGATCACTAA
>SRR4252614_4931442___Sbg5
GTGGTGTACAACTTCTGATCACTAAATC
>SRR4252610_2083955___Sbg5
CATATCCATAATCGTCACTCGCATCAT
>SRR4252610_9838676___Sbg5
ATCCGCACACATACACAATGATCAAATA
>SRR4252612_3866576___Sbg5
TAGAAAACCGGGGTTATCGGCTGTGGG
>SRR4252624_1663778___Sbg5
TACACACACTCATCCTCCTATCCGCACA
>SRR4252606_1446920___Sbg5
GCCGGTCCCCTTGGTGCTTTTCGAAAGGA
>SRR4252611_12025274___Sbg5
ATCCGCACACATACACAATGATCAAATA
>SRR4252610_1685796___Sbg5
GTGGTGTACAACTTCTGATCACTAAATT
>SRR4252606_2532291___Sbg5
GTATCCATAATCGTCACTCACATCATA
>SRR4252610_5802150___Sbg5
ATATCCATAATCGTCACTCACATCATA
>SRR4252610_5732473___Sbg5
GAGTGGTGGTGTACAACTTCTGATCACTAA
>SRR4252622_8649245___Sbg5
TACACACACTTACACACCAATCCATTA
>SRR4252610_11488569___Sbg5
TCCGTCGGATGGGGACGTTAAGCCGTCGGAC
>SRR4252619_8715256___Sbg5
CTCCTATCCGCACACATACACAATGATCA
>SRR4252622_7964822___Sbg5
GTCTAGAAAACAGGGGTTATCGGCTGTGG
>SRR4252612_76225___Sbg5
CCGAGTTTCACCTTCTCCCTCTTTCATA
>SRR4252610_1114141___Sbg5
CTCTTTCATATCCATAATCGTCACTCAT
>SRR4252613_8022694___Sbg5
CCTTGGCCCACATCGGGCTGTTGAGCTATA
>SRR4252605_13943149___Sbg5
TGGTGTACAACTTCTGATCATTAAATT
>SRR4252610_20500___Sbg5
TGGTGTACAACTTCTGATCATTAAATTG
>SRR4252620_11192017___Sbg5
TGGTGTACAACTTCTGATCATTAAATTGTAT
>SRR4252614_647131___Sbg5
AGTAATTAATTAAAATTTCGAATCCTG
>SRR4252610_1034398___Sbg5
CCTTATCCCTCTTTCATATCCATAATCG
>SRR4252605_13208756___Sbg5
TCATATCCATAATCGTCACTCACATCATA
>SRR4252609_9975863___Sbg5
GGAGTGGTGGTGTACAACTTCTGATCACT
>SRR4252608_5607224___Sbg5
CTCCTATCCGCACACATACACAATGATCA
>SRR4252609_4348908___Sbg5
TTATCGGCTGTGGGGATCAGCGTAGTACT
>SRR4252606_9573521___Sbg5
AATCCCGAACTCCTCCGCAATGACTATG
>SRR4252622_1392563___Sbg5
AGGCTACATGCCGGCACCGGGTTTCAC
>SRR4252610_4349041___Sbg5
GGGTGGTGGTGTACAACTTCTGATCACTAA

>SRR4252611_11331338___Sbg5
TTCATATCCATAATCGTCACTCACGTCAT
>SRR4252612_386550___Sbg5
ATCCGCACACATACACAATGATCAAATA
>SRR4252614_641924___Sbg5
GCTGGTCTAGAAAACCGGGGTTATCGGT
>SRR4252606_6450207___Sbg5
ACTCATCTTCCTATCCGCACACATACACA
>SRR4252605_6090968___Sbg5
ATCCGCACACATACACAATGATCAAATA
>SRR4252605_722017___Sbg5
TTCCACCCTATGGCGGCAGCCGCTCGGTA
>SRR4252606_601151___Sbg5
ACACTCATCTCCCTATTCACACACTTACA
>SRR4252611_1844407___Sbg5
TCACACATACACAATGATCAAATAGCT
>SRR4252612_4734609___Sbg5
ACACTCATCTCCCTATTCACACACTTACA
>SRR4252623_263185___Sbg5
TAACGTCCTATGGCGGCAGCCGCTCGGTA
>SRR4252619_8806752___Sbg5
TCGGTAAGCCTTGGCCCACATGGGGCTG
>SRR4252606_9001150___Sbg5
GAGTGGTGGTGTACAACTTCTGATCACTAA
>SRR4252608_2254820___Sbg5
CCTATCCGCACACATACACAATGATCAA
>SRR4252626_6886296___Sbg5
GAGTGGTGGTGTACAACTTCTGATCACTAA
>SRR4252617_15019442___Sbg5
GCTTTTCGGAAGGAGTAGGCTACATGCCG
>SRR4252623_5139925___Sbg5
CCGGGTTTCACCTTCTCCCTCTTTCATA
>SRR4252606_5471777___Sbg5
TCCTATCCGCACACATACACAATGATCA
>SRR4252619_13566663___Sbg5
ATTGTTAATAGTGATCCGTCCGTCGGATGGG
>SRR4252611_9843935___Sbg5
TTGATAGTGATCCGTCCGTCGGATAGGGAC
>SRR4252621_5786780___Sbg5
CACTCATCCTCCTATCCACACACTTACA
>SRR4252614_461738___Sbg5
TAGAAAACCGGGGTTATCGGCTGTGGGG
>SRR4252616_6804842___Sbg5
TCGGCTGTGGGGACCAGCGTAGTACTG
>SRR4252610_887269___Sbg5
CATACTACCCCCCGTTAATTCCGTCCTA
>SRR4252605_4676559___Sbg5
TCCTCCTATCCGCACACATACACAATGA
>SRR4252620_3985273___Sbg5
CAGTGATCCGTCCGTCGGATGGGGAC
>SRR4252613_4877274___Sbg5
TCACACACTTACACACCGATCCATTAACTG
>SRR4252607_8331846___Sbg5
TATCGGCTGTGGGGACCAGCGTAGTACTA
>SRR4252610_1502820___Sbg5
GAGTGGTGGTGTACAACTTCTGATCACTAA
>SRR4252605_2635625___Sbg5
TTGATAGTGATCCGTCCGTCGGATGG
>SRR4252619_15238180___Sbg5
TAGAAAACCGGGGTTATCGGCTGTGGGG
>SRR4252606_5395225___Sbg5
AGGCTACATGCCGGCACCGGGTTTCA
>SRR4252607_9467694___Sbg5
TTCATATCCATAATCGTCACTCACATCA
>SRR4252606_992488___Sbg5
GAGTGGTGGTGTACAACTTCTGATCACTAA
>SRR4252621_10622912___Sbg5
GAGTGGTGGTGTACAACTTCTGATCACTAA
>SRR4252624_5815610___Sbg5
TCATATCCATAATCGTCACTCACATCATA

>SRR4252613_5069980___Sbg5
TGGTGTACAACTTCTGATCACTAAATTGT
>SRR4252623_5091980___Sbg5
CCATACTACCCCCCGTTAATTCCGTCCTA
>SRR4252611_11809239___Sbg5
AAGTGGTGGTGTACAACTTCTGATCACTAA
>SRR4252626_1885356___Sbg5
AAGTGGTGGTGTACAACTTCTGATCACTAA
>SRR4252610_6011144___Sbg5
AGAAGGCATACGTCATTGTTGATAGTGA
>SRR4252624_10437345___Sbg5
CTACATGCTGGCACCGGGTTTCACCTTCT
>SRR4252606_2595597___Sbg5
CCATATCCATAATCGTCACTCACATCATA
>SRR4252606_6435236___Sbg5
CCATATCCATAATCGTCACTCACATCATA
>SRR4252608_5805776___Sbg5
ATCCACACACTTACACACCGATCCATTA
>SRR4252606_4806941___Sbg5
TATCCACACACTTACACACCAATCCATT
>SRR4252608_12334841___Sbg5
TATACTACCCCCCGTTAATTCCGTCCTA
>SRR4252611_14237515___Sbg5
AGTGAACCGTCCGTCGGATGGGGACGTTA
>SRR4252612_6879954___Sbg5
TTCCGTCCTATGGCGGCAGCCGCTCGGTAA
>SRR4252626_4093092___Sbg5
GGAATGGTGGTGTACAACTTCTGATCACTAA
>SRR4252606_9361334___Sbg5
GAATGGTGGTGTACAACTTCTGATCACTAA
>SRR4252610_13531373___Sbg5
TATGTTGAGAAGGCATATGTGATTGTTG
>SRR4252606_9723248___Sbg5
GCCGGTCCCCTTGGTGCTTTTCGAAAGGA
>SRR4252610_9868197___Sbg5
CACCGGGTTTCTCCTTCTCCCTCTTTCATA
>SRR4252606_7208238___Sbg5
TACCGGGTTTCACCTTCTCCCTCTTTCATA
>SRR4252607_12128179___Sbg5
ATATCCATAATCGTCACTCACATCAC
>SRR4252607_14333770___Sbg5
AATGGTGGTGTACAACTTCTGATCACTAA
>SRR4252605_6060257___Sbg5
ATCATATCCATAATCGTCACTCACATCATA
>SRR4252608_7839762___Sbg5
ATCATATCCATAATCGTCACTCACATCATA
>SRR4252609_13258058___Sbg5
AGAAGGCATATGTCATTGTTGATAGTGA
>SRR4252611_12290279___Sbg5
CCGGGTTTCACCTTCTCCCTCTTTCATA
>SRR4252622_8619020___Sbg5
TCCCTCTTTCATATCCATAGTCGTCAC
>SRR4252605_12968273___Sbg5
CCATACTACCCCCCGTTAATTCCGTCCTA
>SRR4252608_3785016___Sbg5
ATTCCGCCCTATGGCGGCAGCCGCTCGGTA
>SRR4252607_9518974___Sbg5
ACCGAGTTTCACCTTCTCCCTCTTTCATA
>SRR4252610_4312514___Sbg5
TTCATATCCATAATCGTCACTCACATCATAC
>SRR4252610_11806353___Sbg5
ATCCGCACACATACACAATGATCAAATA
>SRR4252616_8062790___Sbg5
TAGAAAACCGGGGTTATCGGCTGTGGGG
>SRR4252609_13600955___Sbg5
GAGAAGGCACATGTCATTGTTGATAGT
>SRR4252609_1433765___Sbg5
TATCCATAACCGTCACTCACATCATC
>SRR4252618_9930170___Sbg5
AGGCATATGTCATTGTTGATAGTGATCCGT

>SRR4252615_12778906___Sbg5
TAGTGATCCGTCCGTCGGATGGGACGT
>SRR4252615_1564411___Sbg5
TATCCATAATCGTCACTCACATCATAC
>SRR4252605_8825948___Sbg5
GAGTGTTGGTGTACAACTTCTGATCACTAA
>SRR4252610_12628458___Sbg5
GAGTGTTGGTGTACAACTTCTGATCACTAA
>SRR4252610_13413159___Sbg5
GAGTGTTGGTGTACAACTTCTGATCACTAA
>SRR4252619_3722476___Sbg5
GAGTGTTGGTGTACAACTTCTGATCACTAA
>SRR4252605_2514498___Sbg5
AGTGTTGGTGTACAACTTCTGATCACTAA
>SRR4252605_12934985___Sbg5
TGTTGGTGTACAACTTCTGATCACTAA
>SRR4252613_11357417___Sbg5
ACTAGGAGGAGTGGTGGTGTACAACTT
>SRR4252606_4657272___Sbg5
AGTGGTGGTGTACAACTTCTGATCAC
>SRR4252607_3633612___Sbg5
CTCTTTCATATCCATAATCGTCACTCACATT
>SRR4252612_3272726___Sbg5
CAATACAACACTATACACACACTCACCC
>SRR4252614_8490266___Sbg5
TATGTAGAGAAGGCATATGTCATTGTTGA
>SRR4252612_4351356___Sbg5
GGAGTGGTGGTGTACAACTTCTGATCACTA
>SRR4252610_3453200___Sbg5
TGGTGTACAACTTCTGATCACTAAATTGT
>SRR4252605_14916413___Sbg5
GAAGGCATATGTCATTGTTGATAGTGAT
>SRR4252618_4966788___Sbg5
CATATGTCATTGTTGATAGTGATCCG
>SRR4252605_15036020___Sbg5
TGTCCCCTTGGTGCTTTTCGAAAGGA
>SRR4252608_18530___Sbg5
CCATACTACCCCCCGTTAATTCCGTCCTA
>SRR4252620_6172917___Sbg5
TATGTTGAGAAGGCATATGTCATTGTTG
>SRR4252621_1485091___Sbg5
CATATCCATAATCGTCACTCACATCATA
>SRR4252609_15022791___Sbg5
TCGGCTGTGGGGACCAGCGTAGTACTGT
>SRR4252623_7025878___Sbg5
TCGGCTGTGGGGACCAGCGTAGTACTGT
>SRR4252610_12431321___Sbg5
TCGGCTGTGGGGACCAGCGTAGTACTGA
>SRR4252610_7358328___Sbg5
TGTACAACTTCTGATCACTAAATTGT
>SRR4252610_4946190___Sbg5
TCCTATCCGCACACATACACAATGATCA
>SRR4252606_1548013___Sbg5
TCCTCCTATCCGCACACATACACAATGA
>SRR4252609_13569071___Sbg5
TCCTCCTATCCGCACACATACACAATGA
>SRR4252610_11858515___Sbg5
TCCGCACACATACACAATGATCAAATA
>SRR4252614_571278___Sbg5
ACACATACACAATGATCAAATAGCTGGTC
>SRR4252606_1396610___Sbg5
GCCTCCTATCCGCACACATACACAATGA
>SRR4252618_7454920___Sbg5
TATCCACACACTTACACACCAATCCATTA
>SRR4252614_4721089___Sbg5
ATCCGCACACATACACAATGATCAAATA
>SRR4252605_349257___Sbg5
CACCACACACACAATACAACACTATACAC
>SRR4252615_9749929___Sbg5
TGTTGATAGTGATCCGTCCGTCGGATGG

>SRR4252610_10095244___Sbg5
TTGATAGTGATCCGTCCGTCGGATGGGGAT
>SRR4252619_470609___Sbg5
TGGTGGTGTACAACTTCTGATCTCTAAA
>SRR4252619_11228976___Sbg5
GGTGTACAACTTCTGATCTCTAAATTG
>SRR4252613_1245665___Sbg5
TGATAGTGATCCGTCCGTCGGGTGGGG
>SRR4252606_665172___Sbg5
TTGGTGGTGTACAACTTCTGATCACTAAA
>SRR4252606_7781767___Sbg5
ATCCGTACACATACACAATGATCAAATA
>SRR4252605_4866065___Sbg5
GAGTGGTGGTGTACAACTTCTGATCACTAA
>SRR4252609_11366914___Sbg5
CCCCTTGGTGTTTTTCGAAAGGAGTAGGC
>SRR4252611_8337573___Sbg5
TTGATAGTGATCCGTCCGTCGGATGGGGAT
>SRR4252605_12286310___Sbg5
TCCTCCTATCCGCACACATACACAATGA
>SRR4252612_2356158___Sbg5
GAGTAATTAATTAAAATTTCGAATCCTG
>SRR4252618_5208841___Sbg5
TACCTTCTCCCTCTTTCATATCCATAATCG
>SRR4252611_10077376___Sbg5
ATATCCATAATCGTCACTCACATCATACC
>SRR4252610_13097909___Sbg5
AAGTGGTGGTATACAACTTCTGATCACTAA
>SRR4252606_399097___Sbg5
AGAGTGGTGGTGTACAACTTCTGATCACTA
>SRR4252611_10701231___Sbg5
AGAGTGGTGGTGTACAACTTCTGATCACTAA
>SRR4252612_7827142___Sbg5
AGAGTGGTGGTGTACAACTTCTGATCACTAA
>SRR4252608_5971899___Sbg5
TATTTCATTGTTGATAGTGATCCGTCCGTC
>SRR4252605_5185392___Sbg5
CAAACTCCTCCGCAATGACTATGTTGAGA
>SRR4252613_3521351___Sbg5
CAGTCCCCTTGGTGCTTTTCGAAAGGA
>SRR4252610_5291002___Sbg5
TACAACTTCTGATCACTAAATTGTGT
>SRR4252608_2644838___Sbg5
TACACACACTCATCCTCCTATCCGCACA
>SRR4252610_2834508___Sbg5
TCGGCTGTGGGGATCAGCGTAGTACTGAC
>SRR4252621_1423798___Sbg5
GAGTGGTGGTGTACAACTTCTGATCACTAA
>SRR4252605_119719___Sbg5
GAGTGGTGGTGTACAACTTCTGATCACTAA
>SRR4252605_11264341___Sbg5
GGGCATATGTCATTGTTGATAGTGATCCG
>SRR4252611_2983991___Sbg5
GCCGGTCCCCTTGGTGCTTTTCGAAAGGA
>SRR4252611_10646447___Sbg5
AGAGTGGTGGTGTACAACTTCTGATCACTAA
>SRR4252611_1487908___Sbg5
AGAGTGGTGGTGTACAACTTCTGATCACTA
>SRR4252612_1960963___Sbg5
AGAGTGGTGGTGTACAACTTCTGATCACTAA
>SRR4252612_6374342___Sbg5
GAGTGGTGGTGTACAACTTCTGATCACTAA
>SRR4252621_11258776___Sbg5
ATCCTCCTATCCGCACACATACACAAT
>SRR4252615_13090495___Sbg5
ACACTCATCCTTCTATCCGCACACATACACA
>SRR4252624_202936___Sbg5
CAGCCGCTAGGTAAGCCTTGGCCCACATA
>SRR4252611_4013664___Sbg5
TAGTGATCCGTCCGTCGGATGGGGACGTT

>SRR4252606_9415603___Sbg5
TCATCCTTCTATCCGCACACATACACA
>SRR4252608_12069685___Sbg5
ATCCTTCTATCCGCACACATACACAATGA
>SRR4252612_3928050___Sbg5
CCTTCTATCCGCACACATACACAATGA
>SRR4252612_8002316___Sbg5
CACACTTACACACCGATCCATTAACTGG
>SRR4252612_5080535___Sbg5
TACAACTTCTGATCACTAAATTGTACCT
>SRR4252612_949700___Sbg5
TAATATGTCATTGTTGATAGTGATCCGTC
>SRR4252623_4627520___Sbg5
CACTCATCCCCCTATTCACACACTTACA
>SRR4252612_3181160___Sbg5
CCTTGGCCCACATAGGGCTGTTGAGCT
>SRR4252608_8449341___Sbg5
TGTACAACTTCTGATCACTAAATTGT
>SRR4252611_424722___Sbg5
TGCTTTTCGAAAGGAGTAGGCTACGTG
>SRR4252613_4881709___Sbg5
TGCTTTTCGAAAGGAGTAGGCTACGTG
>SRR4252608_9104773___Sbg5
TACACACACTCATCCTCCTATCCGCACA
>SRR4252611_3717925___Sbg5
CACACTCATCCTCCTATCCGCACACAC
>SRR4252621_13843522___Sbg5
GAGTTGTGGTGTACAACTTCTGATCACTAA
>SRR4252609_10133555___Sbg5
AAGAAGGCATATGTCATTGTTGATAGTGAT
>SRR4252611_9414124___Sbg5
CCGGTTTTCACCTTCTCCCTCTTTCATA
>SRR4252610_7856349___Sbg5
GAGTGGTGGTGTACAACTTCTGATCACTAA
>SRR4252610_8065731___Sbg5
GAGTGGTGGTGTACAACTTCTGATCACTAA
>SRR4252610_387726___Sbg5
TGTACAACTTCTGATCACTAAATTGTGC
>SRR4252611_12952797___Sbg5
TATCCGCACACATACACAATGATCAAATA
>SRR4252610_9227634___Sbg5
CCGCACACATACACAATGATCAAATA
>SRR4252606_6056426___Sbg5
ATCCGCACACATACACAATGATCAAATA
>SRR4252605_8831657___Sbg5
TGGTGTACAACTTCTGATCACTAAATAGT
>SRR4252605_14984525___Sbg5
GAGAACTCCTCCGCAATGACTATGTTGAGA
>SRR4252605_7768522___Sbg5
TCATATCCATAATCGTCACTCACATCATA
>SRR4252611_9405431___Sbg5
TTCATATCCATAATCGTCACTCACGTCAT
>SRR4252619_7007261___Sbg5
TAGAAAACCGGGGTTATCGGCTGTGGGA
>SRR4252605_3496463___Sbg5
GGGGTTATCGGCTGTGGGGACCAGCGT
>SRR4252605_3456545___Sbg5
AGAGTGGTGGTGTACAACTTCTGATCACTA
>SRR4252607_14321420___Sbg5
AGAGTGGTGGTGTACAACTTCTGATCACTAA
>SRR4252624_3402867___Sbg5
AGAGTGGTGGTGTACAACTTCTGATCACTAA
>SRR4252623_2517151___Sbg5
ACACTCATCCCCCTATTCACACACTTACA
>SRR4252609_964190___Sbg5
CAATGATCAAATAGCTGGTCTAGAAAACTG
>SRR4252613_1346875___Sbg5
TCACTCATATCATACCACACCACACA
>SRR4252610_13348970___Sbg5
CCCTCCTATCCGCACACATACACAATGA

```
>SRR4252625_11681365___Sbg5
CCCTCCTATCCGCACACATACACAATGA
>SRR4252605_12752637___Sbg5
ATATCCATAATCGTCACTCACATCAT
>SRR4252610_8503139___Sbg5
TGTACAACTTCTGATCACTAAATTGT
>SRR4252611_13699779___Sbg5
GAGAAGGCATACGTCATTGTTGATAGTGA
>SRR4252624_5722415___Sbg5
TCATATTCACTTTCGTCACTCACATCATA
>SRR4252618_10678076___Sbg5
ATCCCGAACTCCTCCGCAATGACTATGTA
>SRR4252612_3407976___Sbg5
TCGGCTGTGGGGACCAGCATAGTACTGAC
>SRR4252619_872878___Sbg5
CCGATCACTAAATTGTACACCAAATTCA
>SRR4252609_11919239___Sbg5
TGCTTTTCGAAAGGAGTAGGCTACATGT
>SRR4252610_6758703___Sbg5
GAGTGGTGGTGTACAACTTCTGATCACTAA
>SRR4252613_7945886___Sbg5
CCATATTACCCCCCGTTAATTCCGTCCTA
>SRR4252612_6248866___Sbg5
CACCGGGTTTAACCTTCTCCCTCTTTCAT
>SRR4252620_5114476___Sbg5
ACCGGGTTTAACCTTCTCCCTCTTTCATA
>SRR4252622_5217670___Sbg5
CACTCATCCTCCTATCCGCACACATACA
>SRR4252619_1304488___Sbg5
TGGAGTGGTGGTGTACAACTTCTGATCACT
>SRR4252620_10333726___Sbg5
TGGAGTGGTGGTGTACAACTTCTGATCACTA
>SRR4252605_7773276___Sbg5
TGCTTTTCGAAAGGAGTAGGCTACATGC
>SRR4252605_8591656___Sbg5
ACCATACTACCCCCCGTTAATTCCGTCCTA
>SRR4252606_10312870___Sbg5
GTCCTATCCGCACACATACACAATGA
>SRR4252619_11178888___Sbg5
AACCGCTCGGTAAGCCTTGGCCCACATC
>SRR4252606_3087014___Sbg5
TCCTTCTATCCGCACACATACACAATGA
>SRR4252608_8510785___Sbg5
TCCTTCTATCCGCACACATACACAATGA
>SRR4252621_6296676___Sbg5
ACGGTCCCCTTGGTGCTTTTCGAAAGGA
>SRR4252611_8228646___Sbg5
ACACTCATCCCCCTATTCACACACTTACA
>SRR4252608_2381904___Sbg5
CCGAACTCTTCCGCAATGACTATGTTGAGA
>SRR4252625_3800930___Sbg5
TATGGCAGCAGCCGCTCGGTAAGCCTTGG
>SRR4252619_2318784___Sbg5
TCGGTAAGCCTTGGCCCACATCGGACTGT
>SRR4252621_956264___Sbg5
CCATACTACCCCCCGTTAATTCCGTCCTA
>SRR4252606_7863695___Sbg5
TGGTGGTGTACAACTTCTGATCACTAA
>SRR4252606_9144095___Sbg5
GAGTGGTGGTGTACAACTTCTGATCACTA
>SRR4252607_1690565___Sbg5
TGCTTTTCGAAAGGAGTAGGTTACATGC
>SRR4252606_6708705___Sbg5
CACACACTCATCCTCCTATCCGCACACA
>SRR4252611_14948978___Sbg5
CCTCCTATCCGCACACATACACAATGA
>SRR4252611_8808677___Sbg5
GAGTGGTGGTGTACAACTTCTGATCACTAA
>SRR4252611_3166576___Sbg5
ACATCCATAATCGTCACTCACATCATA
```

>SRR4252622_966419___Sbg5
TCCGTCCGTCGGATGGGGACGTTAAGCTT
>SRR4252623_5677367___Sbg5
TCCGTCCGTCGGATGGGGACGTTAAGCTT
>SRR4252605_9041026___Sbg5
TGGTGTACAACTTCTGATCACTAAATTGC
>SRR4252605_5063020___Sbg5
AGAAGGCATATGTCATTGTTGATAGTGA
>SRR4252611_14579250___Sbg5
ATGGTGTTTTTCGAAAGGAGTAGGCTACAT
>SRR4252611_9066736___Sbg5
CCGGGTTTCACCTTCTCCCTCTTTCATA
>SRR4252608_9012786___Sbg5
ACACACTCATCCTCCTATCCGCACACATA
>SRR4252606_4443602___Sbg5
CATCCTCCTATCCGCACACATACACAA
>SRR4252608_6381995___Sbg5
CCTCCTATCCGCACACATACACAATGA
>SRR4252610_4663100___Sbg5
GCCGGTCCCCTTGGTGCTTTTCGAAAGGA
>SRR4252610_4754703___Sbg5
AAGTGGTGGTGTACAACTTCTGATCACTAA
>SRR4252624_224347___Sbg5
AAGTGGTGGTGTACAACTTCTGATCACTAA
>SRR4252613_2239266___Sbg5
CTCACATCATACCACACCACACACACA
>SRR4252611_1951225___Sbg5
TCACATCATACCACACCACACACACAC
>SRR4252606_7008632___Sbg5
ATCAGCACACATACACAATGATCAAAT
>SRR4252607_1299563___Sbg5
ATCAGCACACATACACAATGATCAAATA
>SRR4252607_8155751___Sbg5
CCATACTACCCCCCGTTAATTCCGTCCTA
>SRR4252605_13610418___Sbg5
ACACACATCCTCCTATCCACACACTTACA
>SRR4252605_5919850___Sbg5
ACACACATCCTCCTATCCACACACTTACA
>SRR4252609_14906243___Sbg5
ACACACATCCTCCTATCCACACACTTACA
>SRR4252606_7258057___Sbg5
CACACATCCTCCTATCCACACACTTACA
>SRR4252611_9515758___Sbg5
ACACATCCTCCTATCCACACACTTACA
>SRR4252611_501368___Sbg5
CACATCCTCCTATCCACACACTTACA
>SRR4252609_5002721___Sbg5
GAACTCCTCTGCAATGACTATGTTGAGA
>SRR4252605_2648427___Sbg5
TTGATAGTGATCCGTCCGTCGGATGGGGAC
>SRR4252611_8458947___Sbg5
GAGTGGTGGTGTACAACTTCTGATCACTAA
>SRR4252610_13466305___Sbg5
TCAGGTTTCACCTTCTCCCTCTTTCATA
>SRR4252610_8074795___Sbg5
TCAGGTTTCACCTTCTCCCTCTTTCATA
>SRR4252612_2147301___Sbg5
TCAGGTTTCACCTTCTCCCTCTTTCATA
>SRR4252625_3349107___Sbg5
TCAGGTTTCACCTTCTCCCTCTTTCATA
>SRR4252610_13530734___Sbg5
GGAGTGGTGTACAACTTCTGATCACTAAA
>SRR4252605_1245642___Sbg5
TATGTTGAGAAGGCATATGTCATTGTTGT
>SRR4252611_5197855___Sbg5
ACTCATCCTCCTATCCGCACACATACA
>SRR4252623_12586815___Sbg5
GCGGTGGTGTACAACTTCTGATCACTAA
>SRR4252609_15566180___Sbg5
TGTACAACTTCTGATCACTAAATTGT

>SRR4252612_8984214___Sbg5
ACACACTTACACACCAATCCATTAGCT
>SRR4252610_540163___Sbg5
TAGTGGTGGTGTACAACTTCTGATCACTA
>SRR4252610_6622591___Sbg5
TAGTGGTGGTGTACAACTTCTGATCACTAA
>SRR4252612_74493___Sbg5
TAGTGGTGGTGTACAACTTCTGATCACTAA
>SRR4252609_15467625___Sbg5
CCTCCTATCCGCACACATACACAATGA
>SRR4252611_9808072___Sbg5
ATCCGCACACATACACAATGATCAAATA
>SRR4252610_8460290___Sbg5
GAGTGGTGGTGTACAACTTCTGATCACTAA
>SRR4252609_10137144___Sbg5
CCTGGTTTCACCTTCTCCCTCTTTCATA
>SRR4252608_62734___Sbg5
TGGTGGTGTACAACTTCTGATCACTAAATT
>SRR4252610_8745543___Sbg5
TGCTTTTCGAAAGGAGTAGGCTAGATG
>SRR4252620_3605152___Sbg5
TGTCCGCACACATACACAATGATCAAATA
>SRR4252606_7470000___Sbg5
GTCCGCACACATACACAATGATCAAATA
>SRR4252608_3855174___Sbg5
GTCCGCACACATACACAATGATCAAATA
>SRR4252610_12730693___Sbg5
GTCCGCACACATACACAATGATCAAA
>SRR4252610_3418494___Sbg5
GTCCGCACACATACACAATGATCAAATA
>SRR4252610_8251249___Sbg5
GTCCGCACACATACACAATGATCAAATA
>SRR4252611_8114179___Sbg5
GTCCGCACACATACACAATGATCAAATA
>SRR4252610_5093044___Sbg5
GGAGTGGTGGTGTACAACTTCTGATCACTAA
>SRR4252606_330537___Sbg5
CTTTCATATCCACTTTCGTCACTCACATC
>SRR4252612_2574858___Sbg5
ATCCGCACACATACACAATGATCAAATA
>SRR4252608_12512829___Sbg5
AAGTGGTGGTGTACAACTTCTGATCACTAA
>SRR4252608_13063887___Sbg5
AAGTGGTGGTGTACAACTTCTGATCACTAA
>SRR4252610_2050459___Sbg5
AAGTGGTGGTGTACAACTTCTGATCACTAA
>SRR4252619_681408___Sbg5
AGTGATCCGTCCGTCGGATGGGGACGT
>SRR4252613_11356527___Sbg5
TGATCAAATAGCTGGTCTAGAAAACCTGG
>SRR4252610_4949411___Sbg5
CCAGGTTTCACCTTCTCCCTCTTTCATA
>SRR4252610_1208961___Sbg5
GAGTGGTGGTGTACAACTTCTGATCACTAA
>SRR4252612_2322555___Sbg5
TGGTCTAGAAAACCGGGGTTATCGGCTGT
>SRR4252612_1210336___Sbg5
GGAGTGGTGGTGTACAACTTCTGATCACT
>SRR4252623_1234699___Sbg5
AGTGGTGGTGTACAACTTCTGATCACTAA
>SRR4252608_10051012___Sbg5
TCCTCCTATCCGCACACATACACAATGA
>SRR4252611_7232683___Sbg5
GTGGTGTACAACTTCTGATCACTAAATTGT
>SRR4252610_10742080___Sbg5
GAGTGGTGGTTTACAACTTCTGATCACTAA
>SRR4252620_8418544___Sbg5
GTGGTGGTTTACAACTTCTGATCACTAA
>SRR4252610_11771361___Sbg5
ACACTCATCCTCCTATCCGCACACATACA

>SRR4252612_972772___Sbg5
TCCTATCCGCACACATACACAATGAT
>SRR4252610_12888274___Sbg5
TCCTATCCGCACACATACACAATGATCAA
>SRR4252614_3757209___Sbg5
GTGGTGGTGTACAACTTCTGATCACT
>SRR4252606_4597008___Sbg5
ACTCATCCTCCTATCCGCACACATACAC
>SRR4252610_8401832___Sbg5
TCCTATCCGCACACATACACAATGATCA
>SRR4252611_1777297___Sbg5
CCGCACACATACACAATGATCAAATA
>SRR4252607_14725534___Sbg5
TATGTTGAGAAGGCATATGTCATTGT
>SRR4252605_5732665___Sbg5
CCGGACCCCTTGGTGCTTTTCGAAAGGA
>SRR4252612_6063052___Sbg5
TCGGCTGTGGGGACCAGCATAGTACTGAC
>SRR4252619_6354080___Sbg5
CCTGAGACTAGGACGAGTGGTGGTGTACA
>SRR4252611_1761066___Sbg5
TCCTATCCGCACACATACACAATGATCA
>SRR4252606_1408956___Sbg5
ATCCGCACACATACACAATGATCAAATA
>SRR4252611_11388890___Sbg5
ATCCGCACACATACACAATGATCAAATA
>SRR4252607_13420256___Sbg5
TATGTTGAGAAGGCATATGTCATTGC
>SRR4252619_6430815___Sbg5
TGAGAAGGCATATGTCATTGCTGATAGTG
>SRR4252625_663264___Sbg5
TTGATAGTGATCCGTCCGTCGGATGGGG
>SRR4252619_11397758___Sbg5
TGATCCGTCCGTCGGATGGGGACGTTA
>SRR4252616_4435648___Sbg5
TGATCAAATAGCTGGTCTAGAAAACCTGG
>SRR4252616_9593907___Sbg5
TCCTGGGTTAAATCCCGAACTCCTCCGCAAT
>SRR4252616_5663048___Sbg5
TCCCGAACTCCTCCGCAATGACTATGTTT
>SRR4252610_10426052___Sbg5
GTACACACACTCATCCTCCTATCCGCACA
>SRR4252606_8249520___Sbg5
AACACTATACACACACTCATCCTCCTA
>SRR4252605_4536642___Sbg5
TTGATAGTGATCCGTCCGTCGGATGGGGAT
>SRR4252609_10463002___Sbg5
TTGATAGTGATCCGTCCGTCGGATGG
>SRR4252611_11404910___Sbg5
TTGATAGTGATCCGTCCGTCGGATGGGGAT
>SRR4252606_4124285___Sbg5
GAGTGGTGGTGTACAACTTCTGATCACTAA
>SRR4252606_1585956___Sbg5
CTCTTTCATATCCATAATCGTCACTCACAT
>SRR4252611_2065232___Sbg5
TTCATATCCATAATCGTCACTCACAT
>SRR4252616_8064873___Sbg5
GGTGGTGTACAACTTCTGATCACTAA
>SRR4252609_15403968___Sbg5
GTTCCGTCCTATGGCGGCAGCCGCTCGGTA
>SRR4252610_9780686___Sbg5
GTTCCGTCCTATGGCGGCAGCCGCTCGGTA
>SRR4252619_14542915___Sbg5
TTCGAATCCTGAGACTAGGAGGTGTGGT
>SRR4252611_9998629___Sbg5
GCCGGGTTTCACCTTCTCCCTCTTTCATA
>SRR4252605_13369154___Sbg5
ATGTAGAGAAGGCATATGTCATTGTT
>SRR4252610_13062815___Sbg5
TCCTATCCGCACACATACACAATGATC

>SRR4252610_202832___Sbg5
ATCCGCACACATACACAATGATCAAATA
>SRR4252612_2180005___Sbg5
TGAGTGGTGGTGTACAACTTCTGATCACTA
>SRR4252621_13117363___Sbg5
GGTGTACAACTTCTGATCACTAAATTTT
>SRR4252619_2832822___Sbg5
GTGTACAACTTCTGATCACTAAATTTT
>SRR4252616_13459331___Sbg5
TTCCGCCCTATGGCGGCAGCCGCTCA
>SRR4252608_6699126___Sbg5
GCCGGTCCCCTTGGTGCTTTTCGAAAGGA
>SRR4252610_7477833___Sbg5
CATATCCATAATCGTCACTCACATCATA
>SRR4252612_3685578___Sbg5
AAGAGTGGTGGTGTACAACTTCTGATCACTA
>SRR4252606_5697494___Sbg5
AGAGTGGTGGTGTACAACTTCTGATCACTA
>SRR4252611_5415770___Sbg5
TCATATCCATAATCGTCACTCACATCAT
>SRR4252619_6742855___Sbg5
TCGGCTGTGGGGATCAGCGTAGTACTGG
>SRR4252610_11886499___Sbg5
ATTCCGCCCTATGGCGGCAGCCGCTCGGTA
>SRR4252609_8145159___Sbg5
GAGTAATTAATTAAAATTTTGAATCCTGA
>SRR4252607_7970641___Sbg5
TTCATATCCATAATCGTCACTCACATCAT
>SRR4252612_2298659___Sbg5
GCTCCTATCCGCACACATACACAATGA
>SRR4252613_6530139___Sbg5
CCTCTTTCATATTCACTTTCGTCACTCA
>SRR4252610_1755724___Sbg5
ACACACTCATCCTCCTATCCACACACTTA
>SRR4252605_15185173___Sbg5
GAGTGGTGGTTTACAACTTCTGATCACTAA
>SRR4252613_8605607___Sbg5
TCCTCCTATCCGCACACATACACAATGA
>SRR4252606_9228960___Sbg5
TCCCTCTTTCATATCCATAATCGTCA
>SRR4252605_600185___Sbg5
CTCCTATCCGCACACATACACAATGATCA
>SRR4252613_335169___Sbg5
AGCTGGTCTACAAAACCGGGGTTATCGGC
>SRR4252615_7148017___Sbg5
GCTGGTCTACAAAACCGGGGTTATCGG
>SRR4252615_7197435___Sbg5
GCTGGTCTACAAAACCGGGGTTATCGG
>SRR4252613_222659___Sbg5
TAATTAATTAAAATTTTGAATCCTGAG
>SRR4252618_4038546___Sbg5
GTGGTGTACAACTTCTGATCACTAAATC
>SRR4252614_8311961___Sbg5
TATTCACACACTTACACACCGATCCATTAA
>SRR4252605_7010296___Sbg5
GTCCTATTGCGGCAGCCGCTCGGTAAGCCTT
>SRR4252610_9146425___Sbg5
TGTACAACTTCTGATCACTAAATTGAACA
>SRR4252624_4312377___Sbg5
TGTACAACTTCTGATCACTAAATTGA
>SRR4252608_12019149___Sbg5
ATCCTTCTATCCGCACACATACACAATGA
>SRR4252623_13522178___Sbg5
TGATAGTGATCCGTCCGTCGGATGGGGAC
>SRR4252624_7892765___Sbg5
TTCTCCTATCCGCACACATACACAATGA
>SRR4252610_5694156___Sbg5
ACATACTACCCCCCGTTAATTCCGTCCTA
>SRR4252614_6221756___Sbg5
ACATACTACCCCCCGTTAATTCCGTCCTA

>SRR4252619_12310665___Sbg5
TACTACCCCCCGTTAATTCCGTCCTA
>SRR4252610_9744370___Sbg5
TAGTGGTGGTGTACAACTTCTGATCACTAA
>SRR4252622_8590298___Sbg5
TAGTGGTGGTGTACAACTTCTGATCACTAA
>SRR4252614_7605697___Sbg5
TGGTGTACAACTTCTGATCACTAAATTG
>SRR4252623_7697796___Sbg5
TTGATAGTGATCCGTCCGTCGGATGG
>SRR4252612_5276774___Sbg5
CCTCCTATCCGCACACATACACAATGA
>SRR4252611_14208250___Sbg5
ATCCGCACACATACACAATGATCAAATA
>SRR4252612_1156180___Sbg5
AAACTCATCCTCCTATCCGCACACATA
>SRR4252608_12099146___Sbg5
CAGCGGTCCCCTTGGTGCTTTTCGAAAGGA
>SRR4252605_4707507___Sbg5
CACCAGGTTTTACCTTCTCCCTCTTTCATA
>SRR4252621_5697587___Sbg5
CCAGGTTTTACCTTCTCCCTCTTTCATA
>SRR4252605_1104569___Sbg5
CTCTTTCATATCCATAATCGTCACTCACAT
>SRR4252610_8484072___Sbg5
TACACACACTCATCCTCCTATCCGCACA
>SRR4252619_410586___Sbg5
GAGTGGTGGTGTACAACTTCTGATCACTAA
>SRR4252610_11826794___Sbg5
TAGTGGTGGTGTACAACTTCTGATCACTAA
>SRR4252614_6836767___Sbg5
TATGTCATTGTTGATAGTGATCCGTCCGTT
>SRR4252616_6726511___Sbg5
GCACTCATCCTCCTATCCGCACACATACACA
>SRR4252610_9543584___Sbg5
CCGAGTTTCACCTTCTCCCTCTTTCATA
>SRR4252616_12940822___Sbg5
GATCCGTCCGTCGGATGGGGACGTTAAGT
>SRR4252605_14328847___Sbg5
ATGATGTGAGTGACGATTATGGATATGAAA
>SRR4252606_6723359___Sbg5
TCCTTTCGAAAAGCACCAAGGGGACCGG
>SRR4252612_1815216___Sbg5
TAGGACGGAATTAACGGGGGGTAGTATG
>SRR4252610_4969409___Sbg5
TATGATGTGAGTGACGATTATGGATATGA
>SRR4252611_3934846___Sbg5
ACAATTTAGTGATCAGAAGTTGTACA
>SRR4252610_2880176___Sbg5
CGCCATAGGACGGAATTAACGGGGGGTAG
>SRR4252610_10363449___Sbg5
TGTGTGTATAGTGTTGTATTGTGTGT
>SRR4252623_4012993___Sbg5
ATAGCTCAACAGCCCTATGTGGGCCAAGGCT
>SRR4252614_1237457___Sbg5
CGAGCGGCTGCCGCCATAGGACGGAATTA
>SRR4252608_9958446___Sbg5
TAGGACGGAATTAACGGGGGGTAGTATGG
>SRR4252610_13157128___Sbg5
TTTAGTGATCAGAAGTTGTACACCACCAC
>SRR4252609_2337847___Sbg5
TCACTATCAACAATGACATATGCCTTCT
>SRR4252616_7058044___Sbg5
TAGTGTTGTATTGTGTGTATGGTGTGGT
>SRR4252610_10176868___Sbg5
TATGATGTGAGTGACGATTATGGATATGAT
>SRR4252620_3592017___Sbg5
TATGATGTGAGTGACGATTATGGATATGA
>SRR4252624_2833899___Sbg5
TATGATGTGAGTGACGATTATGGATATGA

>SRR4252610_9392446___Sbg5
GATTATGGATATGAAAGAGGGAGAAG
>SRR4252610_5521711___Sbg5
TTAGTGATCAGAAGTTGTACACCACCACTC
>SRR4252610_7140834___Sbg5
TTAGTGATCAGAAGTTGTACACCACCACTC
>SRR4252624_1639983___Sbg5
TAGGACGGAATTAACGGGGGGTAGTATGG
>SRR4252605_126503___Sbg5
TATGATGTGAGTGACGATTCTGGATATGA
>SRR4252617_4839044___Sbg5
TGATGTGAGTGACGATTCTGGATATGA
>SRR4252606_693992___Sbg5
TTAGTGATCAGAAGTTGTACACCACCACTC
>SRR4252608_13011708___Sbg5
TCCTTTCGAAAAGCACCAAGGGGACCGGC
>SRR4252610_5741058___Sbg5
TTAGTGATCAGAAGTTGTACACCACCACTC
>SRR4252619_12177883___Sbg5
TAGTGATCAGAAGTTGTACACCACCACTCC
>SRR4252610_5469733___Sbg5
TTAGTGATCAGAAGTTGTACACCACCACTC
>SRR4252609_12395311___Sbg5
TCACTATCAACAATGACATATGCCTTCT
>SRR4252612_2561637___Sbg5
TTCCCCATCCGACGGACGGATCACTATCAA
>SRR4252605_938138___Sbg5
TTAGTGATCAGAAGTTGTACACCACCACTC
>SRR4252623_475400___Sbg5
ACAGCCCTATGTGGGCCAAGGCTTACCGA
>SRR4252616_3294770___Sbg5
GCTGGTCCCCACAGCCGATAGCCCCGGTTT
>SRR4252607_7456068___Sbg5
CAACAATGACATATGCCTTCTCTACATA
>SRR4252612_5900214___Sbg5
TAGGACGGAATTAACGGGGGGTAGTATG
>SRR4252610_12293832___Sbg5
CGGTTTTCTAGACCAGCTAATGGATTGGTG
>SRR4252606_5137801___Sbg5
TTAGTGATCAGAAGTTGTACACCACCACTC
>SRR4252618_9759981___Sbg5
CCCCACAGCCGATAACCCCGGTTTTCTA
>SRR4252625_4773832___Sbg5
CCATCCGACGGACGGATCACTATCAACA
>SRR4252621_12250897___Sbg5
TTAGTGATCAGAAGTTGTACACCACCACTC
>SRR4252611_7971313___Sbg5
TATGAAAGAGGGAGAAGGTGAAACCCGGT
>SRR4252610_902663___Sbg5
TTAGTGATCAGAAGTTGTTCACCACCACTC
>SRR4252613_7932650___Sbg5
TTAGTGATCAGAAGTTGTACACCACCACTC
>SRR4252611_11372825___Sbg5
GTCAGTACTACGCTGGTCCCCACAGCCGA
>SRR4252610_7957366___Sbg5
TAGTCATTGCGGAGGAGTTCGGGATTT
>SRR4252623_3771619___Sbg5
TATGATGTGAGTGACGATTATGGATATGA
>SRR4252608_5815377___Sbg5
TTAGTGATCAGAAGTTGTACACCACCACTC
>SRR4252611_14580441___Sbg5
ATGATGTGAGTGACGATTATGGATATGAA
>SRR4252606_376857___Sbg5
TTAGTGATCAGAAGTTGTACACCACCACTC
>SRR4252607_2725055___Sbg5
CAGTACTACGCTGGTCCCCACAGCCGA
>SRR4252608_2668380___Sbg5
ACAATTTAGTGATCAGAAGTTGTACA
>SRR4252610_2583361___Sbg5
TTAGTGATCAGAAGTTGTACACCACCACTC

>SRR4252610_2077817___Sbg5
ACAATTTAGTGATCAGAAGTTGTACA
>SRR4252608_850884___Sbg5
TATGATGTGAGTGACGATTATGGATATGA
>SRR4252612_6441163___Sbg5
TCCTTTCGAAAAGCACCAAGGGGACCGGC
>SRR4252624_3923170___Sbg5
TCCTTTCGAAAAGCACCAAGGGGACCGG
>SRR4252612_4785719___Sbg5
TATGAAAGAGGGAGAAGGTGAAACCCGG
>SRR4252611_1855266___Sbg5
CCCGGTTTTCTAGACCAGCTAATGGAT
>SRR4252622_10346232___Sbg5
AACGTCCCCATCCGACGGACGGATCACTA
>SRR4252623_12878845___Sbg5
GTCCCCATCCGACGGACGGATCACTATCAA
>SRR4252610_12940896___Sbg5
TAATGGATTGGTGTGTAAGTGTGTGGAT
>SRR4252608_1229860___Sbg5
TGTGTGTATAGTGTTGTATTGTGTGTGTGG
>SRR4252605_1522266___Sbg5
TATGATGTGAGTGACGATTATGGATATG
>SRR4252620_7668031___Sbg5
GGCGGCTTAACGTCCCCATCCGACGGAC
>SRR4252623_9195199___Sbg5
GTCCCCATCCGACGGACGGATCACTATCA
>SRR4252609_11103641___Sbg5
TATGAAAGAGGGAGAAGGTGAAACCCGGT
>SRR4252606_7574129___Sbg5
TTAGTGATCAGAAGTTGTACACCACCACTC
>SRR4252608_7530693___Sbg5
TGAGTGTGTGTATAGTGTTGAATTGTGT
>SRR4252606_8030344___Sbg5
TTAGTGATCAGAAGTTGTACACCACCACTC
>SRR4252605_11057838___Sbg5
CATGTAGCCTACTCCTTTCGAAAAGCA
>SRR4252610_13435160___Sbg5
GACGGACGGATCACTATCAACAATGACA
>SRR4252612_4056963___Sbg5
TAGTGATCAGAAGTTGTACACCACCACC
>SRR4252612_7441113___Sbg5
TGTGTGTGTGGTGTGGTATGATGTGAG
>SRR4252606_10047393___Sbg5
TTAGTGATCAGAAGTTGTACACCACCACTA
>SRR4252610_6611175___Sbg5
TTAGTGATCAGAAGTTGTACACCACCACTA
>SRR4252612_4162943___Sbg5
TTAGTGATCAGAAGTTGTACACCACCACTA
>SRR4252611_2018187___Sbg5
TTAGTGATCAGAAGTTGTACACCACCACTC
>SRR4252618_3021807___Sbg5
AGTATGGTCAGTACTACGCTGGTCCCC
>SRR4252612_2106154___Sbg5
TCCTTTCGAAAAGCACCAAGGGGACCAG
>SRR4252606_8658528___Sbg5
AGCGGCTTAACGTCCCCATCCGACGGACG
>SRR4252621_2753857___Sbg5
TGATGTGAGTGACGATTATGGATATGA
>SRR4252615_10838754___Sbg5
CAACAATGACATATGCCTTCTCTACATA
>SRR4252621_2719582___Sbg5
TTAGTGATCAGAAGTTGTACACCACCACTC
>SRR4252605_3665065___Sbg5
TAGGACGGAATTAACGGGGGGTAGTACGG
>SRR4252619_8056401___Sbg5
GCACAATTTAGTGATCAGAAGTTGTACACCA
>SRR4252611_13495536___Sbg5
TTAGTGATCAGAAGTTGTACACCACCACTCC
>SRR4252611_6257654___Sbg5
TATGAAAGAGGGAGAAGGTGAAACCCGG

>SRR4252622_3180670___Sbg5
TGTGCCGGCATGTAGCCTACTCCTTTCGAA
>SRR4252612_2266532___Sbg5
GTGCACAATTTAGTGATCAGAAGTTGTA
>SRR4252605_8087616___Sbg5
ACAATTTAGTGATCAGAAGTTGTACACCA
>SRR4252626_2260101___Sbg5
TAACGTCCCCATCCGACGGACGGATCAC
>SRR4252613_4783711___Sbg5
TACCGAGCGGCTGCCGCCATAGGACG
>SRR4252612_3874422___Sbg5
TTAGTGATCAGAAGTTGTACACCACCACTC
>SRR4252606_6602862___Sbg5
TATGATGTGAGTGACGATTATGGATATG
>SRR4252609_1655679___Sbg5
TAGGACGGAATTAACGGGGGGTAGTATGG
>SRR4252612_2517140___Sbg5
GATGTGAGTGACGATTATGGATATGAA
>SRR4252612_6498331___Sbg5
TCCTTTCGAAAAGCACCAAGGGGACCG
>SRR4252611_4745088___Sbg5
TTAGTGATCAGAAGTTGTACTCCACCACT
>SRR4252612_7276819___Sbg5
GTCCCCATCCGACGGACGGATCACTATCAA
>SRR4252611_2808958___Sbg5
CATGTAGCCTACTCCTTTCGAAAAGCA
>SRR4252609_974053___Sbg5
TAGGACGGAATTAACGGGGGGTAGTATGG
>SRR4252616_1804205___Sbg5
GATCAGAAGTTGTACACCACCACTCCTCC
>SRR4252611_5297641___Sbg5
GCATGTAGCCTACTCCTTTCGAAAAGCA
>SRR4252608_5709371___Sbg5
ACAATTTAGTGATCAGAAGTTGTACA
>SRR4252606_346931___Sbg5
TAGTGATCAGAAGTTGTACACCACCACTC
>SRR4252607_12493432___Sbg5
TCAGTACTACGCTGGTCCCCACAGCCGA
>SRR4252608_11243492___Sbg5
CAACAATGACATATGCCTTCTCTACATA
>SRR4252611_12323123___Sbg5
TATGAAAGAGGGAGAAGGTGAAACCCGG
>SRR4252611_6756449___Sbg5
TACCGAGCGGCTGCCGCCATAGGACGGA
>SRR4252621_651272___Sbg5
TAGGACGGAATTAACGGGGGGTAGTATGG
>SRR4252624_2094951___Sbg5
TGGATATGAAAGAGGGAGAAGGTGAAAC
>SRR4252611_6220210___Sbg5
TGATGTGAGTGACGATTATGGATATG
>SRR4252606_2136381___Sbg5
TTAGTGATCAGAAGTTGTACACCACCACTC
>SRR4252609_11397914___Sbg5
TTAGTGATCAGAAGTTGTACACCACCACT
>SRR4252605_10493772___Sbg5
TAGGACGGAATTAACGGGGGGTAGTATG
>SRR4252619_9202958___Sbg5
TATGGTCAGTACTACGCTGGTCCCCACA
>SRR4252610_4568378___Sbg5
ATGATGTGAGTGACGATTATGGATATGAA
>SRR4252605_8712561___Sbg5
TAGGACGGAATTAACGGGGGGTAGTATGG
>SRR4252608_6207607___Sbg5
TCAGTACTACGCTGGTCCCCACAGCCGA
>SRR4252605_5706325___Sbg5
TAAGTGTGTGGATAGGGGGATGAGTGTGT
>SRR4252616_13830915___Sbg5
CCCCACAGCCGATAACCCCGGTTTTCTA
>SRR4252608_1598459___Sbg5
GCACAATTTAGTGATCAGAAGTTGTA

>SRR4252622_3153357___Sbg5
TTAGTGATCAGAAGTTGTACACCACCACTC
>SRR4252605_9172821___Sbg5
TAGGACGGAATTAACGGGGGGTAGTATGG
>SRR4252624_2716758___Sbg5
TATGAAAGAGGGAGAAGGTGAAACCCGG
>SRR4252607_13887478___Sbg5
GCAATTTAGTGATCAGAAGTTGTACACCA
>SRR4252608_5894927___Sbg5
GCAATTTAGTGATCAGAAGTTGTACA
>SRR4252610_10777777___Sbg5
GCAATTTAGTGATCAGAAGTTGTACACCA
>SRR4252608_1613056___Sbg5
TGCCTTCTCTACATAGTCATTGCGGA
>SRR4252610_13019228___Sbg5
TAGGACGGAATTAACGGGGGGTAGTATGG
>SRR4252605_13739078___Sbg5
TAGGACGGAATTAACGGGGGGTAGTATGG
>SRR4252611_3406858___Sbg5
TAGGACGGAATTAACGGGGGGTAGTATG
>SRR4252606_4330987___Sbg5
TATGATGTGAGTGACGATTATGGATATG
>SRR4252608_7295743___Sbg5
CATGTAGCCTACTCCTTTCGAAAAGCA
>SRR4252615_14766675___Sbg5
GTCCCCATCCGACGGACGGATCACTATCAA
>SRR4252610_4818927___Sbg5
TTAGTGATCAGAAGTTGTACACCACCACTC
>SRR4252622_1456637___Sbg5
TATGGATATGAAAGAGGGAGAAGGTGAAA
>SRR4252621_6308722___Sbg5
TATGAAAGAGGGAGAAGGTGAAACCT
>SRR4252606_4100427___Sbg5
TTAGTGATCAGAAGTTGTACACCACCACTC
>SRR4252610_7295539___Sbg5
TTAGTGATCAGAAGTTGTAGACCACCACTC
>SRR4252611_584292___Sbg5
TGATGTGAGTGACGATTATGGATATGAAA
>SRR4252606_9519780___Sbg5
TTAGTGATCAGAAGTTGTACACCACCACTC
>SRR4252610_8218236___Sbg5
TAGTGATCAGAAGTTGTACACCACCACTCC
>SRR4252621_13298837___Sbg5
TCAACAATGACATATGCCTTCTCTACATA
>SRR4252606_6070679___Sbg5
GATGTGAGTGACGATTATGGATATGAAAG
>SRR4252612_9026831___Sbg5
GTGTATAGTGTTGTATTGTGTGTGTGGTGT
>SRR4252611_9459903___Sbg5
TAGTCATTGCGGAGGAGTTCGGGATT
>SRR4252611_5392956___Sbg5
CAGTACTATGCTGGTCCCCACAGCCGA
>SRR4252611_10599649___Sbg5
TTAGTGATCAGAAGTTGTACACCACCACTC
>SRR4252624_7473373___Sbg5
TAATGGATCGGTGTGTAAGTGTGTGGATA
>SRR4252612_3324148___Sbg5
TATGATGTGAGTGACGATTATGGATATGA
>SRR4252622_2035160___Sbg5
TTAGTGATCAGAAGTTGTACACCACCACC
>SRR4252615_14712192___Sbg5
GTCCCCATCCGACGGACGGATCACTATCAA
>SRR4252610_11748600___Sbg5
TTGGTGTGTAAGTGTGTGGATAGGGGGA
>SRR4252606_2085515___Sbg5
TTAGTGATCAGAAGTTGTACACCACCACTC
>SRR4252620_3090116___Sbg5
ACGTCCCCATCCGACGGACGGATCAC
>SRR4252612_2859990___Sbg5
ACGGACGGATCACTATCAACAATGACAT

>SRR4252622_7502856___Sbg5
CATCCGACGGACGGATCACTATCAACA
>SRR4252619_8045129___Sbg5
TAGTGATCAGAAGTTGTACACCACCACTC
>SRR4252618_5994085___Sbg5
AGTGATCAGAAGTTGTACACCACCACTCCT
>SRR4252620_5847949___Sbg5
CGGCTTAACGTCCCCATCCGACGGACGGA
>SRR4252620_8895639___Sbg5
CGGCTTAACGTCCCCATCCGACGGACG
>SRR4252610_12637242___Sbg5
TAGGACGGAATTAACGGGGGGTAGTAG
>SRR4252623_953987___Sbg5
TAGGACGGAATTAACGGGGGGTAGTAGGG
>SRR4252610_3467289___Sbg5
TAATGGATCGGTGTGTAAGTGTGTGGAT
>SRR4252611_5580697___Sbg5
CCCCATCCGACGGACGGATCACTATCAA
>SRR4252607_12340604___Sbg5
TAGTGATCAGAAGTTGTACACCACCACTC
>SRR4252618_2799285___Sbg5
TGAAACCCGGTGCCGGCATGTAGCCTAC
>SRR4252605_5902219___Sbg5
TAGGACGGAATTAACGGGGGGTGGTATGG
>SRR4252611_6067744___Sbg5
CCCCATCCGACGGACGGATCACTATCAA
>SRR4252607_9171194___Sbg5
ACAATTTAGTGATCAGAAGTTGTACACCA
>SRR4252612_8651471___Sbg5
TGGATATGAAAGAGGGAGAAGGTGAAACCT
>SRR4252610_9157777___Sbg5
TATGAAAGAGGGAGAAGGTGAAACCT
>SRR4252624_445497___Sbg5
TATGAAAGAGGGAGAAGGTGAAACCTGG
>SRR4252612_1528109___Sbg5
TAGTGATCAGAAGTTGTACACCACCACTC
>SRR4252605_4772952___Sbg5
AGGATGAGTGTGTGTATAGTGTTGTAT
>SRR4252611_9383734___Sbg5
GGACGGATCACTATCAACAATGACAT
>SRR4252611_6918876___Sbg5
TTAGTGATCAGAAGTTGTACACCACCACC
>SRR4252607_9212707___Sbg5
TCACTATCAACAATGACATATGCCTTCT
>SRR4252610_9546185___Sbg5
TTAGTGATCAGAAGTTGTACACCACCACC
>SRR4252612_7095728___Sbg5
AGTACTACGCTGGTCCCCACAGCCGA
>SRR4252616_11619750___Sbg5
GAAAGAGGGAGAAGGTGAAACCTGGT
>SRR4252613_10387033___Sbg5
TGTGGTATGATGTGAGTGACGATTATGGA
>SRR4252619_13676984___Sbg5
CAGGATTCGAAATTTTAATTAATTACTC
>SRR4252610_3890721___Sbg5
TTAGTGATCAGAAGTTGTACACCACCACTC
>SRR4252622_2019708___Sbg5
CTACATAGTCATTGCGGAGGAGTTCGG
>SRR4252605_3927433___Sbg5
TGAATTTGGTGTACAATTTAGTGATC
>SRR4252610_6396728___Sbg5
TTAGTGATCAGAAGTTGTACACCACCACTC
>SRR4252610_9322516___Sbg5
TTAGTGATCAGAAGTTGTACACCACCACTC
>SRR4252614_7389761___Sbg5
TATGTGAGTGACGATTATGGATATGAAAGAG
>SRR4252611_7785538___Sbg5
ATTTAGTGATCAGAAGTTGTACACCAC
>SRR4252611_4190346___Sbg5
CCATCCGACGGACGGATCACTATCAA

>SRR4252614_6280053___Sbg5
CCATCCGACGGACGGATCACTATCAACA
>SRR4252622_5607160___Sbg5
AGTCAGTACTACGCTGGTCCCCACAGCCG
>SRR4252605_8097322___Sbg5
TAGGACGGAATTAACGGGAGGTAGTATGG
>SRR4252610_1188531___Sbg5
GACGATTATGGATATGAAAGAGGGAGA
>SRR4252610_2470364___Sbg5
TGTAAGTGTGTGAATAGGGGGATGAGTG
>SRR4252606_8078592___Sbg5
TATGATGTGAGTGACGATTTTGGATATGA
>SRR4252606_4442149___Sbg5
GTATGATGTGAGTGACGATTATGGATATGA
>SRR4252625_13936400___Sbg5
ACAATTTAGTGATCAGAAGTTGTACACCA
>SRR4252609_4973616___Sbg5
TTAGTGATCAGAAGTTGTACACCACCACTC
>SRR4252612_6948002___Sbg5
TAGTGATCAGAAGTTGTACACCACCACTCC
>SRR4252621_12075699___Sbg5
CTTAACGTCCCCATCCGACGGACGGATCA
>SRR4252615_2642228___Sbg5
TCATCCGACGGACGGATCACTATCAACA
>SRR4252611_10847553___Sbg5
GCACAATTTAGTGATCAGAAGTTGTACA
>SRR4252612_1645427___Sbg5
TAGTGATCAGAAGTTGTACACCACCACTCC
>SRR4252605_11718502___Sbg5
TATGGTCAGTACTACGCTGGTCCCCACAGC
>SRR4252622_10543979___Sbg5
GTCCCCATCCGACGGACGGATCACTATCAA
>SRR4252605_2893406___Sbg5
AATGTGAGTGACGATTATGGATATGAA
>SRR4252607_3587868___Sbg5
AATGTGAGTGACGATTATGGATATGAAAGAG
>SRR4252609_3678222___Sbg5
TATGATGTGAGTGACGATTATGGATATG
>SRR4252621_6437425___Sbg5
TGTGTGTGGTGTGGTATGATGTGAGTGACG
>SRR4252623_4723685___Sbg5
TTAGTGATCAGAAGTTGTACACCACCACTC
>SRR4252613_3047162___Sbg5
CAACAATGACATATGCCTTCTCTACATA
>SRR4252611_7154256___Sbg5
GTGCACAATTTAGTGATCAGAAGTTGTA
>SRR4252607_12631979___Sbg5
TCACTATCAACAATGACATATGCCTTCT
>SRR4252609_6371541___Sbg5
ACAATTTAGTGATCAGAAGTTGTACACCA
>SRR4252612_224431___Sbg5
GTCCCCATCCGACGGACGGATCACTATCA
>SRR4252612_1054431___Sbg5
TAGGACGGAATTAACGGGGGGTAGTATGG
>SRR4252606_8989627___Sbg5
TCCTTTCGAAAAGCACCAAGGGGACCGGC
>SRR4252616_12649271___Sbg5
ACGGACGGATCACTATCAACAATGACATA
>SRR4252610_3088533___Sbg5
TGCACAATTTAGTGATCAGAAGTTGTA
>SRR4252610_174002___Sbg5
TTAGTGATCAGAAGTTGTACACCACCACTC
>SRR4252621_6983009___Sbg5
ATGATGTGAGTGACGATTATGGATATGAA
>SRR4252619_2611459___Sbg5
TATGATGTGAGTGACGATTATGGATATG
>SRR4252623_13082551___Sbg5
TACCGAGCGGCTGCCGCCATAGGACGGAA
>SRR4252608_5229783___Sbg5
TGTAAGTGTGTGGATAGGAGGATGAGTG

>SRR4252610_2001364___Sbg5
TAGGACGGAATTAACGGGGGGTAGTATGG
>SRR4252608_10964654___Sbg5
CAGCCGATAACCCCGGTTTTCTAGACCA
>SRR4252611_1133954___Sbg5
TGAAACCCGGTGCCGGCATGTAGCCTACT
>SRR4252610_5699925___Sbg5
CAACAATGACATATGCCTTCTCTACATA
>SRR4252615_10703752___Sbg5
CCCCATCCGACGGACGGATCACTATC
>SRR4252619_14612568___Sbg5
ATAGCTCAACAGCCCTATGTGGGCCAA
>SRR4252612_8925606___Sbg5
TACCGAGCGGCTGCCGCCATAGGACGG
>SRR4252605_11787119___Sbg5
GAAGTTGTACACCACCACTCCTCCTAGTCTC
>SRR4252618_9564204___Sbg5
ACTATCAACAATGACATATGCCTTCTCT
>SRR4252610_11167092___Sbg5
AGCTAATGGATCGGTGTGTAAGTGTG
>SRR4252605_4188639___Sbg5
TCCTTTCGAAAAGCACCAAGGGGACCGGC
>SRR4252610_9256917___Sbg5
AGCTAATGGATTGGTGTGTAAGTGTGT
>SRR4252611_3189189___Sbg5
ATGAGTGACGATTATGGATATGAAAGAG
>SRR4252611_9067376___Sbg5
TATGAAAGAGGGAGAAGGTGAAACCCGG
>SRR4252619_13547972___Sbg5
ACAATTTAGTGATCAGAAGTTGTACA
>SRR4252612_190620___Sbg5
TAGTGATCAGAAGTTGTACACCACCACTC
>SRR4252608_5758385___Sbg5
TATGATGTGAGTGACGATTATGGATATG
>SRR4252610_2991930___Sbg5
TTAGTGATCAGAAGTTGTACACCACCACTC
>SRR4252606_7600066___Sbg5
GACGGACGGATCACTATCAACAATGACA
>SRR4252610_6101110___Sbg5
ACACAATTTAGTGATCAGAAGTTGTACA
>SRR4252612_2626003___Sbg5
AGTGATCAGAAGTTGTACACCACCAC
>SRR4252615_1676888___Sbg5
TGCCTTCTCTACATAGTCATTGCGGA
>SRR4252605_10765426___Sbg5
GTGTATAGTGTTGTATTGTGTATGTGGT
>SRR4252616_6197550___Sbg5
TATGATGTGAGTGACGATTATGGATATGA
>SRR4252606_3785422___Sbg5
TTAGTGATCAGAAGTTGTACACCACCACTC
>SRR4252610_13496158___Sbg5
TATGATGTGAGTGACGATTATGGATATGT
>SRR4252607_9594041___Sbg5
CCCCATCCGACGGACGGATCACTATCAA
>SRR4252612_6319272___Sbg5
TTAGTGATCAGAAGTTGTACACCACCACTC
>SRR4252606_7032580___Sbg5
TCTATAGCTCAACAGCCCTATGTGGGCCA
>SRR4252606_7871050___Sbg5
TATAGCTCAACAGCCCTATGTGGGCCA
>SRR4252614_5219283___Sbg5
TAGGACGGAATTAACGGGGGGTAGTATG
>SRR4252608_1472168___Sbg5
TATGCCTTCTCTACATAGTCATTGCGGA
>SRR4252608_12281046___Sbg5
ACAATTTAGTGATCAGAAGTTGTACA
>SRR4252613_2007158___Sbg5
TCCCCACAGCCGATAGCCCCGGTTTTC
>SRR4252621_1590841___Sbg5
CAACAATGACATATGCCTTCTCTACATA

>SRR4252625_8091635___Sbg5
GTGCACAATTTAGTGATCAGAAGTTGTA
>SRR4252623_1067322___Sbg5
TTAGTGATCAGAAGTTGTACACCACCACTC
>SRR4252606_5415261___Sbg5
CAGCTAATGGATCGGTGTGTAAGTGTGTG
>SRR4252620_10024406___Sbg5
TCAGTACTACGCTGGTCCCCACAGCCGATA
>SRR4252610_13493103___Sbg5
GTGTGGTGTGGTATGATGTGAGTGACGATT
>SRR4252621_3862887___Sbg5
GTTGTATTGTGTGTGTGGTGTGGTATGAT
>SRR4252614_2505514___Sbg5
AGTGTTGTATTGTGTGTGTGGTGTGG
>SRR4252611_2534717___Sbg5
TAGGACGGAATTAACGGGAGGTAGTATGG
>SRR4252611_5064330___Sbg5
GACGGATCACTATCAACAATGACATATG
>SRR4252622_6260386___Sbg5
TTAGTGATCAGAAGTTGTACACCACCACTC
>SRR4252614_6267759___Sbg5
GCACAATTTAGTGATCAGAAGTTGTA
>SRR4252609_9219664___Sbg5
CCAGTTAATGGATCGGTGTGTAAGTGTGTG
>SRR4252607_13260596___Sbg5
GTCATTGCGGAGGAGTTCGGGATTTAAC
>SRR4252611_12572334___Sbg5
TATGATGTGAGTGACGATTATGGATAT
>SRR4252613_4007879___Sbg5
TAGGACGGAATTAACGGGGGGTAGTATGGT
>SRR4252606_6950159___Sbg5
TCCCCATCCGACGGACGGATCACTATCAA
>SRR4252612_5743972___Sbg5
AGCCTACTCCTTTCGAAAAACACCAAGGG
>SRR4252610_12459810___Sbg5
TGTGTGTATAGTGTTGTACTGTGTGTGTG
>SRR4252606_583594___Sbg5
TTAGTGATCAGAAGTTGTACACCACCACTT
>SRR4252610_9819682___Sbg5
TTAGTGATCAGAAGTTGTACACCACCACTT
>SRR4252623_800416___Sbg5
TTAGTGATCAGAAGTTGTACACCACCACTT
>SRR4252612_3960445___Sbg5
GCACAATTTAGTGATCAGAAGTTGTACA
>SRR4252611_2275363___Sbg5
TTAGTGATCAGAAGTTGTACACCACCACT
>SRR4252615_8144399___Sbg5
ATGCACAATTTAGTGATCAGAAGTTGTA
>SRR4252612_7392837___Sbg5
TGTGTGTGTGGTGTGGTATGATGTGAGT
>SRR4252610_4756966___Sbg5
TATGAAAGAGGGAGAAGGTGAAACCTGG
>SRR4252613_7756651___Sbg5
TATGAAAGAGGGAGAAGGTGAAACCTGG
>SRR4252605_2347729___Sbg5
GAAGTTGTACACCACCACTCCTCCTAGTCTC
>SRR4252614_1455572___Sbg5
TAATGGATTGGTGTGTAAGTGTGTGGA
>SRR4252605_8419860___Sbg5
TTAGTGATCAGAAGTTGTACACCACCGCTC
>SRR4252620_1199680___Sbg5
TTAGTGATCAGAAGTTGTACACCACCGCTC
>SRR4252608_1343032___Sbg5
GACCAGCTAATGGATTGGTGTGTAAGTG
>SRR4252612_1704862___Sbg5
GACGGACGGATCACTATCAACAATGACAT
>SRR4252605_8988397___Sbg5
CCATCCGACGGACGGATCACTATCAA
>SRR4252605_8028482___Sbg5
ACAATTTAGTGATCAGAAGTTGTACA

>SRR4252614_1529573___Sbg5
TTAGTGATCAGAAGTTGTACACCACCACTCC
>SRR4252608_10782997___Sbg5
TGCCTTCTCTACATAGTCATTGCGGA
>SRR4252605_4758609___Sbg5
CCAATTTAGTGATCAGAAGTTGTACA
>SRR4252611_6772876___Sbg5
TTAGTGATCAGAAGTTGTACACCACCACT
>SRR4252606_2863510___Sbg5
GTGACTGACGATTATGGATATGAAAG
>SRR4252613_8505191___Sbg5
TATGAAAGAGGGAGAAGGTGAAACTCGG
>SRR4252606_534602___Sbg5
TTAGTGATCAGAAGTTGTACACCACCACTC
>SRR4252608_11853893___Sbg5
TTAGTGATCAGAAGTTGTACACCACCACTC
>SRR4252610_10973512___Sbg5
ATGATGTGAGTGACGATTATGGATATGAA
>SRR4252611_6578227___Sbg5
TATGAAAGAGGGAGAAGGTTAAACCCGGTG
>SRR4252611_3418692___Sbg5
TATGAAAGAGGGAGAAGGTGAAACCCGG
>SRR4252620_9559505___Sbg5
TAGGACGGAATTAACGGGGGGTAGTAAGG
>SRR4252621_11614332___Sbg5
TCATTGCGGAGGAGTTCGGGATTTAACC
>SRR4252605_11516653___Sbg5
TATGATGTGAGTGACGATTATGGATATGA
>SRR4252611_13884396___Sbg5
TAGGACGGAATTAACGGGGGGTAGTATGGC
>SRR4252622_10827573___Sbg5
TTAGTGATCAGAAGTTGTGCACCACCACT
>SRR4252620_3236752___Sbg5
TGGTCAGTACTACGCTGGTCCCCACAGCC
>SRR4252621_9106258___Sbg5
AACGTCCCCATCCGACGGACGGTTCACTATC
>SRR4252608_4413167___Sbg5
GGTCAGTACTACGCTGGTCCCCACAGCCG
>SRR4252612_6027872___Sbg5
TAGTACTACGCTGGTCCCCACAGCCGA
>SRR4252606_3340081___Sbg5
CTACACAGTCATTGCGGAGGAGTTCGGGA
>SRR4252619_14140603___Sbg5
ACAGTCCTATGTGGGCCAAGGCTTACCGA
>SRR4252621_8865734___Sbg5
ACAATTTAGTGATCAGAAGTTGTACACCA
>SRR4252606_1622477___Sbg5
TAGTGATCAGAAGTTGTACACCACCACTC
>SRR4252606_1958830___Sbg5
TTAGTGATCAGAAGTTGTACACCACCACTC
>SRR4252613_4632306___Sbg5
CCCCGCAGCCGATAGCCCCGGTTTTCTA
>SRR4252610_11232024___Sbg5
CCCCATCCGACGGACGGATCACTATCAA
>SRR4252614_9526430___Sbg5
TTAGTGATCAGAAGTTGTACACCACCACTC
>SRR4252613_4416191___Sbg5
GTCCCCATCCGACGGACGGATCACTATCA
>SRR4252611_10589014___Sbg5
TTAGTGATCAGAAGTTGTACACCACCACTC
>SRR4252606_9735788___Sbg5
GAAACCTGGTCCCGGCATGTAGCCTAC
>SRR4252609_6668838___Sbg5
GAAACCTGGTCCCGGCATGTAGCCTAC
>SRR4252612_5340704___Sbg5
GAAACCTGGTCCCGGCATGTAGCCTAC
>SRR4252616_136309___Sbg5
CCATCCGACGGACGGATCACTATCAACA
>SRR4252611_2864364___Sbg5
TTAGTGATCAGAAGTTGTACACCACCACTC

>SRR4252623_14177048___Sbg5
AGAAGGTGAAACCTGGTCCCGGCATGTAG
>SRR4252606_5898551___Sbg5
GAAACCTGGTCCCGGCATGTAGCCTAC
>SRR4252610_8775991___Sbg5
ACAATTTAGTGATCAGAAGTTGTACA
>SRR4252608_10905988___Sbg5
ATGTGAGTGACGATTATGGATATGAAAGA
>SRR4252613_448419___Sbg5
CGGCTTAACGTCCCCATCCGACGGACG
>SRR4252611_13294192___Sbg5
CCATCCGACGGACGGATCACTATCAA
>SRR4252621_4162530___Sbg5
TTAGTGATCAGAAGTTGTACACCACCACTC
>SRR4252611_6084607___Sbg5
GTGCACAATTTAGTGATCAGAAGTTGTA
>SRR4252623_14766162___Sbg5
ATAGTTCAACAGCCCTATGTGGGCCAAGG
>SRR4252610_6295191___Sbg5
TTAGTGATCAGAAGTTGTACACCACCACTC
>SRR4252612_1204695___Sbg5
TAGTGATCAGAAGTTGTACACCACCACTC
>SRR4252611_3222740___Sbg5
TTAGTGATCAGAAGTTGTACACCACCACTC
>SRR4252610_7660563___Sbg5
AAGTTGTACACCACCACTCCTCCTAG
>SRR4252611_12842635___Sbg5
ATTTAGTGATCAGAAGTTGTACACCAC
>SRR4252614_3422108___Sbg5
ACCAGAAGTTGTACACCACCACTCCTCCTAG
>SRR4252606_129084___Sbg5
TTAGTGATCAGAAGTTGTACACCACCACTC
>SRR4252610_12176474___Sbg5
TTAGTGATCAGAAGTTGTACACCACCACTC
>SRR4252610_1334517___Sbg5
ACAGACGATAACCCCGGTTTTCTAGACCA
>SRR4252606_5748980___Sbg5
GCTTAATGTCCCCATCCGACGGACGGA
>SRR4252606_7400218___Sbg5
AGTGATCAGAAGTTGTACACCACCACTCC
>SRR4252610_7310356___Sbg5
ACAATTTAGTGATCAGAAGTTGTACACCA
>SRR4252606_6674167___Sbg5
GACGGACGGATCACTATCAACAATGACG
>SRR4252612_7348000___Sbg5
TGTAAGTGTGTGGATAGGAGGATGAGT
>SRR4252611_691323___Sbg5
TCCTTTCGAAAAGCACCAAGGGGACCGGC
>SRR4252624_6539228___Sbg5
TCCTTTCGAAAAGCACCAAGGGGACCGG
>SRR4252605_13020705___Sbg5
AACGTCCCCATCCGACGGACGGATCA
>SRR4252619_3188686___Sbg5
GCACAATTTAGTGATCAGAAGTTGTA
>SRR4252611_784003___Sbg5
TTAGTGATCAGAAGTTGTACACCACCACTC
>SRR4252612_8200716___Sbg5
TGGATATGAAAGAGGGAGAAGGTGAAACC
>SRR4252615_1890562___Sbg5
TATAGCTCAACAGCCCTATGTGGGCCA
>SRR4252608_2334865___Sbg5
ACAATTTAGTGATCAGAAGTTGTACA
>SRR4252611_8777904___Sbg5
CCCCACAGCCGATAACCCCGGTTTTCTA
>SRR4252616_6819164___Sbg5
GTGTAAGTGTGTGGATAGGGGGATGAGT
>SRR4252621_2739337___Sbg5
TGATGTGAGTGACGATTATGGATATGAA
>SRR4252610_897380___Sbg5
GGTACTACGCTGGTCCCCACAGCCGATA

>SRR4252608_3334590___Sbg5
GAATTTGGTGTACAATTTAGTGATCA
>SRR4252609_7778597___Sbg5
TTAGTGATCAGAAGTTGTACACCACAACTCC
>SRR4252614_6827530___Sbg5
TGTGTGTGTGGTGTGGTATGATGTGAGT
>SRR4252609_16261704___Sbg5
CATGTAGCCTACTCCTTTCGAAAAACA
>SRR4252621_4042707___Sbg5
GTGCACAATTTAGTGATCAGAAGTTGTA
>SRR4252609_16451119___Sbg5
TTAGTGATCAGAAGTTGTACACCACCACTC
>SRR4252618_8515842___Sbg5
AGTGATCAGAAGTTGTACACCACCACT
>SRR4252606_4494229___Sbg5
TAGGACGGAATTAACGGGGGGTAGTAGGT
>SRR4252606_7689110___Sbg5
TAGGACGGAATTAACGGGGGGTAGTAGGT
>SRR4252623_14783678___Sbg5
TAGGACGGAATTAACGGGGGGTAGTAGGT
>SRR4252610_6884433___Sbg5
TGAGTGTGTGTATAGTGTTGTATTGTGT
>SRR4252614_3618968___Sbg5
TGATGTGAGTGACGATTATGGATATG
>SRR4252611_3657736___Sbg5
TTAGTGATCAGAAGTTGTACACCACCACT
>SRR4252608_12014672___Sbg5
CCCCATCCGACGGACGGATCACTATCAA
>SRR4252607_1848872___Sbg5
ACAATGACATATGCCTTCTCTACATA
>SRR4252617_1358804___Sbg5
TAACGTCCCCATCCGACGGACGGATCACT
>SRR4252611_12131917___Sbg5
CGGTTTTCTAGACCAGCTAATGGATTGA
>SRR4252607_7956478___Sbg5
TGTGTGTATAGTGTTGTATTGAGTGTGT
>SRR4252613_2319232___Sbg5
TCTACATAGTCATTGCGGAGGAGTTCG
>SRR4252610_5004786___Sbg5
TAGTGATCAGAAGTTGTACACCACCACTC
>SRR4252611_12668579___Sbg5
CATGTAGCCTACTCCTTTCGAAAAGC
>SRR4252621_12360826___Sbg5
TTAGTGATCAGAAGTTGTACACCACCACTC
>SRR4252610_11932696___Sbg5
TTAGTGATCAGAAGTTGTACACCACCACTC
>SRR4252608_11563626___Sbg5
ACAGTACTACGCTGGTCCCCACAGCCGA
>SRR4252607_10053068___Sbg5
TCCCCATCCGACGGACGGATCACTATCAA
>SRR4252612_4025376___Sbg5
TCACTATCAACAATGACATATGCCTTCT
>SRR4252611_11559270___Sbg5
TAGGACGGAATTAACGGGGGGTAGTA
>SRR4252611_10952765___Sbg5
ACATGTAGCCTACTCCTTTCGAAAAGCA
>SRR4252623_2227990___Sbg5
TCCTTTCGAAAAGCACCAAGGGGACT
>SRR4252610_12011912___Sbg5
TTAGTGATCAGAAGTTGTACACCACCACTC
>SRR4252611_9278008___Sbg5
TTAGTGATCAGAAGTTGTACACCACCACTC
>SRR4252614_8311066___Sbg5
CAACAATGACATATGCCTTCTCTACATA
>SRR4252611_1201872___Sbg5
TGTGTGTATAGTGTTGTACTGTGTGTGT
>SRR4252610_8569137___Sbg5
CAGTACTACGCTGGTCCCCACAGCCGA
>SRR4252605_2904078___Sbg5
TTAGTGATCAGAAGTTGTACACCACCACTC

>SRR4252621_8720954___Sbg5
TTAGTGATCAGAAGTTGTACACCACCACTC
>SRR4252610_7840841___Sbg5
TTATGGATATGAAAGAGGGAGAAGGTGA
>SRR4252609_14643077___Sbg5
TAGGACGGAATTAACGGGGGGTAGTATGG
>SRR4252612_8664228___Sbg5
GCAATTTAGTGATCAGAAGTTGTACACCA
>SRR4252622_10986797___Sbg5
TTAGTGATCAGAAGTTGTAAACCACCACTC
>SRR4252608_6954744___Sbg5
CATGTAGCCTACTCCTTTCGAAAAGCA
>SRR4252608_8361538___Sbg5
AATGATGTGAGTGACGATTATGGATATGA
>SRR4252621_12168108___Sbg5
TTTAGTGATCAGAAGTTGTACACCACCA
>SRR4252605_8630376___Sbg5
TTAGTGATCAGAAGTTGTACACCACCACTC
>SRR4252605_7880769___Sbg5
TTAGTGATCAGAAGTTGTACACCACCACTC
>SRR4252610_12341760___Sbg5
TTAGTGATCAGAAGTTGTACACCACCACTC
>SRR4252605_3777609___Sbg5
TAGTGATCAGAAGTTGTACACCACCACTCC
>SRR4252612_7553277___Sbg5
TATGATGTGAGTGACGATTATGGATATG
>SRR4252617_4984083___Sbg5
TAGTGATCAGAAGTTGTACACCACCACTCC
>SRR4252610_7028007___Sbg5
TGTGGTATGATGTGAGTGACGATTAT
>SRR4252605_5356366___Sbg5
TTAATGATCAGAAGTTGTACACCACCACTC
>SRR4252610_10935768___Sbg5
CCACCACTCCTCCTAGTCTCAGGATTCGA
>SRR4252605_4643600___Sbg5
GCGGCTGCCGCCATAGGACGGAATTAACGG
>SRR4252623_1807606___Sbg5
TAGGACGGAATTAACGGGGGGTAGTATGGT
>SRR4252608_6310053___Sbg5
ACAATTTAGTGATCAGAAGTTGTACACCA
>SRR4252612_7823047___Sbg5
TGAGTGACGATTATGGATATGAAAGAGGG
>SRR4252612_5903659___Sbg5
TCACTATCAACAATGACGTATGCCTTCT
>SRR4252615_456971___Sbg5
CCCCACAGCCGATAACCCCGGTTTTCTA
>SRR4252620_8107086___Sbg5
ATGATGTGAGTGACGATTATGGATATGA
>SRR4252619_4426261___Sbg5
TGAATTTGGTGTACAATTTAGTGATCA
>SRR4252612_6592217___Sbg5
TTAGTGATCAGAAGTTGTACACCACCACTC
>SRR4252619_7334850___Sbg5
TACCGAGCGGCTGCCGCCATAGGACGG
>SRR4252619_2163184___Sbg5
TTAGTGATCAGAAGTTGTACACCACCACC
>SRR4252626_8424363___Sbg5
TATGATGTGAGTGACGATTATGGATATG
>SRR4252605_9921496___Sbg5
GATGTGAGTGACGATTATGGATATGAA
>SRR4252608_10304213___Sbg5
TAGTGATCAGAAGTTGTACACCACCACTC
>SRR4252605_1925722___Sbg5
TAGGACGGAATTAACGGGGGGTAGTATT
>SRR4252612_7633142___Sbg5
TAGGACGGAATTAACGGGGGGTAGTATTC
>SRR4252608_11555053___Sbg5
TCCTTTCGAAAAGCACCAAGGGGTCCGT
>SRR4252619_5355240___Sbg5
GTGCACAATTTAGTGATCAGAAGTTGTA

```
>SRR4252606_10237168___Sbg5
TTAGTGATCAGAAGTTGTACACCACCACTC
>SRR4252625_14660003___Sbg5
TTAGTGATCAGAAGTTGTACACCACCACTC
>SRR4252605_5655651___Sbg5
TAGGACGGAATTAACGGGGGGTAGTATGA
>SRR4252613_7276260___Sbg5
CAGTACTACGCTGGTCCCCACAGCCGA
>SRR4252611_10698514___Sbg5
TAATGGATTGGTGTGTAAGTGTGTGGAT
>SRR4252619_1309014___Sbg5
GGGTCCGACGGCTTAACGTCCCCATCCGA
>SRR4252606_9572469___Sbg5
CCGGTTTTCTAGACCAGTTAATGGAT
>SRR4252605_7162299___Sbg5
CAGTTAATGGATCGGTGTGTAAGTGTGTGA
>SRR4252606_863805___Sbg5
TGTAAGTGTGTGAATAGGGGGATGAGTGT
>SRR4252612_32204___Sbg5
TATGATGTGAGTGACGATTATGGATATGA
>SRR4252605_4043135___Sbg5
CCCCATCCGACGGACGGATCACTATCAA
>SRR4252607_2524008___Sbg5
GTCCCCATCCGACGGACGGATCACTATCA
>SRR4252605_4845928___Sbg5
ACATAGTCATTGCGGAGGAGTTCGGGA
>SRR4252605_10891976___Sbg5
GGTCCCCACAGCCGATAGCCCCGGTTTT
>SRR4252612_7185906___Sbg5
TCACTATCAACAATGACATATGCCTTCTC
>SRR4252608_11301024___Sbg5
AGTGATCAGAAGTTGTACACCACCACTCCT
>SRR4252619_9434780___Sbg5
TATGATGTGAGTGACGATTATGGATATG
>SRR4252610_9261532___Sbg5
TCCTTTCGAAAAGCACCAAGGGGACCGGC
>SRR4252619_14424566___Sbg5
CCCCATCCGACGGACGGATCACTATCAAC
>SRR4252606_4632561___Sbg5
TTAGTGATCAGAAGTTGTACACCACCACTC
>SRR4252605_3118834___Sbg5
TAGGACGGAATTAACGGGGGGTAGTAT
>SRR4252612_1329793___Sbg5
ACGGACGGATCACTATCAACAATGACA
>SRR4252606_6579404___Sbg5
TAATGGATTGGTGTGTAAGTGTGTGG
>SRR4252605_13858042___Sbg5
TCAGTACTACGCTGGTCCCCACAGCCGA
>SRR4252605_5732301___Sbg5
TGTAAGTGTGTGAATAGGGGGATGAGTGC
>SRR4252611_9724872___Sbg5
TCAGTACTACGCTGGTCCCCACAGCCGA
>SRR4252605_11114350___Sbg5
CATGTAGCCTACTCCTTTCGAAAAGCA
>SRR4252608_10300587___Sbg5
CAATTTAGCGATCAGAAGTTGTACACCA
>SRR4252614_9533734___Sbg5
GAGCGGCTGCCGCCATAGGGCGGAATTAACG
>SRR4252612_3737444___Sbg5
ATGGATATGAAAGAGGGAGAAGGTGAA
>SRR4252611_5205743___Sbg5
TATGAAAGAGGGAGAAGGTGAAACCCGGTG
>SRR4252624_6281498___Sbg5
TTAGTGATCAGAAGTTGTACACCACCACTC
>SRR4252608_7353892___Sbg5
AACGTCCCCATCCGACGGACGGATCA
>SRR4252610_5347966___Sbg5
ATGATGTGAGTGACGATTATGGATATGAA
>SRR4252610_2831156___Sbg5
TTAGTGATCAGAAGTTGTACACCACCACTC
```

>SRR4252605_5602272___Sbg5
TGTAAGTGTGTGAATAGGGAGATGAGTGT
>SRR4252611_15417232___Sbg5
TGTAAGTGTGTGAATAGGGAGATGAGT
>SRR4252611_1500517___Sbg5
TTAGTGATCAGAAGTTGTACACCACCACTC
>SRR4252609_6039813___Sbg5
GCTTAACGTCCCCATCCGACGGACGGA
>SRR4252619_12604654___Sbg5
GCATGTAGCCTACTCCTTTCGAAAAGCA
>SRR4252609_13002335___Sbg5
TCAGTACTACGCTGGTCCCCACAGCCGA
>SRR4252614_7380926___Sbg5
TAGTGATCAGAAGTTGTACACCACCACT
>SRR4252621_5980794___Sbg5
CGGATCACTATCAACAATGACATATG
>SRR4252610_5834678___Sbg5
TTAGTGATCAGAAGTTGTACACCACCACT
>SRR4252609_5904025___Sbg5
CAGTACTACGCTGGTCCCCACAGCCGA
>SRR4252614_7372267___Sbg5
TCAGTACTACGCTGGTCCCCACAGCCGA
>SRR4252618_9105252___Sbg5
TGTGGTATGATGTGAGTGACGATTATGG
>SRR4252619_1633699___Sbg5
TCCTTTCGAAAAGCACCAAGGGGACCGGC
>SRR4252610_4713732___Sbg5
TTTAGTGATCAGAAGTTGTACACCACCAC
>SRR4252605_2374603___Sbg5
GAAGGTGAAACCCGGTGCCGGCATGTAGC
>SRR4252607_871794___Sbg5
TAGGACGGAATTAACGGGGGGTAGTG
>SRR4252613_5055485___Sbg5
ATGAGTGTGTGTATAGTGTTGTATTG
>SRR4252610_6066163___Sbg5
TACAATTTAGTGATCAGAAGTTGTACA
>SRR4252611_7329599___Sbg5
TAGGACGGAATTAACGGGGGGTAGTAT
>SRR4252612_1192241___Sbg5
GTACCACGCTGGTCCCCACAGCCGATA
>SRR4252605_11195838___Sbg5
TGTAAGTGTGTGAATAGGAGGATGAGTGT
>SRR4252623_7886026___Sbg5
TATGAAAGAGGGAGAAGGTGAAACCCGG
>SRR4252620_9810949___Sbg5
ACAGCCCTATGTGGGCCAAGGCTTACCGA
>SRR4252615_2819955___Sbg5
GTCCCCATCCGACGGACGGATCACTATCAA
>SRR4252605_5890054___Sbg5
ACAATTTAGTGATCAGAAGTTGTACA
>SRR4252611_9425329___Sbg5
TTAGTGATCAGAAGTTGTACACCACCACTC
>SRR4252612_8262820___Sbg5
TTAGTGATCAGAAGTTGTACACCACCACTC
>SRR4252605_14458368___Sbg5
AAGAGGGAGAAGGTGAAACCCGGTGCCGGCA
>SRR4252610_10933207___Sbg5
TCACTATCAACAATGACGTATGCCTTCTC
>SRR4252621_8358372___Sbg5
TAATGGATTGGTGTGTAAGTGTGTGGA
>SRR4252607_11999772___Sbg5
ACGTGAGTGACGATTATGGATATGAA
>SRR4252612_7349841___Sbg5
TCCCCATCCGACGGACGGATCACTATC
>SRR4252610_5275732___Sbg5
TTAGTGATCAGAAGTTGTACACCACCACTC
>SRR4252611_9767325___Sbg5
TTAGTGATCAGAAGTTGTACACCACCACTC
>SRR4252611_9505088___Sbg5
ATGACGTGAGTGACGATTATGGATATGA

>SRR4252611_9418164___Sbg5
CTATCAACAATGACATATGCCTTCTCTA
>SRR4252619_9767885___Sbg5
TTAGTGATCAGAAGTTGTACACCACCACTC
>SRR4252611_10522433___Sbg5
TAGTATGGTCAGTACTACGCTAGTCCCCT
>SRR4252607_1216694___Sbg5
TCCTTTCGAAAAGCACCAAGGGGACCGG
>SRR4252613_242263___Sbg5
GTGCACAATTTAGTGATCAGAAGTTGTA
>SRR4252610_6710137___Sbg5
TTAGTGATCAGAAGTTGTACACCACCACTC
>SRR4252611_3919269___Sbg5
TTAGTGATCAGAAGTTGTACACCACCACT
>SRR4252623_6234492___Sbg5
TTAGTGATCAGAAGTTGTACACCACCA
>SRR4252609_14540897___Sbg5
TATGATGTGAGTGACGATTATGGATATGA
>SRR4252611_3059501___Sbg5
TAGGACGGAATTAACGGGGGGTAGTATGG
>SRR4252610_5068147___Sbg5
AGTACTACGCTGGTCCCCACAGCCGATAG
>SRR4252606_1210017___Sbg5
TATGATGTGAGTGACGATTATGGATATGAC
>SRR4252608_5187824___Sbg5
TGCATGTAGCCTACTCCTTTCGAAAAGCA
>SRR4252614_6700077___Sbg5
TGCATGTAGCCTACTCCTTTCGAAAA
>SRR4252611_5780319___Sbg5
TTAGTGATCAGAAGTTGTACACCACCACTCC
>SRR4252610_2147227___Sbg5
ACAATTTAGTGATCAGAAGTTGTACAC
>SRR4252612_3566538___Sbg5
TGTACACCACCACTCCTCCTAATCTCAG
>SRR4252610_1272060___Sbg5
GTCCTCATCCGACGGACGGATCACTATCAA
>SRR4252608_9547675___Sbg5
GTGTATAGTGTTGTATTGTGTGTGTA
>SRR4252623_7132992___Sbg5
ATGTAGCCTACTCCTTTCGAAAAGCACC
>SRR4252611_12587585___Sbg5
TTAGTGATCAGAAGTTGTACACCACCACTC
>SRR4252605_2997072___Sbg5
TTAGTGATCAGAAGTTGTACACCAGCACT
>SRR4252606_5172629___Sbg5
TAGTGATCAGAAGTTGTACACCAGCACTCC
>SRR4252621_7243453___Sbg5
TGTGAGTGACGATTATGGATATGAAAG
>SRR4252606_4193809___Sbg5
TTAGTGATCAGAAGTTGTACACCACCACTC
>SRR4252606_7252169___Sbg5
TCTCAACATAGTCATTGCGGAGGAGTTCGGG
>SRR4252610_9826787___Sbg5
TTAGTGATCAGAAGTTGTACACCACCACTC
>SRR4252608_1500229___Sbg5
TATGATGTGAGTGACGATTATGGATATT
>SRR4252619_2966209___Sbg5
CTTAACGTCCCCATCCGACGGACGGATCA
>SRR4252612_4835070___Sbg5
GTCCCCATCCGACGGACGGATCACTATCA
>SRR4252610_9994018___Sbg5
TATGAAAGAGGGAGAAGGTGAAACCCGG
>SRR4252606_255590___Sbg5
TTAGTGATCAGAAGTTGTACACCACCACTT
>SRR4252612_1320478___Sbg5
TAGTGATCAGAAGTTGTACACCACCACTT
>SRR4252612_4396545___Sbg5
AACAGCCCCATGTGGGCCAAGGCTTACCGA
>SRR4252611_13707664___Sbg5
CACAGCCGATAACCCCGGTTTTCTAGA

>SRR4252610_8721796___Sbg5
TTAGTGATCAGAAGTTGTACACCACCACTC
>SRR4252610_12341680___Sbg5
GTAGTATGATGTGAGTGACGATTATGGA
>SRR4252612_4594475___Sbg5
TATGATGTGAGTGACGATTATGGATATG
>SRR4252610_834578___Sbg5
TATGAAAGAGGGAGAAGGTGAAACCCGGTA
>SRR4252625_5028044___Sbg5
TATGAAAGAGGGAGAAGGTGAAACCCGGTA
>SRR4252610_5079555___Sbg5
TTAGTGATCAGAAGTTGTACACCACCACTC
>SRR4252606_10335539___Sbg5
GCTAGTCCCCACAGCCGATAGCCCCGGTTT
>SRR4252607_3787611___Sbg5
CCAGTTAATGGATCGGTGTGTAAGTGTG
>SRR4252610_4135009___Sbg5
TTAGTGATCAGAAGTTGTACACCACCACTT
>SRR4252605_12815347___Sbg5
TAGGACGGAATTAACGGGGGGTAGTATGG
>SRR4252612_1131446___Sbg5
TGTATTGTGTGTGTGGTGTGGTATGATGT
>SRR4252611_12797477___Sbg5
TTAGTGATCAGAAGTTGTACACCACCACTC
>SRR4252611_610927___Sbg5
TAGTGATCAGAAGTTGTACACCACCACTC
>SRR4252606_141911___Sbg5
TATGATGTGAGTGACGATTATGGATATGAC
>SRR4252606_8449195___Sbg5
TATGATGTGAGTGACGATTATGGATATGAC
>SRR4252608_5291389___Sbg5
ACAATTTAGTGATCAGAAGTTGTACA
>SRR4252619_1234284___Sbg5
TACTCCTTTCGAAAAGCACCAAGGGGACTG
>SRR4252606_9727615___Sbg5
TAGGACGGAATTAACGGGGTGTAGTATGG
>SRR4252610_11400725___Sbg5
ACAATTTAGTGATCAGAAGTTGTACACCA
>SRR4252621_9123885___Sbg5
TTACCGAGCGGCTGCCGCCATAGGACGG
>SRR4252611_9530418___Sbg5
TACCGAGCGGCTGCCGCCATAGGACGGAAT
>SRR4252608_4043138___Sbg5
TATGATATGAGTGACGATTATGGATATGA
>SRR4252611_11135978___Sbg5
GCACGTAGCCTACTCCTTTCGAAAAGCA
>SRR4252612_2399156___Sbg5
GCACGTAGCCTACTCCTTTCGAAAAGCA
>SRR4252611_1627378___Sbg5
CACGTAGCCTACTCCTTTCGAAAAGCA
>SRR4252608_2556345___Sbg5
TCCTTTCGAAAAGCACCAAGGGGACCGGT
>SRR4252606_10142906___Sbg5
GTCAGTACCACGCTGGTCCCCACAGCCGA
>SRR4252610_11517826___Sbg5
TTAGTGATCAGAAGTTGTACACCACCACTC
>SRR4252617_10083637___Sbg5
TGTGTATAGTGTTGTATTGTGTGTGTGT
>SRR4252619_14364622___Sbg5
ACAATTTAGTGATCAGAAGTTGTACAC
>SRR4252613_5072295___Sbg5
TCCCCATCCGACGGACGGATCACTATCA
>SRR4252620_7544509___Sbg5
CCATCCGACGGACGGATCACTATCAACAA
>SRR4252612_2533179___Sbg5
GTGGTATGATGTGAGTGACGATTATGGA
>SRR4252606_2226142___Sbg5
TTAGTGATCAGAAGTTGTACACCACCACTT
>SRR4252610_6756415___Sbg5
CTTAACGTCCCCATCCGACGGACGGA

>SRR4252607_10069731___Sbg5
TTAGTGATCAGAAGTTGTACACCACCACTC
>SRR4252611_5728573___Sbg5
TATGATGTGAGTGACGATTATGGATATGA
>SRR4252616_1041052___Sbg5
GTCCCCATCCGACGGACGGATCACTATCAAC
>SRR4252611_1290442___Sbg5
ACAATTTAGTGATCAGAAGTTGTACA
>SRR4252621_9695725___Sbg5
TTAGTGATCAGAAGTTGTACCCCACCACTC
>SRR4252614_1478965___Sbg5
TAACGTCCCCATCCGACGGACGGATCAC
>SRR4252605_7919643___Sbg5
TTAGTGATCAGAAGTTGTACACCACC
>SRR4252606_1577364___Sbg5
TTAGTGATCAGAAGTTGTACACCACCACTC
>SRR4252611_11514713___Sbg5
ACAGGATTCGAAATTTTAATTAATTACTCA
>SRR4252612_6791508___Sbg5
TTAGTGATCAGAAGTTGTACACCACCACTC
>SRR4252605_11953731___Sbg5
GTCCCCATCCGACGGACGGATCACTATCAA
>SRR4252608_1052847___Sbg5
TTAGTGATCAGAAGTTGTACACCACCAT
>SRR4252608_649084___Sbg5
TAGTGATCAGAAGTTGTACACCACCACTC
>SRR4252621_1634663___Sbg5
CCATCCGACGGACGGATCACTATCAACA
>SRR4252611_2443149___Sbg5
TAGGACGGAATTAACGGGGGGTAGTATGG
>SRR4252614_9387652___Sbg5
GGCTTAACGTCCCCATCCGACGGACG
>SRR4252611_14194692___Sbg5
GTCCCCATCCGACGGACGGATCACTATCAA
>SRR4252612_8198529___Sbg5
GTGAGTGACGATTATGGATATGAAAGAG
>SRR4252620_6656680___Sbg5
AGGGGGATGAGTGTGTGTATAGTGTTGTATT
>SRR4252619_6765193___Sbg5
CCCCATCCGACGGACGGATCACTATCAA
>SRR4252611_9241187___Sbg5
ACAATTTAGTGATCAGAAGTTGTACA
>SRR4252607_6735243___Sbg5
CCCCATCCGACGGACGGATCACTATCAA
>SRR4252608_2982956___Sbg5
ACAATTTAGTGATCAGAAGTTGTACACCA
>SRR4252622_7864801___Sbg5
ATGTGAGTGACGATTATGGATATGAAAG
>SRR4252625_10146077___Sbg5
AGTGATCAGAAGTTGTACACCACCAC
>SRR4252621_13237181___Sbg5
TGAGTGACGATTATGGATATGAAAGAGGGA
>SRR4252605_6686931___Sbg5
TATGAAAGAGGGAGAAGGTGAAACCCGGTG
>SRR4252612_2725350___Sbg5
TTAGTGATCAGAAGTTGTACACCACCACTC
>SRR4252612_5320357___Sbg5
TTAGTGATCAGAAGTTGTACACCACCACTC
>SRR4252610_13124911___Sbg5
TATGATGTGAGTGACGATTATGGATA
>SRR4252606_7055539___Sbg5
AACGTCCCCATCCGACGGACGGATCACTAT
>SRR4252607_5545492___Sbg5
TCAACAATGGCATATGCCTTCTCAACATA
>SRR4252605_15049933___Sbg5
TATGATGTGAGTGACGATTATGGATATGA
>SRR4252609_87089___Sbg5
GAGTGTGTGTATAGTGTTGTATTGTGTG
>SRR4252625_3767922___Sbg5
TGGATATGAAAGAGGGAGAAGGTGAAACC

>SRR4252621_11670052___Sbg5
CGGCCGGATCACTATCAACAATGACA
>SRR4252611_845338___Sbg5
TCAGTACTACGCTGGTCCCCACAGCCGA
>SRR4252610_8914007___Sbg5
TTAGTGATCAGAAGTTGTACACCACCACTC
>SRR4252606_2590717___Sbg5
ACGCTGGTCCCCACAGCCGATAGCCC
>SRR4252605_9991739___Sbg5
AGTGTGTGTATAGTGTTGTATTGTGTGT
>SRR4252606_4910993___Sbg5
GTCCCCATCCGACGGACGGATCACTATCAA
>SRR4252611_6620251___Sbg5
TGTGTAAGTGTGTGAATAGGGAGATGAGT
>SRR4252610_2563396___Sbg5
TCACTATCAACAATGACGTATGCCTTCTC
>SRR4252610_6948083___Sbg5
AGTGATCAGAAGTTGTACACCACCACTCC
>SRR4252613_1684616___Sbg5
TAGGACGGAATTAACGGGGGGTAGTATGG
>SRR4252606_5453529___Sbg5
TTAGTGATCAGAAGTTGTACACCACTACT
>SRR4252609_15283296___Sbg5
TAAGTGTGTGGATAGGGGGATGAGTGTG
>SRR4252613_8766175___Sbg5
TATGAAAGAGGGAGAAGGTGAAACCTGG
>SRR4252610_4415268___Sbg5
TAATGGATCGGTGTGTAAGTGTGTGAAT
>SRR4252605_2190973___Sbg5
GGTATGATGTGAGTGACGATTATGGATAT
>SRR4252614_6932103___Sbg5
CACAATTTAGTGATCAGAAGTTGTACA
>SRR4252606_10156180___Sbg5
TAGGACGGAATTAACGGGGGGTAGTATG
>SRR4252616_8886755___Sbg5
CAGTACTATGCTGGTCCCCACAGCCGA
>SRR4252605_4489108___Sbg5
ATTTAGTGATCAGAAGTTGTACACCACCA
>SRR4252606_5278570___Sbg5
GATCAGAAGTTGTACACCACCACTCC
>SRR4252615_4211979___Sbg5
TAGCTCAACAGCCCAATGTGGGCCAAGG
>SRR4252622_8067470___Sbg5
TTAGTGATCAGAAGTTGTACACCACCACA
>SRR4252606_3552602___Sbg5
GATGTGAGTGACGATTATGGATATGAAA
>SRR4252606_9885557___Sbg5
ATGAAAGAGGGAGAAGGTGAAACCTGGTC
>SRR4252617_13477519___Sbg5
GTGGTATGACGTGAGTGACGATTATGGA
>SRR4252609_10863412___Sbg5
TTAGTGATCAGAAGTTGTACACCACCA
>SRR4252611_2294092___Sbg5
TTAGTGATCAGAAGTTGTACACCACCACTC
>SRR4252609_11139463___Sbg5
CCAGCTAATGGATTGGTGTGTAAGTGTG
>SRR4252608_2285426___Sbg5
TGTAAGTGTGTGGATAGGGTGATGAGT
>SRR4252606_3560044___Sbg5
TTAGTGATCAGAAGTTGTACACCACCACTC
>SRR4252620_8214610___Sbg5
AGGTGAAATTGGTGTACAATTTAGTGATC
>SRR4252608_7232721___Sbg5
TGGATATGAAAGAGGGAGAAGGTGAAACC
>SRR4252606_124770___Sbg5
TCTCTACATAGTCATTGCGGAGGAGTTCGG
>SRR4252621_13705140___Sbg5
TTAGTGATCAGAAGTTGTACACAACCACTC
>SRR4252625_1739181___Sbg5
CAGTACTACGCTGGTCCCCACAGCCGA

>SRR4252617_5304580___Sbg5
CCATCCGACGGACGGATCACTATCAACAA
>SRR4252605_10633628___Sbg5
GCAATTTAGTGATCAGAAGTTGTACACCA
>SRR4252610_3085516___Sbg5
GCAATTTAGTGATCAGAAGTTGTACACCA
>SRR4252610_5633342___Sbg5
GCAATTTAGTGATCAGAAGTTGTACACCA
>SRR4252610_6809835___Sbg5
GCAATTTAGTGATCAGAAGTTGTACACCA
>SRR4252606_9109918___Sbg5
TTAGTGATCAGAAGTTGTACACCACCACTC
>SRR4252614_1063974___Sbg5
AACGTCCCCATCCGACGGACGGATCACTA
>SRR4252605_4181829___Sbg5
TACCGAGCGGCTGCCGCCATAGGGCGGAA
>SRR4252624_3745557___Sbg5
ACGGACGGATCACTATCAACAATGACA
>SRR4252605_10214202___Sbg5
CCGACGGCTTAACGTCCCCATCCGACGGACG
>SRR4252606_3050630___Sbg5
TGTAAGTGTGTGAATAGGGAGATGAGTGT
>SRR4252611_8869131___Sbg5
TGTAAGTGTGTGAATAGGGAGATGAGTG
>SRR4252608_11287901___Sbg5
TAAGTGTGTGAATAGGGGGATGAGTGTGT
>SRR4252611_4777209___Sbg5
TCCTTTCGAAAAGCACCAAGGGGACTG
>SRR4252612_1243289___Sbg5
TAGTGATCAGAAGTTGTACACCACCACTC
>SRR4252607_5844597___Sbg5
TATAGCTCAACAGCCCGATGTGGGCCA
>SRR4252621_11642760___Sbg5
TACCGAGCGGCTGCCGCCATAGGGCGGAA
>SRR4252613_10497814___Sbg5
TGACGTGAGTGACGATTATGGATATGAA
>SRR4252624_6418417___Sbg5
CCGACGGACGGATCACTATCAACAATG
>SRR4252610_5301976___Sbg5
ACAATTTAGTGATCAGAAGTTGTACACCA
>SRR4252610_10670442___Sbg5
TTAGTGATCAGAAGTTGTACACCACCAC
>SRR4252611_5030043___Sbg5
TTAGTGATCAGAAGTTGTACACCACCTC
>SRR4252621_7861032___Sbg5
TTAGTGATCAGAAGTTGTACACCACC
>SRR4252624_4274887___Sbg5
TCAATTTAGTGATCAGAAGTTGTACA
>SRR4252605_3698032___Sbg5
AAGTGACGATTATGGATATGAAAGAG
>SRR4252605_2255656___Sbg5
TTAGTGATCAGAAGTTGTACACCACCACTC
>SRR4252608_10670041___Sbg5
TATGATGTGAGTGACGATTATGGATA
>SRR4252613_1681478___Sbg5
TTAGTGATCAGAAGTTGTACACCACCACTC
>SRR4252619_1050485___Sbg5
GTATGATATGAGTGACGATTATGGATATGAA
>SRR4252616_32074___Sbg5
GGTGATCAGAAGTTGTACACCACCACT
>SRR4252623_13109207___Sbg5
CCCCATCCGACGGACGGATCACTATCAA
>SRR4252606_2406009___Sbg5
TTAGTGATCAGAAGTTGTACACCACCACTC
>SRR4252610_4552041___Sbg5
CGTGAGTGACGATTATGGATATGAAAG
>SRR4252622_7461497___Sbg5
TCACTATCAACAATGACATATGCCTTCT
>SRR4252613_4637737___Sbg5
AATCCGACGGACGGATCACTATCAACAA

>SRR4252620_413502___Sbg5
TTACCGAGCGGCTGCCGCCATAGGACGG
>SRR4252611_5386919___Sbg5
TAGGACGGAATTAACGGGGGGTAGTATG
>SRR4252611_5286674___Sbg5
TGTAAGTGTGTGAATAGGGAGATGAGTG
>SRR4252608_3999317___Sbg5
AACGTCCCCATCCGACGGACGGATCACTA
>SRR4252608_5149831___Sbg5
TAATGGATCGGTGTGTAAGTGTGTGAAT
>SRR4252619_6280645___Sbg5
TTAGTGATCAGAAGTTGTACACCACCGCTC
>SRR4252623_13159781___Sbg5
TTAGTGATCAGAAGTTGTACACCACCGC
>SRR4252606_2927751___Sbg5
TAGTGATCAGAAGTTGTACACCACCGCTC
>SRR4252621_6181177___Sbg5
TGTGGTATGACGTGAGTGACGATTATGGA
>SRR4252613_6373742___Sbg5
TATGACGTGAGTGACGATTATGGATATG
>SRR4252611_6565255___Sbg5
TCACTATCAACAATGACATATGCCTTCTCA
>SRR4252611_9490405___Sbg5
ATGACGTGAGTGACGATTATGGATATGAA
>SRR4252606_8658361___Sbg5
AACGTCCCCATCCGACGGACGGATCACTATC
>SRR4252609_1242232___Sbg5
GTGAGTGACGATTATGGATATGAAAG
>SRR4252611_6191772___Sbg5
GTCAGTACTATGCTGGTCCCCACAGCCGA
>SRR4252615_12809708___Sbg5
CAGTACTATGCTGGTCCCCACAGCCGA
>SRR4252610_9054411___Sbg5
TGTAAGTGTGTGAATAGGGAGATGAGTGT
>SRR4252610_10522485___Sbg5
TAGGACGGAATTAACGGGGGATAGTATGG
>SRR4252619_596036___Sbg5
AGTACGCTGGTCCCCACAGCCGATAA
>SRR4252605_10958617___Sbg5
AGCTAATGGATTGGTGTGTAAGTGTGTG
>SRR4252611_13081731___Sbg5
GTACTACGCTGGTCCCCACAGCCGATAA
>SRR4252605_10915359___Sbg5
ATTTAATGATCAGAAGTTGTACACCA
>SRR4252614_5317703___Sbg5
CCCCACAGCCGATAACCCCGGTTTTCTA
>SRR4252611_4999___Sbg5
CCATCCGACGGACGGATCACTATCAACA
>SRR4252612_3900973___Sbg5
GGAGTGACGATTATGGATATGAAAGAG
>SRR4252613_4653380___Sbg5
GGAGTGACGATTATGGATATGAAAGAG
>SRR4252611_13296412___Sbg5
TATGAAAGAGGGAGAAGGTGAAACCTGT
>SRR4252611_4002309___Sbg5
TATGAAAGAGGGAGAAGGTGAAACCTGTGC
>SRR4252615_10308404___Sbg5
ACAATTTAGTGATCAGAAGTTGTACACCA
>SRR4252610_7434828___Sbg5
TCGGTGTGTAAGTGTGTGAATAGGAGGA
>SRR4252620_8114887___Sbg5
CTTACCGAGCGGCTGCCGCCATAGGGCGGA
>SRR4252608_10766027___Sbg5
TTAGTGATCAGAAGTTGTACACCACCACC
>SRR4252608_11211500___Sbg5
TTAGTGATCAGAAGTTGTACACCACCACCA
>SRR4252623_7419843___Sbg5
GAGCGGTTGCCGCCATAGGACGGAATT
>SRR4252623_11365913___Sbg5
ATGGATATGAAAGAGGGAGAAGGTGA

>SRR4252619_15416960___Sbg5
TACCGAGCGGCTGCCGCCATAGGACGGAA
>SRR4252605_10304251___Sbg5
GCGGCTGCCGCCATAGGACGGAATTAACGG
>SRR4252606_10247513___Sbg5
CCCCACAGCCGATAACCCCGGTTTTCTA
>SRR4252619_1713327___Sbg5
GAGAAGGTGAAACCCGGTGCCGGCATGTA
>SRR4252608_1348620___Sbg5
CACGTAGCCTACTCCTTTCGAAAAGCA
>SRR4252611_8306517___Sbg5
ACGTAGCCTACTCCTTTCGAAAAGCA
>SRR4252621_2586560___Sbg5
TCCTTTCGAAAAGCACCAAGGGAACCGG
>SRR4252605_10032224___Sbg5
TTAGTGATCAGAAGTTGTACACCACCTCTC
>SRR4252608_11761721___Sbg5
TGATCATTGTGTATGTGTGCGGATAGGAG
>SRR4252622_7853250___Sbg5
TTAGTGATCAGAAGTTGTACACCACCAC
>SRR4252619_15103397___Sbg5
TAGTGATCAGAAGTTGTACACCACCACTC
>SRR4252611_13471210___Sbg5
TGACGTGAGTGACGATTATGGATATGAA
>SRR4252610_11969484___Sbg5
TCACAGCCGATAGCCCCGGTTTTCTA
>SRR4252622_10046290___Sbg5
TTAGTGATCAGAAGTTGTACACCACCAC
>SRR4252611_4525421___Sbg5
TATGAAAGAGGGAGAAGGTGAAACCCGGC
>SRR4252612_5485920___Sbg5
TCACTATCAACAATGACGTATGCCTTCT
>SRR4252606_2354350___Sbg5
TTAGTGATCAGAAGTTGTACACCACCACTC
>SRR4252611_3863667___Sbg5
TTAGTGATCAGAAGTTGTACACCACCACT
>SRR4252612_6043710___Sbg5
TTAGTGATCAGAAGTTGTACACCACCACTC
>SRR4252610_2768462___Sbg5
TTAGTGATCAGAAGTTGTACACCACCACTC
>SRR4252611_11791293___Sbg5
ACAGCCCCATGTGGGCCAAGGCTTACCGA
>SRR4252606_7385666___Sbg5
TAGGACGGAATTAACGGGGGGTAGTATGG
>SRR4252613_7933695___Sbg5
TGCACAATTTAGTGATCAGAAGTTGTACAC
>SRR4252613_2067680___Sbg5
TAGGACGGAATTAACGGGGGGTAGTATG
>SRR4252619_784285___Sbg5
TGATGTGAGTGACGATTATGGATATGA
>SRR4252612_6764231___Sbg5
GACGGACGGTTCACTATCAACAATGACAT
>SRR4252611_6445228___Sbg5
TCTATAGCTCAACAGCCCGATGTGGG
>SRR4252608_10823906___Sbg5
GCCCCATCCGACGGACGGATCACTATCAA
>SRR4252625_9216955___Sbg5
GTGCACAATTTAGTGATCAGAAGTTGTA
>SRR4252619_14743435___Sbg5
CCCCACAGCCGATAACCCCGGTTTTCTA
>SRR4252609_14943432___Sbg5
TAACGTCCCCATCCGACGGACGGATC
>SRR4252606_7917811___Sbg5
TTAGTGATCAGAAGTTGTACACCACCACTC
>SRR4252605_116955___Sbg5
GAACGGTGTGTAAGTGTGTGAATAGG
>SRR4252605_6904176___Sbg5
TCAACAATGACATATGCCTTCTCTACATA
>SRR4252616_7993312___Sbg5
AGTACTATGCTGGTCCCCACAGCCGATA

```
>SRR4252609_13425946___Sbg5
TAGGACGGAATTAACGGGGGGTAGTATGG
>SRR4252610_5332458___Sbg5
TATGAAAGAGGGAGAAGGTGGAACCCGGT
>SRR4252607_10443990___Sbg5
CCATCCGACGGACGGATCACTATCAA
>SRR4252612_1461988___Sbg5
GAGCGGCTGCCGCCATAGGACGGAAT
>SRR4252619_3892769___Sbg5
TATGGTCAGTACTACGCTGGTCCCCACAGC
>SRR4252610_11162900___Sbg5
TAGTGATCAGAAGTTGTACACCACCACTC
>SRR4252612_4127809___Sbg5
TCATTGTGTATGTGTGCGGATAGGAGGA
>SRR4252619_5327929___Sbg5
ACAATTTAGTGATCAGAAGTTGTACA
>SRR4252625_9733499___Sbg5
ACAATTTAGTGATCAGAAGTTGTACACCA
>SRR4252610_10135168___Sbg5
TTAGTGATCAGAAGTTGTACACCACCACTC
>SRR4252611_11035886___Sbg5
TCACTATCAACAATGACGTATGCCTTCTC
>SRR4252625_7012354___Sbg5
TTAGTGATCAGAAGTTGTACAACACCACTC
>SRR4252613_3263576___Sbg5
TAGTGTTGTATTGTGTGTGGTGTG
>SRR4252607_7925177___Sbg5
GTGAGTGACGATTATGGATATGAAAGAG
>SRR4252611_13931494___Sbg5
TTAGTGATCAGAAGTTGTACACCACCACTC
>SRR4252605_3877405___Sbg5
GAACGGTGTGTAAGTGTGTGAATAGG
>SRR4252605_11847450___Sbg5
TAGGACGGAATTAACGGGAGGTAGTATGG
>SRR4252621_8571232___Sbg5
TAGGACGGAATTAACGGGAGGTAGTATGG
>SRR4252610_9202685___Sbg5
TATTTGATCATTGTGTATGTGTGCGGAT
>SRR4252605_9891611___Sbg5
TGAATTTGGTGTACAATTTAGTGATC
>SRR4252613_8808684___Sbg5
TATGAAAGAGGGAGAAGGTGAAACCTGG
>SRR4252610_11955926___Sbg5
TATGATGTGAGTGACGATTATGGATA
>SRR4252609_4942583___Sbg5
TCAGTACTACGCTGGTCCCCACAGCCGA
>SRR4252623_11859864___Sbg5
TATGATGTGAGTGACGATTGTGGATATGA
>SRR4252609_12835629___Sbg5
CCACCGCTCCTCCTAGTCTCAGGATT
>SRR4252611_2472744___Sbg5
TGTGTATAGTGTTGTATTGTGTGTGTG
>SRR4252616_3828562___Sbg5
CAACAATGACATATGCCTTCTCTACATA
>SRR4252622_114016___Sbg5
TGAGTGACGATTATGGATATGAAAGAGA
>SRR4252610_8989058___Sbg5
TCATTGTGTATGTGTGCGGATAGGAGGA
>SRR4252606_3150495___Sbg5
GGTGTGGTATGATGTGAGTGACGATTATG
>SRR4252619_6489428___Sbg5
TATGATGTGAGTGACGATTATGGATAT
>SRR4252619_12534507___Sbg5
GCTTAACGTCCCCATCCGACGGACGGA
>SRR4252626_9234631___Sbg5
TTAGTGATCAGAAGTTGTACACCACCACTC
>SRR4252607_5789707___Sbg5
CCATCCGACGGACGGATCACTATCAA
>SRR4252606_5036963___Sbg5
ATGGTATGATGTGAGTGACGATTATGGA
```

>SRR4252611_11969714___Sbg5
ACAATTTAGTGATCAGAAGTTGTACACCA
>SRR4252605_8062639___Sbg5
TATGATGTGAGTGACGATTATGGATATG
>SRR4252613_7475635___Sbg5
CCCCATCTGACGGACGGATCACTATCAA
>SRR4252610_7840639___Sbg5
TTAATGATCAGAAGTTGTACACCACCACTC
>SRR4252608_4799350___Sbg5
TAGGACGGAATTAACGGGGGGTAGTATGA
>SRR4252611_11761034___Sbg5
CCCCACAGCCGATAACCCCGGTTTTCTA
>SRR4252611_7352953___Sbg5
CCCCACAGCCGATAACCCCGGTTTTCTA
>SRR4252613_8809160___Sbg5
TCCTTTCGAAAAGCACCAAGGGGACCGG
>SRR4252619_5897031___Sbg5
CCCCACAGCCGATAACCCCGGTTTTCTA
>SRR4252610_13109053___Sbg5
TTTAGTGATCAGAAGTTGTACACCACCA
>SRR4252610_5267256___Sbg5
TTAGTGATCAGAAGTTGTACACCACCACTC
>SRR4252620_1311439___Sbg5
TACCGAGCGGCTGCCGCCATAGGGCGGAA
>SRR4252621_4860633___Sbg5
CCCCACAGCCGATAACCCCGGTTTTCTA
>SRR4252613_4438512___Sbg5
ACAATTTAGTGATCAGAAGTTGTACACCA
>SRR4252608_5123323___Sbg5
TTAGTGATCAGAAGTTGTACACCACCACTC
>SRR4252607_12069913___Sbg5
TGTATGTGTGCGGATAGGATGATGAGTGT
>SRR4252611_7871408___Sbg5
AATGTATAGTGTTGTATTGTGTGTGTGGTG
>SRR4252609_10818473___Sbg5
TAGGACGGAATTAACGGGGGATAGTATGG
>SRR4252610_10076548___Sbg5
GCAATTTAGTGATCAGAAGTTGTACAC
>SRR4252616_13040908___Sbg5
AGTGTACAATTTAGTGATCAGAAGTTGTA
>SRR4252611_2065717___Sbg5
GCTATTTGATCATTGTGTATGTGTGCGGA
>SRR4252611_9059149___Sbg5
TAGGACGGAATTAACGGGGGGTAGTAAGGT
>SRR4252613_1738330___Sbg5
TATTGTGTGTGTGGTGTGGTATGATGC
>SRR4252605_8852322___Sbg5
ATTTGATCATTGTGTATGTGTGCGGATAG
>SRR4252621_6698033___Sbg5
TTAGTGATCAGAAGTTGTACACCACCA
>SRR4252624_3684454___Sbg5
CCCCACAGCCGATAACCCCGGTTTTCTA
>SRR4252620_887466___Sbg5
TACTACGCTGGTCCCCACAGCCGATAA
>SRR4252611_14885158___Sbg5
AGCTATTTGATCATTGTGTATGTGTGCG
>SRR4252612_7840448___Sbg5
TATTTGATCATTGTGTATGTGTGCGGATA
>SRR4252605_2569332___Sbg5
TCATTGTGTATGTGTGCGGATAGGAGGA
>SRR4252607_10024993___Sbg5
TGTAAGTGTGTGAATAGGGAGATGAGTGT
>SRR4252607_4577782___Sbg5
TTAGTGATCAGAAGTTGTACACCACCACT
>SRR4252607_2321002___Sbg5
AATGATGTGAGTGACGATTATGGATATGA
>SRR4252611_3228526___Sbg5
TATGAAAGAGGGAGAAGGTGAAACCCGG
>SRR4252609_14900273___Sbg5
GCATGTAGCCTACTCCTTTCGAAAAGCA

>SRR4252605_10319157___Sbg5
CAGCTAATGGATCGGTGTGTAAGTGT
>SRR4252612_7604911___Sbg5
AGTGATCAGAAGTTGTACACCACCACTCC
>SRR4252614_4999397___Sbg5
CCCACAGCCGATAACCCCGGTTTTCTA
>SRR4252621_12089147___Sbg5
TGGATCGGTGTGTAAGTGTGTGAATAGGG
>SRR4252607_6407909___Sbg5
ACAATGACATATGCCTTCTCAACATA
>SRR4252606_9896873___Sbg5
TTAATGATCAGAAGTTGTACACCACCACTC
>SRR4252612_2080889___Sbg5
TACTACGCTGATCCCCACAGCCGATAAC
>SRR4252611_9411475___Sbg5
GGTCCCGGCATGTAGCCTACTCCTTTCGAA
>SRR4252611_6298540___Sbg5
TCCTTTCGAAAAGCACCAAGGGGACCGG
>SRR4252620_2962357___Sbg5
CCCCACAGCCGATAACCCCGGTTTTCTA
>SRR4252624_6637675___Sbg5
TTAGTGATCAGAAGTTGTACACCACCACT
>SRR4252621_5341550___Sbg5
TGTGTGTATAGTGTTGTATTGTGTGTGT
>SRR4252613_9883084___Sbg5
CCCACAGCCGATAACCCCGGTTTTCTA
>SRR4252610_5149168___Sbg5
TTAGTGATCAGAAGTTGTACACCACCACTC
>SRR4252607_11632610___Sbg5
GTCAGTACTACGCTGGTCCCCACAGCCGA
>SRR4252619_10610002___Sbg5
TTCCCCATCCGACGGACGGATCACTATCA
>SRR4252608_3932063___Sbg5
CCATCCGACGGACGGATCACTATCAA
>SRR4252612_6238531___Sbg5
TCACTATCAACAATGACGTATGCCTTC
>SRR4252611_1739397___Sbg5
TTAGTGATCAGAAGTTGTACACCACCACTCC
>SRR4252606_6611574___Sbg5
AACCCGGTGCCGGCATGTAGCCTACG
>SRR4252615_7113194___Sbg5
TTAGCTCAACAGCCCTATGTGGGCCAA
>SRR4252623_4225953___Sbg5
TTAGTGATCAGAAGTTGTACACCACCACTC
>SRR4252609_14589681___Sbg5
CGGCTTAACGTCCCCATCCGACGGACG
>SRR4252623_917346___Sbg5
TTAGTGATCAGAAGTTGTACACCACCAC
>SRR4252614_3856101___Sbg5
TGTCGGCATGTAGCCTACTCCTTTCGAAAA
>SRR4252610_4918381___Sbg5
GACGTCCCCATCCGACGGACGGATCA
>SRR4252608_10878290___Sbg5
GTGTATGTGTGCGGATAGGATGATGAGT
>SRR4252608_9146575___Sbg5
TGTGTGTATAGTGTTGTATTGTGTGTGTG
>SRR4252608_5379895___Sbg5
TATGATGTGAGTGACGATTATGGATATGAA
>SRR4252611_10927399___Sbg5
TGATCATTGTGTATGTGTGCGGATAGGA
>SRR4252608_3535753___Sbg5
TGTGCGGATAGGAGGATGAGTGTGTGTA
>SRR4252610_26951___Sbg5
TGTGCGGATAGGAGGATGAGTGTGTGTA
>SRR4252605_9968820___Sbg5
TCATTGTGTATGTGTGCGGATAGGAGG
>SRR4252611_742618___Sbg5
GTCCCCACAGCCGATAACCCCGGTTTTCT
>SRR4252622_4880616___Sbg5
TATTTGATCATTGTGTATGTGTGCGGAT

>SRR4252608_12025887___Sbg5
TGTATGTGTGCGGATAGGAGGATGAGTGT
>SRR4252606_4166377___Sbg5
TATGATGTGAGTGACGATTATGGATA
>SRR4252622_2665786___Sbg5
ACAATGACATATGCCTTCTCAACATA
>SRR4252606_8792599___Sbg5
TTAGTGATCAGAAGTTGTACACCACCACTC
>SRR4252613_10761306___Sbg5
TTAGTGATCAGAAGTTGTACACCACCACT
>SRR4252613_4720446___Sbg5
GACGATTATGGATATGAAAGAGGGAGA
>SRR4252611_5472778___Sbg5
CGGTTTTCTAGACCAGCTAATGGATTGGTT
>SRR4252621_5272___Sbg5
GTCCTCATCCGACGGACGGATCACTATCAA
>SRR4252611_7355697___Sbg5
TTAGTGATCAGAAGTTGTACACCACCACC
>SRR4252606_4036670___Sbg5
AGAAGTTCTACACCACCACTCCTCCT
>SRR4252611_12946510___Sbg5
CAACAATGACATATGCCTTCTCAACATA
>SRR4252606_5386833___Sbg5
TGTGTGTATAGTGTTGTATTGTGTGTGTG
>SRR4252605_10237162___Sbg5
CACAATTTAGTGATCAGAAGTTGTACA
>SRR4252613_4905143___Sbg5
CGAGCGGCTGCCGCCATAGGACGGAATTA
>SRR4252623_763717___Sbg5
TATTTGATCATTGTGTATGTGTGCGGA
>SRR4252609_16722403___Sbg5
TATTTGATCATTGTGTATGTGTGCGGAT
>SRR4252609_15686868___Sbg5
TGAGTGTGTGTATAGTGTTGTATTGTGT
>SRR4252620_6408040___Sbg5
CGGTTTTCTAGACCAGCTAATGGATTGA
>SRR4252612_5268513___Sbg5
TATGATGTGAGTGACGATTATGGATAT
>SRR4252605_7637316___Sbg5
AGGATTCGAAATTTTAATTAATTACTC
>SRR4252606_2853208___Sbg5
TCCTTTCGAAAAGCACCAAGGGGACCG
>SRR4252610_293068___Sbg5
GTCCCCATCCGACGGACGGATCACTATCAA
>SRR4252611_14950052___Sbg5
TATTTGATCATTGTGTATGTGTGCGGAT
>SRR4252610_9238084___Sbg5
GGGGTCCGACGGCTTAACGTCCCCATC
>SRR4252605_10234460___Sbg5
GGTGTACAATTTAGTGATCAGAAGTTG
>SRR4252619_11759276___Sbg5
TAGGACGGAATTAACGGGGGGTAGTATGG
>SRR4252610_371762___Sbg5
TGTGCGGATAGGAGGATGAGTGTGTG
>SRR4252605_10633469___Sbg5
TTAGTGATCAGAAGTTGTACACCACCACTCT
>SRR4252610_13056397___Sbg5
TTAGTGATCAGAAGTTGTACACCACCACTCT
>SRR4252612_8361016___Sbg5
TAGTGATCAGAAGTTGTACACCACCACTCT
>SRR4252620_4318787___Sbg5
TAGTGATCAGAAGTTGTACACCACCACTCT
>SRR4252622_5774246___Sbg5
TGTGAGTGACGATTATGGATATGAAAGAG
>SRR4252625_32672___Sbg5
TCCTTTCGAAAAGCACCAAGGGGACCGGT
>SRR4252606_3232019___Sbg5
ATGGATCGGTGTGTAAGTGTGTGAATAG
>SRR4252610_1234304___Sbg5
ACAATTTAGTGATCAGAAGTTGTACACCA

>SRR4252611_12183757___Sbg5
AGTGATCAGAAGTTGTACACCACCACTC
>SRR4252619_6162372___Sbg5
AGCTATTTGATCATTGTGTATGTGTGC
>SRR4252612_6533694___Sbg5
AGCTATTTGATCATTGTGTATGTGTA
>SRR4252621_14043275___Sbg5
CAGCTTAACGTCCCCATCCGACGGACGGA
>SRR4252612_8123787___Sbg5
CCCCATCCGACGGACGGATCACTATCAA
>SRR4252609_7577332___Sbg5
TTAACGTCCCCATCCGACGGACGGAT
>SRR4252608_783038___Sbg5
TCATTGTGTATGTGTGCGGATAGGAGGA
>SRR4252606_8700743___Sbg5
TAGTGATCAGAAGTTGTACACCACCACTC
>SRR4252611_1624947___Sbg5
GCACAATTTAGTGATCAGAAGTTGTACACCA
>SRR4252611_6084918___Sbg5
TTAGTGATCAGAAGTTGTACACCACCACTT
>SRR4252611_6766950___Sbg5
TAGTATGGTCAGTACTACGCTTGTCCCCA
>SRR4252619_214101___Sbg5
CAGTACTACGCTGGTCCCCACAGCCGA
>SRR4252612_4454530___Sbg5
GGTATGATGTGAGTGACGATTATGGA
>SRR4252611_1470190___Sbg5
CAGTTAATGGATCGGTGTGTAAGTGTGTGA
>SRR4252606_8741615___Sbg5
ATGATGTGAGTGACGATTATGGATAT
>SRR4252605_13636647___Sbg5
ATCACTATCAACAATGACGTATGCCTTCTC
>SRR4252608_6930219___Sbg5
CAATTTTGTGATCAGAAGTTGTACACCA
>SRR4252606_2798621___Sbg5
TGTATGTGTGCGGATAGGAGGATGAGTGT
>SRR4252612_6981795___Sbg5
TATGAAAGAGGGAGAAGGTGAAACCTGG
>SRR4252606_6361291___Sbg5
TGAAAGAGGGAGAAGGTGAAACCTGGTGC
>SRR4252610_3588931___Sbg5
TTAGTGATCAGAAGTTGTACACCACCACTC
>SRR4252612_7758834___Sbg5
ACAGCCGATAACCCCGGTTTTCTAGACCA
>SRR4252611_7989125___Sbg5
TGTAAGTGTGTGAATAGGGAGATGAGT
>SRR4252605_14351520___Sbg5
ACAATTTAGTGATCAGAAGTTGTACACCA
>SRR4252606_3645382___Sbg5
TTAGTGATCAGAAGTTGTACACCACCACTT
>SRR4252612_2054543___Sbg5
TTAGTGATCAGAAGTTGTACACCACCACTT
>SRR4252619_6453600___Sbg5
TTTTAGTGATCAGAAGTTGTACACCACCAC
>SRR4252609_2912657___Sbg5
AGTGTGTGTATAGTGTTGTATTGTGTGTGT
>SRR4252610_7049079___Sbg5
ACGGACGGATCACTATCAACAATGACAT
>SRR4252608_13236232___Sbg5
ACAATTTAGTGATCAGAAGTTGTACACCA
>SRR4252624_6025438___Sbg5
TCAGTACTACGCTGGTCCCCACAGCCGA
>SRR4252614_9703292___Sbg5
TATTTGATCATTGTGTATGTGTGCGGAT
>SRR4252622_5533538___Sbg5
TATGTGTGCGGATAGGAGGATGAGTGTGT
>SRR4252606_2014248___Sbg5
TCACTATCAACAATGACGTATGCCTTCT
>SRR4252609_6448106___Sbg5
TATGAAAGAGGGAGAAGGTGAAACCCGGTG

>SRR4252607_3308481___Sbg5
CCCCACAGCCGATAACCCCGGTTTTCTA
>SRR4252611_30539___Sbg5
TACCGAGCGGCTGCCGCCATAGGACGGATT
>SRR4252623_3645018___Sbg5
TTAGTGATCAGAAGTTGTACACCACCACTC
>SRR4252605_15164931___Sbg5
TCATTGTGTATGTGTGCGGATAGGAGGA
>SRR4252612_1151475___Sbg5
TCATTGTGTATGTGTGCGGATAGGAGGA
>SRR4252621_6143991___Sbg5
CCCCACAGCCGATAACCCCGGTTTTCTA
>SRR4252606_1466007___Sbg5
GTCATTGCGGAGGAGTTCGGGATTTA
>SRR4252606_9095260___Sbg5
TTTGATCATTGTGTATGTGTGCGGATA
>SRR4252606_7647867___Sbg5
TCATTGTGTATGTGTGCGGATAGGAGGA
>SRR4252618_424353___Sbg5
TGTGTATGTGTGCGGATAGGAGGATG
>SRR4252611_894982___Sbg5
TTAGTGATCAGAAGTTGTACACCACCACT
>SRR4252610_12662907___Sbg5
TATTTGATCATTGTGTATGTGTGCGG
>SRR4252625_7071205___Sbg5
TCATTGTGTATGTGTGCGGATAGGAGGA
>SRR4252611_5140914___Sbg5
TGAAACCCGGTGCCGGCATGTAGTCTACT
>SRR4252618_10454325___Sbg5
TATTTGATCATTGTGTATGTGTGCGGAT
>SRR4252606_9717577___Sbg5
TGTGTATGTGTGCGGATAGGAGGATGAGT
>SRR4252606_2732263___Sbg5
TGATCATTGTGTATGTGTGCGGATAGGAG
>SRR4252605_12279073___Sbg5
TATGATGTGAGTGACGATTATGGATATG
>SRR4252619_13119860___Sbg5
TACAATTTAGTGATCAGAAGTTGTACA
>SRR4252611_1893307___Sbg5
TACCGAGCGGCTGCCGCCATAGGACGGAA
>SRR4252611_10207563___Sbg5
TTAGTGATCAGAAGTTGTACACCACCACTC
>SRR4252608_5081866___Sbg5
GTCCCCATACGACGGACGGATCACTATCAA
>SRR4252620_8404267___Sbg5
TCGGTGTGTAAGTGTGTGGATAGGGGGATG
>SRR4252620_10898702___Sbg5
AGTGATCAGAAGTTGTACACCACCACT
>SRR4252608_7592291___Sbg5
TTAGTGATCAGAAGTTGTACACCACCACTT
>SRR4252610_11048776___Sbg5
TATTTGATCATTGTGTATGTGTGCGGAT
>SRR4252607_14516037___Sbg5
TAGGACGGAATTAACGGGGGGTAGTATGT
>SRR4252608_9018861___Sbg5
TAGGACGGAATTAACGGGGGGTAGTATGT
>SRR4252611_14352289___Sbg5
TAGGACGGAATTAACGGGGGGTAGTATGT
>SRR4252605_13018224___Sbg5
GCCATTGCGGAGGAGTTCGGGATTTAA
>SRR4252614_8885834___Sbg5
ACAATTTAGTGATCAGAAGTTGTACA
>SRR4252605_12233997___Sbg5
TTAGTGATCAGAAGTTGTACACCACCACTT
>SRR4252611_168563___Sbg5
TTAGTGATCAGAAGTTGTACACCACCACTT
>SRR4252612_1473035___Sbg5
TTAGTGATCAGAAGTTGTACACCACCACTT
>SRR4252611_712575___Sbg5
CATGTAGCCTACTCCTTTCGAAAAGC

>SRR4252610_10109854___Sbg5
ACAATTTAGTGATCAGAAGTTGTACACCA
>SRR4252611_686915___Sbg5
TATTTGATCATTGTGTATGTGTGCGGAT
>SRR4252610_10768764___Sbg5
TCATTGTGTATGTGTGCGGATAGGAGGA
>SRR4252605_14630066___Sbg5
TCACTATCAACAATGACATATGCCTTCT
>SRR4252622_6633909___Sbg5
CCCCACAGCCGATAACCCCGGTTTTCTA
>SRR4252611_6857584___Sbg5
TGATCATTGTGTATGTGTGCGGATAGGA
>SRR4252606_3802887___Sbg5
TCATTGTGTATGTGTGCGGATAGGAGGA
>SRR4252610_8808256___Sbg5
TCATTGTGTATGTGTGCGGATAGGAGGA
>SRR4252606_8662692___Sbg5
TTAGTGATCAGAAGTTGTACACCACCACTC
>SRR4252611_8956375___Sbg5
TATTTGATCATTGTGTATGTGTGCGGA
>SRR4252621_13897935___Sbg5
TATGATGTGAGTGACGATTATGGATATG
>SRR4252615_14787858___Sbg5
TACATAGTCATTGCGGAGGAGTTCGG
>SRR4252611_11912444___Sbg5
ACAATTTAGTGATCAGAAGTTGTACACCA
>SRR4252623_11672942___Sbg5
TATTTGATCATTGTGTATGTGTGCGGAT
>SRR4252610_12845420___Sbg5
TCATTGTGTATGTGTGCGGATAGGAG
>SRR4252618_4501567___Sbg5
TCAGTACTACGCTGGTCCCCACAGCCGATA
>SRR4252611_9978125___Sbg5
TATAGTGTTGTATTGTGTGTGTGGTG
>SRR4252618_1824688___Sbg5
ATTAATGGATCGGTGTGTAAGTGTGTGA
>SRR4252609_2770123___Sbg5
TTAGTGATCAGAAGTTGTACACCACCACTC
>SRR4252610_3758057___Sbg5
TTAGTGATCAGAAGTTGTACACCGCCACTC
>SRR4252608_10888130___Sbg5
TGTGCGGATAGGAGGATGAGTGTGTGTA
>SRR4252625_2051434___Sbg5
TACCGAGCGGCTGCCGCCATAGGACGGAAA
>SRR4252614_2440992___Sbg5
TGTGGTATGATGTGAGTGACGATTATGG
>SRR4252611_7074670___Sbg5
GTCCCCATCCGACGGACGGATCACTATCA
>SRR4252620_5052733___Sbg5
TCAACAATGACATATGCCTTCTCTACATA
>SRR4252610_2303505___Sbg5
TTAGTGATCAGAAGTTGTACACCACCACTC
>SRR4252605_356780___Sbg5
TACCGAGCGGCTGCCGCCATAGGGCGGAA
>SRR4252610_11996313___Sbg5
TATGATGTGAGTGACGATTATGGATA
>SRR4252605_13926562___Sbg5
TGTAGCCTACTCCTTTCGAAAAACACCA
>SRR4252612_1499624___Sbg5
CGTCCCCATCCGACGGACGGATCACTATCAA
>SRR4252620_5722305___Sbg5
GATCACTATCAACAATGACGTATGCCTTCTC
>SRR4252606_5767739___Sbg5
TTAGTGATCAGAAGTTGTACACCACCACTC
>SRR4252611_12341581___Sbg5
TTAGTGATCAGAAGTTGTACACCACCAC
>SRR4252605_1627682___Sbg5
TGATCAGAAGTTGTACACCACCACTCC
>SRR4252608_8858758___Sbg5
TGTATGTGTGCGGATAGGAGGATCAGTGT

```
>SRR4252626_5453147___Sbg5
TGTATGTGTGCGGATAGGAGGATCAGTGT
>SRR4252606_5986578___Sbg5
TATGTGTGCGGATAGGAGGATCAGTGT
>SRR4252608_12152732___Sbg5
TATGTGTGCGGATAGGAGGATCAGTGTG
>SRR4252624_9406464___Sbg5
TCATTGTGTATGTGTGCGGATAGGAGGA
>SRR4252607_14770047___Sbg5
CAGTACTACGCTGGTCCCCACAGCCGA
>SRR4252619_1547195___Sbg5
CCCCACAGCCGATAACCCCGGTTTTCTA
>SRR4252612_7773326___Sbg5
TGCGGTATGATGTGAGTGACGATTATGGA
>SRR4252617_12613580___Sbg5
CACTATCAACAATGACATATGCCTTCTTT
>SRR4252616_11496395___Sbg5
TATGATGTGAGTGACGATTATGGATATG
>SRR4252614_6028061___Sbg5
TAGTGATCAGAAGTTGTACACCACCACTC
>SRR4252619_8218492___Sbg5
TATGATGTGAGTGACGATTATGGATATAA
>SRR4252607_8837391___Sbg5
TTAGTGATCAGAAGTTGTACACCACCACT
>SRR4252605_10502042___Sbg5
ACAATTTAGTGATCAGAAGTTGTACACCA
>SRR4252612_3883819___Sbg5
CAGTTAATGGATCGGTGTGTAAGTGTGTGA
>SRR4252605_5107716___Sbg5
TTAGTGATCAGAAGTTGTACACCACCACTC
>SRR4252605_5808845___Sbg5
TTAGTGATCAGAAGTTGTACACCACCACTCT
>SRR4252610_6647476___Sbg5
TTAGTGATCAGAAGTTGTACACCACCACTC
>SRR4252625_13003039___Sbg5
TTAGTGATCAGAAGTTGTACACCACCACTCT
>SRR4252610_5117527___Sbg5
TATTTGATCATTGTGTATGTGTGCGGAT
>SRR4252610_5230755___Sbg5
TATTTGATCATTGTGTATGTGTGCGGAT
>SRR4252611_7799027___Sbg5
TATTTGATCATTGTGTATGTGTGCGGA
>SRR4252608_12094736___Sbg5
ACGGACGGATCACTATCAACAATGACAT
>SRR4252620_3638735___Sbg5
TAGGACGGAATTAACGGGGGGTAGTATGG
>SRR4252610_11710277___Sbg5
TGTGTGGATAGGAGGATGAGTGTGTGTA
>SRR4252608_3899685___Sbg5
GTCCCCATCCGACGGACGGATCACTATCAA
>SRR4252621_9016137___Sbg5
TGTGGTATGATGTGAGTGACGATTATGG
>SRR4252605_14378528___Sbg5
ACAATTTAGTGATCAGAAGTTGTACA
>SRR4252611_3193212___Sbg5
TACCGAGCGGCTGCCGCCATAGGACGGAAC
>SRR4252610_7860843___Sbg5
ATTTGATCATTGTGTATGTGTGCGGA
>SRR4252611_13651664___Sbg5
ATTTGATCATTGTGTATGTGTGCGGATA
>SRR4252608_6002591___Sbg5
TTAGTGATCAGAAGTTGTACACCACCATTC
>SRR4252611_8391486___Sbg5
TAGTGATCAGAAGTTGTACACCACCACTC
>SRR4252614_3085263___Sbg5
GATCATTGTGTATGTGTGCGGATAGGA
>SRR4252605_11956349___Sbg5
CGGCTTAACGTCCCCATCCGACGGACG
>SRR4252606_7379972___Sbg5
TGTGTATGTGTGCGGATAGGAAGATGAGT
```

>SRR4252605_14674397___Sbg5
ACAATTTAGTGATCAGAAGTTGTACA
>SRR4252608_6122909___Sbg5
ACAATTTAGTGATCAGAAGTTGTACA
>SRR4252614_8619912___Sbg5
CCCACAGCCGATAACCCCGGTTTTCTA
>SRR4252624_2349507___Sbg5
TAATGGATCGGTGTGTAAGTGTGTGGATA
>SRR4252609_7484803___Sbg5
GTACAATATAGTGATCAGAAGTTGTA
>SRR4252608_11518046___Sbg5
ATAGTGTTGTATTGTGTGTGTGATGTGG
>SRR4252611_12183976___Sbg5
ACAATTTAATGATCAGAAGTTGTACACCA
>SRR4252614_1901519___Sbg5
GTGGTATGATGTGAGTGACGATTATGGA
>SRR4252605_8249004___Sbg5
TATGAAAGAGGGAGAAGGTGAAACCTGG
>SRR4252610_5613862___Sbg5
TATGAAAGAGGGAGAAGGTGAAACCTGG
>SRR4252610_909260___Sbg5
TATGAAAGAGGGAGAAGGTGAAACCTGG
>SRR4252612_4175627___Sbg5
GGCATGTAGCCTACTCCTTTCGAAAA
>SRR4252624_4483468___Sbg5
TATTTGATCATTGTGTATGTGTGCGGA
>SRR4252607_3186205___Sbg5
CGAGCGGATGCCGCCATAGGACGGAA
>SRR4252621_4875439___Sbg5
TTAGTGATCAGAAGTTGTACACCACCATTC
>SRR4252610_7996888___Sbg5
TAGTGATCAGAAGTTGTACACCACCATTC
>SRR4252621_11006348___Sbg5
TATGAAAGAGGGAGAAGGCGAAACCCGGT
>SRR4252606_5001710___Sbg5
TAGTGATCAGAAGTTGTACACCACCACTCA
>SRR4252611_10895968___Sbg5
ACGTGAGTGACGATTATGGATATGAA
>SRR4252606_5766989___Sbg5
TTAGTGATCAGAAGTTGTACACCACCACTC
>SRR4252610_4943762___Sbg5
TTTAGTGATCAGAAGTTGTACACCACCAT
>SRR4252606_7295249___Sbg5
ATGTGTGCGGATAGGAGGATGAGTGT
>SRR4252619_11499556___Sbg5
CGGATAGGAGGATGAGTGTGTATATA
>SRR4252611_2707603___Sbg5
TCCTTTCGAAAAGCACCAAGGGTACCGGC
>SRR4252611_8280143___Sbg5
GTCCCCATCCGACGGACGGATCACTATCAA
>SRR4252606_2151982___Sbg5
TTAGTGATCAGAAGTTGTACACCACCACTC
>SRR4252607_4084672___Sbg5
TCTAGACCAGCTATTTGATCATTGTGTAT
>SRR4252606_2376644___Sbg5
TCATTGTGTATGTGTGCGGATAGGAGG
>SRR4252612_4466425___Sbg5
TCATTGTGTATGTGTGCGGATAGGAG
>SRR4252609_4760146___Sbg5
TTAGTGATCAGAAGTTGTACACCACCACT
>SRR4252611_14496429___Sbg5
TATGAAAGAGGGAGAAGGTGAAACCCGA
>SRR4252605_12526659___Sbg5
TTAGTGATCAGAAGTTGTACACCACCACTC
>SRR4252610_9994445___Sbg5
TATGTGTGCGGATAGGAGGATGAGTGTG
>SRR4252609_4635707___Sbg5
AAGTGACGATTATGGATATGAAAGAGG
>SRR4252611_3150007___Sbg5
TCAGTACTATGCTGGTCCCCACAGCCGA

```
>SRR4252606_7452098___Sbg5
TAACGTCCCCATCCGACGGACGGATCA
>SRR4252606_159783___Sbg5
TGTGGTATGATGTGAGTGACGATTAC
>SRR4252612_4020514___Sbg5
CCACAGCCGATAACCCCGGTTTTCTA
>SRR4252608_13278241___Sbg5
TTAATGATCAGAAGTTGTACACCACCACTC
>SRR4252613_1644257___Sbg5
CCACAGCCGATAACCCCGGTTTTCTA
>SRR4252610_3403777___Sbg5
TGGATATGAAAGAGGGAGACGGTGAAACC
>SRR4252610_4724311___Sbg5
TAAGTGTGTGGATAGGGGGATGAGTGT
>SRR4252605_1399331___Sbg5
GCATGTAGTCTACTCCTTTCGAAAAGCA
>SRR4252612_4013164___Sbg5
TCACTATCAACAATGACATATGCCTTCTC
>SRR4252621_2816391___Sbg5
TCCCCATCCGACGGACGGTTCACTATCAAC
>SRR4252610_6121252___Sbg5
GACGGACGGTTCACTATCAACAATGACAT
>SRR4252625_10544276___Sbg5
TTAGTGATCAGAAGTTGTACACCACCAT
>SRR4252625_7201770___Sbg5
TTAGTGATCAGAAGTTGTACACCACCAT
>SRR4252621_3136018___Sbg5
TTAATGATCAGAAGTTGTACACCACCACTC
>SRR4252613_11115411___Sbg5
GGACGGATCACTATCAACAATGACATAT
>SRR4252610_2903436___Sbg5
CCAATTTAGTGATCAGAAGTTGTACACCA
>SRR4252612_4967912___Sbg5
TATTTGATCATTGTGTATGTGTGCGGAT
>SRR4252611_14120541___Sbg5
TTAGTGATCAGAAGTTGTACACCACCACT
>SRR4252605_8977498___Sbg5
AGTGACGATTATGGATATGAAAGAGGA
>SRR4252611_5650761___Sbg5
ATAGCTCAACAGCCCGATGTGGGCCAAG
>SRR4252619_11629196___Sbg5
TCATTGTGTATGTGTGCGGATAGGAGG
>SRR4252618_5453915___Sbg5
GATCAGAAGTTGTACACCACCACTCC
>SRR4252610_8689005___Sbg5
TTGATCATTGTGTATGTGTGCGGATAGGA
>SRR4252610_6476405___Sbg5
GAAATTGGTGTACAATTTAATGATCAGAA
>SRR4252610_1528605___Sbg5
TTAGTGATCAGAAGTTGTACACCACCACTC
>SRR4252623_8935355___Sbg5
TATGTGTGCGGATAGGAGAATGAGTGTG
>SRR4252605_10476196___Sbg5
AGTCCCCATCCGACGGACGGATCACTATCAA
>SRR4252620_10254407___Sbg5
AGTCCCCATCCGACGGACGGATCACTATCA
>SRR4252612_6987902___Sbg5
TACCGAGCGGCTGCCGCCATAGGGCGGAA
>SRR4252605_1088848___Sbg5
ACAGTTTAGTGATCAGAAGTTGTACA
>SRR4252614_4804229___Sbg5
TACCGAGCGGCTGCCGCCATAGGGCGGAATT
>SRR4252626_133613___Sbg5
GGTATGACGTGAGTGACGATTATGGATA
>SRR4252608_1210284___Sbg5
GCAATTTAGTGATCAGAAGTTGTACA
>SRR4252608_781277___Sbg5
GCAATTTAGTGATCAGAAGTTGTACACCA
>SRR4252606_8491717___Sbg5
TTAGTGATCAGAAGTTGTACACCACCACTC
```

```
>SRR4252610_4686107___Sbg5
TTAGTGATCAGAAGTTGTACACCACCACTC
>SRR4252605_12918209___Sbg5
TGATCATTGTGTATGTGTGCGGATAGGA
>SRR4252609_14761317___Sbg5
TATTTGATCATTGTGTATGTGTGCGG
>SRR4252611_7547180___Sbg5
TATTTGATCATTGTGTATGTGTGCGGAT
>SRR4252615_9582746___Sbg5
TCACTATCAACAATGACGTATGCCTTCTC
>SRR4252610_3329811___Sbg5
TTAGTGATCAGAAGTTGTACACCACCACTC
>SRR4252611_1597337___Sbg5
AGTCCCCATCCGACGGACGGATCACTATCAA
>SRR4252611_4211869___Sbg5
TATTTGATCATTGTGTATGTGTGCGGAT
>SRR4252612_5353535___Sbg5
TTAGTGATCAGAAGTTGTACACCACCACTC
>SRR4252606_5129782___Sbg5
TAGTGATCAGAAGTTGTACACCACCACTC
>SRR4252616_10001785___Sbg5
GACGGACGGATCACTATCAACAATGACAT
>SRR4252611_6420902___Sbg5
GTGCACAATTTAGTGATCAGAAGTTGTA
>SRR4252610_7333250___Sbg5
TTAGTGATCAGAAGTTGTACACCACCACTC
>SRR4252610_2570216___Sbg5
GATGTGTGTGACGGTTATGGATATGAA
>SRR4252623_14501770___Sbg5
TTAGTGATCAGAAGTTGTACACCACCACTC
>SRR4252605_4543202___Sbg5
ACAATTTAGTGATCAGAAGTTGTACACCA
>SRR4252605_14178721___Sbg5
TTAGTGATCAGAAGTTGTACACCACCACTC
>SRR4252606_6230556___Sbg5
TCATTGTGTATGTGTGCGGATAGGAGG
>SRR4252605_12840198___Sbg5
GATCCCCACAGCCGATAACCCCGGTTTTCTA
>SRR4252611_11605342___Sbg5
TATTTGATCATTGTGTATGTGTGCGGAT
>SRR4252613_7793825___Sbg5
CCCCACAGCCGATAACCCCGGTTTTCTA
>SRR4252608_3155046___Sbg5
TTGATCATTGTGTATGTGTGCGGATAGGA
>SRR4252623_221783___Sbg5
TTAGTGATCAGAAGTTGTACACCACCACTC
>SRR4252615_10810942___Sbg5
TATTTGATCATTGTGTATGTGTGCGGAT
>SRR4252621_1487534___Sbg5
ATGTAGCCTACTCCTTTCGAAAAACACCA
>SRR4252605_3511529___Sbg5
TAGTGATCAGAAGTTGTACATCACCACTCC
>SRR4252612_4790528___Sbg5
TTAGTGATCAGAAGTTGTACATCACCACT
>SRR4252608_12885627___Sbg5
GTCAGTACTACGCTGATCCCCACAGCCGA
>SRR4252619_15112682___Sbg5
ACAATTTAGTGATCAGAAGTTGTACAC
>SRR4252605_14979419___Sbg5
TAAGTGTGTGGATAGGGGGATGAGTGTGT
>SRR4252606_1848907___Sbg5
TGTGAGTGACGATTATGGATATGAAAGAGG
>SRR4252618_10549612___Sbg5
TATGATGTGAGTGACGATTATGAATATG
>SRR4252616_12101654___Sbg5
CCCCATCCGACGGACGGATCACTATCAAC
>SRR4252610_6984185___Sbg5
TTAGTGATCAGAAGTTGTACACCACCACTC
>SRR4252610_1703710___Sbg5
ACAACCGATAGCCCCGGTTTTCTAGACCA
```

>SRR4252606_9089177___Sbg5
TTAGTGATCAGAAGTTGTACACCACCACTC
>SRR4252606_9573529___Sbg5
TGTAAGTGTGTGAATAGGGAGATGAGTGT
>SRR4252605_11150450___Sbg5
TATGAAAGAGGGAGAAGGTGAAACCCGG
>SRR4252613_187450___Sbg5
TGCACAATTTAGTGATCAGAAGTTGTA
>SRR4252605_4978584___Sbg5
TTAGTGATCAGAAGTTGTACACCACCACTC
>SRR4252607_9674369___Sbg5
TTGATCATTGTGTATGTGTGCGGATAGG
>SRR4252612_7831734___Sbg5
TCATTGTGTATGTGTGCGGATAGGAGGA
>SRR4252606_7595702___Sbg5
TGTATGTGTGCGGATAGGAGGATGAGTGT
>SRR4252612_8339844___Sbg5
ACGTGAGTGACGATTATGGATATGAAAGAG
>SRR4252610_13189880___Sbg5
TCGAAAAACACCAAGGGGTCCGACGGCTT
>SRR4252612_406302___Sbg5
AGTGATCAGAAGTTGTACACCACCACTCC
>SRR4252625_2489280___Sbg5
TGTGTATGTGTGCGGATAGGAGGATGAGT
>SRR4252610_9563746___Sbg5
TGTATGTGTGCGGATAGGAGGATGAGTGT
>SRR4252613_9545588___Sbg5
ACGGATCACTATCAACAATAACATATG
>SRR4252610_7273911___Sbg5
TATTTGATCATTGTGTATGTGTGCGGA
>SRR4252605_12388368___Sbg5
TCATTGTGTATGTGTGCGGATAGGAGG
>SRR4252608_9209958___Sbg5
GATGTGAGTGACGATTATGAATATGA
>SRR4252607_3002819___Sbg5
TAGGACGGAATTAACGGGGGGTAGTATGG
>SRR4252625_1964644___Sbg5
GTCCCCATCCGACGGACGGATCACTATCAA
>SRR4252612_1800015___Sbg5
TGTAAGTGTGTGAATAGGGGGATGAGTT
>SRR4252610_5519514___Sbg5
CTAACGTCCCCATCCGACGGACGGATCA
>SRR4252611_1418358___Sbg5
TACCGAGCGGCTGCCGCCATAGGGCGGAG
>SRR4252606_6376911___Sbg5
AAGTTGTACACCACCACTCCTCCTAGTCTC
>SRR4252606_8611953___Sbg5
AGTGATCAGAAGTTGTACACCACCATTCC
>SRR4252623_10341946___Sbg5
TCCCCATCCGACGGACGGATCAATATCAACA
>SRR4252605_7266061___Sbg5
GAAATTGGTGTACAATTTAATGATCAGA
>SRR4252611_11017154___Sbg5
TAATGGTTCGGTGTGTAAGTGTGTGG
>SRR4252622_9333834___Sbg5
TAATGGTTCGGTGTGTAAGTGTGTGGA
>SRR4252619_10191907___Sbg5
ACAATTTAATGATCAGAAGTTGTACACCA
>SRR4252606_7169168___Sbg5
TTGATCATTGTGTATGTGTGCGGATAGG
>SRR4252611_9532743___Sbg5
TGATGTGAGTGACGATTATGGATATG
>SRR4252610_9549262___Sbg5
TCCTTTCGAAAAGCACCAAGGGGACCGGC
>SRR4252607_11810936___Sbg5
TCTTAACGTCCCCATCCGACGGACGGA
>SRR4252605_8266941___Sbg5
ATCATTGTGTATGTGTGCGGATAGAAGG
>SRR4252608_10047634___Sbg5
GAGGATGAGTGTGTGTATAGTGTTGTAT

```
>SRR4252619_504833___Sbg5
TTAGTGATCAGAAGTTGTATACCACCACT
>SRR4252620_3632273___Sbg5
TGTGTGTATAGTGTTGTATTGTGTGTGT
>SRR4252616_1428934___Sbg5
GCTTACCGAGCGGCTGCCGCCATAGGACG
>SRR4252611_14456097___Sbg5
TATGATGTGAGTGACGATTATGGATATT
>SRR4252607_6735032___Sbg5
TTAGTGATCAGAAGTTGTACACCACCACTT
>SRR4252610_12436186___Sbg5
TTAGTGATCAGAAGTTGTACACCACCACTT
>SRR4252606_129830___Sbg5
TCCTTTCGAAAAGCACCAAGGGGACCGA
>SRR4252610_6052147___Sbg5
TCCTTTCGAAAAGCACCAAGGGGACCGA
>SRR4252611_2336839___Sbg5
AGTCCCCATCCGACGGACGGATCACTATCAA
>SRR4252611_7269095___Sbg5
AGTCCCCATCCGACGGACGGATCACTATCAA
>SRR4252611_7426067___Sbg5
TGTACAATTTAGTGATCAGAAGTTGTA
>SRR4252613_5090222___Sbg5
AGTTAATGGATCGGTGTGTAAGTGTG
>SRR4252613_11305361___Sbg5
TAATGGATCGGTGTGTAAGTGTGTGAATA
>SRR4252608_11765464___Sbg5
TCATTGTGTATGTGTGCGGATAGGAGGA
>SRR4252618_7607505___Sbg5
ACAATTTAGTGATCAGAAGTTGTACAC
>SRR4252611_10088947___Sbg5
CACAATTTAGTGATCAGAAGTTGTACA
>SRR4252612_6675134___Sbg5
TCAGTACTATGCTGGTCCCCACAGCCGA
>SRR4252616_5722999___Sbg5
CAACAATGACATATGCCTTCTCTACATA
>SRR4252610_4418525___Sbg5
ACAATTTATTGATCAGAAGTTGTACACCA
>SRR4252608_6781956___Sbg5
TGAAACCCGGTGCCGGCATGTAGCCT
>SRR4252608_11291024___Sbg5
AGTCCCCATCCGACGGACGGATCACTATCA
>SRR4252611_11221820___Sbg5
AGTCCCCATCCGACGGACGGATCACTATCAA
>SRR4252609_4478428___Sbg5
TAGGACGGAATTAACGGGGGGTAGTATGG
>SRR4252613_10814992___Sbg5
TATAGCTCAACAGCCCGATGTGGGCCAAGG
>SRR4252610_4374002___Sbg5
TAGGACGGAATTAACGGGGGGTAGTATGG
>SRR4252611_10897787___Sbg5
TAGGACGGAATTAACGGGGGATAGTATGG
>SRR4252611_14533620___Sbg5
GACGGATCACTATCAACAATGACATATGCA
>SRR4252610_6424638___Sbg5
TATTTGATCATTGTGTATGTGTGCGG
>SRR4252615_5450802___Sbg5
ATTTGATCATTGTGTATGTGTGCGGATA
>SRR4252605_14301139___Sbg5
TTAGTGATCAGAAGTTGTACACCACCACTC
>SRR4252613_1041566___Sbg5
AGTCCCCATCCGACGGACGGATCACTATCA
>SRR4252610_11734436___Sbg5
TTAGTGATCAGAAGTTGTACACCACCACTC
>SRR4252615_145245___Sbg5
GACCCCACAGCCGATAACCCCGGTTTTCTA
>SRR4252612_8621284___Sbg5
ACCCCACAGCCGATAACCCCGGTTTTCTA
>SRR4252612_6070015___Sbg5
TATGATATGAGTGACGATTATGGATAT
```

>SRR4252606_1694878___Sbg5
TAGGACGGAATTAACGGGGGGTAGTATGG
>SRR4252611_1222453___Sbg5
TTAGTGATCAGAAGTTGTACACCACCACTC
>SRR4252616_8663073___Sbg5
GGACGGAATTAACGGGGGGTAGTATA
>SRR4252610_1894028___Sbg5
TTAGTGATCAGAAGTTGTACACCACCACTC
>SRR4252611_6158685___Sbg5
TATTTGATCATTGTGTATGTGTGCGGA
>SRR4252605_6903836___Sbg5
TCATTGTGTATGTGTGCGGATAGGAGA
>SRR4252610_4160510___Sbg5
TGAGTGTGTGTATAGTGTTGAATTGTGT
>SRR4252619_7023377___Sbg5
TTAGTGATCAGAAGTTGTACACCACCACTC
>SRR4252605_3324261___Sbg5
TAGTGATCAGAAGTTGTACACCACCACTC
>SRR4252612_6974553___Sbg5
TTAGTGATCAGAAGTTGTACACCACCACTC
>SRR4252611_13731647___Sbg5
CCAGTTAATGGATCGGTGTGTAAGTGTG
>SRR4252612_5037871___Sbg5
TAACGTCCCCATCCGACGGACGGATCACT
>SRR4252605_8250994___Sbg5
TTTAGTGATCAGAAGTTGTACACCACCATT
>SRR4252605_4252267___Sbg5
TTAGTGATCAGAAGTTGTACACCACCATTC
>SRR4252608_10984075___Sbg5
TCACTATCAACAATGACATATGCCTA
>SRR4252610_8772168___Sbg5
TTAGTGATCAGAAGTTGTACACCACCACTC
>SRR4252618_7347091___Sbg5
TAACGTCCCCATCCGACGGACGGATCACTA
>SRR4252605_11872359___Sbg5
TATTTGATCATTGTGTATGTGTGCGG
>SRR4252624_6485535___Sbg5
TTAGTGATCAGAAGTTGTACACCACCACTC
>SRR4252610_3034013___Sbg5
TAGTGATCAGAAGTTGTACACCACCACTCT
>SRR4252613_3473236___Sbg5
CCCACAGCCGATAACCCCGGTTTTCTA
>SRR4252611_1876143___Sbg5
CCGATAACCCCGGTTTTCTAGACCAGT
>SRR4252610_3713915___Sbg5
TCCCCATCCGACGGACGGATCACTATCAA
>SRR4252610_12391830___Sbg5
AGCTAATGGATTGGTGTGTAAGTGTGT
>SRR4252611_5836749___Sbg5
GTGTATGTGTGCGGATAGGAGGATGAGT
>SRR4252610_8646578___Sbg5
TTAGTGATCAGAAGTTGTACACCACCACTCA
>SRR4252611_610039___Sbg5
TTAGTGATCAGAAGTTGTACACCACCACTCA
>SRR4252614_2324222___Sbg5
TTAGTGATCAGAAGTTGTACACCACCACTCA
>SRR4252609_12668098___Sbg5
CAGCTAATGGATTGGTGTGTAAGTGTGT
>SRR4252623_14339386___Sbg5
ACAATTTAGTGATCAGAAGTTGTACACCA
>SRR4252606_2605796___Sbg5
TTAGTGATCAGAAGTTGTACACCACT
>SRR4252610_8788481___Sbg5
TAGGACGGAATTAACGGGGGGTAGTATTA
>SRR4252612_6338779___Sbg5
TTAGTGATCAGAAGTTGTACACCACCACTC
>SRR4252613_11178970___Sbg5
CCATCCGACGGACGGATCACTATCAA
>SRR4252611_1229142___Sbg5
AACGTCCCCATCCGACGGACGGATCACTA

>SRR4252606_7106550___Sbg5
TCATTGTGTATGTGTGCGGATAGGAGG
>SRR4252610_3853327___Sbg5
TCATTGTGTATGTGTGCGGATAGGAGG
>SRR4252611_14952518___Sbg5
CCATCCGACGGACGGATCACTATCAA
>SRR4252611_6794382___Sbg5
TGATCATTGTGTATGTGTGCGGATAGGA
>SRR4252606_7030265___Sbg5
TCATTGTGTATGTGTGCGGATAGGAGGA
>SRR4252615_9726310___Sbg5
TCTCAACATAGTCATTGCGGAGGAGTTT
>SRR4252605_4356156___Sbg5
TATTTGATCATTGTGTATGTGTGCGGAT
>SRR4252625_5154226___Sbg5
TCTCAACATAGTCATTGCGGAGGAATTCGG
>SRR4252608_2337981___Sbg5
TTTGATCATTGTGTATGTGTGCGGATAG
>SRR4252610_1333915___Sbg5
ATGATGTGAGTGACGATTATGGATATGAA
>SRR4252621_8914709___Sbg5
AACGTCCCCATCCGACGGACGGATCACTATC
>SRR4252610_4625292___Sbg5
TATGTGTGCGGATAGGAGGATGAGTGTGT
>SRR4252610_12249313___Sbg5
GACGGACGGATCACTATCAACAATGACAC
>SRR4252617_16618031___Sbg5
TGGTATGATGTGAGTGACGATTATGGATA
>SRR4252615_10419755___Sbg5
TATAGCTCAACAGCCCGATGTGGGCCAA
>SRR4252612_2365179___Sbg5
TATGAAAGAGGGAGAAGGTGAAACCCGGC
>SRR4252606_4824922___Sbg5
TATTTGATCATTGTGTATGTGTGCGG
>SRR4252624_1617169___Sbg5
TCCCCATCCGACGGACGGATCACTATCG
>SRR4252609_14015150___Sbg5
TTAGTGATCAGAAGTTGTACACCACCACTC
>SRR4252606_5028217___Sbg5
AACGTCCCCATCCGACGGACGGATCACTATC
>SRR4252610_881679___Sbg5
TCATTGTGTATGTGTGCGGATAGGAGGA
>SRR4252608_9256175___Sbg5
GATGTGAGTGACGATTATGAATATGA
>SRR4252610_3056365___Sbg5
AATGATGTGAGTGACGATTATGGATATGAA
>SRR4252611_14827414___Sbg5
TGAAACCCGGTGCCGGCATGTAGCCTAT
>SRR4252609_486582___Sbg5
ACAATTTAGTGATCAGAAGTTGTACA
>SRR4252612_7104697___Sbg5
TGTGCGGATAGGAGGATGAGTGTGTGT
>SRR4252610_10206793___Sbg5
TGCCTTCTCTACATAGTCATTGCGGA
>SRR4252610_2223071___Sbg5
TTAGTGATCAGAAGTTGTACACCACCACTC
>SRR4252606_4554084___Sbg5
TAGGACGGAATTAACGGGGAGTAGTATG
>SRR4252612_1035362___Sbg5
TAATGGATCGGTGTGTAAGTGTGTGAAT
>SRR4252625_13521548___Sbg5
CGGCTTAACGTCCCCATCCGACGGACGGA
>SRR4252611_585792___Sbg5
TTAGTGATCAGAAGTTGTACACCACCACTC
>SRR4252606_9776034___Sbg5
TAGTACTACGCTGGTCCCCACAGCCGATAGC
>SRR4252612_4910413___Sbg5
GTGCACAATTTAGTGATCAGAAGTTGTACA
>SRR4252610_1904909___Sbg5
TTAGTGATCAGAAGTTGTACACCACCACTC

>SRR4252608_10424531___Sbg5
TATGATGTGAGTGACGATTATGGATATA
>SRR4252611_10048789___Sbg5
TATGAAAGAGGGAGAAGGTGAAACCCGG
>SRR4252606_3056239___Sbg5
TATTTGATCATTGTGTATGTGTGCGGAT
>SRR4252606_5075949___Sbg5
TCATTGTGTATGTGTGCGGATAGGAGGA
>SRR4252605_12883219___Sbg5
CAATTTAGTGATCAGAAGTTGTACACCA
>SRR4252610_13616028___Sbg5
TCATTGTGTATGTGTGCGGATAGGAGGA
>SRR4252606_4772747___Sbg5
TTAGTGATCAGAAGTTGTACACCACCATTC
>SRR4252607_12610598___Sbg5
TAGTGATCAGAAGTTGTACACCACCATTC
>SRR4252608_3931729___Sbg5
TATTTGATCATTGTGTATGTGTGCGGA
>SRR4252611_12931760___Sbg5
TTTGATCATTGTGTATGTGTGCGGAT
>SRR4252624_1583656___Sbg5
TGGCATGTAGCCTACTCCTTTCGAAAAACA
>SRR4252612_6684734___Sbg5
ATAGGGAGATGAGTGTGTGTATAGTG
>SRR4252608_7328683___Sbg5
CACAATTTAGTGATCAGAAGTTGTACA
>SRR4252605_2633235___Sbg5
GAAGTTGTACACCACCACTCCTCCTAGTCTC
>SRR4252610_11893748___Sbg5
TGTGCGGATAGGAGGATGAATGTGTGTAT
>SRR4252605_4854826___Sbg5
TATTTGATCATTGTGTATGTGTGCGG
>SRR4252608_4545558___Sbg5
TCATTGTGTATGTGTGCGGATAGGAGA
>SRR4252605_14996574___Sbg5
TCATTGTGTATGTGTGCGGATAGGAGGATG
>SRR4252614_4503896___Sbg5
TCATTGTGTATGTGTGCGGATAGGAGGAT
>SRR4252626_7948086___Sbg5
TTAGTGATCAGAAGTTGTACACCACCACTC
>SRR4252615_15133606___Sbg5
TGGATTTAACCCAGGTGAATTTGGTGT
>SRR4252612_7663567___Sbg5
TAGTGATCAGAAGTTGTACACCACCACTC
>SRR4252614_7418418___Sbg5
TTTTGTGTGTGTGGTGTGGTATGATGCG
>SRR4252610_226765___Sbg5
GCAATTTAGTGATCAGAAGTTGTACACCA
>SRR4252606_2452978___Sbg5
TTAGTGATCAGAAGTTGTACACCACCACTC
>SRR4252611_13831223___Sbg5
TATTTGATCATTGTGTATGTGTGCGGAT
>SRR4252606_8133470___Sbg5
ATGTGTGCGGATAGGAGGATGAGTGTGT
>SRR4252609_3351509___Sbg5
ATGTGTGCGGATAGGAGGATGAGTGTG
>SRR4252611_10792847___Sbg5
ACCCATCCGACGGACGGATCACTATCAA
>SRR4252605_213596___Sbg5
TCATTGTGTATGTGTGCGGATAGGAGGA
>SRR4252606_6307616___Sbg5
TGTGTATGTGTGCGGATAGGAGGATG
>SRR4252610_7237667___Sbg5
ACCCAGGTGAAATTGGTGTACAATTTA
>SRR4252612_4357423___Sbg5
TGTATAGTGTTGTATTGTGTGTGTGGTG
>SRR4252605_14479710___Sbg5
CACAGCCGATAGCCCCGGTTTTCTAGA
>SRR4252606_3042295___Sbg5
TGTGTATGTGTGCGGATAGGAGGATG

>SRR4252605_13018385___Sbg5
TGTATGTGTGCGGATAGGAAGATGAGTGT
>SRR4252605_1327649___Sbg5
TTAGTGATCAGAAGTTGTACACCACCACTC
>SRR4252619_1487294___Sbg5
TCAGTACTACGCTGATCCCCACAGCCGA
>SRR4252619_10535155___Sbg5
CTTAACGTCCCCATCCGACGGACGGAT
>SRR4252606_8948130___Sbg5
TTAGTGATCAGAAGTTGTACACCACCATTC
>SRR4252615_10049912___Sbg5
ATTTGATCATTGTGTATGTGTGCGGATA
>SRR4252608_9254096___Sbg5
TATGAAAGAGGGAGAAGGTGAAACCTGG
>SRR4252606_1171851___Sbg5
TTAGTGATCAGAAGTTGTACACCACCATTC
>SRR4252624_10263495___Sbg5
TGAGTGTGTATAGTGTTGTATTGTGTGT
>SRR4252605_1431466___Sbg5
CCCCATCCGACGGACGGATCACTATCAA
>SRR4252605_262985___Sbg5
CGGACGGATCACTATCAGCAATGACA
>SRR4252610_8740145___Sbg5
TTAGTGATCAGAAGTTGTACACCACCACTC
>SRR4252622_7126869___Sbg5
CCATCCGACGGACGGATCACTATCAA
>SRR4252613_7760250___Sbg5
AGTACTACGCTGGTCCCCACAGCCGATAA
>SRR4252616_1136698___Sbg5
TACCGAGCGGCTGCCGCCATAGGGCGGAA
>SRR4252614_8855093___Sbg5
CGGATAGGAGGATGAGTGTGTGTATGGTGTT
>SRR4252606_2145173___Sbg5
TATTTGATCATTGTGTATGTGTGCGGAT
>SRR4252626_5843597___Sbg5
TTAGTGATCAGAAGTTGTACGCCACCACTC
>SRR4252609_11495014___Sbg5
AAGGAGAAGGTGAAACCCGGTGCCGGCAT
>SRR4252605_6505262___Sbg5
TCCTTTCGAAAAGCACCAAGGGAACCGGC
>SRR4252605_381647___Sbg5
CACCACCACTCCTCCTAGTCTCAGGATTCGA
>SRR4252609_4441320___Sbg5
GGTATGATGTGAGTGACGATTATGGA
>SRR4252625_13523348___Sbg5
TCACTATCAACAATGACATATGCCTTCT
>SRR4252606_5467108___Sbg5
GGTATGACGTGAGTGACGATTATGGATA
>SRR4252605_1737444___Sbg5
TGATGTGAGTGACGAAAGTGGATATGAAAG
>SRR4252610_2439924___Sbg5
GGCCGATAACCCCGGTTTTCTAGACCA
>SRR4252605_7821062___Sbg5
GTGTGGTATGATGTGAGTGACGATTAT
>SRR4252610_6228416___Sbg5
ATTGGTGTGTAAGTGTGTGAATAGGGG
>SRR4252612_977876___Sbg5
TATGAAAGAGGGAGAAGGTGAAACCTGG
>SRR4252606_6856421___Sbg5
TTAGTGATCAGAAGTTGTACACCACCACTC
>SRR4252623_10976424___Sbg5
TTAGTGATCAGAAGTTGTACACCACCACTC
>SRR4252621_8892247___Sbg5
TAGTGATCAGAAGTTGTACACCACCACTCC
>SRR4252619_6760268___Sbg5
CCCACAGCAGATAACCCCGGTTTTCTA
>SRR4252618_5193027___Sbg5
TCATTGTGTATGTGTGCGGATAGGAGG
>SRR4252608_7711145___Sbg5
TGTGCGGATAGGAGGATGAGTGTGTGTA

>SRR4252605_9228992___Sbg5
GGTGTGGTATGATGTGAGTGACGATTATGGA
>SRR4252606_6930165___Sbg5
TTAGTGATCAGAAGTTGTACACCACCACTC
>SRR4252606_1066731___Sbg5
AGTGTGGTATGATGTGAGTGACGATTATG
>SRR4252605_12211423___Sbg5
TGATGTGAGTGACGATTATGGATATGAA
>SRR4252607_13228239___Sbg5
GTCCCCATCCGACGGACGGATCACTATCAA
>SRR4252611_793949___Sbg5
GTCAGTACTATGCTGGTCCCCACAGCCGA
>SRR4252623_6669951___Sbg5
CCTTTCGAAAAGCACCAAGGGGACCGGC
>SRR4252626_9393069___Sbg5
TAGTGATCAGAAGTTGTACACCACCACTC
>SRR4252613_4034438___Sbg5
CCCCACAGCCGATAACCCCGGTTTTCTA
>SRR4252606_5111521___Sbg5
CATTGTGTATGTGTGCGGATAGGAGGAT
>SRR4252605_1970251___Sbg5
TTATGGATATGAAAGAGGGAGAAGATG
>SRR4252608_12065325___Sbg5
ATGCCTTCTCTACATAGTCATTGCGGA
>SRR4252605_9871006___Sbg5
TTAGTGATCAGAAGTTGTACACCACCACTC
>SRR4252606_2431495___Sbg5
TAGTGATCAGAAGTTGTACACCACCACTC
>SRR4252606_1115356___Sbg5
TATTTGATCATTGTGTATGTGTGCGGT
>SRR4252610_13006463___Sbg5
TATTTGATCATTGTGTATGTGTGCGGT
>SRR4252610_1893328___Sbg5
CCAAGGCTTACCGAGCGGCTGCCGCCAT
>SRR4252614_1625503___Sbg5
AGAAGGTGAAACCCGGTGCTGGCATGT
>SRR4252610_6527867___Sbg5
TATGATGTGAGTGACGATTATGGATATG
>SRR4252610_4847777___Sbg5
GTCCCCATTCGACGGACGGATCACTATCAA
>SRR4252605_6849566___Sbg5
ATGTGAGTGACGATTATGGATATGAA
>SRR4252612_6724158___Sbg5
TGGATATGAAAGAGGGAGACGGTGAAACC
>SRR4252614_2681810___Sbg5
TGGATATGAAAGAGGGAGACGGTGAAACC
>SRR4252605_4102486___Sbg5
TCCTTTCGAAAAGCACCAAGGGGACCGCCC
>SRR4252606_2136889___Sbg5
TAGTGATCAGAAGTTGTACACCACCACTCC
>SRR4252612_1998296___Sbg5
TAGTGATCAGAAGTTGTACACCACCACTC
>SRR4252610_13507571___Sbg5
TATGTGTGCGGATAGGAGGATGAGTGT
>SRR4252606_3618025___Sbg5
GTTGTATTGTGTGTGTGGTGTGTTATG
>SRR4252621_10375880___Sbg5
CAGTATTACGCTGGTCCCCACAGCCGATAA
>SRR4252610_649039___Sbg5
GCCCACAGCCGATAACCCCGGTTTTCTA
>SRR4252610_7392937___Sbg5
TTTGATCATTGTGTATGTGTGCGGATAG
>SRR4252612_6863135___Sbg5
TATGAAAGAGGGAGAAGGTGAAACCTGG
>SRR4252619_3005820___Sbg5
CGATAACCCCGGTTTTCTAGACCAGCTGA
>SRR4252605_11754665___Sbg5
TCACTATCAACAATGACGTATGCCTTCTC
>SRR4252611_750740___Sbg5
TACCGAGCGGCTGCCGCCATAGGGTGGAA

>SRR4252612_2224529___Sbg5
TTAGTGATCAGAAGTTGTACACCACCACTC
>SRR4252611_14192508___Sbg5
TGATGTGAGTGACGGTTATGGATATG
>SRR4252608_9538936___Sbg5
AGTATGATGTGAGTGACGATTATGGA
>SRR4252612_3184300___Sbg5
AAAATTTAGTGATCAGAAGTTGTACACC
>SRR4252610_6053237___Sbg5
GCAATTTAGTGATCAGAAGTTGTACAC
>SRR4252622_5780802___Sbg5
TGATGTGAGTGACGATTATGGATATGA
>SRR4252608_8746429___Sbg5
GCTTAACGTCCCCATCCGACGGACGGA
>SRR4252610_9750903___Sbg5
TTAGTGATCAGAAGTTGTACACCACCACTC
>SRR4252611_8529118___Sbg5
TCAACAATGACATATGCCTTCTCTACATA
>SRR4252613_4534571___Sbg5
TTTAGTGATCAGAAGTTGTACACCATC
>SRR4252606_661581___Sbg5
GTCATTGCGGAGGAGTTCGGGATTTAA
>SRR4252605_9237171___Sbg5
CTTAACGTCCCCATCCGACGGACGGATCA
>SRR4252606_7874780___Sbg5
TAGGACGGAATTAACGGGGGGTAGTATGG
>SRR4252612_5790482___Sbg5
TTAGTGATCAGAAGTTGTACACCACCACTC
>SRR4252605_7113055___Sbg5
TAGTGATCAGAAGTTGTACACCACCACTC
>SRR4252610_9290516___Sbg5
TATTTGATCATTGTGTATGTGTGCGG
>SRR4252612_5370985___Sbg5
TCATTGTGTATGTGTGCGGATAGGAGA
>SRR4252620_5443853___Sbg5
TCATTGTGTATGTGTGCGGATAGGAGA
>SRR4252606_2017309___Sbg5
GACGGCTTAATGTCCCCATCCGACGGACG
>SRR4252611_11736455___Sbg5
TGTCCCCATCCGACGGACGGATCACTATCAA
>SRR4252623_12994005___Sbg5
AGTCCCCACAGCCGATAACCCCGGTTTTCT
>SRR4252613_1707432___Sbg5
CAAATTTAATGATCAGAAGTTGTACACCA
>SRR4252618_7267586___Sbg5
AAATTTAATGATCAGAAGTTGTACACCAC
>SRR4252621_6218157___Sbg5
TTAATGATCAGAAGTTGTACACCACCACTC
>SRR4252607_8463145___Sbg5
TCCCCATCCGACGGACGGATCACTATCAA
>SRR4252608_2126472___Sbg5
TCCTTTCGAAAAGCACCAAGGGGACCGGT
>SRR4252608_5807157___Sbg5
TATGTGTGCGGATAGGAGGATGACTGTGT
>SRR4252608_10636808___Sbg5
ATGTGAGTGACGGTTATGGATATGAA
>SRR4252610_796330___Sbg5
TAGGACGGAATTAACGGGGGGTAGTATGC
>SRR4252613_4574049___Sbg5
CAGTACTACGCTGGTCCCCACAGCCGA
>SRR4252606_9642900___Sbg5
ATTTAGTGATCAGAAGTTGTACACCACT
>SRR4252612_8786944___Sbg5
GATCATTGTGTATGTGTGCGGATAGGA
>SRR4252606_3509761___Sbg5
TCATTGTGTATGTGTGCGGATAGGAGGA
>SRR4252616_10298523___Sbg5
CCAGCTATTTGATCATTGTGTATGTGT
>SRR4252610_11478693___Sbg5
TCATTGTGTATGTGTGCGGATAGGAGG

```
>SRR4252608_130256___Sbg5
GCTTAACGTCCCCATCCGACGGACGGA
>SRR4252605_5799221___Sbg5
TTAGTGATCAGAAGTTGTACACCACCACTT
>SRR4252610_9233343___Sbg5
TAGTGATCAGAAGTTGTACACCACCACTT
>SRR4252606_9517244___Sbg5
TAGGACGGAATTAACGGGGGGTAGTATG
>SRR4252612_4371975___Sbg5
TAGTGATCAGAAGTTGTACACCACCACTCC
>SRR4252608_7325005___Sbg5
TTTGATCATTGTGTATGTGTGCGGATA
>SRR4252614_3733305___Sbg5
TCATTGTGTATGTGTGCGGATAGGAGGA
>SRR4252619_6363562___Sbg5
TTAGTGATCAGAAGTTGTACACCACCACTT
>SRR4252611_12876646___Sbg5
TATTTGATCATTGTGTATGTGTGCGGAT
>SRR4252615_9849871___Sbg5
ACAATTTAGTGATCAGAAGTTGTACA
>SRR4252611_12836909___Sbg5
TACCGAGCGGCTGCCGCCATAGGGCGGAA
>SRR4252611_2285428___Sbg5
ATCCCCATCCGACGGACGGATCACTATCAA
>SRR4252611_1496031___Sbg5
TATGAAAGAGGGAGAAGGTGAAACCCGA
>SRR4252611_7131827___Sbg5
TTAGTGATCAGAAGTTGTACACCACCACTC
>SRR4252605_13318452___Sbg5
AACATAGTCATTGCGGAGGAATTCGGGA
>SRR4252605_8196265___Sbg5
TTTAGTGATCAGAAGTTGTACACCACCATT
>SRR4252619_10426022___Sbg5
TTAGTGATCAGAAGTTGTACACCACCAT
>SRR4252622_1009710___Sbg5
CACTATCAACAATGACATATGCCTTCT
>SRR4252624_1716513___Sbg5
TCACTATCAACAATGACATATGCCTG
>SRR4252614_6600458___Sbg5
TCTCCACAGCCGATAACCCCGGTTTTCTA
>SRR4252606_3689668___Sbg5
TTAGTGATCAGAAGTTGTACACCACCACTC
>SRR4252610_7802415___Sbg5
TATTTGATCATTGTGTATGTGTGCGGATA
>SRR4252611_2874339___Sbg5
TATTTGATCATTGTGTATGTGTGCGGAT
>SRR4252609_12486480___Sbg5
TGTGTGCGGATAGGAGGATGAGTGTG
>SRR4252616_13789425___Sbg5
TCACTATCAACAATGACATATGCCTTCT
>SRR4252610_4779724___Sbg5
TATTTGATCATTGTGTATGTGTGCGGAT
>SRR4252625_13179086___Sbg5
TTAGTGATCAGAAGTTGTACACCACCACTC
>SRR4252610_5723275___Sbg5
TCACAATTTAGTGATCAGAAGTTGTACA
>SRR4252616_11848174___Sbg5
AACGTCCCCATCCGACGGACGGATCACTA
>SRR4252612_700857___Sbg5
TTAGTGATCAGAAGTTGTACACCACCACTC
>SRR4252618_2830469___Sbg5
TTAGTGATCAGAAGTTGTACACCACCACTCC
>SRR4252609_2294773___Sbg5
GTGAGTGACGATTATGGATATGAAAGAG
>SRR4252622_1354917___Sbg5
TCCTTTCGAAAAGCACCAAGGGGACCGA
>SRR4252608_9992630___Sbg5
TTAGTGATCAGAAGTTGTACACCACCACTC
>SRR4252610_6577507___Sbg5
TAGTGATCAGAAGTTGTACACCACCACTCC
```

>SRR4252625_1842628___Sbg5
TCACTATCAACAATGACATGTGCCTTCTC
>SRR4252606_4387183___Sbg5
AAGTTGCACACCACCACTCCTCCTAGT
>SRR4252611_7326105___Sbg5
TGTGCGGATAGGAGGATGAGTGTGTGTAT
>SRR4252611_724745___Sbg5
TCATTGTGTATGTGTGCGGATAGGAG
>SRR4252610_5578957___Sbg5
GATGTGAGTGACGATTATGGATATGAAA
>SRR4252616_3966608___Sbg5
TACAATTTAGTGATCAGAAGTTGTACAC
>SRR4252606_1829919___Sbg5
TGGCTTAACGTCCCCATCCGACGGACGGA
>SRR4252612_4578714___Sbg5
TTAGTGATCAGAAGTTGTACACCACCACTC
>SRR4252612_1428721___Sbg5
TTGTATTGTGTGTGTGGTGTGGTATGATC
>SRR4252607_6550646___Sbg5
ACCCCATCCGACGGACGGATCACTATCAA
>SRR4252611_7294992___Sbg5
TTAGTGATCAGAAGTTGTACACCACCACTT
>SRR4252612_4922125___Sbg5
TTAGTGATCAGAAGTTGTACACCACCACTT
>SRR4252626_6321963___Sbg5
TTAGTGATCAGAAGTTGTACACCACCACTT
>SRR4252612_7632363___Sbg5
TATTTGATCATTGTGTATGTGTGCGGAT
>SRR4252612_3616068___Sbg5
TTTGATCATTGTGTATGTGTGCGGATA
>SRR4252612_2802685___Sbg5
ATCATTGTGTATGTGTGCGGATAGGAG
>SRR4252605_7101134___Sbg5
TGATGTGAGTGACGATTATGGATATGA
>SRR4252611_13631976___Sbg5
ACGGACGGATCACTATCAACAATGACAT
>SRR4252619_1274820___Sbg5
TACCGAGCGGCTGCCGCCATAGGACGGA
>SRR4252606_4865609___Sbg5
TAGTGATCAGAAGTTGTACACCACCACTCC
>SRR4252611_2077230___Sbg5
TCATTGTGTATGTGTGCGGATAGGAGGA
>SRR4252622_2052699___Sbg5
TAGTGTTGTATTGTGTGTGTGGTGTGG
>SRR4252616_434796___Sbg5
ATTTGATCATTGTGTATGTGTGCGGATA
>SRR4252616_7792194___Sbg5
TGAAACCCGGTGCCGGCATGTAGCCTAT
>SRR4252612_545168___Sbg5
CAGTTAATGGATCGGTGTGTAAGTGTG
>SRR4252610_5817892___Sbg5
ACAATTTAGTGATCAGAAGTTGTACA
>SRR4252611_5709880___Sbg5
TAGGACGGAATTAACGGGGGGTAGTGTGG
>SRR4252606_7772234___Sbg5
GTGTGTGAATAGGGGGATGAGTGTGTGTATA
>SRR4252608_1468507___Sbg5
CGATTATGGATATGAAAGAGGGAGAAAGTA
>SRR4252612_3645454___Sbg5
TATGGATATGAAAGAGGGAGAAAGTA
>SRR4252605_2982063___Sbg5
GTATGATGTGAGTGACGATTATGGATATGA
>SRR4252621_2850192___Sbg5
TGTAAGTGTGTGAATAGGGAGATGAGTGT
>SRR4252608_3195020___Sbg5
TGTGTGGATAGGAGGATGAGTGTGTGTA
>SRR4252608_12840455___Sbg5
TATGTAGTCTACTCCTTTCGAAAAGCA
>SRR4252610_2392022___Sbg5
TAGTGATCAGAAGTTGTACACCACCACTC

>SRR4252614_6241713___Sbg5
CAGTTAATGGATCGGTGTGTAAGTGTGTG
>SRR4252609_694870___Sbg5
TGAATTTGGTGTACAATTTAGTGATC
>SRR4252605_5337031___Sbg5
TATTTGATCATTGTGTATGTGTGCGGAT
>SRR4252619_3538391___Sbg5
TATTTGATCATTGTGTATGTGTGCGGA
>SRR4252610_9357307___Sbg5
TGTAAGTGTGTGGATAGGGGGATGACTG
>SRR4252612_7105058___Sbg5
AGCTATTTGATCATTGTGTATGTGTGC
>SRR4252610_2145490___Sbg5
TATTTGATCATTGTGTATGTGTGCGGAT
>SRR4252621_2459301___Sbg5
TATTTGATCATTGTGTATGTGTGCGGAT
>SRR4252610_8182972___Sbg5
AGCTAATGGATTGGTGTGTAAGTGTGTGG
>SRR4252618_5250571___Sbg5
AGCTAATGGATTGGTGTGTAAGTGTGTG
>SRR4252625_6096576___Sbg5
TGTTTTGTGTGTGTGGTGTGGTATGATGTG
>SRR4252620_10994377___Sbg5
TATGATGTGAGTGACGATTATGGATATGA
>SRR4252611_8185936___Sbg5
TGGATATGAAAGAGGGAGAAGGTGAAACCT
>SRR4252605_5152346___Sbg5
GATGTGAGTGACGATTATGGGTATGAA
>SRR4252606_8272586___Sbg5
GTGATGTGAGTGACGATTATGGATATGAA
>SRR4252613_7748727___Sbg5
GAAACCCGGTGCCGGCATGTAGCCTACTC
>SRR4252606_6068076___Sbg5
TTAGTGATCAGAAGTTGTACACCACCTC
>SRR4252610_11892790___Sbg5
TTAGTGATCAGAAGTTGTACACCACCTCC
>SRR4252612_529750___Sbg5
TTAGTGATCAGAAGTTGTACACCACCTC
>SRR4252612_3866161___Sbg5
TGAAACCCGGTGCCGGCATGTAGCCTAT
>SRR4252612_6487818___Sbg5
TACTACGCTGATCCCCACAGCCGATA
>SRR4252606_5296456___Sbg5
TTAGTGATCAGAAGTTGTACACCACCACTT
>SRR4252622_7936901___Sbg5
TTAGTGATCAGAAGTTGTACACCACCACTT
>SRR4252608_8306767___Sbg5
TAGTGATCAGAAGTTGTACACCACCACTT
>SRR4252619_6484574___Sbg5
CAGGATTCGAAATTTTAATTAATTACTC
>SRR4252611_7629862___Sbg5
TGGATCGGTGTGTAAGTGTGTGAATAGG
>SRR4252605_6097217___Sbg5
TTAGTGATCAGAAGTTGTACACCACCACTTA
>SRR4252606_6233799___Sbg5
TTAGTGATCAGAAGTTGTACACCACCACTT
>SRR4252608_489354___Sbg5
TTAGTGATCAGAAGTTGTACACCACCACTT
>SRR4252611_15247040___Sbg5
TTAGTGATCAGAAGTTGTACACCACCACTT
>SRR4252611_4641901___Sbg5
TTAGTGATCAGAAGTTGTACACCACCACTT
>SRR4252614_3267224___Sbg5
ATCCCCATCCGACGGACGGATCACTATCA
>SRR4252612_6583828___Sbg5
TTAGTGATCAGAAGTTGTACAGCACCACTC
>SRR4252624_5520406___Sbg5
TGAGTGTGTATAGTGTTGTATTGTGTGT
>SRR4252607_2233819___Sbg5
ATCATTGCGGAGGAGTTCGGGATTTAAC

```
>SRR4252610_2909541___Sbg5
TCATTGTGTATGTGTGCGGATAGGAGG
>SRR4252611_8880312___Sbg5
TGAAAGAGGGAGAAGGTGAAACCCGGTGC
>SRR4252608_9804406___Sbg5
ACGGACGGATCACTATCAACAATGACATA
>SRR4252606_544800___Sbg5
TATTTGATCATTGTGTATGTGTGCGGAT
>SRR4252612_6657179___Sbg5
TGTATGTGTGCGGATAGGAGGATGAGTGC
>SRR4252608_5419128___Sbg5
TATGATGTGAGTGACGATTATGGACATG
>SRR4252622_7794519___Sbg5
TTAGTGATCAGAAGTTGTACACCTCCACT
>SRR4252619_9487590___Sbg5
TATAGCTCAACAGCCCGATGTGGGCCA
>SRR4252610_7843736___Sbg5
GTGTTGTATTGTGTGTGTGGTGTAAT
>SRR4252606_3512020___Sbg5
TCATTGTGTATGTGTGCGGATAGGAGGA
>SRR4252622_8974767___Sbg5
TATAGTGTTGTATTGTGTGTGTTGTGTGG
>SRR4252610_810149___Sbg5
AAAATTTAGTGATCAGAAGTTGTACA
>SRR4252608_8519760___Sbg5
TTTGATCATTGTGTATGTGTGCGGATAG
>SRR4252608_12583098___Sbg5
TCATTGTGTATGTGTGCGGATAGGAGGA
>SRR4252610_6904655___Sbg5
TGTGTATGTGTGCGGATAGGAGGATGAG
>SRR4252619_2097348___Sbg5
TAGGACGGAATTAACGGGGGGTAGTATGA
>SRR4252610_4800371___Sbg5
GATGTGAGTGACGATTATGGATATGAA
>SRR4252621_8932286___Sbg5
TATGATGTGAGTGACGATTATGGATATGA
>SRR4252610_8552667___Sbg5
ACAATTTAGTGATCAGAAGTTGTACA
>SRR4252610_1057200___Sbg5
TATTTGATCATTGTGTATGTGTGCGGAC
>SRR4252610_8202824___Sbg5
TATTTGATCATTGTGTATGTGTGCGGAC
>SRR4252606_1318628___Sbg5
TTAGTGATCAGAAGTTGTACACCACCACTC
>SRR4252612_1848689___Sbg5
TTAGTGATCAGAAGTTGTACACCACCACTC
>SRR4252626_4585527___Sbg5
TTAGTGATCAGAAGTTGTACACCACCACTC
>SRR4252611_769796___Sbg5
TTAGTGATCAGAAGTTGTACACCACCACTC
>SRR4252610_3469279___Sbg5
TTAGTGATCAGAAGTTGTACACCACCACTC
>SRR4252614_8505544___Sbg5
TTAGTGATCAGAAGTTGTACACCACCACTC
>SRR4252613_10087192___Sbg5
TAACGTCCCCATCCGACGGACGGATC
>SRR4252611_3627113___Sbg5
TTTAGTGATCAGAAGTTGTACACCACCACTC
>SRR4252612_5580690___Sbg5
TAATTACTCAATTAAAATTCCCAACC
>SRR4252610_2284893___Sbg5
TTAGTGATCAGAAGTTGTACACCACCACTC
>SRR4252610_7215431___Sbg5
TATGATGTGAGTGACGATTATGGATATT
>SRR4252610_9200391___Sbg5
TATGATGTGAGTGACGATTATGGATATT
>SRR4252615_9310174___Sbg5
TCCTTTCGAAAAGCACCAAGGGGACCGC
>SRR4252606_369698___Sbg5
TCACTATCAACAATGACGTATGCCTTCT
```

>SRR4252606_7235947___Sbg5
TTAGTGATCAGAAGTTGTACACCAACACTC
>SRR4252609_10839095___Sbg5
TTAGTGATCAGAAGTTGTACACCAACACTC
>SRR4252611_11932989___Sbg5
TTAGTGATCAGAAGTTGTACACCAACACTC
>SRR4252612_7036411___Sbg5
TTAGTGATCAGAAGTTGTACACCAACACTC
>SRR4252620_5155713___Sbg5
TTAGTGATCAGAAGTTGTACACCAACACTC
>SRR4252612_3129829___Sbg5
TAGTGATCAGAAGTTGTACACCAACAC
>SRR4252619_7647025___Sbg5
TAGTGATCAGAAGTTGTACACCAACACTC
>SRR4252611_6062604___Sbg5
CATTGCAGAGGAGTTCGGGATTTAACCCA
>SRR4252619_6144382___Sbg5
CCCCACAGCCGATAACCCCGGTTTTCTA
>SRR4252611_15066934___Sbg5
TATTTGATCATTGTGTATGTGTGCGG
>SRR4252606_2172563___Sbg5
TCATTGTGTATGTGTGCGGATAGAAGGA
>SRR4252609_9068___Sbg5
GTCCCCATCCGACGGACGGATCACTATCAA
>SRR4252612_922489___Sbg5
TCATTGTGTATGTGTGCGGATAGGAGG
>SRR4252612_8338066___Sbg5
GAACGTCCCCATCCGACGGACGGATCACT
>SRR4252605_9227652___Sbg5
CATCCGACGGACGGATCACTATCAACA
>SRR4252611_13769411___Sbg5
TTAGTGATCAGAAGTTGTACACCACCACTC
>SRR4252611_1132171___Sbg5
TTAGTGATCAGAAGTTGTACACCACCACT
>SRR4252612_3081873___Sbg5
TGGTATGATGTGAGTGACGAAAGTGAAT
>SRR4252606_7713382___Sbg5
TATGAAAGAGGGAGAAGGTGAAACCTGG
>SRR4252611_10164620___Sbg5
AATACTACGCTGATCCCCACAGCCGATAA
>SRR4252608_11155296___Sbg5
TAGGACGGAATTAACGGGGGGCAGTATGG
>SRR4252608_7276659___Sbg5
GATGTGAGTGACGATTATGGATATGAC
>SRR4252620_2487892___Sbg5
AAGGCTTACCGAGCGGCTGCCGCCATAA
>SRR4252610_7648518___Sbg5
CAATTTAGAGATCAGAAGTTGTACACCA
>SRR4252610_10632856___Sbg5
ATGTGAGTGACGATTATGGATATGAAAGA
>SRR4252605_11083108___Sbg5
GTGAGTGACGATTATGGATATGAAAG
>SRR4252611_2815101___Sbg5
CAGGATTCGAAATTTTAATTAATTACT
>SRR4252605_5661018___Sbg5
TATGAAAGAGGGAGAAGGTGAAACCTGTT
>SRR4252613_1041155___Sbg5
CCCACAGCCGATAACCCCGGTTTTCTA
>SRR4252611_8826909___Sbg5
CATGTAGCCTACTCCTTTCGAAAAACA
>SRR4252616_15537894___Sbg5
TCCGACGGACGGATCACTATCAACAATGACA
>SRR4252605_4836705___Sbg5
TTAGTGATCAGAAGTTGTACACCACCACTT
>SRR4252610_13299754___Sbg5
TTAGTGATCAGAAGTTGTACACCACCACTT
>SRR4252612_2116726___Sbg5
ACAATTTAGTGATCAGAAGTTGTACACCA
>SRR4252619_10914112___Sbg5
CCCCACAGCCGATAACCCCGGTTTTCTA

>SRR4252611_3629384___Sbg5
AGTTAATGGATCGGTGTGTAAGTGTGTGAA
>SRR4252610_5478449___Sbg5
TAATGGATCGGTGTGTAAGTGTGTGA
>SRR4252611_6380434___Sbg5
TGAAACCCGGTGCCGGCATGTAGCCTAC
>SRR4252619_4736614___Sbg5
TAGTGATCAGAAGTTGTACACCACCA
>SRR4252619_14314986___Sbg5
ACAATTTAGTGATCAGAAGTTGTACACCA
>SRR4252612_1183211___Sbg5
TTAGTGATCAGAAGTTGTACACCACCACTC
>SRR4252619_10379325___Sbg5
TGGATATGAAAGAGGGAGAAGGTGAC
>SRR4252611_5358987___Sbg5
TCCTTTCGAAAAGCACCAAGGGGACCGC
>SRR4252612_7596901___Sbg5
ATGATGTGAGTGACGATTATGGATAT
>SRR4252624_8706941___Sbg5
TCCTTTCGAAAAGCACCAAGGGGACCGCCT
>SRR4252605_9381634___Sbg5
TATGAAAGAGGGAGAAGGTGAAACCTGGT
>SRR4252610_11665282___Sbg5
TATGAAAGAGGGAGAAGGTGAAACCTGG
>SRR4252607_1394365___Sbg5
ACGATGTGAGTGACGATTATGGATATGAA
>SRR4252610_13491382___Sbg5
TATTTGATCATTGTGTATGTGTGCGGA
>SRR4252610_7079888___Sbg5
TATTTGATCATTGTGTATGTGTGCGGAT
>SRR4252608_972873___Sbg5
TGTGCGGATAGGAGGATGAGTGTGTGTG
>SRR4252605_10877160___Sbg5
TTAGTGATCAGAAGTTGTACACCACCACTT
>SRR4252612_1573026___Sbg5
TAGTGATCAGAAGTTGTACACCACCACTT
>SRR4252605_8226225___Sbg5
TTAGTGATCAGAAGTTGTACACCACCATTC
>SRR4252619_1825083___Sbg5
CAGTACCACGCTGGTCCCCACAGCCGATA
>SRR4252611_7227920___Sbg5
GTACCACGCTGGTCCCCACAGCCGATAA
>SRR4252615_4383690___Sbg5
CCCCACAGCCGATAACCCCGGTTTTCTA
>SRR4252611_8316566___Sbg5
TATGAAAGAGGGAGAAGGTGAAACCGG
>SRR4252605_21940___Sbg5
TCTGATCTGATCTATAGCTCAACAGCCCT
>SRR4252605_2853138___Sbg5
TCTGATCTGATCTATAGCTCAACAGCCCT
>SRR4252605_4760821___Sbg5
TCTGATCTGATCTATAGCTCAACAGCCCTA
>SRR4252605_6598955___Sbg5
TCTGATCTGATCTATAGCTCAACAGCCCT
>SRR4252606_4716132___Sbg5
TCTGATCTGATCTATAGCTCAACAGCCCT
>SRR4252606_955832___Sbg5
TCTGATCTGATCTATAGCTCAACAGCCC
>SRR4252610_6889463___Sbg5
TCTGATCTGATCTATAGCTCAACAGCCC
>SRR4252610_7473352___Sbg5
TCTGATCTGATCTATAGCTCAACAGCCCT
>SRR4252610_8188892___Sbg5
TCTGATCTGATCTATAGCTCAACAGCCCT
>SRR4252611_10314488___Sbg5
TCTGATCTGATCTATAGCTCAACAGCCCT
>SRR4252611_11476511___Sbg5
TCTGATCTGATCTATAGCTCAACAGCCCTA
>SRR4252611_12392957___Sbg5
TCTGATCTGATCTATAGCTCAACAGCCCTA

>SRR4252611_12446530___Sbg5
TCTGATCTGATCTATAGCTCAACAGCCCTA
>SRR4252611_12727584___Sbg5
TCTGATCTGATCTATAGCTCAACAGCCCTA
>SRR4252611_1409806___Sbg5
TCTGATCTGATCTATAGCTCAACAGCCCT
>SRR4252611_14455225___Sbg5
TCTGATCTGATCTATAGCTCAACAGCCCT
>SRR4252611_1893456___Sbg5
TCTGATCTGATCTATAGCTCAACAGCCCT
>SRR4252611_1952534___Sbg5
TCTGATCTGATCTATAGCTCAACAGCCCT
>SRR4252611_2920281___Sbg5
TCTGATCTGATCTATAGCTCAACAGCCCT
>SRR4252611_3376542___Sbg5
TCTGATCTGATCTATAGCTCAACAGCCCT
>SRR4252611_392865___Sbg5
TCTGATCTGATCTATAGCTCAACAGCCCT
>SRR4252611_4034755___Sbg5
TCTGATCTGATCTATAGCTCAACAGCCCT
>SRR4252611_4424275___Sbg5
TCTGATCTGATCTATAGCTCAACAGCCCT
>SRR4252611_4867634___Sbg5
TCTGATCTGATCTATAGCTCAACAGCCCTA
>SRR4252611_5318312___Sbg5
TCTGATCTGATCTATAGCTCAACAGCCCT
>SRR4252611_5355002___Sbg5
TCTGATCTGATCTATAGCTCAACAGCCCTA
>SRR4252611_6063642___Sbg5
TCTGATCTGATCTATAGCTCAACAGCCCT
>SRR4252611_6199395___Sbg5
TCTGATCTGATCTATAGCTCAACAGCCCT
>SRR4252611_7054127___Sbg5
TCTGATCTGATCTATAGCTCAACAGCCCT
>SRR4252611_7227850___Sbg5
TCTGATCTGATCTATAGCTCAACAGCCCT
>SRR4252611_8044354___Sbg5
TCTGATCTGATCTATAGCTCAACAGCCCT
>SRR4252611_8474346___Sbg5
TCTGATCTGATCTATAGCTCAACAGCCCTA
>SRR4252611_861668___Sbg5
TCTGATCTGATCTATAGCTCAACAGCCCT
>SRR4252611_9450901___Sbg5
TCTGATCTGATCTATAGCTCAACAGCCCT
>SRR4252611_9987590___Sbg5
TCTGATCTGATCTATAGCTCAACAGCCCT
>SRR4252612_6862376___Sbg5
TCTGATCTGATCTATAGCTCAACAGCCCT
>SRR4252613_1729124___Sbg5
TCTGATCTGATCTATAGCTCAACAGCC
>SRR4252613_2289327___Sbg5
TCTGATCTGATCTATAGCTCAACAGCCCT
>SRR4252613_6324545___Sbg5
TCTGATCTGATCTATAGCTCAACAGCCCTA
>SRR4252613_7771179___Sbg5
TCTGATCTGATCTATAGCTCAACAGCCCT
>SRR4252613_8176459___Sbg5
TCTGATCTGATCTATAGCTCAACAGCCCT
>SRR4252613_9273204___Sbg5
TCTGATCTGATCTATAGCTCAACAGCCCT
>SRR4252614_8451988___Sbg5
TCTGATCTGATCTATAGCTCAACAGCCCTA
>SRR4252615_10986102___Sbg5
TCTGATCTGATCTATAGCTCAACAGCCCT
>SRR4252615_11071929___Sbg5
TCTGATCTGATCTATAGCTCAACAGCCCT
>SRR4252615_12039051___Sbg5
TCTGATCTGATCTATAGCTCAACAGCCCT
>SRR4252615_13135626___Sbg5
TCTGATCTGATCTATAGCTCAACAGCCCT

>SRR4252615_13288522___Sbg5
TCTGATCTGATCTATAGCTCAACAGCCCT
>SRR4252615_13624729___Sbg5
TCTGATCTGATCTATAGCTCAACAGCCCT
>SRR4252615_14702002___Sbg5
TCTGATCTGATCTATAGCTCAACAGCCCT
>SRR4252615_15122913___Sbg5
TCTGATCTGATCTATAGCTCAACAGCCCT
>SRR4252615_2938363___Sbg5
TCTGATCTGATCTATAGCTCAACAGCCCT
>SRR4252615_5474250___Sbg5
TCTGATCTGATCTATAGCTCAACAGCCCT
>SRR4252615_6148045___Sbg5
TCTGATCTGATCTATAGCTCAACAGCCCT
>SRR4252615_8283258___Sbg5
TCTGATCTGATCTATAGCTCAACAGCCCT
>SRR4252615_8868898___Sbg5
TCTGATCTGATCTATAGCTCAACAGCCCT
>SRR4252615_9750932___Sbg5
TCTGATCTGATCTATAGCTCAACAGCCCT
>SRR4252616_12355280___Sbg5
TCTGATCTGATCTATAGCTCAACAGCCCT
>SRR4252616_12864284___Sbg5
TCTGATCTGATCTATAGCTCAACAGC
>SRR4252616_9038946___Sbg5
TCTGATCTGATCTATAGCTCAACAGCCCTA
>SRR4252617_10055699___Sbg5
TCTGATCTGATCTATAGCTCAACAGCCCT
>SRR4252617_1238380___Sbg5
TCTGATCTGATCTATAGCTCAACAGCCCT
>SRR4252617_14178318___Sbg5
TCTGATCTGATCTATAGCTCAACAGCCCT
>SRR4252617_15412154___Sbg5
TCTGATCTGATCTATAGCTCAACAGCCCT
>SRR4252617_3926564___Sbg5
TCTGATCTGATCTATAGCTCAACAGCCCT
>SRR4252617_4583080___Sbg5
TCTGATCTGATCTATAGCTCAACAGCCCT
>SRR4252617_6955110___Sbg5
TCTGATCTGATCTATAGCTCAACAGCCCT
>SRR4252617_7915881___Sbg5
TCTGATCTGATCTATAGCTCAACAGCCCT
>SRR4252618_1752946___Sbg5
TCTGATCTGATCTATAGCTCAACAGCCCT
>SRR4252618_2454712___Sbg5
TCTGATCTGATCTATAGCTCAACAGCCC
>SRR4252618_4575118___Sbg5
TCTGATCTGATCTATAGCTCAACAGCCCT
>SRR4252618_5376422___Sbg5
TCTGATCTGATCTATAGCTCAACAGCCCT
>SRR4252618_5869231___Sbg5
TCTGATCTGATCTATAGCTCAACAGCCCT
>SRR4252618_5923739___Sbg5
TCTGATCTGATCTATAGCTCAACAGCCCT
>SRR4252618_6648938___Sbg5
TCTGATCTGATCTATAGCTCAACAGCCCT
>SRR4252618_9126030___Sbg5
TCTGATCTGATCTATAGCTCAACAGCCCT
>SRR4252618_948789___Sbg5
TCTGATCTGATCTATAGCTCAACAGCCCT
>SRR4252619_10617925___Sbg5
TCTGATCTGATCTATAGCTCAACAGCCCT
>SRR4252619_12804473___Sbg5
TCTGATCTGATCTATAGCTCAACAGCCCT
>SRR4252619_15267748___Sbg5
TCTGATCTGATCTATAGCTCAACAGCCC
>SRR4252619_15324901___Sbg5
TCTGATCTGATCTATAGCTCAACAGCCCT
>SRR4252619_15469694___Sbg5
TCTGATCTGATCTATAGCTCAACAGCCC

>SRR4252619_6762302___Sbg5
TCTGATCTGATCTATAGCTCAACAGC
>SRR4252619_7589072___Sbg5
TCTGATCTGATCTATAGCTCAACAGCCCT
>SRR4252619_8179813___Sbg5
TCTGATCTGATCTATAGCTCAACAGCCCT
>SRR4252619_8546881___Sbg5
TCTGATCTGATCTATAGCTCAACAGCCCT
>SRR4252620_6001168___Sbg5
TCTGATCTGATCTATAGCTCAACAGCCCT
>SRR4252621_10303508___Sbg5
TCTGATCTGATCTATAGCTCAACAGCCC
>SRR4252621_10467425___Sbg5
TCTGATCTGATCTATAGCTCAACAGCCC
>SRR4252621_1338570___Sbg5
TCTGATCTGATCTATAGCTCAACAGCCCT
>SRR4252621_5713768___Sbg5
TCTGATCTGATCTATAGCTCAACAGCCCT
>SRR4252621_9748420___Sbg5
TCTGATCTGATCTATAGCTCAACAGCCC
>SRR4252623_11315799___Sbg5
TCTGATCTGATCTATAGCTCAACAGCCCT
>SRR4252623_12350762___Sbg5
TCTGATCTGATCTATAGCTCAACAGCCCT
>SRR4252623_4344088___Sbg5
TCTGATCTGATCTATAGCTCAACAGCCCT
>SRR4252623_5436405___Sbg5
TCTGATCTGATCTATAGCTCAACAGCCCT
>SRR4252624_1429495___Sbg5
TCTGATCTGATCTATAGCTCAACAGCCCT
>SRR4252624_4876100___Sbg5
TCTGATCTGATCTATAGCTCAACAGCCCTA
>SRR4252624_6054794___Sbg5
TCTGATCTGATCTATAGCTCAACAGCCCT
>SRR4252624_7107157___Sbg5
TCTGATCTGATCTATAGCTCAACAGCCCTA
>SRR4252624_7547032___Sbg5
TCTGATCTGATCTATAGCTCAACAGCCCT
>SRR4252624_8860684___Sbg5
TCTGATCTGATCTATAGCTCAACAGCCCT
>SRR4252625_13640319___Sbg5
TCTGATCTGATCTATAGCTCAACAGCCCT
>SRR4252615_80715___Sbg5
TCTGATCTGATCTATAGCTCAACAGCCCT
>SRR4252616_1641888___Sbg5
TCTGATCTGATCTATAGCTCAACAGCCCT
>SRR4252616_6744754___Sbg5
TCTGATCTGATCTATAGCTCAACAGCCCT
>SRR4252617_15748959___Sbg5
TCTGATCTGATCTATAGCTCAACAGCCCT
>SRR4252620_6553048___Sbg5
TCTGATCTGATCTATAGCTCAACAGCC
>SRR4252614_9402177___Sbg5
CTGATCTGATCTATAGCTCAACAGCCCTA
>SRR4252619_12319638___Sbg5
CTGATCTGATCTATAGCTCAACAGCCCT
>SRR4252612_353681___Sbg5
CTGATCTGATCTATAGCTCAACAGCCCTA
>SRR4252605_164228___Sbg5
TGATCTGATCTATAGCTCAACAGCCC
>SRR4252611_13865386___Sbg5
TGATCTGATCTATAGCTCAACAGCCCTAT
>SRR4252611_14603866___Sbg5
TGATCTGATCTATAGCTCAACAGCCCTAT
>SRR4252611_15472559___Sbg5
TGATCTGATCTATAGCTCAACAGCCCTA
>SRR4252611_1726475___Sbg5
TGATCTGATCTATAGCTCAACAGCCCTAT
>SRR4252611_877333___Sbg5
TGATCTGATCTATAGCTCAACAGCCCTA

>SRR4252611_9994795___Sbg5
TGATCTGATCTATAGCTCAACAGCCCTAT
>SRR4252620_5139198___Sbg5
TGATCTGATCTATAGCTCAACAGCCCTAT
>SRR4252620_6196688___Sbg5
TGATCTGATCTATAGCTCAACAGCCCT
>SRR4252620_6737999___Sbg5
TGATCTGATCTATAGCTCAACAGCCCTAT
>SRR4252621_8765324___Sbg5
TGATCTGATCTATAGCTCAACAGCCCTA
>SRR4252621_8781817___Sbg5
TGATCTGATCTATAGCTCAACAGCCCTAT
>SRR4252622_1949858___Sbg5
TGATCTGATCTATAGCTCAACAGCCCTA
>SRR4252622_3020263___Sbg5
TGATCTGATCTATAGCTCAACAGCCCTA
>SRR4252625_9014213___Sbg5
TGATCTGATCTATAGCTCAACAGCCC
>SRR4252611_11158186___Sbg5
TGATCTGATCTATAGCTCAACAGCCCTA
>SRR4252611_8080910___Sbg5
TGATCTGATCTATAGCTCAACAGCCCT
>SRR4252611_959913___Sbg5
TGATCTGATCTATAGCTCAACAGCCCTA
>SRR4252615_14772340___Sbg5
TGATCTGATCTATAGCTCAACAGCCCT
>SRR4252615_9104861___Sbg5
GATCTGATCTATAGCTCAACAGCCCTAT
>SRR4252617_4226841___Sbg5
GATCTGATCTATAGCTCAACAGCCCTAT
>SRR4252610_8275960___Sbg5
GATCTGATCTATAGCTCAACAGCCCTA
>SRR4252611_14340865___Sbg5
GATCTGATCTATAGCTCAACAGCCCTA
>SRR4252611_8368285___Sbg5
GATCTGATCTATAGCTCAACAGCCCTA
>SRR4252612_1062355___Sbg5
GATCTGATCTATAGCTCAACAGCCCTAT
>SRR4252612_5548825___Sbg5
GATCTGATCTATAGCTCAACAGCCCTA
>SRR4252613_7059664___Sbg5
GATCTGATCTATAGCTCAACAGCCCTA
>SRR4252619_9936652___Sbg5
GATCTGATCTATAGCTCAACAGCCCTA
>SRR4252621_6514264___Sbg5
GATCTGATCTATAGCTCAACAGCCCTA
>SRR4252614_3910318___Sbg5
ATCTGATCTATAGCTCAACAGCCCTA
>SRR4252611_12694937___Sbg5
ATCTGATCTATAGCTCAACAGCCCTAT
>SRR4252607_14081511___Sbg5
TCTGATCTATAGCTCAACAGCCCTAT
>SRR4252614_6249388___Sbg5
ACCGAGCGGCTGCCGCCATAGGGCGGAATA
>SRR4252606_5221907___Sbg5
CCACAGCCGGTAACCCCGGTTTTCTAGACCA
>SRR4252611_13768352___Sbg5
TCACTATCAACAATGACATATGCCTTCTCT
>SRR4252609_9262066___Sbg5
TATTTGATCATTGTGTATGTGTGCGG
>SRR4252610_6336628___Sbg5
TCGGTGTGTAAGTGTGTGAATAGGAGGA
>SRR4252607_10980121___Sbg5
TTGGACGGAATTAACGGGGGTAGTATGGT
>SRR4252613_8133729___Sbg5
TTGGACGGAATTAACGGGGGTAGTATGG
>SRR4252616_3413283___Sbg5
CCCCACAGCCGATAACCCCGGTTTTCTA
>SRR4252621_9606596___Sbg5
TCACTATCAACAATGACGTATGCCTTCT

>SRR4252610_10270315___Sbg5
TAGGACGGAATTAACGGGGGGTAGCATGG
>SRR4252611_8708456___Sbg5
TAGTGATCAGAAGTTGTACACCACCACTCC
>SRR4252624_5749580___Sbg5
TGACGAAAGTGAATATGAAAGAGGGAGAA
>SRR4252614_5586999___Sbg5
TCATTGCGGAGGAGTTCGGGATTTATC
>SRR4252608_1064739___Sbg5
ACAATTTAGCGATCAGAAGTTGTACACCA
>SRR4252609_15771290___Sbg5
GTCCCCATCTGACGGACGGATCACTATCAA
>SRR4252621_13487650___Sbg5
TGTGTGTGTGGTGTGGTACGATGTGAGT
>SRR4252608_11505712___Sbg5
ACGATGTGAGTGACGATTATGGATATGAA
>SRR4252608_1216519___Sbg5
TTAGTGATCAGAAGTTGTACACAACCACTC
>SRR4252610_10736315___Sbg5
AGCTATTTGATCATTGTGTATGTGTGT
>SRR4252610_3862905___Sbg5
TATTTGATCATTGTGTATGTGTGTGGA
>SRR4252612_8589578___Sbg5
ACCCCACAGCCGATAACCCCGGTTTTCTA
>SRR4252605_6634602___Sbg5
TATTTGATCATTGTGTATGTGTGCGT
>SRR4252621_8558951___Sbg5
TGTGAGTGACGATTATGGATATGAAAG
>SRR4252616_4600720___Sbg5
AGGTTTTCTAGACCAGCTATTTGATCA
>SRR4252606_1290849___Sbg5
TATGATGTGAGTGACGGTTATGGATATG
>SRR4252611_12702442___Sbg5
TCACTATCAACAATGACATATGCCTTCT
>SRR4252612_6616884___Sbg5
TTAGTGATCAGAAGTTGTACAGCACCACTC
>SRR4252605_5215400___Sbg5
CCACAGCCGATAACCCCGGTTTTCTA
>SRR4252608_3808249___Sbg5
TGTATGTGTGCGGATAGGAGGATGAGGT
>SRR4252607_10063441___Sbg5
TGTAAGTGTGTGAATAGGAGGATGAGTGT
>SRR4252620_5082581___Sbg5
TGGATATGAAAGAGGGAGAAGGTGAAAT
>SRR4252610_8630724___Sbg5
CCCCCATCCGACGGACGGATCACTATCAA
>SRR4252611_14699499___Sbg5
AATACTACGCTGATCCCCACAGCCGATAA
>SRR4252611_5067322___Sbg5
AATACTACGCTGATCCCCACAGCCGATA
>SRR4252612_1804610___Sbg5
TGCGGATAGGAGGATGAGTGTGTGAAT
>SRR4252624_2071885___Sbg5
TGTGTGTATAGTGTTGTATTGTGTGTGAG
>SRR4252610_12850829___Sbg5
TGTGTGTATAGTGTTGTATTGTGTGTGTT
>SRR4252608_4689733___Sbg5
CCCCACAGCCGATAACCCCGGTTTTCTA
>SRR4252607_12518085___Sbg5
ACAATTCAGTGATCAGAAGTTGTACACCA
>SRR4252610_9023256___Sbg5
CAATTCAGTGATCAGAAGTTGTACACCA
>SRR4252607_3277175___Sbg5
TCACTATCAACAATGACATATGACTT
>SRR4252606_10016759___Sbg5
TAGTGTTGTATTGTGTGTTTGGTGTG
>SRR4252605_4981720___Sbg5
AGCTATTTGATCATTGTGTATGTGTGCGG
>SRR4252612_3682637___Sbg5
TTTGATCATTGTGTATGTGTGCGGATA

```
>SRR4252621_2247108___Sbg5
TCATTGTGTATGTGTGCGGATAGGAGGA
>SRR4252605_9666493___Sbg5
TGTGTGCGGATAGGAGGATGAGTGTGTA
>SRR4252610_12270344___Sbg5
TGTGTGCGGATAGGAGGATGAGTGTGTA
>SRR4252612_4072971___Sbg5
AATGGATCGGTGTGTAAGTGTGTGAATA
>SRR4252609_11088704___Sbg5
GGAGCGGCTGCCGCCATAGGGCGGAA
>SRR4252609_8788059___Sbg5
TCATTGTGTATGTGTGCGGATAGGAG
>SRR4252624_10521168___Sbg5
TATGAAAGAGGGAGAAGGTGAAACCTGG
>SRR4252614_227914___Sbg5
TGAAAGAGGGAGAAGGTGAAACCTGG
>SRR4252611_11571767___Sbg5
TGATGTGAGTGACGATTATGGATATGAA
>SRR4252612_1735701___Sbg5
GTGAGTGACGATTATGGATATGAAAGAG
>SRR4252624_6004877___Sbg5
TATGAAAGAGGGAGAAGGTGAAACCT
>SRR4252606_6469195___Sbg5
TTAGTGATCAGAAGTTGTACACCACCAC
>SRR4252610_7893626___Sbg5
TATGATGTGAGTGACGATTATGAATATG
>SRR4252621_7565201___Sbg5
CGTCCCATCCGACGGACGGATCACTATCAA
>SRR4252605_9564272___Sbg5
CAGGATTCGAAATTTTAATTAATTACTC
>SRR4252609_4284713___Sbg5
AAATGTGAGTGACGATTATGGATATGA
>SRR4252609_5286178___Sbg5
AAATGTGAGTGACGATTATGGATATGA
>SRR4252621_2975327___Sbg5
AAATGTGAGTGACGATTATGGATATGAA
>SRR4252612_2657973___Sbg5
AATGTGAGTGACGATTATGGATATGAAAGA
>SRR4252611_13659108___Sbg5
ACAGCCGATAACCCCGGTTTTCTAGAC
>SRR4252608_1684569___Sbg5
ACAATTTAGTGATCAGAAGTTGTACA
>SRR4252611_4967044___Sbg5
TACCGAGCGGCTGCCGCCATAGGACGGAA
>SRR4252606_8345297___Sbg5
GCTTAACGTCCCCATCCGACGGACGGATC
>SRR4252610_11795706___Sbg5
TTAGTGATCAGAAGTTGTACACCACCACTCC
>SRR4252624_8416570___Sbg5
GTCAATACTACGCTGGTCCCCACAGCCGA
>SRR4252623_7477068___Sbg5
GTGTTGTATTGTGTGTGTGGTGTGGTG
>SRR4252611_7087770___Sbg5
ATAGCTCAACAGCCCGATGTGGGCCAAGG
>SRR4252609_4238110___Sbg5
TAGGACGGAATTAACGGGGGGCAGTATGG
>SRR4252609_7045007___Sbg5
CACAGCCGATAACCCCGGTTTTCTAGACCA
>SRR4252620_7347131___Sbg5
TATGATGTGAGTGACGATTATGGAAAT
>SRR4252619_6679930___Sbg5
TGTGAGAGACGATTATGGATATGAAAGAG
>SRR4252610_12869782___Sbg5
ACAATTTAGTGATCAGAAGTTGTACA
>SRR4252614_2321620___Sbg5
GGTCAGTACTATGCTGATCCCCACAGCCGA
>SRR4252613_7444405___Sbg5
GATCCCCACAGCCGATAACCCCGGTT
>SRR4252606_7632172___Sbg5
CCCCATCCGACGGACGGATCACTATCAA
```

>SRR4252619_7489363___Sbg5
GATGTGAGTGACGATTATGGATATGAA
>SRR4252605_1266187___Sbg5
ACAATTTAGTGATCAGAAGTTGTACACCA
>SRR4252606_1003015___Sbg5
TATTTGATCATTGTGTATGTGTGTGGAT
>SRR4252606_6116610___Sbg5
TAGGACGGAATTAACGGGGGGTAGCATGG
>SRR4252614_9136499___Sbg5
ATGGTATGATGTGAGTGACGATTATGGA
>SRR4252605_2315264___Sbg5
TTAGTGATCAGAAGTTGTACACCACCACTC
>SRR4252606_4930960___Sbg5
TTAGTGATCAGAAGTTGTACACCACCACTCT
>SRR4252608_13236946___Sbg5
TTAGTGATCAGAAGTTGTACACCACCACTCT
>SRR4252612_2026960___Sbg5
TTAGTGATCAGAAGTTGTACACCACCACTCT
>SRR4252620_719273___Sbg5
ATTTAGTGATCAGAAGTTGTACACCACCA
>SRR4252622_8120194___Sbg5
GCATGTAACCTACTCCTTTCGAAAAGCA
>SRR4252610_6546549___Sbg5
TTAGTGATCAGAAGTTGTACACCACCACTC
>SRR4252607_4927373___Sbg5
ACAATTTAGTGATCAGAAGTTGTACACCA
>SRR4252605_8699847___Sbg5
TTAGTGATCAGAAGTTGTACACCACCACTCT
>SRR4252612_3557775___Sbg5
TTAGTGATCAGAAGTTGTACACCACCACTCT
>SRR4252619_12805457___Sbg5
TTAGTGATCAGAAGTTGTACACCACCACTCT
>SRR4252610_4001492___Sbg5
TAGTGATCAGAAGTTGTACACCACCACTCT
>SRR4252619_13142988___Sbg5
AAACGTCCCCATCCGACGGACGGATCACT
>SRR4252610_1124400___Sbg5
AGTACAATTTAGTGATCAGAAGTTGTA
>SRR4252619_4695228___Sbg5
TTAGTGATCAGAAGTTGTACACCACCATTC
>SRR4252609_9323019___Sbg5
TCATTGTGTATGTGTGCGGATAGGAGGA
>SRR4252612_7562731___Sbg5
TGTATTGTGTGTGTGGTGTGGTATGA
>SRR4252619_9273760___Sbg5
TTAGTGATTAGAAGTTGTACACCACCA
>SRR4252621_7493350___Sbg5
TCATTGTGTATGTGTGCGGATAGGAGGA
>SRR4252612_5516607___Sbg5
AGCTATTTGATCATTGTGTATGTGTGCG
>SRR4252621_7720927___Sbg5
ATATGTGAGTGACGATTATGGATATGAA
>SRR4252606_2356270___Sbg5
AACATAGTCATTGCGGAGGAGTTCGGGATT
>SRR4252618_5408311___Sbg5
TTTAACGTCCCCATCCGACGGACGGATCA
>SRR4252610_11532647___Sbg5
TTAGTGATCAGAAGTTGTACACCACCACTT
>SRR4252606_4045670___Sbg5
TATTTGATCATTGTGTATGTGTGTGGAT
>SRR4252610_10972843___Sbg5
TATGATGTGAGTGACGATTATGGATC
>SRR4252610_12790327___Sbg5
TTAGTGATCAGAAGTTGTACACCACCACTT
>SRR4252610_8984034___Sbg5
TTAGTGATCAGAAGTTGTACACCACCACTT
>SRR4252612_7985785___Sbg5
TTAGTGATCAGAAGTTGTACACCACCACTT
>SRR4252626_8084374___Sbg5
TTAGTGATCAGAAGTTGTACACCACCACTT

>SRR4252614_7740686___Sbg5
TCAGTACTATGCTGGTCCCCACAGCCGA
>SRR4252611_15127609___Sbg5
GTCCCCATCCGACGGACGGATCACTATCA
>SRR4252611_13719412___Sbg5
TTAGTGATCAGAAGTTGTACACCACCACTC
>SRR4252611_4691425___Sbg5
TATGAAAGAGGGAGAAGGTGAAACCCGG
>SRR4252612_4972081___Sbg5
GAAATTTAGTGATCAGAAGTTGTACACCACC
>SRR4252612_863818___Sbg5
GAAATTTAGTGATCAGAAGTTGTACACCAC
>SRR4252621_9900214___Sbg5
GAAATTTAGTGATCAGAAGTTGTACA
>SRR4252606_8006728___Sbg5
TTAGTGATCAGAAGTTGTACACCACCACTC
>SRR4252613_5196812___Sbg5
TCTATAGCTCAACAGCCCTATGTGGGCCAAT
>SRR4252606_766117___Sbg5
TTAGTGATCAGAAGTTGTACACCACCACTT
>SRR4252612_1505557___Sbg5
TTAGTGATCAGAAGTTGTACACCACCACTT
>SRR4252608_3487919___Sbg5
AGTTAATGGATCGGTGTGTAAGTGTGTGA
>SRR4252622_320045___Sbg5
TTAGTGATCAGAAGTTGTACACCACTACTC
>SRR4252611_15127682___Sbg5
GCAATTTAGTGATCAGAAGTTGTACACCA
>SRR4252610_9676871___Sbg5
ACCCACAGCCGATAACCCCGGTTTTCTA
>SRR4252614_9743768___Sbg5
ACCCACAGCCGATAACCCCGGTTTTCTA
>SRR4252619_11731031___Sbg5
GAGTGACGATTATGGATATGAAAGAGGGA
>SRR4252610_6027316___Sbg5
CACAATTTAGTGATCAGAAGTTGTACA
>SRR4252621_8524255___Sbg5
TTAGTGATCAGAAGTTGTACACCACCACTCT
>SRR4252611_3729792___Sbg5
TAGTGATCAGAAGTTGTACACCACCACTCT
>SRR4252608_9874768___Sbg5
TATGATGTGAGTGACGATTATGGAAATG
>SRR4252625_9858454___Sbg5
TGTGGGCCAAGGCTTACCGAGCGGCTGT
>SRR4252611_2286175___Sbg5
GTGGTATGATGTGAGTGACGAAAGTGAAT
>SRR4252611_7540891___Sbg5
TGGTATGATGTGAGTGACGAAAGTGAATA
>SRR4252610_5641333___Sbg5
ACAATTTAGTGATCAGAAGTTGTACACCA
>SRR4252611_2563229___Sbg5
CACGTAGCCTACTCCTTTCGAAAAGCA
>SRR4252625_1943717___Sbg5
CACGTAGCCTACTCCTTTCGAAAAGCA
>SRR4252611_13062309___Sbg5
ACGTAGCCTACTCCTTTCGAAAAGCA
>SRR4252608_4745877___Sbg5
TTAGTGATCAGAAGTTGTACACCAACACTC
>SRR4252610_12333642___Sbg5
TTAGTGATCAGAAGTTGTACACCAACACTC
>SRR4252625_5214109___Sbg5
TTAGTGATCAGAAGTTGTACACCAACAC
>SRR4252612_3159653___Sbg5
TAGTGATCAGAAGTTGTACACCAACAC
>SRR4252610_12553092___Sbg5
TGTGTGTATAGTGTTGTATTGTGTGTGC
>SRR4252612_4951136___Sbg5
TCAATTTAGTGATCAGAAGTTGTACAC
>SRR4252607_10802079___Sbg5
GCACAATTTAGTGATCAGAAGTTGTACA

```
>SRR4252606_3442662___Sbg5
TTAGTGATCAGAAGTTGTACACCACCACC
>SRR4252624_9312867___Sbg5
TTAGTGATCAGAAGTTGTACACCACCACCA
>SRR4252607_11739262___Sbg5
TATCCGACGGACGGATCACTATCAACA
>SRR4252606_356258___Sbg5
TATTTGATCATTGTGTATGTGTGCGG
>SRR4252608_3569724___Sbg5
TATGATGTGAGTGACGATTATGGATATGAT
>SRR4252610_1892234___Sbg5
TATGATGTGAGTGACGATTATGGATATGAT
>SRR4252606_463298___Sbg5
TTTGATCATTGTGTATGTGTGCGGATA
>SRR4252624_4706756___Sbg5
TTAGTGATCAGAAGTTGTACACCGCCACTC
>SRR4252606_7448442___Sbg5
GATCATTGTGTATGTGTGCGGATAGGAG
>SRR4252613_8564377___Sbg5
TCATTGTGTATGTGTGCGGATAGGAGGA
>SRR4252605_12514999___Sbg5
TATGAAAGAGGGAGAAGGTGAAACTTGG
>SRR4252605_5410254___Sbg5
TATGAAAGAGGGAGAAGGTGAAACTTGG
>SRR4252605_9268663___Sbg5
TATGAAAGAGGGAGAAGGTGAAACTTGG
>SRR4252611_10218077___Sbg5
TATGAAAGAGGGAGAAGGTGAAACTTGG
>SRR4252611_9278171___Sbg5
TATGAAAGAGGGAGAAGGTGAAACTTGG
>SRR4252621_11448287___Sbg5
TATGAAAGAGGGAGAAGGTGAAACTTGG
>SRR4252623_13140546___Sbg5
TATGAAAGAGGGAGAAGGTGAAACTTGG
>SRR4252605_174907___Sbg5
GCGGACGGATCACTATCAACAATGACATA
>SRR4252619_7609416___Sbg5
GCGGACGGATCACTATCAACAATGACATA
>SRR4252609_16845720___Sbg5
TCACTATCAACAATGACATATGCCTT
>SRR4252616_14150745___Sbg5
TATGAAAGAGGGAGAAGGTGAAACCTGG
>SRR4252611_14661902___Sbg5
TCTATAGCTCAACAGCCCGATGTGGGCCAT
>SRR4252610_7465838___Sbg5
TATTTGATCATTGTGTATGTGTGCGA
>SRR4252606_8065623___Sbg5
TCATTGTGTATGTGTGCGAATAGGAGGA
>SRR4252606_5999514___Sbg5
CCCACGGCCGATAACCCCGGTTTTCTAG
>SRR4252605_14845284___Sbg5
TAGTGATCAGAAGTTGTACACCACCACTCT
>SRR4252611_4989299___Sbg5
TAGTGATCAGAAGTTGTACACCACCACTCT
```
