# Supplementary material for: Identification and functional characterization of the German cockroach, Blattella germanica, short interspersed nuclear elements
Source: PLoS One. 2022 Jun 13;17(6):e0266699. doi: 10.1371/journal.pone.0266699 (PMC9191728; doi:10.1371/journal.pone.0266699)
Supplement: S3 Fig — (A)–The consensus nucleotide sequence of the corresponding SINE with the designation of the tRNA structure. The tRNA nucleotide sequences are highlighted in gray; “A” and “B” boxes are highlighted in blue; nucleotides other than canonical nucleotides are highlighted in yellow background and red font (explanation in the text). (B)–Distribution of the corresponding SINE copies in the B. germanica genome. Blue vertical lines indicate the positions of SINE localized in direct orientation, red vertical lines—in inverted orientation. (C)–The result of the alignment of the consensus sequences and the sequences of twelve similar to the consensus sequence SINE copies presented in the genome, along with the nearest environment. Direct repeats flanking the retrotransposon are highlighted with a green background. Poly(A) sequences and short microsatellite repeats are highlighted in yellow, blue and pink backgrounds. Variable nucleotides are highlighted in red fonts. (D)–Alignment of Sbg1, Sbg8 and Sbg9, demonstrating that Sbg9 was formed by combining Sbg1 and Sbg8. (PDF) [file pone.0266699.s006.pdf]

Sbg1

A

```
>Consensus_Sbg1 (306b)
GGGGCACGCGGTAGCCTAGCGGTTACGGCGCTGGACTACAAGCCGGAAGGTCGGGGGTTCGATTCCCCATGGTGCCATTGGAATTTTTCAATTGGTA
TAATCCTTCTGGCCGCACTATGGCCCTGGGGTGCACTCAGCCTCTAACAGAAATGAGTACCAGGAGCTTTCCTGGGGGTGAAGGCGGCTGCCTCGTG
GGGCTGACAACCTCACAGGCCCTGTATGCCGATTGTCTGTAAAGATGGGTGCCGTAACCTCCCGTCACCCTAAGGGCCTTCCTAGGCCTGTATAAGG
GATACCTTTACCTTT

Consensus_Sbg1.trna1 (3-77)        Length: 75 bp
Type: Cys         Anticodon: ACA at 36-38 (38-40)     Score: 26.8
Possible pseudogene
         *    |    *    |    *    |    *    |    *    |    *    |    *    |
Seq: GGCAcgCGGTAGCCTAGCGGTtACGGCGCTGGACTACAAGCCGGAAGGtCGGGGGTTCGATTCCCCATGGTGCCA
Str: >>>>..>.>..>>>.........<<<..>.>.........<.<.......>>>>.......<<<<.<.<<<<<.
```

B

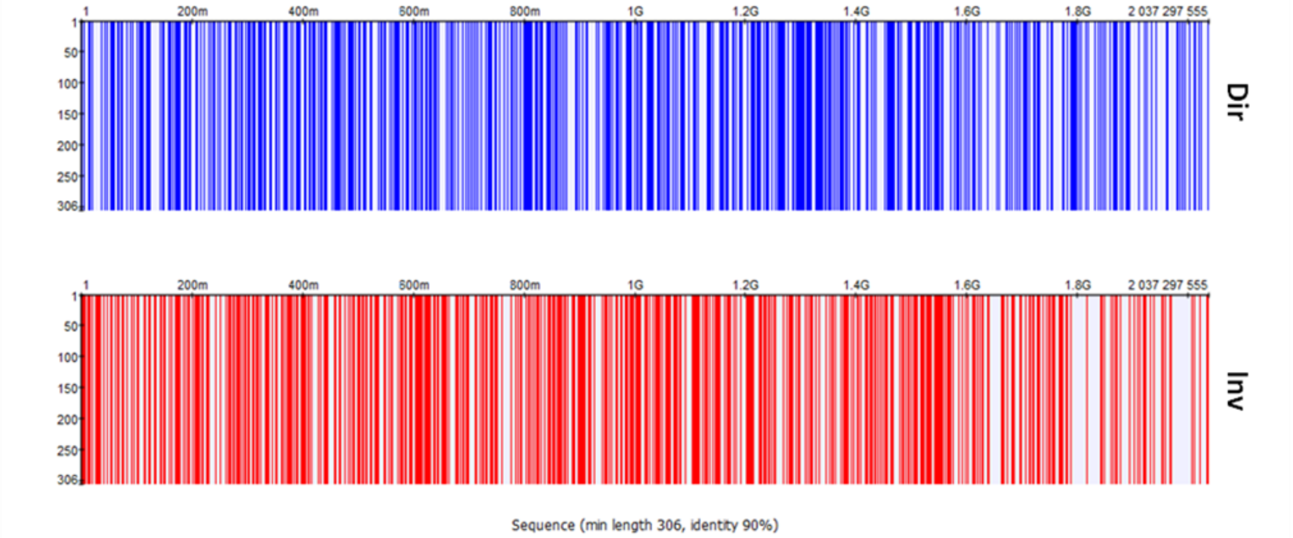

Number of copies: 90% - 1354; 80% - 2100

C

```
Consensus_Sbg1    -----------------ggggcacgcggtagcctagcggttacggcgctggactacaagc
Sbg1_1           -----aatatgtactgt-ggggcacgcggtagcctagcggttacggcgctggactacaagc
Sbg1_2            ----tatatgcacaaggggggcacgcggtagcctagcggttacggcgctggactacaagc
Sbg1_3           -----aagtatgtatgt-ggggcacgcggtagcctagcggttacggcgctggactacaagc
Sbg1_4            -----ccaggttggaaggggcacgcggtagcctagcggttacggcgctggactacaagc
Sbg1_5            ---tacaatattacgaggggcacgcggtagcctagcggttacggcgctggactacaagc
Sbg1_6           -----actatgccttatagggcacgcggtagcctagcggttacggcgctggactacaagc
Sbg1_7           -----agtattagtatt-ggggcacgcggtagcctagcggttacggcgctggactacaagc
Sbg1_8           ------tcttatgaatt-ggggcacgcggtagcctagcggttacggcgctggactacaagc
Sbg1_9            --atataagaatttatggggcacgcggtagcctagcggttacggcgctggactacaagc
Sbg1_10           ----taatagtaaagtggggcacgcggtagcctagcggttacggcgctggactacaagc
Sbg1_11           tagttatgagcagcatggggcacgcggtagcctagcggttacggcgctggactacaagc
Sbg1_12           --tacatataaaccac-ggggcacgcggtagcctagcggttaAggcgctggactacaagc
                                   ************************* *****************
                                          [ 240 bp ]
Consensus_Sbg1    gtataaggatacctttaccttt---------------------
Sbg1_1            gtataaggatacctttaccttt-------aatatgtactgt----
Sbg1_2            gtataaggatacctttaccttt-------tatatgcacaag----
Sbg1_3            gtataaggatacctttaccttt-------aagtatgtatgt----
Sbg1_4            gtataaggatacctttacctttacctttaccaggttggaa-----
Sbg1_5            gtataaggatacctttaccttt------ttacaatattacga---
Sbg1_6            gtataaggatacctttaccttt-------actatgccttat----
Sbg1_7            gtataaggatacctttacctttacctt-agtattagtatt----
Sbg1_8            gtataaggatacctttacctat-------tcttatgaatt-----
Sbg1_9            gtataaggatacctttaccttt-------atataagaatttat--
Sbg1_10           gtataaggatacctttaccttt------ttaatagtaaagt----
Sbg1_11           gtataaggatacctttaccttt-------tagttatgagcagcat
Sbg1_12           gtataaggatacctttacctttacctt-tacatataaaccac--
                  **********************
```

Sbg2

A

```
>Consensus_Sbg2 (313b)
GACCAGCCCCATGGTCTAGTGGTCAGAGTGCCTGGCTACGGAGCAAGGGGTCCCGGGTTCGATTCCCGGCCAGAGCATAGGAAATTTTCTTTAATAT
AAGAATCTTTCCTGTGCTCGCCCATGGTCTGGTATTTAGGTTAAGTTAGGTTTAAGGACCCTCCTGGCTCTCCATATCCATTATCATCCTACCATTT
CTCATCGGGGTAACGTAACCCGCCTCCTAGGTGCCCCAACCTCAGAAATGGGTTACAAAAACAGCCATGCCAGAAGGGAATGCCAGAATGTCGAAAG
GACAACATGGCGGCATTGAATA

Consensus_Sbg2.trna1 (6-77)         Length: 72 bp
Type: Arg          Anticodon: ACG at 33-35 (38-40)      Score: 34.79
Possible pseudogene
         *    |    *    |    *    |    *    |    *    |    *    |    *    |
Seq: GCCCCATGGTCTAGTGGTcAGAGTGCCTGGCTACGGAGCAAGGGGtCCCGGGTTCGATTCCCGGCCAGAGCA
Str: >>.>....>>.>........<.<<.>.>>.........<<.<.....>>>>>.......<<<<<...<.<<.
```

B

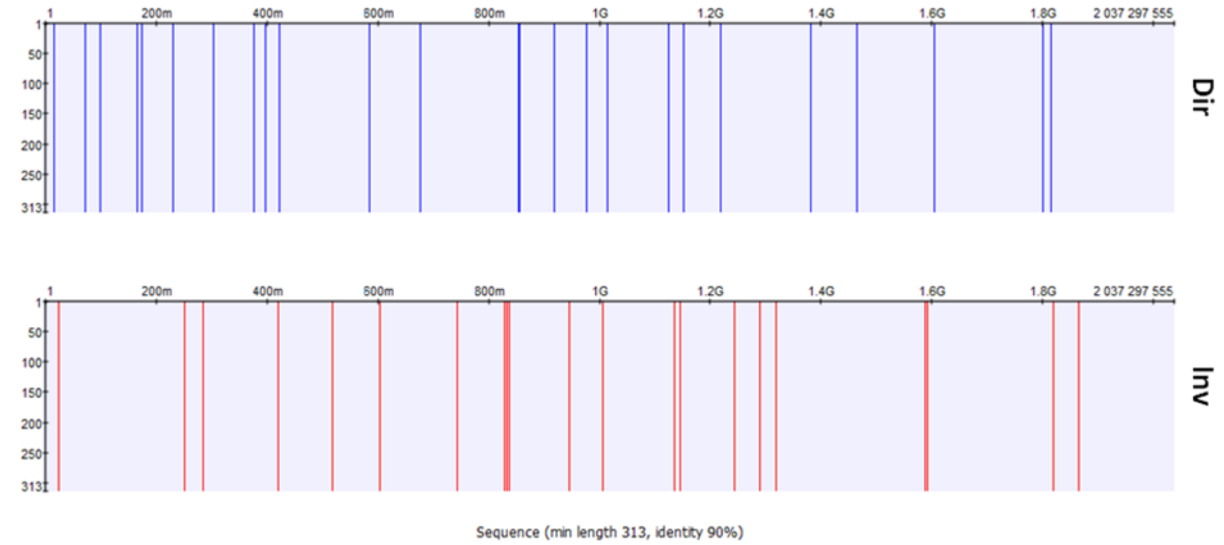

Number of copies: 90% - 49; 80% - 243

C

```
Consensus_Sbg2  ----------------gaccagccccatggtctagtggtcagagtgcctggctacggagc
Sbg2_1          ----cagttataaactgacTagccccatggtctagtggtcagagtgcctggctacggagc
Sbg2_2          -----ttgaataaaaagaccagccccatggtctagtggtcagagtgcctggctacggagc
Sbg2_3          ---aatgtattgacctgaccagccccatggtctagtggtcagagtgcctggctacggagc
Sbg2_4          ------ttgtaaataagaccagccccatggtctagtggtcagagtgcctggctacggagc
Sbg2_5          ---tTtttttttacatgaccagcTccatggtctagtggtcagagtgcctagctacggagc
Sbg2_6          --tattcctaaattaagaccagccccatgAtctagtggtcagagtgcctggctacggagc
Sbg2_7          -atattcttcaatcaagaccagccccatggtctagtggtcagagtgcctagctacggagc
Sbg2_8          ----tatatcatttatgaccagccccatggtctagtggtcagagtAcctggctacggagc
Sbg2_9          ---acattctttaaaggaccagccccTtggtctagtggtcagagtAcctggctacagagc
Sbg2_10         ----tctttttctgcTgaccagccccatggtctagtggtcagagtgcctggctacggagc
Sbg2_11         attgttagaacataaCgaccagccccatggtctagtggtcagagtgcctggctaTggagc
Sbg2_12         ---taatttgaatttcgaccagccccatggtctagtggtcagagtgcctAgctacAgagc
                                *** .***.** **.**************.***.****..****
```

[240 bp]

```
Consensus_Sbg2  tcgaaaggacaacatggcggcattgaata-------------------------------------------
Sbg2_1          tcgaaaggacaacatggcggcattgaataTaaaaaa--------------------cagttataaact----
Sbg2_2          tcgaaaAgacaacatggcggTattgaataTaaaaaaaaaTa----------------ttgaataaaa-----
Sbg2_3          tcgaaaAgacaacatggcggTattgaataTaaaaaaaaaaaGTaaTaaaaaa----aatgtattgacct---
Sbg2_4          tcgaaaggacaacatggcAgcattgaataTaaaaaaaaaaaaa-------------ttgta-agtaa-----
Sbg2_5          tcgaaaggacaacatggcggcattgaataTaTaaaaaaaaaaaaaa----------tGtttttttacat---
Sbg2_6          tcgaaaggacaacatggcggcattgaataaaaaaaaa-------------------tattcctaaattaa--
Sbg2_7          tcgaaaAgaAaacatggTggcattgaataaaaaaaaaaT-----------------atattcttcaatcaa-
Sbg2_8          tcgaaaAgacaacatggTggcattgaataGaaaaaaaaa-----------------tatatcatttat----
Sbg2_9          tcAaaaggacaacatggcggcattgaataaaTaaaaaaTaaa--------------acattctttaaag---
Sbg2_10         tcgaaaggacaacGtggcggcaCtgaataTaTaaaaaaaaaaaaaaaa--------tctttttctgcC----
Sbg2_11         tcgaaaggacaacatggcggcattgaataaaaaaaaaaaaa---------------attgttagaacataaA
Sbg2_12         tcgaaaAgacaacatggTggcattgaataTaTTaaaaaaaaaaTaaTaaTaaaaaataatttgaatttc---
                **.***.** ***.***..*.*.******
```

Sbg3

A

```
>Consensus_Sbg3 (613b)
ACCGAGCGAGGTGGCTCCGTGGTAAGACGCGGGACTCGCATTCGGGAGGTCACGGGTTCAAATCCCGGGGCCGACCCCAACCTGACTGAGGTTTTTC
CGTGGTTTTCCTCAGTTAATTAAGGCAAATGCTGGGTTGGCTTTTCATTTCCACGGTCCCCACCCCTCCCCTTTCACCTTATCATTAATCCATATCA
TTTCATAACTATCATTCATCCGTATGCAAAAATGTGACGTCAGGCTTCGCGCATGTCTCGGTGCGCTTGCGCAGACTATTCCTAATCATACATATTC
CTACAAGTCCTGAGCCTTAGCAACCAACTGTCTGAAGTCAGACCAGGAAACCTCGGGCAACAAAAGAAAGACCTTGGGAGTACAGGGAGGCCAGGCA
GCCTTAGTTATAAGATCCTGATCAACCGGCGAGATGGGCTAAGGGATATGTATGTTCTGGGAGTATGTCAGCAAGCGCATTGAGGGGCTCATGCAGC
CGGAGGGTAATTCAAACCGGTTAACATCACCATCTCATATCCATTCATCGGGGGCAGTACAATATCCTCAGGATCGGTGCTAAAAGGGTCGCCCATA
ACCCTCTCCCGTTTCCCTCTAACAAATCTAA

Consensus_Sbg3.trna1 (10-73)        Length: 64 bp
Type: Ala          Anticodon: CGC at 28-30 (37-39)     Score: 20.82
Possible pseudogene
             *    |    *    |    *    |    *    |    *    |    *    |
Seq: GGTGGCTCCGTGGTAAGACGCGGGACTCGCATTCGGGAGGtCACGGGTTCAAATCCCGGGGCCG
Str: >>..>.>.........<.<.>..>>.......<<..<......>>>>.......<<<<...<<.
```

B

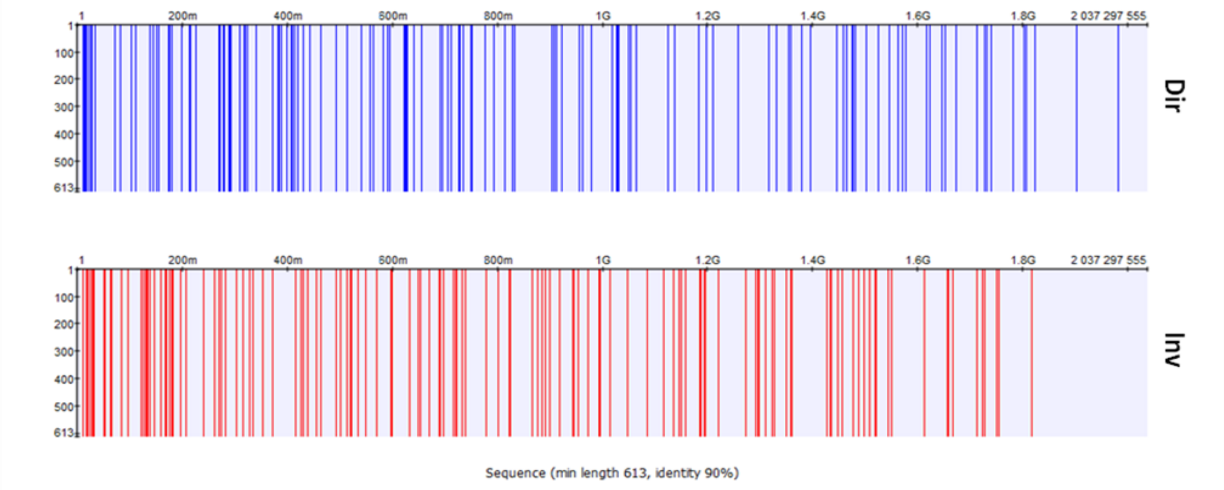

Number of copies: 90% - 277; 80% - 421

C

```
Consensus_Sbg3   ----------------------------accgagcgaggtggctccgtggtaagacgcgggactc
Sbg3_1           ------------acactgttcaa-----TccgagcgaggtggctccAtggtaagacgcgggactc
Sbg3_2           -----------aggggccatttt-----accgagcgaggtggctccgtggtaagacgcgggactc
Sbg3_3           ------------gaggttattta-----accgagcgaggtggctccgtggtaagacgcgggactc
Sbg3_4           -------------atgtaacatt----aaccgagcgaggtggctccgtggtaagacgcgggactc
Sbg3_5           ------------ttaggTtaatt-----accgagcgaggtggctccgtggtaagacgcgggactc
Sbg3_6          -------------aggaaagccattggttaccgagcgaggtggctccgtggtaagacgcgggactc
Sbg3_7           -------------ggtgcacata-----accgagcgaggtggctccgtggtaagacgcgggactc
Sbg3_8           ------------aagcccgaaca-----accgagcgaTgtggctccgtggtaagacgcgggactc
Sbg3_9           ------------tacttttatta-----accgagcgaggtggctccgtggtaagacgcgggactc
Sbg3_10          ---------------gagcaata-----accgagcgaggtggctccgtggtaagacgcgggactT
Sbg3_11          ------------tccctgttgat-----accgagcgaggtggctccgtggtaagTcgcgggactc
Sbg3_12          -------------ttattataaa-----accgagcgaggtggctccgtggtaagacgcgggactc
                                              ******** ********.******* *********.
```

[540 bp]

```
Consensus_Sbg3   ccataaccctctcccgtttccctctaacaaatctaa--------------------------
Sbg3_1           ccataaccctctcccgtttccctctaacaaatctaacaaa----------acactgttcaa-
Sbg3_2           ccataaccctctcccgtttccctctaaGaaatctaa--------------aggggccatttt
Sbg3_3           ccataaccctctcccgtttccctctaacaaatctaaaaatctaaaaa--gaggttattta-
Sbg3_4           ccataaccctctcccgtttccctctaacaaatctaa--------------atgtaacatt--
Sbg3_5           ccataaccctctcccgtttccctctaacaaatctaaaaaaaa-------ttaggAtaatt-
Sbg3_6           ccataaccctctcccgtttccctctaacaaatctaaacaaatctTa----aggaaagccat-
Sbg3_7           ccataaccctctcccgtttccctctaacaaatctaaaaaaaa-------ggtgcacata--
Sbg3_8           ccataaccctctcccgtttccctctaacaaatctaaatctaaa-------aagcccgaaca-
Sbg3_9           ccataaccctctcccgtttccctctaacaaatctaaatc-----------tacttttatta-
Sbg3_10          ccataaccctctcccgtttccctctaacaaatctaaaaatctaatctaagagcaata----
Sbg3_11          ccataaccctctcccgtttccctctaacaaatctaatctaa---------tccctgttgat-
Sbg3_12          ccataaccctctcccgtttccctctaacaaatctaaacaaatctaa----ttattataaa--
                 ************************ ********
```

Sbg4

A

```
>Consensus_Sbg4 (456b)
GTCCACCGTTGTGGAGTAGGGGATAGCGTATGCGCCTCTCACGCAGCGGGCCCGGGTTCGATTCCCGGTCGGGACAAGTTTCCTGGGTGAGGTTTTT
CTGGGGTTTTTCCTCACCCATAAGACAAATGTCAGGAAACTTTAGGCCCAAACGGTCCCCAGACATCATCTGGCCATCATGATCATCTTCATCATAT
TCGCCTTGTTGGTATGAGTGTGGGTGTGAATGGTGTCTTTGTCTATATGTTCGTGTTGTCTCGGAGGTGGCCCTGGCATTGAGCTGATCACTTATCC
AGGGAGGCCCTCCATGTCCTTGTGTAGTCAAAAAAGTATGAATGTATATGTGATCCATAGTCTATCCTCTCCCGACAGGTCTTGGCTCTGTAAGGCC
CGGGTGGCGTGAGTCATGTAAATAGTACCTACTAGTGGGGGAGGTAAAACTTGGAAAATGGAATGGAA

Consensus_Sbg4.trna1 (5-74)        Length: 70 bp
Type: Glu          Anticodon: CTC at 34-36 (38-40)     Score: 28.33
Possible pseudogene
         *    |    *    |    *    |    *    |    *    |    *    |    *
Seq: ACCGTTGTGGAGTAGGGGAtAGCGTATGCGCCTCTCACGCAGCGGGCCCGGGTTCGATTCCCGGTCGGGA
Str: .>>>..>..>.>>........<<.<....>>.......<<.......>>>>>.......<<<<<<<<<..
```

B

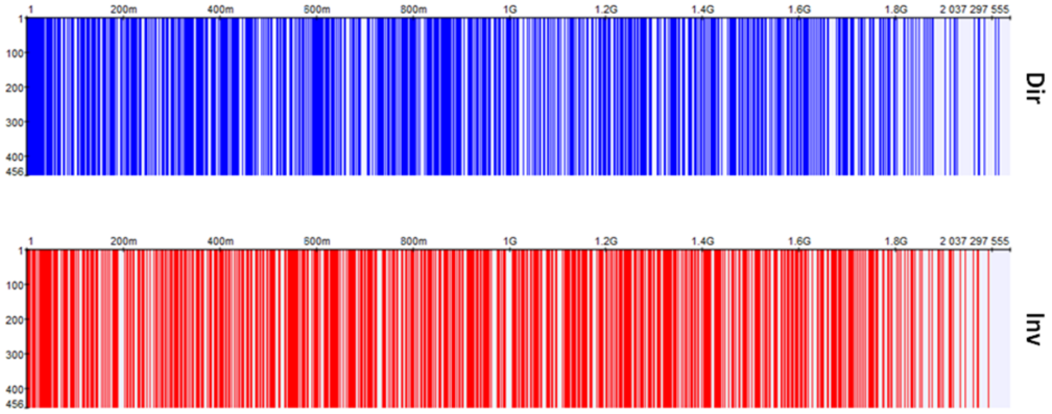

Sequence (min length 456, identity 90%)

Number of copies: 90% - 1849; 80% - 2329

C

```
Consensus_Sbg4  ---------------gtccaccgttgtggagtaggggatagcgtatgcgcctctcacgca
Sbg4_1          --ttatattcatta-gtccaccgttgtggagtaggggatagcgtatgcgcctctcacgca
Sbg4_2          ---tatattaatta-gtccaccgttgtggagtaggggatagcgtatgcgcctctcacgca
Sbg4_3          ---tactttcagaa-gtccaccgttgtggagtaggggatagcgtatgcgcctctcacgca
Sbg4_4          --taacttaaatcT-gtccaccgttgtggagtaggggatagcgtatgcgcctctcacgca
Sbg4_5          -taccttaaatctG-gtccaccgttgtggagtaggggatagcgtatgcgcctctcacgca
Sbg4_6          --taattcttattccgtccaccgttgtggagtaggggatagcgtatgcgcctctcacgca
Sbg4_7          --tgcttattacaG-gtccaccgttgtggagtaggggatagcgtatgcgcctctcacgca
Sbg4_8          ----cttttggtccagtccaccgttgtggagtaggggatagcgtatgcgcctctcacgca
Sbg4_9          ---taatgtttcat-gtccaccgttgtggagtaggggatagcgtatgcgcctctcacgca
Sbg4_10         --tagcacccaaac-gtccaccgttgtggagtaggggatagcgtatgcgcctctcacgca
Sbg4_11         Taagatgtttttag-gtccaccgttgtggagtaggggatagcgtatgcgcctctcacgca
Sbg4_12         -tgtaaagcagtgt-gtccaccgttgtggagtaggggatagcgtatgcgcctctcacgca
                               *********************************************

                                          [ 372 bp ]
Consensus_Sbg4  tactagtgggggaggtaaaacttggaaaatggaatggaa------------------------
Sbg4_1          tactagtgggggaggtaaaacttggaaaatggaatggaa----------ttatattcatta--
Sbg4_2          tactagtgggggaggtaaaacttggaaaatggaatggaatggaatTgaatatattaatta---
Sbg4_3          tactagtgggggaggtaaaacttggaaaatggaatggaatggaa-----tactttcagaa---
Sbg4_4          tactagtgggggaggtaaaacttggaaaatggaatggaatggaa-----taacttaaatcA--
Sbg4_5          tactagtgggggaggtaaaacttggaaaatggaatggaatggaa-----taccttaaatctC-
Sbg4_6          tactagtgggggaggtaaaacttggaaaatggaatggaatggaa-----taattcttattcc-
Sbg4_7          tactagtgggggaggtaaaacttggaaaatggaatggaatggaa-----tgcttattacaT--
Sbg4_8          tactagtgggggaggtaaaacttggaaaatggaatggaatggaa-----cttttggtcca---
Sbg4_9          tactagtgggggaggtaaaacttggaaaatggaatggaa----------taatgtttcat---
Sbg4_10         tactagtgggggaggtaaaacttggaaaatggaatggaatggaa-----tagcacccaaac--
Sbg4_11         tactagtgggggaggtaaaacttggaaaatggaatggaa----------Aaagatgtttttag
Sbg4_12         tactagtgggggaggtaaaacttggaaaatggaatggaa----------tgtaaagcagtgt-
                ***************************************
```

Sbg5

A

```
>Consensus_Sbg5 (582b)
GTCTGCCGAGCTGGCTCAGTGGTTAGCGTGCTTGCCTCTGATCCGGCCGGCCCGGGATCGAATCCCGGCAGGGTTGGGAATTTTAATTAAGTAATTA
ATTAAAATTTCGAATCCTGAGACTAGGAGGAGTGGTGGTGTACAACTTCTGATCACTAAATTGTGCACCAAATTCACCTGGGTTAAATCCCGAACTC
CTCCGCAATGACTATGTAGAGAAGGCATATGTCATTGTTGATAGTGATCCGTCCGTCGGATGGGGACGTTAAGCCGCCGGTCCCCTTGGTGCTTTTC
GAAAGGAGTAGGCTACATGCCGGCACCGGGTTTCACCTTCTCCCTCTTTCATATCCATAATCGTCACTCACATCATACCACACCACACACACAATAC
AACACTATACACACACTCATCCCCCTATCCACACACTTACACACCAATCCATTAGCTGGTCTAGAAAACCGGGGCTATCGGCTGTGGGGACCAGCGT
AGTACTGACCATACTACCCCCCGTTAATTCCGTCCTATGGCGGCAGCCGCTCGGTAAGCCTTGGCCCACATAGGGCTGTTGAGCTATAGATCAGATC
```

```
Consensus_Sbg5.trna1 (6-78)         Length: 73 bp
Type: Gln          Anticodon: CTG at 33-35 (38-40)     Score: 31.78
Possible pseudogene
         *    |    *    |    *    |    *    |    *    |    *    |    *    |
Seq: CCGAGCTGGCTCAGTGGTtAGCGTGCTTGCCTCTGATCCGGCCGGCCCGGGATCGAATCCCGGcaGGGTTGGG
Str: >>>>.>..>>.>........<.<<..>>>.........<<<.....>>>>>.......<<<<<...<.<<<<.
```

B

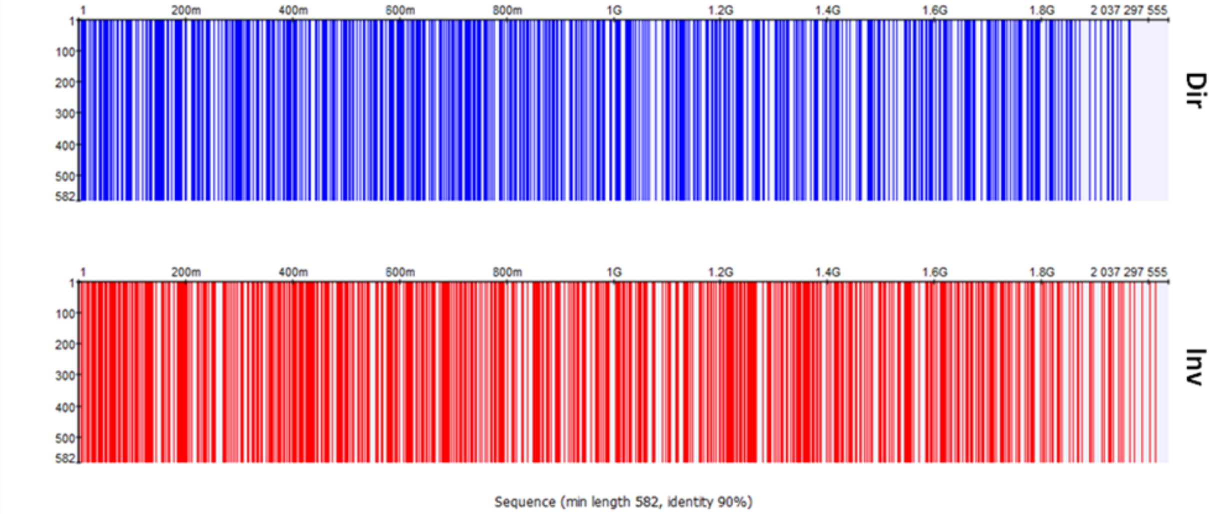

Number of copies: 90% - 1610; 80% - 2392

C

```
Consensus_Sbg5  -----------------gtctgccgagctggctcagtggttagcgtgcttgcctctgatccg
Sbg5_1          ----aagttataagt--gtctgccgagctggctcagtggttagcgtgcttgcctctgatccg
Sbg5_2          -agtgagttggcgga--gtctgccgagctggctcagtggttagcgtgcttgcctctgatccg
Sbg5_3          -----tataaaataa--gtctgccgagctggctcagtggttagcgtgcttgcctctgatccg
Sbg5_4          --agtaaccttataa--gtctgccgagctggctcagtggttagcgtgcttgcctctgatccg
Sbg5_5          agtatggtcagcact--gtctgccgagctggctcagtggttagcgtgcttgcctctgatccg
Sbg5_6          ---agcgtaaggatt--gtctgccgagctggctcagtggttagcgtgcttgcctctgatccg
Sbg5_7          ----aagactattag--gtctgccgagctggctcagtggttagcgtgcttgcctctgatccg
Sbg5_8          --aggcctagtatgg--gtctgccgagctggctcagtggttagcgtgcttgcctctgatccg
Sbg5_9          aatgtttaaaactgt--gtctgccgagctggctcagtggttagcgtgcttgcctctgatccg
Sbg5_10         -agtacaatatcctc--gtctgccgagctggctcagtggttagcgtgcttgcctctgatccg
Sbg5_11         ---tactacagtttg--gtctgccgagctggctcagtggttagcgtgcttgcctctgatccg
Sbg5_12         ---aataactttattgtgtctgccgagctggctcagtggttagcgtgcttgcctctgatccg
                                 **********************************************
```

[480 bp]

```
Consensus_Sbg5  cggcagccgctcggtaagccttggcccacatagggctgttgagctatagatcagatc------------------
Sbg5_1          cggcagccgctcggtaagccttggcccacatagggctgttgagctatagatcagatc---aagttataagt----
Sbg5_2          cggcagccgctcggtaagccttggcccacatagggctgttgagctatagatcagatc---agtgagttggcgga-
Sbg5_3          cggcagccgctcggtaagccttggcccacatagggctgttgagctatagatcagatc---tataaaataa-----
Sbg5_4          cggcagccgctcggtaagccttggcccacatagggctgttgagctatagatcagatc---agtaaccttataa--
Sbg5_5          cggcagccgctcggtaagccttggcccacatagggctgttgagctatagatcagatc---agtatggtcagcact
Sbg5_6          cggcagccgctcggtaagccttggcccacatagggctgttgagctatagatcagatc---agcgtaaggatt---
Sbg5_7          cggcagccgctcggtaagccttggcccacatagggctgttgagctatagatcagatc---aagactattag----
Sbg5_8          cggcagccgctcggtaagccttggcccacatagggctgttgagctatagatcagatc---aggcctagtatgg--
Sbg5_9          cggcagccgctcggtaagccttggcccacatagggctgttgagctatagatcagatc---aatgtttaaaactgt
Sbg5_10         cggcagccgctcggtaagccttggcccTcatagggctgttgagctatagatcagatc---agtacaatatcctc-
Sbg5_11         cggcagccgctcggtaagccttggcccacatagggctgttgagctatagatcagatcagatactacagtttg---
Sbg5_12         cggcagccgctcggtaagccttggcccacatagggctgttgagctatagatcagatc---aataactttatt---
                *************************** *****************************
```

Sbg6

A

```
>Consensus_Sbg6 (359b)
AGCGGCCTCAGTGGAACAATGGTAGCGTGCCAGCCTGTCGTTCCCGAGATCGTGGGTTCAAACCCGGCTGAGGTCGTCGGATTTTAGGGTACAAAA
AATCCATGGCACGCATGTCATCGGTTAGATGGCATGTAAAAGAACCCTTGGGTACACTTCGTACCCTCGGCAAAATTAAATCcCGGCCAAGTTTCGT
ACCCAAATAGAGTCCACACTAGCGCTCCTAGGAGCAATAGTGTGAACGTCCAAATCCGGCGCGAGAGTTCGCCACTAGTGGTGCCAACTCGGAGAAA
CTCTCATCCCTGGGGTCGGCCGGGTCTGACAACGACGTAGTAAGTCGAGATTCAGCCCATTAGATACA

Consensus_Sbg6.trna1 (4-76)         Length: 73 bp
Type: Asp          Anticodon: GTC at 34-36 (37-39)     Score: 30.34
Possible pseudogene
         *    |    *    |    *    |    *    |    *    |    *    |    *    |
Seq: GGCCTCAgTGGAACAATGGTAGCGTGCCAGCCTGTCGTTCCCGAGAtCGTGGGTTCAAACCCGGCTGAGGTCG
Str: >>>>>>>...>..>.......<..<.>..>.........<..<.....>>.>>.......<<.<<<<<<<<<.
```

B

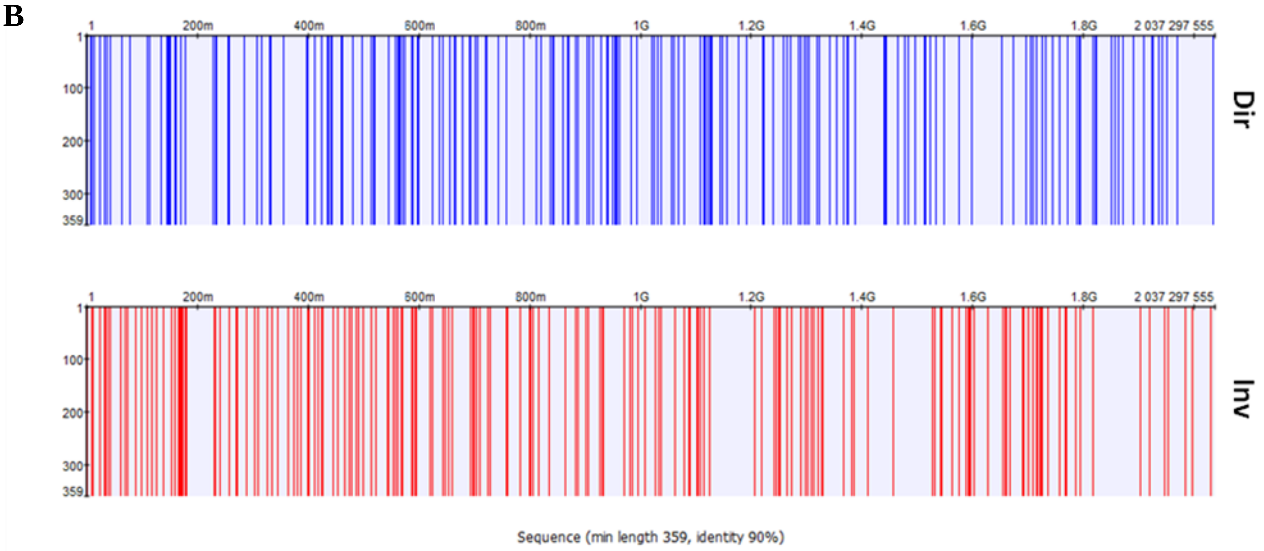

Number of copies: 90% - 404; 80% - 549

C

```
Consensus_Sbg6  ---------------agcggcctcagtggaacaatggtagcgtgccagcctgtcgttccc
Sbg6_1          ---cctaaaagtag-agcggcctcagtggaacaatggtagcgtgccagcctgtcgttccc
Sbg6_2          ---ataatggtggc-agcggcctcagtggaacaatggtaAcgtgccagcctgtcgttccc
Sbg6_3          --tatacaaaatat-agcggcctcagtggaacaatggtagcgtgccagcctgtcgttcTc
Sbg6_4          -ggattagagtcag-agcggcctcagtggaacaatggtagcgtgccagcctgtcgttccc
Sbg6_5          ----cttacttgta-agcggcctcagtggaacaatggtagcgtgccagcctgtcgAtccc
Sbg6_6          -gatcacaaatcca-agcggcctcagtggaacaatggtagcgtgccagcctgtcgttccc
Sbg6_7          -ctaaacataatataagcggcctcagtggaacaatggtagcgtgccagcctgtcgttccc
Sbg6_8          --gaattgcgatttaagcggcctcagtggaacaatgAtagcgGgccagcctgtcgttccc
Sbg6_9          taaaaagaaaaaaaaagcggcctcagtggaacaatggtagcgtgccagcctgtcgttccc
Sbg6_10         ---ttttatgatac-agcggcctcagtggaacaatggtagcgtgccagcctgtcgttccc
Sbg6_11         ---aagagtacaga-agcggcctcagtggaacaatggtagcAtgccagcctgtcgttccG
Sbg6_12         ---tcactgtagta-agcggcctcagtggaacaatggtagcgtgccagcctgtcgttccc
                               *********************.**.*. *********** **.
                                        [ 298 bp ]
Consensus_Sbg6  cagcccattagataca----------------------------------
Sbg6_1          cagcccattagatacacacacacaca----------cctaaaagtCg---
Sbg6_2          cagcccattagatacacacacacacacacacaca----ataatggtggc---
Sbg6_3          cagcccattagatacacacacacaca----------tatacaaaatat--
Sbg6_4          cagcccattagatacacacaca--------------ggattagagtcag-
Sbg6_5          cagcccattagatacacacacaca------------cttacttgta----
Sbg6_6          cagcccattagatacacacacaca------------gatcacaaatcca-
Sbg6_7          cagcccattagatacacacccacacaca--------ctaaacataatat-
Sbg6_8          cagcccattagatacacacaca--------------gaattgcgattt--
Sbg6_9          cagcccattagatacacacaca--------------taaaaagaaaaaaa
Sbg6_10         cagcccattagatacacacacacacacacacacacattttatgatac---
Sbg6_11         cagcccatAagatacacacacaca------------aagagtacaga---
Sbg6_12         Tagcccattagatacacacaca--------------tcactgtagta---
                .******* *****
```

Sbg7

A

```
>Consensus_Sbg7 (564b)
ACCGAACGAGATGGCTCATACGGTAGCGTGTGAGGCTCGTATTCGGGAGGTCCCGGGTTCAAACCCGGGGCCGGCCAATCTGATCGGGGTTTTTAGT
GGTTTCCTCGATCTGAAATAACTAATTAGTTGAAAAGGCAAATGCCGGATTGGTTCCCATTCTCCACTATTACCGATATAAGAATTACAGTTTCATG
AGTTCTGCTGTATCCATTGTTGTGTTGTCATAAAACAGTCAATCCTACAGACTACTGCAGAGATTGACTGCTAGATGGCAGCTCTGCAGACTACTCC
GAGATGTGATCTAGATGGCAGCACACAAAGGAATAACCAAAAAGTTCCTCAAGGAAGGCAGTGCCTTTGATCGCAGTGTTCACGGCGCTTCTACGAT
GTTTGAGACTACTCTCATTCATACGTGGGGCGACAGATGTCTCTGCGTGTTGGAGGCCATTGTCACTATGAGAACAAGGAATGAAGTACCATATATC
GATAAACTAATGACCATCATAAGATCAATGAATAGTCGCGATATGACCTCGTTGTTAAAGCGACTATAAATAAACTTGA

Consensus_Sbg7.trna1 (5-77)         Length: 73 bp
Type: Thr          Anticodon: CGT at 34-36 (38-40)     Score: 20.98
Possible pseudogene
         *    |    *    |    *    |    *    |    *    |    *    |    *    |
Seq: AACGAGATGGCTCATACGGTAGCGTGTGAGGCTCGTATTCTGGAGGtCCCGGGTTCAAACCCGGGGCCGGCCA
Str: ..>..>...>>.>........<.<<.>.>>>.......<<<.<.....>>.>>.......<<.<<.<..<...
```

B

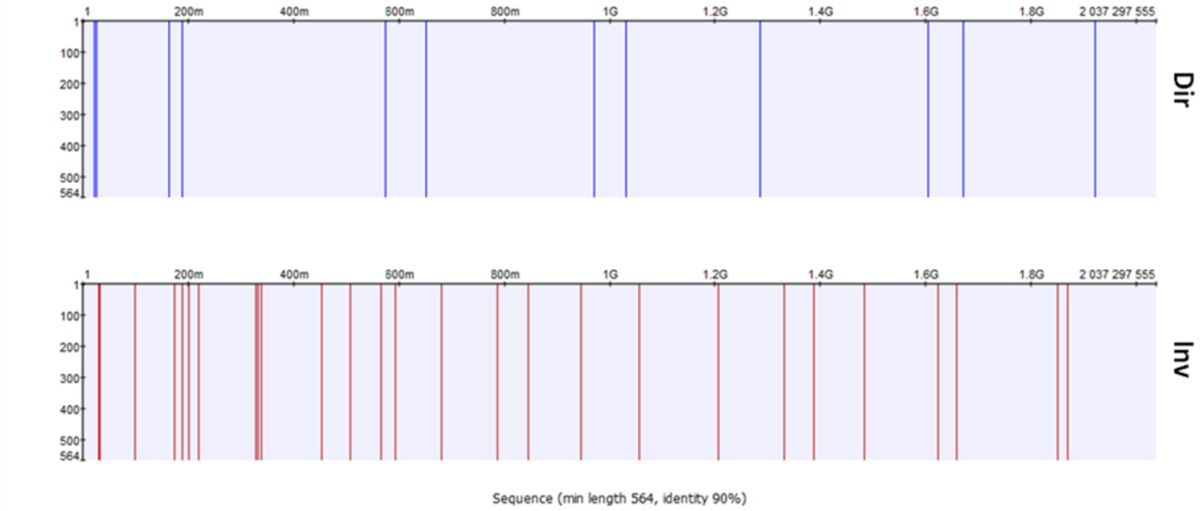

Number of copies: 90% - 40; 80% - 48

C

```
Consensus_Sbg7  -------------accgaacgagatggctcatacggtagcgtgtgaggctcgtattcggg
Sbg7_1          --aaaatttacccaccgaacgagatggctcatacggtagcgtgtgaggctcgtattcggg
Sbg7_2          -aagaactgtagtaccgaacgagatggctcatacggtagcgtgtgaggctcgtattcggg
Sbg7_3          --aacttttctaaccgaacgagatggctcatacggtagcgtgtgaggctcgtattcggg
Sbg7_4          --aaaattatttaccgaacgagatggctcatacggtagcgtgtgaggctcgtattcggg
Sbg7_5          -aagtatttacttaccgaacgagatggctcatacggtagcgtgtgaggctcgtattcggg
Sbg7_6          -aatagcgtagagaccgaacgagatggctcatacggtagcgtgtgaggctcgtattcggg
Sbg7_7          ---aatacagaaaaccgaacgagatggctcatacggtagcgtgtgaggctcgtattcggg
Sbg7_8          --aatttcatccaaccgaacgagatggctcatacggtagcgtgtgaggctcgtattcggg
Sbg7_9          -aatatcatatgAaccgaacgagatggctcatacggtagcgtAtgaggctcgtattcggg
Sbg7_10         --aacaaactgataccgaacgagatggctcatacggtagAgtgtgaggctcgtattcggg
Sbg7_11         aatacctgtttgtaccgaacgagatggctcatacggtagcgtgtgaggctcgtattcggg
Sbg7_12         --aatactttaataccgaacgagatggctcatacggtagcgtgtgaggctcgtattcggg
                             ************************** **.*****************
```

[480 bp]

```
Consensus_Sbg7  atgacctcgttgttaaagcgactataaataaacttga--------------------------
Sbg7_1          atgacctcgttgttaaagcgactataaataaacttgaaaaaaaaaaaaaaaaatttaccc--
Sbg7_2          atgacctTgttgttaaagcgactataaataaacttgaaaaaaaaaaaaaaaagaactgtagt-
Sbg7_3          atgacctcgttgttaaagcgactataaataaacttgaaaaaaaaaaaa--aacttttcta--
Sbg7_4          atgacctcgttgttaaagcgactataaataaacttgaaaaaaa-------aaaattatttt--
Sbg7_5          atgacctcgttgttaaagcgactataaataaacttgaaaaaaa-------aagtatttactt-
Sbg7_6          atgacctcgttgttaaagcgactataaataaacttgaaaaaa--------aatagcgtagag-
Sbg7_7          atgacctcgttgttaaagcgGctataaataaacttgaaaaaaa-------aatacagaaa---
Sbg7_8          atgacctcgttgttaaagcgactataaataaacttgaaaaaaa-------aatttcatcca--
Sbg7_9          atgacctcgttgttaaagcgactataaataaacttgaaaaaaaaa-----aatatcatatG--
Sbg7_10         atgacctcgttgttaaagcgactataaataaacttgaaaaaaa-------aacaaactgat--
Sbg7_11         atgacctcgttgttaaagcgactataaataaacttgaaaaaaaa------aatacctgtttgt
Sbg7_12         atgacctcgttgttaaagcgactataaataaacttgaaaaaaaa------aatactttaat--
                *******.************.****************
```

Sbg8

A

```
>Consensus_Sbg8 (355b)
GTCCCGCACCGTGGCTCAGTGGACTAAGGCAGTGTGCTTTGGTTCCGGGTCACGGGACGCGCGCTGGTTCGAGTCTCCGTGGGGGAAGTAATTTCCT
CATGGATTTCGGCCAGTGTATGGGACCGGTGCCCACCCAGCATCGTGAGGAAAGTGGGGAGCTACGATAGGTAGCGGACTTCGGTTACGGACACTAG
CTTAACGGCTGGGGGAACCGACGTGCTAACCACACGTCACCCCTTCACTGGTTGGATGACGGTTCACCTCCATGTCTTAGGACATGTGTGACCCTGA
GGCCAGCAGCCGGCTGGTAGGCCTAGGCCCTTCATGGGCTGTCGCGCCACGGAGTTAGTTAGTT
```

```
Consensus_Sbg8.trna1 (2-87)  Length: 86 bp
Type: Undet         Anticodon: NNN at 0-0 (0-0)  Score: 21.1
Possible pseudogene
         *    |    *    |    *    |    *    |    *    |    *    |    *    |    *    |    *
Seq: TCCCgcaCCGTGGCTCAGTGGActAAGGCAGTGTGcTTTGGTTCCggGTCACGGGACGcGCGGtGGTTCGAGTCCCCGTGGGGGAA
Str: >>>>...>>>..>>>...........<<<.>......<..>>>>>.......<<<<<...>>>.>>.......<<<<<<<<<<<<.
```

B

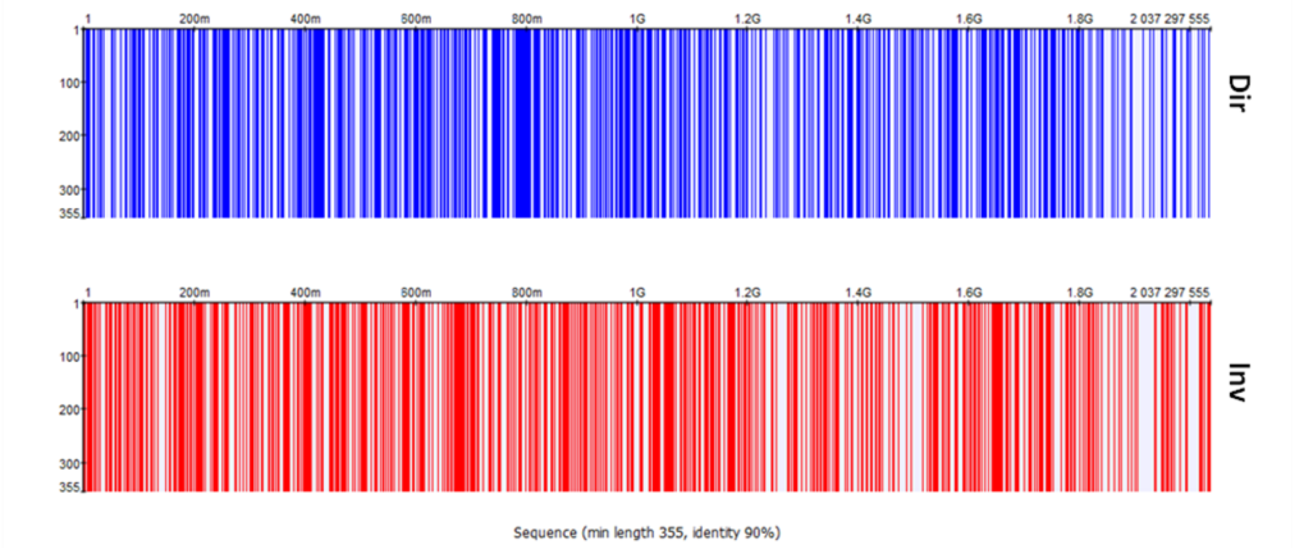

Number of copies: 90% - 1453; 80% - 2219

C

```
Consensus_Sbg8   --------------gtcccgcaccgtggctcagtggactaaggcagtgtgctttggttcc
Sbg8_1           ttatagtgtagagtgtcccgcaccgtggctcagtggactaaggcagtgtgctttggttcc
Sbg8_2           --cttatggaaactgtcccgcaccgtggctcagtggactaaggcagtgtgctttggttcc
Sbg8_3           --tttcgaaactgggtcccgcaccgtggctcagtggactaaggcagtgtgctttggttcc
Sbg8_4           ---tttggcgttcggtcccgcaccgtggctcagtggactaaggcagtgtgctttggttcc
Sbg8_5           -ttatttatacttcgtcccgcaccgtggctcagtggactaaggcagtgtgctttggttcc
Sbg8_6           -agtctattcatgggtcccgcaccgtggctcagtggactaaggcagtgtgctttggttcc
Sbg8_7           ---aacattttatgtcccgcaccgtggctcagtggactaaggcagtgtgctttggttcc
Sbg8_8           --attactttgtcgtcccgcaccgtggctcagtggactaaggcagtgtgctttggttcc
Sbg8_9           -tagtggggaagtgtcccgcaccgtggctcagtggactaaggcagtgtgctttggttcc
Sbg8_10          ---atagtttagaggtcccgcaccgtggctcagtggactaaggcagtgtgctttggttcc
Sbg8_11          --tgatgaatctacgtcccgcaccgtggctcagtggactaaggcagtgtgctttggttcc
Sbg8_12          tatactacagtactgtcccgcaccgtggctcagtggactaaggcagtgtgctttggttcc
                               **********************************************
```

[291 bp]

```
Consensus_Sbg8   ccacggagttagttagtt--------------------------------
Sbg8_1           ccacggagttagttagttagttagttagttagttagttatagtgtagagt
Sbg8_2           ccacggagttagttagtt------------------cttatggaaact--
Sbg8_3           ccacggagttagttagttag----------------tttcgaaactgg--
Sbg8_4           ccacggagttagttagttag----------------tttggcgttcg---
Sbg8_5           ccacggagttagttagttag----------------ttatttatacttc-
Sbg8_6           ccacggagttagttagttagttagtttt--------agtctattcatgg-
Sbg8_7           ccacggagttagttagtt------------------aacattttat---
Sbg8_8           ccacggagttagttagtt------------------attactttgtc--
Sbg8_9           ccacggagttagttagttagttagtt----------tagtggggaagt-
Sbg8_10          ccacggagttagttagtt------------------atagtttagag---
Sbg8_11          ccacggagttagttagttagttag------------tgatgaatctac--
Sbg8_12          ccacggagttagttagttagt---------------tatactacagtact
                 ******************
```

Sbg9 (Sbg1 + Sbg8)

A

```
>Consensus_Sbg9 (658b)
GGGGCACGCGGTAGCCTAGCGGTTACGGCGCTGGACTACAAGCCGGAAGGTCGCGGGTTCGATTCCCGATGGTGCCATTGGAATTTTTCAATTGGTA
TAATCCTTCTGGCCGCACTATGGCCCTGGGGTGCACTCAGCCTCTAACAGAAATGAGTACCAGGAGCTTTCCTGGGGGTAAAGGCGGCTGCCTCGTG
GGGCTGACAACCTCACAGGCCTTGTATGCCGATTGTCTGCAAAGATGGGTGCCGTAACCTCCCGTCACCCTAAGGGCCTTCCTAGGCCTGTATAGGG
ATACCTTTACCTTTGTCCCGCACCGTGGCTCAGTGGACTAAGGCAGTGTGCTTTGGTTCCGGGTCACGGGACGCGCGCTGGTTCGAGTCTCCGTGGG
GGAAGAAATTTCCTCATGGATTTCGGTCAGTGTATGGGACCGGTGCCCACCCAGCATCGTGAGGAAAGTGGGGAGCTACGATAGGTAGCGGACTTCG
GTTACGGATACTAGCTTAACGGCTGGGGGAACCGACGTGCTAACCACACGTCACCCCTTCACTGGTTGGATGACGGTTCACCTCCATGTCTTAGGAC
ATGTGTGACCCTGAGGCCAGCAGCCGGCTGGTAGGCCTAGGCCCTTCATGGGCTGTCGCGCCACGGAGTTAGTTAG
```

B

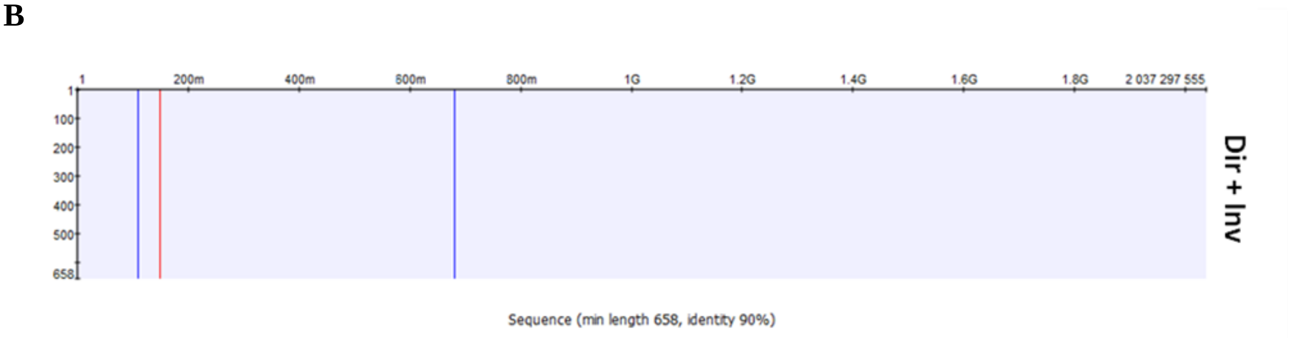

Number of copies: 90% - 3; 80% - 4

C

```
Consensus_Sbg9  -------------ggggcacgcggtagcctagcggttacggcgctggactacaagccgga
Sbg9_1          --taacctatatgggggcacgcggtagcctagcggttacggcgctggactacaagccgga
Sbg9_2          --accttcgagaaggggcacgcggtagcctagcggttacggcgctggactacaagccgga
Sbg9_3          tacatatataccaggggcacgcggtagcctagcggttacggcgctggactacaagccgga
                             ***********************************************

                                  [ 572 bp ]
Consensus_Sbg9  taggcccttcatgggctgtcgcgccacggagttag-ttag--------------------
Sbg9_1          taggcccttcatgggctgtcTcgccacggagttag-ttagttag---taacctatatg--
Sbg9_2          Aaggcccttcatgggctgtcgcgccacggagttag-ttag-------accttcgagaa--
Sbg9_3          taggcccttcatgggctgtcgcgccacggagttagtttagttagttttacatatatacca
                ******************** ************** ****
```

D

```
CLUSTAL format alignment by MAFFT (v7.487)

Consensus_Sbg9   ggggcacgcggtagcctagcggttacggcgctggactacaagccggaaggtcgcgggttc
Consensus_Sbg8   ------------------------------------------------------------
Consensus_Sbg1   ggggcacgcggtagcctagcggttacggcgctggactacaagccggaaggtcggggttc


Consensus_Sbg9   gattcccgatggtgccattggaatttttcaattggtataatccttctggccgcactatgg
Consensus_Sbg8   ------------------------------------------------------------
Consensus_Sbg1   gattccccatggtgccattggaatttttcaattggtataatccttctggccgcactatgg


Consensus_Sbg9   ccctggggtgcactcagcctctaacagaaatgagtaccaggagctttcctgggggtaaag
Consensus_Sbg8   ------------------------------------------------------------
Consensus_Sbg1   ccctggggtgcactcagcctctaacagaaatgagtaccaggagctttcctgggggtgaag



Consensus_Sbg9  gcggctgcctcgtggggctgacaacctcacaggccttgtatgccgattgtctgcaaagat
```

```
Consensus_Sbg8   ------------------------------------------------------------
Consensus_Sbg1   gcggctgcctcgtggggctgacaacctcacaggccctgtatgccgattgtctgtaaagat



Consensus_Sbg9   gggtgccgtaacctcccgtcaccctaagggccttcctaggcctgtat-agggatacctttt
Consensus_Sbg8   ------------------------------------------------------------
Consensus_Sbg1   gggtgccgtaacctcccgtcaccctaagggccttcctaggcctgtataagggatacctttt


Consensus_Sbg9   acctttgtcccgcaccgtggctcagtggactaaggcagtgtgctttggttccgggtcacg
Consensus_Sbg8   ------gtcccgcaccgtggctcagtggactaaggcagtgtgctttggttccgggtcacg
Consensus_Sbg1   acctttt------------------------------------------------------


Consensus_Sbg9   ggacgcgcgctggttcgagtctccgtgggggaagaaatttcctcatggatttcggtcagt
Consensus_Sbg8   ggacgcgcgctggttcgagtctccgtgggggaagtaatttcctcatggatttcggccagt
Consensus_Sbg1   ------------------------------------------------------------


Consensus_Sbg9   gtatgggaccggtgcccacccagcatcgtgaggaaagtggggagctacgataggtagcgg
Consensus_Sbg8   gtatgggaccggtgcccacccagcatcgtgaggaaagtggggagctacgataggtagcgg
Consensus_Sbg1   ------------------------------------------------------------


Consensus_Sbg9   acttcggttacggatactagcttaacggctgggggaaccgacgtgctaaccacacgtcac
Consensus_Sbg8   acttcggttacggacactagcttaacggctgggggaaccgacgtgctaaccacacgtcac
Consensus_Sbg1   ------------------------------------------------------------


Consensus_Sbg9   cccttcactggttggatgacggttcacctccatgtcttaggacatgtgtgaccctgaggc
Consensus_Sbg8   cccttcactggttggatgacggttcacctccatgtcttaggacatgtgtgaccctgaggc
Consensus_Sbg1   ------------------------------------------------------------


Consensus_Sbg9   cagcagccggctggtaggcctaggcccttcatgggctgtcgcgccacggagttagttag--
Consensus_Sbg8   cagcagccggctggtaggcctaggcccttcatgggctgtcgcgccacggagttagttagtt
Consensus_Sbg1   -------------------------------------------------------------
```

Figure S3

Nucleotide sequences and structural features of SINEs of *Blattella germanica* (Sbg1-Sbg9).

(A) – The consensus nucleotide sequence of the corresponding SINE with the designation of the tRNA structure. The tRNA nucleotide sequences are highlighted in gray; “A” and “B” boxes are highlighted in blue; nucleotides other than canonical nucleotides are highlighted in yellow background and red font (explanation in the text). (**B)** – Distribution of the corresponding SINE copies in the *B. germanica* genome. Blue vertical lines indicate the positions of SINE localized in direct orientation, red vertical lines - in inverted orientation. (**C)** – The result of the alignment of the consensus sequences and the sequences of twelve similar to the consensus sequence SINE copies presented in the genome, along with the nearest environment. Direct repeats flanking the retrotransposon are highlighted with a green background. Poly(A) sequences and short

microsatellite repeats are highlighted in yellow, blue and pink backgrounds. Variable nucleotides are highlighted in red fonts. (**D)** – Alignment of Sbg1, Sbg8 and Sbg9, demonstrating that Sbg9 was formed by combining Sbg1 and Sbg8.
