# Supplementary material for: Identification and functional characterization of the German cockroach, Blattella germanica, short interspersed nuclear elements
Source: PLoS One. 2022 Jun 13;17(6):e0266699. doi: 10.1371/journal.pone.0266699 (PMC9191728; doi:10.1371/journal.pone.0266699)
Supplement: S4 Fig — The consensus nucleotide sequences of the corresponding SINEs with the designation of the tRNA structure are shown. The tRNA nucleotide sequences are highlighted in gray; “A” and “B” boxes are highlighted in yellow; nucleotides other than canonical are highlighted in red font. The distances from the start of transcription of SINEs to the corresponding “A” boxes are highlighted in pink. (PDF) [file pone.0266699.s007.pdf]

```
>Talua  SINE2/tRNA     Reticulitermes lucifugus (11 b)
gccgatcccagtggccgcgcggtctaaggcgtgggtctgcggccgctcgcttactggattgtgggttcgaatcccgccgggggcatggatgtctgt
ctcttgtgagtgttgtgtgttgtcaggtagaggtctctgcgacgggctgatcactcgtccagaggagtcctaccgagtgtggtgtgtctgagtgtga
tcgtgaagcctcgataatgaggaggccctaggccccctagggctgttgagccatgggaa
```

```
2.trna1 (5-86)     Length: 82 bp
Type: Arg Anticodon: GCG at 35-37 (39-41)       Score: 20.45
Possible intron: 39-46 (43-50)
Possible pseudogene
         *    |    *    |    *    |    *    |    *    |    *    |    *    |    *    |
Seq: ATCCCAGTGGCCGCGCGGTctAAGGCGTGGGTCTGCGGccgctcgcTTACTGGGAtTGTGGGTTCGAATCCCGCCGGGGGCA
Str: .>>>>.>..>>>...........<<<.>>>...................<<<.....>>>>>.......<<<<<<.<<<<..
```

```
>Talub  SINE2/tRNA     Reticulitermes lucifugus (10 b)
ggccgtgccatggctcaggcggttagtcgccggcctctcaccgcggaggcccgggttcga_tcccgggtcagtccatgtgggatttgtggtggacaa
agtggcactgggacaggttttccccgagtacttcggttttcccctgtcaatttcattccaccggtgctccattacttggaaaaacgaaaaaaacta
atcattcatcttcatcacagggttgcacaataagccttaaggctgcggtgcgtccgtagcatctgctgcggggcccttcaccacaaaaaaaaa
```

```
2.trna1 (5-75)     Length: 71 bp
Type: Glu Anticodon: CTC at 33-35 (37-39)       Score: 21.02
Possible pseudogene
         *    |    *    |    *    |    *    |    *    |    *    |    *    |
Seq: GTGCCATGGCTCAGGCGGTtAGTCGCCGGCCTCTCACCGCGGAGGCCCGGGTTCGATCCCGGGTCAGTCCA
Str: >.>>.>..>..>.........<.<.>>>...........<<<....>>.>>.......<<.<<<..<<.<.
```

```
>Taluc  SINE2/tRNA     Reticulitermes lucifugus (11 b)
gtcgccctcggtggactagtggttagcgtgctcgccactggacccaaggttcgcgggttcga_tcccgaccgaggtcgatggattttaagggtgat
aaaaatccgtagcaccacttccttcggaggggaagtaaagccgtcggtcccatgtcgtagatttacggcatgtaaaagaaccttacgggcatgaata
gaaatgtttcgtaagcaaaattcagcggccatttctcacccaagtctcctgactgcctgccagatggctctggcagtcacatcaggattgagtagaa
cttgtgcggtcagtgggctggtcacccactacangaatgcacangacctagaaacctcggaaagngaggcccnagcccatatagggtggagccatgg
```

```
2.trna1 (5-76)     Length: 72 bp
Type: Gln Anticodon: CTG at 34-36 (38-40)       Score: 21.47
Possible pseudogene
         *    |    *    |    *    |    *    |    *    |    *    |    *    |
Seq: CCCTCGGTGGACTAGTGGTtAGCGTGCTCGCCACTGGACCCAAGGttCGCGGGTTCGATCCCGACCGAGGTC
Str: .>>>>>>..>..>........<..<..>.>.........<.<.......>>>>......<<<<.<<<<<<..
```

```
>Talud  SINE2/tRNA     Reticulitermes lucifugus (5 b)
ggcggtagcgtagtnggtatagcgactcgctacgggctggacggtccggggttcgaatcccggtggggtcaagaattcttcnncatccgtccagacc
ggccctggggcccacccagcctcctatacaatgggtaccgggctctttcccgggggtaaagcggccggtgcgtggngttgaccacccacccccatct
agtgccgaggtcaagaaagagtggagcctcaactccctaagggcctgtccaggcctgtaatgggattctttctttcttt
```

```
2.trna1 (1-71)     Length: 71 bp
Type: Arg Anticodon: ACG at 32-34 (32-34)       Score: 20.26
Possible pseudogene
         *    |    *    |    *    |    *    |    *    |    *    |    *    |
Seq: GGCGGTAGCGTAGTNGGtATAGCGACTCGCTACGGGCTGGACGGtCCGGGGTTCGAATCCCGGTGGGGTCA
Str: >>>..>>>>............<<..>>.>.......<.<<......>.>>>.......<<<.<<<..<<<.
```

Figure S4.

Nucleotide sequences and structural features of SINEs of *Reticulitermes lucifugus* (Talua. Talub, Taluc and Talud).

The consensus nucleotide sequences of the corresponding SINEs with the designation of the tRNA structure are shown. The tRNA nucleotide sequences are highlighted in gray; "A" and "B" boxes are highlighted in yellow; nucleotides other than canonical nucleotides are highlighted in red font.

The distances from the start of transcription of SINEs to the corresponding "A" boxes are highlighted in pink.
