# Supplementary material for: Identification and functional characterization of the German cockroach, Blattella germanica, short interspersed nuclear elements
Source: PLoS One. 2022 Jun 13;17(6):e0266699. doi: 10.1371/journal.pone.0266699 (PMC9191728; doi:10.1371/journal.pone.0266699)
Supplement: S5 Fig — (A)–Masked Sbg8 sequence and the local sequence alignment of Sbg8 and RTE retrotransposons of Locusta migratoria. (B)–Sequence of RTE retrotransposon of L. migratoria. The sequence fragment similar to the 3’-end of Sbg8 is highlighted in blue. (PDF) [file pone.0266699.s008.pdf]

A

Name	From	To	Name	From	To	Class	Dir	Sim	Pos/Mm:Ts	Score
Consensus_Sbg8	310	354	RTE-34_LMi	2295	2339	NonLTR/RTE	d	0.8667	99.0000	278

Masked Sequence

```
>Consensus_Sbg8
GTCCCGCACCGTGGCTCAGTGGACTAAGGCAGTGTGCTTTGGTTCCGGGTCACGGGACGCGCGCTGGTTC
GAGTCTCCGTGGGGGAAGTAATTTCCTCATGGATTTCGGCCAGTGTATGGGACCGGTGCCCACCCAGCAT
CGTGAGGAAAGTGGGGAGCTACGATAGGTAGCGGACTTCGGTTACGGACACTAGCTTAACGGCTGGGGA
ACCGACGTGCTAACCACACGTCACCCCTTCACTGGTTGGATGACGGTTCACCTCCATGTCTTAGGACATG
TGTGACCCTGAGGCCAGCAGCCGGCTGGTXXXXXXXXXXXXXXXXXXXXXXXXXXXXXXXXXXXXXXXXX
XXXXT
```

Local Alignments*

Name	From	To	Name	From	To	Dir	Sim	Pos/Mm:Ts	Score
Consensus_Sbg8	310	354	RTE-34_LMi	2295	2339	d	0.8667	99.0000	278

```
 310 AGGCCTAGGCCCTTCATGGGCTGTCGCGCCACGGAGTTAGTTAGT 354
     ||||| ||||||| |||||||||| |||||| ||||| ||| |||
2295 AGGCCAAGGCCCTACATGGGCTGTAGCGCCAAGGAGTAAGTAAGT 2339
```

Masked Regions

```
>Consensus_Sbg8 FRAGMENT 310 -> 354
AGGCCTAGGCCCTTCATGGGCTGTCGCGCCACGGAGTTAGTTAGT
```

B

```
>RTE-34_LMi    RTE    Locusta migratoria
tagagaaacagtaggaaatgaaaaagcctgcatgaggtgtcaaagactataactttgccatgggcaatgcagtggtagtgaaggtacatggctccagggaaacacaggaaagcacacggtgctcgccaggcactacttttaatcaaactga
cattggtaatcacaggggcatgcaccgattgatagctgcagggaggtgaccgatctttatatggcaagatggaatacacacgagatccagaggaagggaaagctctgaagacacaatgtttccaaattgaaggaagggagccaagccaga
agcatcagggggagtacgattaaagaagctctacaagaaactgcagaagaagttttaggcttagaagaaagagtgaaaagggcagatggtacgatgaagattgtaaaaaggctgtggaagaaagaaatgaggcaaggaagaaaatgataa
acaggacaacaagatctactacagaaaattcatgaagaaaggaaagaggcagataggatttgcagaagaaagaaacgagaattagagagggatggattatggagctagagaagagtacaatgatgaagaagtaagaaaattctacaagaa
agttcgggaactgaagaaaggcttccaaccacgtactctgcagagataaagaaggaaattttataggaggagaagaacaggttctagaccaatgggccaaatatttcagtgagctgctgaatgtcactacagaaagagaagacaatgaaa
cagagggtgaccaaccgtggcaaaatgttagtcaggatgaaagacctgtacctataccaacaatagaggaagttaggaaggcaattaaaagcctaggaataataaggctccgggtaatgacaatatttcagcagagatgatcaagggcag
aggtgaacatctaacaagattggtacatcagctgatagtggaaatatgggaaaaggaagagatgccagaccagtggaacatggccattatatgcccgattcacaagaagaaggacaaagcaaattgtgaaactatcgcggcattgcatta
gtcagtgtggtgtacaaagtattagccaaagtcattgctatgagatttgcaccaattacagaagaaatactcaggagactaccagtgcggctttcggcagggcagatcaacaacagaccaaattttcattataaggcaaataatggaaaag
```

tgttatgaatataatgtggatgtacaccagctttttgtagacttcaaacaggcctatgatagtgttaaaaggaaaaagctgtatcagaccttaatggagatggaattcccaagtaaactaatcagactggtacaaatgaccctcaccagca
caaaatgccaagtaaaaatagaaggaaaactctctaaagaatttgaggtcaaccaagggttaagacagggtgatgtattatccacgctactcttcaacatagtgttggagagagtgatgcgaagggtggagattgataacccaggtggcac
tctgtttaaccgtatgaaccaaaatctagcatatgtcgatgatgtgtgtatgctgtctagaagattgaaagaactggaagaaggatttgacagattagaaaaggaggcagaacagttgggagttaacaacaccaaaacacagtatctcttt
gcttccaggaaaaatgctgttgttaaagagaaatatattaagttgaacagaggcaattatgagagatgtgagaagtttcaatcctggggtgctgttacggaggatagcagataagagaagaaataaaatcaaggattgctgcgggaaatag
agcttactgggccatgatcaagatccttcagtcacgtagcctgagcagaaaatcgaaggtgactgtctatmgaacagtcataagaccggtggtaatgtatggctcagaaacatggacaatgactgtagcagaagaggagctcttgagaaga
tgggagaggaaaatattaagaaagatttttggagccgtgattgaggtgggacaatggagaatacggagaaacaaggaattggaagaactgtatagaaatctgacttagtgactgaaattaagtgtaacagattgagatggctgggccatgt
agagcgaatgtcggaggatcgagctgttaaaaaggtctacaaggaaatccaggtgaaagaagcaagggaggcctcggaaacgatggctggaggatgtggaaaacgatttgagaaagatgggagtaggagatggaggaaacgagcagaaaa
tcgtcaggattgggctgcgatcatcagagaggccaaggccctacatgggctgtagcgccaaggagtaagtaagta

Figure S5 The result of the comparison of the consensus Sbg8 nucleotide sequence with the sequences presented in the RepBase database (https://www.girinst.org). (A) – Masked Sbg8 sequence and the local sequence alignment of Sbg8 and RTE retrotransposons of *Locusta migratoria*. **(B)** – Sequence of RTE retrotransposon of *L. migratoria*. The sequence fragment similar to the 3'-end of Sbg8 is highlighted in blue.
