# Supplementary material for: Identification and functional characterization of the German cockroach, Blattella germanica, short interspersed nuclear elements
Source: PLoS One. 2022 Jun 13;17(6):e0266699. doi: 10.1371/journal.pone.0266699 (PMC9191728; doi:10.1371/journal.pone.0266699)
Supplement: S6 Fig — Reads mapped in direct orientation are highlighted in blue, and reads mapped in reverse complement orientation are green. (PDF) [file pone.0266699.s009.pdf]

## Sbg1

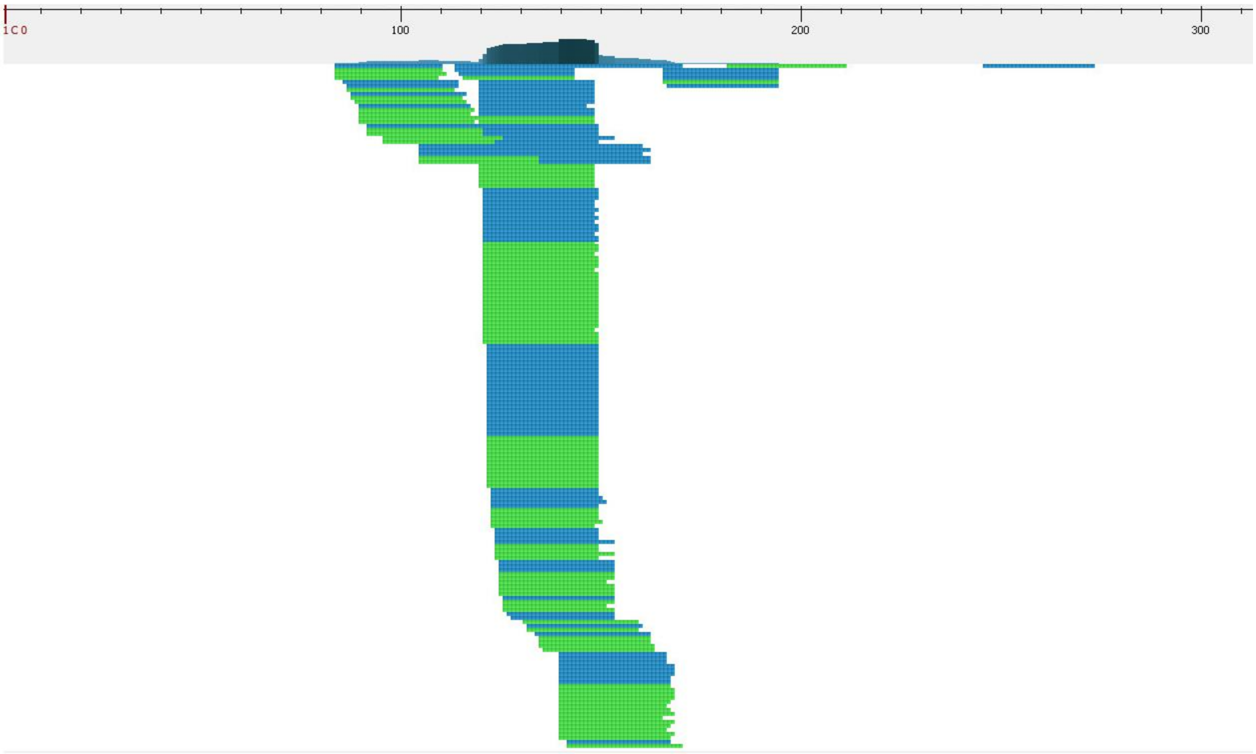


Total number of reads – 205; Orientation: direct – 100 reads, reverse complement – 105 reads. The number of reads per 100 bases of SINE sequence per one thousand mapped reads – 7,23.

## Sbg2

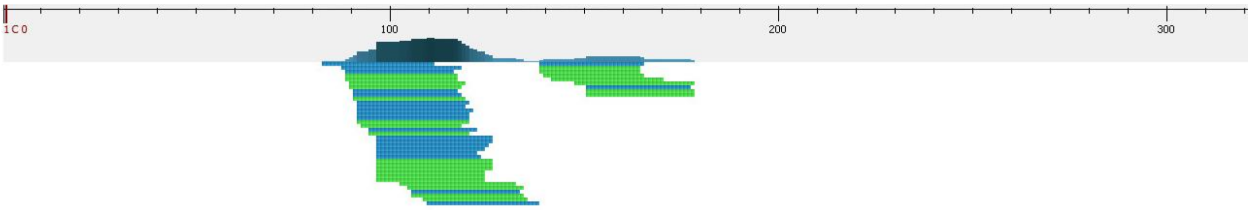


Total number of reads – 46; Orientation: direct – 21 reads, reverse complement – 25 reads. The number of reads per 100 bases of SINE sequence per one thousand mapped reads – 1,59.

**Sbg4**

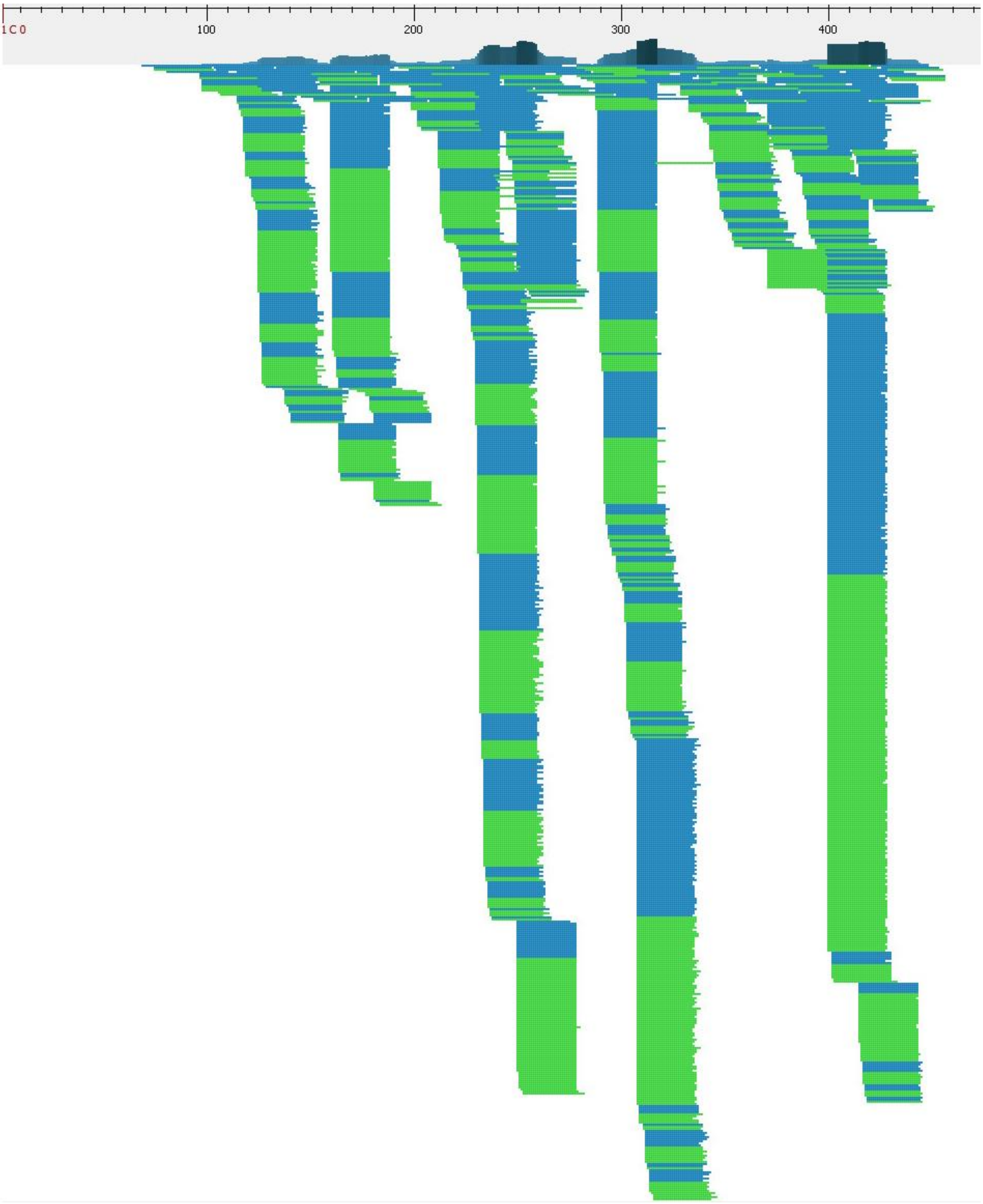


Total number of reads – 2290; Orientation: direct – 1109 reads, reverse complement – 1181 reads.

The number of reads per 100 bases of SINE sequence per one thousand mapped reads – 54,23.

## Sbg5

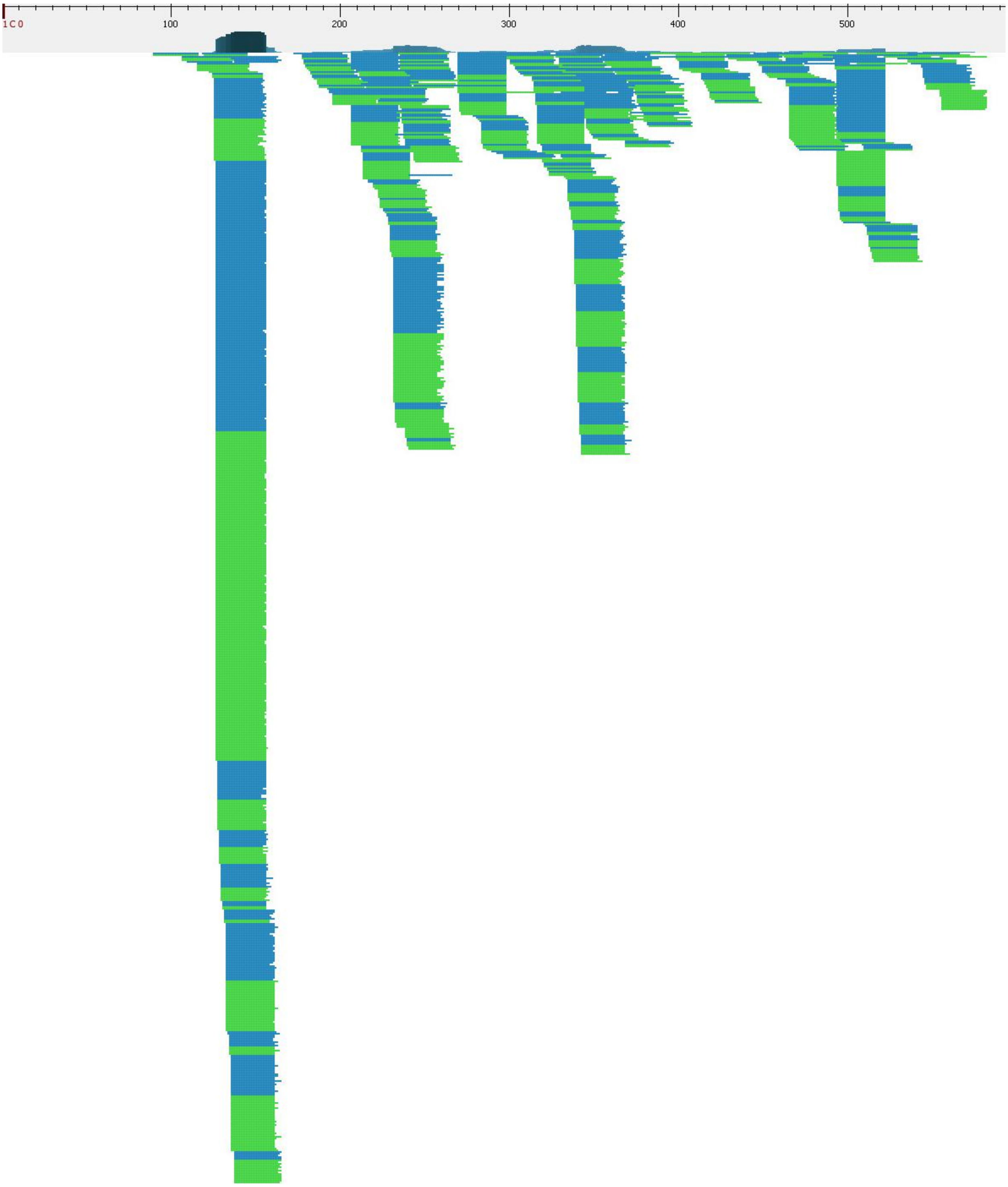


Total number of reads – 1653; Orientation: direct – 805 reads, reverse complement – 848 reads. The number of reads per 100 bases of SINE sequence per one thousand mapped reads – 30,67.

**Sbg6**

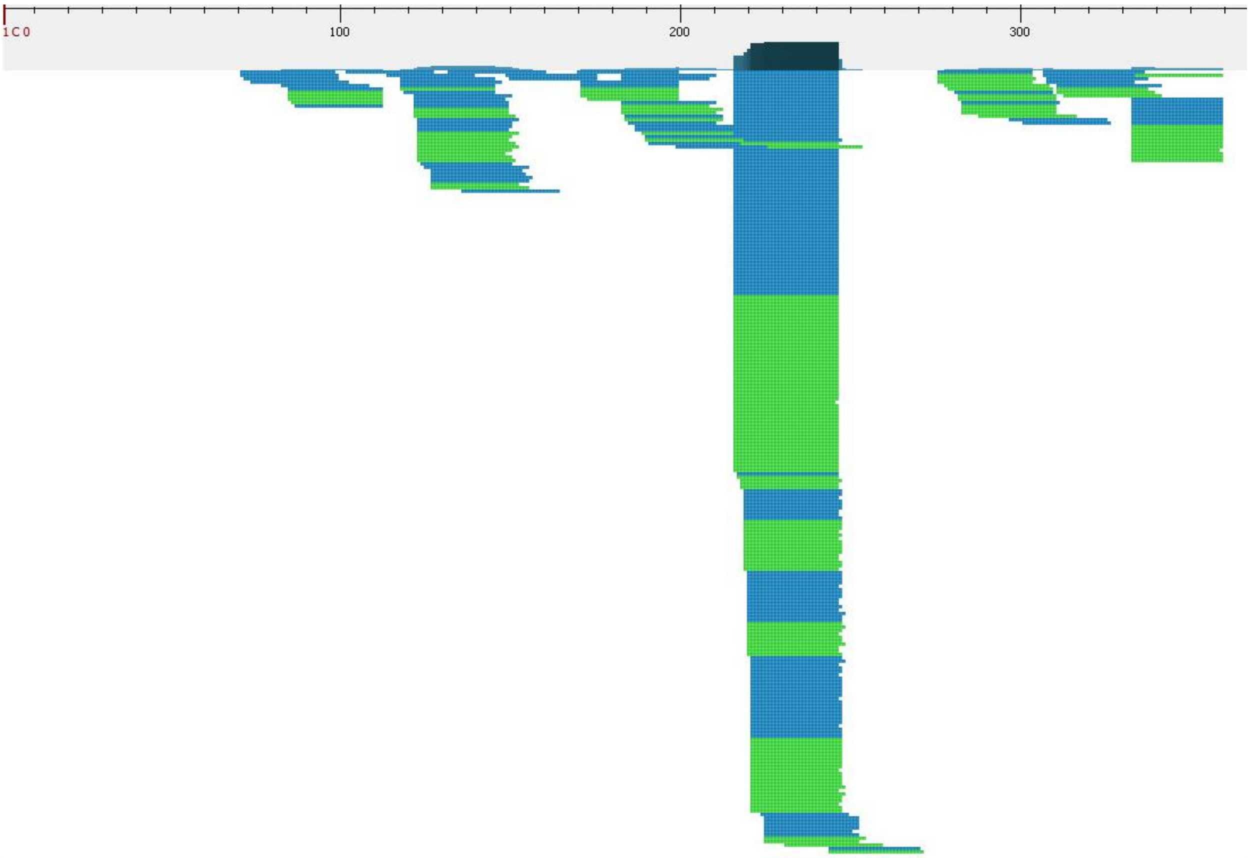


Total number of reads – 347; Orientation: direct – 183 reads, reverse complement – 164 reads. The number of reads per 100 bases of SINE sequence per one thousand mapped reads – 10,44.

**Sbg8**

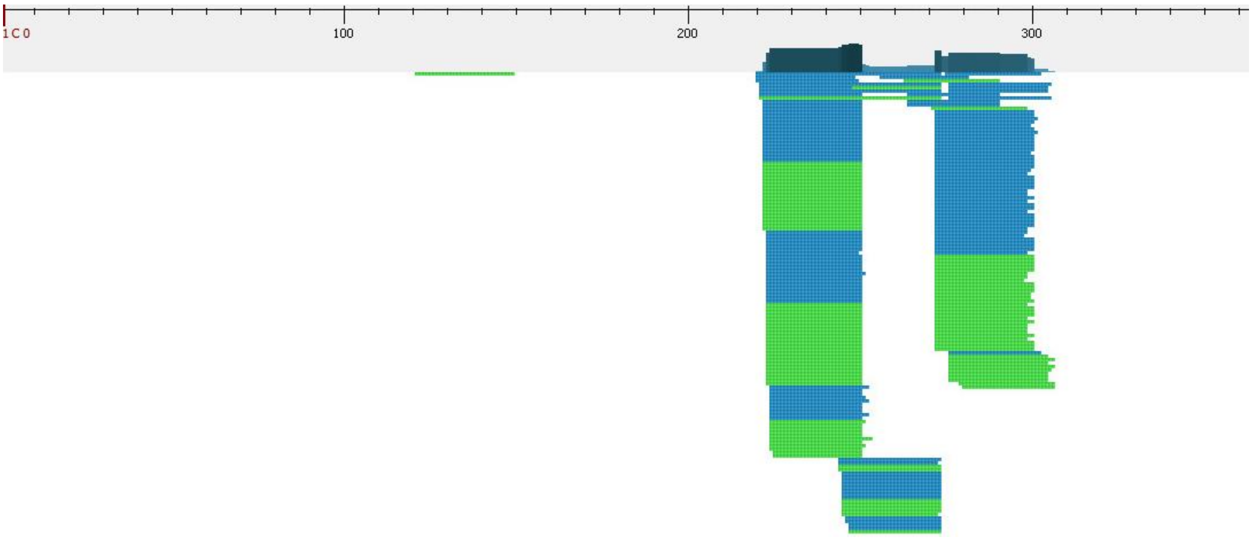


Total number of reads – 232; Orientation: direct – 125 reads, reverse complement – 107 reads. The number of reads per 100 bases of SINE sequence per one thousand mapped reads – 7,06.

## Figure S6

The result of piRNA reads mapping to the Sbg1, Sbg3 – Sbg6, and Sbg8 consensus sequences. Reads mapped in direct orientation are highlighted in blue, and reads mapped in reverse complement orientation are green.
