# Supplementary material for: Identification and functional characterization of the German cockroach, Blattella germanica, short interspersed nuclear elements
Source: PLoS One. 2022 Jun 13;17(6):e0266699. doi: 10.1371/journal.pone.0266699 (PMC9191728; doi:10.1371/journal.pone.0266699)
Supplement: S1 Table — F and R represent forward and reverse complement orientations, respectively. (PDF) [file pone.0266699.s010.pdf]

Table S1. The analysis of 100 genes, for each of which 1000 or more piRNA reads were mapped.

Contig ID	Strand	Start	End	Gene ID	Gene full name	Reads count
PYGN01000005	R	832095	893324	C1TC	C-1-tetrahydrofolate synthase	3557 (F - 1798; R - 1759)
PYGN01000073	F	440373	516715	LAC_1	Lachesin	2978 (F - 1450; R - 1528)
PYGN01000033	F	355625	439785	tdc-1_0	Tyrosine decarboxylase	2889 (F - 1547; R - 1342)
PYGN01000033	F	656364	694669	K11H3-3	Tricarboxylate transport protein	2717 (F - 1343; R - 1374)
PYGN01000022	F	2406772	2456620	lhx3	LIM/homeobox protein Lhx3	2716 (F - 1396; R - 1320)
PYGN01000072	R	549086	581815	Diap1	Death-associated inhibitor of apoptosis 1	2655 (F - 1375; R - 1280)
PYGN01000005	R	2480629	2498464	Or88	Odorant receptor 88	2488 (F - 1127; R - 1361)
PYGN01000049	R	1701575	1765331	Cher_2	Filamin-A	2478 (F - 1267; R - 1211)
PYGN01000083	R	266347	296944	Pex1	Peroxisome biogenesis factor 1	2469 (F - 1167; R - 1302)
PYGN01000050	R	461156	514648	Ir76b	Ionotropic receptor 76b	2433 (F - 1150; R - 1283)
PYGN01000086	R	1254016	1267262	SCD	Acyl-CoA desaturase	2416 (F - 1247; R - 1169)
PYGN01000005	F	3153139	3200419	IFT140	Intraflagellar transport protein 140	2414 (F - 1183; R - 1231)
PYGN01000060	F	519801	563025	APN1_0	Aminopeptidase N	2400 (F - 1127; R - 1273)
PYGN01000016	F	1455716	1479561	Ir41a12	Ionotropic receptor 41a12	2290 (F - 1109; R - 1181)
PYGN01000056	F	343658	370413	Rcbtb1	RCC1 and BTB domain-containing protein 1	2290 (F - 1181; R - 1109)
PYGN01000029	R	1139008	1166057	March6	E3 ubiquitin-protein ligase MARCH6	2104 (F - 1032; R - 1072)
PYGN01000024	R	2118061	2140175	CCND2	G1/S-specific cyclin-D2	2081 (F - 1089; R - 992)
PYGN01000005	F	1745668	1812688	Tle4_1	Transducin-like enhancer protein 4	2072 (F - 1020; R - 1052)
PYGN01000010	F	1604309	1616070	Smg9	Protein SMG9	2055 (F - 999; R - 1056)
PYGN01000038	F	2126408	2158714	HIRA	Protein HIRA	2047 (F - 1051; R - 996)
PYGN01000040	R	147779	221540	Mpp7	MAGUK p55 subfamily member 7	2041 (F - 994; R - 1047)
PYGN01000071	F	1149544	1172943	Nrf-6	Nose resistant to fluoxetine protein 6	2024 (F - 1037; R - 987)
PYGN01000025	R	3259549	3335016	RpL44	60S ribosomal protein L44	1991 (F - 1024; R - 967)
PYGN01000008	F	4166658	4207307	AGO3	Piwi-like protein Ago3	1966 (F - 1024; R - 942)
PYGN01000051	F	1474330	1553401	SEC31A	Protein transport protein Sec31A	1909 (F - 947; R - 962)
PYGN01000021	F	841764	864513	Prpf3	U4/U6 small nuclear ribonucleoprotein Prp3	1890 (F - 930; R - 960)
PYGN01000012	R	980891	1006400	Nfxl1	NF-X1-type zinc finger protein NFXL1	1886 (F - 841; R - 1045)

PYGN01000034	F	237723	271180	Ptprr	Receptor-type tyrosine-protein phosphatase R	1871 (F - 985; R - 886)
PYGN01000026	R	1307483	1365427	SYG	Glycine--tRNA ligase	1854 (F - 952; R - 902)
PYGN01000022	R	1897462	2016583	Ddc_3	Aromatic-L-amino-acid decarboxylase	1827 (F - 845; R - 982)
PYGN01000041	R	245473	259506	FABP4	Fatty acid-binding protein	1806 (F - 920; R - 886)
PYGN01000061	F	1035760	1072769	Gpt2	Alanine aminotransferase 2	1801 (F - 908; R - 893)
PYGN01000083	F	1449655	1480506	Nop60B	H/ACA ribonucleoprotein complex subunit 4	1798 (F - 924; R - 874)
PYGN01000012	F	2106895	2139363	Sgl	UDP-glucose 6-dehydrogenase	1789 (F - 881; R - 908)
PYGN01000016	F	1421731	1449049	Ir41a11	Ionotropic receptor 41a11	1788 (F - 855; R - 933)
PYGN01000013	R	530387	565339	TBC1D22B	TBC1 domain family member 22B	1761 (F - 780; R - 981)
PYGN01000099	F	1353107	1411101	Ggt1_1	Gamma-glutamyltranspeptidase 1	1760 (F - 858; R - 902)
PYGN01000023	F	3241268	3269327	Fbln2	Fibulin-2	1758 (F - 787; R - 971)
PYGN01000037	R	942452	1000989	CDK11B	Cyclin-dependent kinase 11B	1739 (F - 848; R - 891)
PYGN01000035	F	1813764	1864068	Pu	GTP cyclohydrolase 1	1699 (F - 834; R - 865)
PYGN01000019	F	51330	92695	TOP3B	DNA topoisomerase 3-beta-1	1690 (F - 887; R - 803)
PYGN01000092	R	634794	661561	Sca	Protein scabrous	1686 (F - 942; R - 744)
PYGN01000072	F	1155947	1208398	SIM1	Single-minded 1	1684 (F - 850; R - 834)
PYGN01000075	F	1983373	2014907	ACO11_3	Acyl-CoA Delta(11) desaturase	1624 (F - 806; R - 818)
PYGN01000049	R	2432452	2491455	RhoGAPp190	Rho GTPase-activating protein 190	1614 (F - 836; R - 778)
PYGN01000008	F	4118100	4148806	For	cGMP-dependent protein kinase	1588 (F - 833; R - 755)
PYGN01000069	R	2563669	2566278	FZD1	Frizzled-1	1574 (F - 745; R - 829)
PYGN01000051	R	2107759	2186041	Shaw_1	Potassium voltage-gated channel protein Shaw	1561 (F - 765; R - 796)
PYGN01000059	R	547145	581037	amdhd1	Amidazolonepropionase	1544 (F - 777; R - 767)
PYGN01000035	F	289350	312563	Ap2b1	AP-2 complex subunit beta	1531 (F - 756; R - 775)
PYGN01000065	F	233426	255319	Ir75a	Ionotropic receptor	1527 (F - 721; R - 806)
PYGN01000060	R	1448745	1475474	BBS1	Bardet-Biedl syndrome 1 protein	1527 (F - 787; R - 740)
PYGN01000082	R	541742	547023	SLC5A8_2	Sodium-coupled monocarboxylate transporter 1	1521 (F - 732; R - 789)
PYGN01000010	F	1771174	1791444	CTSC	Dipeptidyl peptidase 1	1505 (F - 780; R - 725)
PYGN01000082	R	215833	239014	SLC5A8_0	Sodium-coupled monocarboxylate transporter 1	1449 (F - 693; R - 756)
PYGN01000041	R	206462	229223	Mlec-a	Malectin-A	1440 (F - 697; R - 743)
PYGN01000024	R	947969	975059	Drc7	Dynein regulatory complex subunit 7	1439 (F - 703; R - 736)
PYGN01000075	F	1957464	1984125	ACO11_1	Acyl-CoA Delta(11) desaturase	1428 (F - 691; R - 737)

PYGN01000026	F	2324292	2346021	NDUBA	NADH dehydrogenase [ubiquinone] 1 beta	1421 (F - 687; R - 734)
PYGN01000026	R	2324364	2328765	Rrs1_0	Ribosome biogenesis regulatory protein	1421 (F - 674; R - 747)
PYGN01000056	F	3513432	3547183	Taf6	Transcription initiation factor TFIID subunit 6	1409 (F - 672; R - 737)
PYGN01000082	R	406406	448300	SLC5A12_3	Sodium-coupled monocarboxylate transporter 2	1399 (F - 744; R - 655)
PYGN01000038	F	2342042	2360391	TRPC5	Short transient receptor potential channel 5	1395 (F - 670; R - 725)
PYGN01000079	R	1895059	1901826	BMP10	Bone morphogenetic protein 10	1392 (F - 672; R - 720)
PYGN01000008	F	4578967	4640152	P115	General vesicular transport factor p115	1381 (F - 799; R - 582)
PYGN01000092	R	380948	422907	Ift122	Intraflagellar transport protein 122	1376 (F - 620; R - 756)
PYGN01000076	R	2467920	2499119	ACE_2	Angiotensin-converting enzyme	1373 (F - 596; R - 777)
PYGN01000079	R	993916	1012363	WDR48	WD repeat-containing protein 48	1362 (F - 655; R - 707)
PYGN01000079	R	1221090	1241997	Rim2_1	Mitochondrial carrier protein Rim2	1357 (F - 701; R - 656)
PYGN01000035	R	733524	751228	HMCN1	Hemicentin-1	1349 (F - 702; R - 647)
PYGN01000097	F	590693	597444	Fcp3C	Follicle cell protein 3C-1	1337 (F - 659; R - 678)
PYGN01000046	F	132349	230906	MFSD14A	Hippocampus abundant transcript 1 protein	1334 (F - 672; R - 662)
PYGN01000056	R	104417	118332	Rraga	Ras-related GTP-binding protein A	1328 (F - 681; R - 647)
PYGN01000014	R	2572191	2595882	Itpkb	Inositol-trisphosphate 3-kinase B	1322 (F - 689; R - 633)
PYGN01000038	F	1714417	1734951	Bi	Optomotor-blind protein	1311 (F - 679; R - 632)
PYGN01000012	R	858678	887912	CNDP2_2	Cytosolic non-specific dipeptidase	1301 (F - 645; R - 656)
PYGN01000014	R	1097754	1168458	Aldh18a1	Delta-1-pyrroline-5-carboxylate synthase	1300 (F - 612; R - 688)
PYGN01000058	R	650952	690564	Tre1	Protein trapped in endoderm-1	1294 (F - 553; R - 741)
PYGN01000043	R	2433536	2459100	Alas2	5-aminolevulinate synthase	1282 (F - 646; R - 636)
PYGN01000048	F	2638821	2674214	Glg1	Golgi apparatus protein 1	1257 (F - 671; R - 586)
PYGN01000017	R	1727722	1747322	Rab11fip4a	Rab11 family-interacting protein 4A	1256 (F - 597; R - 659)
PYGN01000035	F	1160198	1182147	SLC9B2_2	Sodium/hydrogen exchanger 9B2	1225 (F - 619; R - 606)
PYGN01000034	R	1075982	1103469	Orb2	Translational regulator orb2	1196 (F - 590; R - 606)
PYGN01000023	R	1961596	1973550	Dnah12_1	Dynein heavy chain 12	1195 (F - 586; R - 609)
PYGN01000002	R	4134265	4178946	PyK_1	Pyruvate kinase	1192 (F - 619; R - 573)
PYGN01000018	R	420344	463097	Gyc89Db	Soluble guanylate cyclase 89Db	1182 (F - 559; R - 623)
PYGN01000001	R	2987687	3043430	Ptp99A	Tyrosine-protein phosphatase 99A	1145 (F - 548; R - 597)
PYGN01000008	R	2388877	2419707	CRPI_0	Allergen Cr-PI	1141 (F - 616; R - 525)
PYGN01000011	F	272897	282681	Mkln1_1	Muskelin	1136 (F - 574; R - 562)

PYGN01000012	F	662188	684584	TCF25	Transcription factor 25	1117 (F - 575; R - 542)
PYGN01000097	F	1320003	1346178	ANPEP_1	Aminopeptidase N	1103 (F - 548; R - 555)
PYGN01000045	F	1692950	1702026	Mpc1	Mitochondrial pyruvate carrier 1	1076 (F - 529; R - 547)
PYGN01000082	R	1108388	1143374	Ntm	Neurotrimin	1075 (F - 573; R - 502)
PYGN01000079	F	1569224	1617001	SamDC	S-adenosylmethionine decarboxylase proenzyme	1070 (F - 575; R - 495)
PYGN01000058	R	1687233	1724488	HMGCS-2	Hydroxymethylglutaryl-CoA synthase 2	1066 (F - 509; R - 557)
PYGN01000057	F	3438456	3512808	Vas_1	ATP-dependent RNA helicase vasa	1064 (F - 530; R - 534)
PYGN01000025	R	601250	673160	Celf2	CUGBP Elav-like family member 2	1050 (F - 515; R - 535)
PYGN01000058	R	1790496	1869956	Ptp69D	Tyrosine-protein phosphatase 69D	1044 (F - 530; R - 514)
PYGN01000090	R	881804	900752	Bowl_1	Protein bowel	1036 (F - 537; R - 499)
PYGN01000005	F	3854345	3877861	ADSL	Adenylosuccinate lyase	1000 (F - 497; R - 503)

F and **R** represent forward and reverse complement orientations.
